# Decarboxylative Cross-Coupling Enabled by Fe and Ni Metallaphotoredox Catalysis

**DOI:** 10.1021/jacs.4c09621

**Published:** 2024-10-18

**Authors:** Reem Nsouli, Sneha Nayak, Venkadesh Balakrishnan, Jung-Ying Lin, Benjamin K. Chi, Hannah G. Ford, Andrew V. Tran, Ilia A. Guzei, John Bacsa, Nicholas R. Armada, Fedor Zenov, Daniel J. Weix, Laura K. G. Ackerman-Biegasiewicz

**Affiliations:** † Department of Chemistry, Emory University, Atlanta, Georgia 30322, United States; ‡ Department of Chemistry, University of Wisconsin-Madison, Madison, Wisconsin 53716, United States; § School of Molecular Science, Arizona State University, Tempe, Arizona 85281, United States

## Abstract

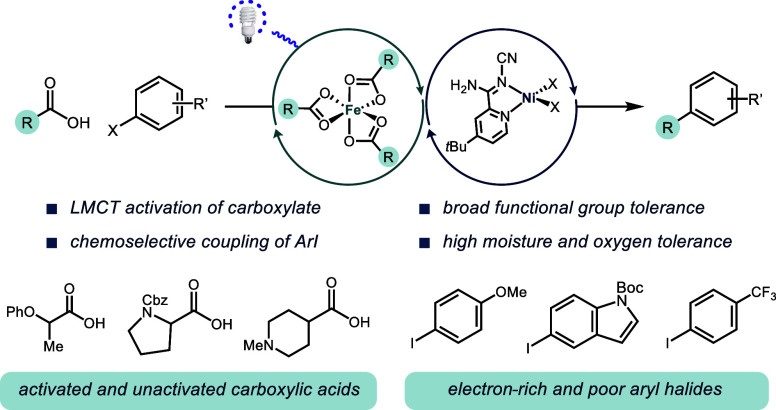

Decarboxylative cross-coupling of carboxylic acids and aryl halides has become a key transformation in organic synthesis to form C(sp^2^)–C(sp^3^) bonds. In this report, a base metal pairing between Fe and Ni has been developed with complementary reactivity to the well-established Ir and Ni metallaphotoredox reactions. Utilizing an inexpensive FeCl_3_ cocatalyst along with a pyridine carboxamidine Ni catalyst, a range of aryl iodides can be preferentially coupled to carboxylic acids over boronic acid esters, triflates, chlorides, and even bromides in high yields. Additionally, carboxylic acid derivatives containing heterocycles, *N*-protected amino acids, and protic functionality can be coupled in 23–96% yield with a range of sterically hindered, electron-rich, and electron-deficient aryl iodides. Preliminary catalytic and stoichiometric reactions support a mechanism in which Fe is responsible for the activation of carboxylic acid upon irradiation with light and a Ni^I^ alkyl intermediate is responsible for activation of the aryl iodide coupling partner followed by reductive elimination to generate product.

## Introduction

**Figure 1:**
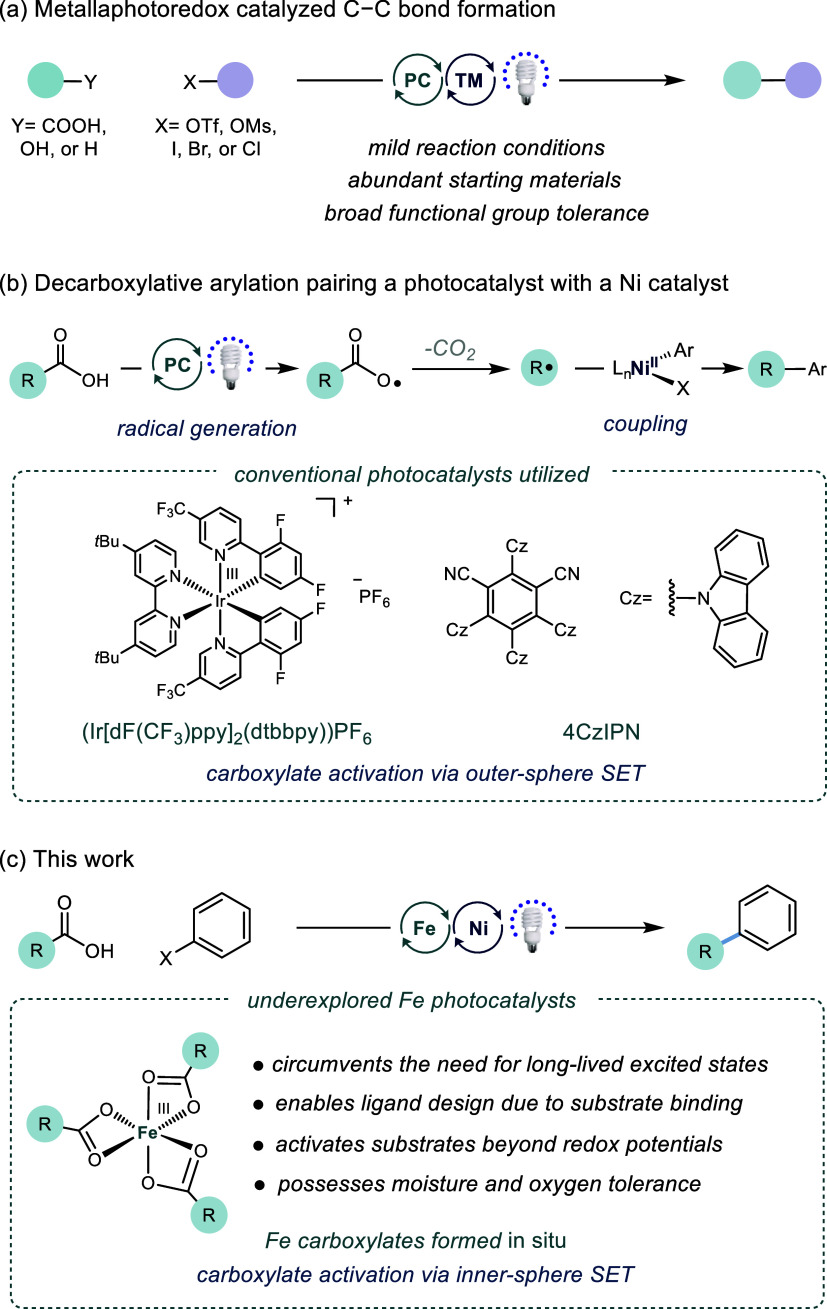
(a) Metallaphotoredox catalysis for C–C bond formation; (b) Metallaphotoredox strategies for decarboxylative arylation facilitated by Ni and outer-sphere photocatalysts (precious metal photocatalyst or an organic dye); (c) Fe and Ni decarboxylative arylation methodology.

The merger of photoredox catalysis with transition metal catalysis, metallaphotoredox catalysis, provides a valuable approach for the formation of C–C bonds.^1^ These transformations enable the cross-coupling of a diverse array of carbon electrophiles and carbon nucleophile equivalents by relying on a synergy between catalysts. Photoredox catalysts readily activate abundant, nonstandard coupling partners via single-electron, photoinduced charge transfer followed by decarboxylation (of alkanoic acids),^2^ β-scission (from alcohols),^3^ or hydrogen atom transfer mechanisms (from hydrocarbons).^4^ By comparison, transition metal catalysts are adept at net two-electron processes, such as coordination/activation of Lewis basic or π-rich functionality^5^ or oxidative addition of organohalides.^6,7^ The ability to combine these distinct mechanisms of substrate activation has proven fruitful in a variety of contexts including arylations, alkylations, and acylations (Figure 1a).^1,8^

A wide variety of transition metal bond-forming catalysts can be productive in these reactions, such as Pd, Au, Co, Cu, and Ni. However, the range of photocatalysts capable of enabling these transformations has been more limited.^9^ Typically, metallaphotoredox reactions have been reliant on precious metal catalysts, such as Ir and Ru, due to their stability, broad redox capabilities (for both oxidation and reduction steps), and well-understood reactivity.^10^ In 2014 Doyle and MacMillan first demonstrated the use of [4,4′-*Bis*(1,1-dimethylethyl)-2,2′-bipyridine-*N*1,*N*1′]*bis*[3,5-difluoro-2-[5-(trifluoromethyl)-2-pyridinyl-*N*]phenyl-C]Iridium(III) hexafluorophosphate ((Ir[dF(CF_3_)ppy]_2_(dtbbpy))PF_6_) and di-*tert*-butylbipyridine nickel ((dtbbpy)Ni) catalysts for the decarboxylative cross-coupling of alkyl carboxylic acids with aryl halides (Figure 1b).^11,12^ Since then, a growing number of researchers have extended this concept to a wide range of C(sp^2^)–C(sp^3^) bond-forming reactions,^1^ but the original decarboxylative arylation remains the most-used industrially.^13^ The high cost and limited availability of Ir have motivated a search for alternative photocatalysts, such as organic dyes made from more abundant elements. While promising results have been demonstrated, organic dyes often do not possess the same wide redox potential range as Ir photocatalysts and there are limitations in facilitating both oxidation of the substrate and the reduction of a cocatalyst.^14^ Alternatively, replacement of Ir and Ru with earth-abundant metals have suffered from short excited-state lifetimes that are insufficient to induce bimolecular electron transfer (outer-sphere electron transfer).^15^ An emerging strategy for the development of earth-abundant metal photocatalysts is one where substrate coordination to the metal is followed by a ligand-to-metal charge transfer (LMCT) event, which induces bond homolysis (inner-sphere electron transfer). This distinct mechanism provides an opportunity for catalyst-controlled substrate activation as there is a direct interaction between the substrate and metal. Metal complexes of Co, Cu, Ni, V, Ce, and Fe have been demonstrated to undergo photoinduced bond homolysis;^16^ however, only Ce has been paired with Ni for decarboxylative C–C bond formation via electrocatalysis,^17^ opening opportunities for chemical reaction discovery.

Fe salts have emerged as an attractive alternative to Ir photocatalysts because they are inexpensive, abundant, environmentally benign, and can offer complementary reactivity to precious metal-based photocatalysts.^18,19^ As an effective Lewis acid, Fe holds promise for modulating the chemoselectivity and regioselectivity of a transformation via substrate-guided ligand effects (Figure 1c).

Fe already has been demonstrated to overcome outer-sphere redox limitations, such as the activation of fluoroalkyl carboxylic acids.^20^ Additionally, Fe photocatalysts are tolerant of moisture and oxidizing atmospheric conditions, simplifying reaction setup on the benchtop.^21^ Recently, it has been demonstrated by our group and others that catalytic amounts of Fe enable photoinduced decarboxylative functionalization with broad applications.^22^ These decarboxylative transformations are hypothesized to occur through Fe^III^ (O(CO)R)*_n_* (R = alkyl) complexes that can undergo LMCT under visible light irradiation to generate a carboxylate radical and a Fe^II^ (O(CO)R)_*n*−1_ species.^19e^ However, while Fe has been used with Ni in several electrochemically driven reactions,^23^ it has not been applied broadly in metallaphotoredox reactions and is yet to be developed for decarboxylative arylations.^24,25^ We envisioned that the versatility of the Fe LMCT chemistry, when paired with the modularity of Ni, could enable a wide array of metallaphotoredox reactions.^26^

## Results and Discussion

**Figure 2:**
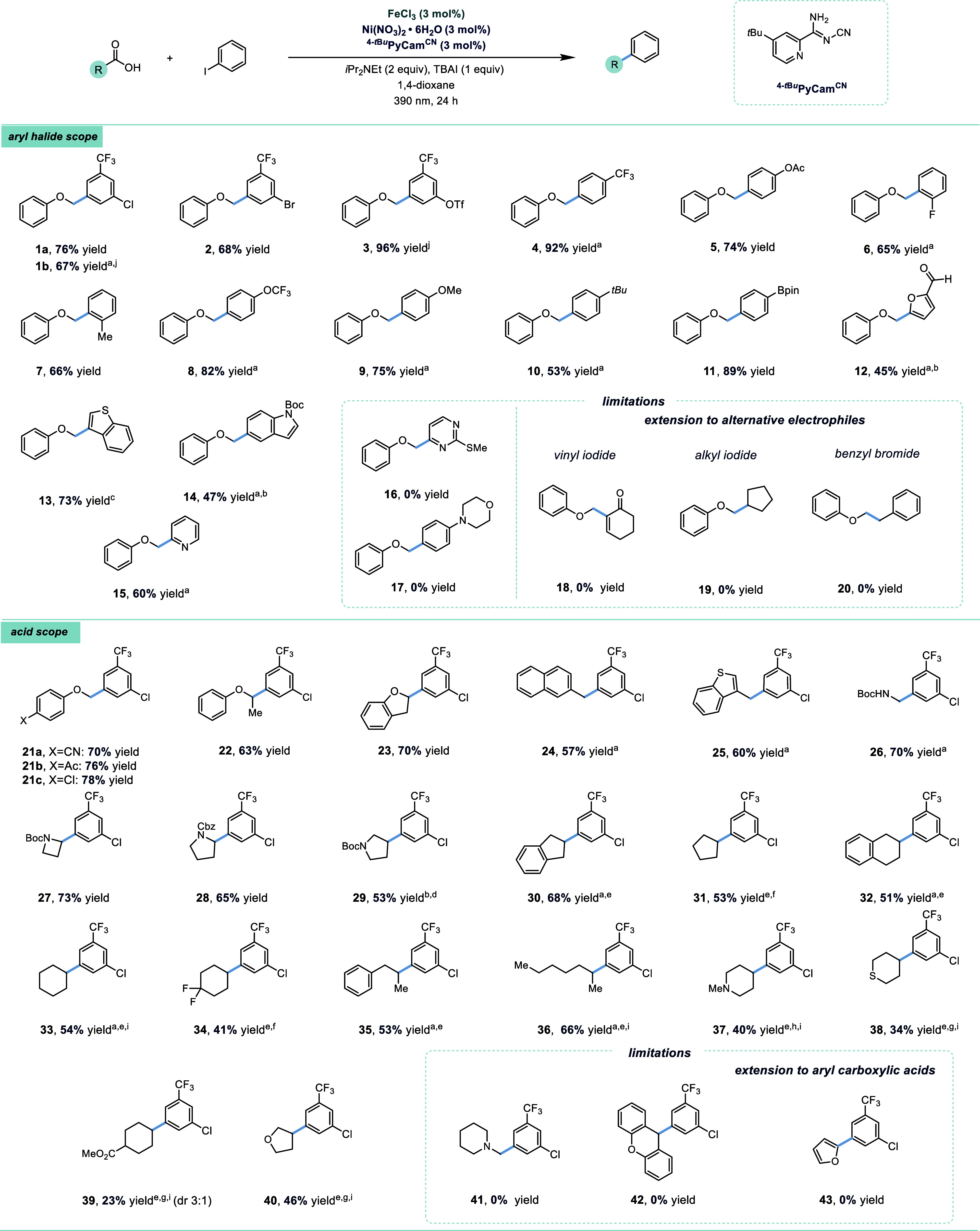
Organohalide and carboxylic acid scope for the Fe and Ni decarboxylative arylation reaction. Yields shown are isolated yields after purification. Reactions were performed on a 0.5 mmol scale with acid (1.3 equiv), aryl halide (1 equiv), *i*Pr_2_NEt (2 equiv), TBAI (1 equiv), FeCl_3_ (3 mol %), Ni(NO_3_)_2_·H_2_O and ^4-*t*Bu^PyCam^CN^ (3 mol %) in 10 mL 1,4-dioxane and sparged with N_2_. Reactions were irradiated at 390 nm for 24 h. ^a^0.5 equiv of phthalimide was added; ^b^MeCN was used as the solvent; ^c^15 mL of solvent was used; ^d^Na_2_CO_3_ was used as the base; ^e^Reactions run on 0.1 mmol scale with 1,4,8,11-tetraazacyclotetradecane (cyclam) as a ligand for FeCl_3_ (3 mol %); ^f^15 mol % of Zn was added; ^g^15 mol % of 4-ethylpyridine was added; ^h^30 mol % of ZnCl_2_ was added; ^i 1^H NMR yield vs an internal standard (1,2-dibromomethane or 1,3,5-trimethoxybenzene); ^j^Aryl bromide was used instead of aryl iodide.

To realize our proposed Fe and Ni metallaphotoredox methodology, 2-phenoxyacetic acid and 1-chloro-3-iodo-5-(trifluoromethyl)benzene were chosen as model substrates, as outlined in Table 1. These coupling partners provided access to medicinally relevant CF_3_-containing compounds and would facilitate the exmination of the chemoselectivity in the Fe and Ni reaction. Based on our previous Fe-catalyzed decarboxylation studies,^21^ we chose FeCl_3_ as the photocatalyst precursor and *N*,*N*-diisopropylethylamine (*i*Pr_2_NEt) as the base in acetonitrile under 390 nm irradiation for the first set of experiments. Optimization commenced by assessing a variety of established ligands for Ni cross-coupling reactions. We found that 4-(*tert*-butyl)-2-(*N*-cyanocarboxamidine)pyridine(^4-*t*Bu^PyCam^CN^), a ligand developed in collaboration with Pfizer,^27^ surfaced as the best ligand, delivering the desired product in 60% yield (Table S1). Commonly used ligands for Ni metallaphotoredox reactions such as 4,4′-di-*tert*-butylbipyridine (^4-*t*Bu^Bpy) or ligands that were previously found to promote decarboxylation on Fe (2-picolinic acid and 2,2′-dipicolylamine) gave the product in only 37, 11, and 3% yields, respectively (Table S1). When the reaction was further investigated C−H arylation of the solvent, dimerization of the alkanoic acid, and hydrodehalogenation were observed. To suppress side reactivity, several additives were explored, including tetrabutylammonium salts and cyclic imides (Table S7).^28,29^ It was found that TBAI was superior to other salt additives (Table 1, entries 9 and 10) and the addition of 0.5 equiv of phthalimide improved yields slightly (Table 1, entry 7). It was later shown that the impact of phthalimide was more pronounced for other substrates and was essential for accessing a general aryl iodide scope (Figure 2). Upon examining the influence of the wavelength of light, a 67% yield could be attained with blue (427 nm) light, but improved reactivity was observed at 390 nm (Table 1, entries 1 and 11). The reaction was also amenable to a range of polar aprotic solvents and proceeded effectively in 1,4-dioxane, dimethyl carbonate, and acetonitrile (Table 1, entry 12 and Table S17). While equimolar amounts of the acid and the aryl halide resulted in high yields (Table S18) it was noted that a slight excess of acid (1.3 equiv) was beneficial. Control experiments for the Fe and Ni system demonstrated the need for irradiation, base, and both catalysts for practical yields to be achieved (Table 1, entries 2–5 and Table S18).

**Table 1:**
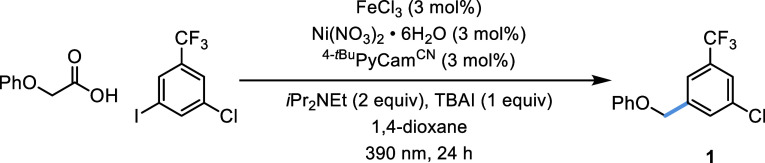
Selected Optimization and Control Data for the Fe and Ni Decarboxylative Coupling^a^

entry	deviation from above	yield 1 (%)
1	none	86
2	no Fe catalyst	19
3	no Ni catalyst	0
4	no ligand	0
5	no light (60 °C)	0
6	no TBAI	73
7	with 0.5 equiv of phthalimide	90
8	Cs_2_CO_3_ instead of *i*Pr_2_NEt	9
9	KI instead of TBAI	72
10	TBACl instead of TBAI	31
11	427 nm instead of 390 nm	62
12	MeCN instead of 1,4-dioxane	67

a Reactions were performed on a 0.5 mmol scale. Acid (1.3 equiv), aryl halide (1 equiv), *i*Pr_2_NEt (2 equiv), TBAI (1 equiv), FeCl_3_ (3 mol %), Ni(NO_3_)_2_·6H_2_O (3 mol %) and ^4-*t*Bu^PyCam^CN^ (3 mol %) were added to 10 mL of 1,4-dioxane, and the mixture was sparged with N_2_ for 5 min. The mixture was irradiated at 390 nm for 24 h. Yields were determined by ^1^H NMR vs an internal standard (1,2-dibromomethane).

Having identified the optimal reaction conditions, we next assessed the generality of the decarboxylative arylation reaction with respect to the aryl halide coupling partner. Unlike many metallaphotoredox reactions, these reactions exhibited high selectivity for reaction at the C–I bond over C–Cl, C–Br, or C–OTf (**1**–**3**). It was unclear if this high selectivity was due to the ligand or the oxidation state of Ni in oxidative addition, which both influence selectivity.^30^ In the absence of a C–I bond, oxidative addition at C–Br also afforded the alkylated product (**1b** and **3**), but C–Cl and C–OTf bonds were unreactive under these conditions. The optimized conditions tolerated both electron-deficient and electron-rich aryl iodides with a variety of substituents at *ortho, meta*, and *para* positions. Aryl iodides bearing trifluoromethyl (**4**, 92% yield), acetate (**5**, 60% yield), fluorine (**6**, 65% yield), and trifluoromethoxy (**8**, 82% yield) substituents all provided useful yields. Electron-rich aryl iodides (**9** and **10**), which can be challenging to use in metallaphotoredox reactions, were tolerated.^1b,31^ Reactive functional groups, such as a boronic acid pinacol ester (**11**) and an aldehyde (**12**), were also tolerated under these conditions. Moreover, this method could be utilized to functionalize several heteroaryl iodides containing furan (**12**), benzothiophene (**13**), *N*-protected indole (**14**), and pyridine (**15**). It is notable that unsubstituted pyridine does not yield Minisci chemistry side products.^22b^ Applying the standard protocol to the coupling of alternative electrophiles such as vinyl iodide (**18**), alkyl iodide (**19**), or benzyl bromide (**20**) was unsuccessful without further ligand optimization.

After examining the aryl iodide scope, we examined a range of carboxylic acids under the optimized conditions (Figure 2). Prior methods using Ir as a photocatalyst have required a substantial excess (3 equiv or more) of carboxylic acid and exclusion of oxygen and water (attributed in part to the slow bimolecular SET step).^32^ We anticipated that the use of an Fe photocatalyst might be advantageous in this context due to its facile decarboxylation via a unimolecular process.^19^ Initially, activated carboxylic acids were examined. Carboxylic acids with an α–heteroatom reacted under these conditions to give the expected product in excellent yields (**21**–**23** and **26**–**29**, 65–78% yield). *N*-protected α-amino acids (glycine (**26**), and proline (**28**)) and a β-amino acid (**29**) were also coupled in good yields. Benzylic acids with extended π-systems can be employed effectively (**24**) however several substrates generated unproductive alkyl dimer leading to reduced yields of cross-coupled product (**42**). While the standard protocol could not be extended generally to unactivated carboxylic acids, the substitution of an inorganic base for *i*Pr_2_NEt or the addition of a ligand on Fe (cyclam, to facilitate decarboxylation) enabled successful coupling. Decarboxylative arylation of cyclopentane carboxylic acid (**31**), cyclohexane carboxylic acid (**33**), and their derivatives (**30**, **32**, and **34**) were achieved in good yields (41–68% yield). Additionally, acyclic carboxylic acids (**35** and **36**) were amenable to decarboxylative coupling (53 and 66% yield, respectively). For several substrates, the addition of Zn (**31** and **34**) or 4-ethylpyridine (**38**–**40**) was necessary for productive coupling. Finally, while electron-rich trialkyl amines are susceptible to oxidation by photocatalysts proceeding via an outer-sphere mechanism, 1-methylpiperidine-4-carboxylic acid (**37**) was tolerated and underwent successful arylation in 40% yield. This result is promising and demonstrates the potential of Fe to overcome some of the challenges faced when using traditional photoredox systems (Table S23). Although the reaction was applicable to a variety of sp^3^-carboxylic acids, the activation of sp^2^-carboxylic acids remains a challenge (**43**).

**Figure 3:**
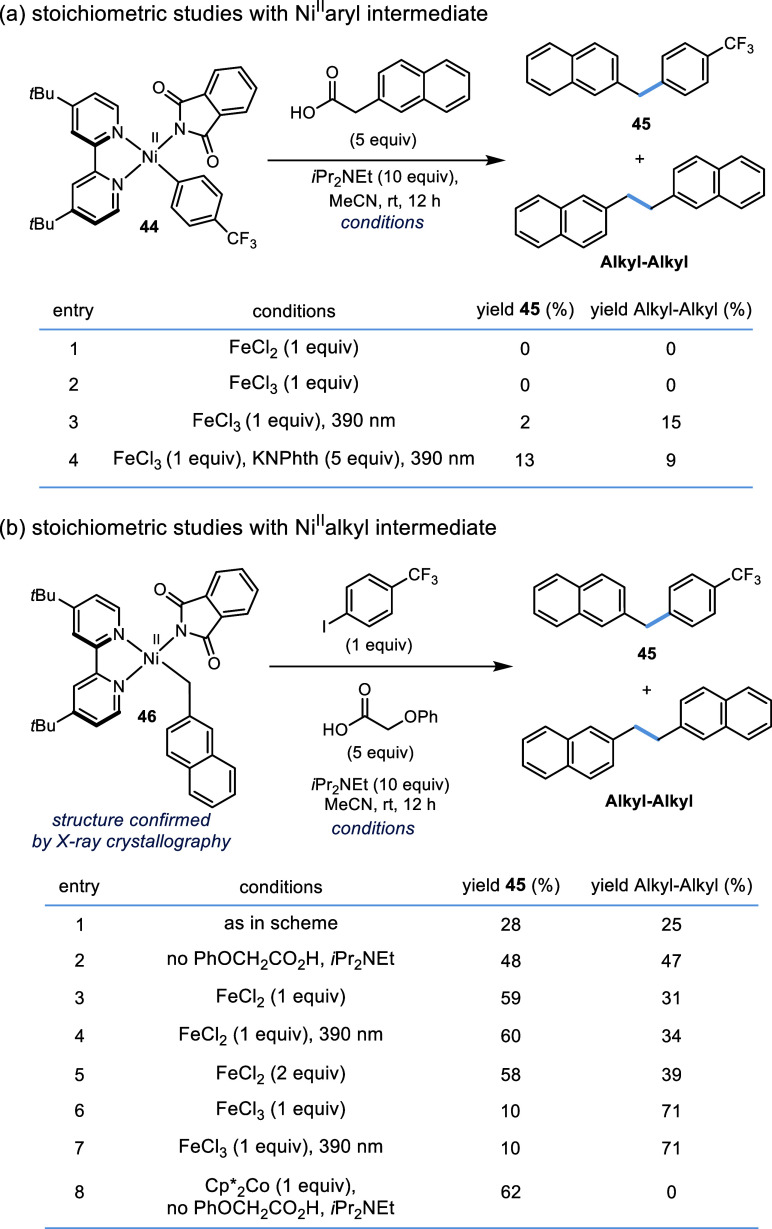
Stoichiometric reactions of Ni^II^ complexes. (a) Reactivity of Ni^II^(Aryl)(NPhth) intermediate. The yield of Alkyl–Alkyl is based on 5 equiv of carboxylic acid; (b) Reactivity of Ni^II^(Alkyl)(NPhth) intermediate. The yield of Alkyl–Alkyl is based on **46**.

To shed light on the different reactivity between this new Fe system and more established Ir systems, we elected to study the reactivity of two potential organonickel intermediates. Many decarboxylative metallaphotoredox systems proceed through the oxidative addition of a low valent Ni^0^ species into an aryl halide, yielding a Ni^II^(Aryl)X intermediate which subsequently captures a radical derived from a cocatalyst.^1b,28b^ However, a conceivable alternative mechanistic pathway toward the same high valent Ni^III^ species could occur through radical capture from Ni^I^X to Ni^II^(Alkyl)X. This Ni^II^ species, upon reduction to Ni^I^, could react with ArI by oxidative addition. To begin our study, we synthesized (^4-*t*Bu^Bpy)Ni^II^(Aryl)(NPhth) (**44**) and (^4-*t*Bu^Bpy)Ni^II^(Alkyl)(NPhth) (**46**)^28b,33^ and studied their stoichiometric reactivity under catalytically relevant conditions (Figure 3 and Tables S29–S34). Yamamoto et al. and MacMillan et al. noted that when X = phthalimido, Ni^II^ alkyl and Ni^II^ aryl species are remarkably stable to protonation, disproportionation, and thermal decomposition.^28^ Although reactions using ^4-*t*Bu^Bpy as a ligand provided lower yields of product when compared to ^4-*t*Bu^PyCam^CN^ (25–50% yield, Table S28), ^4-*t*Bu^Bpy offers increased synthetic tractability and avoids ambiguity around the NH protonation state while maintaining a similar reaction profile to ^4-*t*Bu^PyCam^CN^. Therefore, we elected to use ^4-*t*Bu^Bpy for the following mechanistic experiments.

To begin, subjecting aryl Ni precursor **44** to either FeCl_2_ or FeCl_3_ without irradiation yielded no cross-coupled product **45** and only Ar–H (Figure 3a, entries 1 and 2 and Tables S33 and S34).Upon irradiation, in the presence of FeCl_3_, only 2% of **45** was obtained (Figure 3a, entry 3). MacMillan also noted that **44** was quickly protonated by a carboxylic acid/base mixture, but they obtained a 67% yield of coupled product when excess KNPhth was added. When we added 5 equiv of KNPhth along with FeCl_3_, acid, and base, 13% of the product was observed. However, a comparable amount of Ar–NPhth (6%) was obtained, a product not observed in catalytic reactions (Table S34). Taken together, this suggests that the reaction does not predominantly proceed through a Ni^II^ aryl intermediate.

In contrast to the instability and poor selectivity observed with **44**, reactions with **46** (fully characterized, including by X-ray, CCDC 2371033) formed product under a variety of conditions (Figure 3b).^34^ There are several key observations that are made from these experiments. First, light was not required for product formation, suggesting that light-driven electron-transfer pathways are not essential for this step in the catalytic cycle. (Figure 3b, entries 3 and 4). Second, Ni reduction seems to be required for product formation (Figure 3b, entries 1–5, 8 vs entries 6 and 7). While the highest yields were obtained with added reductants (Figure 3b, entries 3 and 8), a reasonable yield was obtained without any added reductant (Figure 3b, entry 1). The observation of bialkyl side products (Figure 3b, entries 1 and 2) suggests the formation of Ni^0^ from reductive elimination of (L)Ni^II^(Alkyl)_2_ species. Among other pathways involving low-valent Ni species, the generated Ni^0^ could react with **46** to form (^4-*t*Bu^Bpy)Ni^I^(Alkyl). Ni(I) species can undergo oxidative addition with iodoarenes with relatively low barriers^30b,35^ and reductive elimination from (^4-*t*Bu^Bpy)Ni^III^(Aryl)(Alkyl)X is expected to be fast. Third, in general, product formation from **46** can outcompete protonation pathways which were problematic for reactions with **44** (Figure 3a,b). This can be understood by considering relative concentration of reagents: **44** needs an alkyl radical (low concentration) but **46** needs Ar–I (high concentration) to form product.

**Figure 4:**
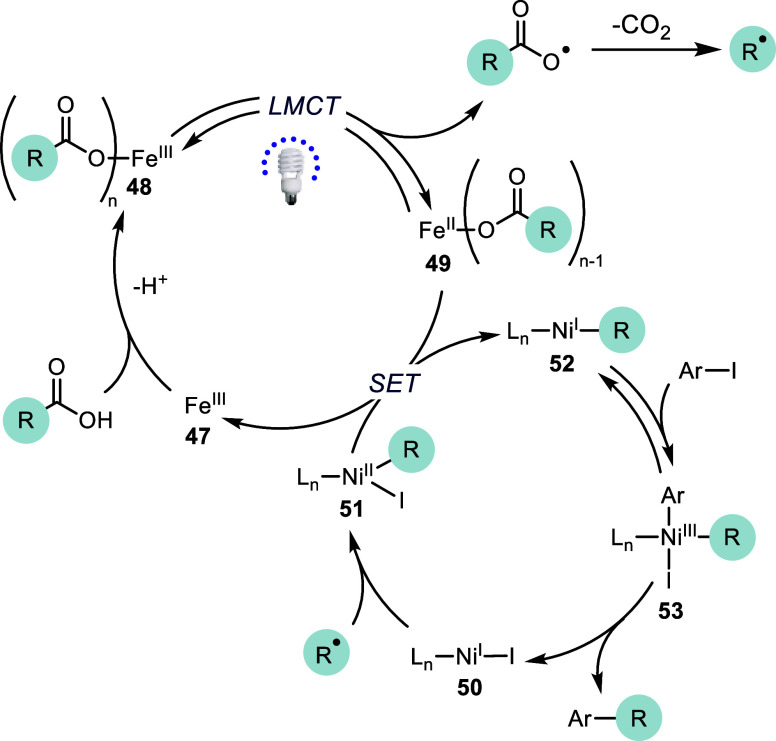
Proposed mechanism of Fe and Ni mediated decarboxylative arylation. R = alkyl and L = ^4-*t*Bu^PyCam^CN^.

These results, in addition to the known ability of Fe^III^ carboxylate species to absorb light and induce decarboxylation to form alkyl radicals and Fe^II^ species,^19−22^ allows us to propose the following decarboxylative catalytic cycle (Figure 4). First, Fe carboxylate complex **48** can be generated through the reaction of an Fe salt with base and carboxylic acid.^21^ Under irradiation with 390 nm wavelength light, this complex then undergoes an LMCT event to generate a carboxylate radical and Fe^II^ species **49**. Rapid radical decarboxylation and CO_2_ extrusion leads to generation of an alkyl radical. Addition of this radical into (L*_n_*)Ni^I^X **50** forms (L*_n_*)Ni^II^(Alkyl)X intermediate **51**. This species then undergoes single electron reduction by **49** or (L*_n_*)Ni^I^X to form (L*_n_*)Ni^I^(Alkyl) **52**. Reversible oxidative addition of Ar–I forms (L*_n_*)Ni^III^(Alkyl)(Aryl)(I) **53** and reductive elimination forms cross-coupled product regenerating (L*_n_*)Ni^I^X **50**. While this proposal is consistent with our data, additional experiments are needed to understand key aspects of the proposed cycle, such as the exact Fe species involved with and without ligand, the radical capture step by Ni, and the role of several additives that were used to expand the scope (iodide, zinc salts, and 4-ethylpyridine). Of particular interest would be an understanding of the effects of Ir and Fe mediated radical generation on the Ni catalytic cycle. These experiments are underway and will be reported in due course.

## Conclusions

In conclusion, we report an Fe and Ni metallaphotoredox enabled decarboxylative cross-coupling. This procedure not only utilizes low catalyst loading (3 mol %) of inexpensive Fe and Ni catalysts on the benchtop but tolerates a range of functionality including aldehydes, esters, and heterocycles. Additionally, a range of activated and unactivated carboxylic acids operate as feasible coupling partners. In the absence of an aryl iodide, electron-deficient aryl bromides could be used as viable substrates. Through mechanistic investigations, it was demonstrated that this Fe/Ni decarboxylative arylation is mechanistically distinct from traditional Ir/Ni decarboxylation systems, proceeding through a Ni^I^/Ni^III^ oxidative addition pathway. Future work will be directed at understanding this new Fe and Ni catalyst system and toward developing other base metal metallaphotoredox transformations.

## Supporting Information Available

Materials and experimental procedures; optimization and control reactions; time course data; UV–vis spectra; CV data; XYZ coordinates for X-ray structure of **46**; stoichiometric experiments; ^1^H NMR, ^13^C NNMR, ^19^F NMR, and ^11^B NMR spectra and HRMS data for compounds (PDF)

## References

[ref1] aSkubi K. L.; Blum T. R.; Yoon T. P. Dual Catalysis Strategies in Photochemical Synthesis. Chem. Rev. 2016, 116, 10035–10074. 10.1021/acs.chemrev.6b00018. 27109441 PMC5083252

[ref2] Xuan J.; Zhang Z.-G.; Xiao W.-J. Visible-Light Induced Decarboxylative Functionalization of Carboxylic Acids and their Derivatives. Angew. Chem., Int. Ed. 2015, 54, 15632–15641. 10.1002/anie.201505731. 26509837

[ref3] aYayla H. G.; Wang H.; Tarantino K. T.; Orbe H. S.; Knowles R. R. Catalytic Ring-Opening of Cyclic Alcohols Enabled by PCET of Strong O–H Bonds. J. Am. Chem. Soc. 2016, 138, 10794–10797. 10.1021/jacs.6b06517. 27515494 PMC5110324

[ref4] aNagib D. A.; Scott M. E.; MacMillan D. W. C. Enantioselective α-Trifluoromethylation of Aldehydes via Photoredox Organocatalysis. J. Am. Chem. Soc. 2009, 131, 10875–10877. 10.1021/ja9053338. 19722670 PMC3310169

[ref5] Williams L. J.; Bhonoah Y.; Wilkinson L. A.; Walton J. W. As Nice as π: Aromatic Reactions Activated by π-Coordination to Transition Metals. Chem. - Eur. J. 2021, 27, 3650–3660. 10.1002/chem.202004621. 33210827 PMC7986375

[ref6] aHalpern J. Oxidative-Addition Reactions of Transition Metal Complexes. Acc. Chem. Res. 1970, 3, 386–392. 10.1021/ar50035a004.

[ref7] Marzo L.; Pagire S. K.; Reiser O.; König B. Visible-Light Photocatalysis: Does It Make a Difference in Organic Synthesis?. Angew. Chem., Int. Ed. 2018, 57, 10034–10072. 10.1002/anie.201709766. 29457971

[ref8] aJoe C. L.; Doyle A. G. Direct Acylation of C(sp^3^)–H Bonds Enabled by Nickel and Photoredox Catalysis. Angew. Chem., Int. Ed. 2016, 55, 4040–4043. 10.1002/anie.201511438. PMC4807873 26890705

[ref9] aKalyani D.; McMurtrey K. B.; Neufeldt S. R.; Sanford M. S. Room-Temperature C–H Arylation: Merger of Pd-Catalyzed C–H Functionalization and Visible-Light Photocatalysis. J. Am. Chem. Soc. 2011, 133, 18566–18569. 10.1021/ja208068w. 22047138 PMC3222896

[ref10] aPrier C. K.; Rankic D. A.; MacMillan D. W. C. Visible Light Photoredox Catalysis with Transition Metal Complexes: Applications in Organic Synthesis. Chem. Rev. 2013, 113, 5322–5363. 10.1021/cr300503r. 23509883 PMC4028850

[ref11] Zuo Z.; Ahneman D. T.; Chu L.; Terrett J. A.; Doyle A. G.; MacMillan D. W. C. Merging photoredox with nickel catalysis: Coupling of α-carboxyl sp^3^–carbons with aryl halides. Science 2014, 345, 437–440. 10.1126/science.1255525. 24903563 PMC4296524

[ref12] The Molander group also helped in establishing the reactivity of Ir/Ni systems using BF_3_K salts as the electrophiles instead of carboxylic acids. (refer to reference (9e)).

[ref13] aHsieh H.-W.; Coley C. W.; Baumgartner L. M.; Jensen K. F.; Robinson R. I. Photoredox Iridium–Nickel Dual-Catalyzed Decarboxylative Arylation Cross-Coupling: From Batch to Continuous Flow via Self-Optimizing Segmented Flow Reactor. Org. Process Res. Dev. 2018, 22, 542–550. 10.1021/acs.oprd.8b00018.

[ref14] For the photophysical and electrochemical properties of common organic photoredox catalysts see: Romero N. A.; Nicewicz D. A. Organic Photoredox Catalysis. Chem. Rev. 2016, 116, 10075–10166. 10.1021/acs.chemrev.6b00057. 27285582

[ref15] aWenger O. S. Photoredox Complexes with Earth-Abundant Metals. J. Am. Chem. Soc. 2018, 140, 13522–13533. 10.1021/jacs.8b08822. 30351136

[ref16] aAbderrazak Y.; Bhattacharyya A.; Reiser O. Visible-Light-Induced Homolysis of Earth-Abundant Metal-Substrate Complexes: A Complementary Activation Strategy in Photoredox Catalysis. Angew. Chem., Int. Ed. 2021, 60, 21100–21115. 10.1002/anie.202100270. PMC8519011 33599363

[ref17] aChen Y.; Wang X.; He X.; An Q.; Zuo Z. Photocatalytic Dehydroxymethylative Arylation by Synergistic Cerium and Nickel Catalysis. J. Am. Chem. Soc. 2021, 143, 4896–4902. 10.1021/jacs.1c00618. 33756079

[ref18] Based on prices listed on Sigma Aldrich, FeCl_3_ costs $0.052/g ($8.43/mol) while IrCl_3_ costs $346/g ($103,308.68/mol) and Ir[dF(CF_3_)ppy]_2_(dtbbpy)PF_6_ costs $998/g ($1,119,666.18/mol). Based on prices listed on Oakwood Chemical, FeCl_3_ costs $0.094/g ($15.24/mol) while IrCl_3_ costs $390/g ($116,446.2/mol) and Ir[dF(CF_3_)ppy]_2_(dtbbpy)PF_6_ costs $674/g ($756,167.34/mol).

[ref19] aFrey P. A.; Reed G. H. The Ubiquity of Iron. ACS Chem. Biol. 2012, 7, 1477–1481. 10.1021/cb300323q. 22845493

[ref20] Bian K.-J.; Lu Y.-C.; Nemoto D. Jr; Kao S.-C.; Chen X.; West J. G. Photocatalytic hydrofluoroalkylation of alkenes with carboxylic acids. Nat. Chem. 2023, 15, 1683–1692. 10.1038/s41557-023-01365-0. 37957278 PMC10983801

[ref21] Crocker M. S.; Lin J.-Y.; Nsouli R.; McLaughlin N. D.; Musaev D. G.; Mehranfar A.; Lopez E. R.; Ackerman-Biegasiewicz L. K. G. Transformative Ligand Effects in Fe Photocatalyzed Giese-type Additions Chem. Catal. 10.1016/j.checat.2024.101131.

[ref22] aFeng G.; Wang X.; Jin J. Decarboxylative C–C and C–N Bond Formation by Ligand-Accelerated Iron Photocatalysis. Eur. J. Org. Chem. 2019, 2019, 6728–6732. 10.1002/ejoc.201901381.

[ref23] aXue P.; Li L.; Fu N. Pairing Iron and Nickel Catalysis for Electrochemical Esterification of Aryl Halides with Carbazates. Org. Lett. 2022, 24, 7595–7599. 10.1021/acs.orglett.2c03034. 36201293

[ref24] For a concurrent, complementary study demonstrating the use of Fe/Ni metallaphotoredox catalysis for the coupling of alcohols with aryl halides, see: Jaber M.; Ozbay Y.; Chefdeville E.; Tran G.; Amgoune A. Unified Photocatalytic Strategy for the Cross-Coupling of Alcohols with Aryl Halides Enabled by Synergistic Nickel and Iron LMCT Catalysis. ACS Catal. 2024, 14, 12757–12768. 10.1021/acscatal.4c03799.

[ref25] Xiong N.; Li Y.; Zeng R. Merging Photoinduced Iron-Catalyzed Decarboxylation with Copper Catalysis for C–N and C–C Coupling. ACS Catal. 2023, 13, 1678–1685. 10.1021/acscatal.2c05293.

[ref26] Dicciainni J. B.; Diao T. Mechanism of Nickel-Catalyzed Cross-Coupling Reactions. Trends Chem. 2019, 1, 830–844. 10.1016/j.trechm.2019.08.004.

[ref27] Hansen E. C.; Pedro D. J.; Wotal A. C.; Gower N. J.; Nelson J. D.; Caron S.; Weix D. J. New ligands for nickel catalysis from diverse pharmaceutical heterocycle libraries. Nat. Chem. 2016, 8, 1126–1130. 10.1038/nchem.2587. 27874864 PMC5123601

[ref28] aYamamoto T.; Kohara T.; Yamamoto A. Preparation and Properties of Monoalkylnickel(II) Complexes NiR(NR^1^R^2^)L_2_ Having Imido, Imidazolato, or Methyl Phenylcarbamato–*N* Ligand. Bull. Chem. Soc. Jpn. 1981, 54, 1720–1726. 10.1246/bcsj.54.1720.

[ref29] Piber M.; Jensen A. E.; Rottländer M.; Knochel P. New Efficient Nickel- and Palladium-Catalyzed Cross-Coupling Reactions Mediated by Tetrabutylammonium Iodide. Org. Lett. 1999, 1, 1323–2326. 10.1021/ol9907872.

[ref30] aBajo S.; Laidlaw G.; Kennedy A. R.; Sproule S.; Nelson D. J. Oxidative Addition of Aryl Electrophiles to a Prototypical Nickel(0) Complex: Mechanism and Structure/Reactivity Relationships. Organometallics 2017, 36, 1662–1672. 10.1021/acs.organomet.7b00208.

[ref31] Zuo Z.; Cong H.; Li W.; Choi J.; Fu G. C.; MacMillan D. W. C. Enantioselective Decarboxylative Arylation of α-Amino Acids via the Merger of Photoredox and Nickel Catalysis. J. Am. Chem. Soc. 2016, 138, 1832–1835. 10.1021/jacs.5b13211. 26849354 PMC4862596

[ref32] Beil S. B.; Chen T. Q.; Intermaggio N. E.; MacMillan D. W. C. Carboxylic Acids as Adaptive Functional Groups in Metallaphotoredox Catalysis. Acc. Chem. Res. 2022, 55, 3481–3494. 10.1021/acs.accounts.2c00607. 36472093 PMC10680106

[ref33] Zhang Y.; Tanabe Y.; Kuriyama S.; Nishibayashi Y. Photoredox- and Nickel-Catalyzed Hydroalkylation of Alkynes with 4-Alkyl-1,4-dihydropyridines: Ligand-Controlled Regioselectivity. Chem. - Eur. J. 2022, 28, e202200727 10.1002/chem.202200727. 35475521

[ref34] Yields met or exceeded the maximum yield observed in catalytic reactions with ^*t*Bu^Bpy (50%). We also noted that iodide additives, while important for catalytic reactions, had no effect on stoichiometric reactions. This suggests that iodide’s role is outside of the reduction/oxidative/addition/product-forming steps.

[ref35] Tang T.; Hazra A.; Min D. S.; Williams W. L.; Jones E.; Doyle A. G.; Sigman M. S. Interrogating the Mechanism Features of Ni(I)-Mediated Aryl Iodide Oxidative Addition Using Electroanalytical and Statistical Modeling Techniques. J. Am. Chem. Soc. 2023, 145, 8689–8699. 10.1021/jacs.3c01726. PMC10548350 37014945

