# Supplementary material for: Decarboxylative Cross-Coupling Enabled by Fe and Ni Metallaphotoredox Catalysis
Source: J Am Chem Soc. 2024 Oct 18;146(43):29551–9. doi: 10.1021/jacs.4c09621 (PMC11528444; doi:10.1021/jacs.4c09621)

Decarboxylative Cross-Coupling Enabled by Fe and Ni Metallaphotoredox Catalysis

Reem Nsouli[a], Sneha Nayak[‡a], Venkadesh Balakrishnan[‡a], Jung-Ying Lin[‡a], Benjamin K. Chi[b], Hannah G. Ford[a], Andrew V. Tran[a], Ilia A. Guzei[b], John Bacsa[a], Nicholas R. Armada[c], Fedor Zenov[c], Daniel J. Weix[*b], Laura K. G. Ackerman-Biegasiewicz[*a]

[a]Department of Chemistry, Emory University, Atlanta, GA 30322, United States
[b]Department of Chemistry, University of Wisconsin-Madison, Madison, WI 53716, United States
[c]School of Molecular Science, Arizona State University, Tempe, AZ 85281, United States

Supporting Information

CONTENTS

I. GENERAL INFORMATION 10

II. REACTION SETUP 12

III. EXPERIMENTAL PROCEDURES 13

A. GENERAL PROCEDURE FOR OPTIMIZATION IN THE DECARBOXYLATIVE ARYLATION 13

1. EXAMINATION OF LIGANDS FOR THE REACTION OF 2-PHENOXYACETIC ACID (0.1 MMOL SCALE) WITH 1-BROMO-3-IODO-5-(TRIFLUOROMETHYL)BENZENE IN MECN 13

2. OPTIMIZATION OF THE REACTION OF 2-PHENOXYACETIC ACID (0.1 MMOL SCALE) WITH 1-BROMO-3-IODO-5-(TRIFLUOROMETHYL)BENZENE IN MECN 15

3. TRANSLATION OF OPTIMIZED CONDITIONS TO THE REACTION OF 2-PHENOXYACETIC ACID (0.1 MMOL SCALE) WITH 1-CHLORO-3-IODO-5-(TRIFLUOROMETHYL)BENZENE IN 1,4-DIOXANE 16

B. GENERAL PROCEDURE FOR ARYL IODIDE SCOPE (0.5 MMOL SCALE) IN THE DECARBOXYLATIVE ARYLATION 17

C. GENERAL PROCEDURE FOR CARBOXYLIC ACID SCOPE (0.5 MMOL SCALE) IN THE DECARBOXYLATIVE ARYLATION 18

D. GENERAL PROCEDURE FOR UNACTIVATED CARBOXYLIC ACID SCOPE (0.1 MMOL SCALE) IN THE DECARBOXYLATIVE ARYLATION 19

E. PROCEDURE FOR TIME COURSE STUDIES 20

1. 2-PHENOXYACETIC ACID COUPLED WITH 1-CHLORO-3-IODO-5-(TRIFLUOROMETHYL)BENZENE 20

2. 2,3-DIHYDRO-1H-INDENE-2-CARBOXYLIC ACID COUPLED WITH 1-CHLORO-3-IODO-5-(TRIFLUOROMETHYL)BENZENE 21

F. PROCEDURE FOR THE SYNTHESIS OF 1,3-DIOXOISOINDOLIN-2-YL 2,3-DIHYDRO-1H-INDENE-2-CARBOXYLATE 22

G. PROCEDURE FOR THE SYNTHESIS OF Ni COMPLEXES 22

1. PROCEDURE FOR SYNTHESIS OF ($^{4-TBU}$BPY)Ni(4-TRIFLUOROMETHYLPHENYL)(PHTHALIMIDO) (44) 22

2. PROCEDURE FOR SYNTHESIS OF ($^{4-TBU}$BPY)Ni(2-NAPHTHALENYLMETHYL)(PHTHALIMIDO) (46) 23

IV. OPTIMIZATION 24

A. LIGANDS 24

1. LIGAND OPTIMIZATION ON Ni 25

2. LIGAND OPTIMIZATION ON Fe 26

3. LIGAND OPTIMIZATION ON Fe WITH $^{4-TBU}$PYCAM^{CN} ON Ni 27

B. SOLVENT 28

C. BASE 29

D. BASE EQUIVALENTS 30

E. ADDITIVES 31

F. ADDITIVE RATIOS 32

G. CATALYST LOADING....33
H. CATALYST RATIOS....34
I. WAVELENGTH....35
1. USING 1-BROMO-3-IODO-5-(TRIFLUOROMETHYL)BENZENE....35
2. USING 1-CHLORO-3-IODO-5-(TRIFLUOROMETHYL)BENZENE....36
J. SOLVENT WITH ADDITIVES....41
K. CONTROLS....42
V. REACTION ROBUSTNESS....44
A. WATER TOLERANCE....44
B. AIR TOLERANCE....45
C. COMPARISON WITH IR AND NI METALLAPHOTOREDOX CATALYSIS....46
VI. UV-vis SPECTROSCOPY STUDIES....48
A. UV-VIS SPECTRA OF LIGANDS AND ADDITIVES....48
1. $^{4-tBu}$PYCAM^{CN}....48
2. 1,4,8,11-TETRAAZACYCLOTETRADECANE (CYCLAM)....49
3. TBAI....50
4. PHTHALIMIDE....51
B. UV-VIS SPECTRA OF NI COMPLEX....52
1. $Ni(NO_3)_2$•$6H_2O$....52
2. $Ni(NO_3)_2$•$6H_2O$, $^{4-tBu}$PYCAM^{CN}....53
3. COMBINED SPECTRA FOR $Ni(NO_3)_2$•$6H_2O$....54
C. UV-VIS OF FE COMPLEX....60
1. $FeCl_3$....60
2. $FeCl_3$, CYCLAM....62
3. COMBINED SPECTRA FOR $FeCl_3$....63
VII. MATCHED AND MIS-MATCHED METAL-LIGAND PAIRINGS UNDER STANDARD REACTION CONDITIONS....71
A. ACTIVATED ACID: 2-PHENOXYACETIC ACID....71
B. UNACTIVATED ACID: 2,3-DIHYDRO-1H-INDENE-2-CARBOXYLIC ACID....72
VIII. MECHANISTIC EXPERIMENTS....73
A. TIME COURSE STUDIES....73
1. STANDARD REACTION USING 2-PHENOXYACETIC ACID....73
2. STANDARD REACTION USING 2,3-DIHYDRO-1H-INDENE-2-CARBOXYLIC ACID....74
B. CATALYTIC REACTIONS....75
1. TESTING $Fe_3(O)(OAC)_6CLO_4$ AS AN Fe CATALYST....75

2. TESTING Fe(OAC)$_2$ AS AN Fe CATALYST 76
3. REACTION WITH REDOX ACTIVE ESTER 76
4. DATA TO VALIDATE STOICHIOMETRIC EXPERIMENTS 77
C. STOICHIOMETRIC REACTIONS WITH Ni COMPLEXES 44 AND 46 78
1. REACTIVITY OF ($^{4-TBU}$BPY)Ni(2-NAPHTHENYLMETHYL)(PHTHALIMIDO) WITH ARYL IODIDE IN THE PRESENCE OF FeCl$_2$ 78
2. REACTIVITY OF ($^{4-TBU}$BPY)Ni(2-NAPHTHENYLMETHYL)(PHTHALIMIDO) WITH INCREASING CONCENTRATION OF ARYL IODIDE IN THE PRESENCE OF FeCl$_2$ 79
3. REACTIVITY OF ($^{4-TBU}$BPY)Ni(2-NAPHTHENYLMETHYL)(PHTHALIMIDO) WITH ARYL IODIDE IN THE PRESENCE OF FeCl$_3$ 80
4. REDUCTION OF ($^{4-TBU}$BPY)Ni(2-NAPHTHENYLMETHYL)(PHTHALIMIDO) WITH DECAMETHYLCOBALTACENE 81
5. REACTIVITY OF ($^{4-TBU}$BPY)Ni(4-TRIFLUOROMETHYL PHENYL)(PHTHALIMIDO) IN THE PRESENCE OF FeCl$_2$ 82
6. REACTIVITY OF ($^{4-TBU}$BPY)Ni(4-TRIFLUOROMETHYL PHENYL)(PHTHALIMIDO) IN THE PRESENCE OF FeCl$_3$ 83
7. STABILITY OF ($^{4-TBU}$BPY)Ni(4-TRIFLUOROMETHYL PHENYL)(PHTHALIMIDO) 84
8. STABILITY OF ($^{4-TBU}$BPY)Ni(2-NAPHTHYLENYLMETHYL)(PHTHALIMIDO) 85
D. CYCLIC VOLTAMMETRY AND DIFFERENTIAL PULSE VOLTAMMETRY EXPERIMENTS 87
1. CV OF ($^{4-TBU}$BPY)Ni(2-NAPHTHYLENYLMETHYL)(PHTHALIMIDO) 87
2. DPV OF ($^{4-TBU}$BPY)Ni(2-NAPHTHYLENYLMETHYL)(PHTHALIMIDO) 88
IX. CRYSTALLOGRAPHIC EXPERIMENTS....89
A. DATA COLLECTION....89
B. STRUCTURE SOLUTION AND REFINEMENT 90
C. CRYSTALLOGRAPHIC CHARACTERIZATION 93
X. COMPOUND CHARACTERIZATION 127
4-(*TERT*-BUTYL)-N-CYANOPICOLINIMIDAMIDE ($^{4-TBU}$PYCAM^{CN})....127
1,3-DIOXOISOINDOLIN-2-YL 2,3-DIHYDRO-1H-INDENE-2-CARBOXYLATE....128
1-CHLORO-3-(PHENOXYMETHYL)-5-(TRIFLUOROMETHYL)BENZENE (1A) 128
1-CHLORO-3-(PHENOXYMETHYL)-5-(TRIFLUOROMETHYL)BENZENE (1B)....129
1-BROMO-3-(PHENOXYMETHYL)-5-(TRIFLUOROMETHYL)BENZENE (2)....130
3-(PHENOXYMETHYL)-5-(TRIFLUOROMETHYL)PHENYL TRIFLUOROMETHANESULFONATE (3)....130
1-(PHENOXYMETHYL)-4-(TRIFLUOROMETHYL)BENZENE (4)....131
4-(PHENOXYMETHYL)PHENYL ACETATE (5)....132
1-FLUORO-2-(PHENOXYMETHYL)BENZENE(6)....132
1-METHYL-2-(PHENOXYMETHYL)BENZENE (7) 133
1-(PHENOXYMETHYL)-4-(TRIFLUOROMETHOXY)BENZENE (8) 133

1-METHOXY-4-(PHENOXYMETHYL)BENZENE (9) 134
1-(*TERT*-BUTYL)-4-(PHENOXYMETHYL)BENZENE (10) 135
4,4,5,5-TETRAMETHYL-2-(4-(PHENOXYMETHYL)PHENYL)-1,3,2-DIOXABOROLANE (11) 135
5-(PHENOXYMETHYL)FURAN-2-CARBALDEHYDE (12) 136
3-(PHENOXYMETHYL)BENZO[B]THIOPHENE (13) 137
TERT-BUTYL-5-(PHENOXYMETHYL)-1H-INDOLE-1-CARBOXYLATE (14) 137
2-(PHENOXYMETHYL)PYRIDINE (15) 138
4-((3-CHLORO-5-(TRIFLUOROMETHYL)BENZYL)OXY)BENZONITRILE (21A) 139
1-(4-((3-CHLORO-5-(TRIFLUOROMETHYL)BENZYL)OXY)PHENYL)ETHAN-1-ONE (21B) 139
1-CHLORO-3-((4-CHLOROPHENOXY)METHYL)-5-(TRIFLUOROMETHYL)BENZENE (21C) 140
1-CHLORO-3-(1-PHENOXYETHYL)-5-(TRIFLUOROMETHYL)BENZENE (22) 141
2-(3-CHLORO-5-(TRIFLUOROMETHYL)PHENYL)-2,3-DIHYDROBENZOFURAN (23) 141
2-(3-CHLORO-5-(TRIFLUOROMETHYL)BENZYL)NAPHTHALENE (24) 142
3-(3-CHLORO-5-(TRIFLUOROMETHYL)BENZYL)BENZO[B]THIOPHENE (25) 143
TERT-BUTYL (3-CHLORO-5-(TRIFLUOROMETHYL)BENZYL)CARBAMATE (26) 143
TERT-BUTYL-2-(3-CHLORO-5-(TRIFLUOROMETHYL)PHENYL)AZETADINE-1-CARBOXYLATE (27) 144
BENZYL-2-(3-CHLORO-5-(TRIFLUOROMETHYL)PHENYL)PYRROLIDINE-1-CARBOXYLATE (28) 145
TERT-BUTYL 3-(3-CHLORO-5-(TRIFLUOROMETHYL)PHENYL)PYRROLIDINE-1-CARBOXYLATE (29) 146
2-(3-CHLORO-5-(TRIFLUOROMETHYL)PHENYL)-2,3-DIHYDRO-1H-INDENE (30) 146
1-CHLORO-3-CYCLOPENTYL-5-(TRIFLUOROMETHYL)BENZENE (31) 147
2-(3-CHLORO-5-(TRIFLUOROMETHYL)PHENYL)-1,2,3,4-TETRAHYDRONAPHTHALENE (32) 148
1-CHLORO-3-CYCLOHEXYL-5-(TRIFLUOROMETHYL)BENZENE (33) 148
1-CHLORO-3-(4,4-DIFLUOROCYCLOHEXYL)-5-(TRIFLUOROMETHYL)BENZENE (34) 149
1-CHLORO-3-(1-PHENYLPROPAN-2-YL)-5-(TRIFLUOROMETHYL)BENZENE (35) 150
1-CHLORO-3-(HEPTAN-2-YL)-5-(TRIFLUOROMETHYL)BENZENE (36) 151
4-(3-CHLORO-5-(TRIFLUOROMETHYL)PHENYL)-1-METHYLPIPERIDINE (37) 151
4-(3-CHLORO-5-(TRIFLUOROMETHYL)PHENYL)TETRAHYDRO-2H-THIOPYRAN (38) 152
METHYL 4-(3-CHLORO-5-(TRIFLUOROMETHYL)PHENYL)CYCLOHEXANE-1-CARBOXYLATE (39) 153
3-(3-CHLORO-5-(TRIFLUOROMETHYL)PHENYL)TETRAHYDROFURAN (40) 154
(^{TBU}BPY)NI(2-NAPHTHYLENYLMETHYL)(PHTHALIMIDO) (46) 155
XI. NMR SPECTRA 156
^{1}H NMR SPECTRUM OF 4-(*TERT*-BUTYL)-CYANOPICOLINIMIDAMIDE (500 MHZ, $CDCl_3$) 156
^{13}C{^{1}H} NMR SPECTRUM OF 4-(*TERT*-BUTYL)-N-CYANOPICOLINIMIDAMIDE 157
^{1}H NMR SPECTRUM OF 1,3-DIOXOISOINDOLIN-2-YL 2,3-DIHYDRO-1H-INDENE-2-CARBOXYLATE 158

$^{13}C\{^{1}H\}$ NMR SPECTRUM OF 1,3-DIOXOISOINDOLIN-2-YL 2,3-DIHYDRO-1H-INDENE-2-CARBOXYLATE..159
^{1}H NMR SPECTRUM OF 1A 160
$^{13}C\{^{1}H\}$ NMR SPECTRUM OF 1A 161
^{19}F NMR SPECTRUM OF 1A 162
^{1}H NMR SPECTRUM OF 1B 163
$^{13}C\{^{1}H\}$ NMR SPECTRUM OF 1B 164
^{19}F NMR SPECTRUM OF 1B 165
^{1}H NMR SPECTRUM OF 2 166
$^{13}C\{^{1}H\}$ NMR SPECTRUM OF 2 167
^{19}F NMR SPECTRUM OF 2 168
^{1}H NMR SPECTRUM OF 3 169
$^{13}C\{^{1}H\}$ NMR SPECTRUM OF 3 170
^{19}F NMR SPECTRUM OF 3 171
^{1}H NMR SPECTRUM OF 4 172
$^{13}C\{^{1}H\}$ NMR SPECTRUM OF 4 173
^{19}F NMR SPECTRUM OF 4 174
^{1}H NMR SPECTRUM OF 5 175
$^{13}C\{^{1}H\}$ NMR SPECTRUM OF 5 176
^{1}H NMR SPECTRUM OF 6 177
$^{13}C\{^{1}H\}$ NMR SPECTRUM OF 6 178
^{19}F NMR SPECTRUM OF 6 179
^{1}H NMR SPECTRUM OF 7 180
$^{13}C\{^{1}H\}$ NMR SPECTRUM OF 7 181
^{1}H NMR SPECTRUM OF 8 182
$^{13}C\{^{1}H\}$ NMR SPECTRUM OF 8 183
^{19}F NMR SPECTRUM OF 8 184
^{1}H NMR SPECTRUM OF 9 185
$^{13}C\{^{1}H\}$ NMR SPECTRUM OF 9 186
^{1}H NMR SPECTRUM OF 10 187
$^{13}C\{^{1}H\}$ NMR SPECTRUM OF 10 188
^{1}H NMR SPECTRUM OF 11 189
$^{13}C\{^{1}H\}$ NMR SPECTRUM OF 11 190
^{11}B NMR SPECTRUM OF 11 191
^{1}H NMR SPECTRUM OF 12 192
$^{13}C\{^{1}H\}$ NMR SPECTRUM OF 12 193

^{1}H-^{13}C HSQC OF 12 194
^{1}H NMR SPECTRUM OF 13 195
$^{13}C\{^{1}H\}$ NMR SPECTRUM OF 13 196
^{1}H NMR SPECTRUM OF 14 197
$^{13}C\{^{1}H\}$ NMR SPECTRUM OF 14 198
^{1}H NMR SPECTRUM OF 15 199
$^{13}C\{^{1}H\}$ NMR SPECTRUM OF 15 200
^{1}H NMR SPECTRUM OF 21A 201
$^{13}C\{^{1}H\}$ NMR SPECTRUM OF 21A 202
^{19}F NMR SPECTRUM OF 21A 203
^{1}H NMR SPECTRUM OF 21B 204
$^{13}C\{^{1}H\}$ NMR SPECTRUM OF 21B 205
^{19}F NMR SPECTRUM OF 21B 206
^{1}H NMR SPECTRUM OF 21C 207
$^{13}C\{^{1}H\}$ NMR SPECTRUM OF 21C 208
^{19}F NMR SPECTRUM OF 21C 209
^{1}H NMR SPECTRUM OF 22 210
$^{13}C\{^{1}H\}$ NMR SPECTRUM OF 22 211
^{19}F NMR SPECTRUM OF 22 212
^{1}H NMR SPECTRUM OF 23 213
$^{13}C\{^{1}H\}$ NMR SPECTRUM OF 23 214
^{19}F NMR SPECTRUM OF 23 215
^{1}H NMR SPECTRUM OF 24 216
$^{13}C\{^{1}H\}$ NMR SPECTRUM OF 24 217
^{19}F NMR SPECTRUM OF 24 218
^{1}H NMR SPECTRUM OF 25 219
$^{13}C\{^{1}H\}$ NMR SPECTRUM OF 25 220
^{19}F NMR SPECTRUM OF 25 221
^{1}H NMR SPECTRUM OF 26 222
$^{13}C\{^{1}H\}$ NMR SPECTRUM OF 26 223
^{19}F NMR SPECTRUM OF 26 224
^{1}H NMR SPECTRUM OF 27 225
$^{13}C\{^{1}H\}$ NMR SPECTRUM OF 27 226
^{19}F NMR SPECTRUM OF 27 227
^{1}H NMR SPECTRUM OF 28 228

$^{13}C\{^1H\}$ NMR SPECTRUM OF 28 229
^{19}F NMR SPECTRUM OF 28 230
^1H NMR SPECTRUM OF 29 231
$^{13}C\{^1H\}$ NMR SPECTRUM OF 29 232
^{19}F NMR SPECTRUM OF 29 233
^1H NMR SPECTRUM OF 30 234
$^{13}C\{^1H\}$ NMR SPECTRUM OF 30 235
^{19}F NMR SPECTRUM OF 30 236
^1H NMR SPECTRUM OF 31 237
$^{13}C\{^1H\}$ NMR SPECTRUM OF 31 238
^{19}F NMR SPECTRUM OF 31 239
^1H NMR SPECTRUM OF 32 240
$^{13}C\{^1H\}$ NMR SPECTRUM OF 32 241
^{19}F NMR SPECTRUM OF 32 242
^1H NMR SPECTRUM OF 33 243
$^{13}C\{^1H\}$ NMR SPECTRUM OF 33 244
^{19}F NMR SPECTRUM OF 33 245
^1H NMR SPECTRUM OF 34 246
$^{13}C\{^1H\}$ NMR SPECTRUM OF 34 247
^{19}F NMR SPECTRUM OF 34 248
^1H NMR SPECTRUM OF 35 249
$^{13}C\{^1H\}$ NMR SPECTRUM OF 35 250
^{19}F NMR SPECTRUM OF 35 251
^1H NMR SPECTRUM OF 36 252
$^{13}C\{^1H\}$ NMR SPECTRUM OF 36 253
^{19}F NMR SPECTRUM OF 36 254
^1H NMR SPECTRUM OF 37 255
$^{13}C\{^1H\}$ NMR SPECTRUM OF 37 256
^{19}F NMR SPECTRUM OF 37 257
^1H NMR SPECTRUM OF 38 258
$^{13}C\{^1H\}$ NMR SPECTRUM OF 38 259
^{19}F NMR SPECTRUM OF 38 260
^1H NMR SPECTRUM OF 39 MINOR DIASTEREOMER 261
$^{13}C\{^1H\}$ NMR SPECTRUM OF 39 MINOR DIASTEREOMER 262
^{19}F NMR SPECTRUM OF 39 MINOR DIASTEREOMER 263

^{1}H NMR SPECTRUM OF 39 MAJOR DIASTEREOMER 264
^{13}C{^{1}H} NMR SPECTRUM OF 39 MAJOR DIASTEREOMER..265
^{19}F NMR SPECTRUM OF 39 MAJOR DIASTEREOMER 266
^{1}H NMR SPECTRUM OF 40 267
^{13}C{^{1}H} NMR SPECTRUM OF 40..268
^{19}F NMR SPECTRUM OF 40..269
^{1}H NMR SPECTRUM OF ($^{4\text{-}TBU}$BPY)Ni(2-NAPHTHYLENYLMETHYL)(PHTHALIMIDO) 46.........................270
^{13}C{^{1}H} NMR SPECTRUM OF ($^{4\text{-}TBU}$BPY)Ni(2-NAPHTHYLENYLMETHYL)(PHTHALIMIDO) 46.........................271
^{1}H-^{13}C HSQC OF ($^{4\text{-}TBU}$BPY)Ni(2-NAPHTHYLENYLMETHYL)(PHTHALIMIDO) 46...272

I. General Information

Materials.

Unless otherwise stated, all reagents and substrates are commercial, purchased as reagent grade, and used without further purification. Reagents were sourced from Sigma-Aldrich, Oakwood Chemical, Combi-Blocks, TCI, AmBeed, Honeywell, Strem Chemicals, and J.T. Baker. $FeCl_3$, $Ni(NO_3)_2{\cdot}6H_2O$, phthalimide, tetrabutylammonium iodide (TBAI) and 1,4-dioxane (reagent grade) were obtained from Oakwood Chemical. $^{4\text{-}t\mathrm{Bu}}$PyCam^{CN} was synthesized according to the literature procedure.[1] MeCN was obtained from Fisher Chemical (HPLC grade). For reactions where water was added, DI water was utilized.

For the synthesis of Ni complex (**44**) and (**46**), $Ni(COD)_2$ was purchased from Thermo Scientific Chemicals, stored in a N_2-filled glovebox, and used as received. Anhydrous THF (≥99.9%, inhibitor-free), anhydrous toluene (99.8%), and anhydrous diethyl ether (≥99.9%, contains BHT as inhibitor), were purchased from Sigma-Aldrich, stored in a N_2-filled glovebox, and used as received. CD_3CN was purchased from Sigma-Aldrich and dried over 3 Å molecular sieves and stored in an N_2 filled glovebox. For the stoichiometric experiments, $FeCl_2$ (98%) was purchased from Strem Chemicals and $FeCl_3$ (97%) was purchased from Sigma-Aldrich. Both Fe salts were stored in a N_2-filled glovebox and used as received. Anhydrous MeCN (99.9%, Extra Dry) was purchased from Thermo Scientific Chemicals, stored in a N_2-filled glovebox, and used as received.

Methods.

Masses were measured using either an Accuris instruments model W3101A-120 or VWR model VWR-224AC balance. Reactions were monitored using thin-layer chromatography on glass-backed Uniplate plates with GHLF 250 µm silica gel. Compounds were visualized using Ultraviolet (UV)-light (254 nm), potassium permanganate ($KMnO_4$), or phosphomolybdic acid stain (PMA). Rotary evaporation was accomplished using either a Heidolph Hei-VAP Core or Hei-Vap Ultimate rotary evaporator at 25-35 °C and <50 torr. Silica gel column chromatography used SiliaFlash P60 silica gel with 230-400 mesh (40-63 µm particle size). Unless otherwise noted, solvents were dried using 4 Å molecular sieves for a minimum of 12 hours prior to use. Sieves were activated by vacuum oven at 150 °C for 24 hours.

Instrumentation.

NMR spectra were recorded on a Bruker spectrometer equipped with a 5 mm iProbe (^{1}H at 400 MHz, 500 MHz or 600 MHz, ^{13}C at 101 MHz or 201 MHz, ^{19}F at 377 MHz, and ^{11}B at 128 MHz). Chemical shifts for protons and carbons are reported in parts per million downfield from tetramethylsilane. ^{1}H NMR spectra a^{13}C NMR spectra are internally referenced to $CDCl_3$.[2,3] NMR data are represented as follows: chemical shift (ppm), multiplicity (br = broad, s = singlet, d = doublet, t = triplet, q = quartet,

---

[1] Hansen, E. C.; Pedro, D. J.; Wotal, A. C.; Gower, N.J.; Nelson, J. D.; Carson, S.; Weix, D. J. *Nat.Chem.* **2016**, *8,*1126–1130.

[2] Fulmer, G. R.; Miller, A. J. M.; Sherden, N. H.; Gottlieb, H. E.; Nudelman, A.; Stoltz, B. M.; Bercaw, J. E; Goldberg, K. I. *Organometallics* **2010**, *29*, 2176–2179.

[3] Gottlieb, H. E.; Kotlyar, V.; Nudelman, A. *J. Org. Chem.* **1997**, *62*, 7512–7515.

p = pentet, m = multiplet), coupling constant in Hertz (Hz), integration. Data for ^{1}H-decoupled ^{13}C NMR spectra are reported in terms of chemical shift; multiplicity and coupling constants are included only in the case of coupling with ^{19}F nuclei.

Ultraviolet-visible (UV-vis) absorbance spectra were collected on a Shimadzu UV-3600 UV-Vis-NIR spectrophotometer.

GC analyses were performed on an Agilent 7890A GC equipped with dual DB-5 columns (20 m × 180 μm × 0.18 μm), dual FID detectors, and hydrogen as the carrier gas. A sample volume of 1 μL was injected at a temperature of 300 °C and a 100:1 split ratio. The initial inlet pressure was 20.3 psi but varied as the column flow was held constant at 1.8 mL/min for the duration of the run. The initial oven temperature of 50 °C was held for 0.46 min followed by a temperature ramp of 65 °C/min up to 300 °C. The total run time was 5.0 min and the FID temperature was 325 °C. To authenticate product, GC/MS analyses were performed on a Shimadzu GCMS-QP2010 equipped with an RTX-5MS column (30 m × 0.25 mm × 0.25 μm) with a quadrupole mass analyzer using helium as the carrier gas. The analysis method used in all cases was 1 μL injection of sample, an injection temp of 250 °C, and a 20:1 split ratio. The initial inlet pressure was 8.1 psi, but varied as the column flow was held constant at 1.0 mL/min for the duration of the run. The interface temperature was held at 275 °C, and the ion source (EI^+, 30 eV) was held at 200 °C. The initial oven temperature was held at 60 °C for 1 min with the detector off, followed by a temperature ramp, with the detector on, to 300 °C at 20 °C/min. The total run time was 13.00 min.Alternatively, GC/MS analyses were performed on a Agilent 5977C GC/MSD equipped with an HP-5MS UI column (30 m × 0.25 mm × 0.25 μm) with a quadrupole mass analyzer using helium as the carrier gas. The analysis method used in all cases was 1 μL injection of sample, an injection temp of 250 °C, and a 20:1 split ratio. The initial inlet pressure was 20.792 psi, but varied as the column flow was held constant at 1.8 mL/min for the duration of the run. The interface temperature was held at 280 °C, and the ion source (EI^+, 70 eV) was held at 280 °C. The initial oven temperature was held at 60 °C for 0.5 min, followed by a temperature ramp to 300 °C at 60 °C/min. The detector was turned on after 1.80 min. The total run time was 6.50 min.

High-resolution ESI and atmospheric solid analysis probe mass spectra was obtained on a Waters SYNAPT G2-Si mass spectrometer. High resolution electron impact ionization and chemical ionization was obtained on a VG 70-VSE mass spectrometer.

Cyclic voltammetry and differential pulse voltammetry were performed in a N_2 filled glovebox utilizing a Pine Research WaveNowXV potentiostat. The working electrode was a 3.0 mm glassy carbon electrode that was polished with an alumina slurry prior to being rinsed, dried, and taken into the glovebox. The counter electrode was a 0.5 mm diameter platinum wire. Measurements were performed in anhydrous MeCN from Sigma Aldrich which was opened and stored in a glovebox. All measurements were acquired at 32 °C.

To obtain crystal structures for the Ni complexes a Bruker D8 VENTURE Photon III X-ray diffractometer was used.

Light Sources.

Photocatalytic reactions were performed using Kessil PR160L lights (purchased from Kessil: https://kessil.com/products/science_main.php) with a maximum wavelength of the specified wavelength-370, 390, 427, 440, 456, and 525 nm at 100% intensity. A Kessil fan rig was used to

maintain temperatures at 30-35 °C. When the fan is omitted from the reaction setup the vials are heated to 45 °C.

II. Reaction Setup

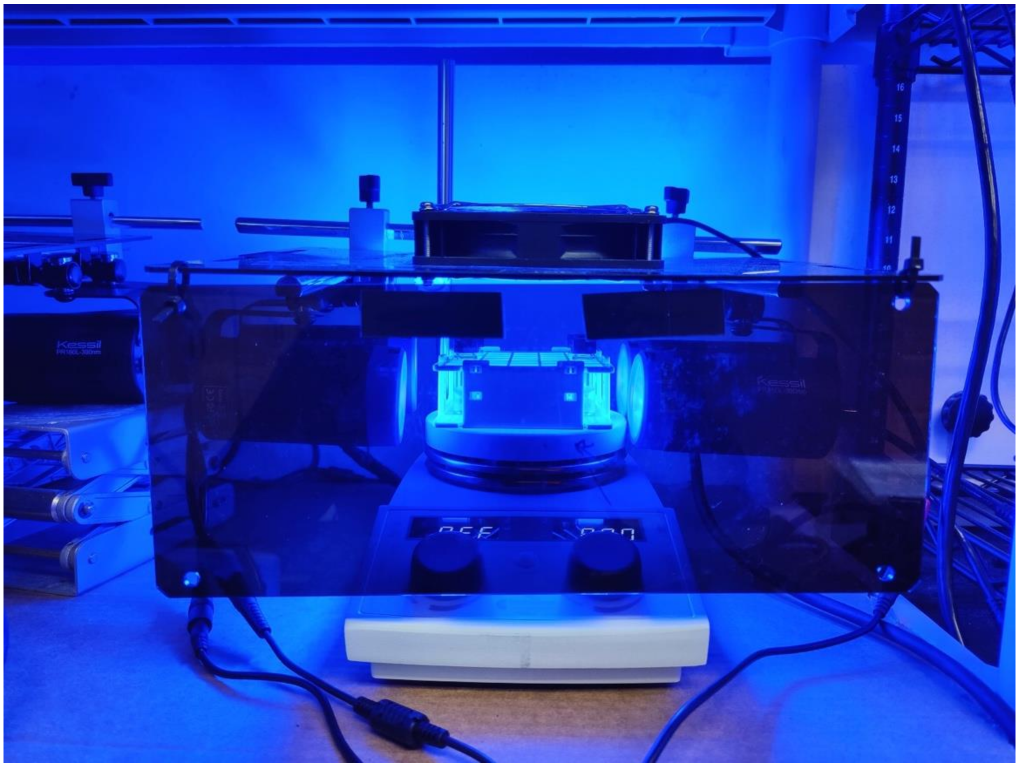

Figure S1. Kessil Rig Setup (Kessil, KSPRRM03) equipped with 40 W, 390 nm Kessil Lamps and a Magnetic Stirrer.

III. Experimental Procedures

A. General Procedure for Optimization in the Decarboxylative Arylation

1. Examination of Ligands for the Reaction of 2-Phenoxyacetic Acid (0.1 mmol scale) with 1-Bromo-3-iodo-5-(trifluoromethyl)benzene in MeCN

i. Varying Ligands on Ni

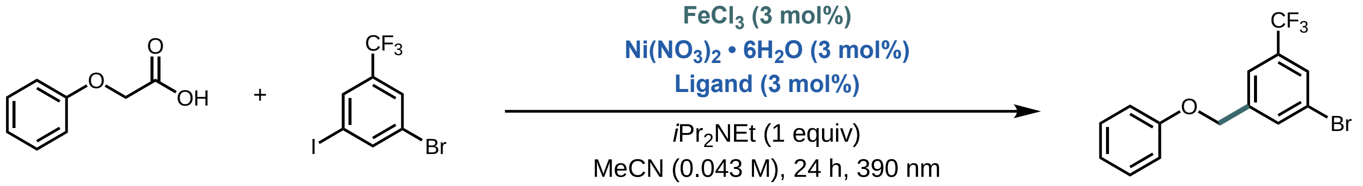

Ni complex solution. In a 1-dram vial (Fisherbrand Class A Glass Vial, 0333925B) equipped with a PTFE-coated stir bar (VWR spinbar micro, 3 × 10 mm, 58948-375), a 0.0300 M solution of $Ni(NO_3)_2{\cdot}6H_2O$ (0.00300 mmol in 0.100 mL) was prepared in MeCN. In another vial, a 0.00333 M solution of ligand (0.00300 mmol in 0.900 mL) was also prepared and capped. To form the complex, the ligand solution was added to the Ni solution and was stirred at 60 °C for 30 min. The mixture was then removed from heating and used as communicated below.

Reaction setup. A 1-dram vial (Fisherbrand Class A Glass Vial, 0333925B) equipped with a PTFE-coated stir bar (VWR spinbar micro, 3 × 10 mm, 58948-375) was charged with MeCN (0.985 mL). To the reaction vial, 1.00 mL of the ligated Ni catalyst stock solution (0.00300 M) was added, which was prepared as described above. Following addition of Ni catalyst stock solution, 2-phenoxyacetic acid (15.2 mg, 0.100 mmol, 1.00 equiv) in 0.300 mL of dry MeCN was added. Next, 15.0 µL of a 0.200 M $FeCl_3$ solution (0.487 mg, 0.00300 mmol, 0.0300 equiv) in dry MeCN was added. This was followed by the addition of *i*Pr_2NEt (17.4 µL, 0.100 mmol, 1.00 equiv) and 1-bromo-3-iodo-5-(trifluoromethyl)benzene (16.1 µL, 0.100 mmol, 1.00 equiv). The vial was fitted with a cap secured with a PTFE septum (Chemglass CG-4910-01 and CV-4080-0013 respectively). A 21-gauge needle was used to sparge the solution under positive pressure of N_2 for 5 min. The reaction was then placed 4 cm away from a 390 nm Kessil lamp and irradiated for 24 h while stirring at 800 rpm. After 24 h, the reaction mixture was filtered through a pad of silica gel in a pasteur pipette fitted with glass wool and rinsed with diethyl ether (5.00 mL). The combined filtrate was concentrated *in vacuo*. ^{1}H NMR yields are reported using dibromomethane (6.95 µL, 0.100 mmol) as an internal standard in $CDCl_3$.

ii. *Varying ligands on Fe*

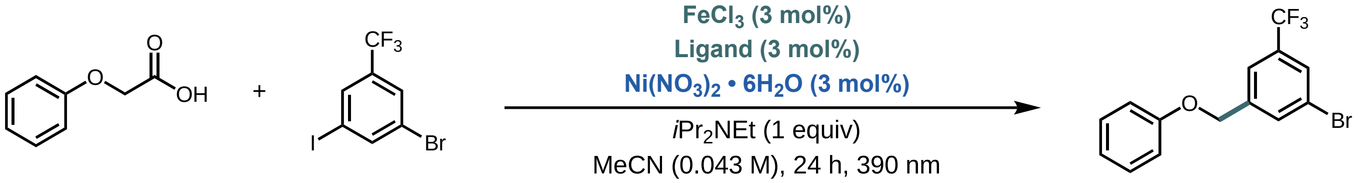

Fe complex solution. In a 1-dram vial (Fisherbrand Class A Glass Vial, 0333925B) equipped with a PTFE-coated stir bar (VWR spinbar micro, 3 × 10 mm, 58948-375), a 0.0300 M solution of $FeCl_3$ (0.00300 mmol in 0.100 mL) was prepared in MeCN. In another vial, a 0.00333 M solution of ligand (0.00300 mmol in 0.900 mL) was also prepared and capped. To form the complex, the ligand solution was added to the Fe solution and was stirred at 60 °C for 30 min. The mixture was then removed from heating and used as communicated below.

Reaction setup. A 1-dram vial (Fisherbrand Class A Glass Vial, 0333925B) equipped with a PTFE-coated stir bar (VWR spinbar micro, 3 × 10 mm, 58948-375) was charged with MeCN (0.300 mL). To the reaction vial, 1.00 mL of the Fe/ligand solution (0.00300 M) was added, which was prepared as described above. Following addition of the Fe stock solution, 2-phenoxyacetic acid (15.2 mg, 0.100 mmol, 1.00 equiv) in 0.500 mL of dry MeCN was added. Next, *i*Pr_2NEt (17.4 μL, 0.100 mmol, 1.00 equiv), 0.500 mL of a 0.00600 M $Ni(NO_3)_2$•$6H_2O$ solution (0.870 mg, 0.00300 mmol, 0.0300 equiv), and 1-bromo-3-iodo-5-(trifluoromethyl)benzene (16.1 μL, 0.100 mmol, 1.00 equiv) were added. The vial was fitted with a cap secured with a PTFE septum (Chemglass CG-4910-01 and CV-4080-0013 respectively). A 21-gauge needle was used to sparge the solution under positive pressure of N_2 for 5 min. The reaction was placed 4 cm away from a 390 nm Kessil lamp and irradiated for 24 h while stirring at 800 rpm. After 24 h, the reaction mixture was filtered through a pad of silica gel in a pasteur pipette fitted with glass wool and rinsed with diethyl ether (5.00 mL). The combined filtrate was concentrated *in vacuo*. ^1H NMR yields are reported using dibromomethane (6.95 μL, 0.100 mmol) as an internal standard in $CDCl_3$.

iii. *Varying ligands on Fe using $^{4\text{-}t\text{Bu}}$PyCam^{CN} as the ligand on Ni*

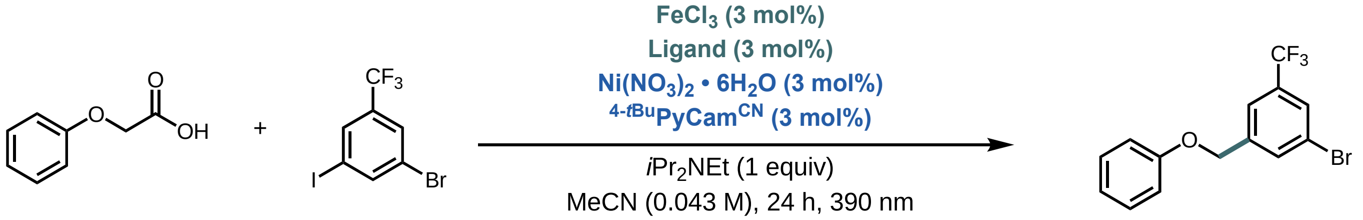

Ni complex solution. In a 1-dram vial (Fisherbrand Class A Glass Vial, 0333925B) equipped with a PTFE-coated stir bar (VWR spinbar micro, 3 × 10 mm, 58948-375), a 0.0300 M solution of $Ni(NO_3)_2$•$6H_2O$ (0.00300 mmol in 0.100 mL) was prepared in MeCN. In another vial, a 0.00333 M solution of $^{4\text{-}t\text{Bu}}$PyCam^{CN} (0.00300 mmol in 0.900 mL) was also prepared and capped. To form the complex, the $^{4\text{-}t\text{Bu}}$PyCam^{CN} solution was added to the Ni solution and was stirred at 60 °C for 30 min. The mixture was then removed from heating and used as communicated below.

Fe complex solution. In a 1-dram vial (Fisherbrand Class A Glass Vial, 0333925B) equipped with a PTFE-coated stir bar (VWR spinbar micro, 3 × 10 mm, 58948-375), a 0.0300 M solution of $FeCl_3$ (0.00300 mmol in 0.100 mL) was prepared in MeCN. In another vial, a 0.00333 M solution of ligand (0.00300 mmol in 0.900 mL) was also prepared and capped. To form the complex, the ligand solution was added to the Fe solution and was stirred at 60 °C for 30 min. The mixture was then removed from heating and used as communicated below.

Reaction setup. A 1-dram vial (Fisherbrand Class A Glass Vial, 0333925B) equipped with a PTFE-coated stir bar (VWR spinbar micro, 3 × 10 mm, 58948-375) was charged with MeCN (0.300 mL). To the reaction vial, 1.00 mL of the Fe catalyst solution (0.00300 M) was added which was prepared as described above. 2-phenoxyacetic acid (15.2 mg, 0.100 mmol, 1.00 equiv) was then added. This was followed by the addition of *i*Pr_2NEt (17.4 µL, 0.100 mmol, 1.00 equiv), 1.00 mL of the Ni catalyst stock solution (0.00300 M) as prepared above, and 1-bromo-3-iodo-5-(trifluoromethyl)benzene (16.1 µL, 0.100 mmol, 1.00 equiv). The vial was fitted with a cap secured with a PTFE septum (Chemglass CG-4910-01 and CV-4080-0013 respectively). A 21-gauge needle was used to sparge the solution under positive pressure of N_2 for 5 min. The reaction was placed 4 cm away from a 390 nm Kessil lamp and irradiated for 24 h while stirring at 800 rpm. After 24 h, the reaction mixture was filtered through a pad of silica gel in a pasteur pipette fitted with glass wool and rinsed with diethyl ether (5.00 mL). The combined filtrate was concentrated *in vacuo*. ^{1}H NMR yields are reported using dibromomethane (6.95 µL, 0.100 mmol) as an internal standard in $CDCl_3$.

2. Optimization of the Reaction of 2-Phenoxyacetic Acid (0.1 mmol scale) with 1-Bromo-3-iodo-5-(trifluoromethyl)benzene in MeCN

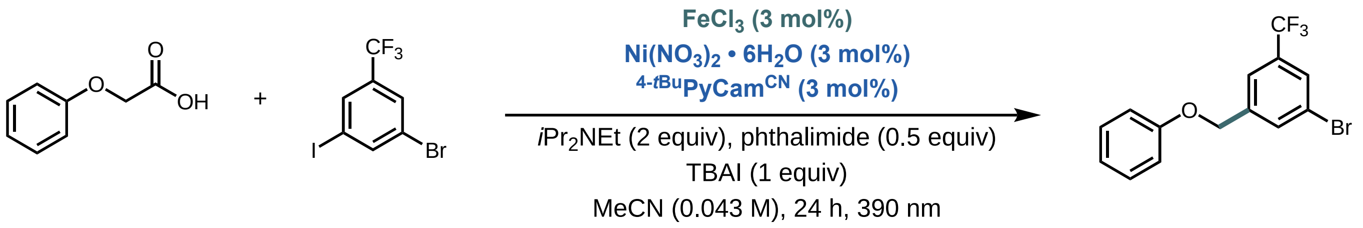

Ni complex solution. In a 1-dram vial (Fisherbrand Class A Glass Vial, 0333925B) equipped with a PTFE-coated stir bar (VWR spinbar micro, 3 × 10 mm, 58948-375), a 0.0300 M solution of $Ni(NO_3)_2{\cdot}6H_2O$ (0.00300 mmol in 0.100 mL) was prepared in MeCN. In another vial, a 0.00333 M solution of $^{4\text{-}t\text{Bu}}$PyCam^{CN} (0.00300 mmol in 0.900 mL) was also prepared and capped. To form the complex, the $^{4\text{-}t\text{Bu}}$PyCam^{CN} solution was added to the stirring Ni solution and was stirred at 60 °C for 30 min. The mixture was then removed from heating and used as communicated below.

Reaction setup. A 1-dram vial (Fisherbrand Class A Glass Vial, 0333925B) equipped with a PTFE-coated stir bar (VWR spinbar micro, 3 × 10 mm, 58948-375) was charged with MeCN (0.985 mL). To the reaction vial, phthalimide (7.40 mg, 0.0500 mmol, 0.500 equiv) was added. This was followed by the addition of 2-phenoxyacetic acid (15.2 mg, 0.100 mmol, 1.00 equiv) in 0.150 mL of MeCN and TBAI (36.9 mg, 0.100 mmol, 1.00 equiv) in 0.150 mL of MeCN. Next, 15.0 µL of a 0.200 M $FeCl_3$ solution (0.486 mg, 0.00300 mmol, 0.0300 equiv) in dry MeCN was then added followed by base

(0.200 mmol, 2.00 equiv) and 1.00 mL of the Ni catalyst stock solution (0.00300 M). Finally, 1-bromo-3-iodo-5-(trifluoromethyl)benzene (16.1 μL, 0.100 mmol, 1.00 equiv) was added. The vial was fitted with a cap equipped with a PTFE septum (Chemglass CG-4910-01 and CV-4080-0013 respectively). A 21-gauge needle was used to sparge the solution under positive pressure of N_2 for 5 min. The reaction was placed 4 cm away from a 390 nm Kessil lamp and irradiated for 24 h while stirring at 800 rpm. After 24 h, the reaction mixture was filtered through a pad of silica gel in a pasteur pipette fitted with glass wool and rinsed with diethyl ether (5.00 mL). The combined filtrate was concentrated *in vacuo*. ^{1}H NMR yields are reported using dibromomethane (6.95 μL, 0.100 mmol) as an internal standard in $CDCl_3$.

3. Translation of Optimized Conditions to the Reaction of 2-Phenoxyacetic Acid (0.1 mmol scale) with 1-Chloro-3-iodo-5-(trifluoromethyl)benzene in 1,4-Dioxane

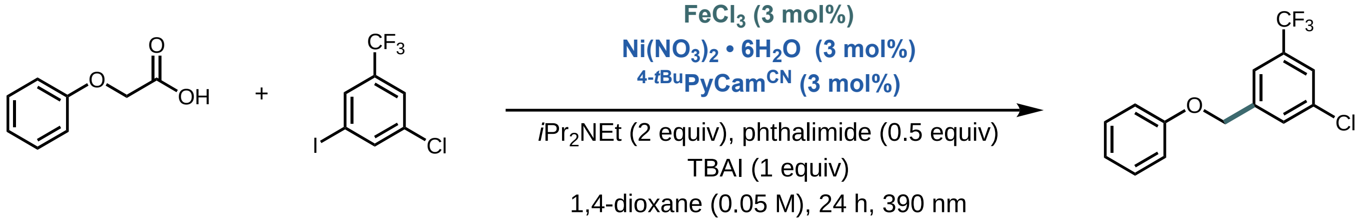

Ni complex solution. In a 20-mL scintillation vial (Fisherbrand Glass Vial, 03-337-15) equipped with a PTFE-coated stir bar (VWR spinbar stir bar, octagon, 58949-036), a 0.0300 M solution of $Ni(NO_3)_2{\cdot}6H_2O$ (0.0300 mmol, 8.70 mg in 1.00 mL) was prepared in 1,4-dioxane. In another vial, a 0.00333 M solution of $^{4\text{-}t\text{Bu}}$PyCam^{CN} (0.0300 mmol, 6.00 mg in 9.00 mL) was prepared. To form the complex, the $^{4\text{-}t\text{Bu}}$PyCam^{CN} solution was added to the Ni solution and was stirred at 60 °C for 30 min. The mixture was then removed from heating and used as communicated below.

Reaction setup. A 1-dram vial (Fisherbrand Class A Glass Vial, 0333925B) equipped with a PTFE-coated stir bar (VWR spinbar micro, 3 × 10 mm, 58948-375) was charged with 1,4-dioxane (0.985 mL). To the reaction vial, phthalimide (7.40 mg, 0.0500 mmol, 0.500 equiv) was added. This was followed by the addition of 2-phenoxyacetic acid (19.8 mg, 0.130 mmol, 1.30 equiv) and TBAI (36.9 mg, 0.100 mmol, 1.00 equiv) to the reaction vial. Next, 15.0 μL of a 0.200 M $FeCl_3$ solution (0.486 mg, 0.00300 mmol, 0.030 equiv) in 1,4-dioxane was then added followed by *i*Pr_2NEt (34.8 μL, 0.200 mmol, 2.00 equiv), and 1.00 mL of the Ni catalyst stock solution (0.00300 M). Finally, 1-chloro-3-iodo-5-(trifluoromethyl)benzene (15.7 μL, 0.100 mmol, 1.00 equiv) was added. The vial was fitted with a cap secured with a PTFE septum (Chemglass CG-4910-01 and CV-4080-0013 respectively). A 21-gauge needle was used to sparge the solution under positive pressure of N_2 for 5 min. The reaction was placed 4 cm away from a 390 nm Kessil lamp and irradiated for 24 h while stirring at 800 rpm. After 24 h, the reaction mixture was filtered through a pad of silica gel in a pasteur pipette fitted with glass wool and rinsed with diethyl ether (5.00 mL). The combined filtrate was concentrated *in vacuo*. ^{1}H NMR yields are reported using dibromomethane (6.95 μL, 0.100 mmol) as an internal standard in $CDCl_3$.

B. General Procedure for Aryl Iodide Scope (0.5 mmol scale) in the Decarboxylative Arylation

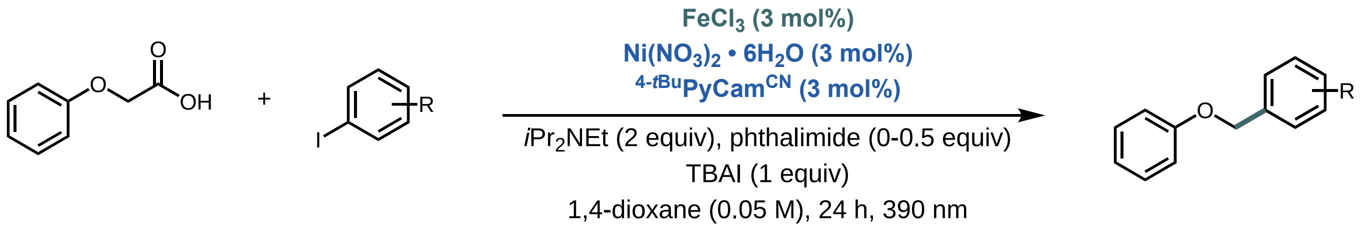

Ni complex solution. In a 20-mL scintillation vial (Fisherbrand Glass Vial, 03-337-15) equipped with a PTFE-coated stir bar (VWR spinbar stir bar, octagon, 58949-036), a 0.0300 M solution of $Ni(NO_3)_2{\cdot}6H_2O$ (0.0150 mmol, 4.35 mg in 0.500 mL) was prepared in 1,4-dioxane. In another vial, a 0.00333 M solution of $^{4\text{-}t\text{Bu}}$PyCam^{CN} (0.0150 mmol, 3.00 mg in 4.50 mL) was prepared. To form the complex, $^{4\text{-}t\text{Bu}}$PyCam^{CN} solution was added to the Ni solution and was stirred at 60 °C for 30 min. The mixture was then removed from heating and used as communicated below.

Reaction setup. A 20-mL scintillation vial (Fisherbrand Glass Vial, 03-337-15) equipped with a PTFE-coated stir bar (VWR spinbar stir bar, octagon, 58949-036) was charged with 1,4-dioxane (4.85 mL). To the reaction vial phthalimide (37.0 mg, 0.250 mmol, 0.500 equiv) was added. This was followed by the addition of 2-phenoxyacetic acid (98.8 mg, 0.650 mmol, 1.30 equiv) and TBAI (185 mg, 0.500 mmol 1.00 equiv) to the reaction vial. Next, 0.150 mL of a 0.100 M $FeCl_3$ solution (0.0150 mmol, 0.0300 equiv) in 1,4-dioxane was added, followed by *i*Pr_2NEt (174 μL, 1.00 mmol, 2.00 equiv) and 5.00 mL of the Ni catalyst stock solution (0.00300 M) which was prepared as described above. Finally, aryl iodide (0.500 mmol, 1.00 equiv) was added. The vial was fitted with a cap secured with a PTFE septum (Duran Wheaton Kimble W240845SP). A 21-gauge needle was used to sparge the solution under positive pressure of N_2 for 5 min. The reaction was placed 4 cm away from a 390 nm Kessil lamp and irradiated for 24 h while stirring at 800 rpm. After 24 h, the reaction mixture was filtered through a pad of silica gel in a pasteur pipette fitted with glass wool and was rinsed with diethyl ether (15.0 mL). The crude residue was purified by column chromatography on silica gel.

C. General Procedure for Carboxylic Acid Scope (0.5 mmol scale) in the Decarboxylative Arylation

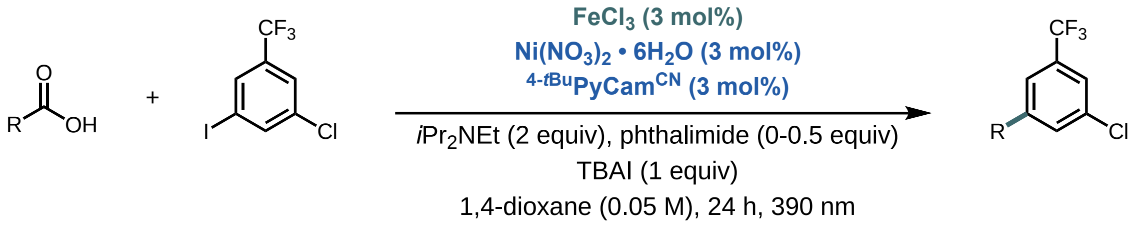

Ni complex solution. In a 20-mL scintillation vial (Fisherbrand Glass Vial, 03-337-15) equipped with a PTFE-coated stir bar (VWR spinbar stir bar, octagon, 58949-036), a 0.0300 M solution of $Ni(NO_3)_2{\cdot}6H_2O$ (0.0150 mmol, 4.35 mg in 0.500 mL) was prepared in 1,4-dioxane. In another vial, a 0.00333 M solution of $^{4\text{-}t\text{Bu}}$PyCam^{CN} (0.0150 mmol, 3.00 mg in 4.50 mL) was prepared. To form the complex, $^{4\text{-}t\text{Bu}}$PyCam^{CN} solution was added to the Ni solution and was stirred at 60 °C for 30 min. The mixture was then removed from heating and used as communicated below.

Reaction setup. A 20-mL scintillation vial (Fisherbrand Glass Vial, 03-337-15) equipped with a PTFE-coated stir bar (VWR spinbar stir bar, octagon, 58949-036) was charged with 1,4-dioxane (4.85 mL). To the reaction vial, phthalimide (37.0 mg, 0.250 mmol, 0.500 equiv) was added. This was followed by the addition of carboxylic acid (0.650 mmol, 1.30 equiv) and TBAI (185 mg, 0.500 mmol 1.00 equiv). 0.150 mL of a 0.100 M $FeCl_3$ solution (0.015 mmol, 0.030 equiv) in 1,4-dioxane. Next, *i*Pr_2NEt (174 µL, 1.00 mmol, 2.00 equiv) and 5.00 mL of the Ni catalyst stock solution (0.00300 M) which was prepared as described above were added. Finally, 1-chloro-3-iodo-5-(trifluoromethyl)benzene (78.5 µL, 0.500 mmol, 1.00 equiv) was added. The vial was fitted with a cap secured with a PTFE septum (Duran Wheaton Kimble W240845SP). A 21-gauge needle was used to sparge the solution under positive pressure of N_2 for 5 min. The reaction was placed 4 cm away from a 390 nm Kessil lamp and irradiated for 24 h while stirring at 800 rpm. After 24 h, the reaction mixture was filtered through a pad of silica gel in a pasteur pipette fitted with glass wool and was rinsed with diethyl ether (15.0 mL). The crude residue was purified by column chromatography on silica gel.

D. General Procedure for Unactivated Carboxylic Acid Scope (0.1 mmol scale) in the Decarboxylative Arylation

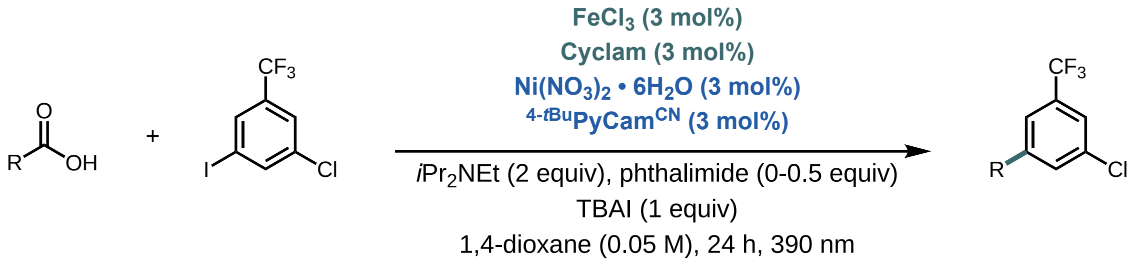

Ni complex solution. In a 1-dram vial (Fisherbrand Class A Glass Vial, 0333925B) equipped with a PTFE-coated stir bar (VWR spinbar micro, 3 × 10 mm, 58948-375), a 0.0300 M solution of $Ni(NO_3)_2{\cdot}6H_2O$ (0.00300 mmol, 0.870 mg in 0.100 mL) was prepared in MeCN. In another vial, a 0.00333 M solution of $^{4\text{-}t\text{Bu}}$PyCam^{CN} (0.00300 mmol, 0.600 mg in 0.900 mL) was also prepared and capped. To form the complex, the $^{4\text{-}t\text{Bu}}$PyCam^{CN} solution was added to the stirring Ni solution and was left to stir at 60 °C for 30 min. The mixture was then removed from heating and utilized as communicated below.

Fe complex solution. In a 1-dram vial (Fisherbrand Class A Glass Vial, 0333925B) equipped with a PTFE-coated stir bar (VWR spinbar micro, 3 × 10 mm, 58948-375), $FeCl_3$ (0.00300 mmol, 0.480 mg) and cyclam (0.0300 mmol, 0.600 mg) were dissolved in 1.00 mL of 1,4-dioxane. To form the complex, the mixture was stirred at 60 °C for 30 min. The mixture was then removed from heating and used as communicated below.

Reaction setup. A 1-dram vial (Fisherbrand Class A Glass Vial, 0333925B) equipped with a PTFE-coated stir bar (VWR spinbar micro, 3 × 10 mm, 58948-375) was charged with phthalimide as necessary (7.40 mg, 0.050 mmol, 0.500 equiv). This was followed by the addition of carboxylic acid (0.130 mmol, 1.30 equiv) and TBAI (36.9 mg, 0.100 mmol, 1.00 equiv). 1.00 mL of the Fe catalyst stock (0.00300 M) which was prepared as mentioned above. Next, *i*Pr_2NEt (34.8 µL, 0.200 mmol, 2.00 equiv) and 1.00 mL of the Ni catalyst stock solution, which was prepared as mentioned above, were added. This was followed by additives as necessary: Zn (1.00 mg, 0.015 mmol, 0.150 equiv) or $ZnCl_2$ (4.10 mg, 0.0300 mmol, 0.300 equiv) or 4-ethylpyridine (1.71 µL, 0.015 mmol, 0.150 equiv). Finally, 1-chloro-3-iodo-5-(trifluoromethyl)benzene (15.7 µL, 0.100 mmol, 1.00 equiv) was added. aThe vial was fitted with a cap secured with a PTFE septum (Chemglass CG-4910-01 and CV-4080-0013 respectively). A 21-gauge needle was used to sparge within the solution under positive pressure of N_2 for 5 min. The reaction was placed 4 cm away from a 390 nm Kessil lamp and irradiated for 24 h while stirring at 800 rpm. After 24 h, the reaction mixture was filtered through a pad of silica gel in a pasteur pipette fitted with glass wool and rinsed with diethyl ether (5.00 mL). The combined filtrate was concentrated *in vacuo*. ^{1}H NMR yields are reported using dibromomethane (6.95 µL, 0.100 mmol) as an internal standard in $CDCl_3$.

E. Procedure for Time Course Studies

1. 2-Phenoxyacetic Acid Coupled with 1-Chloro-3-iodo-5-(trifluoromethyl)benzene

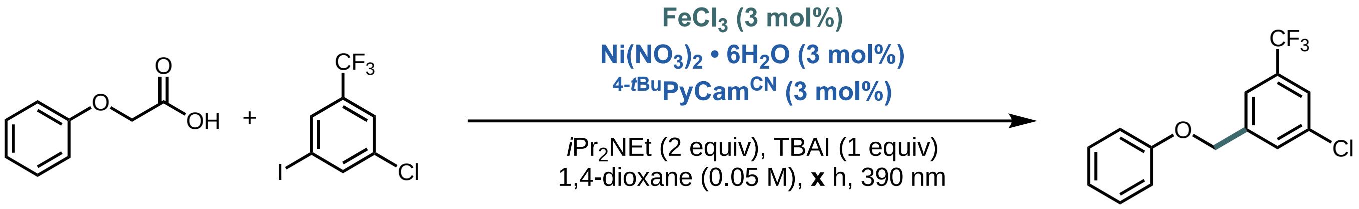

Ni complex solution. In a 20-mL scintillation vial (Fisherbrand Glass Vial, 03-337-15) equipped with a PTFE-coated stir bar (VWR spinbar stir bar, octagon, 58949-036), a 0.0300 M solution of $Ni(NO_3)_2{\cdot}6H_2O$ (0.0150 mmol, 4.35 mg in 0.500 mL) was prepared in 1,4-dioxane. In another vial, a 0.00333 M solution of $^{4\text{-}t\text{Bu}}$PyCam^{CN} (0.0150 mmol, 3.00 mg in 4.50 mL) was prepared. To form the complex, $^{4\text{-}t\text{Bu}}$PyCam^{CN} solution was added to the Ni solution and was stirred at 60 °C for 30 min. The mixture was then removed from heating and used as communicated below.

Reaction Setup. A 20-mL scintillation vial (Fisherbrand Glass Vial, 03-337-15) equipped with a PTFE-coated stir bar (VWR spinbar stir bar, octagon, 58949-036) was charged with 1,4-dioxane (4.85 mL). This was followed by the addition of 2-phenoxyacetic acid (98.5 mg, 0.65 mmol, 1.30 equiv) and TBAI (185 mg, 0.500 mmol 1.00 equiv) to the reaction vial. Next, 0.150 mL of a 0.100 M $FeCl_3$ solution (0.015 mmol, 0.030 equiv) in 1,4-dioxane was then added followed by *i*Pr_2NEt (174 µL, 1.00 mmol, 2.00 equiv) and 5.00 mL of the Ni catalyst stock solution (0.00300 M) which was prepared as described above. Biphenyl (77.1 mg, 0.500 mmol, 1.00 equiv) was added as an internal standard. Finally, 1-chloro-3-iodo-5-(trifluoromethyl)benzene (78.5 µL, 0.500 mmol, 1.00 equiv) was added. The vial was fitted with a cap secured with a PTFE septum (Duran Wheaton Kimble, W240845SP). A 21-gauge needle was used to sparge within the solution under positive pressure of N_2 for 5 min. The reaction was placed 4 cm away from a 390 nm Kessil lamp and irradiated for 24 h while stirring at 800 rpm with a N_2 balloon to maintain an inert atmosphere while taking aliquots. At every time point (0 min, 10 min, 30 min, 1 h, 2 h, 3 h, 4 h, 12 h, 20 h, and 24 h), aliquots of 100 µL each were taken out from the reaction. The reaction mixture was filtered through a pad of silica gel in a pasteur pipette fitted with glass wool and rinsed with diethyl ether (1.00 mL). The samples were analyzed by GC/MS and ^{1}H NMR spectroscopy to quantify the products and different byproducts. ^{1}H NMR yields are reported using 1,3,5-trimethoxybenzene as an internal standard in $CDCl_3$. GC/MS yields are reported using biphenyl as an internal standard.

2. 2,3-Dihydro-1H-indene-2-carboxylic Acid Coupled with 1-Chloro-3-iodo-5-(trifluoromethyl)benzene

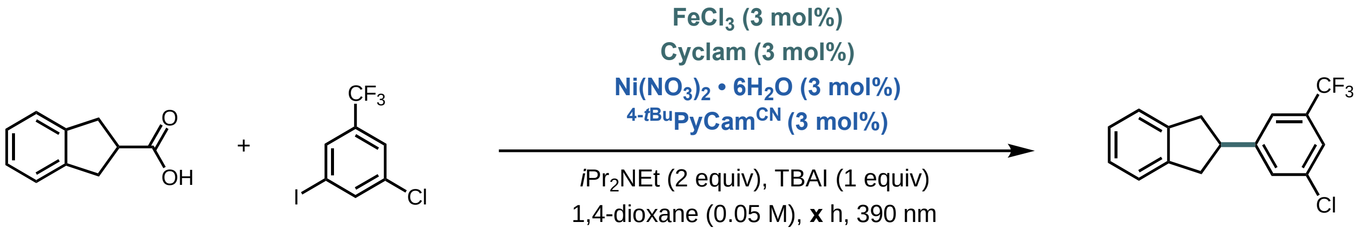

Ni complex solution. In a 20-mL scintillation vial (Fisherbrand Glass Vial, 03-337-15) equipped with a PTFE-coated stir bar (VWR spinbar stir bar, octagon, 58949-036), a 0.0300 M solution of $Ni(NO_3)_2$•$6H_2O$ (0.0150 mmol, 4.35 mg in 0.500 mL) was prepared in 1,4-dioxane. In another vial, a 0.00333 M solution of $^{4\text{-}t\text{Bu}}$PyCam^{CN} (0.0150 mmol, 3.00 mg in 4.50 mL) was prepared. To form the complex, $^{4\text{-}t\text{Bu}}$PyCam^{CN} solution was added to the Ni solution and was stirred at 60 °C for 30 min. The mixture was then removed from heating and used as communicated below.

Fe complex solution. In a 20-mL scintillation vial (Fisherbrand Glass Vial, 03-337-15) equipped with a PTFE-coated stir bar (VWR spinbar stir bar, octagon, 58949-036), $FeCl_3$ (0.0150 mmol, 2.43 mg) and cyclam (0.0150 mmol, 3.00 mg) were dissolved in 5.00 mL of 1,4-dioxane. To form the complex, the mixture was left to stir at 60 °C for 30 min. The mixture was then removed from heating and utilized as communicated below.

Reaction Setup. A 20-mL scintillation vial (Fisherbrand Glass Vial, 03-337-15) equipped with a PTFE-coated stir bar (VWR spinbar stir bar, octagon, 58949-036) was charged with 2,3-dihydro-1H-indene-2-carboxylic acid (105 mg, 0.650 mmol, 1.30 equiv) and TBAI (186 mg, 0.500 mmol 1.00 equiv). Next, 5.00 mL of the Fe catalyst stock solution (0.00300 M) ,which was prepared as mentioned above was added, followed by *i*Pr_2NEt (174 µL, 1.00 mmol, 2.00 equiv) and 5.00 mL of the Ni catalyst stock solution (0.00300 M) which was prepared as mentioned above. Biphenyl (77.1 mg, 0.500 mmol, 1.00 equiv) was added as an internal standard. Finally, 1-chloro-3-iodo-5-(trifluoromethyl)benzene (78.5 µL, 0.500 mmol, 1.00 equiv) was added. The vial was fitted with a cap secured with a PTFE septum (Duran Wheaton Kimble, W240845SP). A 21-gauge needle was used to sparge within the solution under positive pressure of N_2 for 5 min. The reaction was placed 4 cm away from a 390 nm Kessil lamp and irradiated for 24 h while stirring at 800 rpm with a N_2 balloon to maintain an inert atmosphere while taking aliquots. At every time point (0 min, 10 min, 30 min, 1 h, 2 h, 3 h, 4 h, 12 h, 20 h, and 24 h), aliquots of 100 µL each were taken out. The reaction mixture was filtered through a pad of silica gel in a pasteur pipette fitted with glass wool and rinsed with diethyl ether (1.00 mL). The samples were analyzed by GC/MS and ^{1}H NMR spectroscopy to quantify the product and different byproducts. ^{1}H NMR yields are reported using 1,3,5-trimethoxybenzene as an internal standard in $CDCl_3$. GC/MS yields are reported using biphenyl as an internal standard.

F. Procedure for the Synthesis of 1,3-Dioxoisoindolin-2-yl 2,3-dihydro-1H-indene-2-carboxylate

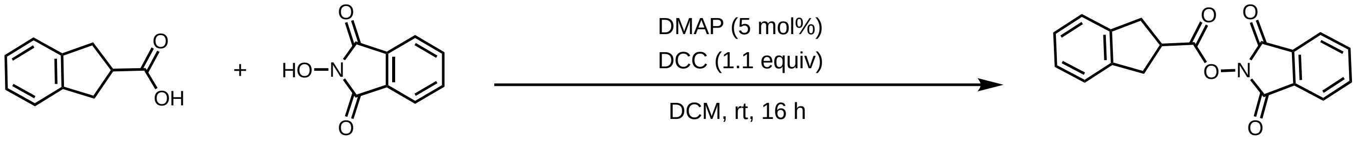

1,3-dioxoisoindolin-2-yl 2,3-dihydro-1H-indene-2-carboxylate was synthesized according to literature procedure.[4]

G. Procedure for the Synthesis of Ni complexes

1. Procedure for Synthesis of ($^{4\text{-}t\text{Bu}}$Bpy)Ni(4-trifluoromethylphenyl)(phthalimido) (**44**)

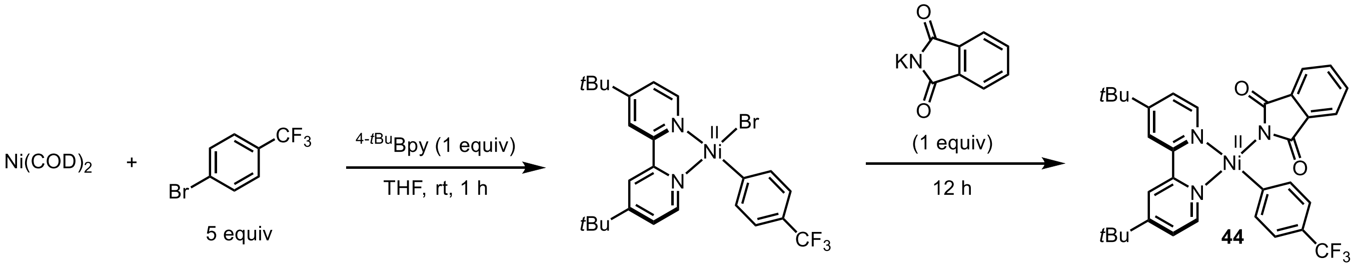

The title compound was prepared according to a modified literature procedure.[5] In a N_2-filled glovebox, an oven-dried 20-mL scintillation vial equipped with a PTFE-coated stir bar (VWR spinbar stir bar, octagon, 58949-036) was charged with Ni(COD)$_2$ (275.1 mg, 1.000 mmol, 1.00 equiv), $^{4\text{-}t\text{Bu}}$Bpy (268 mg, 1.00 mmol, 1.00 equiv), and THF (10.0 mL). The vial was sealed with a PTFE-coated screw cap, and the mixture was stirred for 1 h at room temperature. To the mixture was added 4-bromobenzotrifluoride (1.13 g, 5.00 mmol, 5.00 equiv) dropwise by syringe. The color of the mixture transitioned from dark indigo to dark red. After stirring at rt for 1 h, potassium phthalimide (185 mg, 1.00 mmol, 1.00 equiv) was added in one portion, and the mixture was allowed to stir for 12 h, resulting in the formation of a yellow orange solid. The mixture was filtered, and the solid collected was washed with pentane (3 × 5.00 mL). The solid was extracted with DCM (3 × 4.00 mL), filtered through celite, and the product purified by crystallization from vapor diffusion of Et_2O into the filtrate to give dark orange needles (168 mg, 27% yield). NMR characterization data matched those reported in the literature. The purity of the complex was measured by quantitative ^{1}H NMR to be 90% with DCM (3 wt%) and Et_2O (< 1 wt%) as residual solvent. The remaining unaccounted mass is attributed to NMR silent impurities.

---

[4] Zhang, Y-L.; Yang, L.; Wu, J.; Zhu, C.; Wang, P. *Org. Lett.* **2020**, *22,* 7768–7772.

[5] Prieto Kullmer, C. N.; Kautzky, J. A.; Krska, S. W.; Nowak, T.; Dreher, S. D.; MacMillan, D. W. C. *Science.* **2022**, 376, 532–539.

2. Procedure for Synthesis of ($^{4\text{-}t\text{Bu}}$Bpy)Ni(2-naphthalenylmethyl)(phthalimido) (**46**)

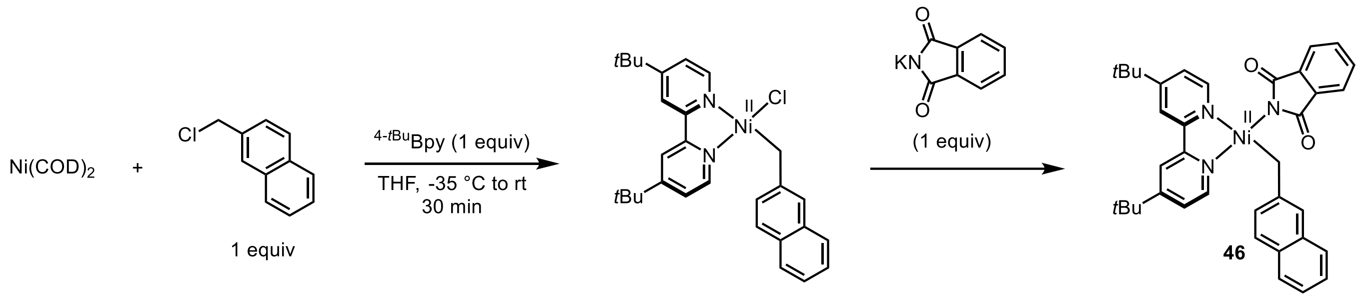

The intermediate oxidative addition complex was prepared following a modified literature procedure.[6] In a N_2-filled glovebox, an oven-dried 20-mL scintillation vial equipped with a PTFE-coated stir bar (VWR spinbar stir bar, octagon, 58949-036) was charged with $Ni(COD)_2$ (275.1 mg, 1.000 mmol, 1.00 equiv), $^{4\text{-}t\text{Bu}}$Bpy (268 mg, 1.00 mmol, 1.00 equiv), and THF (5.00 mL). The vial was sealed with a PTFE-coated screw cap, and the mixture was stirred for 1 h at rt. The vial was placed in the glovebox freezer to cool to -35 °C for 10 min. The vial was removed from the freezer, and a solution of 2-(chloromethyl)naphthalene (177 mg, 1.00 mmol, 1.00 equiv) in THF (1.00 mL) was added dropwise to the mixture while stirring and warming to rt, resulting in the formation of a dark purple precipitate. The mixture was allowed to stir for 30 min before potassium phthalimide (185 mg, 1.00 mmol, 1.00 equiv) was added in one portion. The mixture was allowed to stir at rt for 12 h, resulting in the disappearance of the purple precipitate and formation of a dark red solution. The mixture was filtered through celite, and the product purified by crystallization from vapor diffusion of pentane into the filtrate to give dark purple needles (350 mg, 57% yield). The purity of the complex was measured by quantitative ^{1}H NMR to be 89%. Dark precipitate was observed in the sample after NMR measurements over a period of 1 h. **Note**: Prolonged solutions of the complex in THF showed signs of decomposition to give a light-yellow solution and formation of green crystals.

[6] Zhang, Y.; Tanabe, Y.; Kuriyama, S.; Nishibayashi, Y. *Chem. A. Eur. J.* **2022**, 28, e202200727.

IV. Optimization

A. Ligands

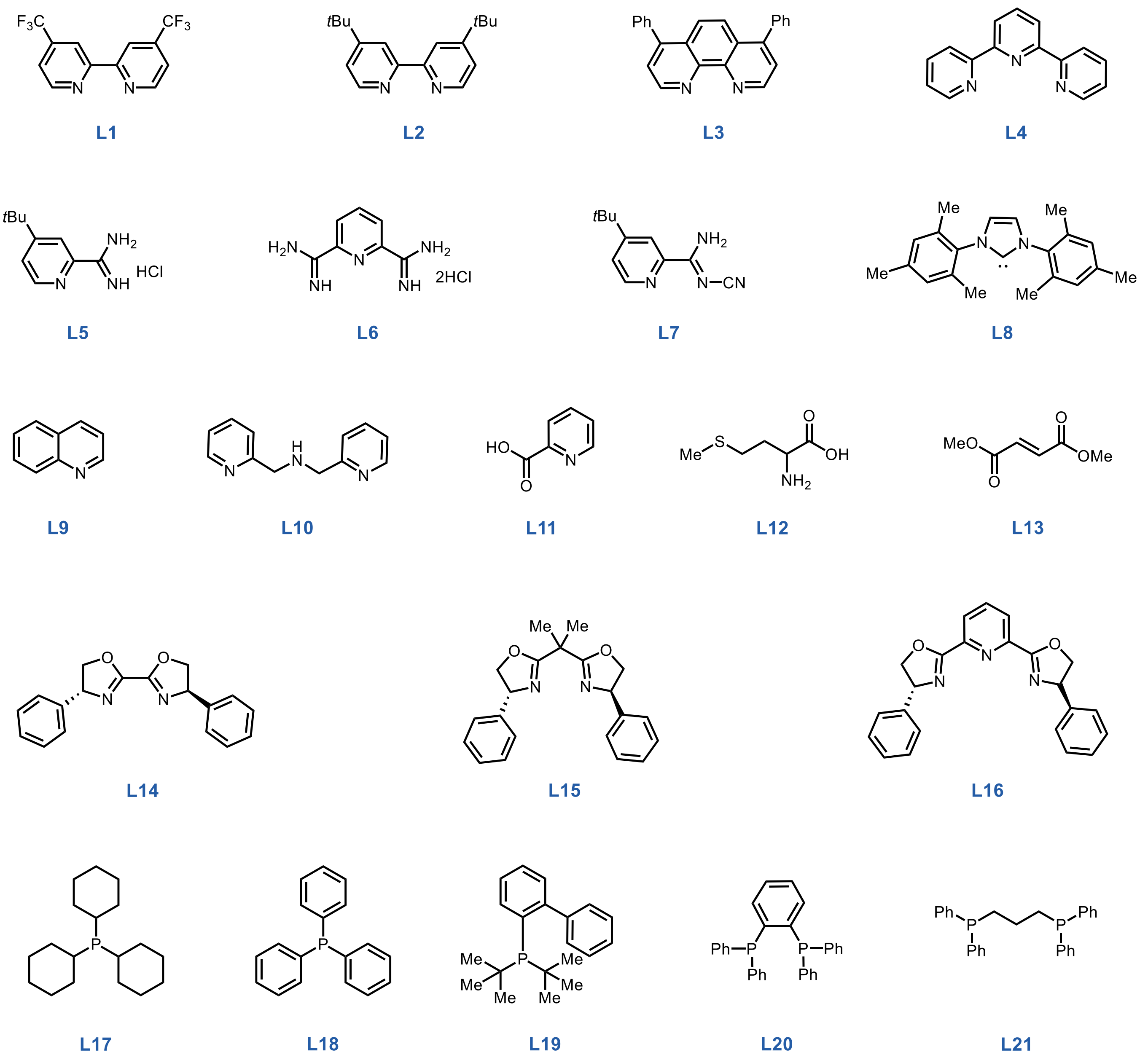

Figure S2. Structures of ligands evaluated in the optimization of the decarboxylative arylation.

1. Ligand optimization on Ni

Reaction yield was examined with different ligands on Ni according to the general procedure for optimization of ligand in the decarboxylative arylation (see section IIIA1i).

Table S1. Summary of the optimization of ligand for the Ni catalyst[a,b]

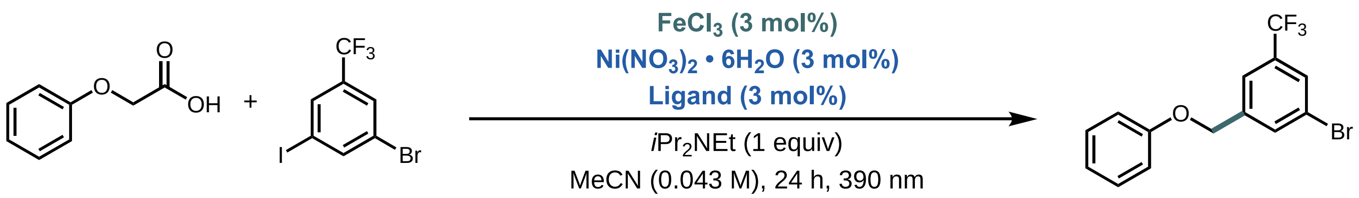

entry	ligand	product (%)
1	**no ligand**	trace
2	**L1**	5
3	**L2**	49
4	**L3**	7
5	**L4**	3
6	**L5**	50
7	**L6**	5
8	**L7**	60
9	**L8**	4
10	**L9**	3
11	**L10**	3
12	**L11**	11
13	**L12**	3
14	**L13**	4
15	**L14**	4
16	**L15**	2
17	**L16**	2
18	**L17**	4
19	**L18**	3
20	**L19**	4
21	**L20**	1
22	**L21**	2

[a] Reactions were set up following the general procedure for examination of ligand on Ni using phenoxyacetic acid (0.100 mmol, 1.00 equiv) and 1-bromo-3-iodo-5-(trifluoromethyl)benzene (0.100 mmol, 1.00 equiv) in 2.30 mL of MeCN. [b] Yields were determined by ^{1}H NMR spectroscopy using dibromomethane as an internal standard.

2. Ligand optimization on Fe

Reaction yield was examined with different ligands on Fe according to the general procedure for optimization of ligand in the decarboxylative arylation (see section IIIA1ii).

Table S2. Summary of the optimization of ligand for the Fe catalyst[a,b]

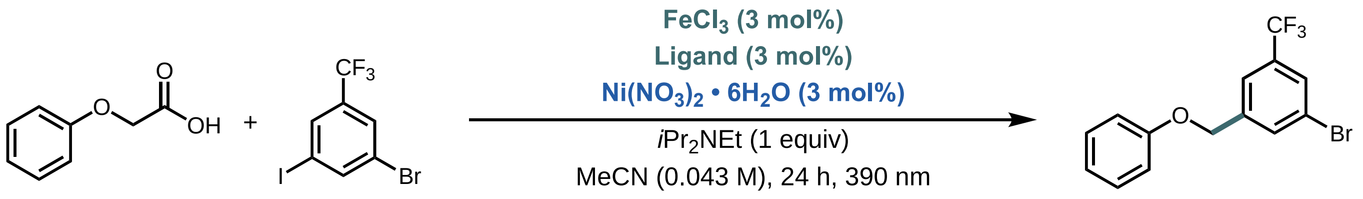

entry	ligand	product (%)
1	**no ligand**	trace
2	**L1**	5
3	**L2**	25
4	**L3**	9
5	**L4**	3
6	**L5**	51
7	**L6**	5
8	**L7**	56
9	**L8**	3
10	**L9**	5
11	**L10**	11
12	**L11**	7
13	**L12**	5
14	**L13**	4
15	**L14**	5
16	**L15**	5
17	**L16**	trace
18	**L17**	4
19	**L18**	3
20	**L19**	4
21	**L20**	2
22	**L21**	trace

[a] Reactions were set up following the general procedure for examination of ligand on Fe using phenoxyacetic acid (0.100 mmol, 1.00 equiv) and 1-bromo-3-iodo-5-(trifluoromethyl)benzene (0.100 mmol, 1.00 equiv) in 2.30 mL of MeCN. [b] Yields were determined by ^1H NMR spectroscopy using dibromomethane as an internal standard.

3. Ligand optimization on Fe with ${}^{4\text{-}t\mathrm{Bu}}$Pycam^{CN} on Ni

Reaction yield was examined with different ligands on Fe and ${}^{4\text{-}t\mathrm{Bu}}$PyCam^{CN} ligated to Ni according to the general procedure for optimization of ligand in the decarboxylative arylation (see section IIIA1iii).

Table S3. Summary of the optimization of ligand for the Fe catalyst using ${}^{4\text{-}t\mathrm{Bu}}$PyCam^{CN} as a ligand for the Ni catalyst[a,b]

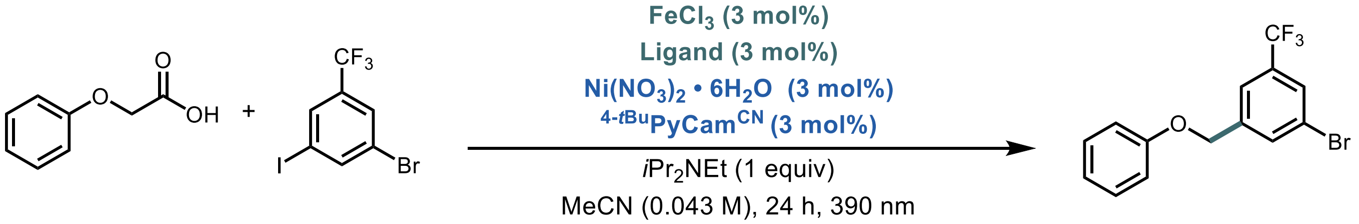

entry	ligand	product (%)
1	**no ligand**	trace
2	**L1**	11
3	**L2**	21
4	**L3**	19
5	**L4**	8
6	**L5**	48
7	**L6**	42
8	**L7**	27
9	**L8**	57
10	**L9**	58
11	**L10**	trace
12	**L11**	37
13	**L12**	50
14	**L13**	55
15	**L14**	55
16	**L15**	47
17	**L16**	21
18	**L17**	53
19	**L18**	53
20	**L19**	54
21	**L20**	8
22	**L21**	18

[a] Reactions were set up following the general procedure for examination of ligand on Fe with ${}^{4\text{-}t\mathrm{Bu}}$PyCam^{CN} as ligand on Ni using phenoxyacetic acid (0.100 mmol, 1.00 equiv) and 1-bromo-3-iodo-5-(trifluoromethyl)benzene (0.100 mmol, 1.00

equiv) in 2.30 mL of MeCN. [b] Yields were determined by ^{1}H NMR spectroscopy using dibromomethane as an internal standard.

B. Solvent

Reaction yield was examined with different solvents according to the general procedure for optimization in the decarboxylative arylation (see section IIIA2).

Table S4. Summary of the optimization of solvent[a,b]

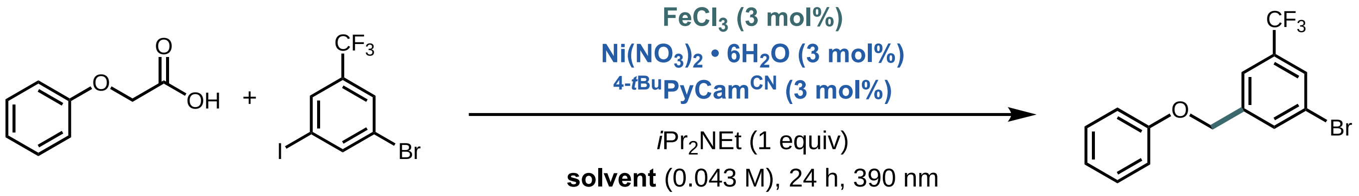

entry	solvent	product (%)
1	acetonitrile	60
2	N,N-dimethylacetamide	28
3	dimethyl sulfoxide	11
4	tetrahydrofuran	49
5	1,4-dioxane	57
6	ethyl acetate	52
7	ethanol	60
8	dichloroethane	53
9	dimethyl carbonate	63
10	toluene	51
11	hexane	1

[a] Reactions were set up following the general procedure for optimization in the decarboxylative arylation using 2-phenoxyacetic acid (0.100 mmol, 1.00 equiv) and 1-bromo-3-iodo-5-(trifluoromethyl)benzene (0.100 mmol, 1.00 equiv) in 2.30 mL of MeCN. [b] Yields were determined by ^{1}H NMR spectroscopy using dibromomethane as an internal standard.

C. Base

Reaction yield was examined with different bases according to the general procedure for optimization in the decarboxylative arylation (see section IIIA2).

Table S5. Summary of the optimization of base[a,b]

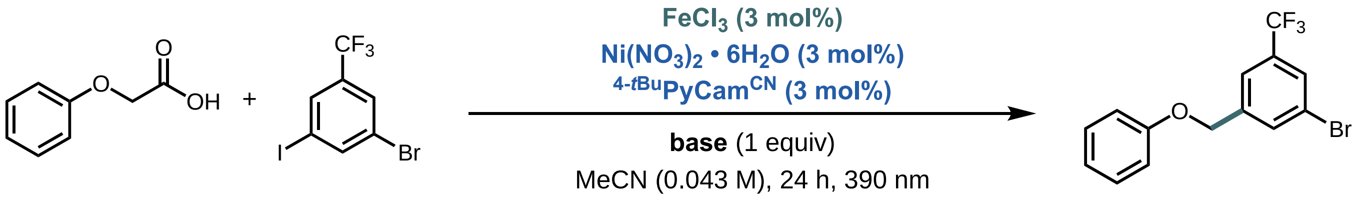

entry	base	product (%)
1	sodium hydroxide	9
2	potassium *tert*-butoxide	2
3	potassium phosphate dibasic	1
4	sodium bicarbonate	1
5	cesium carbonate	18
6	N,N-diisopropylethylamine	55
7	1,1,3,3-tetramethylguanidine	49
8	N,N-dimethylaminopyridine	10
9	triethylamine	52
10	2,6-lutidine	12
11	quinuclidine	53

[a] Reactions were set up following the general procedure for optimization in the decarboxylative arylation using 2-phenoxyacetic acid (0.100 mmol, 1.00 equiv) and 1-bromo-3-iodo-5-(trifluoromethyl)benzene (0.100 mmol, 1.00 equiv) in 2.30 mL of MeCN. [b] Yields were determined by ^{1}H NMR spectroscopy using dibromomethane as an internal standard.

It is noted that alkyl amine bases (iPr$_2$NEt, TEA, and quinuclidine) were comparable bases. The bases were tested on different substrates and iPr$_2$NEt was chosen as optimal for achieving desired reactivity.

D. Base Equivalents

Reaction yield was examined with different base equivalents according to the general procedure for optimization in the decarboxylative arylation (see section IIIA2).

Table S6. Summary of the optimization of base equivalents[a,b]

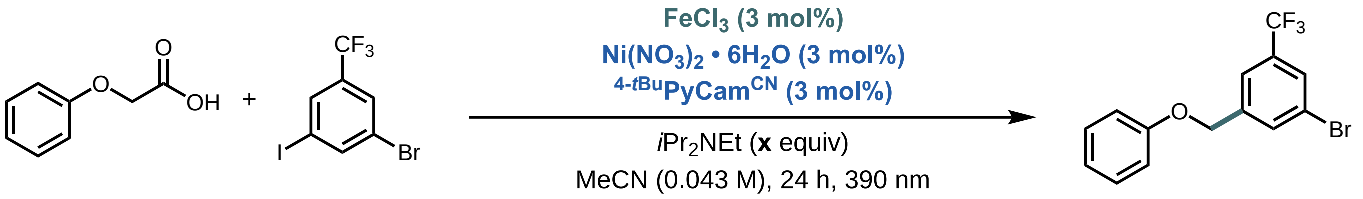

entry	equiv	product (%)
1	0.1	3
2	0.5	23
3	1	58
4	1.5	57
5	2	52
6	2.5	42
7	3	31
8	3.5	26
9	4	22
10	4.5	18
11	5	16

[a] Reactions were set up following the general procedure for optimization in the decarboxylative arylation using 2-phenoxyacetic acid (0.100 mmol, 1.00 equiv) and 1-bromo-3-iodo-5-(trifluoromethyl)benzene (0.100 mmol, 1.00 equiv) in 2.30 mL of MeCN. [b] Yields were determined by ^{1}H NMR spectroscopy using dibromomethane as an internal standard.

The yield of the reactions was comparable when using 1-2 equivalents of *i*Pr$_2$NEt however the use of 2 equivalents of base allowed for a more general scope.

E. Additives

Reaction yield was examined with different additives according to the general procedure for optimization in the decarboxylative arylation (see section IIIA2).

Table S7. Summary of the optimization of additives[a,b]

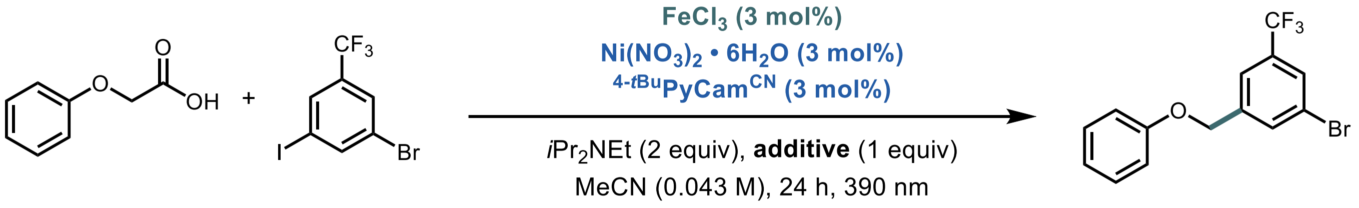

entry	additive	product (%)
1	phthalimide	66
2	succinimide	55
3	TBAI	63
4	TBABr	67
5	TBACl	62
6	phthalimide + TBAI	70
7	phthalimide + TBABr	73
8	phthalimide + TBACl	61
9	succinimide + TBAI	64
10	succinimide + TBABr	69
11	succinimide + TBACl	68

[a] Reactions were set up following the general procedure for optimization in the decarboxylative arylation using 2-phenoxyacetic acid (0.100 mmol, 1.00 equiv) and 1-bromo-3-iodo-5-(trifluoromethyl)benzene (0.100 mmol, 1.00 equiv) in 2.30 mL of MeCN. [b] Yields were determined by ^{1}H NMR using dibromomethane as an internal standard.

Several additives and additive combinations were observed to improve the yield of the desired product. When analyzing different substrates, the addition of TBAI was found to be necessary for obtaining high yields while the addition of phthalimide allowed for enhanced yields for select substrates.

F. Additive Ratios

Reaction yield was examined with different additive ratios according to the general procedure for optimization in the decarboxylative arylation (see section IIIA2).

Table S8. Summary of the optimization of additive ratios[a,b]

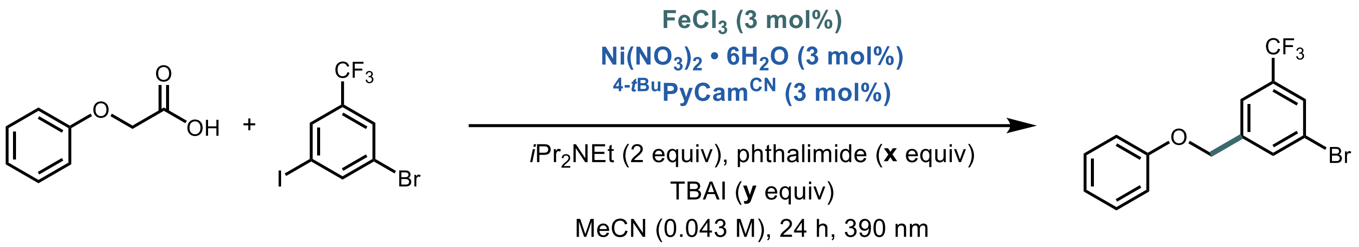

entry	phthalimide (x)	TBAI (y)	product (%)
1	1	1	80
2	0.5	1	81
3	0.25	1	74
4	0	1	50
5	1	0	68
6	1	0.25	68
7	1	0.5	72

[a] Reactions were set up following the general procedure for optimization in the decarboxylative arylation using 2-phenoxyacetic acid (0.100 mmol, 1.00 equiv) and 1-bromo-3-iodo-5-(trifluoromethyl)benzene (0.100 mmol, 1.00 equiv) in 2.30 mL of MeCN. [b] Yields were determined by ^1H NMR spectroscopy using dibromomethane as an internal standard.

The yield of the reaction was comparable when using phth:TBAI in a 1:1 or 0.5:1 ratio. Given these results, lower loading of phthalimide was still enough to achieve high yields.

G. Catalyst Loading

Reaction yield was examined with different catalyst loadings according to the general procedure for optimization in the decarboxylative arylation (see section IIIA2).

Table S9. Summary of the optimization of catalyst loading[a,b]

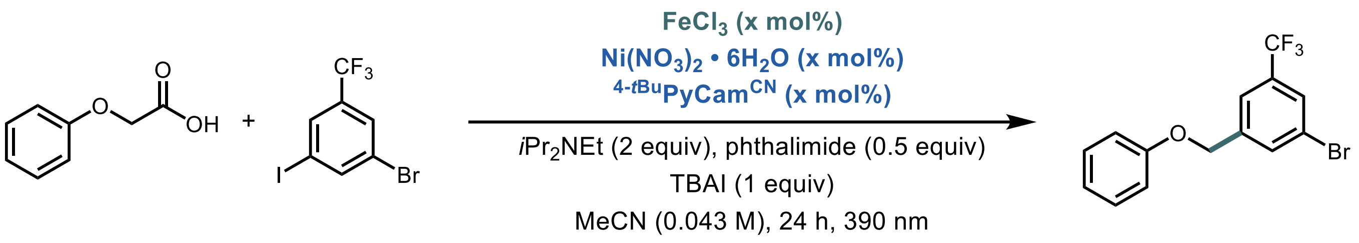

entry	Fe (x mol%)	Ni (x mol%)	product (%)
1	0.5	0.5	66
2	1	1	64
3	2	2	76
4	3	3	79
5	5	5	71
6	10	10	74
7	15	15	64
8	20	20	68

[a] Reactions were set up following the general procedure for optimization in the decarboxylative arylation using 2-phenoxyacetic acid (0.100 mmol, 1.00 equiv) and 1-bromo-3-iodo-5-(trifluoromethyl)benzene (0.100 mmol, 1.00 equiv) in 2.30 mL of MeCN. [b] Yields were determined by ^1H NMR spectroscopy using dibromomethane as an internal standard.

H. Catalyst Ratios

Reaction yield was examined with different catalyst ratios according to the general procedure for optimization in the decarboxylative arylation (see section IIIA2).

Table S10. Summary of the optimization of catalyst ratios[a,b]

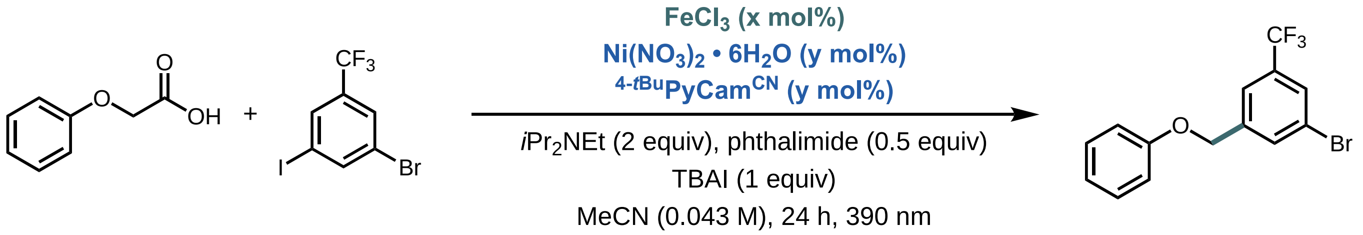

entry	Fe:Ni (x:y mol%)	product (%)
1	3:1	63
2	3:3	80
3	3:5	84
4	3:7	72
5	3:9	69
6	3:12	68
7	1:3	71
8	5:3	71
9	7:3	67
10	9:3	61
11	12:3	59

[a] Reactions were set up following the general procedure for optimization in the decarboxylative arylation using 2-phenoxyacetic acid (0.100 mmol, 1.00 equiv) and 1-bromo-3-iodo-5-(trifluoromethyl)benzene (0.100 mmol, 1.00 equiv) in 2.30 mL of MeCN. [b] Yields were determined by ^{1}H NMR spectroscopy using dibromomethane as an internal standard.

Upon examining different ratios of the Fe and the Ni catalyst, it was observed that Fe:Ni in a 3:3 or 3:5 ratio gave comparable yields. The catalyst ratios were tested on different substrates and a 3:3 ratio was chosen as optimal for achieving desired reactivity.

I. Wavelength

1. Using 1-bromo-3-iodo-5-(trifluoromethyl)benzene

Reaction yield was examined upon irradiation at different wavelengths according to the general procedure for optimization in the decarboxylative arylation (see section IIIA2).

Table S11. Summary of the optimization of wavelength[a,b]

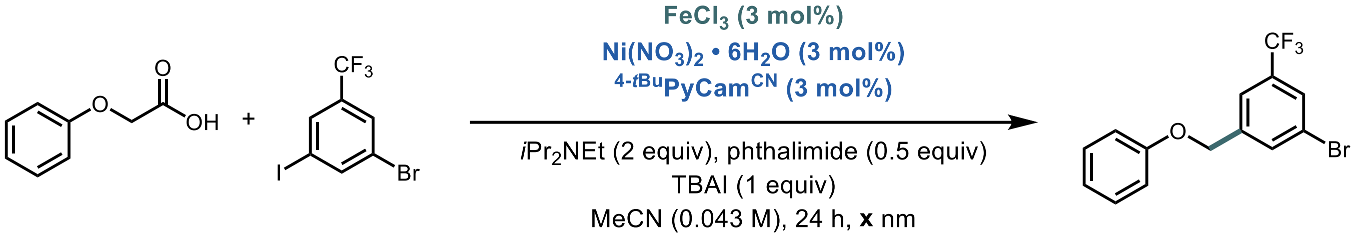

entry	wavelength (nm)	product (%)
1	370	42
2	390	72
3	427	66
4	440	62
5	456	46
6	525	0

[a] Reactions were set up following the general procedure for optimization in the decarboxylative arylation using 2-phenoxyacetic acid (0.100 mmol, 1.00 equiv) and 1-bromo-3-iodo-5-(trifluoromethyl)benzene (0.100 mmol, 1.00 equiv) in 2.30 mL of MeCN. [b] Yields were determined by ^{1}H NMR spectroscopy using dibromomethane as an internal standard.

This data indicates that 390 nm is optimal; however, the yield is also appreciable even in the visible region (427 nm and 440 nm). To track the byproduct distribution effectively, a detailed study using 1-bromo-3-iodo-5-(trifluoromethyl)benzene was performed and the analysis is shown below.

2. Using 1-chloro-3-iodo-5-(trifluoromethyl)benzene

i. Standard reaction

Reaction yield was examined upon irradiation at different wavelengths according to the general procedure for optimization in the decarboxylative arylation (see section IIIA3).

Table S12. Summary of yields irradiating the standard reaction at different wavelengths under standard reaction conditions[a,b,c]

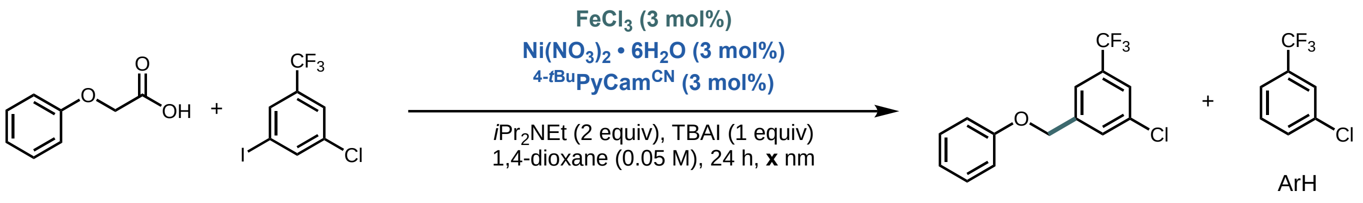

entry	wavelength (nm)	product (%)	ArH (%)	ArI (%)	acid (%)
1	370	64	22	0	0
2	390	85	6	0	0
3	427	74	7	0	15
4	440	70	4	22	28
5	456	23	4	42	50
6	525	0	3	98	68

[a] Reactions were set up following the general procedure for optimization in the decarboxylative arylation using 2-phenoxyacetic acid (0.130 mmol, 1.30 equiv) and 1-chloro-3-iodo-5-(trifluoromethyl)benzene in 2.00 mL of 1,4-dioxane. [b] Product yields were determined by ^{1}H NMR spectroscopy using dibromomethane as an internal standard. [c] Hydrodehalogenation yields were obtained by GC/MS using 1,3,5-trimethoxybenzene as an internal standard.

ii. Reaction without Ni catalyst

Reaction yield in the absence of Ni catalyst was examined upon irradiation at different wavelengths according to the general procedure for optimization in the decarboxylative arylation (see section IIIA3).

Table S13. Summary of yields irradiating the standard reaction at different wavelengths under standard reaction conditions without Ni catalyst[a,b,c]

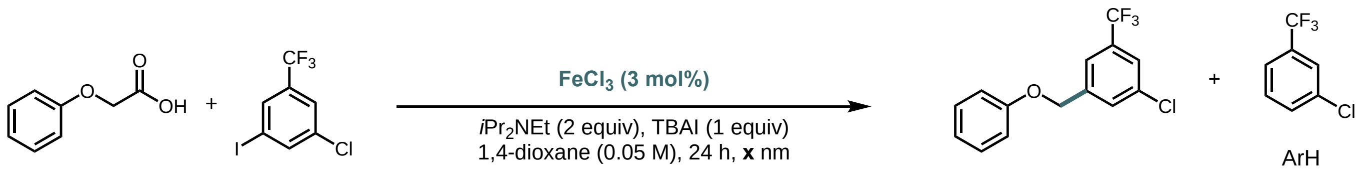

entry	wavelength (nm)	product (%)	ArH (%)	ArI (%)	acid (%)
1	370	0	74	0	20
2	390	0	45	28	48
3	427	0	4	80	77
4	440	0	1	80	80
5	456	0	trace	94	90
6	525	0	0	92	97

[a] Reactions were set up following the general procedure for optimization in the decarboxylative arylation using 2-phenoxyacetic acid (0.130 mmol, 1.30 equiv) and 1-chloro-3-iodo-5-(trifluoromethyl)benzene in 2.00 mL of 1,4-dioxane. [b] Product yields were determined by ^{1}H NMR spectroscopy using dibromomethane as an internal standard. [c] Hydrodehalogenation yields were obtained by GC/MS using 1,3,5-trimethoxybenzene as an internal standard.

iii. *Reaction without Fe catalyst*

Reaction yield in the absence of Fe catalyst was examined upon irradiation at different wavelengths according to the general procedure for optimization in the decarboxylative arylation (see section IIIA3).

Table S14. Summary of yields irradiating the standard reaction at different wavelengths under standard reaction conditions without Fe catalyst[a,b,c]

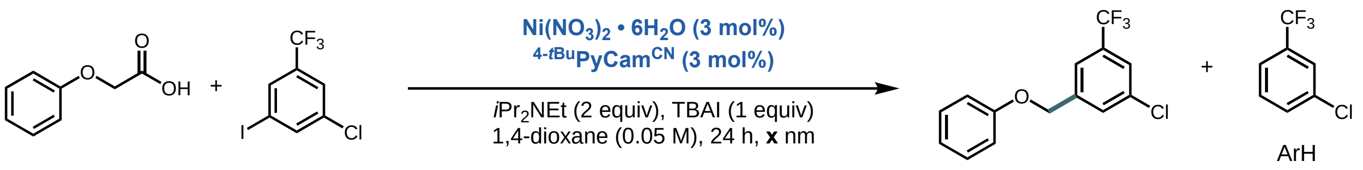

entry	wavelength (nm)	product (%)	ArH (%)	ArI (%)	acid (%)
1	370	9	71	0	21
2	390	11	52	24	36
3	427	0	16	72	57
4	440	0	9	80	82
5	456	0	5	80	74
6	525	0	0	94	76

[a] Reactions were set up following the general procedure for optimization in the decarboxylative arylation using 2-phenoxyacetic acid (0.130 mmol, 1.30 equiv) and 1-chloro-3-iodo-5-(trifluoromethyl)benzene in 2.00 mL of 1,4-dioxane. [b] Product yields were determined by ^{1}H NMR spectroscopy using dibromomethane as an internal standard. [c] Hydrodehalogenation yields were obtained by GC/MS using 1,3,5-trimethoxybenzene as an internal standard.

iv. Reaction without catalyst (with ${}^{4\text{-}tBu}$PyCam^{CN})

Reaction yield in the absence of both catalysts (with *${}^{4\text{-}tBu}$PyCam^{CN}*) was examined upon irradiation at different wavelengths according to the general procedure for optimization in the decarboxylative arylation (see section IIIA3).

Table S15. Summary of yields irradiating the standard reaction at different wavelengths under standard reaction conditions without Fe or Ni catalyst[a,b,c]

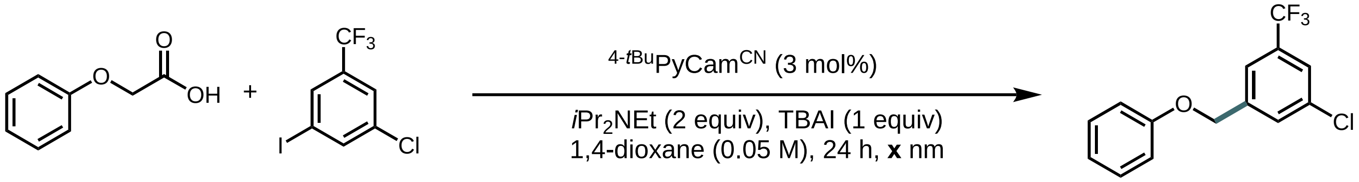

entry	wavelength (nm)	product (%)	ArH (%)	ArI (%)	acid (%)
1	370	0	100	0	0
2	390	0	82	20	45
3	427	0	6	50	97
4	440	0	9	70	96
5	456	0	5	52	100
6	525	0	0	72	100

[a] Reactions were set up following the general procedure for optimization in the decarboxylative arylation using 2-phenoxyacetic acid (0.130 mmol, 1.30 equiv) and 1-chloro-3-iodo-5-(trifluoromethyl)benzene in 2.00 mL of 1,4-dioxane. [b] Product yields were determined by ^{1}H NMR spectroscopy using dibromomethane as an internal standard. [c] Hydrodehalogenation yields were obtained by GC/MS using 1,3,5-trimethoxybenzene as an internal standard.

v. Reaction without catalyst

Reaction yield in the absence of catalyst was examined upon irradiation at different wavelengths according to the general procedure for optimization in the decarboxylative arylation (see section IIIA3).

Table S16. Summary of yields irradiating the standard reaction at different wavelengths under standard reaction conditions without Fe or Ni catalyst[a,b,c]

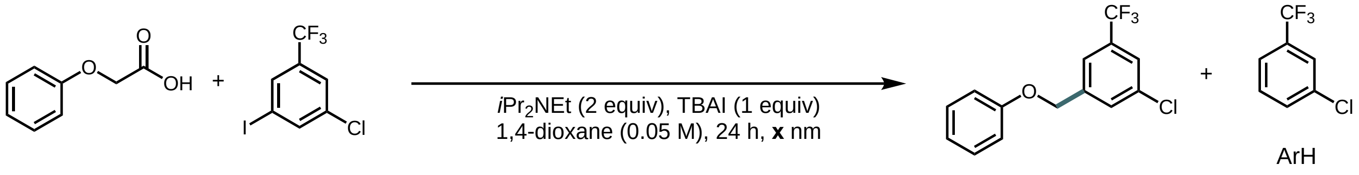

entry	wavelength (nm)	product (%)	ArH (%)	ArI (%)	acid (%)
1	370	0	89	0	44
2	390	0	45	36	75
3	427	0	5	80	99
4	440	0	5	82	97
5	456	0	5	82	93
6	525	0	trace	86	100

[a] Reactions were set up following the general procedure for optimization in the decarboxylative arylation using 2-phenoxyacetic acid (0.130 mmol, 1.30 equiv) and 1-chloro-3-iodo-5-(trifluoromethyl)benzene in 2.00 mL of 1,4-dioxane. [b] Product yields were determined by ^{1}H NMR spectroscopy using dibromomethane as an internal standard. [c] Hydrodehalogenation yields were obtained by GC/MS using 1,3,5-trimethoxybenzene as an internal standard.

J. Solvent with Additives

Reaction yield was examined in different solvents with additives according to the general procedure for optimization in the decarboxylative arylation (see section IIIA2).

Table S17. Summary of additive effects under different solvents[a,b]

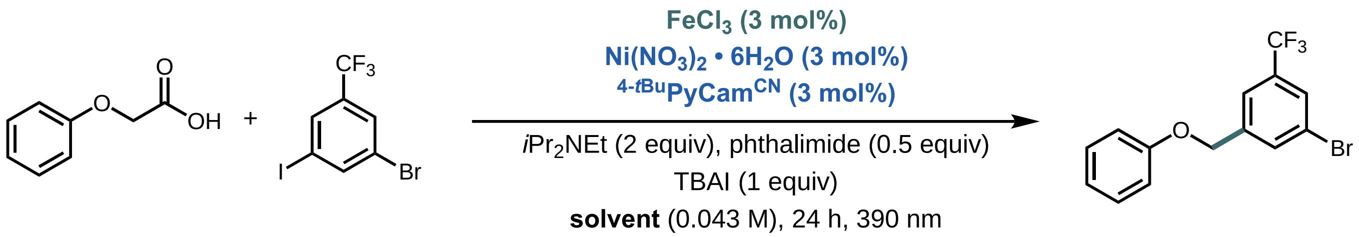

entry	solvent	product (%)
1	acetonitrile	81
2	ethyl alcohol	27
3	ethyl acetate	58
4	1,4-dioxane	75
5	dimethyl carbonate	84

[a] Reactions were set up following the general procedure for optimization in the decarboxylative arylation using 2-phenoxyacetic acid (0.100 mmol, 1.00 equiv) and 1-bromo-3-iodo-5-(trifluoromethyl)benzene (0.100 mmol, 1.00 equiv) in 2.30 mL of solvent. [b] Yields were determined by ^1H NMR spectroscopy using dibromomethane as an internal standard.

K. Controls

Reaction yield for control reactions were examined according to the general procedure for optimization in the decarboxylative arylation (see section IIIC). Table 1 in the manuscript includes control and optimization data. Shared below is a more extensive set.

Table S18. Summary of various control reactions under standard reaction conditions[a,b]

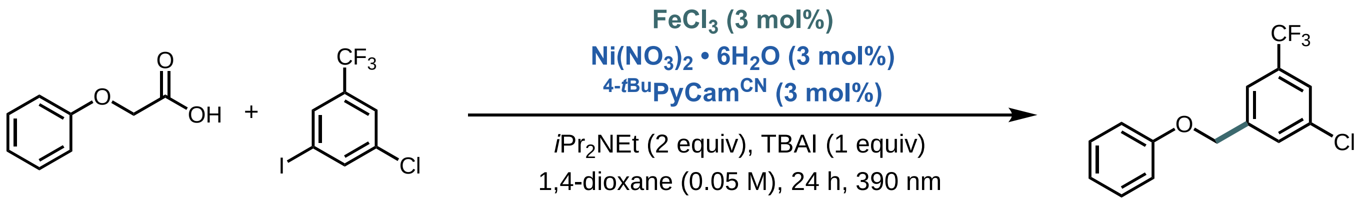

entry	deviation from above	product (%)
1	none	86
2	1.0 equiv of acid instead of 1.3	82
3	**L2** instead of $^{4\text{-}t\text{Bu}}$PyCam^{CN}	37
4	no Fe catalyst	19
5	no Ni catalyst	0
6	no light (rt)	0
7	no light (60 °C)	0
8	no ligand	0
9	no TBAI	73
10	with 0.5 equiv of phthalimide	90
11	Cs_2CO_3 instead of *i*Pr_2NEt	9
12	370 nm instead of 390 nm	66
13	427 nm instead of 390 nm	62
14	KI instead of TBAI	72
15	TBACl instead of TBAI	31
16	MeCN instead of 1,4-dioxane	67
17	DMC instead of 1,4-dioxane	75
18[c]	air instead of N_2	64

[a] Reactions were set up following the general procedure for aryl iodide scope in the decarboxylative arylation using 2-phenoxyacetic acid (0.650 mmol, 1.30 equiv) and 1-chloro-3-iodo-5-(trifluoromethyl)benzene (0.500 mmol, 1.00 equiv) in 10.0 mL of 1,4-dioxane. [b] Yields were determined by ^{1}H NMR spectroscopy using dibromomethane as an internal standard. [c] The reaction was set up on the benchtop without any sparging (no exclusion of O_2) and the vial was not sealed and left open to air.

Reaction yield for control reactions were examined according to the general procedure for optimization in the decarboxylative arylation (see section IIID).

The effect of the additives on the reactivity of unactivated acids was also tested as this could change with the different reaction conditions being used. These reactions were examined at a 0.1 mmol scale using 2,3-dihydro-1H-indene-2-carboxylic acid as the model carboxylic acid for the unactivated carboxylic acid.

Table S19. Summary of various control reactions for unactivated acids[a,b]

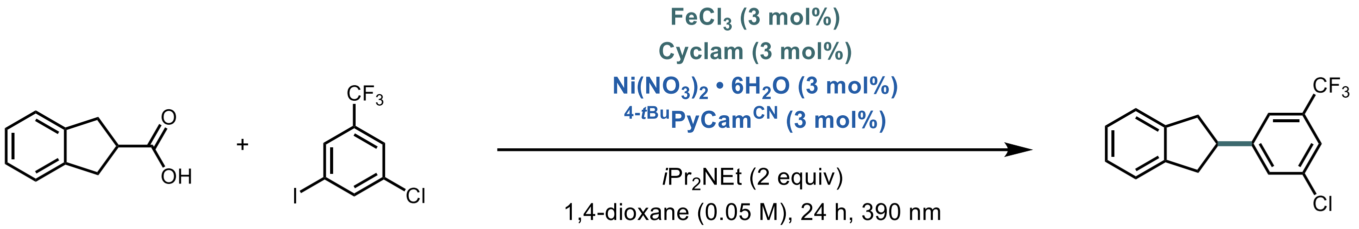

entry	deviation from above	product (%)
1	none	43
2	with 1 equiv of TBAI	73
3	with 0.5 equiv of phthalimide	71
4	with 1 equiv of TBAI, 0.5 equiv of phthalimide	66
5	with 1 equiv TBAI, no *i*Pr_2NEt	1
6	with 1 equiv TBAI, no *i*Pr_2NEt, 2 equiv Cyclam	0

[a] Reactions were setup following the general procedure for unactivated carboxylic acid scope in the decarboxylative arylation using 2,3-dihydro-1H-indene-2-carboxylic acid (0.130 mmol, 1.30 equiv) and 1-chloro-3-iodo-5-(trifluoromethyl)benzene (0.100 mmol, 1.00 equiv) in 2.00 mL of 1,4-dioxane. [b] Yields were determined by ^{19}F NMR spectroscopy using fluorobenzene as an internal standard.

V. Reaction Robustness

A. Water Tolerance

Reaction yield was examined upon addition of exogenous water to reactions prepared according to the general procedure for optimization in the decarboxylative arylation (see section IIIA2).

Table S20. Summary of yields upon the addition of DI water to the reaction[a,b]

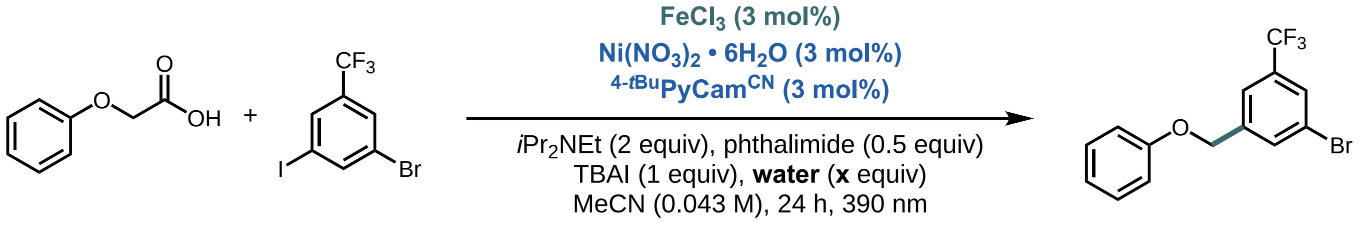

entry	water (equiv)	product(%)
1	0	76
2	0.1	76
3	0.2	76
4	0.5	78
5	1	74
6	2	74
7	3	66
8	4	69
9	5	62
10	10	57

[a] Reactions were set up following the general procedure for optimization in the decarboxylative arylation using 2-phenoxyacetic acid (0.100 mmol, 1.00 equiv) and 1-bromo-3-iodo-5-(trifluoromethyl)benzene (0.100 mmol, 1.00 equiv) in 2.30 mL of MeCN. [b] Yields were determined by ^{1}H NMR spectroscopy using dibromomethane as an internal standard.

B. Air Tolerance

Reaction yield was examined upon the addition of air to the reactions prepared according to the general procedure for optimization in the decarboxylative arylation (see section IIIA2). Air was introduced into the system. For entry 7, the reaction was under air.

Table S21. Summary of reaction[a,b]

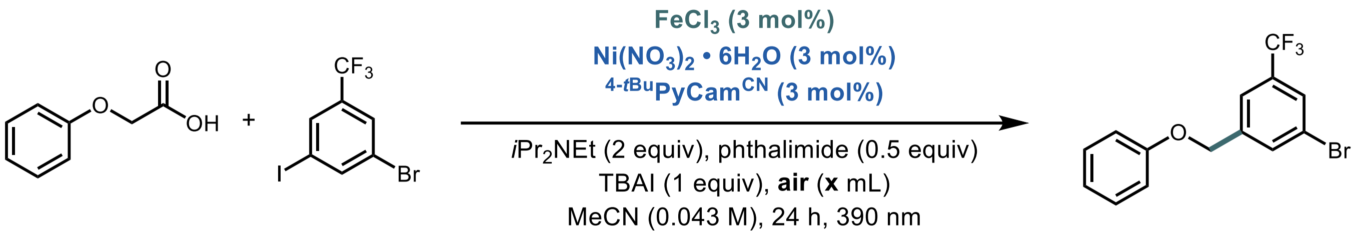

entry	air (mL)	product (%)
1	0	81
2	0.2	76
3	0.4	76
4	0.6	73
5	0.8	69
6	1	71
7[c]	air	39

[a] Reactions were set up following the general procedure for optimization in the decarboxylative arylation using 2-phenoxyacetic acid (0.100 mmol, 1.00 equiv) and 1-bromo-3-iodo-5-(trifluoromethyl)benzene (0.100 mmol, 1.00 equiv) in 2.30 mL of MeCN. Air was introduced into the system. [b] Yields were determined by ^1H NMR spectroscopy using dibromomethane as an internal standard. [c] The reaction was on the benchtop with no exclusion of O_2 and left open to air.

C. Comparison with Ir and Ni Metallaphotoredox Catalysis

Comparative studies were conducted with the Ir/Ni decarboxylative arylation protocol established by MacMillan and Doyle in 2014.[7] Prior to testing the reactions, the reported procedure was successfully reproduced using the model substrates employed in their work.

Table S22. Comparison of the referenced work[7] and the current work using 2-phenoxyacetic acid[a,b]

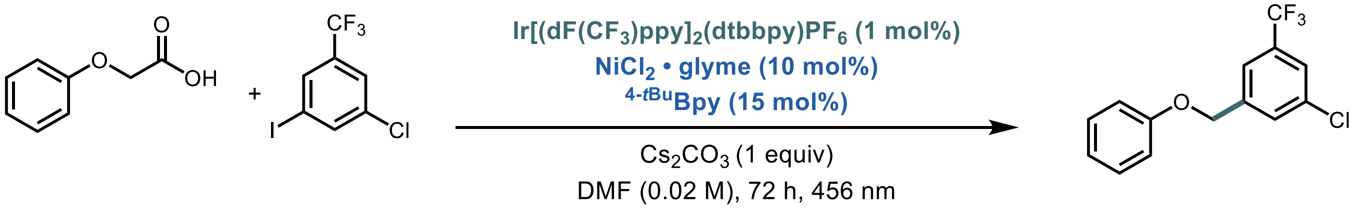

entry	deviation from above	product (%)
1[c]	our method	86
2	none	12
3	$Ni(COD)_2$ instead of $NiCl_2$ • glyme	10
4	${}^{4\text{-}t\text{Bu}}$PyCam^{CN} instead of ${}^{4\text{-}t\text{Bu}}$Bpy	0

[a] Reactions were set up following literature precedent procedure[5] using 2-phenoxacetic acid (0.130 mmol, 1.30 equiv) and 1-chloro-3-iodo-5-(trifluoromethyl)benzene (0.100 mmol, 1.00 equiv) in 5.00 mL of N,N-dimethylformamide in the glovebox (under N_2 atmosphere). [b] Yields were determined by ^{1}H NMR spectroscopy using dibromomethane as an internal standard. [c] For our Fe/Ni metallaphotoredox method, the reactions were set up according to the optimized conditions (see section IIIA3) using 2-phenoxacetic acid (0.130 mmol, 1.30 equiv) and 1-chloro-3-iodo-5-(trifluoromethyl)benzene (0.100 mmol, 1.00 equiv) in 2.00 mL of 1,4-dioxane.

[7] Zuo, Z.; Ahneman, D. T.; Chu, L.; Terrett, J. A.; Doyle, A. G.; MacMillan, D. W. *Science.* **2014**, *345*, 437-440.

Table S23. Comparative experiment between the referenced work[7] and the current work using 1-methylpiperidine-4-carboxylic acid[a,b]

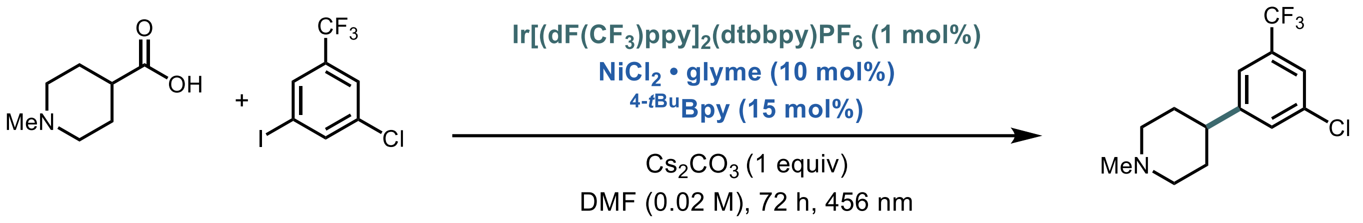

entry	deviation from above	product (%)
1[c]	our method	40
2	none	trace
3	$Ni(COD)_2$ instead of $NiCl_2$ • glyme	0
4	$^{4\text{-}t\text{Bu}}$PyCam^{CN} instead of $^{4\text{-}t\text{Bu}}$Bpy	0

[a] Reactions were set up following literature precedent procedure[5] using 1-methylpiperidine-4-carboxylic acid (0.130 mmol, 1.30 equiv) and 1-chloro-3-iodo-5-(trifluoromethyl)benzene (0.100 mmol, 1.00 equiv) in 5.00 mL of N,N-dimethylformamide in the glovebox (under N_2 atmosphere). [b] Yields were determined by ^1H NMR spectroscopy using dibromomethane as an internal standard. [c] For our Fe/Ni metallaphotoredox method, the reactions were set up according to the optimized conditions (see section IIID) using 1-methylpiperidine-4-carboxylic acid (0.130 mmol, 1.30 equiv) and 1-chloro-3-iodo-5-(trifluoromethyl)benzene (0.100 mmol, 1.00 equiv) with phthalimide (0.050 mmol, 0.500 equiv) and $ZnCl_2$ (0.0300 mmol, 0.300 equiv) in 2.00 mL of 1,4-dioxane .

VI. UV-vis Spectroscopy Studies

To gain insight into the ligation and absorbance of various Ni and Fe complexes relevant to the optimized conditions, UV-vis spectroscopy studies were performed. The spectroscopic signatures of the catalyst, ligands, and additives were examined independently and in combination.

A. UV-vis Spectra of Ligands and Additives

1. $^{4\text{-}t\text{Bu}}$PyCam^{CN}

To a 1-dram vial, $^{4\text{-}t\text{Bu}}$PyCam^{CN} (1.01 mg, 0.005000 mmol) was added and diluted with 1.00 mL of MeCN. A 10.0 µL aliquot of the solution was taken from the mixture and diluted with MeCN to 2.00 mL in a quartz cuvette. The sample was then subjected to UV-vis analysis. The UV-vis spectrum of $^{4\text{-}t\text{Bu}}$PyCam^{CN} exhibited strong absorption bands at 240 nm and 281 nm (Figure S3).

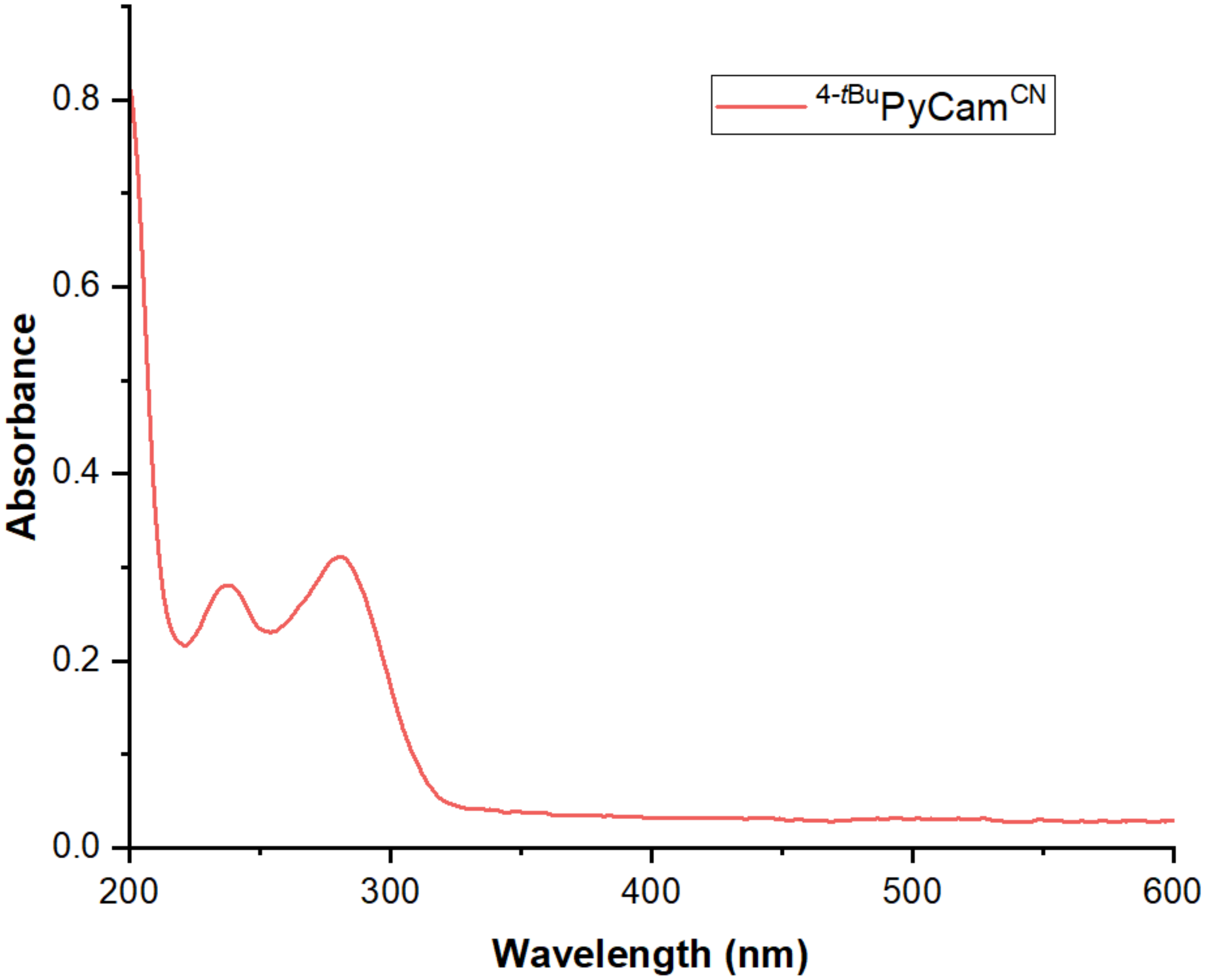

Figure S3. UV-vis spectra for $^{4\text{-}t\text{Bu}}$PyCam^{CN}.

2. 1,4,8,11-Tetraazacyclotetradecane (Cyclam)

To a 1-dram vial, cyclam (1.00 mg, 0.005000 mmol) was added and diluted with 2.00 mL of MeCN. Subsequently, the mixture was stirred at 60 °C for 5 min to fully dissolve cyclam in MeCN. A of 20.0 μL aliquot of the solution was taken from the mixture and diluted with MeCN to 2.00 mL in a quartz cuvette. The sample was then subjected to UV-vis analysis. The UV-vis spectrum of cyclam did not exhibit any significant absorption between 250-600 nm (Figure S4).

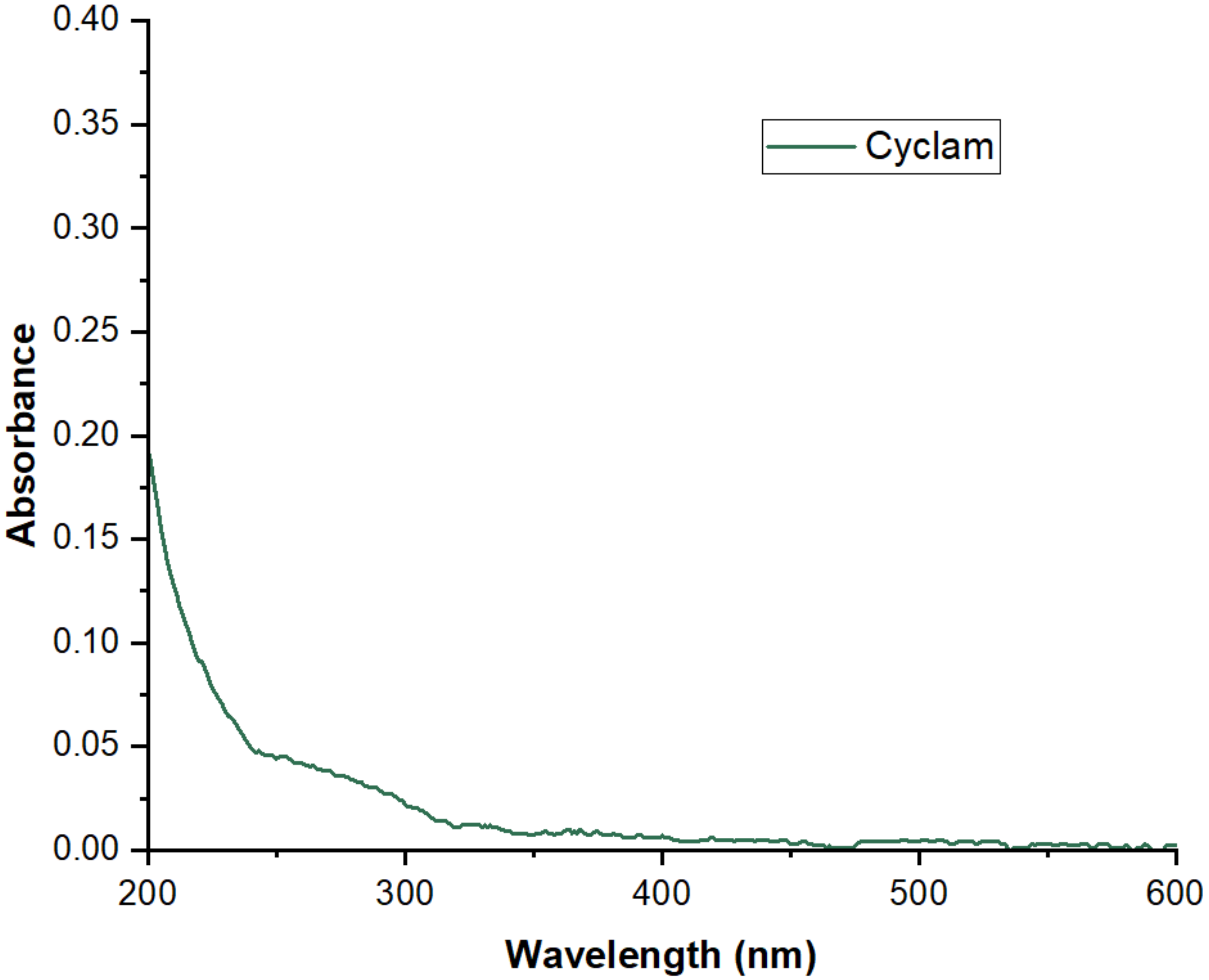

Figure S4. UV-vis spectra for cyclam.

3. TBAI

To a 1-dram vial, TBAI (1.84 mg, 0.00500 mmol) was added and diluted with 1.00 mL of MeCN. A 10.0 μL aliquot of the solution was taken from the mixture and diluted with MeCN to 2.00 mL in a quartz cuvette. The sample was then subjected to UV-vis analysis. The UV-vis spectrum of TBAI exhibited strong absorption band at 207 nm and 246 nm (Figure S5).

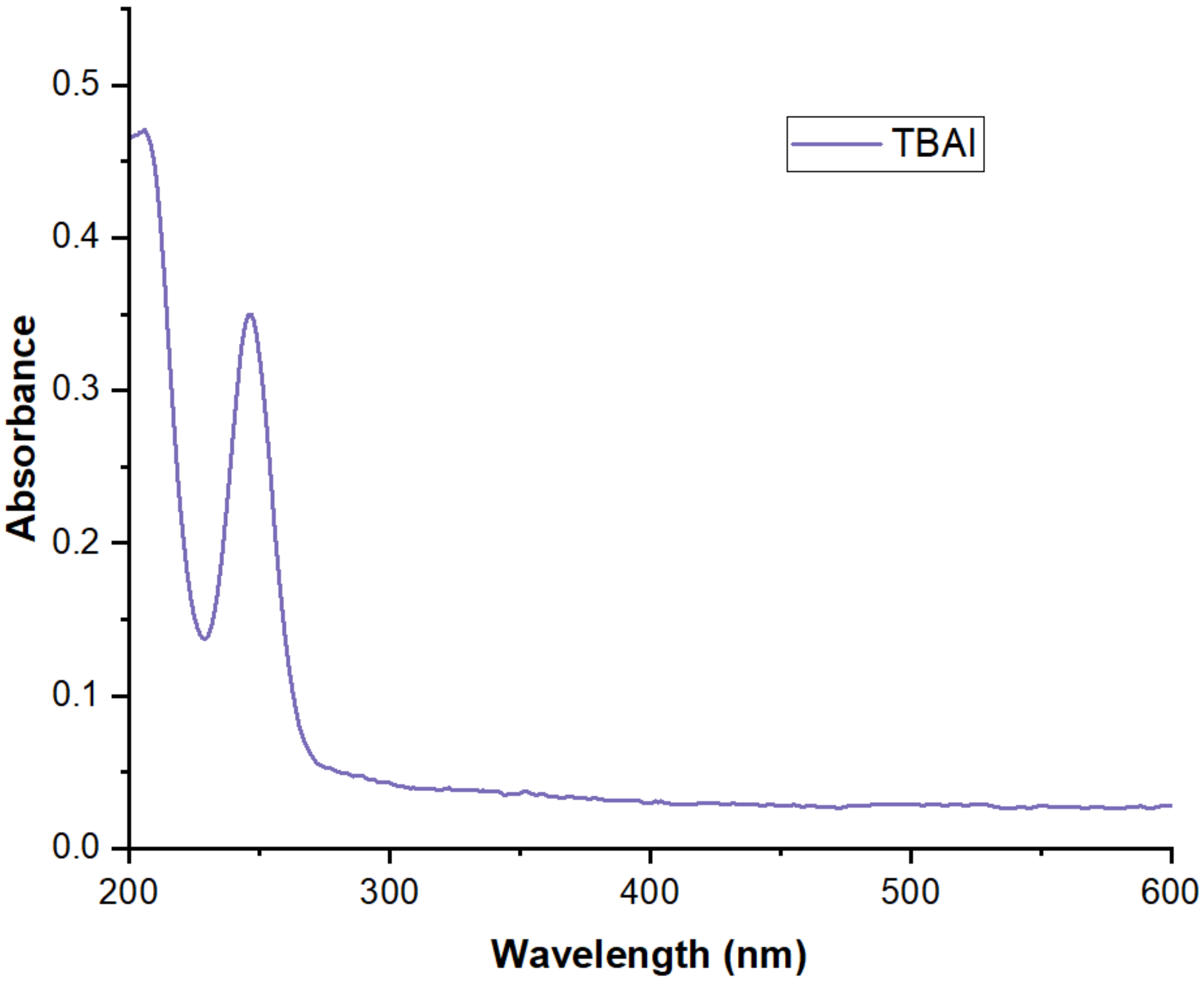

Figure S5. UV-vis spectra for TBAI.

4. Phthalimide

To a 1-dram vial, phthalimide (0.74 mg, 0.00500 mmol) was added and diluted with 1.00 mL of MeCN. Subsequently, the mixture was sonicated for 5 min to dissolve the phthalimide. A 10.0 μL aliquot of the solution was taken from the mixture and diluted with MeCN to 2.00 mL in a quartz cuvette. The sample was then subjected to UV-vis analysis. The UV-vis spectrum of phthalimide exhibited a strong absorption bands at 214 nm, a minor absorption band at 228 nm, and a broad absorption band at 294 nm (Figure S6).

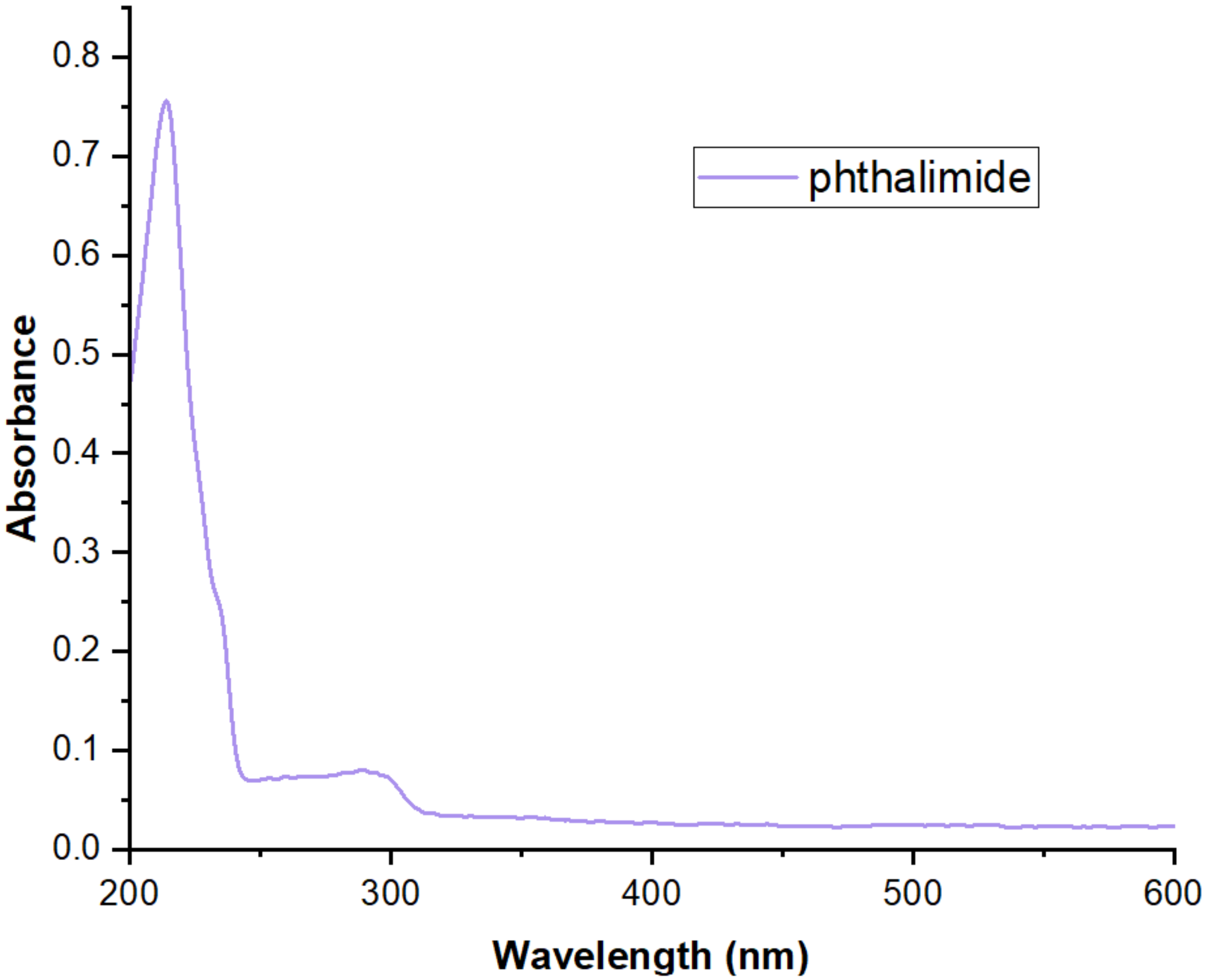

Figure S6. UV-vis spectra for phthalimide.

B. UV-vis Spectra of Ni Complexes

1. $Ni(NO_3)_2{\cdot}6H_2O$

To a 1-dram vial, $Ni(NO_3)_2{\cdot}6H_2O$ (1.45 mg, 0.00500 mmol) was added and diluted with 1.00 mL of MeCN. A 10.0 μL aliquot of the solution was taken from the mixture and diluted with MeCN to 2.00 mL in a quartz cuvette. The sample was then subjected to UV-vis analysis. The UV-vis spectrum of $Ni(NO_3)_2{\cdot}6H_2O$ exhibited a weak absorption band at 252 nm (Figure S7).

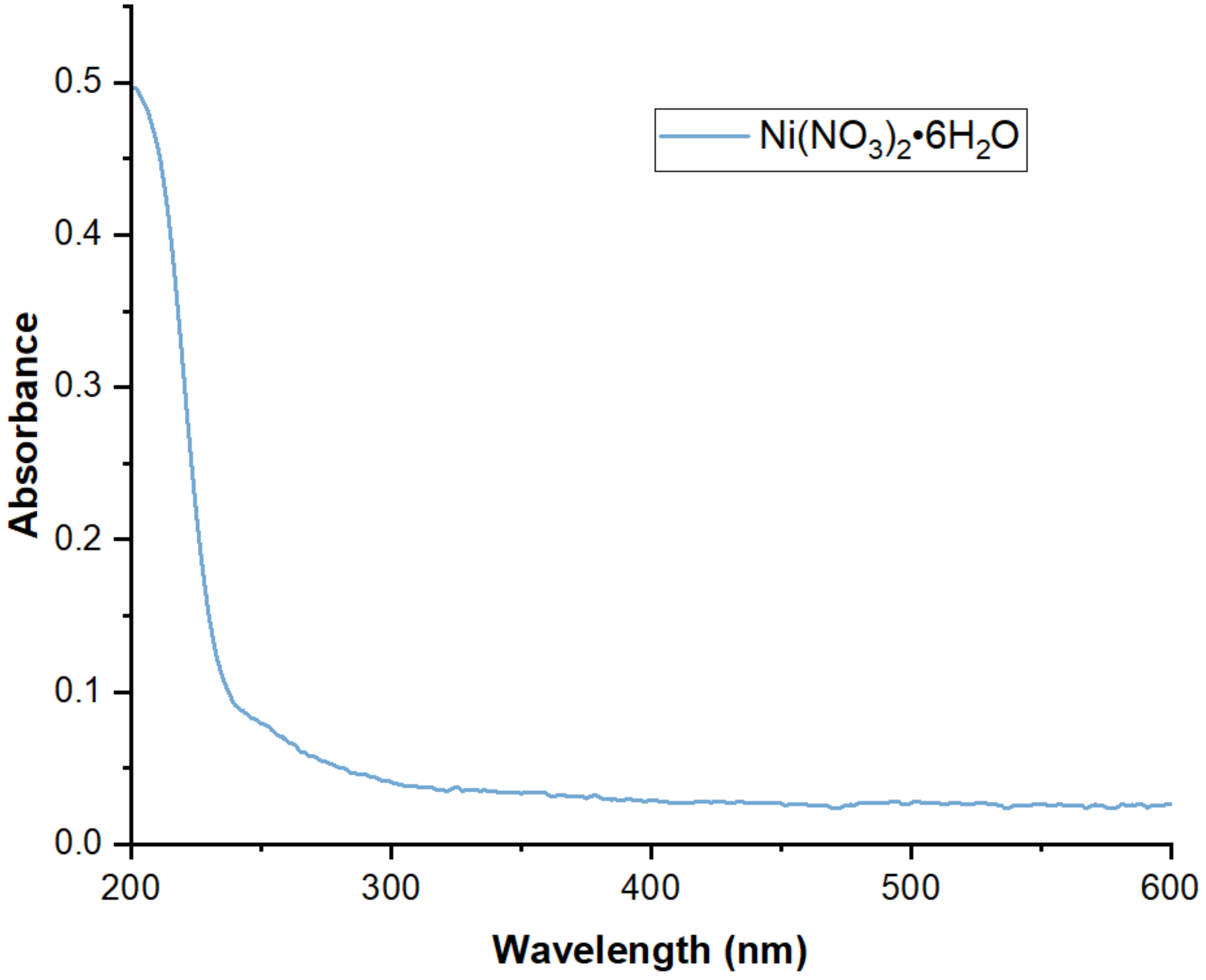

Figure S7. UV-vis spectra for $Ni(NO_3)_2{\cdot}6H_2O$ salt.

2. $Ni(NO_3)_2$•$6H_2O$, $^{4\text{-}tBu}$PyCam^{CN}

To a 1-dram vial, $Ni(NO_3)_2$•$6H_2O$ (1.45 mg, 0.00500 mmol, 1.00 equiv) and $^{4\text{-}tBu}$PyCam^{CN} (1.01 mg, 0.00500 mmol, 1.00 equiv) were added and diluted with 1.00 mL of MeCN. Subsequently, the mixture was stirred for 30 min at 60 °C. A 10.0 μL aliquot of the solution was taken from the mixture and diluted with MeCN to 2.00 mL in a quartz cuvette. The sample was then subjected to UV-vis analysis. The UV-vis spectrum of the $Ni(NO_3)_2$•$6H_2O$ and $^{4\text{-}tBu}$PyCam^{CN} mixture exhibited strong absorption bands at 204 nm at 280 nm (Figure S8).

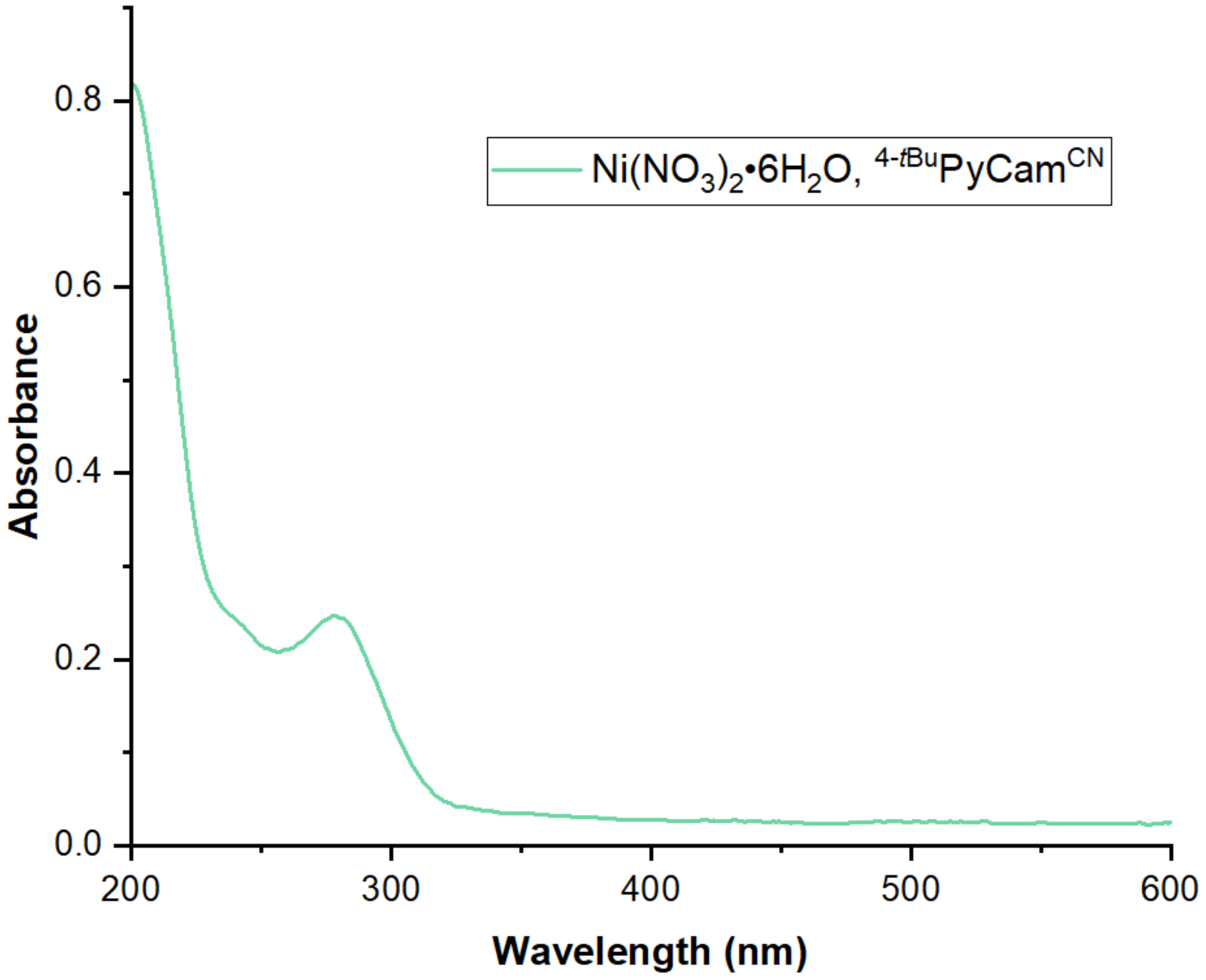

Figure S8. UV-vis spectra for $Ni(NO_3)_2$•$6H_2O$ with $^{4\text{-}tBu}$PyCam^{CN}.

The disappearance of peak at 240 nm corresponding to the $^{4\text{-}tBu}$PyCam^{CN}, suggests the formation of $Ni(NO_3)_2$•$6H_2O$,$^{4\text{-}tBu}$PyCam^{CN} complex.

3. Combined Spectra for $Ni(NO_3)_2 \cdot 6H_2O$

i. *$Ni(NO_3)_2 \cdot 6H_2O$, $^{4\text{-}tBu}$PyCam^{CN}*

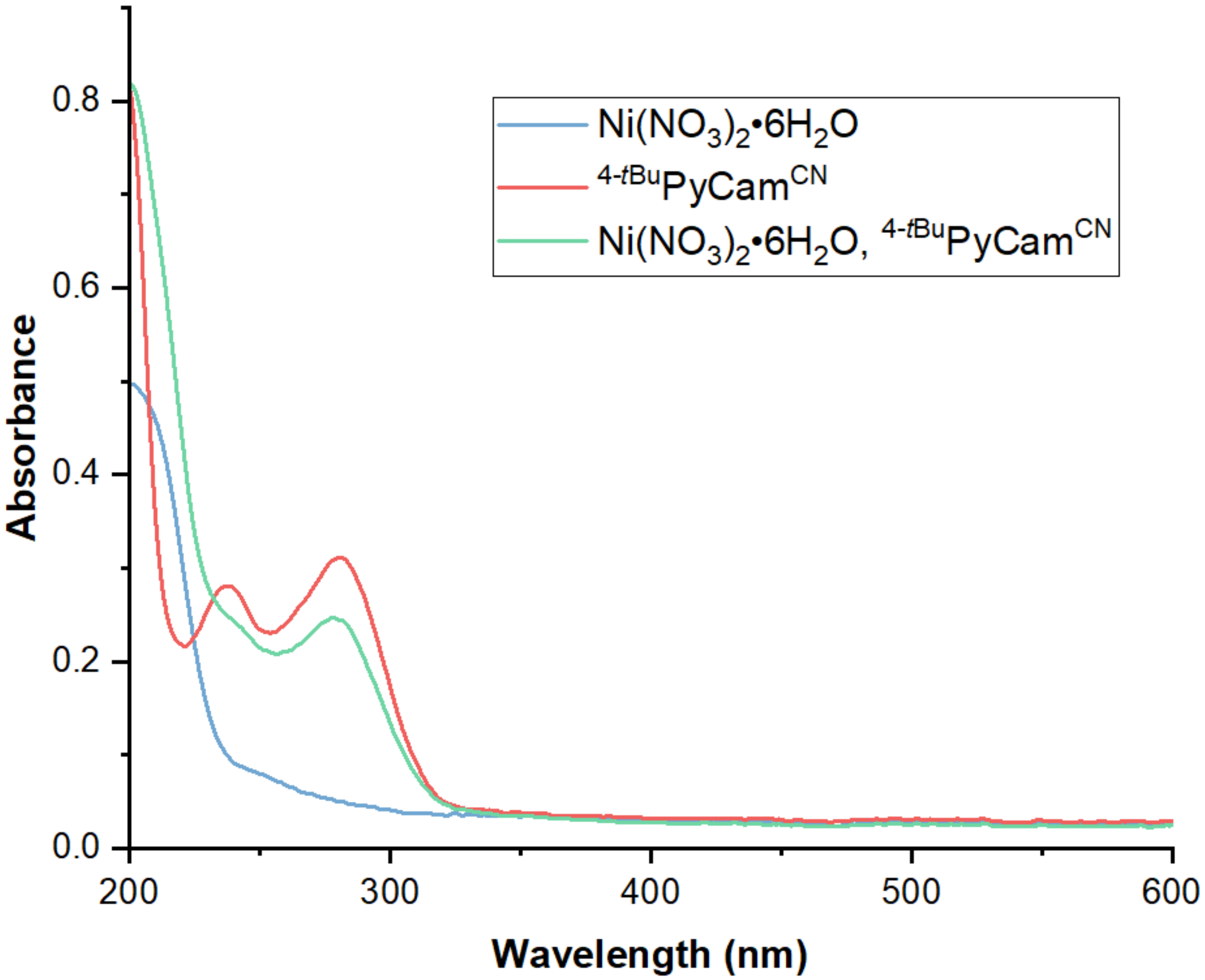

Figure S9. UV-vis spectra for $Ni(NO_3)_2 \cdot 6H_2O$ with $^{4\text{-}tBu}$PyCam^{CN}. UV-vis spectra of $Ni(NO_3)_2 \cdot 6H_2O$ solution (blue), $^{4\text{-}tBu}$PyCam^{CN} solution (pink), and $Ni(NO_3)_2 \cdot 6H_2O$ with $^{4\text{-}tBu}$PyCam^{CN} solution (green).

ii. $Ni(NO_3)_2$•$6H_2O$, $^{4\text{-}tBu}$PyCam^{CN} + phthalimide

To a 1-dram vial, $Ni(NO_3)_2$•$6H_2O$ (1.45 mg, 0.00500 mmol, 1.00 equiv) and $^{4\text{-}tBu}$PyCam^{CN} (1.01 mg, 0.00500 mmol, 1.00 equiv) were added and diluted with 1.00 mL of MeCN. Subsequently, the mixture was stirred for 30 min at 60 °C. To the mixture, phthalimide (0.74 mg, 0.00500 mmol, 1.00 equiv) was added and the solution was stirred for another 30 min at rt. A 10.0 µL aliquot of the solution was taken from the mixture and diluted with MeCN to 2.00 mL in a quartz cuvette. The sample was then subjected to UV-vis analysis. The UV-Vis spectrum of the $Ni(NO_3)_2$•$6H_2O$, $^{4\text{-}tBu}$PyCam^{CN}, and phthalimide mixture exhibited a strong absorption band at 210 nm, a minor absorption band at 236 nm, and a broad absorption band at 282 nm (Figure S10).

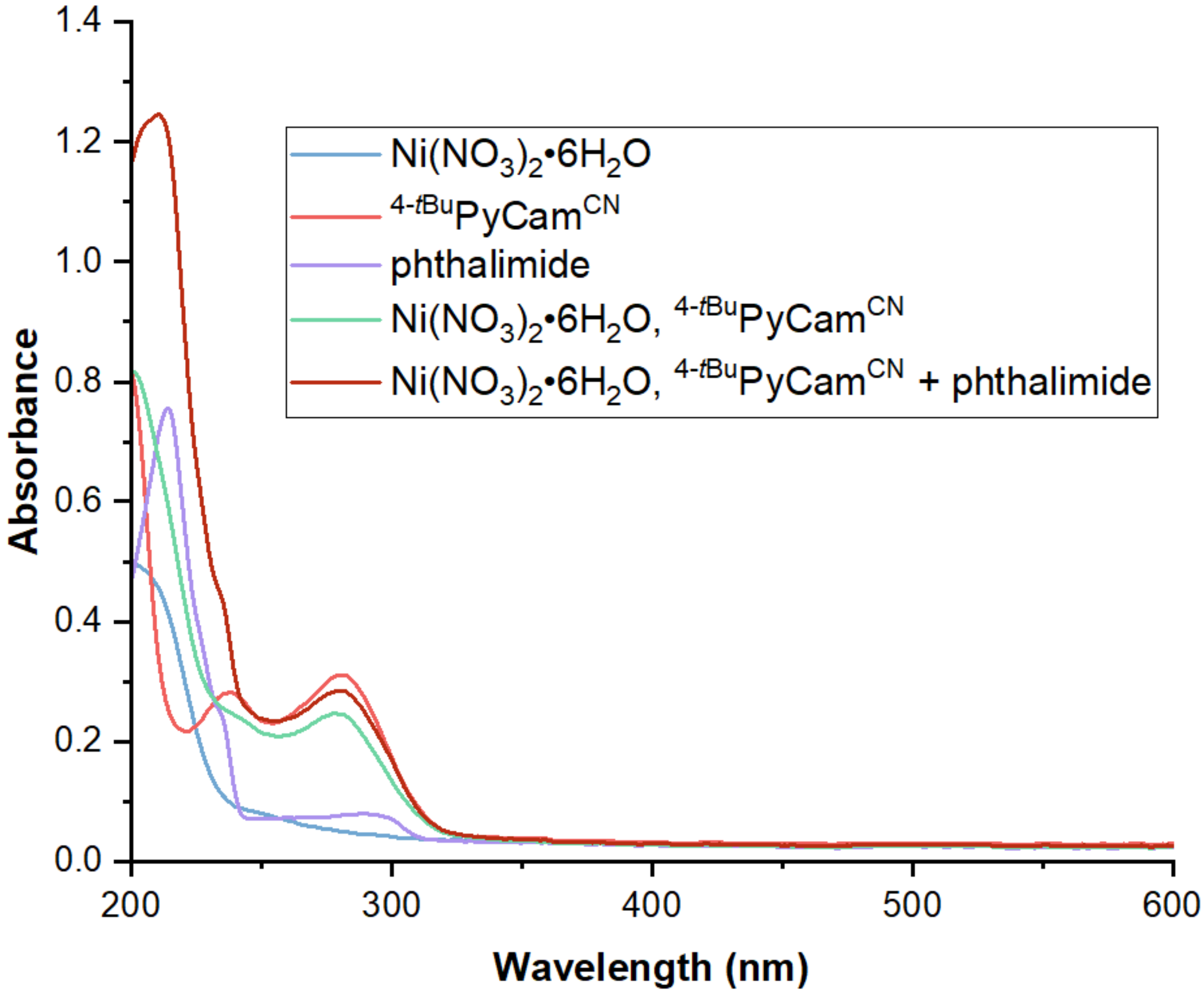

Figure S10. UV-vis spectra for $Ni(NO_3)_2$•$6H_2O$ with $^{4\text{-}tBu}$PyCam^{CN} and phthalimide. UV-vis spectra of $Ni(NO_3)_2$•$6H_2O$ solution (blue), $^{4\text{-}tBu}$PyCam^{CN} solution (pink), phthalimide solution (purple), $Ni(NO_3)_2$•$6H_2O$ with $^{4\text{-}tBu}$PyCam^{CN} solution (green), and $Ni(NO_3)_2$•$6H_2O$ with $^{4\text{-}tBu}$PyCam^{CN} solution with phthalimide (red).

When phthalimide was added, a minute red shift from 279 nm to 282 nm was noted which suggests the interaction of $Ni(NO_3)_2$•$6H_2O$, $^{4\text{-}tBu}$PyCam^{CN} complex with phthalimide.

iii. *$Ni(NO_3)_2$•$6H_2O$, ${}^{4\text{-}tBu}$PyCam^{CN} + TBAI*

To a 1-dram vial, $Ni(NO_3)_2$•$6H_2O$ (1.45 mg, 0.00500 mmol, 1.00 equiv) and ${}^{4\text{-}t\text{Bu}}$PyCam^{CN} (1.01 mg, 0.00500 mmol, 1.00 equiv) were added and diluted with 1.00 mL of MeCN. Subsequently, the mixture was stirred for 30 min at 60 °C. To the mixture, TBAI (1.84 mg, 0.00500 mmol, 1.00 equiv) was added and the solution was stirred for another 30 min at rt. A 10.0 µL aliquot of the solution was taken from the mixture and diluted with MeCN to 2.00 mL in a quartz cuvette. The sample was then subjected to UV-vis analysis. The UV-vis spectrum of the $Ni(NO_3)_2$•$6H_2O$,${}^{4\text{-}t\text{Bu}}$PyCam^{CN}, and TBAI mixture exhibited absorption bands at 247 nm and 280 nm (Figure S11).

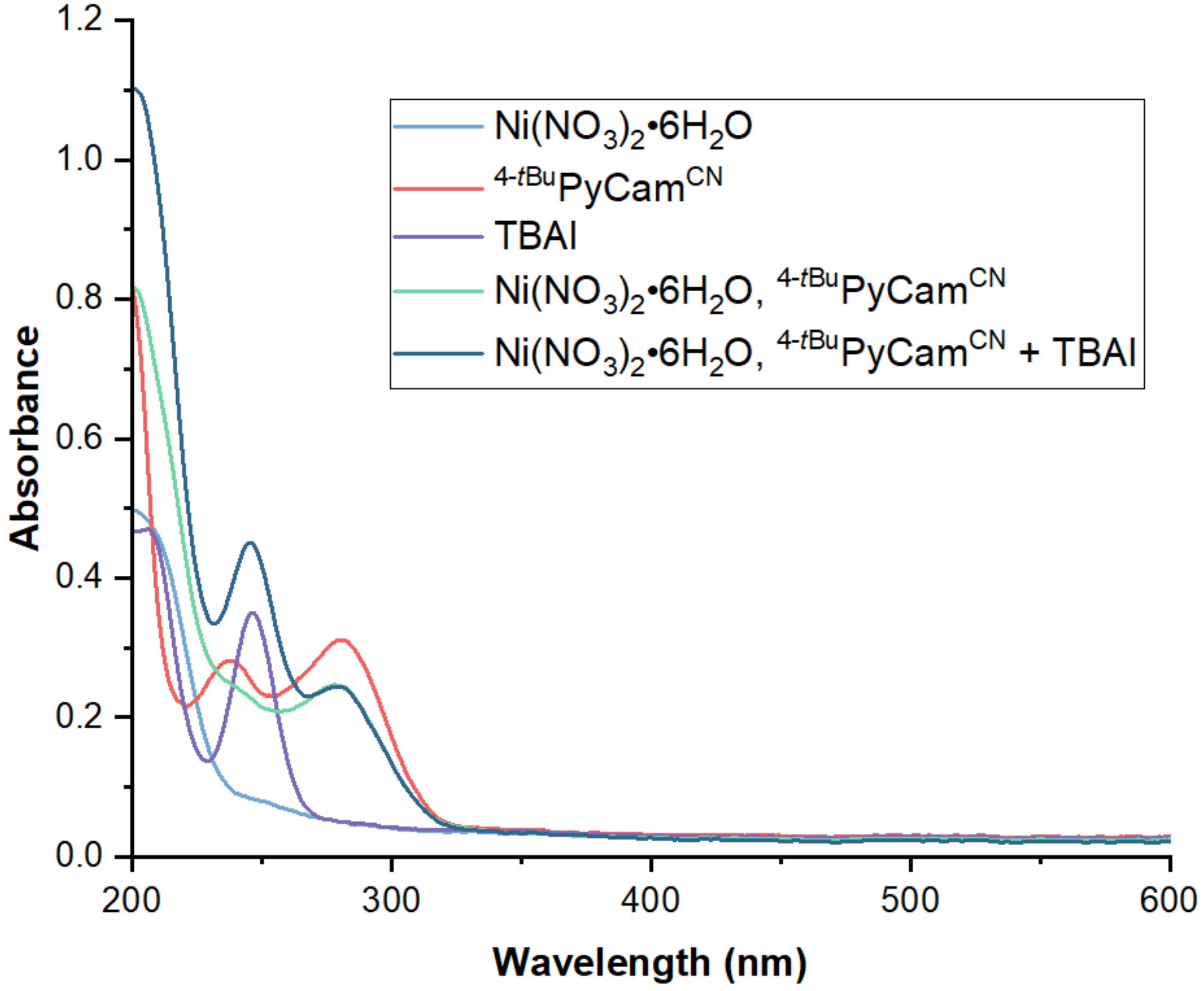

Figure S11. UV-vis spectra for $Ni(NO_3)_2$•$6H_2O$ with ${}^{4\text{-}t\text{Bu}}$PyCam^{CN} and TBAI. UV-vis spectra of $Ni(NO_3)_2$•$6H_2O$ solution (blue), ${}^{4\text{-}t\text{Bu}}$PyCam^{CN} solution (pink), TBAI solution (dark purple),

$Ni(NO_3)_2{\cdot}6H_2O$ with $^{4\text{-}t\text{Bu}}$PyCam^{CN} solution (green), and $Ni(NO_3)_2{\cdot}6H_2O$ with $^{4\text{-}t\text{Bu}}$PyCam^{CN} solution with TBAI (dark blue).

When TBAI was added to Ni, $^{4\text{-}t\text{Bu}}$PyCam^{CN} no significant shift or changes in the absorption between 200-650 nm was observed.

iv. $Ni(NO_3)_2{\cdot}6H_2O$, $^{4\text{-}t\text{Bu}}$PyCam^{CN} + TBAI + phthalimide

To a 1-dram vial, $Ni(NO_3)_2{\cdot}6H_2O$ (1.45 mg, 0.00500 mmol, 1.00 equiv) and $^{4\text{-}t\text{Bu}}$PyCam^{CN} (1.01 mg, 0.00500 mmol, 1.00 equiv) were added and diluted with 1.00 mL of MeCN. Subsequently, the mixture was stirred for 30 min at 60 °C. To the mixture, TBAI (1.84 mg, 0.00500 mmol, 1.00 equiv) and phthalimide (0.74 mg, 0.00500 mmol, 1.00 equiv) were added and the solution was stirred for another 30 min at rt. A 10.0 μL aliquot of the solution was taken from the mixture and diluted with MeCN to 2.00 mL in a quartz cuvette. The sample was then subjected to UV-vis analysis. The UV-vis spectrum of the $Ni(NO_3)_2{\cdot}6H_2O$, $^{4\text{-}t\text{Bu}}$PyCam^{CN}, TBAI, and phthalimide mixture exhibited strong absorbance at 209 nm, very minor absorption bands at 235 nm and 246 nm and a broad absorption band at 280 nm (Figure S12).

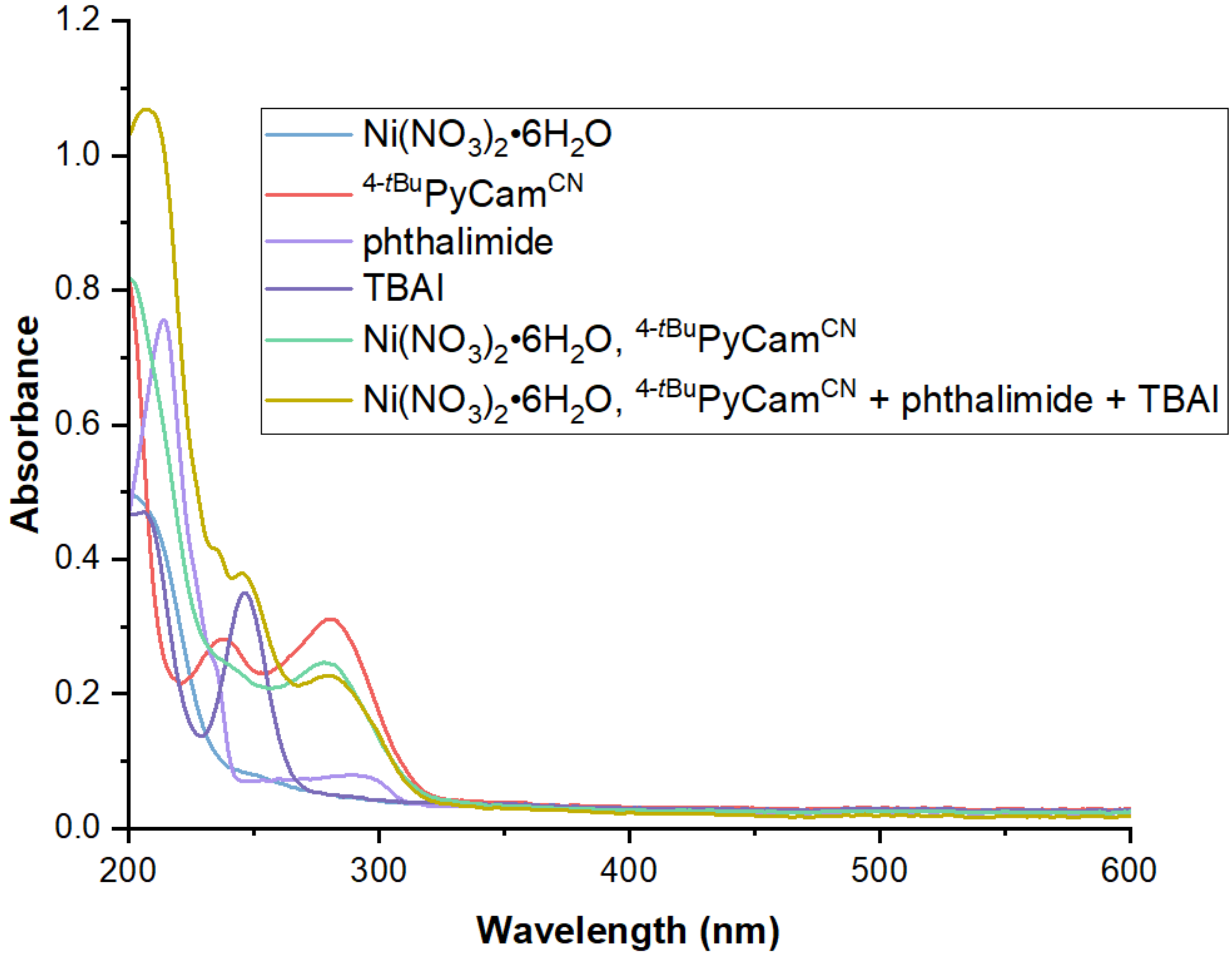

Figure S12. UV-vis spectra for $Ni(NO_3)_2{\cdot}6H_2O$ with $^{4\text{-}t\text{Bu}}$PyCam^{CN} and TBAI and phthalimide. UV-vis spectra of $Ni(NO_3)_2{\cdot}6H_2O$ solution (blue), $^{4\text{-}t\text{Bu}}$PyCam^{CN} solution (dark orange), phthalimide solution (purple), TBAI solution (dark purple), $Ni(NO_3)_2{\cdot}6H_2O$ with $^{4\text{-}t\text{Bu}}$PyCam^{CN} solution (green), and $Ni(NO_3)_2{\cdot}6H_2O$ with $^{4\text{-}t\text{Bu}}$PyCam^{CN} solution with phthalimide and TBAI (yellow).

Similar to the reaction with the preformed $Ni(NO_3)_2{\cdot}6H_2O$, $^{4\text{-}t\text{Bu}}$PyCam^{CN} complex in the presence of phthalimide (Figure S10, red), a minute red shift from was observed when both phthalimide and TBAI were added. This result suggests that only phthalimide interacts with $Ni(NO_3)_2{\cdot}6H_2O$, $^{4\text{-}t\text{Bu}}$PyCam^{CN} complex.

v. $Ni(NO_3)_2{\cdot}6H_2O$, $^{4\text{-}t\text{Bu}}$PyCam^{CN} + ArI (1-chloro-3-iodo-5-(trifluoromethyl)benzene)

To a 1-dram vial, $Ni(NO_3)_2{\cdot}6H_2O$ (1.45 mg, 0.00500 mmol, 1.00 equiv) and $^{4\text{-}t\text{Bu}}$PyCam^{CN} (1.01 mg, 0.00500 mmol, 1.00 equiv) were added and diluted with 1.00 mL of MeCN. Subsequently, the mixture was stirred for 30 min at 60 °C. To the mixture, 1-chloro-3-iodo-5-(trifluormethyl)benzene (0.78 μL, 0.00500 mmol, 1.00 equiv) was added and the solution was stirred for another 30 min at rt. A 10.0 μL aliquot of the solution was taken from the mixture and diluted with MeCN to 2.00 mL in a quartz

cuvette. The sample was then subjected to UV-vis analysis. The UV-vis spectrum of the $Ni(NO_3)_2{\cdot}6H_2O$, $^{4\text{-}tBu}$PyCam^{CN}, and 1-chloro-3-iodo-5-(trifluormethyl)benzene mixture exhibited the broadening the broadening of the absorbance peak at around 240 nm (Figure S13).

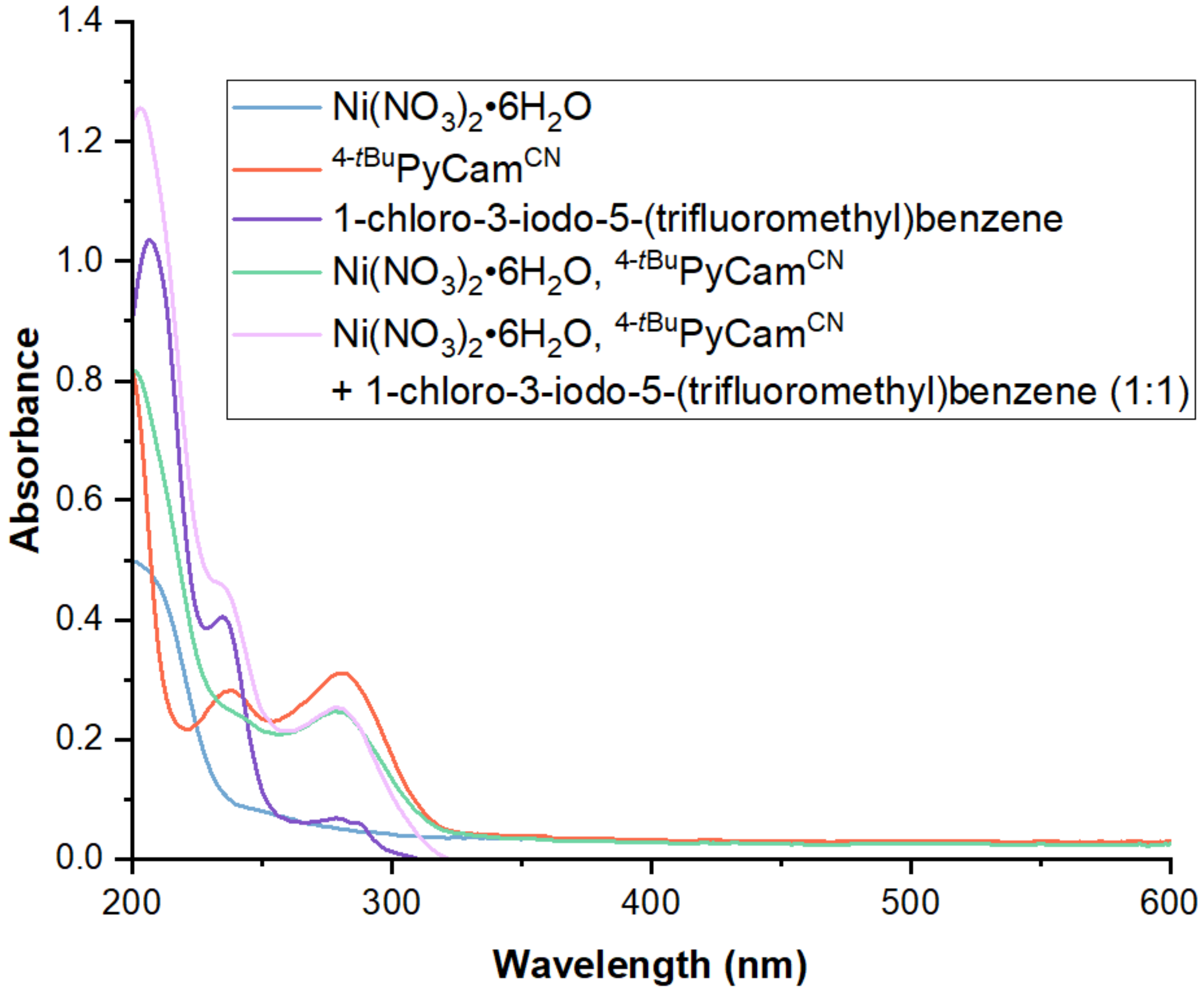

Figure S13. UV-vis spectra for $Ni(NO_3)_2{\cdot}6H_2O$ with $^{4\text{-}tBu}$PyCam^{CN} and 1-chloro-3-iodo-5-(trifluormethyl)benzene. UV-vis spectra of $Ni(NO_3)_2{\cdot}6H_2O$ solution (blue), $^{4\text{-}tBu}$PyCam^{CN} solution (dark orange), 1-chloro-3-iodo-5-(trifluormethyl)benzene solution (dark purple), $Ni(NO_3)_2{\cdot}6H_2O$ with $^{4\text{-}tBu}$PyCam^{CN} solution (green), and $Ni(NO_3)_2{\cdot}6H_2O$ with $^{4\text{-}tBu}$PyCam^{CN} solution with phthalimide and 1-chloro-3-iodo-5-(trifluormethyl)benzene (pink).

vi. $Ni(NO_3)_2{\cdot}6H_2O$, Cyclam

To a 1-dram vial, $Ni(NO_3)_2{\cdot}6H_2O$ (1.45 mg, 0.00500 mmol, 1.00 equiv) and cyclam (1.00 mg, 0.00500 mmol, 1.00 equiv) were added and diluted with 2.00 mL of MeCN. Subsequently, the mixture was stirred for 30 min at 60 °C. A 20.0 μL aliquot of the solution was taken from the mixture and diluted to 2.00 mL in a quartz cuvette. The sample was then subjected to UV-vis analysis. The UV-vis spectrum

of the $Ni(NO_3)_2•6H_2O$ and cyclam mixture did not show any significant absorption between 200-600 nm (Figure S14).

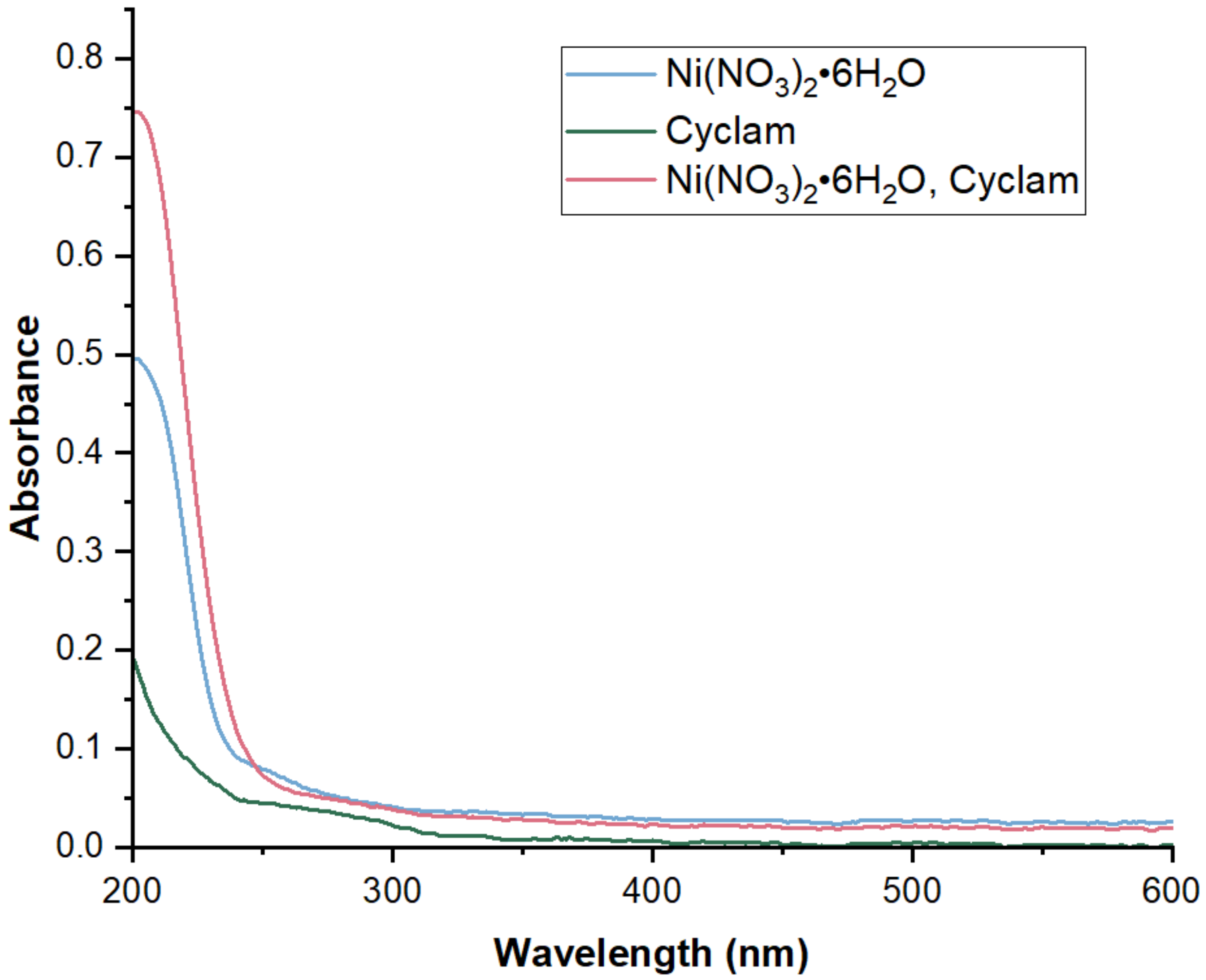

Figure S14. UV-Vis spectra for Ni nitrate with cyclam. UV-vis spectra of $Ni(NO_3)_2•6H_2O$ solution (blue), cyclam solution (green), and $Ni(NO_3)_2•6H_2O$ with cyclam solution (purple).

C. UV-vis Spectra of Fe Complex

1. $FeCl_3$

To a 1-dram vial, $FeCl_3$ (0.81 mg, 0.00500 mmol) was added and diluted with 1.00 mL of MeCN. A 10.0 μL aliquot of the solution was taken from the mixture and diluted with MeCN to 2.00 mL in a quartz cuvette. The sample was then subjected to UV-vis analysis. The UV-vis spectrum of $FeCl_3$ exhibited strong absorption bands at 240 nm, 310 nm, and 360 nm (Figure S15).

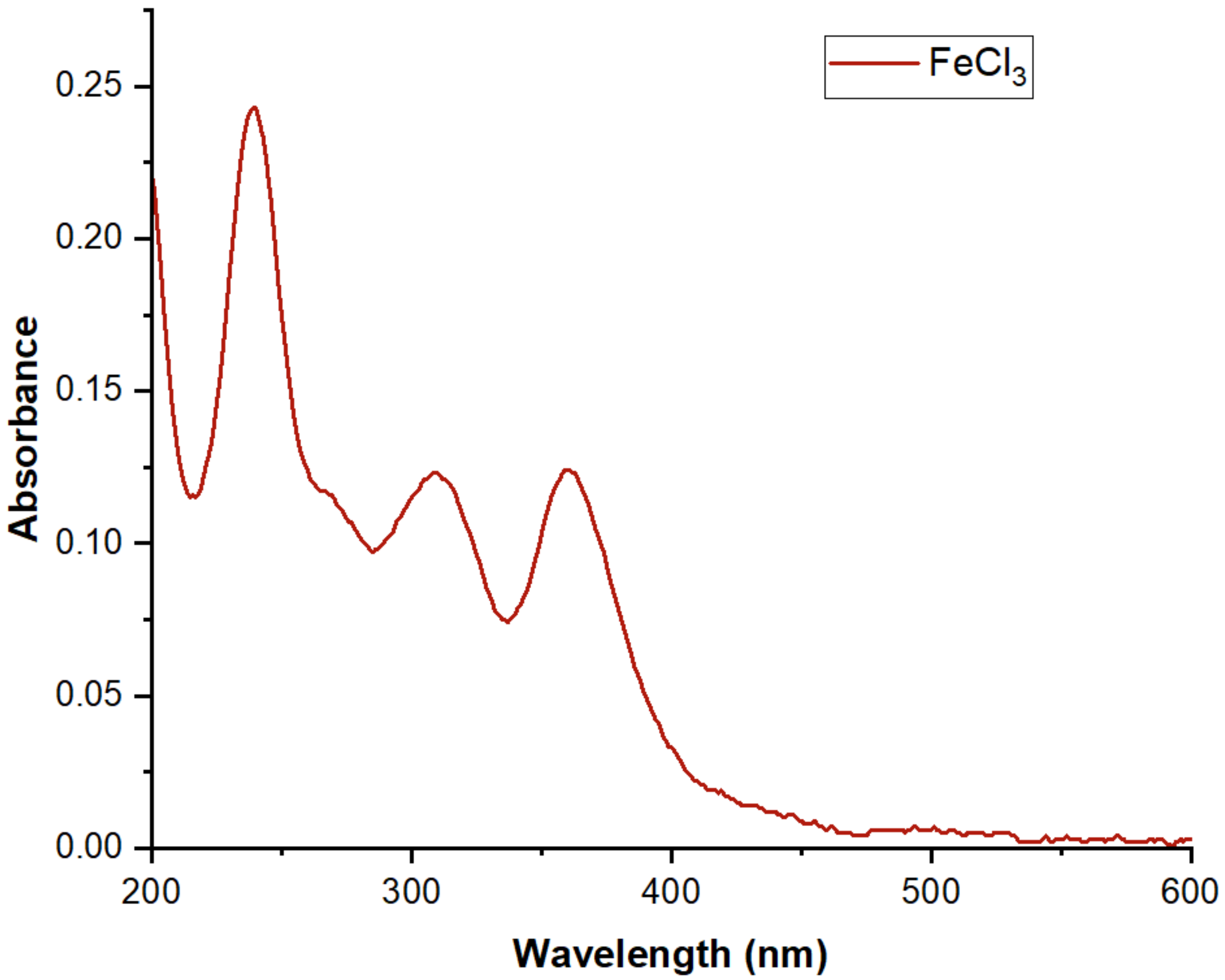

Figure S15. UV-vis spectra for $FeCl_3$.

2. $FeCl_3$, Cyclam

To a 1-dram vial, $FeCl_3$ (0.81 mg, 0.00500 mmol, 1.00 equiv) and cyclam (1.00 mg, 0.00500 mmol, 1.00 equiv) were added and diluted with 2.00 mL of MeCN. Subsequently, the mixture was stirred for 30 min at 60 °C. A 20.0 μL aliquot of the solution was taken from the mixture and diluted with MeCN to 2.00 mL in a quartz cuvette sealed with a cap. The sample was then subjected to UV-vis analysis. The UV-vis spectrum of the $FeCl_3$ and cyclam mixture exhibited absorption bands at 247 nm and 340 nm (Figure S16).

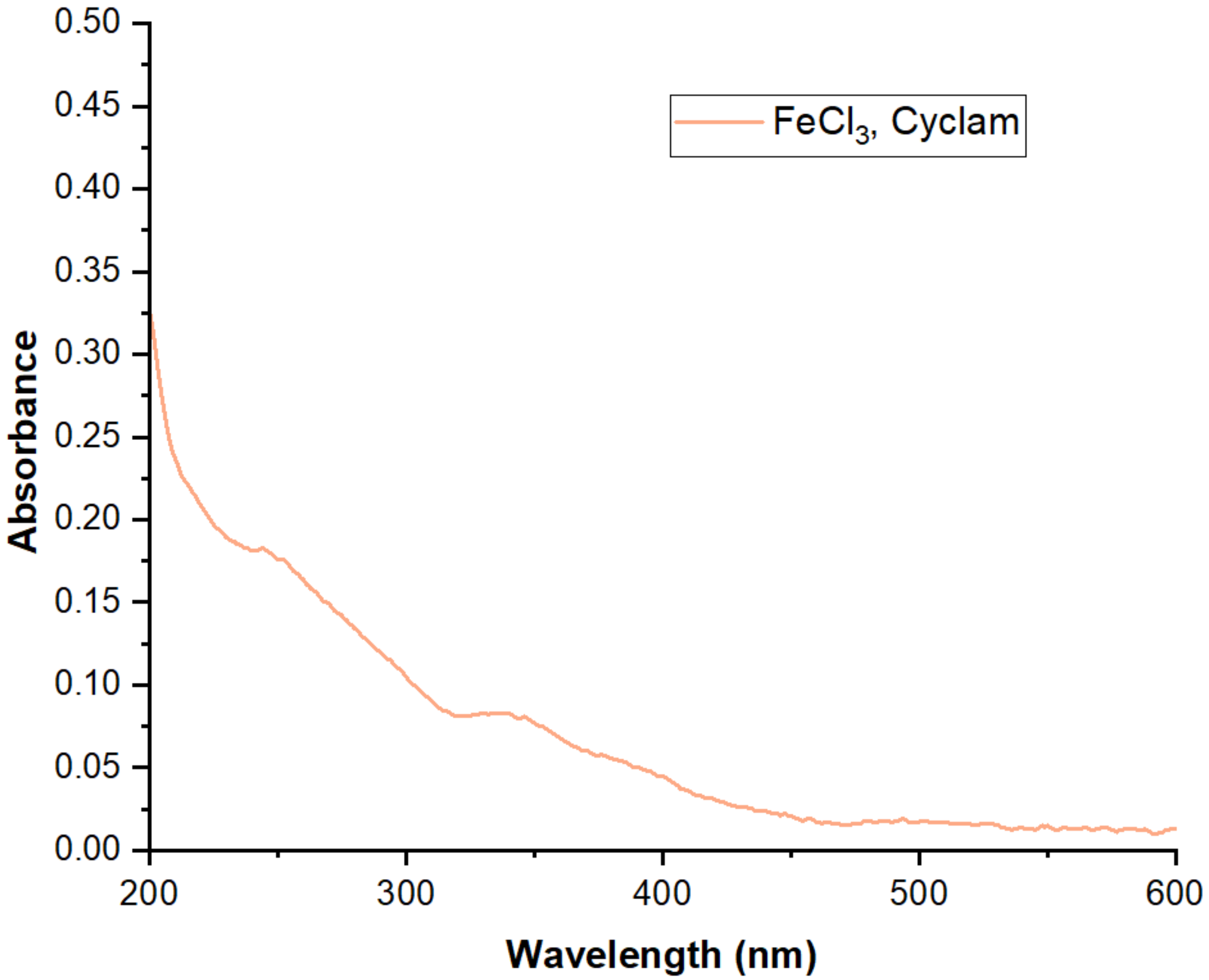

Figure S16. UV-vis spectra for $FeCl_3$ with cyclam.

The disappearance of the signals at 240 nm, 310 nm, and 360 nm which corresponds to $FeCl_3$ was noticed and suggests the formation of $FeCl_3$, cyclam complex.

3. Combined Spectra for $FeCl_3$, Cyclam

i. $FeCl_3$, Cyclam

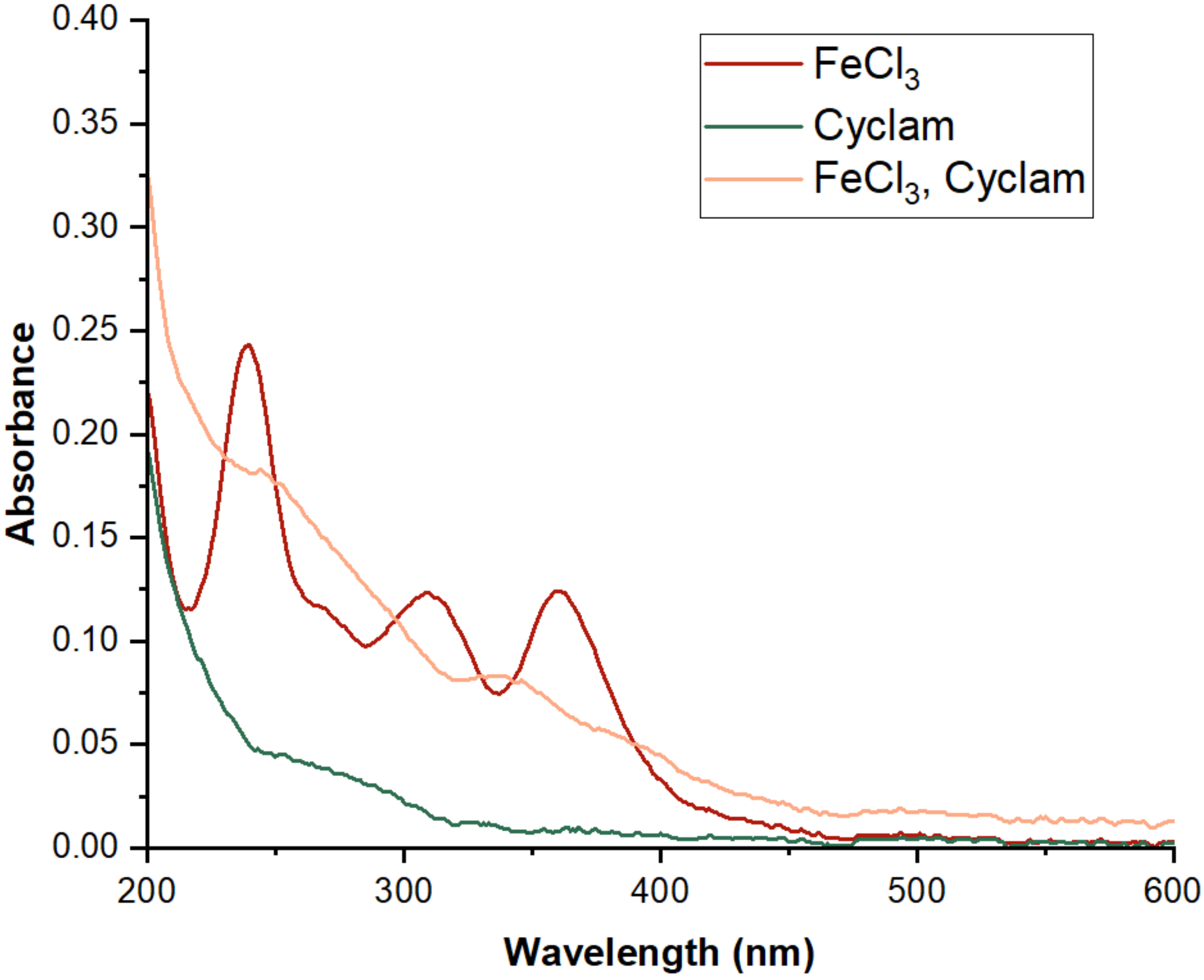

Figure S17. UV-vis spectra for $FeCl_3$ with cyclam. UV-vis spectra of $FeCl_3$ solution (red), cyclam solution (green), and $FeCl_3$ with cyclam solution (orange).

ii. *$FeCl_3$ + phthalimide*

To a 1-dram vial, $FeCl_3$ (0.81 mg, 0.00500 mmol, 1.00 equiv) and phthalimide (0.74 mg, 0.00500 mmol, 1.00 equiv) were added and diluted with 1.00 mL of MeCN. Subsequently, the mixture was stirred for 30 min at rt. A 10.0 μL aliquot of the solution was taken from the mixture and diluted with MeCN to 2.00 mL in a quartz cuvette sealed with a cap. The sample was then subjected to UV-vis analysis. The UV-vis spectrum of the $FeCl_3$ and phthalimide mixture exhibited very minor absorption bands at 214 nm and 234 nm and broad absorption bands at 300 nm and 360 nm (Figure S18).

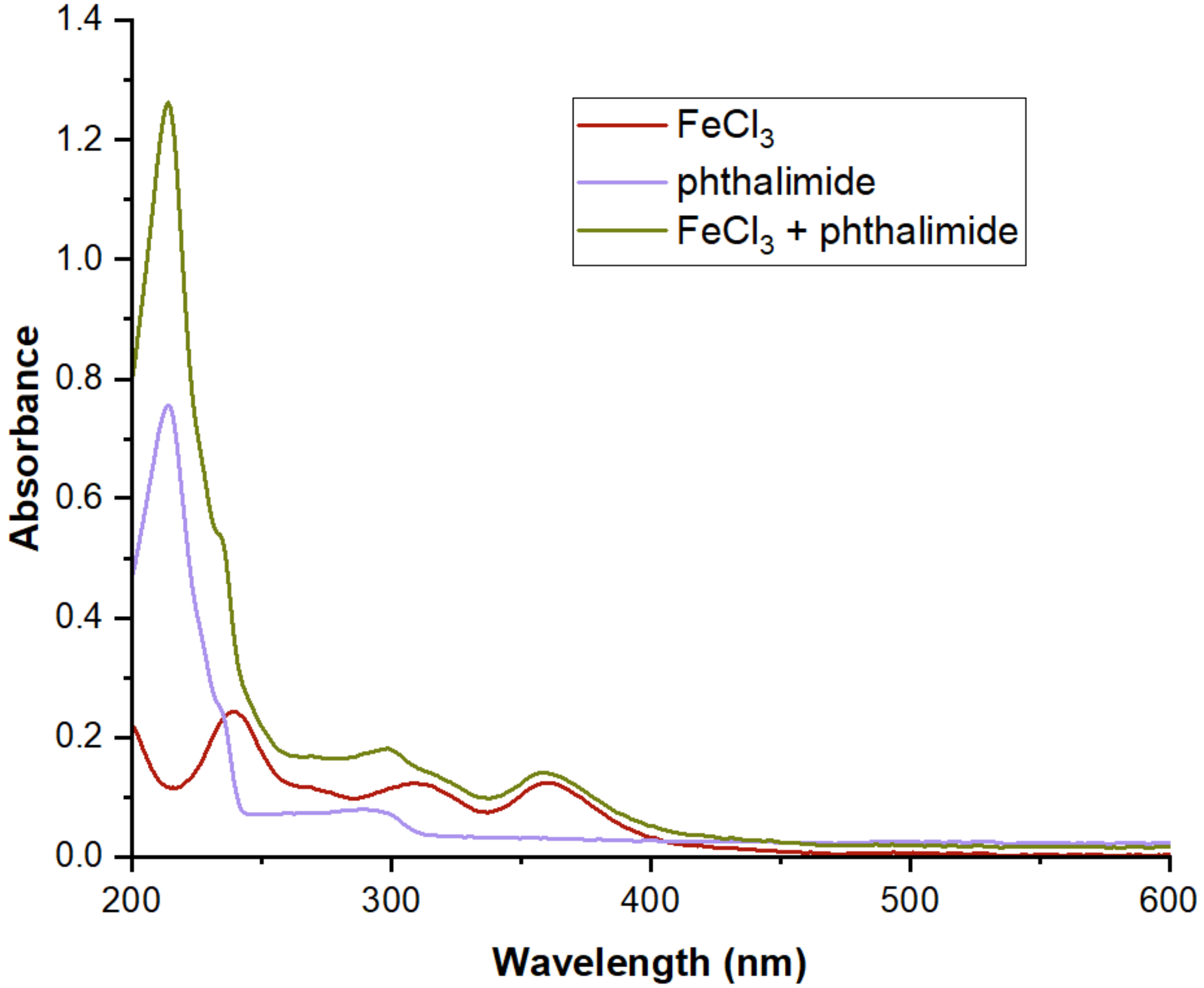

Figure S18. UV-vis spectra for $FeCl_3$ with phthalimide. UV-vis spectra of $FeCl_3$ solution (red), phthalimide solution (purple), and $FeCl_3$ solution with phthalimide (green).

A disappearance of signal at 240 nm in the resultant spectrum which corresponds to $FeCl_3$ and the appearance of a new peak at 301 nm was observed. This result suggests the interaction between phthalimide with $FeCl_3$.

iii. $FeCl_3$ + TBAI

To a 1-dram vial, $FeCl_3$ (0.81 mg, 0.00500 mmol, 1.00 equiv) and TBAI (1.84 mg, 0.00500 mmol, 1.00 equiv) were added and diluted with 1.00 mL of MeCN. Subsequently, the mixture was stirred for 30 min at rt. A 10.0 μL aliquot of the solution was taken from the mixture and diluted with MeCN to 2.00 mL in a quartz cuvette sealed with a cap. The sample was then subjected to UV-vis analysis. The UV-vis spectrum of the $FeCl_3$ and TBAI mixture exhibited a weak absorption band starting at 240 nm and strong absorption bands at 292 nm and 360 nm (Figure S19).

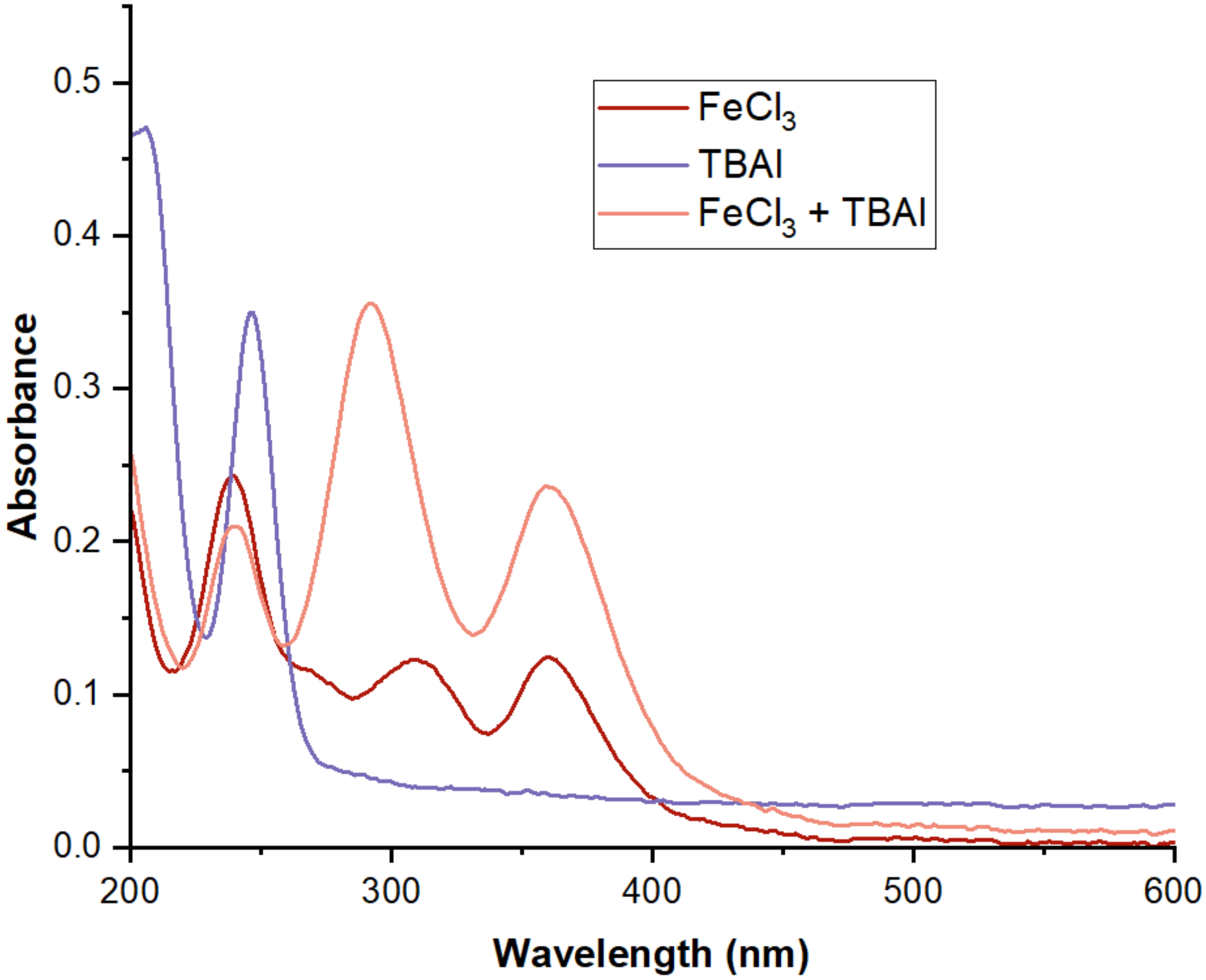

Figure S19. UV-vis spectra for $FeCl_3$ with TBAI. UV-vis spectra of $FeCl_3$ solution (red), TBAI solution (dark purple), and $FeCl_3$ solution with TBAI (orange).

When TBAI is added, the disappearance of the signal at 274 nm and the appearance of a new signal at 292 nm, which suggests an interaction between TBAI and $FeCl_3$.

iv. $FeCl_3$ + TBAI + phthalimide

To a 1-dram vial, $FeCl_3$ (0.81 mg, 0.00500 mmol, 1.00 equiv), TBAI (1.84 mg, 0.00500 mmol, 1.00 equiv), and phthalimide (0.74 mg, 0.00500 mmol, 1.00 equiv) were added and diluted with 1.00 mL of MeCN. Subsequently, the mixture was stirred for 30 min at rt. A 10.0 μL aliquot of the solution was taken from the mixture and diluted with MeCN to 2.00 mL in a quartz cuvette sealed with a cap. The sample was then subjected to UV-vis analysis. The UV-vis spectrum of $FeCl_3$, phthalimide, and TBAI mixture exhibited very minor absorption bands at 214 nm and 235 nm and broad absorption bands at 290 nm and 360 nm (Figure S20).

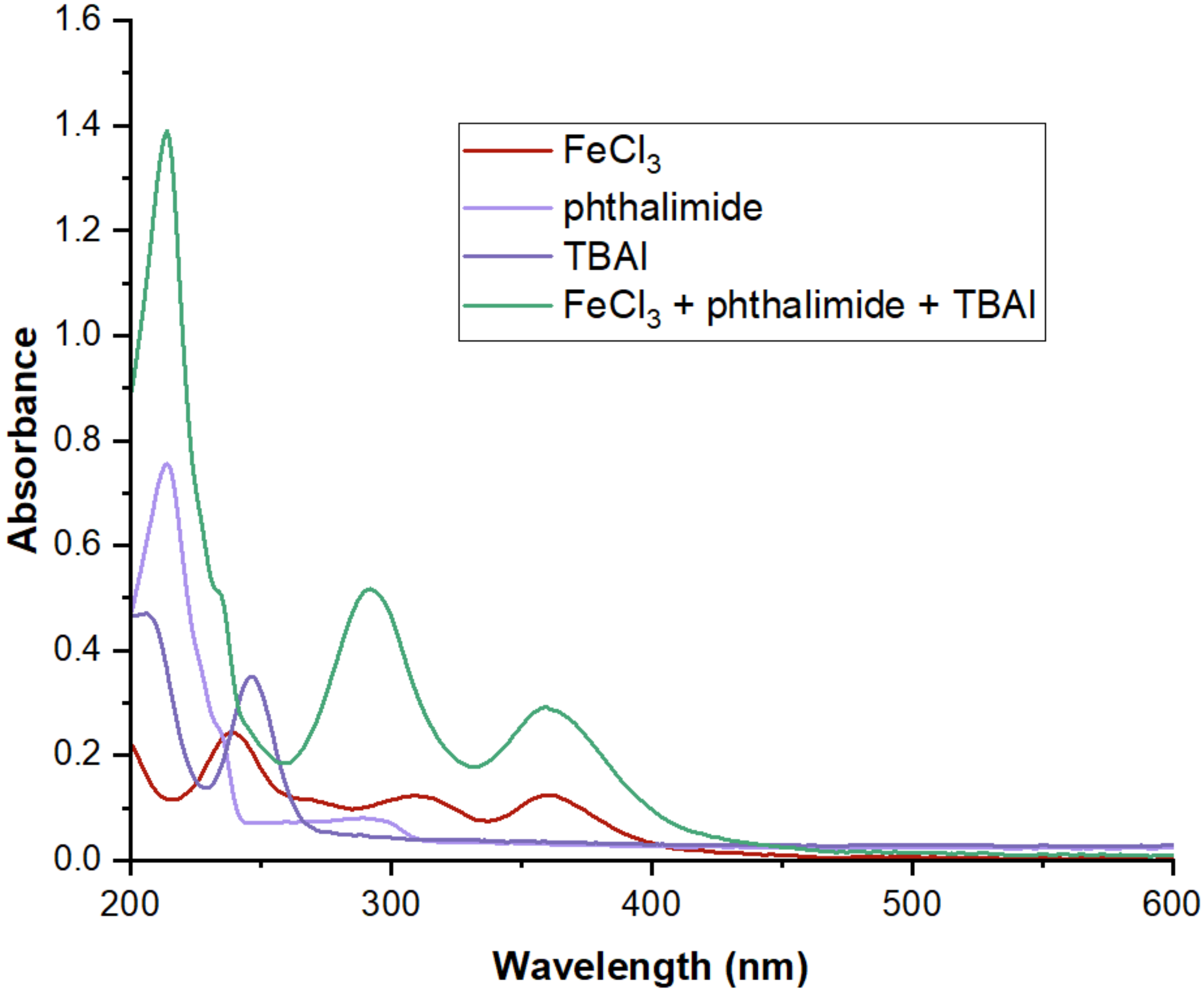

Figure S20. UV-vis spectra for $FeCl_3$ with additives. UV-vis spectra of $FeCl_3$ solution (red), phthalimide solution (purple), TBAI solution (dark purple), and $FeCl_3$ solution with TBAI and phthalimide (green).

In the presence of TBAI and phthalimide, the disappearance of the signal a 240 nm and 274 nm and the appearance of new signal at 292 nm was observed. This result suggests that $FeCl_3$ can interact and form new species in the presence of these additives.

v. *$FeCl_3$, Cyclam + phthalimide*

To a 1-dram vial, $FeCl_3$ (0.81 mg, 0.00500 mmol, 1.00 equiv) and cyclam (1.00 mg, 0.00500 mmol, 1.00 equiv) were added and diluted with 2.00 mL of MeCN. Subsequently, the mixture was stirred for 30 min at 60 °C. To the mixture, phthalimide (0.74 mg, 0.00500 mmol, 1.00 equiv) was added and the solution was stirred for another 30 min at rt. A 20.0 μL aliquot of the solution was taken from the mixture and diluted with MeCN to 2.00 mL in a quartz cuvette. The sample was then subjected to UV-vis analysis. The UV-vis spectrum of the $FeCl_3$, Cyclam and phthalimide mixture exhibited absorbance bands at 214 nm, 236 nm, 295 nm, and 362 nm (Figure S21).

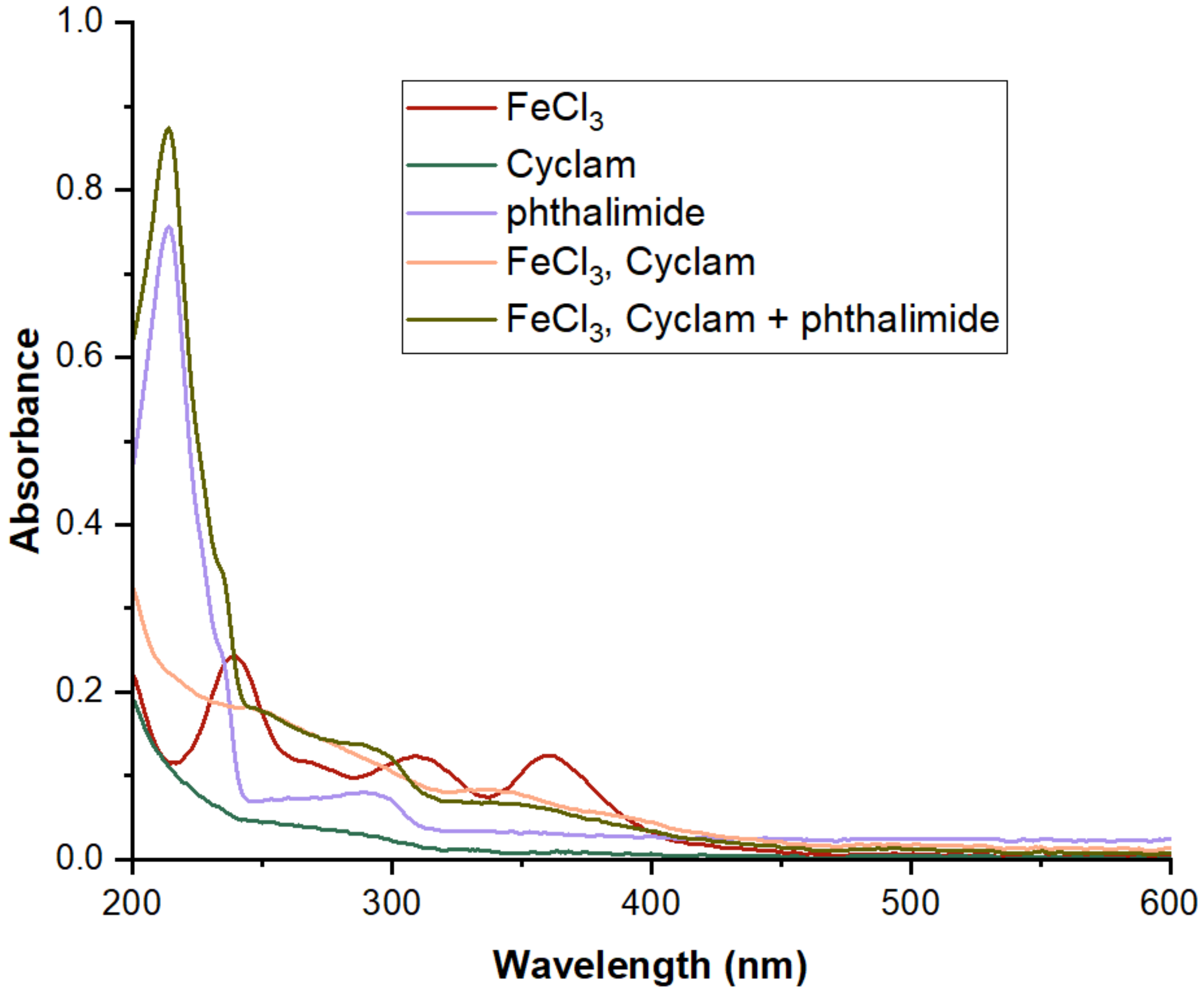

Figure S21. UV-vis spectra for $FeCl_3$ with cyclam with phthalimide. UV-vis spectra of $FeCl_3$ solution (red), cyclam solution (green), phthalimide solution (purple), $FeCl_3$ with cyclam solution (orange), $FeCl_3$ with cyclam solution with phthalimide (dark green).

When phthalimide is added to $FeCl_3$, cyclam, no new absorbances that correspond to neither phthalimide nor $FeCl_3$, cyclam were observed suggesting no observable interactions in the UV-vis region between the two species.

vi. $FeCl_3$, Cyclam + TBAI

To a 1-dram vial, $FeCl_3$ (0.81 mg, 0.00500 mmol, 1.00 equiv) and cyclam (1.00 mg, 0.00500 mmol, 1.00 equiv) were added and diluted with 2.00 mL of MeCN. Subsequently, the mixture was stirred for 30 min at 60 °C. To the mixture, TBAI (1.84 mg, 0.00500 mmol, 1.00 equiv) was added and the solution was stirred for another 30 min at rt. A 20.0 μL aliquot of the solution was taken from the mixture and diluted with MeCN to 2.00 mL in a quartz cuvette. The sample was then subjected to UV-vis analysis. The UV-vis spectrum of the $FeCl_3$, Cyclam and TBAI mixture exhibited strong absorbance at 247 nm and broad absorbances peaks at 295 nm and 350 nm (Figure S22).

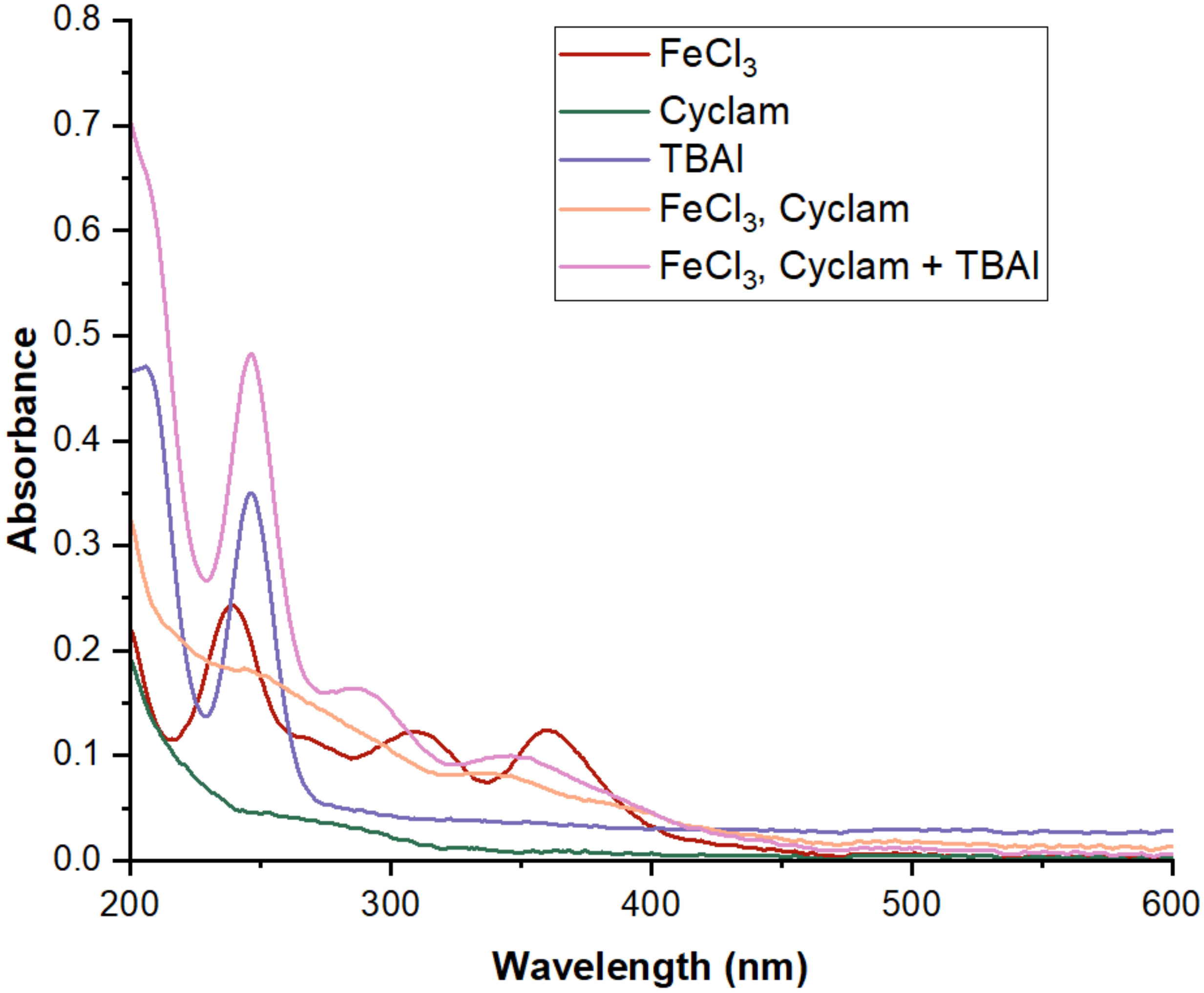

Figure S22. UV-vis spectra for $FeCl_3$ with cyclam with TBAI. UV-vis spectra of $FeCl_3$ solution (red), cyclam solution (green), TBAI solution (dark purple), $FeCl_3$ with cyclam solution (orange), $FeCl_3$ with cyclam solution with TBAI (pink).

The shift in the peak at 360 nm and the appearance of a peak at 295 nm suggests an interaction is present between the Fe, cyclam species and TBAI.

vii. $FeCl_3$, Cyclam + TBAI + phthalimide

To a 1-dram vial, $FeCl_3$ (0.81 mg, 0.00500 mmol, 1.00 equiv) and cyclam (1.00 mg, 0.00500 mmol, 1.00 equiv) were added and diluted with 2.00 mL of MeCN. Subsequently, the mixture was stirred for 30 min at 60 °C. To the mixture, TBAI (1.84 mg, 0.00500 mmol, 1.00 equiv) and phthalimide (0.74 mg, 0.00500 mmol, 1.00 equiv) were added and the solution was stirred for another 30 min at rt. A 20.0 μL aliquot of the solution was taken from the mixture and diluted with MeCN to 2.00 mL in a quartz cuvette. The sample was then subjected to UV-vis analysis. The UV-vis spectrum of the $FeCl_3$, Cyclam and TBAI mixture exhibited a strong absorbance at 213 nm, minor absorbances at 236 nm and 248 nm, and broad absorbances at 295 nm and 361 nm (Figure S23).

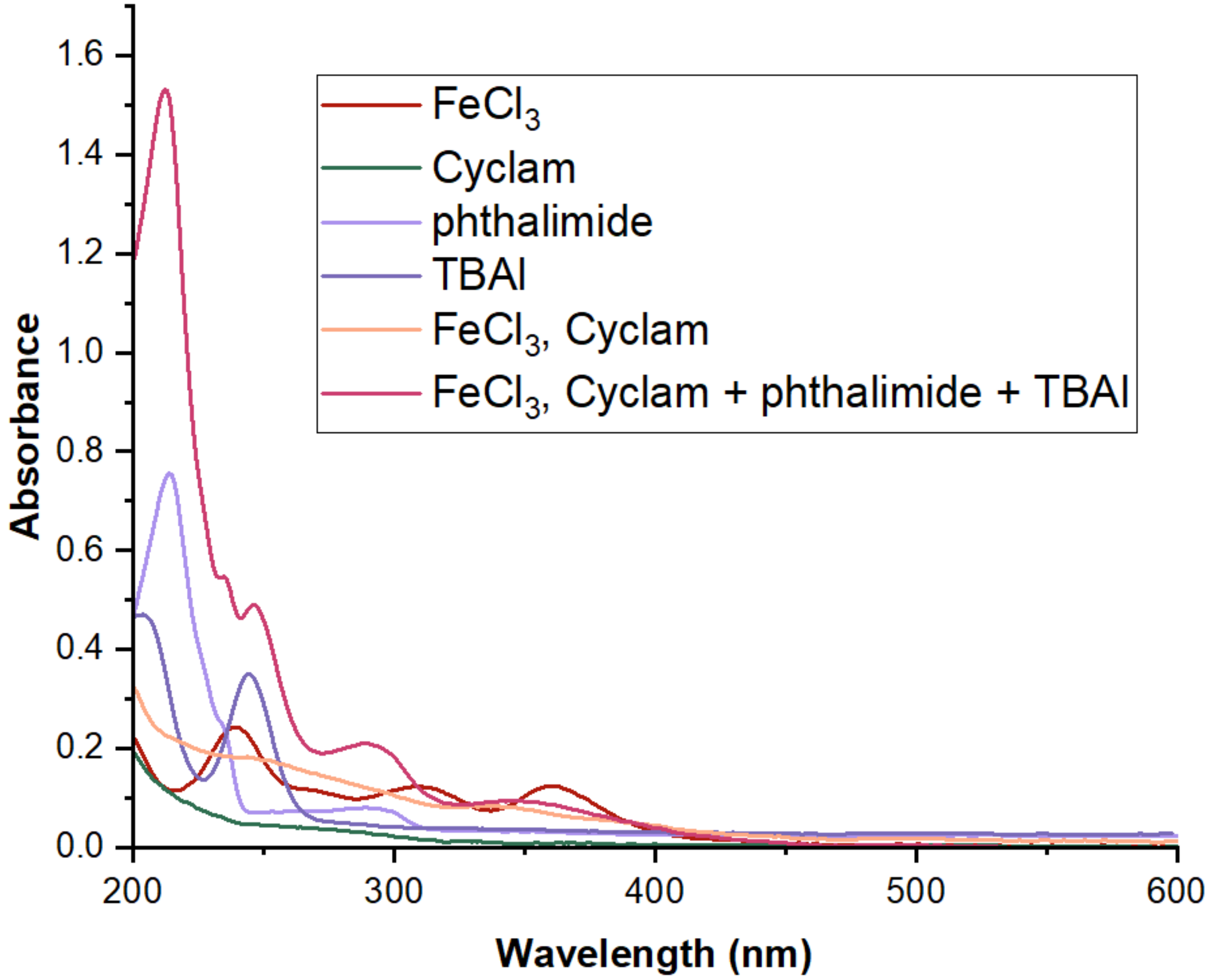

Figure S23. UV-vis spectra for $FeCl_3$ with cyclam with TBAI and phthalimide. UV-vis spectra of $FeCl_3$ solution (red), cyclam solution (green), phthalimide solution (purple), TBAI solution (dark purple), $FeCl_3$ with cyclam solution (orange), $FeCl_3$ with cyclam solution with TBAI and phthalimide (dark pink).

The appearance of the shifted peak at 295 nm suggests an interaction when Fe, cyclam is mixed with the additives. The similarity of the graph (Figure S23, dark pink) to the spectra obtained when mixing Fe, cyclam with TBAI (Figure S22, pink), suggesting an interaction of the Fe, cyclam with TBAI.

viii. $FeCl_3$, ${}^{4\text{-}tBu}$PyCam^{CN}

To a 1-dram vial, $FeCl_3$ (0.81 mg, 0.00500 mmol, 1.00 equiv) and ${}^{4\text{-}tBu}$PyCam^{CN} (1.01 mg, 0.00500 mmol, 1.00 equiv) were added and diluted with 1.00 mL of MeCN. Subsequently, the mixture was stirred for 30 min at 60 °C. A 10.0 µL aliquot of the solution was taken from the mixture and diluted with MeCN to 2.00 mL in a quartz cuvette sealed with a cap. The sample was then subjected to UV-vis analysis. The UV-vis spectrum of the $FeCl_3$ and ${}^{4\text{-}tBu}$PyCam^{CN} mixture exhibited an absorption band starting at 242 nm and weak absorption bands at 276 nm, and 360 nm (Figure S24).

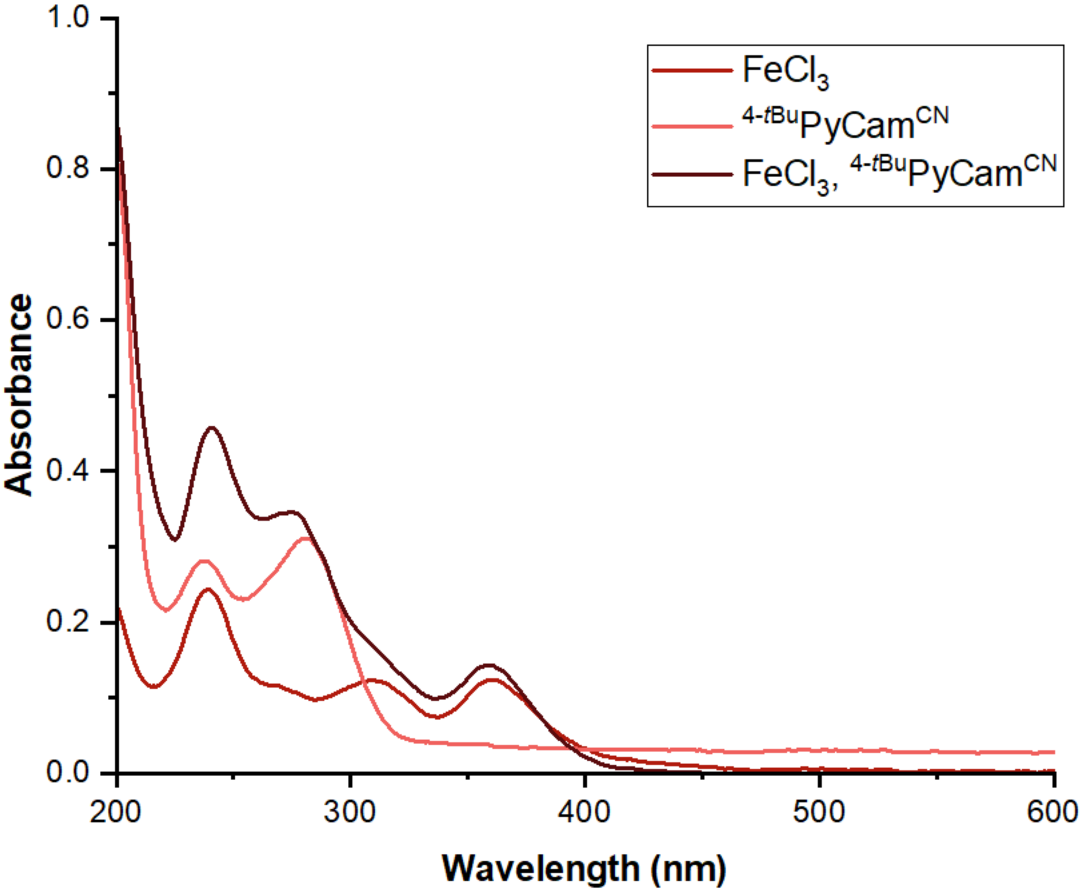

Figure S24. UV-Vis spectra for $FeCl_3$ with ${}^{4\text{-}tBu}$PyCam^{CN}. UV-vis spectra of $FeCl_3$ solution (red), ${}^{4\text{-}tBu}$PyCam^{CN} solution (pink), and $FeCl_3$ with ${}^{4\text{-}tBu}$PyCam^{CN} solution (dark brown).

VII. Matched and Mis-Matched Metal-Ligand Pairings under Standard Reaction Conditions

To gain insight into the favorability of ligand exchange between Fe and Ni complexes in this decarboxylative arylation method, mixtures of Fe and Ni salts with and without ligand were examined.

A. Activated Acid: 2-Phenoxyacetic Acid

Table S24. Summary of yields using 2-phenoxyacetic acid[a,b]

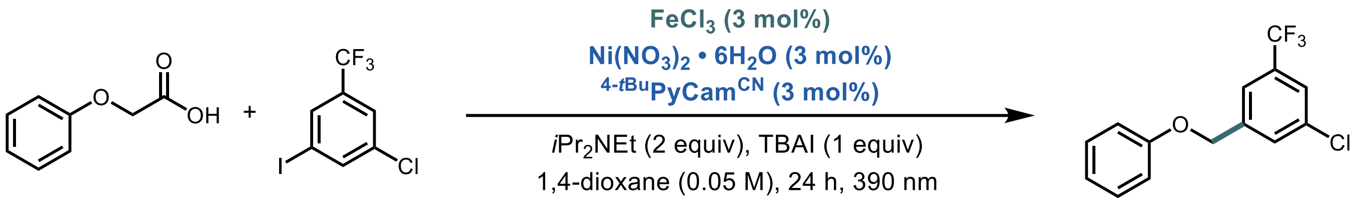

entry	deviation from above	product (%)
1	none	94
2	no $^{4\text{-}t\text{Bu}}$PyCam^{CN}	0
3	Fe with cyclam	90
4	no ligand on Ni, Fe with $^{4\text{-}t\text{Bu}}$PyCam^{CN}	87
5	Fe with $^{4\text{-}t\text{Bu}}$PyCam^{CN}, Ni with cyclam	64
6	Fe and Ni with $^{4\text{-}t\text{Bu}}$PyCam^{CN}	68
7	Fe and Ni with cyclam	9

[a] Reactions were set up following the general procedure for optimization in the decarboxylative arylation using 2-phenoxyacetic acid (0.130 mmol, 1.00 equiv) and 1-chloro-3-iodo-5-(trifluoromethyl)benzene (0.100 mmol, 1.00 equiv) in 2.00 mL of 1,4-dioxane. [b] Yields were determined by ^{1}H NMR spectroscopy using dibromomethane as an internal standard.

B. Unactivated Acid: 2,3-Dihydro-1H-indene-2-carboxylic Acid

Table S25. Summary of yields using 2,3-dihydro-1H-indene-2-carboxylic acid [a,b]

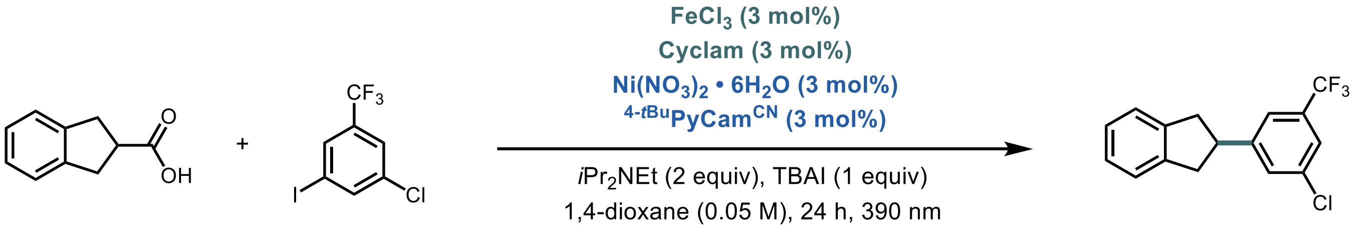

entry	deviation from above	product (%)
1	none	80
2	no cyclam	0
3	no cyclam, no $^{4\text{-}t\text{Bu}}$PyCam^{CN}	0
4	no ligand on Ni, Fe with $^{4\text{-}t\text{Bu}}$PyCam^{CN}	4
5	Fe with $^{4\text{-}t\text{Bu}}$PyCam^{CN}, Ni with cyclam	0
6	Fe and Ni with $^{4\text{-}t\text{Bu}}$PyCam^{CN}	10
7	Fe and Ni with cyclam	0

[a] Reactions were set up following the general procedure for unactivated carboxylic acid scope in the decarboxylative arylation using 2,3-dihydro-1H-indene-2-carboxylic acid (0.130 mmol, 1.30 equiv) and 1-chloro-3-iodo-5-(trifluoromethyl)benzene (0.100 mmol, 1.00 equiv) in 2.00 mL of 1,4-dioxane. [b] Yields were determined by ^{19}F NMR spectroscopy using fluorobenzene as an internal standard.

VIII. Mechanistic Experiments

A. Time Course Studies

1. Standard Reaction Using 2-Phenoxyacetic Acid

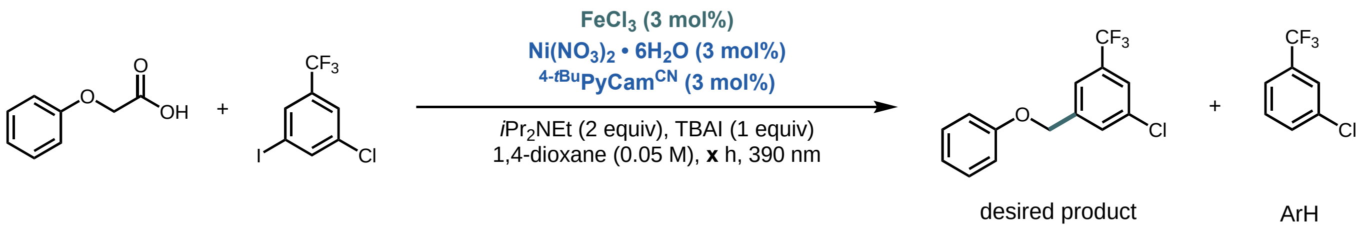

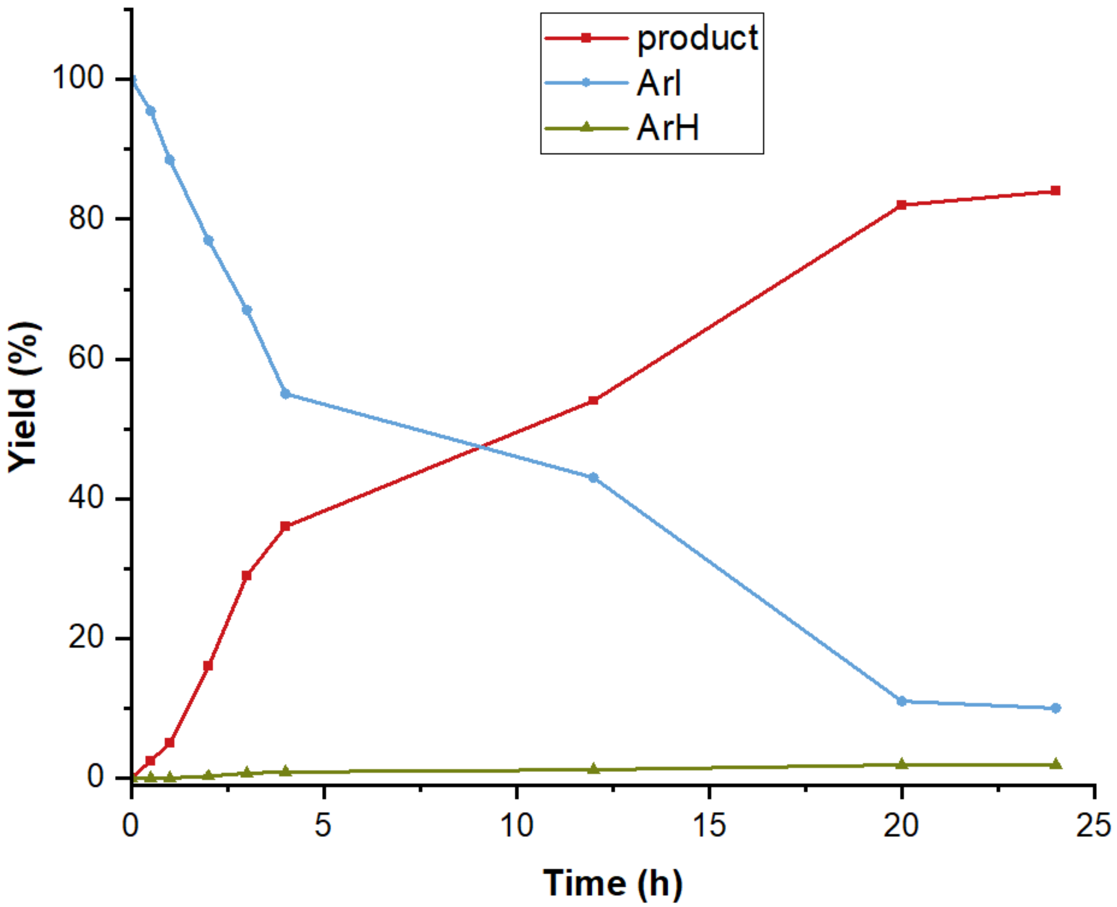

Figure S25. Reaction profile diagram for the coupling of 2-phenoxyacetic acid with 1-chloro-3-iodo-5-(trifluoromethyl)benzene. Yields for desired product and ArI were determined by ^1H NMR spectroscopy using 1,3,5-trimethoxybenzene as an internal standard. Yields for ArH were determined by GC/MS analysis using biphenyl as an internal standard.

2. Standard Reaction Using 2,3-Dihydro-1H-indene-2-carboxylic Acid

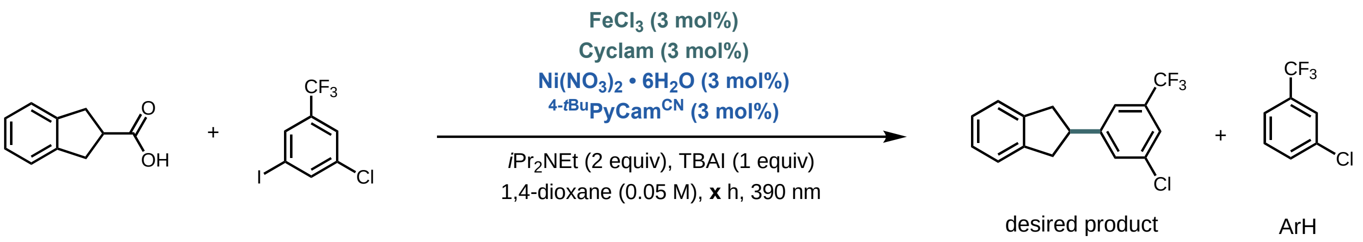

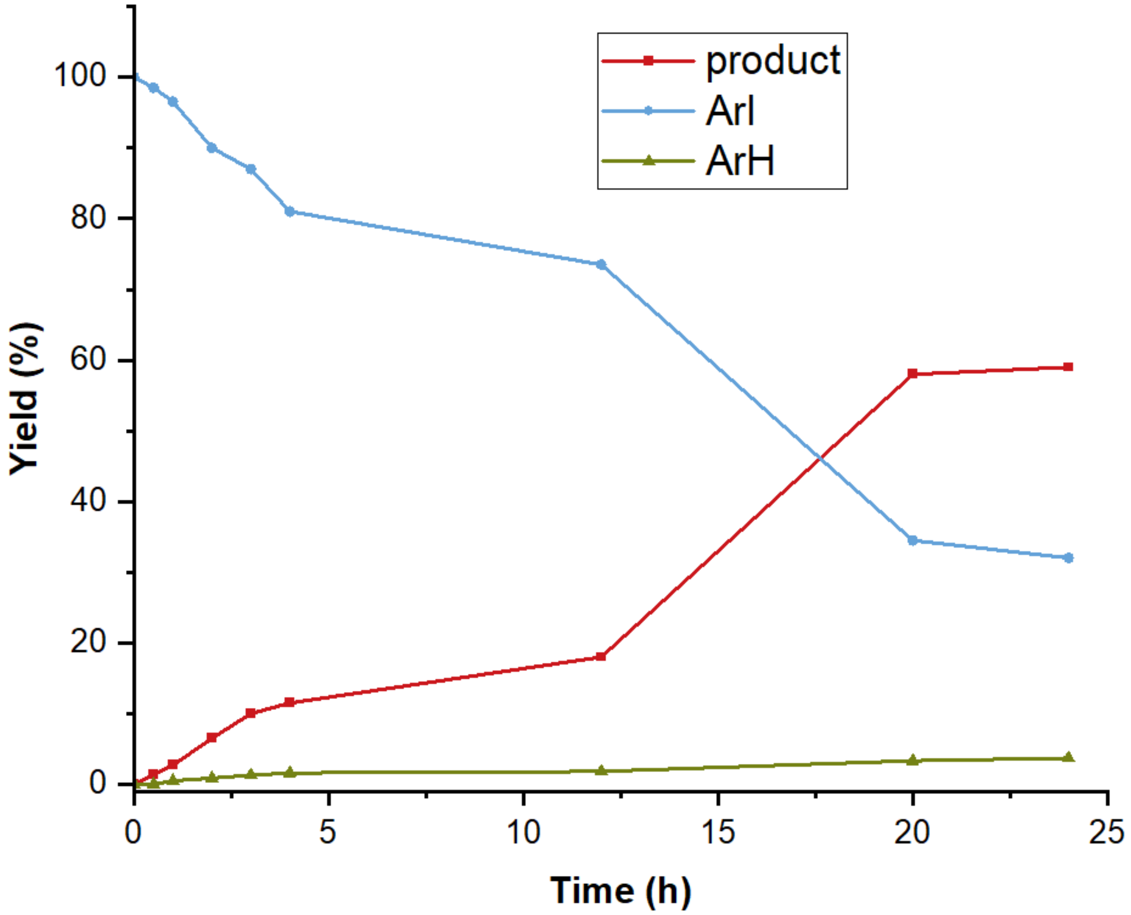

Figure S26. Reaction profile diagram for the coupling of 2,3-dihydro-1H-indene-2-carboxylic acid with 1-chloro-3-iodo-5-(trifluoromethyl)benzene. Yields for desired product and ArI were determined by ^{1}H NMR spectroscopy using 1,3,5-trimethoxybenzene as an internal standard. Yields for ArH were determined by GC/MS analysis using biphenyl as an internal standard.

B. Catalytic Reactions

1. Testing $Fe_3(O)(OAc)_6ClO_4$ as an Fe Catalyst

Table S26. Summary of yields for catalytic reactions using $Fe_3(O)(OAc)_6ClO_4$ as an Fe catalyst[a,b]

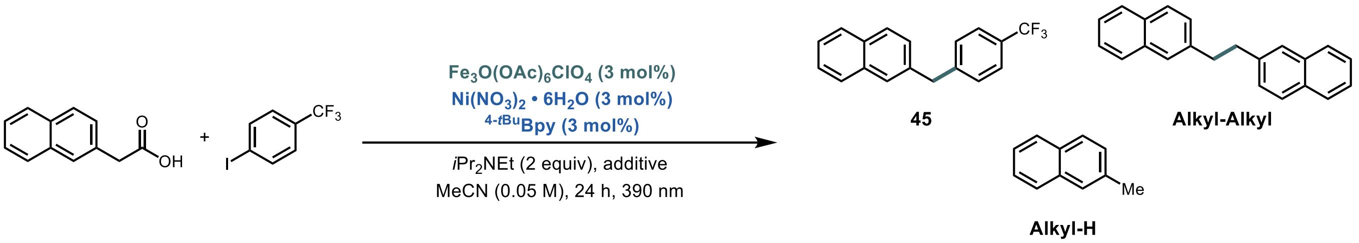

entry	deviation from above	45 (%)	Alkyl-Alkyl (%)	Alkyl-H (%)
1	with 1 equiv TBAI, MeCN	34	18	0
2	with 1 equiv TBAI, 1,4-dioxane	92	25	0
3	with 0.5 equiv phthalimide, MeCN	57	14	6
4	with 0.5 equiv phthalimide, 1,4-dioxane	87	25	0

[a] Reactions were set up following the general procedure for optimization in the decarboxylative arylation (A.2) using 2-(naphthalen-2-yl)acetic acid (0.130 mmol, 1.30 equiv) and 4-iodobenzotrifluoride (0.100 mmol, 1.00 equiv) in 2.00 mL of 1,4-dioxane or MeCN. [b] Yields were determined by ^1H NMR spectroscopy using dibromomethane as an internal standard.

2. Testing $Fe(OAc)_2$ as an Fe Catalyst

Table S27. Summary of yields for catalytic reactions using $Fe(OAc)_2$ as an Fe catalyst[a,b]

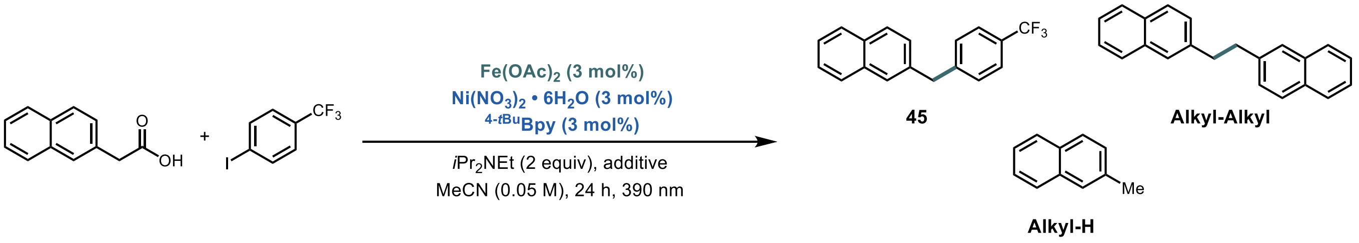

entry	deviation from above	45 (%)	Alkyl-Alkyl (%)	Alkyl-H (%)
1	with 1 equiv TBAI, MeCN	46	7	trace
2	with 1 equiv TBAI, 1,4-dioxane	88	10	0
3	with 0.5 equiv phthalimide, MeCN	23	3	4
4	with 0.5 equiv phthalimide, 1,4-dioxane	83	7	0

[a] Reactions were set up following the general procedure for optimization in the decarboxylative arylation (A.2) using 2-(naphthalen-2-yl)acetic acid (0.130 mmol, 1.30 equiv) and 4-iodobenzotrifluoride (0.100 mmol, 1.00 equiv) in 2.00 mL of 1,4-dioxane or MeCN. [b] Yields were determined by ^1H NMR spectroscopy using dibromomethane as an internal standard.

3. Reaction with Redox Active Ester

Scheme 1. Reaction of 1,3-dioxoisoindolin-2-yl 2,3-dihydro-1H-indene-2-carboxylate following the general procedure for optimization in the decarboxylative arylation (see section IIID).

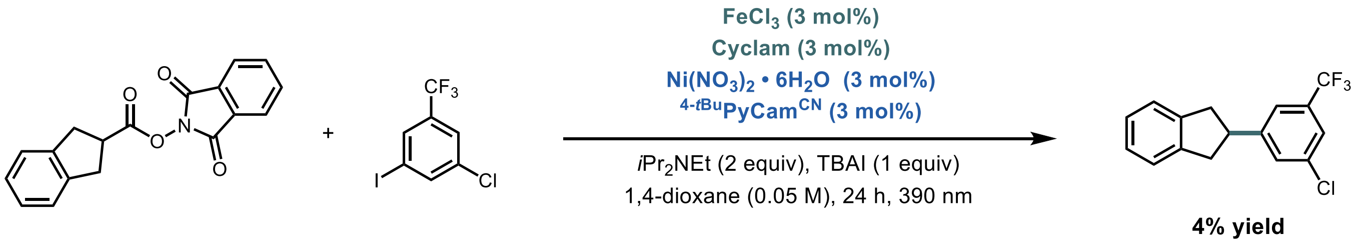

4. Data to Validate Stoichiometric Experiments

Table S28. Summary of yields for catalytic reactions to validate mechanistic experiments[a,b]

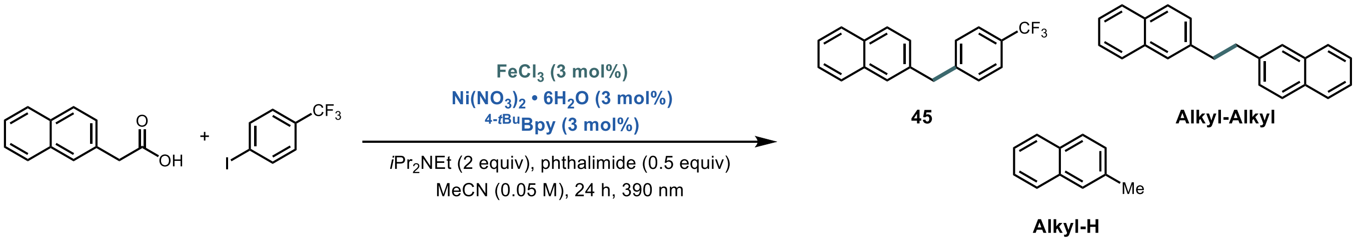

entry	deviation from above	45 (%)	Alkyl-Alkyl (%)	Alkyl-H (%)
1	none	25	0	25
2	with 1 equiv of TBAI	50	18	12
3	1,4-dioxane instead of MeCN	36	34	5
4	with 1 equiv of TBAI, 1,4-dioxane instead of MeCN	52	40	trace

[a] Reactions were set up following the general procedure for optimization in the decarboxylative arylation using 2-(naphthalen-2-yl)acetic acid (0.130 mmol, 1.30 equiv) and 4-iodobenzotrifluoride (0.10 mmol, 1.00 equiv) in 2.00 mL of 1,4-dioxane or MeCN. The Ni/$^{4\text{-}t\text{Bu}}$BPy solution was prepared in the same procedure as the Ni/$^{4\text{-}t\text{Bu}}$PyCam^{CN} solution prior to using in the reaction. [b] Yields were determined by ^{1}H NMR spectroscopy using dibromomethane as an internal standard.

C. Stoichiometric Reactions with Ni Complexes **44** and **46**

1. Reactivity of ($^{4\text{-}t\text{Bu}}$Bpy)Ni(2-naphthenylmethyl)(phthalimido) with Aryl Iodide in the Presence of $FeCl_2$

Table S29. Summary of yields for stoichiometric reactions of ($^{4\text{-}t\text{Bu}}$Bpy)Ni(2-naphthenylmethyl)(phthalimido) with aryl iodide in the presence of $FeCl_2$

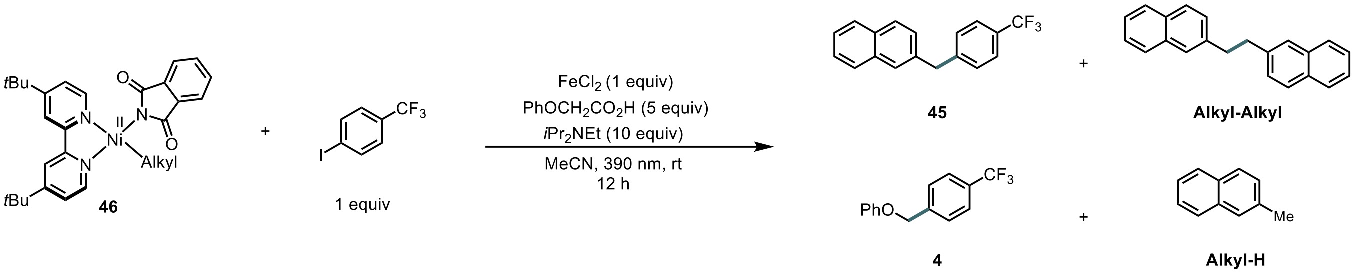

entry	deviation from above	390 nm	45 (%)[a]	4 (%)[a]	ArH (%)[a]	ArI (%)[a]	Alkyl-H (%)[b]	Alkyl-Alkyl(%)[b]
1	none	yes	60	17	11	12	3	34
2	none	no	59	0	13	28	17	31
3	$FeCl_2$ (2 equiv)	yes	51	16	2	31	3	31
4	$FeCl_2$ (2 equiv)	no	58	0	4	31	4	39
5	no $FeCl_2$	yes	29	3	40	0	44	23
6	no $FeCl_2$	no	28	0	11	59	36	25
7	no acid, no *i*Pr_2NEt	yes	58	0	0	38	0	36
8	no acid, no *i*Pr_2NEt	no	54	0	0	43	0	33
9	no acid, no ArI, no $FeCl_2$	yes	-	-	-	-	26	22
10	no acid, no ArI, no $FeCl_2$	no	-	-	-	-	1	32
11	no acid, no ArI	yes	-	-	-	-	5	90
12	no acid, no ArI	no	-	-	-	-	5	84

[a] ^{19}F NMR spectroscopy yields using 4,4'-difluorobiphenyl as an internal standard. [b] GC-FID yields using 4,4'-difluorobiphenyl as an internal standard. The yield of Alkyl-Alkyl was based on **46**.

Reactions were set up in a N_2-filled glovebox. Three separate dry 1-mL volumetric flasks were charged with either $FeCl_2$ (12.2 mg, 0.0750 mmol), *i*Pr_2NEt (96.9 mg, 130.6 μL, 0.750 mmol), or phenoxyacetic acid (57.1 mg, 0.375 mmol). For the flask containing $FeCl_2$, $FeCl_2$ was first added to the volumetric flask, followed by MeCN (0.750 mL), and the mixture was heated to 60 °C until the solid completely dissolved. The flask was then allowed to cool to rt before diluting the solution to the 1 mL mark. To a separate dry 1-mL volumetric flask, 4-iodobenzotrifluoride (20.4 mg, 0.0750 mmol) was added along with the internal standard 4,4'-difluorobiphenyl (14.3 mg, 0.075 mmol, 1.00 equiv). MeCN was added up to the 1 mL mark, then the flask was fitted with a glass stopper and mixed thoroughly. An oven-dried 1-dram vial equipped with a PTFE-coated magnetic stir bar (VWR spinbar micro, 3 × 10 mm, 58948-375) was charged with ($^{4\text{-}t\text{Bu}}$Bpy)Ni(2-naphthalenylmethyl)(phthalimido) **46** (4.6 mg, 0.0075 mmol, 1.00 equiv). Remaining MeCN and aliquots of 4-iodobenzotrifluoride (100 μL, 0.0075 mmol, 1.00 equiv), $FeCl_2$ (100 μL, 0.0075 mmol, 1.00 equiv) *i*Pr_2NEt (100 μL, 0.075 mmol, 10.0 equiv), and

phenoxyacetic acid (100 μL, 0.0375 mmol, 5.00 equiv) stock solutions were added sequentially to give a total volume of 0.500 mL. The vial was sealed with a PTFE-faced silicone septum cap, removed from the glovebox and placed 4 cm away from a 390 nm Kessil lamp and irradiated for 12 h while stirring at 650 rpm and cooling with a stream of air. After 12 h, the reaction mixture was diluted with EtOAc (0.500 mL) and filtered through a pad of silica gel in a pasteur pipette fitted with glass wool and rinsed with EtOAc (0.500 mL). The combined filtrate was analyzed by ^{19}F NMR for fluorinated products using 4,4'-difluorobiphenyl as an internal standard and GC-FID for the remaining side products and byproducts.

2. Reactivity of ($^{4\text{-}tBu}$Bpy)Ni(2-naphthenylmethyl)(phthalimido) with increasing concentration of Aryl Iodide in the Presence of $FeCl_2$

Table S30. Summary of yields for stoichiometric reactions *of* ($^{4\text{-}tBu}$Bpy)Ni(2-naphthenylmethyl)(phthalimido) with increasing concentration of aryl iodide in the presence of $FeCl_2$

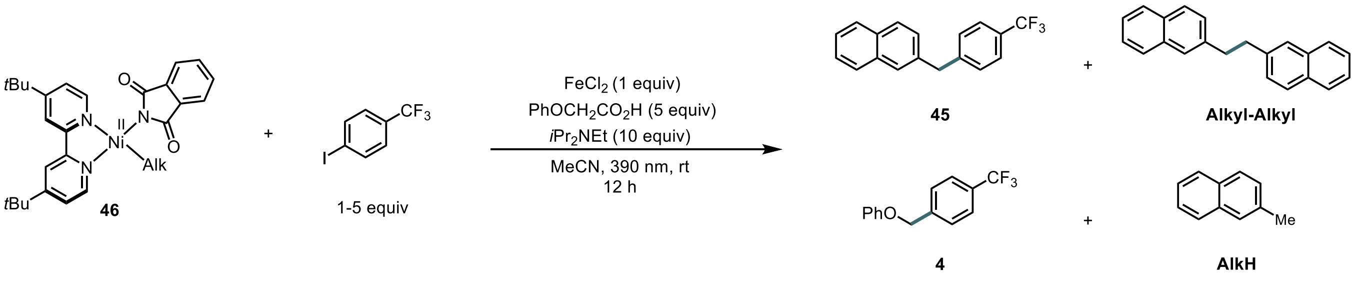

entry	ArI (equiv)	390 nm	45 (%)[a]	4 (%)[a]	ArH (%)[a]	ArI (%)[a]	Alkyl-H (%)[b]	Alkyl-Alkyl (%)[b]
1	1	yes	60	17	11	12	3	34
2	1	no	59	0	13	28	17	31
3	2	yes	59	29	7	35	0	36
4	2	no	56	0	7	68	4	39
5	3	yes	53	27	5	50	1	35
6	3	no	50	0	6	78	0	42
7	5	yes	49	19	4	67	35	25
8	5	no	48	0	3	86	4	39

[a] ^{19}F NMR spectroscopy yields using 4,4'-difluorobiphenyl as an internal standard. [b] GC-FID yields using 4,4'-difluorobiphenyl as an internal standard. The yield of Alkyl-Alkyl was based on **46.**

Reactions were set up in a N_2-filled glovebox. Two separate dry 1-mL volumetric flasks were charged with either $FeCl_2$ (9.5 mg, 0.075 mmol, 1.00 equiv) or 4-iodobenzotrifluoride (20.4 mg, 0.075 mmol). MeCN was added up to the mark, then the flasks were fitted with a glass stopper and mixed thoroughly. For the flask containing $FeCl_2$, $FeCl_2$ was first added to the volumetric flask, followed by MeCN (0.750 mL), and the mixture was heated to 60 °C until the solid completely dissolved. The flask was then allowed to cool to rt before diluting the solution to the 1 mL mark. To a separate dry 1-mL volumetric flask, phenoxyacetic acid (57.1 mg, 0.375 mmol, 5.00 equiv) was added along with the internal standard 4,4'-difluorobiphenyl (14.3 mg, 0.075 mmol, 1.00 equiv). MeCN (0.750 mL) was added to this vial,

followed by *i*Pr$_2$NEt (130.6 μL, 0.75 mmol, 10.0 equiv), and the contents of the flask were mixed followed by filling the flask to the mark, fitting the flask with a glass stopper, and mixing thoroughly. An oven-dried 1 dram vial equipped with a PTFE-coated stir bar (VWR spinbar micro, 3 × 10 mm, 58948-375) was charged with ($^{4\text{-}t\text{Bu}}$Bpy)Ni(2-naphthalenylmethyl)(phthalimido) **46** (4.6 mg, 0.0075 mmol, 1.00 equiv). Remaining MeCN and aliquots of 4-iodobenzotrifluoride (100 μL, 0.0075 mmol, 1.00 equiv), FeCl$_2$ (100 μL, 0.0075 mmol, 1.00 equiv), *i*Pr$_2$NEt (100 μL, 0.075 mmol, 10.0 equiv), and phenoxyacetic acid (100 μL, 0.0375 mmol, 5.00 equiv) stock solutions were added sequentially to give a total volume of 0.500 mL. The vials were sealed with a PTFE-faced silicone septum cap, removed from the glovebox and placed 4 cm away from a 390 nm Kessil lamp and irradiated for 12 h while stirring at 650 rpm and cooling with a stream of air. After 12 h, the reaction mixture was diluted with EtOAc (0.500 mL) and filtered through a pad of silica gel in a pasteur pipette fitted with glass wool and rinsed with EtOAc (0.500 mL). The combined filtrate was analyzed by ^{19}F NMR for fluorinated products using 4,4'-difluorobiphenyl as an internal standard and GC-FID for the remaining side products and byproducts.

3. Reactivity of ($^{4\text{-}t\text{Bu}}$Bpy)Ni(2-naphthenylmethyl)(phthalimido) with Aryl Iodide in the Presence of FeCl$_3$

Table S31. Summary of yields for stoichiometric reactions *of* ($^{4\text{-}t\text{Bu}}$Bpy)Ni(2-naphthenylmethyl)(phthalimido) with aryl iodide in the presence of FeCl$_3$

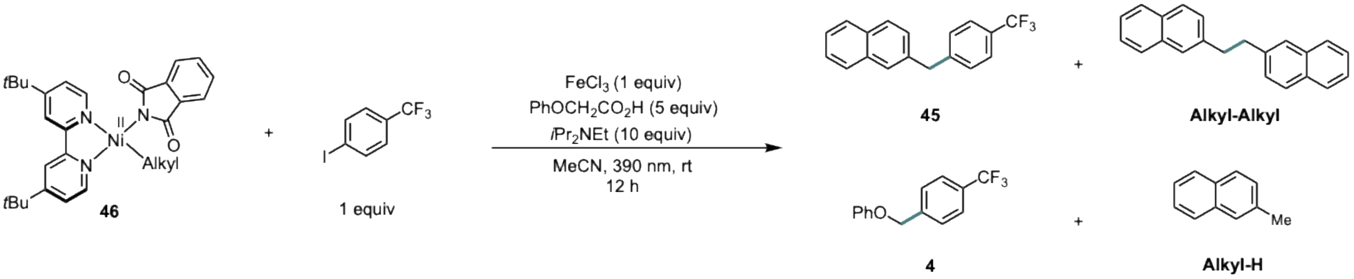

entry	deviation from above	390 nm	45 (%)[a]	4 (%)[a]	ArH (%)[a]	ArI (%)[a]	Alkyl-H (%)[b]	Alkyl-Alkyl (%)[b]
1	none	yes	10	54	30	0	1	71
2	none	no	10	0	0	85	1	71
3	no acid, no *i*Pr$_2$NEt	yes	10	0	0	87	0	78
4	no acid, no *i*Pr$_2$NEt	no	7	0	0	91	1	68

[a] ^{19}F NMR spectroscopy yields using 4,4'-difluorobiphenyl as an internal standard. [b] GC-FID yields using 4,4'-difluorobiphenyl as an internal standard. The yield of Alkyl-Alkyl was based on **46.**

Reactions were set up in a N$_2$-filled glovebox. Three separate dry 1-mL volumetric flasks were charged with either FeCl$_3$ (12.2 mg, 0.075 mmol), *i*Pr$_2$NEt (96.9 mg, 130.6 μL, 0.75 mmol), or phenoxyacetic acid (57.1 mg, 0.375 mmol). MeCN was added up to the 1 mL mark, then the flasks were fitted with a glass stopper and mixed thoroughly. To a separate dry 1-mL volumetric flask, 4-iodobenzotrifluoride

(20.4 mg, 0.075 mmol) along with the internal standard 4,4'-difluorobiphenyl (14.3 mg, 0.075 mmol). MeCN was added up to the 1 mL mark, then the flask was fitted with a glass stopper and mixed thoroughly. An oven-dried 1-dram vial equipped with a PTFE-coated magnetic stir bar (VWR spinbar micro, 3 × 10 mm, 58948-375) was charged with ($^{4\text{-}t\text{Bu}}$Bpy)Ni(2-naphthalenylmethyl)(phthalimido) **46** (4.6 mg, 0.0075 mmol, 1.00 equiv). Remaining MeCN and stock solutions of 4-iodobenzotrifluoride (100 μL, 0.00750 mmol, 1.00 equiv), $FeCl_3$ (100 μL, 0.00750 mmol, 1.00 equiv), *i*Pr_2NEt (100 μL, 0.0750 mmol, 10.0 equiv), and phenoxyacetic acid (100 μL, 0.0375 mmol, 5.00 equiv) were added sequentially to give a total volume of 0.500 mL. The vials were sealed with a PTFE-faced silicone septum cap, removed from the glovebox and placed 4 cm away from a 390 nm Kessil lamp and irradiated for 12 h while stirring at 650 rpm and cooling with a stream of air. After 12 h, the reaction mixture was diluted with EtOAc (0.500 mL) and filtered through a pad of silica gel in a pasteur pipette fitted with glass wool and rinsed with EtOAc (0.500 mL). The combined filtrate was analyzed by ^{19}F NMR for fluorinated products using 4,4'-difluorobiphenyl as an internal standard and GC-FID for the remaining side products and byproducts.

4. Reduction of ($^{4\text{-}t\text{Bu}}$Bpy)Ni(2-naphthenylmethyl)(phthalimido) with Decamethylcobaltacene

Table S32. Summary of yields for stoichiometric reactions *of* ($^{4\text{-}t\text{Bu}}$Bpy)Ni(2-naphthenylmethyl)(phthalimido) using decamethylcobaltacene as a reductant

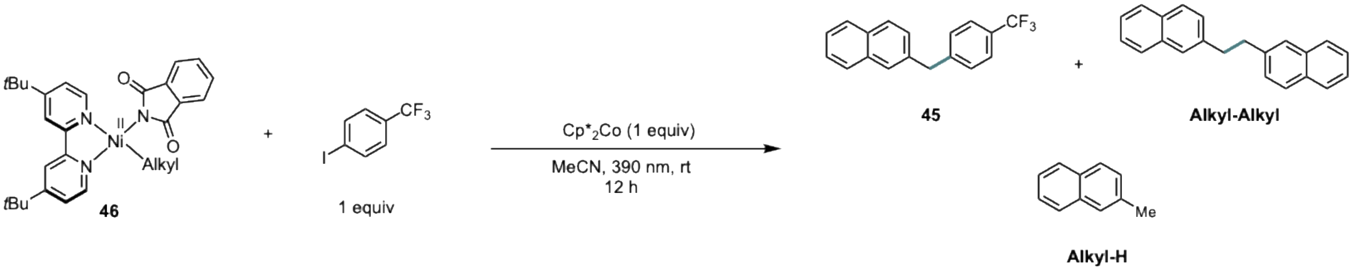

entry	deviation from above	390 nm	45 (%)[a]	ArH (%)[a]	ArI (%)[a]	Alkyl-H (%)[b]	Alkyl-Alkyl (%)[b]
1	none	yes	72	6	0	5	0
2	none	no	62	6	0	12	0
3	no reductant	yes	44	0	46	0	47
4	no reductant	no	48	0	35	0	47

[a] ^{19}F NMR spectroscopy yields using 4,4'-difluorobiphenyl as an internal standard. [b] GC-FID yields using 4,4'-difluorobiphenyl as an internal standard. The yield of Alkyl-Alkyl was based on **46.**

Reactions were set up in a N_2-filled glovebox. A dry 5 mL volumetric flask was charged with 4-iodobenzotrifluoride (20.4 mg, 0.0750 mmol, 1.00 equiv) and the internal standard 4,4'-difluorobiphenyl (14.3 mg, 0.0750 mmol, 1.00 equiv). MeCN was added up the mark, then the flask was fitted with a glass stopper and mixed thoroughly. An oven-dried 1-dram vial equipped with a PTFE-coated magnetic stir bar (VWR spinbar micro, 3 × 10 mm, 58948-375) was charged with ($^{4\text{-}}$

^{tBu}Bpy)Ni(2-naphthalenylmethyl)(phthalimido) **46** (4.6 mg, 0.00750 mmol, 1.00 equiv), decamethylcobaltacene (2.5 mg, 0.00750 mmol, 1.00 equiv), and a 0.500 mL aliquot of the 4-iodobenzotrifluoride solution. The vials were sealed with a PTFE-faced silicone septum cap, removed from the glovebox and placed 4 cm away from a 390 nm Kessil lamp and irradiated for 12 h while stirring at 650 rpm and cooling with a stream of air. After 12 h, the reaction mixture was diluted with EtOAc (0.500 mL) and filtered through a pad of silica gel in a pasteur pipette fitted with glass wool and rinsed with EtOAc (0.500 mL). The combined filtrate was analyzed by ^{19}F NMR for fluorinated products using 4,4'-difluorobiphenyl as an internal standard and GC-FID for the remaining side products and byproducts.

5. Reactivity of ($^{4\text{-}tBu}$Bpy)Ni(4-trifluoromethyl phenyl)(phthalimido) in the Presence of $FeCl_2$

Table S33. Summary of yields for stoichiometric reactions *of* ($^{4\text{-}tBu}$Bpy)Ni(4-trifluoromethyl phenyl)(phthalimido) in the presence of $FeCl_2$ and alkyl carboxylic acids

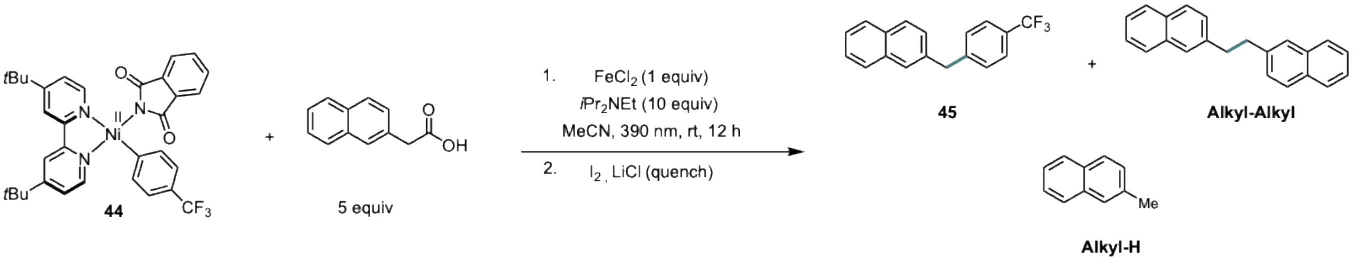

entry	390 nm	45 (%)[a]	ArH (%)[a]	ArI (%)[a]	Alkyl-H (%)[b]	Alkyl-Alkyl (%)[b]
1	yes	0	101	0	1	1
2	no	0	108	0	0	0

[a] ^{19}F NMR spectroscopy yields using 4,4'-difluorobiphenyl as an internal standard. [b] GC-FID yields using 4,4'-difluorobiphenyl as an internal standard. The yield of Alkyl-Alkyl was based on 5 equiv of the carboxylic acid with theoretical maximum yield of 100%.

Reactions were set up in a N_2-filled glovebox. Two separate dry 1-mL volumetric flasks were charged with either $FeCl_2$ (12.2 mg, 0.075 mmol) or 2-naphthaleneacetic acid (69.8 mg, 0.375 mmol). MeCN was added up to the mark, then the flasks were fitted with a glass stopper and mixed thoroughly. For the flask containing $FeCl_2$, $FeCl_2$ was first added to the volumetric flask, followed by MeCN (0.750 mL), and the mixture was heated to 60 °C until the solid completely dissolved. The flask was then allowed to cool to rt before diluting the solution to the 1 mL mark. To a separate dry 1-mL volumetric flask, $i Pr_2NEt$ (96.9 mg, 130.6 μL, 0.750 mmol) was added along with the internal standard 4,4'-difluorobiphenyl (14.3 mg, 0.075 mmol). MeCN was added up to the 1 mL mark, then the flask was fitted with a glass stopper and mixed thoroughly. An oven-dried 1 dram vial equipped with a PTFE-coated stir bar (VWR spinbar micro, 3 × 10 mm, 58948-375) was charged with ($^{4\text{-}tBu}$Bpy)Ni(4-trifluoromethylphenyl)(phthalimido) **44** (4.6 mg, 0.0075 mmol, 1.00 equiv). Remaining MeCN and stock solutions of $FeCl_2$ (100 μL, 0.00750 mmol, 1.00 equiv), $i Pr_2NEt$ (100 μL, 0.0750 mmol, 10.0 equiv), and 2-naphthaleneacetic acid (100 μL, 0.0375 mmol, 5.00 equiv) were added sequentially to give a total

volume of 0.500 mL. The vials were sealed with a PTFE-faced silicone septum cap, removed from the glovebox and placed 4 cm away from a 390 nm Kessil lamp and irradiated for 12 h while stirring at 650 rpm and cooling with a stream of air. After 12 h, the vial was brought back into the glovebox, and the reaction was quenched with 0.500 mL of 0.250 M I_2/0.500 M LiCl solution in THF. The reaction mixture was filtered through a pad of silica gel in a pasteur pipette fitted with glass wool and rinsed with EtOAc (0.500 mL). The combined filtrate was analyzed by ^{19}F NMR for fluorinated products using 4,4'-difluorobiphenyl as an internal standard and GC-FID for the remaining side products and byproducts.

6. Reactivity of ($^{4\text{-}t\text{Bu}}$Bpy)Ni(4-trifluoromethyl phenyl)(phthalimido) in the Presence of $FeCl_3$

Table S34. Summary of yields for stoichiometric reactions *of* ($^{4\text{-}t\text{Bu}}$Bpy)Ni(4-trifluoromethyl phenyl)(phthalimido) in the presence of $FeCl_3$ with alkyl carboxylic acids

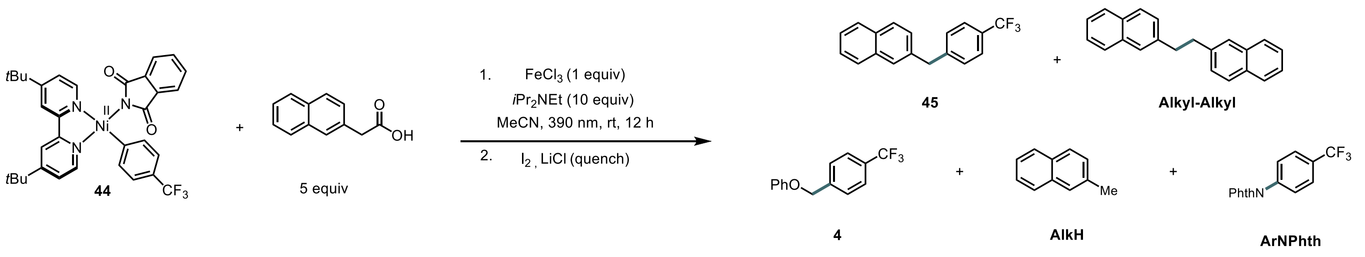

entry	deviation from above	390 nm	45 (%)[a]	ArH (%)[a]	ArI (%)[a]	ArI-NPhth (%)[a]	Alkyl-H (%)[b]	Alkyl-Alkyl (%)[b]
1	none	yes	2	75	0	1	1	15
2	none	no	0	90	0	2	0	0
3	with KNPhth (5 equiv)	yes	13	72	0	6	1	9
4	with KNPhth (5 equiv	no	0	90	0	7	0	0

[a] ^{19}F NMR spectroscopy yields using 4,4'-difluorobiphenyl as an internal standard. [b] GC-FID yields using 4,4'-difluorobiphenyl as an internal standard. The yield of Alkyl-Alkyl was based on 5 equiv of the carboxylic acid with theoretical maximum yield of 100%.

Reactions were set up in a N_2-filled glovebox. Two separate dry 1 mL volumetric flasks were charged with either $FeCl_3$ (12.2 mg, 0.075 mmol) or 2-naphthaleneacetic acid (69.8 mg, 0.375 mmol). MeCN was added up to the mark, then the flasks were fitted with a glass stopper and mixed thoroughly. To a separate dry 1 mL volumetric flask, *i*Pr_2NEt (96.9 mg, 130.6 µL, 0.750 mmol) along with 4,4'-difluorobiphenyl (14.3 mg, 0.075 mmol) was added. MeCN was added up to the 1 mL mark, then the flask was fitted with a glass stopper and mixed thoroughly. An oven-dried 1- dram vial equipped with a PTFE-coated magnetic stir bar (VWR spinbar micro, 3 × 10 mm, 58948-375) was charged with ($^{4\text{-}t\text{Bu}}$Bpy)Ni(4-trifluoromethylphenyl)(phthalimido) **44** (4.6 mg, 0.00750 mmol, 1.00 equiv) and potassium phthalimide (6.90 mg, 0.0375 mmol, 5.00 equiv, entries 3 and 4). Remaining MeCN and stock solutions of $FeCl_3$ (100 µL, 0.0075 mmol, 1.00 equiv), *i*Pr_2NEt (100 µL, 0.075 mmol, 10.0 equiv), and 2-naphthaleneacetic acid (100 µL, 0.0375 mmol, 5.00 equiv) were added sequentially to give a total volume of 0.500 mL. The vials were sealed with a PTFE-faced silicone septum cap, removed from the glovebox and placed 4 cm away from a 390 nm Kessil lamp and irradiated for 12 h while stirring at 650

rpm and cooling with a stream of air. After 12 h, the vial was brought back into the glovebox, and the reaction was quenched with 0.500 mL of 0.250 M I_2/0.500 M LiCl solution in THF. The reaction mixture was filtered through a pad of silica gel in a pasteur pipette fitted with glass wool and rinsed with EtOAc (0.500 mL). The combined filtrate was analyzed by ^{19}F NMR for fluorinated products using 4,4'-difluorobiphenyl as an internal standard and GC-FID for the remaining side products and byproducts.

7. Stability of ($^{4\text{-}t\text{Bu}}$Bpy)Ni(4-trifluoromethyl phenyl)(phthalimido)

Table S35. Summary of yields for stoichiometric reactions probing protodehalogenation of ($^{4\text{-}t\text{Bu}}$Bpy)Ni(4-trifluoromethyl phenyl)(phthalimido)

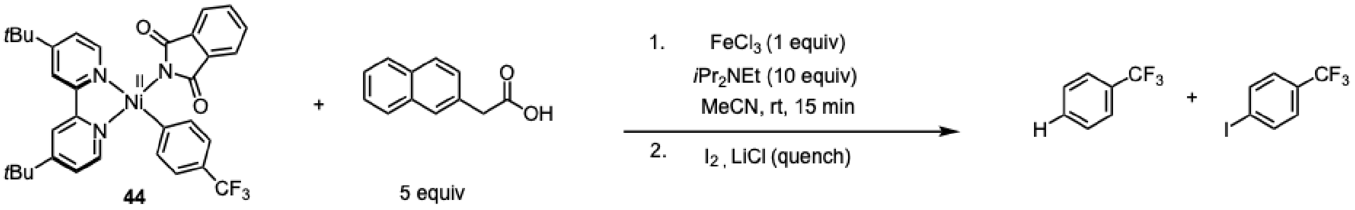

entry	deviation from above	ArH (%)	ArI (%)
1	none	99	0
2	$FeCl_2$ instead of $FeCl_3$	99	0
3	with KNPhth (5 equiv)	72	5
4	no $FeCl_3$	86	0

Yields were determined by ^{19}F NMR spectroscopy using 4,4'-difluorobiphenyl as an internal standard.

Reactions were set up in a N_2-filled glovebox. Two separate dry 5 mL volumetric flasks were charged with $FeCl_2$ (19.0 mg, 0.150 mmol) or $FeCl_3$ (24.3 mg, 0.150 mmol). MeCN was added up to the mark, then the flasks were fitted with a glass stopper and mixed thoroughly. For $FeCl_2$, MeCN (4.00 mL) was added, and the mixture heated to 60 ºC until the solid completely dissolved. After allowing the flask to cool to rt, MeCN was added to the 5 mL mark. A separate dry 5 mL volumetric flask was charged with 2-naphthaleneacetic acid (139.7 mg, 0.750 mmol, 1.00 equiv) and the internal standard 4,4'-difluorobiphenyl (28.5 mg, 0.150 mmol, 1.00 equiv). MeCN was added to the 5 mL mark, followed by *i*Pr_2NEt (261 µL, 1.50 mmol, 10.0 equiv), and the contents of the flask were mixed followed by filling the flask to the mark, fitting the flask with a glass stopper, and mixing thoroughly. An oven-dried 1 dram vial equipped with a PTFE-coated stir bar (VWR spinbar micro, 3 × 10 mm, 58948-375) was charged with ($^{4\text{-}t\text{Bu}}$Bpy)Ni(4-trifluoromethylphenyl)(phthalimido) **44** (4.60 mg, 0.0075 mmol, 1.00 equiv) and potassium phthalimide (6.90 mg, 0.0375 mmol, 5.00 equiv, entry 3). Remaining MeCN and 0.250 mL aliquots of the $FeCl_2$ (0.00750 mmol, 1.00 equiv) or $FeCl_3$ (0.00750 mmol, 1.00 equiv) stock solutions and of the carboxylic acid/base stock solution containing *i*Pr_2NEt (0.0750 mmol, 10.0 equiv),

2-naphthaleneacetic acid (0.0375 mmol, 5.00 equiv), and 4,4'-difluorobiphenyl (0.00750 mmol, 1.00 equiv) were added sequentially to give a total volume of 0.500 mL. The vial was sealed with a PTFE-faced silicone septum cap and placed on a stir plate (650 rpm). After 15 min, the reaction was quenched with 0.500 mL of 0.250 M I_2/0.500 M LiCl solution in THF. The reaction mixture was filtered through a pad of silica gel in a pasteur pipette fitted with glass wool and rinsed with EtOAc (0.500 mL). The combined filtrate was analyzed by ^{19}F NMR for fluorinated products using 4,4'-difluorobiphenyl as an internal standard.

8. Stability of ($^{4\text{-}tBu}$Bpy)Ni(2-naphthylenylmethyl)(phthalimido)

Table S36. Summary of yields for stoichiometric reactions probing protodehalogenation of ($^{4\text{-}tBu}$Bpy)Ni(2-naphthalenylmethyl)(phthalimido)

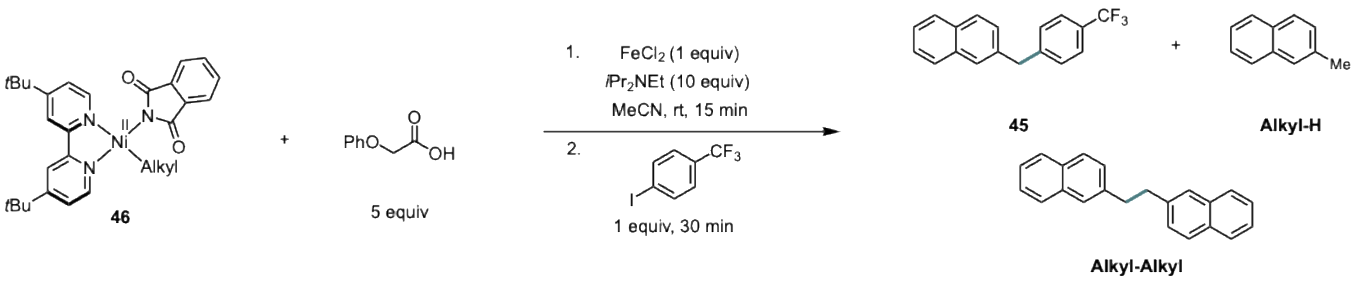

entry	deviation from above	45 (%)[a]	ArI (%)[a]	Alkyl-H (%)[b]	Alkyl-Alkyl (%)[b]
1	none	0	91	90	0
2	no $FeCl_2$	1	92	93	0

[a] ^{19}F NMR spectroscopy yields using 4,4'-difluorobiphenyl as an internal standard. [b] GC-FID yields using 4,4'-difluorobiphenyl as an internal standard. The yield of Alkyl-Alkyl was based on **46.**

Reactions were set up in a N_2-filled glovebox. A dry 5 mL volumetric flask was charged $FeCl_2$ (19.0 mg, 0.150 mmol). MeCN (4.00 mL) was added and the mixture heated to 60 ºC until the solid completely dissolved. The flask was cooled to rt before diluting the solution to the mark, fitting the flask with a glass stopper, and mixing thoroughly. A separate dry 5 mL volumetric flask was charged with phenoxyacetic acid (114 mg, 0.750 mmol, 5.00 equiv) and the internal standard 4,4'-difluorobiphenyl (28.5 mg, 0.150 mmol, 1.00 equiv). MeCN (4.00 mL) was added, followed by *i*Pr_2NEt (261.3 µL, 1.50 mmol, 10.0 equiv), and the contents of the flask were mixed followed by filling the flask to the mark, fitting the flask with a glass stopper, and mixing thoroughly. A dry 1 mL volumetric flask was charged with 4-iodobenzotrifluoride (20.4 mg, 0.0750 mmol). MeCN was added up to the 1 mL mark before fitting the flask with a glass stopper and mixing thoroughly. An oven-dried 1-dram vial equipped with a PTFE-coated stir bar (VWR spinbar micro, 3 × 10 mm, 58948-375) was charged with ($^{4\text{-}tBu}$Bpy)Ni(2-naphthalenylmethyl)(phthalimido) **46** (4.6 mg, 0.0075 mmol, 1.0 equiv). Remaining MeCN, 0.250 mL aliquots of the $FeCl_2$ (0.00750 mmol, 1.00 equiv) stock solution, and the carboxylic acid/base stock

solution containing $i\text{Pr}_2\text{NEt}$ (0.075 mmol, 10.0 equiv), phenoxyacetic acid (0.0375 mmol, 5.00 equiv), and 4,4'-difluorobiphenyl (0.00750 mmol, 1.00 equiv) were added sequentially to give a total volume of 0.500 mL. The vials were sealed with a PTFE-faced silicone septum cap and stirred for 15 min at 650 rpm. After 15 min, the reaction mixture was charged with an aliquot (0.100 mL) of the 4-iodobenzotrifluoride (0.00750 mmol, 1.00 equiv) stock solution, and the resulting mixture was stirred for an additional 30 min. The reaction mixture was filtered through a pad of silica gel in a pasteur pipette fitted with glass wool and rinsed with EtOAc (0.500 mL). The combined filtrate was analyzed by ^{19}F NMR for fluorinated products using 4,4'-difluorobiphenyl as an internal standard and GC-FID for the remaining side products and byproducts.

D. Cyclic Voltammetry and Differential Pulse Voltammetry Experiments

Cyclic voltammetry (CV) and differential pulse voltammetry (DPV) measurements were performed in a N_2-filled glovebox at 32 ºC. The Ag/$AgNO_3$ reference electrode was filled with 10 mM $AgNO_3$, 100 mM $TBAPF_6$ in MeCN. A glassy carbon working electrode and a platinum wire counter electrode were used. Redox potentials are referenced to the $E_{1/2}$ of a ferrocene standard (10 mM ferrocene, 100 mM $TBAPF_6$ in MeCN) analyzed prior to other measurements.

1. CV of ($^{4\text{-}t\text{Bu}}$Bpy)Ni(2-naphthylenylmethyl)(phthalimido)

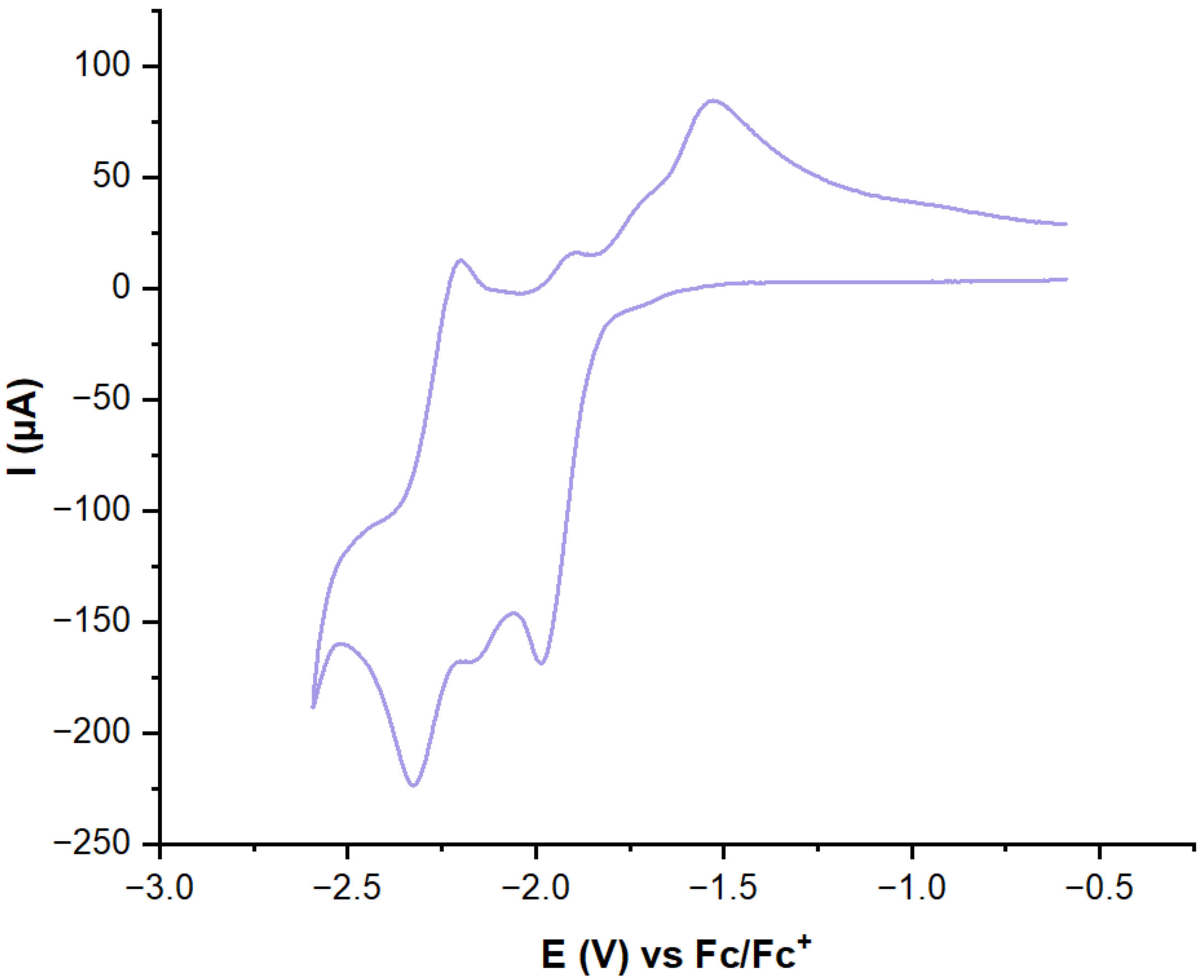

Figure S27: CV of ($^{4\text{-}t\text{Bu}}$Bpy)Ni(2-naphthylenylmethyl)(phthalimido) (10 mM), 100 mM $TBAPF_6$ in MeCN. Scan rate of 100 mV/s.

In the CV of complex **46**, the first reduction wave is assigned to Ni(II)/Ni(I) and appeared electrochemically irreversible. The reduction potential was measured at Ni(II)/Ni(I) = -1.94 V vs Fc/Fc$^+$ using DPV.

2. DPV of ($^{4\text{-}t\text{Bu}}$Bpy)Ni(2-naphthylenylmethyl)(phthalimido)

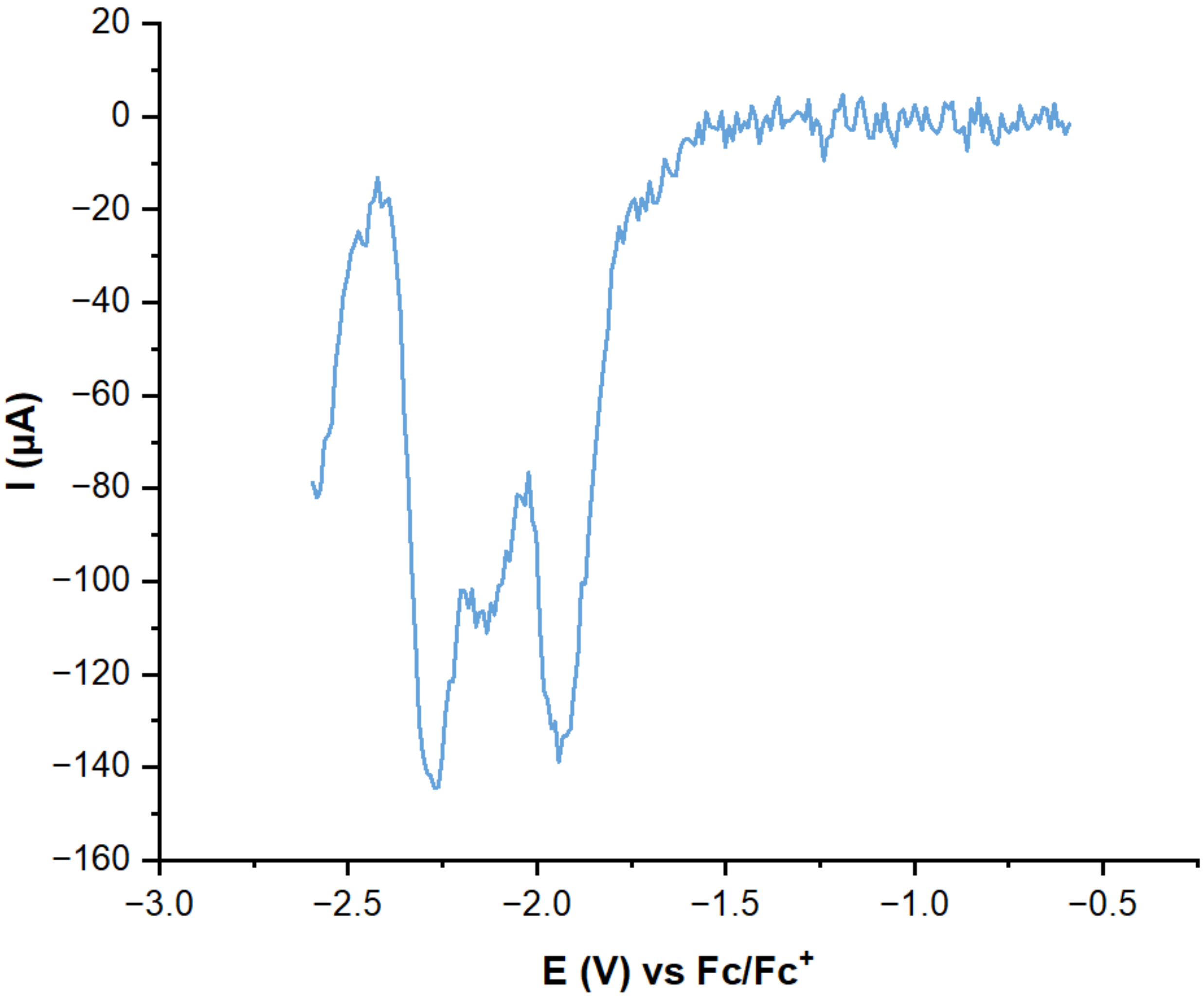

Figure S28: DPV of ($^{4\text{-}t\text{Bu}}$Bpy)Ni(2-naphthylenylmethyl)(phthalimido) (10 mM), 100 mM $TBAPF_6$ in MeCN.

IX. Crystallographic Experiments

A. Data Collection

*i. Compound **13***

Complex (**13**) was characterized by X-ray crystallography. A colorless plate-shaped-shaped crystal with dimensions 0.40 × 0.11 × 0.03 mm^3 was mounted. Data were collected using a XtaLAB Synergy, Dualflex, and HyPix diffractometer equipped with an Oxford Cryosystems low-temperature device operating at T = 100.03(11) K. The structure was solved with the ShelXT 2018/2[8], solution program using dual methods and by using Olex2 1.5-alpha[9] as the graphical interface. The model was refined with olex2. refine 1.5-alpha[10] using full matrix least squares minimization on $\boldsymbol{F}^2$.

Data were measured using w scans with Mo K_a radiation. The diffraction pattern was indexed and the total number of runs and images was based on the strategy calculation from the program CrysAlisPro system (CCD 43.92a 64-bit (release 05-10-2023)). The maximum resolution that was achieved was Q = 29.20° (0.73 Å). The unit cell was refined using CrysAlisPro 1.171.43.103a (Rigaku OD, 2023) on 8389 reflections, 53% of the observed reflections. Data reduction, scaling and absorption corrections were performed using CrysAlisPro 1.171.43.103a.[11] The final completeness is 99.36 % out to 29.20° in Q. A numerical absorption correction based on gaussian integration over a multifaceted crystal model was performed using CrysAlisPro 1.171.42.74a.[12] An empirical absorption correction using spherical harmonics, implemented in SCALE3 ABSPACK scaling algorithm was also applied. The absorption coefficient m of this material is 0.248 mm^{-1} at this wavelength (l = 0.71073Å) and the minimum and maximum transmissions are 0.688 and 1.000.

The crystal structure is polar, and the molecule itself exhibits chirality despite the absence of chiral centers at individual atoms, the two molecules are non-superimposable on their mirror images. However, they can be brought coincident by rotation about the bond C2-C9. This rotation results in two rotamers (these are two rotamers: and each molecule is chiral). Collectively they are non-chiral: it crystallizes as an inversion-related pair of molecules. Even though the crystal structure is non-chiral, it is still polar. This is a notable deviation from most other crystal structures where inversion partners typically exhibit symmetry operations. The breaking of symmetry, which gives rise

[8] Sheldrick, G. M. *Acta Cryst.* A, **2015**, *71*, 3-8.
[9] Dolomanov, O. V.; Bourhis, L. J.; Gildea, R. J.; Howard, J. A. K.; Puschmann, H. *J. Appl. Crystallogr.* **2009**, *42*, 339-341.
[10] Bourhis, L. J.; Dolomanov, O. V.; Gildea, R. J.; Howard, J. A. K.; Puschmann, H. *Acta Cryst.* A, **2015**, *71*, 59-71.
[11] CrysAlisPro Software System, Rigaku Oxford Diffraction, (2023).
[12] CrysAlisPro (ROD), Rigaku Oxford Diffraction, Poland (2022).

to the polar axis, is intriguing. It appears that a 2-fold rotation around C2-C9 effectively acts as an inversion through a center of inversion. The twinning and this pseudo-centrosymmetry are closely related to each other. The twin law corresponds to a 2-fold rotation along the c-axis within the crystal.

*ii. Compound **46***

Complex (**46**) was characterized by X-ray crystallography. A red crystal with approximate dimensions 0.12 × 0.1 × 0.05 mm^3 was selected under oil under ambient conditions and attached to the tip of a MiTeGen MicroMount©. The crystal was mounted in a stream of cold N_2 at 100(1) K and centered in the X-ray beam by using a video camera.

The crystal evaluation and data collection were performed on a Bruker D8 VENTURE PhotonIII four-circle diffractometer with Cu Kα (λ = 1.54178 Å) radiation and the detector to crystal distance of 5.0 cm.[13] The initial cell constants were obtained from a 180° φ scan conducted at a 2θ = 50° angle with the exposure time of 5 second per frame. The reflections were successfully indexed by an automated indexing routine built in the APEX5 program. The final cell constants were calculated from a set of 9920 strong reflections from the actual data collection. The data were collected by using the full sphere data collection routine to survey the reciprocal space to the extent of a full sphere to a resolution of 0.79 Å. A total of 64028 data were harvested by collecting 22 sets of frames with 0.9º scans in ω and φ with an exposure time 2–20 sec per frame. These highly redundant datasets were corrected for Lorentz and polarization effects. The absorption correction was based on fitting a function to the empirical transmission surface as sampled by multiple equivalent measurements.[14]

B. Structure Solution and Refinement

*i. Compound **13***

The structure was solved and the space group *Pc* (# 7) determined by the ShelXT 2018/2 (Sheldrick, 2018) structure solution program using using dual methods and refined by full matrix least squares minimisation on $\boldsymbol{F^2}$ using version of olex2.refine 1.5-alpha (Bourhis et al., 2015). All atoms were refined anisotropically. Hydrogen atom positions positions were refined using the Hirshfeld model. Refinement was by using NoSpherA2, an implementation of non-spherical atom-form-factors.[15] NoSpherA2 implementation of HAR makes use of tailor-made aspherical atomic form factors

[13] Bruker AXS LLC (2023). *APEX5.* Version 2023.9-RC115. Madison, Wisconsin, USA.

[14] Krause, L.; Herbst-Irmer, R.; Sheldrick, G. M.; Stalke, D. *J. Appl. Cryst.* **2015**, *48*, 3-10.

[15] Kleemiss, F.; Dolomanov, O. V.; Bodensteiner, M.; Peyerimhoff, N.; Midgley. L.; Bourhis, L. J.; Genoni, A.; Malaspina, L. A.; Jayatilaka, D.; Spencer, J. L.; White, F., Grundkötter-Stock, B.; Steinhauer, S.; Lentz, D.; Puschmann, H.; Grabowsky, S. *Chem. Sci.* **2021**, *12*, 1675-1692.

calculated from a Hirshfeld-partitioned electron density (ED) not from spherical-atom form factors. The ED was calculated from a Gaussian basis set single determinant SCF wavefunction from DFT using selected functionals for a fragment of this crystal. This fragment was embedded in an electrostatic crystal field by employing cluster charges. The following options were used: SOFTWARE: ORCA PARTITIONING: NoSpherA2 INT ACCURACY: Normal METHOD: PBE BASIS SET: def2-TZVP CHARGE: 0 MULTIPLICITY: 1 DATE: 2024-02-18_16-11-11. The value of Z' is 2. This means that there are two independent molecules in the asymmetric unit. The moiety formula is $C_{15}H_{12}OS$.

The Flack parameter was refined to -0.00(4). Determination of absolute structure using Bayesian statistics on Bijvoet differences using the Olex2 results in -0.00(4). This structure is in chiral space group, but there are no chiral atoms. Note: The Flack parameter is used to determine chirality of the crystal studied, the value should be near 0, a value of 1 means that the stereochemistry is wrong and the model should be inverted. A value of 0.5 means that the crystal consists of a racemic mixture of the two enantiomers.

Crystal Data for $C_{15}H_{12}OS$, M_r = 240.328, monoclinic, *Pc* (No. 7), a = 16.7224(5) Å, b = 7.3682(2) Å, c = 9.8175(3) Å, b = 96.295(3)°, $a = g$ = 90°, V = 1202.36(6) Å^3, T = 100.03(11) K, Z = 4, Z' = 2, m(Mo K_a) = 0.248, 15763 reflections measured, 4794 unique (R_{int} = 0.0460) which were used in all calculations. The final wR_2 was 0.0653 (all data) and R_1 was 0.0347 (I≥2 s(I)).

*ii. Compound **46***

The systematic absences in the diffraction data were consistent for the space groups $P\bar{1}$ and $P1$. The *E*-statistics strongly suggested the centrosymmetric space group $P\bar{1}$ that yielded chemically reasonable and computationally stable results of refinement.[8,9,16,17] A successful solution by intrinsic phasing provided most non-hydrogen atoms from the *E*-map. The remaining non-hydrogen atoms were located in an alternating series of least-squares cycles and difference Fourier maps. All non-hydrogen atoms were refined with anisotropic displacement coefficients. All hydrogen atoms were included in the structure factor calculation at idealized positions and were allowed to ride on the neighboring atoms with relative isotropic displacement coefficients. There are two symmetry-independent Ni complexes and ~1.5 toluene solvent molecules in the asymmetric unit. The two Ni complexes have identical compositions but different conformations. Both ^{t}Bu groups in the Ni1 complex exhibit positional disorder. The ^{t}Bu group at C11 is disordered over two positions with the major component contribution of 0.741(14). The ^{t}Bu group at C16 is disordered over two positions with the major component contribution of 0.846(4). The naphthyl moiety in the Ni1a complex is disordered over two positions with the major component contribution of 0.652(4). All disordered groups were refined with geometric and atomic displacement parameter restraints. There were three or more partially occupied

[16] Sheldrick, G. M. (2013b). *XPREP*. Version 2013/1. Georg-August-Universität Göttingen, Göttingen, Germany.
[17] Sheldrick, G. M. (2013a). The *SHELX* homepage, http://shelx.uni-ac.gwdg.de/SHELX/.

solvent molecules of toluene also present in the asymmetric unit. A significant amount of time was invested in identifying and refining the disordered molecules. Bond length restraints were applied to model the molecules, but the resulting isotropic displacement coefficients suggested the molecules were mobile. In addition, the refinement was computationally unstable. Option "solvent mask" of program OLEX2[7] was used to correct the diffraction data for diffuse scattering effects and to identify the solvate molecule. The masking routine calculated the upper limit of volume that can be occupied [18]by the solvent to be 680 Å^3 or 19 % of the unit cell volume. The program calculated 143 electrons in the unit cell for the diffuse species. This approximately corresponds to three molecules of toluene in the unit cell (150 electrons), or 1.5 molecules of toluene in the asymmetric unit, or 0.75 molecules of toluene per Ni complex. It is very likely that the solvent molecules are disordered over several positions. Please note that all derived results in the following tables are based on the known contents. No data are given for the diffusely scattering species. The final least-squares refinement of 940 parameters against 15058 data resulted in residuals R (based on F^2 for $I \geq 2\sigma$) and wR (based on F^2 for all data) of 0.0417 and 0.1117, respectively. The final difference Fourier map was featureless.

Crystal Data for $C_{42.25}H_{43}N_3NiO_2$ (M=683.50 g/mol): triclinic, space group P-1 (no. 2), a = 14.9062(19) Å, b = 15.092(2) Å, c = 17.023(3) Å, α = 95.044(5)°, β = 110.577(7)°, γ = 90.126(6)°, V = 3569.1(9) Å^3, Z = 4, T = 100.00 K, μ(Cu Kα) = 1.089 mm^{-1}, $Dcalc$ = 1.272 g/cm^3, 64028 reflections measured (5.57° ≤ 2Θ ≤ 155.26°), 15058 unique (R_{int} = 0.0586, R_{sigma} = 0.0495) which were used in all calculations. The final R_1 was 0.0417 (I > 2σ(I)) and wR_2 was 0.1117 (all data).

C. Crystallographic Characterization

*i. Compound **13***

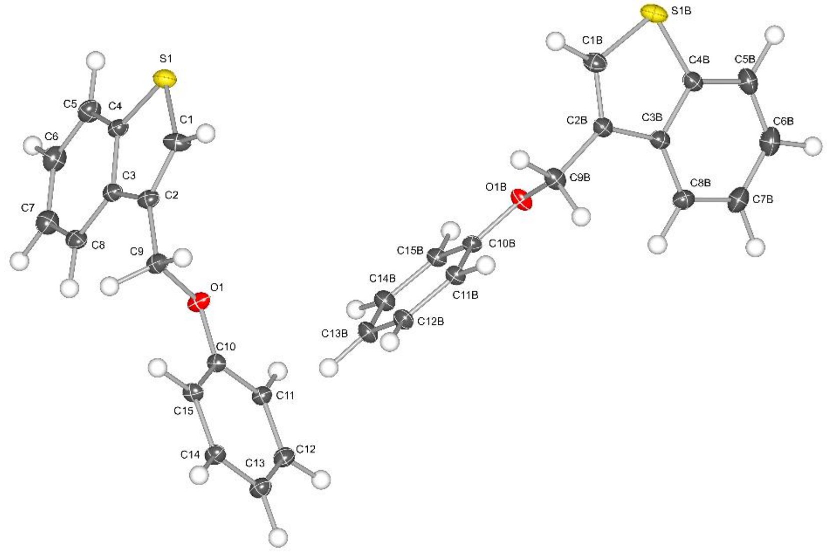

Figure S29: A molecular drawing of the compound (**13**). Thermal ellipsoidal (50% probability, non-H atoms only) representation of the asymmetric unit of the crystal structure.

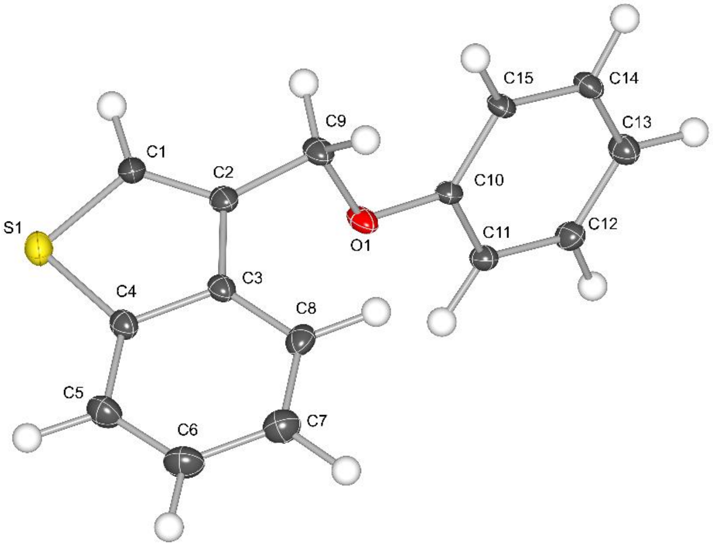

Figure S30: A molecular drawing of the compound (**13**). Thermal ellipsoidal (50% probability, non-H atoms only) representation of one of the two independent molecules in the asymmetric unit of the crystal structure.

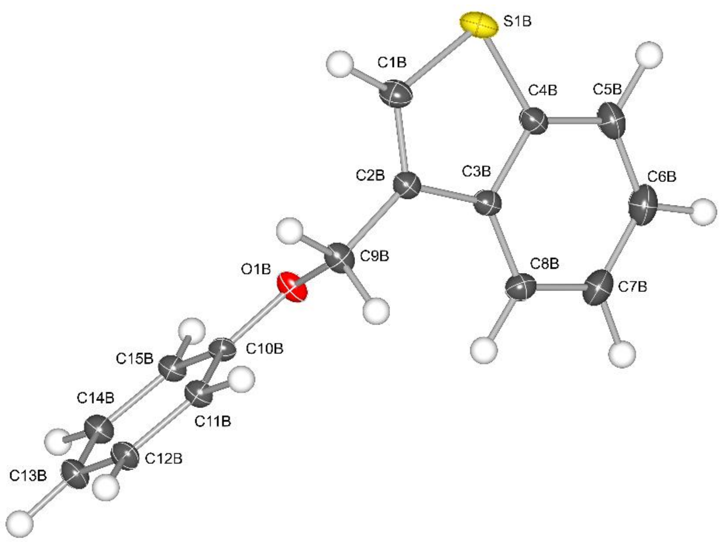

Figure S31: A molecular drawing of the compound (**13**). Thermal ellipsoidal (50% probability, non-H atoms only) representation of the second of the two independent molecules in the asymmetric unit of the crystal structure.

Table S37. Crystal data and structure refinement for compound **(13)**

Identification code	Compound **(13)**
Formula	$C_{15}H_{12}OS$
$D_{calc.}$/ g cm^{-3}	1.328
m/mm^{-1}	0.248
Formula Weight	240.328
Color	colorless
Shape	plate-shaped
Size/mm^3	0.40×0.11×0.03
T/K	100.03(11)
Crystal System	monoclinic
Flack Parameter	-0.00(4)
Hooft Parameter	-0.00(4)
Space Group	*Pc*
a/Å	16.7224(5)
b/Å	7.3682(2)
c/Å	9.8175(3)
a/°	90
b/°	96.295(3)
g/°	90
V/Å^3	1202.36(6)

Z	4
Z'	2
Wavelength/Å	0.71073
Radiation type	Mo K_a
$Q_{min}/^{\circ}$	3.46
$Q_{max}/^{\circ}$	29.20
Measured Refl's.	15763
Indep't Refl's	4794
Refl's I≥2 s(I)	4325
R_{int}	0.0460
Parameters	500
Restraints	439
Largest Peak	0.4619
Deepest Hole	-0.2862
GooF	1.0426
wR_2 (all data)	0.0653
wR_2	0.0632
R_1 (all data)	0.0413
R_1	0.0347

Table 38: Fractional Atomic Coordinates (×10^4) and Equivalent Isotropic Displacement Parameters (Å^2×10^3) for Compound **(13)**. U_{eq} is defined as 1/3 of the trace of the orthogonalised U_{ij}.

Atom	x	y	z	U_{eq}
S1	2027.9(3)	-591.8(6)	5003.9(5)	20.84(12)
O1	3135.2(8)	4934.4(18)	3941.7(12)	18.8(3)
C1	2491.1(13)	658(3)	3826.0(19)	21.1(4)
C2	2272.0(11)	2426(3)	3776.0(17)	17.2(4)
C3	1679.7(11)	2836(3)	4698.6(17)	15.8(4)
C4	1494.5(11)	1295(2)	5458.3(18)	17.2(4)
C5	960.0(11)	1393(3)	6451(2)	22.7(4)
C6	600.8(12)	3050(3)	6665(2)	25.6(5)
C7	767.4(13)	4593(3)	5901(2)	24.9(5)
C8	1303.6(12)	4490(3)	4924(2)	19.3(4)
C9	2644.9(12)	3849(3)	2957.6(19)	18.8(4)
C10	3567.2(11)	6315(2)	3457.4(17)	14.2(4)
C11	4086.4(11)	7225(2)	4446.0(19)	15.5(4)
C12	4564.3(11)	8620(3)	4056.1(19)	18.3(4)
C13	4523.3(11)	9155(3)	2678.5(19)	19.6(4)
C14	4000.2(10)	8244(3)	1708.1(19)	17.3(4)
C15	3522.7(11)	6819(2)	2080.1(18)	16.2(4)
S1B	7926.8(3)	-5763.3(7)	6010.6(5)	22.20(12)

Atom	x	y	z	U_{eq}
O1B	6876.2(8)	-104.5(17)	4650.3(11)	18.1(3)
C1B	7469.7(12)	-4425(3)	4709(2)	20.4(4)
C2B	7712.7(11)	-2671(3)	4779.2(18)	17.1(4)
C3B	8310.6(11)	-2350(3)	5927.2(18)	16.9(4)
C4B	8484.2(11)	-3931(2)	6712.5(19)	18.5(4)
C5B	9020.6(12)	-3927(3)	7900(2)	24.2(4)
C6B	9392.8(12)	-2300(3)	8301(2)	27.5(5)
C7B	9232.2(12)	-713(3)	7533(2)	25.2(4)
C8B	8698.1(12)	-725(3)	6346(2)	19.8(4)
C9B	7356.5(12)	-1208(3)	3848.5(19)	18.8(4)
C10B	6443.4(11)	1274(2)	4002.1(17)	13.8(4)
C11B	6487.5(11)	1784(3)	2645.5(18)	15.9(4)
C12B	6006.3(11)	3205(3)	2087(2)	17.7(4)
C13B	5482.9(11)	4110(3)	2857.6(19)	19.0(4)
C14B	5444.6(11)	3583(3)	4219.1(19)	18.4(4)
C15B	5923.9(11)	2192(2)	4791.9(19)	15.8(4)

Table 39: Anisotropic Displacement Parameters (×10[4]) for Compound **(13)**. The anisotropic displacement factor exponent takes the form: $-2p^2[h^2a^{*2} \times U_{11} + ... +2hka^* \times b^* \times U_{12}]$

Atom	U_{11}	U_{22}	U_{33}	U_{23}	U_{13}	U_{12}
S1	25.4(3)	15.8(2)	21.2(3)	-1.2(2)	1.8(2)	2.6(2)
O1	23.8(7)	21.4(7)	10.7(7)	-7.8(5)	-0.7(5)	1.2(5)
C1	34.9(12)	14.6(9)	14.3(10)	-1.4(7)	5.5(8)	-1.0(7)
H1	53(11)	30(8)	29(10)	10(4)	18(5)	1(4)
C2	21.4(10)	16.7(9)	13.3(9)	-4.2(6)	0.9(7)	-1.6(6)
C3	17.9(9)	16.7(9)	12.5(9)	-3.0(6)	-0.2(7)	0.7(6)
C4	18.4(10)	17.1(9)	15.9(9)	-4.1(7)	1.7(7)	1.0(6)
C5	22.1(10)	26.6(11)	20.2(10)	-3.5(7)	5.7(7)	2.1(7)
H5	56(14)	34(4)	55(12)	5(4)	34(7)	17(3)
C6	23.4(11)	32.0(11)	22.3(11)	-0.9(7)	7.0(8)	-2.5(7)
H6	55(11)	39(9)	50(9)	10(4)	37(5)	7(4)
C7	23.8(11)	26.4(11)	24.8(11)	-0.1(8)	3.8(7)	-3.0(7)
H7	20(10)	28(3)	38(11)	-1(2)	-1(5)	-9(3)
C8	22.6(10)	15.7(10)	18.7(11)	-1.8(7)	-1.7(7)	-3.0(7)
H8	32(11)	18(4)	40(10)	9(3)	19(5)	5(3)
C9	22.6(11)	20.6(10)	12.5(10)	-5.3(7)	-0.8(7)	0.4(7)
H9a	31(5)	26(7)	14(4)	1(2)	3(2)	4(2)

Atom	U_{11}	U_{22}	U_{33}	U_{23}	U_{13}	U_{12}
H9b	30(4)	27(6)	17(5)	1(2)	-4(2)	-1(2)
C10	17.6(9)	15.2(9)	9.8(9)	-0.6(6)	1.0(6)	-0.4(6)
C11	17.6(9)	16.3(9)	12.7(9)	-1.9(6)	2.2(6)	-1.1(6)
H11	55(10)	29(8)	11(2)	-22(5)	-2.1(19)	0.6(16)
C12	19.8(10)	17.8(10)	17.1(10)	-3.4(7)	2.1(7)	-2.4(6)
H12	40(9)	33(8)	14(2)	-14(5)	1.3(16)	4.1(14)
C13	20.3(10)	20.9(10)	17.4(10)	-4.2(7)	2.0(7)	0.7(7)
H13	55(12)	47(10)	34(9)	-31(5)	2(4)	8(4)
C14	19.7(10)	20.4(10)	12.0(10)	-3.0(7)	2.0(7)	2.5(7)
H14	40(9)	33(8)	14(2)	-14(5)	1.3(16)	4.1(14)
C15	20.8(10)	18.2(9)	9.7(9)	-1.2(7)	1.6(7)	1.5(6)
H15	55(10)	29(8)	11(2)	-22(5)	-2.1(19)	0.6(16)
S1B	32.1(3)	13.9(2)	21.1(3)	1.2(2)	5.2(2)	2.46(19)
O1B	23.4(7)	20.6(7)	10.7(7)	5.8(5)	4.5(5)	3.2(5)
C1B	26.7(11)	16.4(9)	18.3(10)	0.8(7)	2.2(8)	-0.9(7)
H1B	44(10)	22(8)	32(8)	-6(4)	-12(4)	1(4)
C2B	18.1(9)	17.4(9)	16.2(9)	1.3(6)	3.5(6)	0.6(6)
C3B	18.2(9)	15.7(9)	17.6(9)	1.0(6)	4.9(6)	0.9(6)
C4B	19.1(9)	17.7(9)	19.0(10)	2.9(6)	3.9(7)	3.0(6)
C5B	22.3(10)	29.6(11)	20.3(10)	6.2(7)	-0.5(7)	1.8(7)
H5B	33(12)	33(4)	31(9)	3(4)	-7(6)	8(3)
C6B	20.3(11)	38.2(11)	23.4(11)	0.8(7)	-0.9(8)	-2.2(7)
H6B	60(12)	39(10)	50(8)	1(4)	-33(5)	-2(4)
C7B	18.9(10)	31.0(11)	25.3(11)	-3.9(8)	1.3(7)	-3.2(7)
H7B	56(16)	35(4)	73(12)	-9(3)	-29(7)	-6(3)
C8B	20.5(10)	18.4(10)	21.0(11)	-2.2(7)	4.4(7)	-0.2(7)
H8B	34(12)	22(4)	36(9)	-7(3)	-9(5)	6(3)
C9B	20.9(11)	21.2(10)	15.3(10)	4.2(7)	6.4(7)	2.1(6)
H9Ba	26(5)	21(6)	31(7)	5(2)	14(2)	3(3)
H9Bb	35(6)	24(7)	19(4)	0(3)	1(2)	3(2)
C10B	17.0(9)	13.5(8)	11.1(9)	-0.6(6)	2.9(6)	1.2(6)
C11B	19.2(10)	17.5(9)	11.3(9)	0.8(7)	2.4(7)	0.8(6)
H11B	57(10)	38(8)	19(3)	29(6)	18(2)	12(2)
C12B	20.7(10)	18.9(9)	13.3(10)	1.7(7)	0.5(7)	3.4(7)
H12B	32(7)	51(9)	12(6)	23(4)	-1(3)	1(3)
C13B	21.0(10)	19.1(10)	16.7(10)	4.0(7)	1.2(7)	2.6(7)
H13B	30(10)	28(7)	23(8)	13(4)	7(4)	9(3)
C14B	21.4(10)	17.8(9)	16.0(10)	2.0(7)	2.6(7)	0.1(6)
H14B	32(7)	51(9)	12(6)	23(4)	-1(3)	1(3)
C15B	19.1(9)	15.5(9)	12.9(9)	1.1(6)	2.4(6)	0.1(6)
H15B	57(10)	38(8)	19(3)	29(6)	18(2)	12(2)

Table 40: Bond Lengths in Å for Compound **(13)**

Atom	Atom	Length/Å
S1	C1	1.727(2)
S1	C4	1.7361(19)
O1	C9	1.439(2)
O1	C10	1.363(2)
C1	H1	1.08(2)
C1	C2	1.352(3)
C2	C3	1.446(3)
C2	C9	1.498(3)
C3	C4	1.411(2)
C3	C8	1.400(3)
C4	C5	1.395(3)
C5	H5	1.10(2)
C5	C6	1.387(3)
C6	H6	1.04(2)
C6	C7	1.407(3)
C7	H7	1.14(2)
C7	C8	1.385(3)
C8	H8	1.08(2)
C9	H9a	1.08(2)
C9	H9b	1.09(2)
C10	C11	1.400(2)
C10	C15	1.396(2)
C11	H11	1.040(19)
C11	C12	1.381(3)
C12	H12	1.08(2)
C12	C13	1.403(3)
C13	H13	1.11(2)
C13	C14	1.393(3)
C14	H14	1.043(19)
C14	C15	1.392(3)
C15	H15	1.06(2)
S1B	C1B	1.725(2)
S1B	C4B	1.7393(19)
O1B	C9B	1.437(2)
O1B	C10B	1.364(2)

Atom	Atom	Length/Å
C1B	H1B	1.07(2)
C1B	C2B	1.354(3)
C2B	C3B	1.442(3)
C2B	C9B	1.494(3)
C3B	C4B	1.410(2)
C3B	C8B	1.401(3)
C4B	C5B	1.391(3)
C5B	H5B	1.14(2)
C5B	C6B	1.388(3)
C6B	H6B	1.06(2)
C6B	C7B	1.402(3)
C7B	H7B	1.13(2)
C7B	C8B	1.388(3)
C8B	H8B	1.10(2)
C9B	H9Ba	1.04(2)
C9B	H9Bb	1.09(2)
C10B	C11B	1.394(2)
C10B	C15B	1.401(3)
C11B	H11B	1.08(2)
C11B	C12B	1.395(3)
C12B	H12B	1.07(2)
C12B	C13B	1.389(3)
C13B	H13B	1.072(19)
C13B	C14B	1.400(3)
C14B	H14B	1.13(2)
C14B	C15B	1.382(3)
C15B	H15B	1.081(19)

Table 41: Bond Angles in Å for Compound **(13)**

Atom	Atom	Atom	Angle/°
C4	S1	C1	91.49(9)
C10	O1	C9	117.73(13)
H1	C1	S1	118.2(12)
C2	C1	S1	113.47(16)
C2	C1	H1	128.3(12)
C3	C2	C1	112.34(17)
C9	C2	C1	124.51(18)
C9	C2	C3	122.96(17)

Atom	Atom	Atom	Angle/°
C4	C3	C2	111.60(16)
C8	C3	C2	129.15(17)
C8	C3	C4	119.24(17)
C3	C4	S1	111.08(13)
C5	C4	S1	127.44(14)
C5	C4	C3	121.47(16)
H5	C5	C4	118.7(12)
C6	C5	C4	118.24(18)
C6	C5	H5	123.1(12)
H6	C6	C5	117.9(12)
C7	C6	C5	121.06(19)
C7	C6	H6	121.0(12)
H7	C7	C6	119.7(11)
C8	C7	C6	120.43(19)
C8	C7	H7	119.9(11)
C7	C8	C3	119.53(18)
H8	C8	C3	120.9(11)
H8	C8	C7	119.5(11)
C2	C9	O1	105.60(15)
H9a	C9	O1	107.3(10)
H9a	C9	C2	111.0(10)
H9b	C9	O1	111.7(11)
H9b	C9	C2	108.6(11)
H9b	C9	H9a	112.5(15)
C11	C10	O1	115.32(15)
C15	C10	O1	124.20(16)
C15	C10	C11	120.48(17)
H11	C11	C10	117.0(11)
C12	C11	C10	119.85(17)
C12	C11	H11	123.2(11)
H12	C12	C11	117.6(10)
C13	C12	C11	120.50(17)
C13	C12	H12	121.9(10)
H13	C13	C12	120.1(12)
C14	C13	C12	118.97(18)
C14	C13	H13	120.9(12)
H14	C14	C13	120.7(11)
C15	C14	C13	121.29(17)
C15	C14	H14	117.9(11)
C14	C15	C10	118.90(17)
H15	C15	C10	119.3(11)

Atom	Atom	Atom	Angle/°
H15	C15	C14	121.7(11)
C4B	S1B	C1B	91.40(10)
C10B	O1B	C9B	117.76(13)
H1B	C1B	S1B	120.9(12)
C2B	C1B	S1B	113.69(16)
C2B	C1B	H1B	125.4(12)
C3B	C2B	C1B	112.03(17)
C9B	C2B	C1B	124.06(18)
C9B	C2B	C3B	123.73(17)
C4B	C3B	C2B	112.04(16)
C8B	C3B	C2B	128.95(17)
C8B	C3B	C4B	118.99(17)
C3B	C4B	S1B	110.83(14)
C5B	C4B	S1B	127.02(15)
C5B	C4B	C3B	122.13(18)
H5B	C5B	C4B	123.2(11)
C6B	C5B	C4B	117.90(18)
C6B	C5B	H5B	118.9(11)
H6B	C6B	C5B	123.3(13)
C7B	C6B	C5B	120.95(19)
C7B	C6B	H6B	115.7(13)
H7B	C7B	C6B	119.1(13)
C8B	C7B	C6B	120.9(2)
C8B	C7B	H7B	119.8(13)
C7B	C8B	C3B	119.10(19)
H8B	C8B	C3B	118.7(11)
H8B	C8B	C7B	122.2(11)
C2B	C9B	O1B	106.41(14)
H9Ba	C9B	O1B	112.9(11)
H9Ba	C9B	C2B	108.1(11)
H9Bb	C9B	O1B	110.1(11)
H9Bb	C9B	C2B	111.1(10)
H9Bb	C9B	H9Ba	108.2(15)
C11B	C10B	O1B	124.46(17)
C15B	C10B	O1B	115.55(15)
C15B	C10B	C11B	119.99(17)
H11B	C11B	C10B	117.4(11)
C12B	C11B	C10B	119.28(18)
C12B	C11B	H11B	123.3(11)
H12B	C12B	C11B	119.2(11)
C13B	C12B	C11B	121.13(18)

Atom	Atom	Atom	Angle/°
C13B	C12B	H12B	119.7(11)
H13B	C13B	C12B	123.2(10)
C14B	C13B	C12B	118.97(18)
C14B	C13B	H13B	117.7(10)
H14B	C14B	C13B	120.5(10)
C15B	C14B	C13B	120.60(18)
C15B	C14B	H14B	118.9(10)
C14B	C15B	C10B	120.02(17)
H15B	C15B	C10B	117.7(11)
H15B	C15B	C14B	122.2(11)

Table 42: Torsion Angles in Å for Compound **(13)**

Atom	Atom	Atom	Atom	Angle/°
S1	C1	C2	C3	-1.75(16)
S1	C1	C2	C9	173.23(12)
S1	C4	C3	C2	-1.16(14)
S1	C4	C3	C8	179.82(12)
S1	C4	C5	C6	179.33(16)
O1	C9	C2	C1	-106.57(16)
O1	C9	C2	C3	67.90(17)
O1	C10	C11	C12	178.57(15)
O1	C10	C15	C14	-179.41(18)
C1	C2	C3	C4	1.87(18)
C1	C2	C3	C8	-179.23(15)
C2	C3	C4	C5	177.47(14)
C2	C3	C8	C7	-177.81(19)
C3	C4	C5	C6	0.9(2)
C3	C8	C7	C6	0.1(2)
C4	C5	C6	C7	0.2(2)
C5	C6	C7	C8	-0.7(2)
C10	C11	C12	C13	1.2(2)
C10	C15	C14	C13	0.7(2)
C11	C12	C13	C14	-0.8(2)
C12	C13	C14	C15	-0.2(2)
S1B	C1B	C2B	C3B	0.85(16)
S1B	C1B	C2B	C9B	-174.44(12)
S1B	C4B	C3B	C2B	1.01(15)
S1B	C4B	C3B	C8B	179.31(13)
S1B	C4B	C5B	C6B	-178.57(17)

Atom	Atom	Atom	Atom	Angle/°
O1B	C9B	C2B	C1B	106.59(16)
O1B	C9B	C2B	C3B	-68.16(18)
O1B	C10B	C11B	C12B	179.15(17)
O1B	C10B	C15B	C14B	-178.45(15)
C1B	C2B	C3B	C4B	-1.20(18)
C1B	C2B	C3B	C8B	-179.29(16)
C2B	C3B	C4B	C5B	-177.48(15)
C2B	C3B	C8B	C7B	177.0(2)
C3B	C4B	C5B	C6B	-0.3(2)
C3B	C8B	C7B	C6B	0.7(2)
C4B	C5B	C6B	C7B	0.0(2)
C5B	C6B	C7B	C8B	-0.2(2)
C10B	C11B	C12B	C13B	-0.3(2)
C10B	C15B	C14B	C13B	-1.2(2)
C11B	C12B	C13B	C14B	0.3(2)
C12B	C13B	C14B	C15B	0.5(2)

Table 43: Hydrogen Fractional Atomic Coordinates (×10[4]) and Equivalent Isotropic Displacement Parameters (Å[2]×10[3]) for Compound **(13)**. U_{eq} is defined as 1/3 of the trace of the orthogonalised U_{ij}.

Atom	x	y	z	U_{eq}
H1	2911(14)	-20(30)	3240(20)	36(6)
H5	842(16)	160(30)	7030(30)	46(7)
H6	197(14)	3110(30)	7400(20)	46(6)
H7	476(13)	5950(30)	6120(20)	29(5)
H8	1411(13)	5680(30)	4320(20)	29(5)
H9a	3040(12)	3250(30)	2287(19)	24(3)
H9b	2168(12)	4640(30)	2410(20)	25(3)
H11	4092(13)	6790(30)	5460(20)	32(4)
H12	4981(13)	9240(30)	4840(20)	29(4)
H13	4904(15)	10290(30)	2370(20)	45(7)
H14	3981(12)	8560(30)	670(20)	29(4)
H15	3110(14)	6150(30)	1350(20)	32(4)
H1B	7018(14)	-4970(30)	3970(20)	34(6)
H5B	9162(14)	-5180(30)	8560(20)	33(6)
H6B	9831(15)	-2180(30)	9160(20)	53(7)

Atom	x	y	z	U_{eq}
H7B	9510(16)	610(40)	7930(30)	57(8)
H8B	8555(14)	500(30)	5740(20)	32(6)
H9Ba	7824(12)	-490(30)	3490(20)	25(4)
H9Bb	6983(12)	-1780(30)	2970(20)	26(4)
H11B	6917(14)	1080(30)	2090(20)	37(4)
H12B	6026(12)	3560(30)	1040(20)	32(4)
H13B	5125(13)	5250(30)	2480(20)	27(5)
H14B	5014(13)	4280(30)	4860(20)	32(4)
H15B	5896(14)	1730(30)	5830(20)	37(4)

*ii. Compound **46***

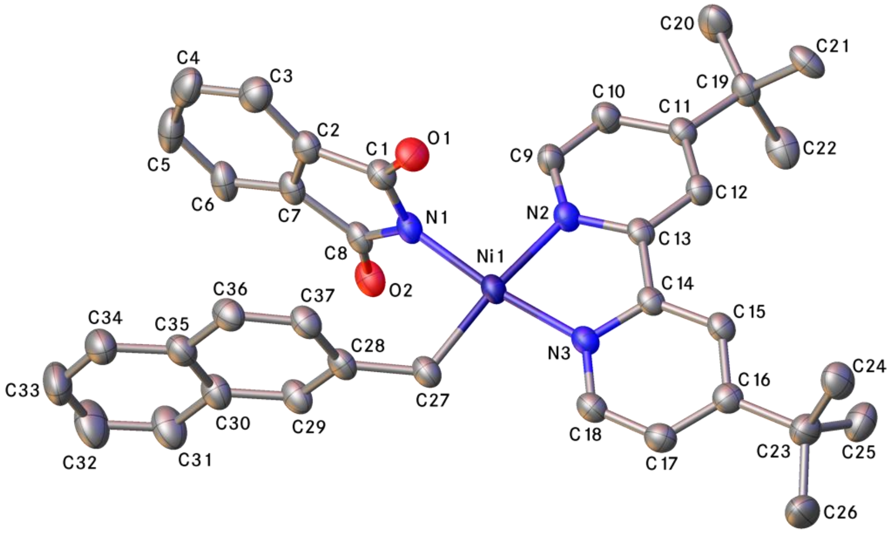

Figure S32: A molecular drawing of the first symmetry-independent Ni complex (**46**) shown with 50% probability ellipsoids. All H atoms and minor disorder components are omitted.

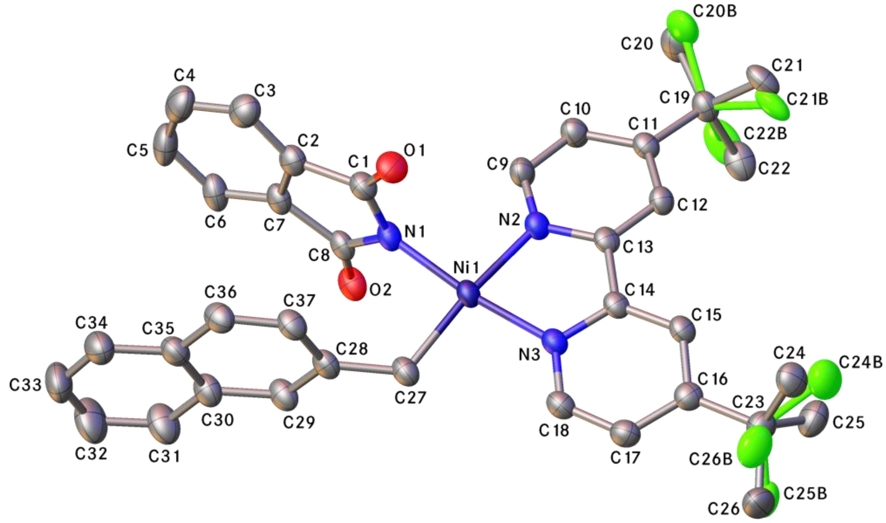

Figure S33: A molecular drawing of the first symmetry-independent Ni complex (**46**) shown with 50% probability ellipsoids. All H atoms are omitted but the minor disorder components are shown in green.

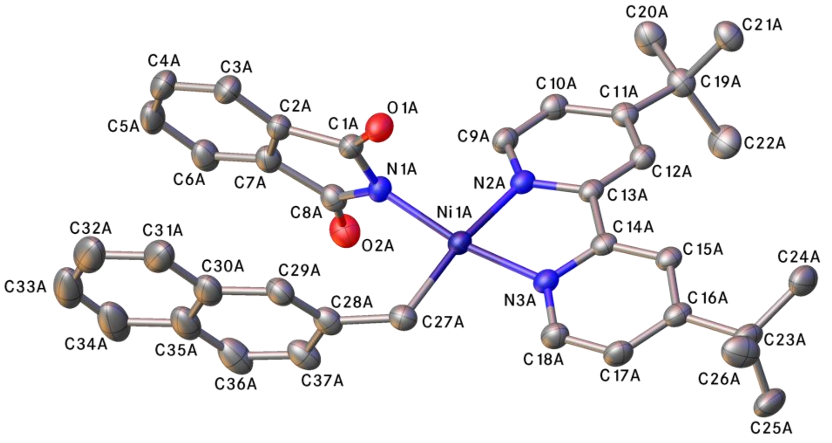

Figure S34: A molecular drawing of the second symmetry-independent Ni complex (**46**) shown with 50% probability ellipsoids. All H atoms and minor disorder components are omitted.

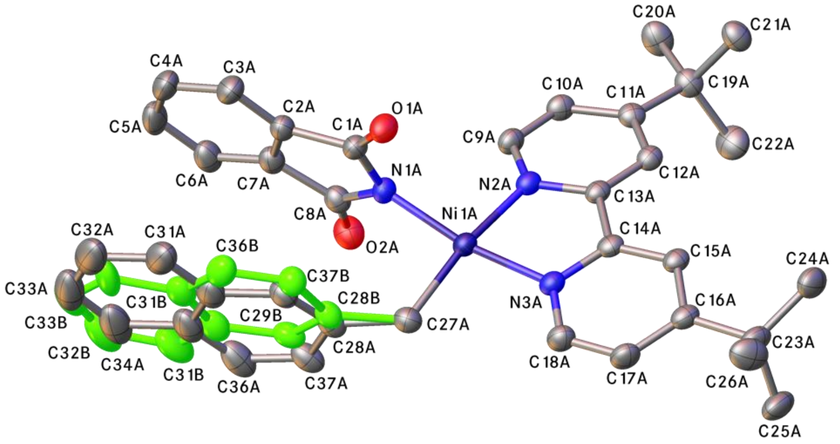

Figure S35: A molecular drawing of the second symmetry-independent Ni complex (**46**) shown with 50% probability ellipsoids. All H atoms are omitted but the minor disorder components are shown in green.

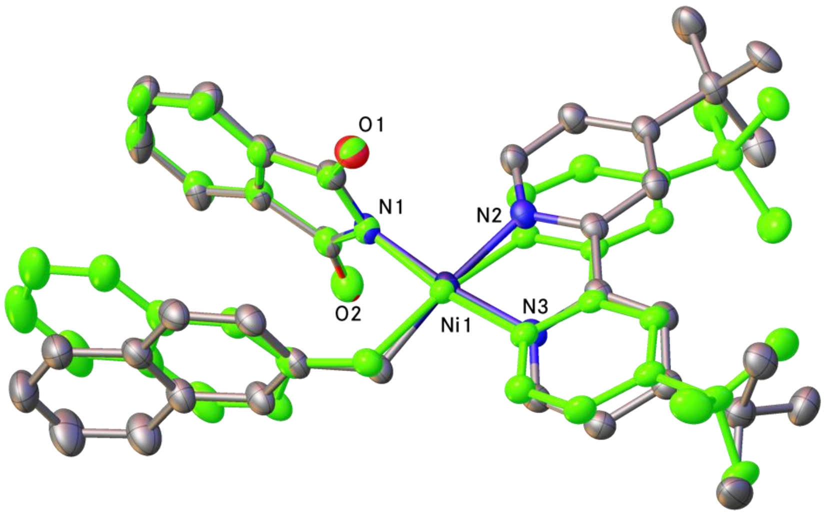

Figure S36: An overlay of the two symmetry-independent Ni complexes (**46**) shown with 50% probability ellipsoids. All H atoms and minor disorder components are omitted. The Ni1a complex is shown in green.

Table S44. Crystal data and structure refinement for Ni complex **(46)**

Identification code	Ni complex (**46**)
Empirical formula	$C_{37}H_{37}N_3NiO_2\ 0.75C_7H_8$
Formula weight	683.50
Temperature/K	100.00
Crystal system	triclinic
Space group	$P\bar{1}$
a/Å	14.9062(19)
b/Å	15.092(2)
c/Å	17.023(3)
α/°	95.044(5)
β/°	110.577(7)
γ/°	90.126(6)
Volume/Å^3	3569.1(9)
Z	4
ρ_{calc}g/cm^3	1.272
μ/mm^{-1}	1.089
F(000)	1446.0
Crystal size/mm^3	0.12 × 0.1 × 0.05
Radiation	Cu Kα (λ = 1.54178)
2Θ range for data collection/°	5.57 to 155.26

Index ranges	$-18 \le h \le 18$, $-19 \le k \le 19$, $-21 \le l \le 18$
Reflections collected	64028
Independent reflections	15058 [R_{int} = 0.0586, R_{sigma} = 0.0495]
Data/restraints/parameters	15058/381/940
Goodness-of-fit on F^2	1.031
Final R indexes [I>=2σ (I)]	R_1 = 0.0417, wR_2 = 0.1041
Final R indexes [all data]	R_1 = 0.0554, wR_2 = 0.1117
Largest diff. peak/hole / e Å^{-3}	0.41/-0.29

Table S45. Fractional Atomic Coordinates (×10^4) and Equivalent Isotropic Displacement Parameters (Å^2×10^3) for Ni complex (46) U_{eq} is defined as 1/3 of the trace of the orthogonalised U_{IJ} tensor

Atom	*x*	*y*	*z*	U(eq)
Ni1	5821.5(2)	6780.9(2)	1453.3(2)	26.35(8)
O1	4339.5(10)	7660.6(9)	-70.2(8)	37.4(3)
O2	5043.9(10)	7953.2(8)	2772.5(8)	34.2(3)
N1	4868.5(11)	7617.1(9)	1372.6(9)	28.8(3)
N2	4931.4(11)	5757.9(9)	1224.3(9)	27.4(3)
N3	6695.4(11)	5859.6(9)	1377.1(9)	27.1(3)
C1	4340.7(14)	7940.2(12)	626.7(12)	31.2(4)
C2	3790.7(14)	8710.9(12)	818.1(12)	32.6(4)
C3	3161.1(16)	9265.6(14)	300.3(14)	40.7(4)
C4	2836.7(17)	9981.2(14)	692.9(17)	47.9(5)
C5	3121.7(18)	10116.6(14)	1562.2(17)	48.2(5)
C6	3730.8(16)	9533.2(12)	2076.3(14)	38.4(4)
C7	4050.7(14)	8830.6(11)	1683.7(12)	30.1(4)
C8	4712.4(13)	8106.2(11)	2038.9(11)	28.1(3)
C9	4061.9(13)	5773.7(12)	1291.0(12)	32.0(4)
C10	3526.7(14)	5007.5(12)	1242.0(12)	32.6(4)
C11	3866.9(13)	4177.5(11)	1095.4(11)	27.0(3)
C12	4765.0(13)	4165.8(11)	1006.9(11)	27.6(3)
C13	5283.5(12)	4954.7(11)	1091.2(10)	25.6(3)
C14	6286.3(12)	5022.0(11)	1127.1(10)	26.0(3)
C15	6797.6(13)	4295.8(12)	985.0(11)	28.6(3)
C16	7778.1(13)	4388.2(12)	1137.6(11)	31.0(4)
C17	8186.1(14)	5244.1(13)	1400.5(12)	33.3(4)
C18	7634.0(13)	5946.0(12)	1502.3(12)	32.1(4)
C19	3325.1(13)	3315.5(12)	1092.1(12)	32.1(4)

Table S45. Fractional Atomic Coordinates (×10⁴) and Equivalent Isotropic Displacement Parameters (Å²×10³) for Ni complex (46) U_{eq} is defined as 1/3 of the trace of the orthogonalised U_{IJ} tensor

Atom	*x*	*y*	*z*	U(eq)
C20	2319(3)	3484(2)	1122(4)	45.9(11)
C21	3262(4)	2662(3)	344(3)	36.3(10)
C22	3906(4)	2889(3)	1910(2)	42.1(10)
C20B	2239(5)	3450(7)	650(14)	61(4)
C21B	3585(12)	2580(8)	522(9)	41(3)
C22B	3579(14)	3031(10)	1961(5)	53(4)
C23	8362.7(14)	3583.5(13)	1033.7(13)	38.1(4)
C27	6816.1(14)	7733.5(11)	1824.1(12)	31.4(4)
C28	6586.3(13)	8694.0(12)	1896.8(12)	31.7(4)
C29	6729.9(15)	9172.6(12)	2653.8(12)	34.7(4)
C30	6545.4(15)	10096.9(12)	2726.4(13)	36.8(4)
C31	6726(2)	10601.3(15)	3505.5(15)	50.9(6)
C32	6576(2)	11494.9(16)	3546.1(16)	61.7(7)
C33	6227(2)	11927.6(15)	2804.9(17)	57.8(7)
C34	6022.6(17)	11465.6(14)	2033.3(15)	46.3(5)
C35	6183.1(14)	10533.9(12)	1977.5(13)	36.2(4)
C36	6004.7(15)	10031.5(12)	1196.3(12)	36.1(4)
C37	6211.6(14)	9147.9(12)	1156.2(12)	33.8(4)
C24	8033.2(19)	3247.7(17)	86.5(16)	42.0(6)
C25	8136(2)	2821.1(17)	1496.2(18)	47.0(7)
C26	9429.5(18)	3786.3(19)	1360(2)	54.3(8)
C24B	7828(10)	2711(6)	726(11)	65(5)
C25B	9177(8)	3566(8)	1917(6)	44(3)
C26B	8893(10)	3844(9)	447(8)	54(4)
Ni1A	3005.9(2)	5364.8(2)	3364.1(2)	25.00(8)
O1A	2828.9(10)	6879.6(9)	4692.4(8)	34.6(3)
O2A	2705.7(10)	6834.0(10)	1977.9(9)	38.5(3)
N1A	2834.3(10)	6605.4(9)	3335.7(9)	26.8(3)
N2A	4393.7(10)	5460.6(9)	3643.8(9)	24.6(3)
N3A	3297.7(10)	4165.2(9)	3651.9(9)	25.6(3)
C1A	2682.3(12)	7110.8(11)	3987.2(11)	27.9(3)
C2A	2302.1(13)	7986.3(12)	3682.9(12)	30.1(4)
C3A	1983.1(15)	8696.7(13)	4069.1(14)	39.5(4)
C4A	1604.8(16)	9401.0(13)	3585.7(16)	45.9(5)
C5A	1551.6(15)	9379.4(14)	2759.8(17)	46.7(5)

Table S45. Fractional Atomic Coordinates (×10⁴) and Equivalent Isotropic Displacement Parameters (Å²×10³) for Ni complex (46) U_{eq} is defined as 1/3 of the trace of the orthogonalised U_{IJ} tensor

**Atom**	***x***	***y***	***z***	**U(eq)**
C6A	1879.0(14)	8657.1(13)	2377.3(14)	39.4(4)
C7A	2257.0(13)	7967.5(12)	2861.1(12)	31.5(4)
C8A	2614.4(12)	7087.0(12)	2639.3(12)	29.4(4)
C9A	4873.6(13)	6118.0(11)	3465.8(11)	27.8(3)
C10A	5837.4(13)	6073.7(11)	3565.2(11)	28.0(3)
C11A	6353.1(12)	5335.3(11)	3877.2(10)	25.7(3)
C19A	7399.5(13)	5224.0(12)	3952.5(11)	30.1(4)
C20A	7805.5(14)	6040.2(14)	3695.0(14)	40.0(4)
C21A	8014.1(13)	5088.8(14)	4865.4(12)	34.7(4)
C22A	7449.3(16)	4398.6(15)	3375.5(14)	42.7(5)
C12A	5848.3(12)	4657.9(11)	4083.6(10)	25.1(3)
C13A	4881.9(12)	4731.6(10)	3941.7(10)	23.1(3)
C14A	4246.0(12)	4012.3(11)	4015.2(10)	24.2(3)
C15A	4576.7(13)	3225.8(11)	4372.1(10)	25.6(3)
C16A	3931.1(14)	2540.2(11)	4341.4(11)	28.6(3)
C17A	2961.8(14)	2696.6(12)	3939.6(13)	33.4(4)
C18A	2676.7(13)	3496.2(12)	3613.2(12)	32.2(4)
C23A	4233.0(15)	1638.4(11)	4673.7(12)	34.6(4)
C24A	5289.5(16)	1652.5(13)	5236.3(14)	41.5(5)
C25A	4057.9(19)	962.8(13)	3906.6(15)	46.4(5)
C26A	3624.3(18)	1366.2(15)	5187.5(15)	48.0(5)
C27A	1605.5(13)	5115.6(12)	2912.6(12)	31.2(4)
C28A	968(4)	5808(4)	2466(3)	31.8(10)
C29A	556(2)	6383(2)	2889(3)	34.4(8)
C30A	-17(3)	7089(3)	2497(3)	45.9(11)
C31A	-394(2)	7721(2)	2942(3)	59.8(12)
C32A	-929(3)	8410(3)	2547(4)	73.9(14)
C33A	-1095(4)	8470(4)	1691(5)	71.6(16)
C34A	-746(3)	7874(3)	1232(4)	66.6(13)
C35A	-186(3)	7165(3)	1635(3)	49.2(12)
C36A	210(2)	6541(3)	1193(2)	48.0(9)
C37A	771(2)	5889(3)	1597(2)	38.3(8)
C28B	907(9)	5859(7)	2696(5)	31(2)
C29B	660(4)	6181(4)	1925(4)	34.7(14)
C30B	83(6)	6934(5)	1693(4)	40.1(19)

Table S45. Fractional Atomic Coordinates (×10^4^) and Equivalent Isotropic Displacement Parameters ($Å^2×10^3$) for Ni complex (46) U_{eq} is defined as 1/3 of the trace of the orthogonalised U_{IJ} tensor

Atom	*x*	*y*	*z*	U(eq)
C31B	-157(5)	7281(5)	910(4)	58(2)
C32B	-680(5)	8038(5)	738(6)	71(2)
C33B	-973(8)	8491(7)	1360(6)	67(3)
C34B	-763(5)	8174(5)	2124(6)	58(2)
C35B	-225(5)	7390(4)	2313(4)	43.8(19)
C36B	16(4)	7055(4)	3109(4)	41.4(15)
C37B	572(4)	6327(4)	3299(5)	32.8(14)

Table S46. Anisotropic Displacement Parameters ($Å^2×10^3$) for Ni complex (**46**). The Anisotropic displacement factor exponent takes the form: $-2\pi^2[h^2a^{*2}U_{11}+2hka^*b^*U_{12}+\ldots]$

Atom	U_{11}	U_{22}	U_{33}	U_{23}	U_{13}	U_{12}
Ni1	31.51(16)	21.19(14)	25.08(15)	-2.06(10)	9.42(12)	-2.36(11)
O1	42.7(8)	40.4(7)	26.3(6)	-3.5(5)	10.0(6)	1.8(6)
O2	46.9(8)	28.4(6)	29.8(7)	2.4(5)	16.9(6)	-4.7(5)
N1	36.2(8)	21.8(7)	27.6(7)	-1.7(5)	11.1(6)	-2.4(6)
N2	31.3(7)	24.6(7)	26.1(7)	-2.1(5)	10.9(6)	-0.6(6)
N3	29.8(7)	25.4(7)	24.5(7)	-0.7(5)	8.3(6)	-2.8(6)
C1	34.4(9)	28.2(8)	29.2(9)	-1.1(7)	9.8(7)	-3.5(7)
C2	36.8(9)	26.6(8)	36.2(10)	2.7(7)	15.3(8)	-3.4(7)
C3	42.8(11)	37.3(10)	43.5(11)	9.8(8)	16.0(9)	1.9(8)
C4	48.7(12)	32.7(10)	66.7(15)	16.4(10)	22.9(11)	8.2(9)
C5	61.7(14)	24.9(9)	69.1(16)	4.8(9)	36.5(12)	8.1(9)
C6	52.0(12)	24.2(8)	45.8(11)	-2.0(8)	27.2(10)	-2.7(8)
C7	36.5(9)	21.4(8)	35.4(9)	0.6(7)	16.7(8)	-4.9(7)
C8	35.8(9)	20.9(7)	29.4(9)	-0.3(6)	14.5(7)	-6.7(7)
C9	33.2(9)	25.5(8)	37.5(10)	-3.6(7)	14.2(8)	0.4(7)
C10	32.4(9)	30.7(9)	35.3(9)	-3.2(7)	14.4(8)	-1.8(7)
C11	29.8(8)	26.9(8)	23.5(8)	-1.0(6)	9.2(7)	-2.6(6)
C12	33.4(9)	24.2(8)	25.4(8)	-1.5(6)	11.6(7)	0.4(7)
C13	30.1(8)	26.2(8)	18.9(7)	-2.6(6)	7.9(6)	-1.1(6)
C14	30.6(8)	26.0(8)	20.4(7)	-1.5(6)	8.7(6)	-2.2(6)
C15	32.4(9)	26.5(8)	25.4(8)	-3.3(6)	9.7(7)	-2.4(7)

Table S46. Anisotropic Displacement Parameters (Å^2×10^3) for Ni complex (**46**). The Anisotropic displacement factor exponent takes the form: $-2\pi^2[h^2a^{*2}U_{11}+2hka^*b^*U_{12}+\ldots]$

**Atom**	**U_{11}**	**U_{22}**	**U_{33}**	**U_{23}**	**U_{13}**	**U_{12}**
C16	32.8(9)	33.3(9)	26.3(8)	0.2(7)	10.2(7)	1.9(7)
C17	29.3(9)	34.9(9)	34.1(9)	1.5(7)	9.7(7)	-2.3(7)
C18	32.2(9)	30.5(9)	31.4(9)	-1.3(7)	9.5(7)	-5.1(7)
C19	36.4(9)	27.3(8)	35.9(10)	-0.9(7)	17.7(8)	-3.5(7)
C20	41.7(17)	36.8(16)	66(3)	-1.2(17)	29.3(18)	-7.7(13)
C21	40(3)	37.5(17)	29.4(18)	-9.2(14)	13.3(17)	-16.4(16)
C22	63(3)	37.4(18)	33.0(16)	0.8(13)	25.8(15)	-4.1(17)
C20B	42(4)	34(5)	98(10)	-2(6)	17(5)	-13(3)
C21B	45(8)	38(5)	38(6)	-9(4)	14(5)	-23(4)
C22B	86(9)	53(7)	36(4)	-8(4)	42(5)	-19(6)
C23	34.0(10)	37.3(10)	41.5(11)	-0.9(8)	12.6(8)	5.5(8)
C27	36.6(9)	26.4(8)	29.0(9)	-0.1(7)	9.5(7)	-5.2(7)
C28	34.4(9)	26.3(8)	34.5(9)	-1.1(7)	13.1(8)	-7.4(7)
C29	44.3(10)	29.0(9)	29.9(9)	2.6(7)	11.8(8)	-6.7(8)
C30	44.8(11)	29.0(9)	36.7(10)	0.9(7)	15.0(8)	-4.9(8)
C31	72.7(16)	40.4(11)	37.0(11)	-3.0(9)	17.6(11)	-4.7(11)
C32	92(2)	42.5(13)	45.9(13)	-10.8(10)	21.4(13)	-0.9(13)
C33	71.5(17)	29.3(10)	62.0(16)	-6.4(10)	12.9(13)	2.8(10)
C34	47.8(12)	32.9(10)	50.8(13)	2.5(9)	8.7(10)	-1.3(9)
C35	35.5(10)	31.4(9)	39.3(10)	-2.2(8)	11.6(8)	-5.8(7)
C36	40.7(10)	32.3(9)	32.3(10)	6.1(7)	8.4(8)	-4.7(8)
C37	40.7(10)	31.8(9)	27.4(9)	-1.8(7)	11.2(8)	-5.5(8)
C24	45.4(14)	41.1(13)	42.1(14)	-5.7(10)	21.1(11)	3.4(10)
C25	51.6(15)	40.2(13)	49.5(16)	8.8(11)	17.4(12)	14.5(11)
C26	33.5(13)	45.6(15)	75(2)	-10.3(14)	11.6(13)	6.1(11)
C24B	56(8)	52(6)	85(11)	-14(6)	26(7)	8(5)
C25B	47(6)	28(6)	56(6)	4(4)	17(5)	13(5)
C26B	62(8)	56(8)	54(7)	1(6)	32(7)	19(6)
Ni1A	26.60(15)	20.27(14)	26.73(15)	1.05(10)	7.94(11)	0.91(11)
O1A	43.2(7)	30.0(6)	28.4(7)	4.0(5)	9.5(6)	4.5(5)
O2A	38.2(7)	46.1(8)	34.2(7)	7.4(6)	15.5(6)	5.8(6)
N1A	27.2(7)	22.6(7)	29.6(7)	3.6(5)	8.5(6)	2.8(5)
N2A	29.8(7)	21.3(6)	21.8(7)	0.9(5)	8.4(6)	0.3(5)

Table S46. Anisotropic Displacement Parameters (Å^2×10^3) for Ni complex (**46**). The Anisotropic displacement factor exponent takes the form: $-2\pi^2[h^2a^{*2}U_{11}+2hka^*b^*U_{12}+\ldots]$

**Atom**	U_{11}	U_{22}	U_{33}	U_{23}	U_{13}	U_{12}
N3A	29.1(7)	21.3(6)	26.2(7)	-0.5(5)	10.0(6)	0.3(5)
C1A	27.3(8)	22.2(8)	31.2(9)	1.5(6)	7.0(7)	0.7(6)
C2A	26.9(8)	24.6(8)	34.7(9)	3.8(7)	5.7(7)	1.1(6)
C3A	39.1(10)	28.5(9)	44.8(11)	-2.5(8)	8.8(9)	5.2(8)
C4A	40.4(11)	25.7(9)	62.9(14)	-0.3(9)	8.3(10)	6.2(8)
C5A	35.5(10)	28.8(10)	68.2(15)	17.0(9)	5.9(10)	4.8(8)
C6A	32.2(9)	34.9(10)	48.0(12)	16.2(8)	7.7(8)	-0.4(8)
C7A	25.4(8)	28.3(8)	38.5(10)	7.9(7)	7.3(7)	-0.7(7)
C8A	24.9(8)	32.1(9)	30.8(9)	6.2(7)	8.7(7)	0.5(7)
C9A	33.4(9)	21.7(8)	28.2(8)	4.6(6)	10.1(7)	0.6(6)
C10A	33.6(9)	25.3(8)	25.9(8)	4.6(6)	11.0(7)	-2.3(7)
C11A	29.6(8)	27.8(8)	19.5(7)	1.4(6)	8.8(6)	-0.6(6)
C19A	30.7(9)	34.6(9)	27.3(9)	5.7(7)	12.5(7)	0.7(7)
C20A	33.0(10)	47.7(11)	44.0(11)	15.7(9)	16.6(9)	-0.3(8)
C21A	30.6(9)	41.5(10)	31.5(9)	9.6(8)	8.7(7)	0.9(8)
C22A	42.8(11)	48.7(12)	40.9(11)	-1.0(9)	21.5(9)	5.3(9)
C12A	30.9(8)	24.1(8)	20.7(7)	2.1(6)	9.5(6)	1.4(6)
C13A	29.9(8)	20.2(7)	18.9(7)	0.2(6)	8.7(6)	-0.1(6)
C14A	30.0(8)	21.7(7)	21.2(7)	-2.9(6)	10.6(6)	-1.0(6)
C15A	32.3(8)	22.4(8)	21.5(8)	-0.2(6)	9.3(6)	-0.1(6)
C16A	38.0(9)	21.8(8)	25.4(8)	-0.5(6)	11.0(7)	-1.8(7)
C17A	34.2(9)	23.3(8)	42.1(10)	1.4(7)	13.0(8)	-5.9(7)
C18A	29.9(9)	26.9(8)	38.9(10)	1.1(7)	11.4(8)	-2.3(7)
C23A	46.2(11)	20.5(8)	32.9(9)	3.3(7)	8.8(8)	-4.4(7)
C24A	50.3(12)	26.8(9)	41.7(11)	9.3(8)	7.8(9)	2.2(8)
C25A	63.0(14)	23.0(9)	43.6(12)	-1.0(8)	7.8(10)	0.8(9)
C26A	55.6(13)	43.9(12)	42.0(12)	13.3(9)	12.0(10)	-12.7(10)
C27A	28.4(8)	31.6(9)	30.9(9)	1.2(7)	7.7(7)	-1.3(7)
C28A	23.7(16)	37.3(19)	34(2)	8.3(19)	8.6(18)	-3.5(13)
C29A	26.1(14)	38.2(17)	35(2)	4.1(15)	5.8(14)	0.6(12)
C30A	24.8(19)	38(2)	68(3)	8.9(17)	6.8(17)	-2.3(14)
C31A	31.3(16)	40.7(18)	96(3)	-5.9(18)	12.1(18)	1.4(14)
C32A	29.6(19)	45(2)	133(4)	-2(3)	14(2)	2.1(16)

Table S46. Anisotropic Displacement Parameters ($Å^2 \times 10^3$) for Ni complex (**46**). The Anisotropic displacement factor exponent takes the form: $-2\pi^2[h^2a^{*2}U_{11}+2hka^*b^*U_{12}+\ldots]$

Atom	U_{11}	U_{22}	U_{33}	U_{23}	U_{13}	U_{12}
C33A	31(2)	43(2)	130(5)	27(3)	11(3)	1.0(17)
C34A	33.2(18)	51(2)	105(4)	35(2)	5(2)	-6.2(16)
C35A	25(2)	46(3)	69(3)	17.6(18)	4.4(17)	-3.8(16)
C36A	33.3(16)	62(2)	47(2)	25.1(17)	7.2(14)	-5.0(14)
C37A	28.5(15)	51(2)	33.4(18)	9.1(15)	7.0(13)	-5.4(14)
C28B	25(3)	35(3)	32(4)	5(3)	7(3)	0(2)
C29B	29(3)	42(3)	30(3)	1(2)	6(2)	-5(2)
C30B	26(4)	41(4)	50(4)	12(3)	8(3)	-4(3)
C31B	46(4)	68(4)	48(4)	23(3)	-4(3)	-8(3)
C32B	42(4)	70(5)	83(5)	36(4)	-7(3)	-7(3)
C33B	33(5)	55(5)	93(6)	27(4)	-6(4)	-2(4)
C34B	22(3)	44(4)	97(5)	21(4)	1(3)	5(3)
C35B	26(3)	38(4)	60(4)	9(3)	5(3)	-4(3)
C36B	31(3)	35(3)	60(4)	2(2)	20(3)	0(2)
C37B	30(3)	34(3)	36(3)	5(2)	13(3)	2(2)

Table S47. Bond Lengths for Ni complex (**46**)

Atom	Atom	Length/Å	Atom	Atom	Length/Å
Ni1	N1	1.8791(16)	Ni1A	C27A	1.9767(18)
Ni1	N2	1.9538(15)	O1A	C1A	1.225(2)
Ni1	N3	1.9344(15)	O2A	C8A	1.210(2)
Ni1	C27	1.9587(18)	N1A	C1A	1.375(2)
O1	C1	1.223(2)	N1A	C8A	1.386(2)
O2	C8	1.214(2)	N2A	C9A	1.338(2)
N1	C1	1.370(2)	N2A	C13A	1.356(2)
N1	C8	1.387(2)	N3A	C14A	1.359(2)
N2	C9	1.340(2)	N3A	C18A	1.350(2)
N2	C13	1.354(2)	C1A	C2A	1.501(2)
N3	C14	1.368(2)	C2A	C3A	1.381(3)
N3	C18	1.343(2)	C2A	C7A	1.374(3)

Table S47. Bond Lengths for Ni complex (**46**)

Atom	Atom	Length/Å	Atom	Atom	Length/Å
C1	C2	1.502(3)	C3A	C4A	1.398(3)
C2	C3	1.382(3)	C4A	C5A	1.378(4)
C2	C7	1.380(3)	C5A	C6A	1.397(3)
C3	C4	1.396(3)	C6A	C7A	1.381(3)
C4	C5	1.384(4)	C7A	C8A	1.500(3)
C5	C6	1.395(3)	C9A	C10A	1.389(3)
C6	C7	1.378(3)	C10A	C11A	1.391(2)
C7	C8	1.495(3)	C11A	C19A	1.530(2)
C9	C10	1.383(3)	C11A	C12A	1.407(2)
C10	C11	1.387(3)	C19A	C20A	1.534(2)
C11	C12	1.399(2)	C19A	C21A	1.534(2)
C11	C19	1.528(2)	C19A	C22A	1.536(3)
C12	C13	1.387(2)	C12A	C13A	1.381(2)
C13	C14	1.477(2)	C13A	C14A	1.482(2)
C14	C15	1.388(2)	C14A	C15A	1.393(2)
C15	C16	1.396(3)	C15A	C16A	1.396(2)
C16	C17	1.394(3)	C16A	C17A	1.395(3)
C16	C23	1.530(2)	C16A	C23A	1.528(2)
C17	C18	1.379(3)	C17A	C18A	1.378(3)
C19	C20	1.539(3)	C23A	C24A	1.528(3)
C19	C21	1.516(3)	C23A	C25A	1.529(3)
C19	C22	1.555(4)	C23A	C26A	1.542(3)
C19	C20B	1.550(6)	C27A	C28A	1.487(4)
C19	C21B	1.550(6)	C27A	C28B	1.512(6)
C19	C22B	1.494(6)	C28A	C29A	1.361(5)
C23	C24	1.548(3)	C28A	C37A	1.419(5)
C23	C25	1.550(3)	C29A	C30A	1.427(5)
C23	C26	1.509(3)	C30A	C31A	1.405(5)
C23	C24B	1.491(7)	C30A	C35A	1.415(5)
C23	C25B	1.569(7)	C31A	C32A	1.386(5)
C23	C26B	1.550(7)	C32A	C33A	1.400(7)
C27	C28	1.495(2)	C33A	C34A	1.362(6)
C28	C29	1.367(3)	C34A	C35A	1.429(5)
C28	C37	1.424(3)	C35A	C36A	1.411(5)

Table S47. Bond Lengths for Ni complex (**46**)

Atom	Atom	Length/Å	Atom	Atom	Length/Å
C29	C30	1.426(3)	C36A	C37A	1.364(4)
C30	C31	1.408(3)	C28B	C29B	1.368(6)
C30	C35	1.420(3)	C28B	C37B	1.426(7)
C31	C32	1.367(3)	C29B	C30B	1.426(7)
C32	C33	1.405(4)	C30B	C31B	1.403(7)
C33	C34	1.364(3)	C30B	C35B	1.420(7)
C34	C35	1.427(3)	C31B	C32B	1.380(7)
C35	C36	1.410(3)	C32B	C33B	1.406(8)
C36	C37	1.371(3)	C33B	C34B	1.358(8)
Ni1A	N1A	1.8928(14)	C34B	C35B	1.426(7)
Ni1A	N2A	1.9548(15)	C35B	C36B	1.413(7)
Ni1A	N3A	1.9278(14)	C36B	C37B	1.369(6)

Table S48. Bond Angles for Ni complex **(46)**

Atom	Atom	Atom	Angle/˚	Atom	Atom	Atom	Angle/˚
N1	Ni1	N2	93.83(6)	N2A	Ni1A	C27A	169.76(7)
N1	Ni1	N3	172.02(6)	N3A	Ni1A	N2A	82.07(6)
N1	Ni1	C27	90.68(7)	N3A	Ni1A	C27A	93.62(7)
N2	Ni1	C27	172.14(7)	C1A	N1A	Ni1A	121.24(12)
N3	Ni1	N2	82.06(6)	C1A	N1A	C8A	109.72(14)
N3	Ni1	C27	94.19(7)	C8A	N1A	Ni1A	127.31(12)
C1	N1	Ni1	122.56(13)	C9A	N2A	Ni1A	125.99(12)
C1	N1	C8	110.07(15)	C9A	N2A	C13A	117.87(15)
C8	N1	Ni1	126.41(13)	C13A	N2A	Ni1A	115.50(11)
C9	N2	Ni1	125.64(12)	C14A	N3A	Ni1A	115.12(11)
C9	N2	C13	117.92(15)	C18A	N3A	Ni1A	127.74(13)
C13	N2	Ni1	115.89(12)	C18A	N3A	C14A	116.78(15)
C14	N3	Ni1	115.07(12)	O1A	C1A	N1A	125.54(16)
C18	N3	Ni1	128.32(12)	O1A	C1A	C2A	126.14(17)
C18	N3	C14	116.54(15)	N1A	C1A	C2A	108.32(15)
O1	C1	N1	125.64(18)	C3A	C2A	C1A	131.21(18)
O1	C1	C2	126.19(18)	C7A	C2A	C1A	106.79(16)
N1	C1	C2	108.15(16)	C7A	C2A	C3A	121.90(18)

Table S48. Bond Angles for Ni complex **(46)**

Atom	Atom	Atom	Angle/°	Atom	Atom	Atom	Angle/°
C3	C2	C1	131.64(19)	C2A	C3A	C4A	117.1(2)
C7	C2	C1	106.71(16)	C5A	C4A	C3A	121.1(2)
C7	C2	C3	121.61(18)	C4A	C5A	C6A	121.26(19)
C2	C3	C4	117.0(2)	C7A	C6A	C5A	117.2(2)
C5	C4	C3	121.2(2)	C2A	C7A	C6A	121.45(19)
C4	C5	C6	121.1(2)	C2A	C7A	C8A	107.46(16)
C7	C6	C5	117.3(2)	C6A	C7A	C8A	130.97(19)
C2	C7	C8	107.34(16)	O2A	C8A	N1A	125.39(17)
C6	C7	C2	121.66(19)	O2A	C8A	C7A	126.94(17)
C6	C7	C8	130.98(18)	N1A	C8A	C7A	107.67(15)
O2	C8	N1	125.46(17)	N2A	C9A	C10A	122.59(16)
O2	C8	C7	126.93(17)	C9A	C10A	C11A	120.42(16)
N1	C8	C7	107.59(15)	C10A	C11A	C19A	122.78(15)
N2	C9	C10	122.62(17)	C10A	C11A	C12A	116.55(16)
C9	C10	C11	120.51(17)	C12A	C11A	C19A	120.58(15)
C10	C11	C12	116.62(16)	C11A	C19A	C20A	111.73(15)
C10	C11	C19	121.94(16)	C11A	C19A	C21A	109.81(14)
C12	C11	C19	121.31(15)	C11A	C19A	C22A	108.31(15)
C13	C12	C11	120.27(16)	C20A	C19A	C22A	109.30(16)
N2	C13	C12	122.00(16)	C21A	C19A	C20A	108.48(16)
N2	C13	C14	112.56(15)	C21A	C19A	C22A	109.17(16)
C12	C13	C14	125.21(16)	C13A	C12A	C11A	119.99(15)
N3	C14	C13	113.77(15)	N2A	C13A	C12A	122.50(15)
N3	C14	C15	122.45(16)	N2A	C13A	C14A	112.38(15)
C15	C14	C13	123.61(15)	C12A	C13A	C14A	124.88(15)
C14	C15	C16	120.52(16)	N3A	C14A	C13A	113.53(14)
C15	C16	C23	121.09(16)	N3A	C14A	C15A	122.55(15)
C17	C16	C15	116.25(16)	C15A	C14A	C13A	123.79(16)
C17	C16	C23	122.65(17)	C14A	C15A	C16A	120.43(16)
C18	C17	C16	120.57(17)	C15A	C16A	C23A	123.85(17)
N3	C18	C17	123.59(17)	C17A	C16A	C15A	116.20(16)
C11	C19	C20	112.30(19)	C17A	C16A	C23A	119.86(16)
C11	C19	C22	106.8(2)	C18A	C17A	C16A	120.73(17)
C11	C19	C20B	108.2(4)	N3A	C18A	C17A	123.24(17)
C11	C19	C21B	108.5(6)	C16A	C23A	C25A	107.04(16)
C20	C19	C22	107.1(2)	C16A	C23A	C26A	110.13(17)

Table S48. Bond Angles for Ni complex **(46)**

Atom	Atom	Atom	Angle/˚	Atom	Atom	Atom	Angle/˚
C21	C19	C11	111.7(2)	C24A	C23A	C16A	112.03(15)
C21	C19	C20	110.5(3)	C24A	C23A	C25A	109.53(18)
C21	C19	C22	108.2(3)	C24A	C23A	C26A	108.42(17)
C20B	C19	C21B	105.3(6)	C25A	C23A	C26A	109.68(18)
C22B	C19	C11	111.4(6)	C28A	C27A	Ni1A	118.7(3)
C22B	C19	C20B	113.6(7)	C28B	C27A	Ni1A	121.3(6)
C22B	C19	C21B	109.6(7)	C29A	C28A	C27A	120.1(4)
C16	C23	C24	108.60(17)	C29A	C28A	C37A	118.4(3)
C16	C23	C25	108.92(17)	C37A	C28A	C27A	121.6(3)
C16	C23	C25B	104.5(4)	C28A	C29A	C30A	121.9(4)
C16	C23	C26B	105.8(5)	C31A	C30A	C29A	122.4(4)
C24	C23	C25	107.10(19)	C31A	C30A	C35A	119.2(4)
C26	C23	C16	113.26(18)	C35A	C30A	C29A	118.4(4)
C26	C23	C24	109.1(2)	C32A	C31A	C30A	120.9(5)
C26	C23	C25	109.7(2)	C31A	C32A	C33A	119.1(5)
C24B	C23	C16	116.9(6)	C34A	C33A	C32A	122.2(5)
C24B	C23	C25B	112.7(7)	C33A	C34A	C35A	119.1(5)
C24B	C23	C26B	111.6(7)	C30A	C35A	C34A	119.4(4)
C26B	C23	C25B	104.3(7)	C36A	C35A	C30A	119.1(3)
C28	C27	Ni1	122.34(14)	C36A	C35A	C34A	121.4(4)
C29	C28	C27	122.78(17)	C37A	C36A	C35A	120.5(4)
C29	C28	C37	117.48(17)	C36A	C37A	C28A	121.6(4)
C37	C28	C27	119.74(16)	C29B	C28B	C27A	119.9(5)
C28	C29	C30	122.84(18)	C29B	C28B	C37B	116.7(5)
C31	C30	C29	123.14(19)	C37B	C28B	C27A	122.9(5)
C31	C30	C35	118.46(18)	C28B	C29B	C30B	124.0(6)
C35	C30	C29	118.38(18)	C31B	C30B	C29B	124.4(6)
C32	C31	C30	121.1(2)	C31B	C30B	C35B	117.9(6)
C31	C32	C33	120.4(2)	C35B	C30B	C29B	117.6(5)
C34	C33	C32	120.8(2)	C32B	C31B	C30B	121.6(7)
C33	C34	C35	119.7(2)	C31B	C32B	C33B	120.0(7)
C30	C35	C34	119.57(19)	C34B	C33B	C32B	120.3(8)
C36	C35	C30	118.61(17)	C33B	C34B	C35B	120.5(7)
C36	C35	C34	121.81(19)	C30B	C35B	C34B	119.6(6)
C37	C36	C35	121.06(18)	C36B	C35B	C30B	118.8(5)
C36	C37	C28	121.57(17)	C36B	C35B	C34B	121.6(6)

Table S48. Bond Angles for Ni complex **(46)**

Atom	Atom	Atom	Angle/˚	Atom	Atom	Atom	Angle/˚
N1A	Ni1A	N2A	94.49(6)	C37B	C36B	C35B	121.2(6)
N1A	Ni1A	N3A	167.64(6)	C36B	C37B	C28B	121.6(6)
N1A	Ni1A	C27A	91.60(7)				

Table S49. Torsion Angles for Ni complex **(46)**

A	B	C	D	Angle/˚	A	B	C	D	Angle/˚
Ni1	N1	C1	O1	9.0(3)	Ni1A	N2A	C13A	C14A	-1.65(17)
Ni1	N1	C1	C2	-169.33(11)	Ni1A	N3A	C14A	C13A	-13.42(17)
Ni1	N1	C8	O2	-14.7(3)	Ni1A	N3A	C14A	C15A	170.46(12)
Ni1	N1	C8	C7	166.65(12)	Ni1A	N3A	C18A	C17A	-170.93(15)
Ni1	N2	C9	C10	-170.12(14)	Ni1A	C27A	C28A	C29A	-95.8(5)
Ni1	N2	C13	C12	173.32(13)	Ni1A	C27A	C28A	C37A	84.4(6)
Ni1	N2	C13	C14	-1.43(18)	Ni1A	C27A	C28B	C29B	84.9(11)
Ni1	N3	C14	C13	-9.13(18)	Ni1A	C27A	C28B	C37B	-87.0(11)
Ni1	N3	C14	C15	175.53(13)	O1A	C1A	C2A	C3A	-4.2(3)
Ni1	N3	C18	C17	-177.49(14)	O1A	C1A	C2A	C7A	179.55(18)
Ni1	C27	C28	C29	104.2(2)	N1A	C1A	C2A	C3A	175.47(19)
Ni1	C27	C28	C37	-76.5(2)	N1A	C1A	C2A	C7A	-0.8(2)
O1	C1	C2	C3	1.5(3)	N2A	Ni1A	N1A	C1A	-103.60(14)
O1	C1	C2	C7	-176.06(18)	N2A	Ni1A	N1A	C8A	92.96(15)
N1	C1	C2	C3	179.9(2)	N2A	C9A	C10A	C11A	-1.4(3)
N1	C1	C2	C7	2.3(2)	N2A	C13A	C14A	N3A	9.69(19)
N2	Ni1	N1	C1	-89.35(14)	N2A	C13A	C14A	C15A	-174.24(15)
N2	Ni1	N1	C8	102.95(14)	N3A	Ni1A	N1A	C1A	-30.3(4)
N2	C9	C10	C11	-1.8(3)	N3A	Ni1A	N1A	C8A	166.2(3)
N2	C13	C14	N3	6.8(2)	N3A	C14A	C15A	C16A	2.3(2)
N2	C13	C14	C15	-177.91(16)	C1A	N1A	C8A	O2A	177.36(18)
N3	C14	C15	C16	3.4(3)	C1A	N1A	C8A	C7A	-2.16(19)
C1	N1	C8	O2	176.32(17)	C1A	C2A	C3A	C4A	-175.53(19)
C1	N1	C8	C7	-2.33(19)	C1A	C2A	C7A	C6A	175.99(17)
C1	C2	C3	C4	-174.1(2)	C1A	C2A	C7A	C8A	-0.48(19)
C1	C2	C7	C6	174.82(17)	C2A	C3A	C4A	C5A	0.3(3)
C1	C2	C7	C8	-3.55(19)	C2A	C7A	C8A	O2A	-177.90(18)
C2	C3	C4	C5	-1.1(3)	C2A	C7A	C8A	N1A	1.6(2)

Table S49. Torsion Angles for Ni complex **(46)**

A	B	C	D	Angle/˚	A	B	C	D	Angle/˚
C2	C7	C8	O2	-174.87(17)	C3A	C2A	C7A	C6A	-0.7(3)
C2	C7	C8	N1	3.75(19)	C3A	C2A	C7A	C8A	-177.19(17)
C3	C2	C7	C6	-3.1(3)	C3A	C4A	C5A	C6A	-0.4(3)
C3	C2	C7	C8	178.55(17)	C4A	C5A	C6A	C7A	0.0(3)
C3	C4	C5	C6	-1.1(4)	C5A	C6A	C7A	C2A	0.6(3)
C4	C5	C6	C7	1.3(3)	C5A	C6A	C7A	C8A	176.09(19)
C5	C6	C7	C2	0.7(3)	C6A	C7A	C8A	O2A	6.1(3)
C5	C6	C7	C8	178.68(19)	C6A	C7A	C8A	N1A	-174.40(19)
C6	C7	C8	O2	7.0(3)	C7A	C2A	C3A	C4A	0.3(3)
C6	C7	C8	N1	-174.42(19)	C8A	N1A	C1A	O1A	-178.49(17)
C7	C2	C3	C4	3.2(3)	C8A	N1A	C1A	C2A	1.88(19)
C8	N1	C1	O1	178.48(18)	C9A	N2A	C13A	C12A	1.6(2)
C8	N1	C1	C2	0.2(2)	C9A	N2A	C13A	C14A	-173.09(14)
C9	N2	C13	C12	1.3(2)	C9A	C10A	C11A	C19A	176.34(16)
C9	N2	C13	C14	-173.42(15)	C9A	C10A	C11A	C12A	-0.3(2)
C9	C10	C11	C12	0.3(3)	C10A	C11A	C19A	C20A	3.4(2)
C9	C10	C11	C19	176.10(17)	C10A	C11A	C19A	C21A	123.81(18)
C10	C11	C12	C13	1.9(3)	C10A	C11A	C19A	C22A	-117.05(19)
C10	C11	C19	C20	9.3(3)	C10A	C11A	C12A	C13A	2.5(2)
C10	C11	C19	C21	134.0(3)	C11A	C12A	C13A	N2A	-3.3(2)
C10	C11	C19	C22	-107.9(3)	C11A	C12A	C13A	C14A	170.70(15)
C10	C11	C19	C20B	39.6(10)	C19A	C11A	C12A	C13A	-174.20(15)
C10	C11	C19	C21B	153.4(7)	C12A	C11A	C19A	C20A	179.91(16)
C10	C11	C19	C22B	-85.9(9)	C12A	C11A	C19A	C21A	-59.7(2)
C11	C12	C13	N2	-2.8(3)	C12A	C11A	C19A	C22A	59.5(2)
C11	C12	C13	C14	171.22(16)	C12A	C13A	C14A	N3A	-164.83(15)
C12	C11	C19	C20	-175.1(3)	C12A	C13A	C14A	C15A	11.2(2)
C12	C11	C19	C21	-50.4(3)	C13A	N2A	C9A	C10A	0.8(2)
C12	C11	C19	C22	67.7(3)	C13A	C14A	C15A	C16A	-173.40(15)
C12	C11	C19	C20B	-144.8(10)	C14A	N3A	C18A	C17A	1.7(3)
C12	C11	C19	C21B	-31.0(7)	C14A	C15A	C16A	C17A	0.0(2)
C12	C11	C19	C22B	89.7(9)	C14A	C15A	C16A	C23A	176.56(16)
C12	C13	C14	N3	-167.75(16)	C15A	C16A	C17A	C18A	-1.4(3)
C12	C13	C14	C15	7.5(3)	C15A	C16A	C23A	C24A	14.6(3)
C13	N2	C9	C10	1.0(3)	C15A	C16A	C23A	C25A	-105.5(2)
C13	C14	C15	C16	-171.48(16)	C15A	C16A	C23A	C26A	135.33(19)

Table S49. Torsion Angles for Ni complex **(46)**

A	B	C	D	Angle/˚	A	B	C	D	Angle/˚
C14	N3	C18	C17	-0.7(3)	C16A	C17A	C18A	N3A	0.5(3)
C14	C15	C16	C17	-2.6(3)	C17A	C16A	C23A	C24A	-168.98(18)
C14	C15	C16	C23	176.36(17)	C17A	C16A	C23A	C25A	70.9(2)
C15	C16	C17	C18	0.3(3)	C17A	C16A	C23A	C26A	-48.2(2)
C15	C16	C23	C24	68.0(2)	C18A	N3A	C14A	C13A	173.00(15)
C15	C16	C23	C25	-48.3(2)	C18A	N3A	C14A	C15A	-3.1(2)
C15	C16	C23	C26	-170.6(2)	C23A	C16A	C17A	C18A	-178.06(18)
C15	C16	C23	C24B	2.8(8)	C27A	Ni1A	N1A	C1A	84.64(14)
C15	C16	C23	C25B	-122.5(6)	C27A	Ni1A	N1A	C8A	-78.80(15)
C15	C16	C23	C26B	127.7(6)	C27A	C28A	C29A	C30A	176.4(4)
C16	C17	C18	N3	1.4(3)	C27A	C28A	C37A	C36A	-177.9(4)
C17	C16	C23	C24	-113.1(2)	C27A	C28B	C29B	C30B	-173.6(8)
C17	C16	C23	C25	130.5(2)	C27A	C28B	C37B	C36B	173.3(8)
C17	C16	C23	C26	8.2(3)	C28A	C29A	C30A	C31A	-175.7(5)
C17	C16	C23	C24B	-178.4(8)	C28A	C29A	C30A	C35A	2.5(7)
C17	C16	C23	C25B	56.3(6)	C29A	C28A	C37A	C36A	2.3(8)
C17	C16	C23	C26B	-53.4(6)	C29A	C30A	C31A	C32A	178.5(4)
C18	N3	C14	C13	173.65(15)	C29A	C30A	C35A	C34A	-179.1(4)
C18	N3	C14	C15	-1.7(2)	C29A	C30A	C35A	C36A	0.4(7)
C19	C11	C12	C13	-173.90(16)	C30A	C31A	C32A	C33A	0.3(6)
C23	C16	C17	C18	-178.61(18)	C30A	C35A	C36A	C37A	-1.8(7)
C27	Ni1	N1	C1	97.09(15)	C31A	C30A	C35A	C34A	-0.8(7)
C27	Ni1	N1	C8	-70.61(15)	C31A	C30A	C35A	C36A	178.7(4)
C27	C28	C29	C30	177.55(18)	C31A	C32A	C33A	C34A	-0.3(8)
C27	C28	C37	C36	-179.04(18)	C32A	C33A	C34A	C35A	-0.3(8)
C28	C29	C30	C31	-177.5(2)	C33A	C34A	C35A	C30A	0.8(7)
C28	C29	C30	C35	1.0(3)	C33A	C34A	C35A	C36A	-178.7(5)
C29	C28	C37	C36	0.2(3)	C34A	C35A	C36A	C37A	177.7(4)
C29	C30	C31	C32	177.1(3)	C35A	C30A	C31A	C32A	0.3(6)
C29	C30	C35	C34	-177.9(2)	C35A	C36A	C37A	C28A	0.5(6)
C29	C30	C35	C36	1.2(3)	C37A	C28A	C29A	C30A	-3.8(8)
C30	C31	C32	C33	0.8(5)	C28B	C29B	C30B	C31B	178.6(10)
C30	C35	C36	C37	-2.7(3)	C28B	C29B	C30B	C35B	2.1(14)
C31	C30	C35	C34	0.7(3)	C29B	C28B	C37B	C36B	1.2(15)
C31	C30	C35	C36	179.8(2)	C29B	C30B	C31B	C32B	-176.8(8)
C31	C32	C33	C34	0.5(5)	C29B	C30B	C35B	C34B	176.9(7)

Table S49. Torsion Angles for Ni complex **(46)**

A	B	C	D	Angle/˚	A	B	C	D	Angle/˚
C32	C33	C34	C35	-1.2(4)	C29B	C30B	C35B	C36B	-2.8(12)
C33	C34	C35	C30	0.6(3)	C30B	C31B	C32B	C33B	1.1(13)
C33	C34	C35	C36	-178.5(2)	C30B	C35B	C36B	C37B	2.9(11)
C34	C35	C36	C37	176.4(2)	C31B	C30B	C35B	C34B	0.2(13)
C35	C30	C31	C32	-1.4(4)	C31B	C30B	C35B	C36B	-179.6(7)
C35	C36	C37	C28	2.0(3)	C31B	C32B	C33B	C34B	-1.6(15)
C37	C28	C29	C30	-1.7(3)	C32B	C33B	C34B	C35B	1.5(14)
Ni1A	N1A	C1A	O1A	15.4(2)	C33B	C34B	C35B	C30B	-0.8(13)
Ni1A	N1A	C1A	C2A	-164.19(11)	C33B	C34B	C35B	C36B	179.0(8)
Ni1A	N1A	C8A	O2A	-17.6(3)	C34B	C35B	C36B	C37B	-176.8(7)
Ni1A	N1A	C8A	C7A	162.84(12)	C35B	C30B	C31B	C32B	-0.4(13)
Ni1A	N2A	C9A	C10A	-169.67(13)	C35B	C36B	C37B	C28B	-2.1(11)
Ni1A	N2A	C13A	C12A	173.02(12)	C37B	C28B	C29B	C30B	-1.2(16)

Table S50. Hydrogen Atom Coordinates (Å×10^4) and Isotropic Displacement Parameters (Å^2×10^3) for Ni complex (**46**)

Atom	*x*	*y*	*z*	U(eq)
H3	2958.13	9164.53	-296.18	49
H4	2412.5	10382.88	357.23	58
H5	2898.65	10614.97	1812.78	58
H6	3917.51	9616.71	2672.65	46
H9	3802.94	6333.56	1375.26	38
H10	2921.56	5049.76	1309.24	39
H12	5020.84	3615.17	888.54	33
H15	6477.73	3731.49	782.39	34
H17	8850.3	5344.88	1510.5	40
H18	7933.78	6522.22	1670.11	39
H20A	2001.58	2915.74	1124.12	69
H20B	1939.69	3778.96	627.08	69
H20C	2376.23	3865.98	1635.26	69
H21A	3907.25	2564.43	331.63	54
H21B	2865.6	2904.14	-177.91	54
H21C	2973.53	2095.69	393.75	54
H22A	3993.35	3317.29	2400.67	63

Table S50. Hydrogen Atom Coordinates (Å×10[4]) and Isotropic Displacement Parameters (Å[2]×10[3]) for Ni complex (**46**)

**Atom**	***x***	***y***	***z***	**U(eq)**
H22B	4534.57	2724.47	1891.45	63
H22C	3557.24	2355.51	1954.63	63
H20D	1879.92	2896.26	622.25	91
H20E	2118.16	3613.92	78.63	91
H20F	2031.07	3926.03	971.32	91
H21D	4239.34	2393.52	810.04	62
H21E	3543.45	2812.62	-8.94	62
H21F	3136.18	2067.93	403.83	62
H22D	3249.63	2460.3	1933.7	80
H22E	3379.44	3481.01	2311.52	80
H22F	4273.46	2965.67	2206.15	80
H27A	7264.32	7605.67	2385.09	38
H27B	7179.21	7669.73	1435.67	38
H29	6962.59	8876.24	3152.92	42
H31	6954.65	10316.06	4011.31	61
H32	6709.92	11825.19	4078.37	74
H33	6130.88	12549.81	2841.49	69
H34	5774.93	11762.49	1535.56	56
H36	5736.67	10309.83	689.51	43
H37	6102.58	8830.62	621.74	41
H24A	7339.5	3121.18	-130.61	63
H24B	8365.12	2703.5	15.35	63
H24C	8186.44	3705.93	-225.02	63
H25A	8322.92	3014.47	2098.52	70
H25B	8494.29	2297.02	1419.85	70
H25C	7447.15	2669.77	1262.5	70
H26A	9578.72	4219.61	1023.65	81
H26B	9766.89	3237.58	1318.65	81
H26C	9635.67	4033.54	1950.83	81
H24D	7362.02	2743.66	156.05	97
H24E	7491.91	2573.31	1103.98	97
H24F	8280.29	2244.07	714.16	97
H25D	9537.01	4138.22	2071.45	66
H25E	9611.57	3087.31	1891.25	66
H25F	8892.14	3462.96	2340.99	66

Table S50. Hydrogen Atom Coordinates (Å×10[4]) and Isotropic Displacement Parameters (Å[2]×10[3]) for Ni complex (**46**)

**Atom**	*x*	*y*	*z*	**U(eq)**
H26D	9483.69	3512.34	562.55	81
H26E	9053.43	4483.81	549.12	81
H26F	8479.01	3699.76	-142.37	81
H3A	2019.11	8706.6	4638.14	47
H4A	1380.66	9902.62	3830.95	55
H5A	1287.34	9865.07	2444.32	56
H6A	1842.96	8641.06	1807.82	47
H9A	4541.88	6635.54	3263.3	33
H10A	6146.62	6550.52	3419.29	34
H20G	7409.33	6145.26	3117.38	60
H20H	8464.39	5933.86	3724.58	60
H20I	7801.97	6562.81	4078.62	60
H21G	8012.38	5626.64	5232.01	52
H21H	8672.96	4971.15	4901.35	52
H21I	7749.53	4582.1	5046.32	52
H22G	7253.37	3867.11	3574.76	64
H22H	8107.77	4340.2	3385.28	64
H22I	7018.73	4461.91	2798.11	64
H12A	6172.35	4150.03	4320.44	30
H15A	5245.73	3155.32	4638.19	31
H17A	2493.04	2246.95	3890.33	40
H18A	2010.19	3580.7	3347.98	39
H24G	5688.8	1790.51	4909.33	62
H24H	5449.44	1068.38	5448.3	62
H24I	5407.92	2107.47	5712.36	62
H25G	3375.22	936.2	3558.2	70
H25H	4251.21	374.58	4095.16	70
H25I	4435.18	1144.7	3574.81	70
H26G	3694.99	1826.83	5650.44	72
H26H	3842.12	799.94	5418.01	72
H26I	2948.91	1298.84	4820.62	72
H27C	1479.72	4567.55	2519.89	37
H27D	1408.71	4980.78	3388.66	37
H27E	1464.52	4703.68	2394.92	37
H27F	1449.88	4783.63	3327.34	37

Table S50. Hydrogen Atom Coordinates (Å×10[4]) and Isotropic Displacement Parameters (Å[2]×10[3]) for Ni complex (**46**)

Atom	*x*	*y*	*z*	U(eq)
H29A	651.53	6311.66	3461.22	41
H31A	-280.67	7675.23	3522.22	72
H32A	-1179.4	8836.48	2851.86	89
H33A	-1464.84	8941.37	1421.19	86
H34A	-872.07	7929.08	651.33	80
H36A	85.9	6576.22	609.84	58
H37A	1035.2	5477.78	1289.86	46
H29B	886.67	5884.46	1518.71	42
H31B	45.9	6987.08	487.09	70
H32B	-843.02	8254.4	199.24	85
H33B	-1318.64	9020.74	1245.48	80
H34B	-976.93	8475.7	2536.41	70
H36B	-211.86	7339.36	3518.81	50
H37B	738.95	6130.15	3845.12	39

Table S51. Atomic Occupancy for Ni complex (**46**)

Atom	*Occupancy*	Atom	*Occupancy*	Atom	*Occupancy*
C20	0.741(14)	H20A	0.741(14)	H20B	0.741(14)
H20C	0.741(14)	C21	0.741(14)	H21A	0.741(14)
H21B	0.741(14)	H21C	0.741(14)	C22	0.741(14)
H22A	0.741(14)	H22B	0.741(14)	H22C	0.741(14)
C20B	0.259(14)	H20D	0.259(14)	H20E	0.259(14)
H20F	0.259(14)	C21B	0.259(14)	H21D	0.259(14)
H21E	0.259(14)	H21F	0.259(14)	C22B	0.259(14)
H22D	0.259(14)	H22E	0.259(14)	H22F	0.259(14)
C24	0.846(4)	H24A	0.846(4)	H24B	0.846(4)
H24C	0.846(4)	C25	0.846(4)	H25A	0.846(4)
H25B	0.846(4)	H25C	0.846(4)	C26	0.846(4)
H26A	0.846(4)	H26B	0.846(4)	H26C	0.846(4)
C24B	0.154(4)	H24D	0.154(4)	H24E	0.154(4)
H24F	0.154(4)	C25B	0.154(4)	H25D	0.154(4)
H25E	0.154(4)	H25F	0.154(4)	C26B	0.154(4)
H26D	0.154(4)	H26E	0.154(4)	H26F	0.154(4)

Table S51. Atomic Occupancy for Ni complex (**46**)

Atom	*Occupancy*	Atom	*Occupancy*	Atom	*Occupancy*
H27C	0.652(4)	H27D	0.652(4)	H27E	0.348(4)
H27F	0.348(4)	C28A	0.652(4)	C29A	0.652(4)
H29A	0.652(4)	C30A	0.652(4)	C31A	0.652(4)
H31A	0.652(4)	C32A	0.652(4)	H32A	0.652(4)
C33A	0.652(4)	H33A	0.652(4)	C34A	0.652(4)
H34A	0.652(4)	C35A	0.652(4)	C36A	0.652(4)
H36A	0.652(4)	C37A	0.652(4)	H37A	0.652(4)
C28B	0.348(4)	C29B	0.348(4)	H29B	0.348(4)
C30B	0.348(4)	C31B	0.348(4)	H31B	0.348(4)
C32B	0.348(4)	H32B	0.348(4)	C33B	0.348(4)
H33B	0.348(4)	C34B	0.348(4)	H34B	0.348(4)
C35B	0.348(4)	C36B	0.348(4)	H36B	0.348(4)
C37B	0.348(4)	H37B	0.348(4)		

Table S52. Solvent masks information for Ni complex (**46**)

Number	X	Y	Z	Volume	Electron count	Content
1	0.000	-0.006	-0.232	680.5	143.1	3 C7H8

X. Compound Characterization

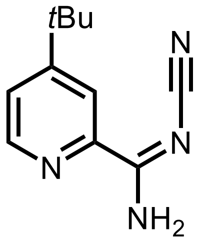

4-(*TERT*-BUTYL)-N-CYANOPICOLINIMIDAMIDE ($^{4\text{-}tBu}$PYCAM^{CN})

An oven-dried 250 mL round-bottom flask equipped with a PTFE-coated stir bar and reflux condenser was charged 4-tert-butyl-2-cyanopyridine (5.00 g, 31.2 mmol, 1.00 equiv) and MeOH (50 mL) under a N_2 atmosphere. To the mixture was added NaOMe (0.167 g, 3.10 mmol, 0.100 equiv). The mixture was heated at reflux for 12 h, then cooled to rt before cyanamide was added in one portion. The resulting mixture was heated at reflux again for 12 h, during which a white precipitate formed. After cooling to rt, the contents of the flask were filtered, and the filter cake was washed with cold MeOH (2 × 10 mL) to afford the product as a white solid (3.64 g, 58% yield).

<u>^{1}H NMR (500 MHz, $CDCl_3$):</u> δ 8.5 (dd, *J* = 5.2, 0.7 Hz, 1H), 8.4 (s, 1H), 8.3 (d, *J* = 1.1 Hz, 1H), 7.5 (dd, *J* = 5.2, 2.0 Hz, 1H), 6.5 (s, 1H), 1.4 (s, 9H).

<u>^{13}C{^{1}H} NMR (126 MHz, $CDCl_3$):</u> δ 166.6, 162.4, 148.8, 147.2, 124.7, 119.8, 116.3, 35.3, 30.6.

<u>HRMS-ESI+ (m/z):</u> $[M+H]^+$ calculated for $C_{11}H_{15}N_4^+$ 203.1291, found 203.1291.

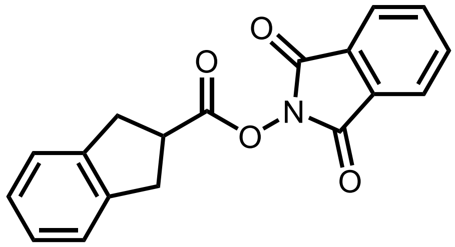

1,3-DIOXOISOINDOLIN-2-YL 2,3-DIHYDRO-1H-INDENE-2-CARBOXYLATE [CAS: 1872262-67-3][4]

Prepared according to the literature procedure.[4] The title compound was purified via column chromatography (eluting with 15% EtOAc in hexanes) to produce a white solid.

^{1}H NMR (400 MHz, $CDCl_3$): δ 7.90 – 7.88 (m, 2H), 7.82 – 7.78 (m, 2H), 7.27 – 7.24 (m, 2H), 7.22 – 7.19 (m, 2H), 3.72 (p, J=8.9 Hz, 1H), 3.52 – 3.42 (m, 4H).

^{13}C{^{1}H} (101 MHz, $CDCl_3$): δ 171.8, 162.2 (2C), 140.7 (2 x Ar-C), 134.9 (2 x Ar-C), 129.1 (2 x Ar-C), 127.1 (2 x Ar-C), 124.6 (2 x Ar-C), 124.1 (2 x Ar-C), 40.6, 36.5 (2C).

HRMS-ESI+ (m/z): $[M+Na]^+$ calculated for $C_{18}H_{13}NO_4Na^+$ 330.0742, found 330.0747.

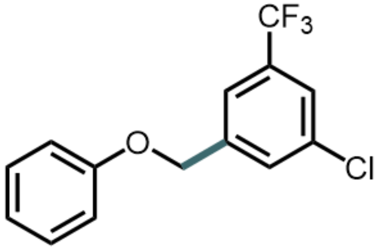

1-CHLORO-3-(PHENOXYMETHYL)-5-(TRIFLUOROMETHYL)BENZENE (1A)

Prepared according to the general procedure for aryl iodide scope in the decarboxylative arylation from 1-chloro-3-iodo-5-(trifluoromethyl)benzene (153.2 mg, 78.5 μL, 0.50 mmol, 1.00 equiv) and 2-phenoxyacetic acid (98.9 mg, 0.65 mmol, 1.30 equiv) in 10.0 mL of 1,4-dioxane. The title compound was isolated via column chromatography (eluting with 100% hexanes) to produce a colorless oil (109 mg, 76% yield).

^{1}H NMR (400 MHz, $CDCl_3$): δ 7.64 (s, 1H), 7.59 (s, 1H), 7.57 (s, 1H), 7.34 -7.30 (m, 2H), 7.03 – 6.96 (m, 3H), 5.08 (s, 2H).

$^{13}C\{^1H\}$ NMR (101 MHz, CDCl$_3$): δ 158.2, 140.4, 135.3, 132.6 (q, $^2J_{C-F}$ = 33.0 Hz), 130.7 (q, $^4J_{C-F}$ = 1.1 Hz), 129.8 (2 x Ar-C), 125.2 (q, $^3J_{C-F}$ = 3.8 Hz), 123.4 (q, $^1J_{C-F}$ = 272.8 Hz), 122.2 (q, $^3J_{C-F}$ = 3.7 Hz), 121.7, 114.9 (2 x Ar-C), 68.5.

^{19}F NMR (377 MHz, CDCl$_3$): δ -62.8

HRMS-EI+ (m/z): [M]$^{\cdot+}$ calculated for $C_{14}H_{10}F_3OCl^+$, 286.0372; found, 286.0383.

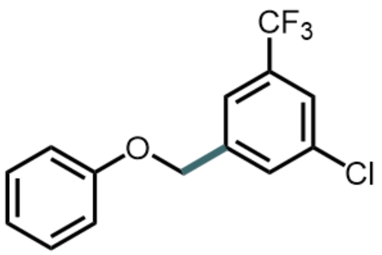

1-CHLORO-3-(PHENOXYMETHYL)-5-(TRIFLUOROMETHYL)BENZENE (1B)
Prepared according to the general procedure for aryl iodide scope in the decarboxylative arylation from 1-chloro-3-bromo-5-(trifluoromethyl)benzene (129.7 mg, 76.3 μL, 0.50 mmol, 1.00 equiv) and 2-phenoxyacetic acid (98.9 mg, 0.65 mmol, 1.30 equiv) with phthalimide (37.0 mg, 0.25 mmol, 0.50 equiv) in 10.0 mL of 1,4-dioxane. The title compound was isolated via column chromatography (eluting with 100% hexanes) to produce a colorless oil (96 mg, 67% yield).

^1H NMR (400 MHz, CDCl$_3$): δ 7.64 (s, 1H), 7.59 (s, 1H), 7.57 (s, 1H), 7.34 -7.30 (m, 2H), 7.03 – 6.95 (m, 3H), 5.09 (s, 2H).

$^{13}C\{^1H\}$ NMR (101 MHz, CDCl$_3$): δ 158.3, 140.5, 135.4, 132.6 (q, $^2J_{C-F}$ = 33.0 Hz), 130.7 (q, $^4J_{C-F}$ = 1.1 Hz), 129.8 (2 x Ar-C), 125.2 (q, $^3J_{C-F}$ = 3.8 Hz), 123.4 (q, $^1J_{C-F}$ = 274.1 Hz), 122.2 (q, $^3J_{C-F}$ = 3.8 Hz), 121.7, 114.9 (2 x Ar-C), 68.6.

^{19}F NMR (377 MHz, CDCl$_3$): δ -62.8

HRMS-EI+ (m/z): [M]$^{\cdot+}$ calculated for $C_{14}H_{10}F_3OCl^+$, 286.0372; found, 286.0379.

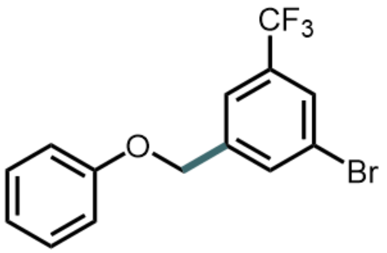

1-BROMO-3-(PHENOXYMETHYL)-5-(TRIFLUOROMETHYL)BENZENE (2)

Prepared according to the general procedure for aryl iodide scope in the decarboxylative arylation from 1-bromo-3-iodo-5-(trifluoromethyl)benzene (175.5 mg, 80.6 µL, 0.50 mmol, 1.00 equiv) and 2-phenoxyacetic acid (98.9 mg, 0.65 mmol, 1.30 equiv) in 10.0 mL of 1,4-dioxane. The title compound was isolated via column chromatography (eluting with 100% hexanes) to produce a colorless oil (111.5 mg, 68% yield).

^{1}H NMR (400 MHz, $CDCl_3$): δ 7.79 (s,1H), 7.73 (s, 1H), 7.64 (s, 1H), 7.34 –7.30 (m, 2H), 7.03 – 6.95 (m, 3H), 5.08 (s, 2H).

^{13}C{^{1}H} (101 MHz, $CDCl_3$): δ 158.3, 140.6, 133.6 (q, $^{4}J_{C-F}$ = 1.1 Hz), 132.7 (q, $^{2}J_{C-F}$ = 33.1 Hz), 129.8 (2 x Ar-C), 128.1(q, $^{3}J_{C-F}$ = 4.0 Hz), 123.2 (q, $^{1}J_{C-F}$ =272.8 Hz), 123.1, 122.8 (q, $^{3}J_{C-F}$ = 3.7 Hz), 121.8, 114.9 (2 x Ar-C), 68.5.

^{19}F NMR (377 MHz, $CDCl_3$): δ -62.8.

HRMS-EI+ (m/z): $[M]^{\cdot+}$ calculated for $C_{14}H_{10}F_3OBr^+$, 329.9867; found, 329.9859.

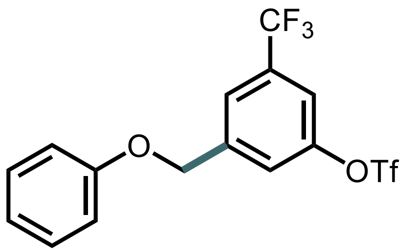

3-(PHENOXYMETHYL)-5-(TRIFLUOROMETHYL)PHENYL TRIFLUOROMETHANESULFONATE (3)

Prepared according to the general procedure for aryl iodide scope in the decarboxylative arylation from 3-bromo-5-(trifluoromethyl)phenyl trifluoromethanesulfonate (186.5 mg, 114.7 µL, 0.50 mmol, 1.00 equiv) and 2-phenoxyacetic acid (98.9 mg, 0.65 mmol, 1.30 equiv) in 10.0 mL of 1,4-dioxane.The title compound was isolated via column chromatography (eluting with 100% hexanes) to produce a colorless oil (192 mg, 96% yield).

^{1}H NMR (400 MHz, CDCl$_3$): δ 7.76 (s, 1H), 7.59 (s,1H), 7.49 (s,1H), 7.35 – 7.31 (m, 2H), 7.04 – 6.96 (m, 3H), 5.16 (s, 2H).

^{13}C{^{1}H} (101 MHz, CDCl$_3$): δ 158.0, 149.7, 141.9, 133.3 (q, $^{2}J_{C\text{-}F}$ = 33.8 Hz), 129.9 (2 x Ar-C), 124.3, 123.8 (q, $^{3}J_{C\text{-}F}$ = 3.8 Hz), 123.5, 122.9 (q, $^{1}J_{C\text{-}F}$ = 272.8 Hz), 122.0, 118.1 (q, $^{3}J_{C\text{-}F}$ = 3.5 Hz), 114.9 (2 x Ar-C), 68.2.

^{19}F NMR (377 MHz, CDCl$_3$): δ -72.7, -62.8.

HRMS-EI+ (m/z): [M]$^{\cdot+}$ calculated for $C_{15}H_{10}F_6O_4S^+$, 400.0204; found, 400.0217.

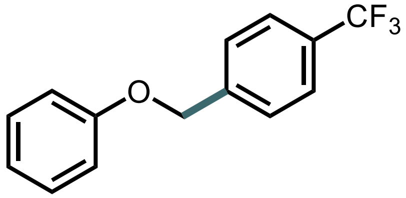

1-(PHENOXYMETHYL)-4-(TRIFLUOROMETHYL)BENZENE (4) [CAS: 19962-24-4][19]

Prepared according to the general procedure for aryl iodide scope in the decarboxylative arylation from 1-iodo-4-(trifluoromethyl)benzene (136.0 mg, 73.0 µL, 0.50 mmol, 1.00 equiv) and 2-phenoxyacetic acid (98.9 mg, 0.65 mmol, 1.30 equiv) with phthalimide (37.0 mg, 0.25 mmol, 0.50 equiv) in 10.0 mL of 1,4-dioxane. The title compound was isolated via column chromatography (eluting with 10% DCM in hexanes) to produce a white solid (116 mg, 92% yield).

^{1}H NMR (400 MHz, CDCl$_3$): δ 7.65 (d, J = 8.1 Hz, 2H), 7.55 (d, J = 8.1 Hz, 2H), 7.32 – 7.29 (m, 2H), 7.01 – 6.96 (m, 3H), 5.13 (s, 2H).

^{13}C{^{1}H} (101 MHz, CDCl$_3$): δ 158.5, 141.3, 130.4 (q, $^{2}J_{C\text{-}F}$= 32.4 Hz), 129.7 (2 x Ar-C), 127.5 (2 x Ar-C), 125.7 (q, $^{3}J_{C\text{-}F}$= 3.8 Hz, 2 x Ar-C), 124.2 (q, $^{1}J_{C\text{-}F}$= 273.2 Hz), 121.4, 114.9 (2 x Ar-C), 69.1.

^{19}F NMR (377 MHz, CDCl$_3$): δ -62.5.

HRMS-EI+ (m/z): [M]$^{\cdot+}$ calculated for $C_{14}H_{11}F_3O^+$, 252.0762; found, 252.0756.

---

[19] Kuwano, R.; Kusano, H. *Org. Lett.* **2008**, *10*, 1979-1982.

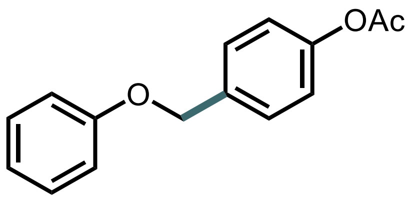

4-(PHENOXYMETHYL)PHENYL ACETATE (5)
Prepared according to the general procedure for aryl iodide scope in the decarboxylative arylation from 4-iodophenyl acetate (131.0 mg, 72.7 µL, 0.50 mmol, 1.00 equiv) and 2-phenoxyacetic acid (98.9 mg, 0.65 mmol, 1.30 equiv) in 10.0 mL of 1,4-dioxane. The title compound was isolated via gradient column chromatography (eluting with 1 - 2% EtOAc in hexanes) to produce a white solid (89.5 mg, 74% yield).

^{1}H NMR (400 MHz, $CDCl_3$): δ 7.45 (d, *J* = 8.6 Hz 2H), 7.31 – 7.27 (m, 2H), 7.12 – 7.10 (d, *J* =8.6 Hz, 2H), 6.99 – 6.95 (m, 3H), 5.05 (s, 2H), 2.31 (s, 3H).

^{13}C{^{1}H} (101 MHz, $CDCl_3$): δ 169.7, 158.8, 150.4, 134.8, 129.7 (2 x Ar-C), 128.7 (2 x Ar-C), 121.9 (2 x Ar-C), 121.2, 114.9 (2 x Ar-C), 69.4, 21.3.

HRMS-EI+ (m/z): $[M+Na]^+$ calculated for $C_{15}H_{14}O_3Na^+$, 265.0841; found, 265.0841.

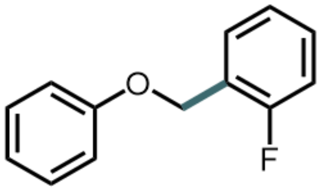

1-FLUORO-2-(PHENOXYMETHYL)BENZENE (6) [CAS: 1460383-72-5][20]
Prepared according to the general procedure for aryl iodide scope in the decarboxylative arylation from 1-fluoro-2-iodobenzene (111.0 mg, 58.4 µL, 0.50 mmol, 1.00 equiv) and 2-phenoxyacetic acid (98.9 mg, 0.65 mmol, 1.30 equiv) with phthalimide (37.0 mg, 0.25 mmol, 0.50 equiv) in 10.0 mL of 1,4-dioxane. The title compound was isolated via column chromatography (eluting with 100% pentane) to produce a colorless oil (66 mg, 65% yield).

^{1}H NMR (400 MHz, $CDCl_3$): δ 7.53 – 7.49 (m, 1H), 7.32 – 7.28 (m, 3H), 7.18 – 7.06 (m, 2H), 7.01 – 6.95 (m, 3H), 5.14 (s, 2H).

---

[20] Wang, H.; Ma, Y.; Tian, H.; Yu, A.; Chang, J.; Wu, Y. *Tetrahedron* **2014**, *70*, 2669-2673.

^{13}C{^{1}H} (101 MHz, CDCl$_3$): δ 160.6 (d, $^1J_{C-F}$ = 246.9 Hz), 158.7, 129.83 (d, $^3J_{C-F}$ = 4.2 Hz), 129.78 (d, $^3J_{C-F}$ = 8.1 Hz), 129.7 (2 x Ar-C), 124.40 (d, $^3J_{C-F}$ = 3.6 Hz), 124.39 (d, $^2J_{C-F}$ = 14.2 Hz), 121.3, 115.5 (d, $^2J_{C-F}$ = 21.3 Hz), 114.9 (2 x Ar-C), 63.7 (d, $^3J_{C-F}$ = 4.4 Hz).

^{19}F NMR (377 MHz, CDCl$_3$): δ -118.8.
HRMS-EI+ (m/z): $[M]^{\cdot+}$ calculated for $C_{13}H_{11}FO^+$, 202.0794; found, 202.0786.

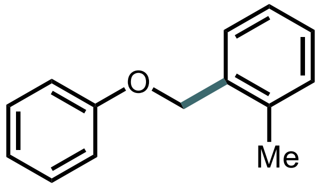

1-METHYL-2-(PHENOXYMETHYL)BENZENE (7) [CAS: 57076-47-8][17]
Prepared according to the general procedure for aryl iodide scope in the decarboxylative arylation from 1-iodo-2-methylbenzene (109.0 mg, 63.6 μL, 0.50 mmol, 1.00 equiv) and 2-phenoxyacetic acid (98.9 mg, 0.65 mmol, 1.30 equiv) in 10.0 mL of 1,4-dioxane. The title compound was isolated via column chromatography (eluting with 100% hexanes) to produce a colorless oil (65.5 mg, 66% yield).

^{1}H NMR (400 MHz, CDCl$_3$): δ 7.42 – 7.40 (m, 1H), 7.32 – 7.28 (m, 2H), 7.25 – 7.20 (m, 3H), 7.01 – 6.95 (m, 3H), 5.03 (s, 2H), 2.37 (s, 3H).

^{13}C{^{1}H} (101 MHz, CDCl$_3$): δ 159.1, 136.9, 135.0, 130.5, 129.6 (2 x Ar-C), 128.8, 128.4, 126.2, 121.1, 114.9 (2 x Ar-C), 68.6, 19.0.

HRMS-EI+ (m/z): $[M]^{\cdot+}$ calculated for $C_{14}H_{14}O^+$, 198.1045; found, 198.1051.

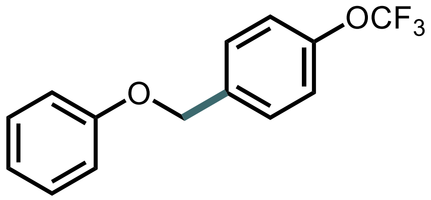

1-(PHENOXYMETHYL)-4-(TRIFLUOROMETHOXY)BENZENE (8) [CAS: 1456351-39-5][21]

---

[21] Chao, H.J.; Tuerdi, H.; Herpin, T.; Roberge, J.Y.; Liu, Y.; Lawrence, M.R.; Rehfuss, R.P.; Clark, C.G.; Qiao, J.X.; Gungor, T.; Lam, P.Y.S.; Wang, T.C.; Ruel, R.; L Heureux, A.L.; Thibeault, C.; Bouthillier, G.; Schnur, D.M. Urea antagonists of P2Y1 receptor useful in the treatment of thrombotic conditions EP1751113A1 **2007**.

Prepared according to the general procedure for aryl iodide scope in the decarboxylative arylation from 1-iodo-4-(trifluoromethoxy)benzene (144.0 mg, 78.3 μL, 0.50 mmol, 1.00 equiv) and 2-phenoxyacetic acid (98.9 mg, 0.65 mmol, 1.30 equiv) with phthalimide (37.0 mg, 0.25 mmol, 0.50 equiv) in 10.0 mL of 1,4-dioxane. The title compound was isolated via column chromatography (eluting with 100% pentane) to produce a colorless oil (110 mg, 82% yield).

^{1}H NMR (400 MHz, $CDCl_3$): δ 7.48 – 7.45 (d, J = 8.6 Hz, 2H), 7.33 – 7.28 (m, 2H), 7.25 (d, J = 8.6 Hz, 2H), 7.00 – 6.95 (m, 3H), 5.1 (s, 2H).

^{13}C{^{1}H} (101 MHz, $CDCl_3$): δ 158.6, 149.0 (q, $^{3}J_{C-F}$ = 1.8 Hz), 135.9, 129.7 (2 x Ar-C), 129.0 (2 x Ar-C), 121.3, 121.3 (d, $^{4}J_{C-F}$ = 1.0 Hz, 2 x Ar-C), 120.6 (d, $^{1}J_{C-F}$ = 257.2 Hz), 114.9 (2 x Ar-C), 69.1.

^{19}F NMR (377 MHz, $CDCl_3$): δ -57.9.

HRMS-EI+ (m/z): $[M]^{\cdot+}$ calculated for $C_{14}H_{11}F_3O_2^+$, 268.0711; found, 268.0705.

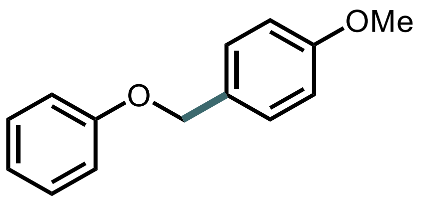

1-METHOXY-4-(PHENOXYMETHYL)BENZENE (9) [CAS: 19962-28-8][22]

Prepared according to the general procedure for aryl iodide scope in the decarboxylative arylation from 1-iodo-4-methoxybenzene (117.0 mg, 0.50 mmol, 1.00 equiv) and 2-phenoxyacetic acid (98.9 mg, 0.65 mmol, 1.30 equiv) with phthalimide (37.0 mg, 0.25 mmol, 0.50 equiv) in 10.00 mL of 1,4-dioxane. The title compound was isolated via column chromatography (eluting with 12% DCM in hexanes) to produce a pale yellow solid (80 mg, 75% yield).

^{1}H NMR (400 MHz, $CDCl_3$): δ 7.37 (d, J = 8.7 Hz, 2H), 7.31 – 7.27 (m, 2H), 6.99 – 6.96 (m, 3H), 6.95 – 6.91 (m, 2H), 4.99 (s, 2H), 3.82 (s, 3H).

^{13}C{^{1}H} (101 MHz, $CDCl_3$): δ 159.6, 159.0, 129.6 (2 x Ar-C), 129.4 (2 x Ar-C), 129.2, 121.0, 114.9 (2 x Ar-C), 114.1 (2 x Ar-C), 69.8, 55.5.

HRMS-EI+ (m/z): $[M]^{\cdot+}$ calculated for $C_{14}H_{14}O_2^+$, 214.0994; found, 214.1002.

---

[22] Iiangovan, A.; Anandhan, K.; Kaushik, M.P. *Tetrahedron Lett.* **2015**, *56*, 1080-1084.

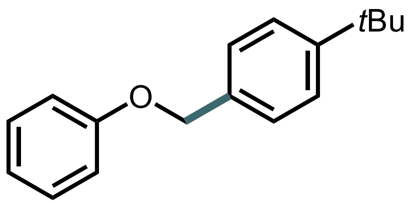

1-(*TERT*-BUTYL)-4-(PHENOXYMETHYL)BENZENE (10) [CAS: 101747-17-5][23]
Prepared according to the general procedure for aryl iodide scope in the decarboxylative arylation from 4-*tert*-butyliodobenzene (130.0 mg, 88.6 μL, 0.50 mmol, 1.00 equiv) and 2-phenoxyacetic acid (98.9 mg, 0.65 mmol, 1.30 equiv) with phthalimide (37.0 mg, 0.25 mmol, 0.50 equiv) in 10.0 mL of 1,4-dioxane. The title compound was isolated via gradient column chromatography (eluting with 0-1% EtOAc in hexanes) to produce a colorless oil (63.6 mg, 53% yield).

^{1}H NMR (400 MHz, $CDCl_3$): δ 7.43 – 7.37 (m, 4H), 7.32 – 7.28 (m, 2H), 6.98 – 6.94 (m, 3H), 5.03 (s, 2H), 1.33 (s, 9H).

$^{13}C\{^1H\}$ (101 MHz, $CDCl_3$): δ 159.0, 151.2, 134.1, 129.6 (2 x Ar-C), 127.6 (2 x Ar-C), 125.7 (2 x Ar-C), 121.0, 114.9 (2 x Ar-C), 69.9, 34.7, 31.5 (3 C)

HRMS-EI+ (m/z): $[M]^{\cdot+}$ calculated for $C_{17}H_{20}O^+$, 240.1514; found, 240.1519.

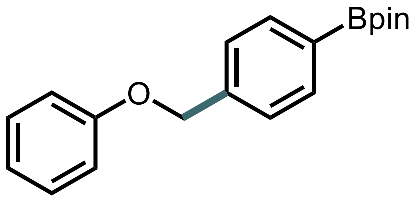

4,4,5,5-TETRAMETHYL-2-(4-(PHENOXYMETHYL)PHENYL)-1,3,2-DIOXABOROLANE (11) [CAS: 946409-21-8][24]
Prepared according to the general procedure for aryl iodide scope in the decarboxylative arylation from 2-(4-iodophenyl)-4,4,5,5-tetramethyl-1,3,2-dioxaborolane (164.9 mg, 0.50 mmol, 1.00 equiv) and 2-phenoxyacetic acid (98.9 mg, 0.65 mmol, 1.30 equiv) in 10.0 mL of 1,4-dioxane. The title compound was isolated via gradient column chromatography (eluting with 0 - 1% EtOAc in hexanes) to produce a colorless oil (138.5 mg, 89% yield).

^{1}H NMR (400 MHz, $CDCl_3$): δ 7.82 (d, *J* = 8.0 Hz, 2H), 7.43 (d, *J* = 8.0 Hz, 2H), 7.30 – 7.25 (m, 2H), 6.97 – 6.95 (m, 3H), 5.09 (s, 2H), 1.34 (s, 12H).

---

[23] Huy, P.H.; Motsch, S.; Kappler, S.M. *Angew. Chem. Int. Ed.* **2016**, *55*, 10145 -10149.
[24] Spencer, J.; Baltus, C.B.; Patel, H.; Press, N.J.; Callear, S.K.; Male, L.; Coles, S.J. *ACS Comb. Sci.* **2011**, *13*, 24-31.

$^{13}C\{^1H\}$ (101 MHz, $CDCl_3$): δ 158.8, 140.4, 135.2 (2 x Ar-C), 129.6 (2 x Ar-C, *1 Carbon Overlap*), 126.6 (2 x Ar-C), 121.1, 115.0 (2 x Ar-C), 84.0 (2 C), 69.9, 25.0 (4 C).

^{11}B (128 MHz, $CDCl_3$): δ 31.0

HRMS-ASAP+ (m/z): $[M+H]^+$ calculated for $C_{19}H_{24}O_3B^+$, 311.1819; found, 311.1818.

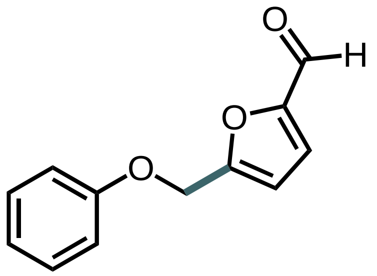

5-(PHENOXYMETHYL)FURAN-2-CARBALDEHYDE (12) [CAS: 685904-18-1][25]

Prepared according to the general procedure for aryl iodide scope in the decarboxylative arylation from 5-iodofuran-2-carbaldehyde (111.0 mg, 0.50 mmol, 1.00 equiv) and 2-phenoxyacetic acid (98.9 mg, 0.65 mmol, 1.30 equiv) with phthalimide (37.0 mg, 0.25 mmol, 0.50 equiv) in 10.00 mL of MeCN. The title compound was isolated via gradient column chromatography (eluting with 10-20% EtOAc in hexanes) to produce a colorless solid (45.9 mg, 45% yield).

^1H NMR (400 MHz, $CDCl_3$): δ 9.65 (s, 1H), 7.33 – 7.29 (m, 2H), 7.24 – 7.23 (m, 1H), 7.03 – 6.95 (m, 3H), 6.63 – 6.61 (d, J = 3.5 Hz, 1H), 5.11 (s, 2H).

$^{13}C\{^1H\}$ (101 MHz, $CDCl_3$): δ 177.9, 158.0, 157.0, 152.8, 129.8 (2 x Ar-C), 122.1, 121.9, 114.9 (2 x Ar-C), 111.7, 62.6.

HRMS-ESI+ (m/z): $[M+H]^+$ calculated for $C_{12}H_{11}O_3^+$, 203.0708; found, 203.0706.

[25] Chang, F.; Dutta, S.; Becnel, J.J.; Estep, A.S.; Mascal, M. *J. Agric. Food Chem.* **2014**, *62*, 476-480.

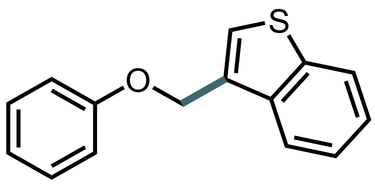

3-(PHENOXYMETHYL)BENZO[B]THIOPHENE (13)

Prepared according to the general procedure for aryl iodide scope in the decarboxylative arylation from 3-iodobenzo[b]thiophene (130.0 mg, 68.5 μL, 0.50 mmol, 1.00 equiv) and 2-phenoxyacetic acid (98.9 mg, 0.65 mmol, 1.30 equiv) in 15.0 mL of MeCN. The title compound was isolated via column chromatography (eluting with 2% EtOAc in hexanes) to produce a colorless solid (88 mg, 73% yield). Our NMR data did not match those reported in the literature.[26] To confirm the connectivity of the molecule through XRD analysis, 50 mg of pure product was dissolved in 1.00 mL of DCM followed by 0.500 mL of hexanes and subjected to slow evaporation resulting in colorless plate-shaped crystals.

^{1}H NMR (400 MHz, $CDCl_3$): δ 7.90 – 7.85 (m, 2H), 7.51 – 7.50 (s, 1H), 7.42 – 7.31 (m, 4H), 7.05 – 6.98 (m, 3H), 5.31 (s, 2H).

^{13}C{^{1}H} (101 MHz, $CDCl_3$): δ 158.8, 140.8, 138.0, 132.0, 129.7 (2 x Ar-C), 125.4, 124.8, 124.5, 123.0, 122.2, 121.3, 115.0 (2 x Ar-C), 64.9.

HRMS-EI+ (m/z): $[M]^{\cdot+}$ calculated for $C_{15}H_{12}OS^+$, 240.0609; found, 240.0612.

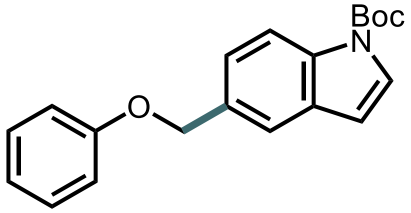

TERT-BUTYL-5-(PHENOXYMETHYL)-1H-INDOLE-1-CARBOXYLATE (14)

Prepared according to the general procedure for aryl iodide scope in the decarboxylative arylation from 5-iodo-1H-indole-1-carboxylate (171.6 mg, 0.50 mmol, 1.00 equiv) and 2-phenoxyacetic acid (98.9 mg, 0.65 mmol, 1.30 equiv) with phthalimide (37.0 mg, 0.25 mmol, 0.50 equiv) in 10.0 mL of MeCN. The title compound was isolated via gradient column chromatography (eluting with 1-2% ether in hexanes) to produce a colorless oil (75.4 mg, 47% yield).

[26] Tang, Y.; Zhuang, K.; Zhang, X.; Xie, F.; Yang, L.; Lin, B.; Cheng, M.; Li, D.; Liu, Y. *Eur. J. Org. Chem.* **2020**, *23*, 3441-3451.

^{1}H NMR (400 MHz, CDCl$_3$): δ 8.14 (d, *J* = 8.5 Hz, 1H), 7.63 (d, *J* = 1.73 Hz, 1H), 7.60 (d, *J* =3.7 Hz, 1H), 7.37 (m, *J* = 8.5, 1.7 Hz, 1H), 7.31 – 7.27 (m, 2H), 7.02 – 6.93 (m, 3H), 6.56 (d, *J* = 3.7, 0.8 Hz, 1H), 5.16 (s, 2H), 1.67 (s, 9H).

^{13}C{^{1}H} (101 MHz, CDCl$_3$): δ 159.0, 149.8, 135.0, 131.5, 130.9, 129.6 (2 x Ar-C), 126.6, 124.1, 120.9, 120.3, 115.4, 115.1 (2 x Ar-C), 107.4, 83.9, 70.4, 28.3 (3C).

HRMS-EI+ (m/z): $[M]^{\cdot+}$ calculated for $C_{20}H_{21}NO_3^+$, 323.1522; found, 323.1525.

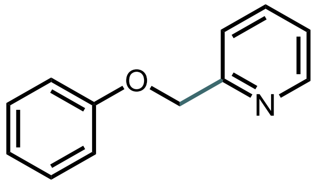

2-(PHENOXYMETHYL)PYRIDINE (15) [CAS: 104294-19-1][27]

Prepared according to the general procedure for aryl iodide scope in the decarboxylative arylation from 2-iodopyridine (102.5 mg, 53.2 μL, 0.50 mmol, 1.00 equiv) and 2-phenoxyacetic acid (98.9 mg, 0.65 mmol, 1.30 equiv) with phthalimide (37.0 mg, 0.25 mmol, 0.50 equiv) in 10.0 mL of 1,4-dioxane. The title compound was isolated via column chromatography (eluting with 15% EtOAc in hexanes) to produce a brown-colored oil (55.6 mg, 60% yield).

^{1}H NMR (400 MHz, CDCl$_3$): δ 8.61 – 8.59 (m, 1H), 7.74 – 7.69 (m, 1H), 7.54 – 7.52 (m, 1H), 7.32 – 7.27 (m, 2H), 7.24 – 7.21 (m, 1H), 7.01 – 6.95 (m, 3H), 5.22 (s, 2H).

^{13}C{^{1}H} (101 MHz, CDCl$_3$): δ 158.5, 157.5, 149.4, 136.9, 129.7 (2 x Ar-C), 122.8, 121.4, 121.3, 114.9 (2 x Ar-C), 70.6.

HRMS-ESI+ (m/z): $[M+H]^+$ calculated for $C_{12}H_{12}NO^+$, 186.0919; found, 186.0923.

[27] Cong, X.; You, J.; Gao, G.; Lan, J. *Chem. Commun.* **2013**, *49*, 662-664.

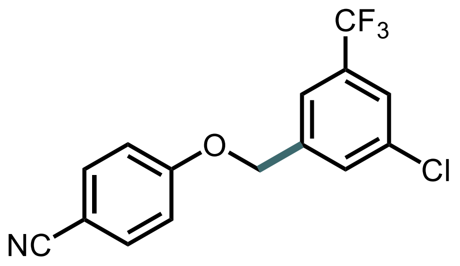

4-((3-CHLORO-5-(TRIFLUOROMETHYL)BENZYL)OXY)BENZONITRILE (21A)
Prepared according to the general procedure for carboxylic acid scope in the decarboxylative arylation from 1-chloro-3-iodo-5-(trifluoromethyl)benzene (153.2 mg, 78.5 µL, 0.50 mmol, 1.00 equiv) and 2-(4-cyanophenoxy)acetic acid (115.2 mg, 0.65 mmol, 1.30 equiv) in 10.0 mL of 1,4-dioxane. The title compound was isolated via column chromatography (eluting with 6% EtOAc in hexanes) to produce a white solid (109 mg, 70% yield).

^{1}H NMR (400 MHz, $CDCl_3$): δ 7.65 – 7.60 (m, 4H), 7.58 – 7.57 (s, 1 H), 7.05 – 7.01 (m, 2H), 5.13 (s, 2H).

$^{13}C\{^1H\}$ (101 MHz, $CDCl_3$): δ 161.4, 139.0, 135.6, 134.3 (2 x Ar-C), 132.8 (q, $^2J_{C-F}$ = 33.3 Hz), 130.7, 125.7 (q, $^3J_{C-F}$ = 3.8 Hz), 123.2 (q, $^1J_{C-F}$ = 272.9 Hz), 122.3 (q, $^3J_{C-F}$ = 3.7 Hz), 119.0, 115.6 (2 x Ar-C), 105.2, 68.8.

^{19}F NMR (377 MHz, $CDCl_3$): δ -62.9.

HRMS-ASAP+ (m/z): $[M+H]^+$ calculated for $C_{15}H_{10}NOClF_3^+$, 312.0403; found, 312.0398.

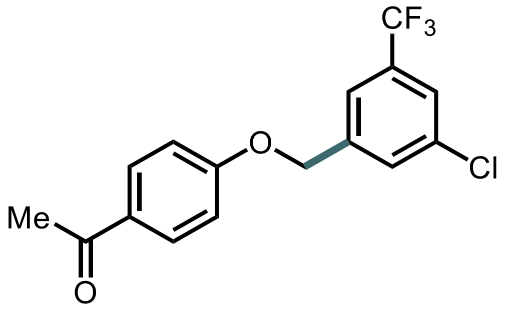

1-(4-((3-CHLORO-5-(TRIFLUOROMETHYL)BENZYL)OXY)PHENYL)ETHAN-1-ONE (21B)
Prepared according to the general procedure for carboxylic acid scope in the decarboxylative arylation from 1-chloro-3-iodo-5-(trifluoromethyl)benzene (153.2 mg, 78.5 µL, 0.50 mmol, 1.00 equiv) and 2-(4-acetylphenoxy)acetic acid (126.2 mg, 0.65 mmol, 1.30 equiv) in 10.00 mL of 1,4-dioxane. The title compound was isolated via gradient column chromatography (eluting with 10-15 % ether in hexanes) to produce a white solid (124.9 mg, 76% yield).

^{1}H NMR (400 MHz, $CDCl_3$): δ 7.98 – 7.95 (m, 2H), 7.64 (s, 1H), 7.60 – 7.59 (m, 2H), 7.02 – 7.00 (m, 2H), 5.16 (s, 2H), 2.58 (s, 3H).

^{13}C{^{1}H} (101 MHz, $CDCl_3$): δ 196.8, 162.0, 139.5, 135.5, 132.8 (q, $^2J_{C-F}$ = 33.1 Hz), 131.3, 130.9 (2 x Ar-C), 130.7 (q, $^4J_{C-F}$ = 1.2 Hz), 125.5 (q, $^3J_{C-F}$ = 3.9 Hz), 123.3 (q, $^1J_{C-F}$ = 272.8 Hz), 122.3 (q, $^3J_{C-F}$ = 3.8 Hz), 114.6 (2 x Ar-C), 68.7, 26.5.

^{19}F{^{1}H} NMR (377 MHz, $CDCl_3$): δ -62.8.

HRMS-ASAP+ (m/z): $[M+H]^+$ calculated for $C_{16}H_{13}O_2F_3Cl^+$, 329.0556; found, 329.0551.

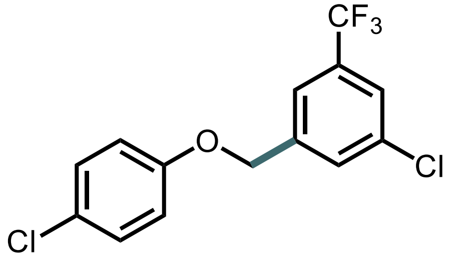

1-CHLORO-3-((4-CHLOROPHENOXY)METHYL)-5-(TRIFLUOROMETHYL)BENZENE (21C)

Prepared according to the general procedure for carboxylic acid scope in the decarboxylative arylation from 1-chloro-3-iodo-5-(trifluoromethyl)benzene (153.2 mg, 78.5 μL, 0.50 mmol, 1.00 equiv) and 2-(4-chlorophenoxy)acetic acid (121.3 mg, 0.65 mmol, 1.30 equiv) in 10.0 mL of 1,4-dioxane. The title compound was isolated via column chromatography (eluting with 100% pentane) to produce a colorless oil (125 mg, 78% yield).

^{1}H NMR (400 MHz, $CDCl_3$): δ 7.62 (s, 1H), 7.58 (s, 1H), 7.57 (s, 1H), 7.29 – 7.25 (m, 2H), 6.92 – 6.88 (m, 2H), 5.06 (s, 2H).

^{13}C{^{1}H} (101 MHz, $CDCl_3$): δ 156.8, 139.9, 135.4, 132.7 (q, $^2J_{C-F}$ = 33.4 Hz), 130.7, 129.7 (2 x Ar-C), 126.7, 125.4 (q, $^3J_{C-F}$ = 3.7 Hz), 123.3 (q, $^1J_{C-F}$ = 272.8 Hz), 122.2 (q, $^3J_{C-F}$ = 3.8 Hz), 116.2 (2 x Ar-C), 68.8.

^{19}F NMR (377 MHz, $CDCl_3$): δ -62.8.

HRMS-ASAP- (m/z): $[M-H]^-$ calculated for $C_{14}H_8OF_3Cl_2^+$, 318.9904; found, 318.9906.

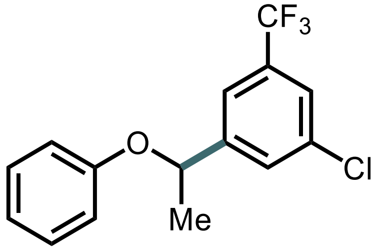

1-CHLORO-3-(1-PHENOXYETHYL)-5-(TRIFLUOROMETHYL)BENZENE (22)

Prepared according to the general procedure for carboxylic acid scope in the decarboxylative arylation from 1-chloro-3-iodo-5-(trifluoromethyl)benzene (153.2 mg, 78.5 μL, 0.50 mmol, 1.00 equiv) and 2-phenoxypropanoic acid (108 mg, 0.65 mmol, 1.30 equiv) in 10.0 mL of 1,4-dioxane. The title compound was isolated via column chromatography (eluting with 100% hexanes) to produce a colorless oil (95 mg, 63% yield).

<u>^{1}H NMR (400 MHz, $CDCl_3$):</u> δ 7.75 (s, 1H), 7.53 (s, 1H), 7.52 (s, 1H), 7.27 -7.20 (m, 2H), 6.95 – 6.92 (m, 1H), 6.87 – 6.83 (m, 2H), 5.33 (q, 1H), 1.63 (d, 3H).

<u>^{13}C{^{1}H} NMR (101 MHz, $CDCl_3$):</u> δ 157.3, 146.6, 135.3, 132.5 (q, $^{2}J_{C-F}$ = 33.0 Hz), 129.6 (2 x Ar-C), 129.1, 124.8 (q, $^{3}J_{C-F}$ = 3.8 Hz), 123.2 (q, $^{1}J_{C-F}$ = 272.8 Hz), 121.8, 120.7 (q, $^{3}J_{C-F}$ = 3.7 Hz), 115.9 (2 x Ar-C), 74.8, 24.2.

<u>^{19}F NMR (377 MHz, $CDCl_3$):</u> δ -62.8.

<u>HRMS-EI+ (m/z):</u>$[M]^{\cdot+}$ calculated for $C_{15}H_{12}OF_3Cl^+$, 300.0529; found, 300.0524.

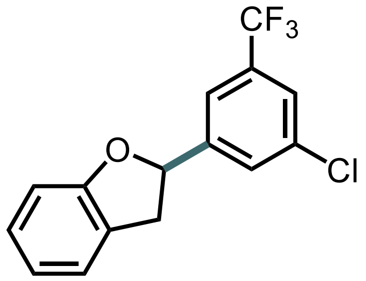

2-(3-CHLORO-5-(TRIFLUOROMETHYL)PHENYL)-2,3-DIHYDROBENZOFURAN (23)

Prepared according to the general procedure for carboxylic acid scope in the decarboxylative arylation from 1-chloro-3-iodo-5-(trifluoromethyl)benzene (153.2 mg, 0.50 mmol, 1.00 equiv) and 2,3-dihydrobenzofuran-2-carboxylic acid (106.7 mg, 0.65 mmol, 1.30 equiv) in 10.00 mL of 1,4-dioxane. The title compound was isolated via column chromatography (eluting with 100% hexanes) to produce a colorless oil (105.5 mg, 70% yield).

^{1}H NMR (400 MHz, $CDCl_3$): δ 7.59 (s, 1H), 7.56 (s, 1H), 7.54 (s, 1H), 7.21 – 7.18 (m, 2H), 6.94 – 6.90 (m, 2H), 5.80 – 5.75 (t, *J* = 9.6 Hz, 1H), 3.67 – 3.73 (dd, *J* = 15.6, 9.6 Hz, 1H), 3.20 – 3.14 (dd, *J* = 15.6, 8.0 Hz, 1H).

^{13}C{^{1}H} (101 MHz, $CDCl_3$): δ 159.3, 145.5, 135.5, 132.7 (q, $^{2}J_{C-F}$ = 33.0 Hz), 129.3, 128.7, 125.6, 125.2 (q, $^{3}J_{C-F}$ = 3.8 Hz), 125.1, 123.3 (q, $^{1}J_{C-F}$ = 272.9 Hz) 121.4, 120.9 (q, $^{3}J_{C-F}$ = 3.7 Hz), 109.8, 82.4, 38.6.

^{19}F NMR (377 MHz, $CDCl_3$): δ -62.8.

HRMS-ESI+ (m/z): $[M+H]^+$ calculated for $C_{15}H_{11}F_3OCl^+$, 299.0451; found, 299.0445.

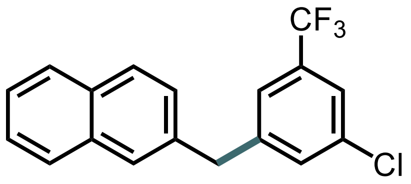

2-(3-CHLORO-5-(TRIFLUOROMETHYL)BENZYL)NAPHTHALENE (24)

Prepared according to the general procedure for carboxylic acid scope in the decarboxylative arylation from 1-chloro-3-iodo-5-(trifluoromethyl)benzene (153.2 mg, 78.5 μL, 0.50 mmol, 1.00 equiv) and 2-(naphthalen-2-yl)acetic acid (121.0 mg, 0.65 mmol, 1.30 equiv) with phthalimide (37.0 mg, 0.25 mmol, 0.50 equiv) in 10.0 mL of 1,4-dioxane. The title compound was isolated via column chromatography (eluting with 100% hexanes) to produce a colorless oil (91.4 mg, 57% yield).

^{1}H NMR (400 MHz, $CDCl_3$): δ 7.84 – 7.79 (m, 3H), 7.63 (s, 1H), 7.51 – 7.44 (m, 3H), 7.39 – 7.37 (d, *J* = 5.4, 2H), 7.28 (d, *J* = 9.7 Hz, 1H), 4.17 (s, 2H).

^{13}C{^{1}H} (101 MHz, $CDCl_3$): δ 144.2, 136.6, 135.1, 133.7, 132.6 (q, $^{4}J_{C-F}$ = 1.3 Hz), 132.4, 128.8, 127.9, 127.8, 127.6, 127.3, 126.5, 126.0, 124.8, 124.1 (q, $^{3}J_{C-F}$ = 3.7 Hz), 123.7 (q, $^{3}J_{C-F}$ = 3.7 Hz), 123.5 (d, $^{1}J_{C-F}$ = 273.1 Hz), 41.7.

^{19}F NMR (377 MHz, $CDCl_3$): δ -62.7.

HRMS-ESI+ (m/z): $[M]^{\cdot+}$ calculated for $C_{18}H_{12}F_3Cl^+$, 320.0580; found, 320.0579.

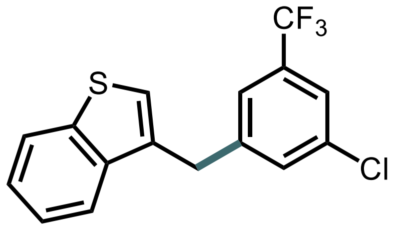

3-(3-CHLORO-5-(TRIFLUOROMETHYL)BENZYL)BENZO[B]THIOPHENE (25)

Prepared according to the general procedure for carboxylic acid scope in the decarboxylative arylation from 1-chloro-3-iodo-5-(trifluoromethyl)benzene (153.2 mg, 78.5 μL, 0.50 mmol, 1.00 equiv) and 2-(benzo[b]thiophen-3-yl)acetic acid (124.9 mg, 0.65 mmol, 1.30 equiv) with phthalimide (37.0 mg, 0.25 mmol, 0.50 equiv) in 10.0 mL of 1,4-dioxane. The title compound was isolated via column chromatography (eluting with 1.5% EtOAc in hexanes) to produce a colorless oil (98 mg, 60% yield).

^{1}H NMR (400 MHz, CDCl$_3$): δ 7.90 – 7.86 (m, 1H), 7.65 – 7.60 (m, 1H), 7.49 – 7.48 (d, J = 1.9 Hz, 1H), 7.43 (s, 1H), 7.40 – 7.34 (m, 3H), 7.06 (d, J = 1.1 Hz, 1H), 4.22 (s, 2H).

^{13}C{^{1}H} (101 MHz, CDCl$_3$): δ 142.7, 140.8, 138.4, 135.2, 133.6, 132.5 (q, $^{2}J_{C\text{-}F}$ = 33.0 Hz), 132.3 (q, $^{4}J_{C\text{-}F}$ = 1.3 Hz), 124.7, 124.4, 124.1, 124.0 (q, $^{3}J_{C\text{-}F}$ = 3.8 Hz), 123.9 (q, $^{3}J_{C\text{-}F}$ = 3.7 Hz), 123.4 (q, $^{1}J_{C\text{-}F}$ = 272.9 Hz), 123.2, 121.7, 34.6.

^{19}F NMR (377 MHz, CDCl$_3$): δ -62.7.

HRMS-EI+ (m/z): $[M]^{\cdot+}$ calculated for $C_{16}H_{10}F_3ClS^+$, 326.0144; found, 326.0158.

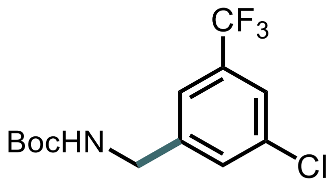

TERT-BUTYL (3-CHLORO-5-(TRIFLUOROMETHYL)BENZYL)CARBAMATE (26)

Prepared according to the general procedure for carboxylic acid scope in the decarboxylative arylation from 1-chloro-3-iodo-5-(trifluoromethyl)benzene (153.2 mg, 78.5 μL, 0.50 mmol, 1.00 equiv) and (*tert*-butoxycarbonyl)glycine (113.9 mg, 0.65 mmol, 1.30 equiv) with phthalimide (37.0 mg, 0.25 mmol, 0.50 equiv) in 10.0 mL of 1,4-dioxane. The title compound was isolated via column chromatography (eluting with 10% EtOAc in hexanes) to produce a colorless oil (108.5 mg, 70% yield).

^{1}H NMR (400 MHz, $CDCl_3$): δ 7.49 (s, 1H), 7.46 (s, 1H), 7.41 (s, 1H), 5.02 (s, 1H), 4.35 – 4.34 (d, J = 6.2 Hz, 2H), 1.46 (s, 9H).

^{13}C{^{1}H} (101 MHz, $CDCl_3$): δ 156.0, 142.5, 135.3, 132.5 (q, $^{2}J_{C-F}$ = 33.0 Hz), 130.8, 124.6 (q, $^{3}J_{C-F}$ = 3.8 Hz), 123.4 (q, $^{1}J_{C-F}$ = 272.9 Hz), 122.3 (q, $^{3}J_{C-F}$ = 3.7 Hz), 80.3, 43.9, 28.5 (3C).

^{19}F NMR (377 MHz, $CDCl_3$): δ -62.9.

HRMS-CI+ (m/z): $[M+H]^+$ calculated for $C_{13}H_{16}NF_3O_2Cl^+$, 310.0822; found, 310.0814.

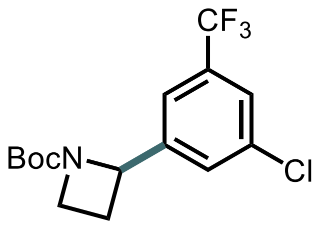

TERT-BUTYL-2-(3-CHLORO-5-(TRIFLUOROMETHYL)PHENYL)AZETADINE-1-CARBOXYLATE (27)
Prepared according to the general procedure for carboxylic acid scope in the decarboxylative arylation from 1-chloro-3-iodo-5-(trifluoromethyl)benzene (153.2 mg, 78.5 μL, 0.50 mmol, 1.00 equiv) and 1-(tert-butoxycarbonyl)azetidine-2-carboxylic acid (130.7 mg, 0.65 mmol, 1.30 equiv) in 10.0 mL of 1,4-dioxane. The title compound was isolated via column chromatography (eluting with 15% EtOAc in hexanes) to produce a colorless oil (122.5 mg, 73% yield).

^{1}H NMR (400 MHz, $CDCl_3$): δ 7.52 (s, 1H), 7.50 (s, 1H), 7.47 (s, 1H), 5.20 (dd, J= 6.44 Hz, 8.82 Hz, 1H), 4.05 – 3.92 (m, 2H), 2.69 – 2.60 (m, 1H), 2.15 – 2.07 (m, 1H), 1.35 (s, 9H).

^{13}C{^{1}H} NMR (101 MHz, $CDCl_3$): δ 156.6, 145.8, 135.2, 132.4 (q, $^{2}J_{C-F}$ = 33.0 Hz), 129.5, 123.3 (q, $^{1}J_{C-F}$ = 273 Hz), 123.5 (q, $^{3}J_{C-F}$ = 3.7 Hz), 121.1 (q, $^{3}J_{C-F}$ = 3.7 Hz), 80.2, 63.1, 46.5, 28.2 (3C), 25.1.

^{19}F NMR (377 MHz, $CDCl_3$): δ -62.8.

HRMS-EI+ (m/z): $[M]^+$ calculated for $C_{13}H_{16}NF_3O_2Cl^+$, 310.0822; found, 310.0814.

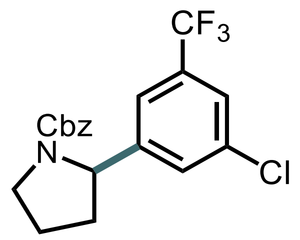

BENZYL-2-(3-CHLORO-5-(TRIFLUOROMETHYL)PHENYL)PYRROLIDINE-1-CARBOXYLATE (28)
Prepared according to the general procedure for carboxylic acid scope in the decarboxylative arylation from 1-chloro-3-iodo-5-(trifluoromethyl)benzene (153.2 mg, 78.5 µL, 0.50 mmol, 1.00 equiv) and ((benzyloxy)carbonyl)-D-proline (162.3 mg, 0.65 mmol, 1.30 equiv) in 10.0 mL of 1,4-dioxane. The title compound was isolated via column chromatography (eluting with 10% EtOAc in hexanes) to produce a colorless oil (125 mg, 65% yield).

^{1}H NMR (400 MHz, $CDCl_3$): δ 7.47 (s, 1H), 7.38 – 7.29 (m, 4H), 7.22 -7.21 (m, 2H), 6.94 – 6.92 (m, 1H), 5.14 – 4.88 (m, 3H), 3.73 – 3.65 (m, 2H), 2.42 – 2.35 (m, 1H), 1.96 – 1.89 (m, 2H), 1.87 – 1.82 (m, 1H).

^{13}C{^{1}H} (201 MHz, $CDCl_3$): mixture of rotamers; δ 155.1, 154.9, 147.9, 147.1, 136.8, 136.3, 135.2, 132.4 (q, $^2J_{C\text{-}F}$ = 32.9 Hz), 129.2, 128.7, 128.6, 128.5, 128.2, 128.1, 128.0, 127.6, 125.5, 125.4, 124.3, 124.2, 124.1, 124.0, 122.75, 122.69, 121.3, 120.8, 67.2, 67.1, 61.0, 60.7, 47.9, 47.4, 36.0, 34.85, 23.83, 23.3.

^{19}F NMR (377 MHz, $CDCl_3$): δ -62.7.

HRMS-ESI+ (m/z): $[M+H]^+$ calculated for $C_{15}H_{17}F_3ClO_2N^+$, 335.0899; found, 335.0897.

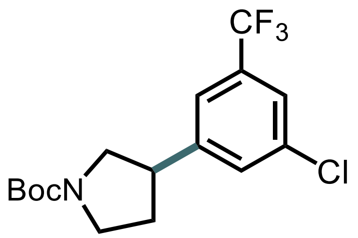

TERT-BUTYL 3-(3-CHLORO-5-(TRIFLUOROMETHYL)PHENYL)PYRROLIDINE-1-CARBOXYLATE (29)

Prepared according to the general procedure for carboxylic acid scope in the decarboxylative arylation from 1-chloro-3-iodo-5-(trifluoromethyl)benzene (153.2 mg, 78.5 µL, 0.50 mmol, 1.00 equiv) and (*tert*-butoxycarbonyl)pyrrolidine-3-carboxylic acid (139.9 mg, 0.65 mmol, 1.30 equiv) using Na_2CO_3 (105.9 mg, 1 mmol, 2.00 equiv) in 10.0 mL of MeCN. The title compound was isolated via column chromatography (eluting with 10% EtOAc in hexanes) to produce a white solid (93 mg, 53% yield).

^{1}H NMR (400 MHz, $CDCl_3$): δ 7.49 (s, 1H), 7.41 (s, 1H), 7.37 (s, 1H), 3.86 – 3.30 (*overlapping proton,* m, 5H), 2.34 – 2.27 (m, 1H), 2.03 – 1.93 (m, 1H), 1.49 (s, 9H).

^{13}C{^{1}H} (201 MHz, $CDCl_3$): mixture of rotamers; *indicates resonances for resolved rotamers: δ 154.5, 144.8, 135.3, 132.6 (q, $^{2}J_{C\text{-}F}$ = 32.7 Hz), 130.8, 124.2 (q, $^{3}J_{C\text{-}F}$ = 3.9 Hz), 123.4 (q, $^{1}J_{C\text{-}F}$ = 272.9 Hz), 122.4 (q, $^{3}J_{C\text{-}F}$ = 3.7 Hz), 79.8, 52.2*, 51.7*, 45.7*, 43.9*, 43.1*, 33.3*, 32.5, 28.7 (3 C).

^{19}F NMR (377 MHz, $CDCl_3$): δ -62.8.

HRMS-ESI+ (m/z): $[M+Na]^+$ calculated for $C_{16}H_{19}NF_3O_2NaCl^+$, 372.0954; found, 372.0945.

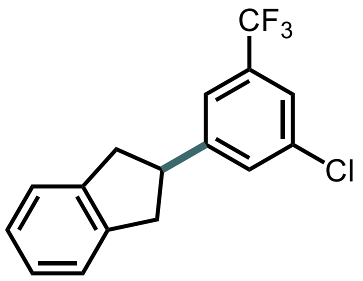

2-(3-CHLORO-5-(TRIFLUOROMETHYL)PHENYL)-2,3-DIHYDRO-1H-INDENE (30)

Prepared according to the general procedure for carboxylic acid scope in the decarboxylative arylation from 1-chloro-3-iodo-5-(trifluoromethyl)benzene (30.6 mg, 15.7 µL, 0.50 mmol, 1.00 equiv) and 2,3-dihydro-1H-indene-2-carboxylic acid (21.1 mg, 0.13 mmol, 1.30 equiv) with phthalimide (7.40 mg, 0.05 mmol, 0.50 equiv) in 2.00 mL of 1,4-dioxane. The title compound was isolated by combining three identical 0.10 mmol scale reactions and purified via column chromatography (1% THF in pentane) to produce a clear oil (60.5 mg, 68% yield).

^{1}H NMR (400 MHz, CDCl$_3$): δ 7.47 – 7.44 (m, 3H), 7.27 – 7.19 (m, 4H), 3.72 (p, J = 8.6 Hz, 1H), 3.40 (dd, J = 15.6, 8.2 Hz, 2H), 3.07 (dd, J = 15.5, 8.7 Hz, 2H).

^{13}C{^{1}H} (201 MHz, CDCl$_3$): δ 148.7, 142.1 (2 x Ar-C), 135.1, 132.4 (q, $^{2}J_{C-F}$ = 32.7 Hz), 130.8, 127.0 (2 x Ar-C), 124.6 (2 x Ar-C), 123.5, (q, $^{1}J_{C-F}$ = 272.5 Hz), 123.6(q, $^{3}J_{C-F}$ = 3.9 Hz), 122.4 (q, $^{3}J_{C-F}$ = 3.7 Hz), 45.0, 40.8 (2C).
^{19}F NMR (377 MHz, CDCl$_3$): δ -62.7.

HRMS-EI+ (m/z): $[M]^{\cdot+}$ calculated for $C_{16}H_{12}F_3Cl^+$, 296.0579; found, 296.0573.

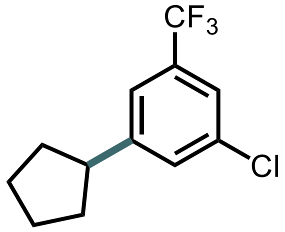

1-CHLORO-3-CYCLOPENTYL-5-(TRIFLUOROMETHYL)BENZENE (31)
Prepared according to the general procedure for unactivated carboxylic acid scope in the decarboxylative arylation from 1-chloro-3-iodo-5-(trifluoromethyl)benzene (30.6 mg, 15.7 µL, 0.10 mmol, 1.00 equiv) and cyclopentanecarboxylic acid (14.8 mg, 14.1 µL, 0.13 mmol, 1.30 equiv) with Zn (1.00 mg, 0.015 mmol, 0.15 equiv) in 2.00 mL of 1,4-dioxane. The title compound was isolated by combining three identical 0.10 mmol scale reactions and purified via column chromatography (eluting with 100% pentane) to produce a clear oil (39 mg, 53% yield).

^{1}H NMR (400 MHz, CDCl$_3$): δ 7.42 (s, 1H), 7.39 (s, 1H), 7.36 (s, 1H), 3.02 (tt, J = 9.8, 7.5 Hz, 1H), 2.17 – 2.03 (m, 2H), 1.89 – 1.77 (m, 2H), 1.77 – 1.65 (m, 2H), 1.63 – 1.51 (m, 2H).

^{13}C{^{1}H} (201 MHz, CDCl$_3$): δ 149.8, 134.7, 132.1 (q, $^{2}J_{C-F}$ = 32.6 Hz), 130.8, 123.6 (q, $^{1}J_{C-F}$ = 273 Hz), 123.0 (q, $^{3}J_{C-F}$ = 3.9 Hz), 122.4 (q, $^{3}J_{C-F}$ = 3.7 Hz), 45.7, 34.5 (2C), 25.5 (2C).

^{19}F NMR (377 MHz, CDCl$_3$): δ -62.7.

HRMS-EI+ (m/z): $[M-H]^{\cdot-}$ calculated for $C_{12}H_{11}F_3Cl^+$, 247.0501; found, 247.0492.

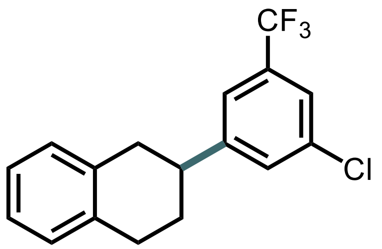

2-(3-CHLORO-5-(TRIFLUOROMETHYL)PHENYL)-1,2,3,4-TETRAHYDRONAPHTHALENE (32)
Prepared according to the general procedure for unactivated carboxylic acid scope in the decarboxylative arylation from 1-chloro-3-iodo-5-(trifluoromethyl)benzene (30.6 mg, 15.7 µL, 0.10 mmol, 1.00 equiv) and 1,2,3,4-tetrahydronaphthalene-2-carboxylic acid (22.9 mg, 0.13 mmol, 1.30 equiv) with phthalimide (7.40 mg, 0.05 mmol, 0.50 equiv) in 2.00 mL of 1,4-dioxane. The title compound was isolated by combining three identical 0.10 mmol scale reactions and purified via column chromatography (eluting with 1% THF in pentane) to produce a clear oil (47.5 mg, 51% yield).

^{1}H NMR (400 MHz, $CDCl_3$): δ 7.53 (d, *J* = 1.8 Hz, 1H), 7.49 (d, *J* = 1.9 Hz, 1H), 7.46 (s, 1H), 7.24 – 7.16 (m, 3H), 7.14 (dt, *J* = 5.5, 3.5 Hz, 1H), 3.13 – 2.89 (m, 5H), 2.19 (dtd, *J* = 13.0, 4.6, 2.2 Hz, 1H), 2.06 – 1.90 (m, 1H).

^{13}C{^{1}H} (201 MHz, $CDCl_3$): δ 149.8, 135.9, 135.7, 135.1, 132.4 (q, $^{2}J_{C\text{-}F}$ = 32.7 Hz), 130.7, 130.7, 129.1, 126.2, 126.0, 123.6 (q, $^{1}J_{C\text{-}F}$ = 272.9) 123.6 (q, $^{3}J_{C\text{-}F}$ = 3.8 Hz), 122.2 (q, $^{3}J_{C\text{-}F}$ = 3.5 Hz), 40.7, 37.5, 30.2, 29.6.

^{19}F NMR (377 MHz, $CDCl_3$): δ -62.7.

HRMS-EI+ (m/z): $[M]^{\cdot+}$ calculated for $C_{17}H_{14}F_3Cl^{+}$, 310.0736; found, 310.0730.

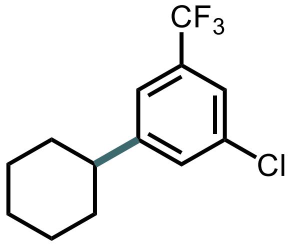

1-CHLORO-3-CYCLOHEXYL-5-(TRIFLUOROMETHYL)BENZENE (33)
Prepared according to the general procedure for unactivated carboxylic acid scope in the decarboxylative arylation from 1-chloro-3-iodo-5-(trifluoromethyl)benzene (30.6 mg, 15.7 µL, 0.10

mmol, 1.00 equiv) and cyclohexanecarboxylic acid (16.6 mg, 0.13 mmol, 1.30 equiv) with phthalimide (7.40 mg, 0.05 mmol, 0.50 equiv) in 2.00 mL of 1,4-dioxane. The title compound was purified via column chromatography (eluting with 1% THF in pentane) to produce a clear oil. The yield (54%) was determined by ^{1}H NMR spectroscopy using dibromomethane as an internal standard.

^{1}H NMR (400 MHz, $CDCl_3$): δ 7.42 (s, 1H), 7.37 (s, 1H), 7.34 (s, 1H), 2.55 (tt, *J* = 11.6, 2.9 Hz, 1H), 1.94 – 1.81 (m, 4H), 1.77 (ddd, *J* = 12.7, 3.2, 1.6 Hz, 1H), 1.40 (td, *J* = 9.3, 3.4 Hz, 4H), 1.26 (dtd, *J* = 12.7, 5.9, 3.2 Hz, 1H).

^{13}C{^{1}H} (201 MHz, $CDCl_3$): δ 151.1, 134.8, 132.1 (q, $^{2}J_{C\text{-}F}$ = 32.7 Hz), 130.6, 127.7, 125.0, 123.1 (q, $^{3}J_{C\text{-}F}$ = 3.7 Hz), 123.6 (q, $^{1}J_{C\text{-}F}$ = 274.7 Hz), 122.2 (q, $^{3}J_{C\text{-}F}$ = 3.7 Hz), 44.4, 34.2, 26.7, 26.0.

^{19}F NMR (377 MHz, $CDCl_3$): δ -62.7.

HRMS-EI+ (m/z): $[M]^{\cdot+}$ calculated for $C_{13}H_{14}F_3Cl^+$, 262.0736; found, 262.0731.

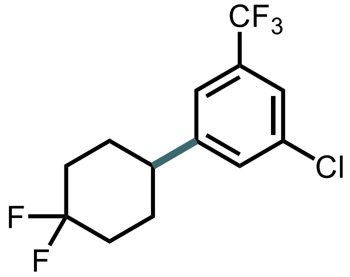

1-CHLORO-3-(4,4-DIFLUOROCYCLOHEXYL)-5-(TRIFLUOROMETHYL)BENZENE (34)

Prepared according to the general procedure for unactivated carboxylic acid scope in the decarboxylative arylation from 1-chloro-3-iodo-5-(trifluoromethyl)benzene (30.6 mg, 15.7 μL, 0.10 mmol, 1.00 equiv) and 4,4-difluorocyclohexane-1-carboxylic acid (21.3 mg, 0.13 mmol, 1.30 equiv) with Zn (1.00 mg, 0.015 mmol, 0.15 equiv) in 2.00 mL of 1,4-dioxane. The title compound was isolated by combining three identical 0.10 mmol scale reactions and purified via column chromatography (eluting with 100% pentane) to produce a clear oil (36.7 mg, 41% yield).

^{1}H NMR (400 MHz, $CDCl_3$): δ 7.47 (s, 1H), 7.39 (s, 1H), 7.36 (s, 1H), 2.67 (ddt, *J* = 11.4, 6.6, 3.5 Hz, 1H), 2.31 – 2.16 (m, 2H), 2.01 – 1.72 (m, 6H).

^{13}C{^{1}H} (201 MHz, $CDCl_3$): 148.3, 135.2, 132.5 (q, $^{2}J_{C\text{-}F}$ = 32.7 Hz), 130.5, 123.9 (q, $^{3}J_{C\text{-}F}$ = 3.7 Hz), 123.4 (q, $^{1}J_{C\text{-}F}$ = 274.0 Hz), 122.8 (dd, *J* = 244.4.240.4 Hz), 122.1 (q, $^{3}J_{C\text{-}F}$ = 3.7 Hz), 42.3, 34.0 (dd, $^{1}J_{C\text{-}F}$ = 23.2, 26.3 Hz), 30.1 (d, $^{2}J_{C\text{-}F}$ = 10.1 Hz, 2 x C).

^{19}F NMR (377 MHz, $CDCl_3$): -62.8, -92.1 (d, *J* = 236.9 Hz), -102.5 (m).

HRMS-EI+ (m/z): $[M]^{\cdot+}$ calculated for $C_{13}H_{12}F_5Cl^+$, 298.0547; found, 298.0555.

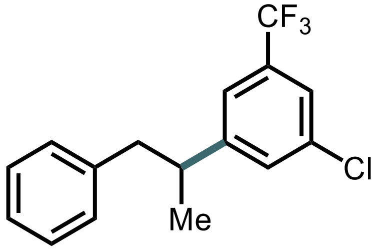

1-CHLORO-3-(1-PHENYLPROPAN-2-YL)-5-(TRIFLUOROMETHYL)BENZENE (35)

Prepared according to the general procedure for unactivated carboxylic acid scope in the decarboxylative arylation from 1-chloro-3-iodo-5-(trifluoromethyl)benzene (30.6 mg, 15.7 µL, 0.10 mmol, 1.00 equiv) and 2-methyl-3-phenylpropanoic acid (21.3 mg, 0.13 mmol, 1.30 equiv) with phthalimide (7.40 mg, 0.05 mmol, 0.50 equiv) in 2.00 mL of 1,4-dioxane. The title compound was isolated by combining three identical 0.10 mmol scale reactions and purified via column chromatography (eluting with 100% pentane) to produce a clear oil (47.5 mg, 53% yield).

^{1}H NMR (400 MHz, $CDCl_3$): δ 7.46 (s, 1H), 7.35 (s, 1H), 7.30 – 7.20 (m, 4H), 7.08 – 7.06 (d, J = 12.0 Hz, 2H), 3.06 (h, J = 7.1 Hz, 1H), 2.89 (dd, J = 13.4, 6.7 Hz, 1H), 2.80 (dd, J = 13.4, 8.1 Hz, 1H), 1.27 (d, J = 6.9 Hz, 3H).

$^{13}C\{^1H\}$ (201 MHz, $CDCl_3$): δ 150.0, 139.7, 134.8, 132.2 (q, $^2J_{C\text{-}F}$ = 32.7 Hz), 130.8, 129.2 (2 x Ar-C), 128.5 (2 x Ar-C), 126.4, 123.6 (q, $^1J_{C\text{-}F}$ = 273.7 Hz), 123.4 (q, $^3J_{C\text{-}F}$ = 4.0 Hz), 122.5 (q, $^3J_{C\text{-}F}$ = 4.0 Hz), 44.8, 41.9, 20.9.

^{19}F NMR (377 MHz, $CDCl_3$): δ -62.7.

HRMS-EI+ (m/z): $[M]^{\cdot+}$ calculated for $C_{16}H_{14}F_3Cl^+$, 298.0736; found, 298.0747.

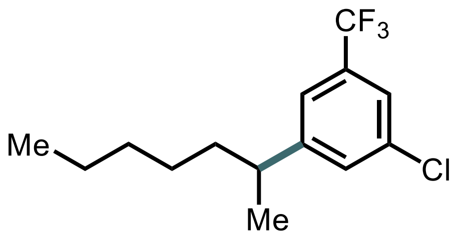

1-CHLORO-3-(HEPTAN-2-YL)-5-(TRIFLUOROMETHYL)BENZENE (36)
Prepared according to the general procedure for unactivated carboxylic acid scope in the decarboxylative arylation from 1-chloro-3-iodo-5-(trifluoromethyl)benzene (30.6 mg, 15.7 µL, 0.10 mmol, 1.00 equiv) and 2-methylheptanoic acid (14.4 mg, 0.13 mmol, 1.30 equiv) with phthalimide (7.40 mg, 0.05 mmol, 0.50 equiv) in 2.00 mL of 1,4-dioxane. The title compound was purified via column chromatography (eluting with 1% THF in pentane) to produce a clear oil. The yield (66%) was determined by ^{1}H NMR spectroscopy using dibromomethane as an internal standard.

<u>^{1}H NMR (400 MHz, $CDCl_3$):</u> 7.43 (s, 1H), 7.34 (s, 1H), 7.31 (s, 1H), 2.73 (h, J = 7.1 Hz, 1H), 1.56 (q, J = 7.4 Hz, 2H), 1.31 – 1.16 (m, 9H), 0.88 – 0.82 (m, 3H).

<u>^{13}C{^{1}H} (201 MHz, $CDCl_3$):</u> δ 151.2, 134.8, 132.2 (q, $^{2}J_{C-F}$= 32.7 Hz), 130.7, 123.6 (q, $^{1}J_{C-F}$ = 274.7 Hz), 123.2 (q, $^{3}J_{C-F}$ = 3.7 Hz), 122.3 (q, $^{3}J_{C-F}$ = 3.7 Hz), 40.0, 38.2, 31.9, 27.3, 22.7, 22.1, 14.2.

<u>^{19}F NMR (377 MHz, $CDCl_3$):</u> δ -62.7.

<u>HRMS-EI+ (m/z):</u> $[M]^{\cdot+}$ calculated for $C_{14}H_{18}F_3Cl^+$, 278.1049; found, 278.1046.

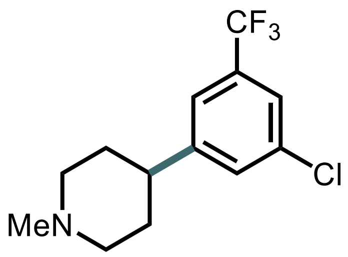

4-(3-CHLORO-5-(TRIFLUOROMETHYL)PHENYL)-1-METHYLPIPERIDINE (37)
Prepared according to the general procedure for unactivated carboxylic acid scope in the decarboxylative arylation from 1-chloro-3-iodo-5-(trifluoromethyl)benzene (30.6 mg, 15.7 µL, 0.10 mmol, 1.00 equiv) and 1-methylpiperidine-4-carboxylic acid (18.6 mg, 0.13 mmol, 1.30 equiv) with $ZnCl_2$ (4.10 mg, 0.0300 mmol, 0.300 equiv) in 2.00 mL of 1,4-dioxane. The resultant crude mixture was diluted with EtOAc (30mL) and brine (30 mL x 1). The organic phase was then washed with 1M NaOH solution (30 mL x 2). The base washed organic phase was then treated with 0.1M HCl solution (30 mL). The acidic aqueous phase was then washed with EtOAc (30 mL x 3). After removal of the organic phase, an additional 30 mL of EtOAc was added to the acidic aqueous phase. The organic phase

was discarded and the aqueous phase was then basified by 1M NaOH solution until the aqueous phase reached a pH of 11 as indicated by pH paper. The basified aqueous phase was then washed with EtOAc (30 mL x 3). The organic layer was collected. Following solvent removal *in vacuo*, mild heating was applied to the crude mixture for 10 minutes to produce a white solid. The yield (40%) was determined by ^{19}F NMR spectroscopy using fluorobenzene as an internal standard.

<u>^{1}H NMR (400 MHz, $CDCl_3$):</u> δ 7.43 (s, 1H), 7.38 (s, 1H), 7.36 (s, 1H), 2.98 (d, *J* = 11.6 Hz, 2H), 2.52 (tt, *J* = 12.1, 3.9 Hz, 1H), 2.32 (s, 3H), 2.04 (td, *J* = 11.8, 2.7 Hz, 2H), 1.84 (d, *J* = 13.0 Hz, 2H), 1.77 (qd, *J* = 12.0, 3.8 Hz, 2H).

<u>^{13}C{^{1}H} (201 MHz, $CDCl_3$):</u> δ 149.5, 135.0, 132.3 (d, $^{2}J_{C\text{-}F}$ = 32.6 Hz), 130.6, 123.5 (q, $^{1}J_{C\text{-}F}$ = 272.6), 123.5 (q, $^{3}J_{C\text{-}F}$ = 3.7 Hz), 122.2 (q, $^{3}J_{C\text{-}F}$ = 3.7 Hz), 56.1 (2C), 46.5, 41.9, 33.3 (2C).

<u>^{19}F NMR (377 MHz, $CDCl_3$):</u> δ -62.8.

<u>HRMS-ES+ (m/z):</u> $[M+H]^+$ calculated for $C_{13}H_{16}NClF_3^+$, 278.0923; found, 278.0927

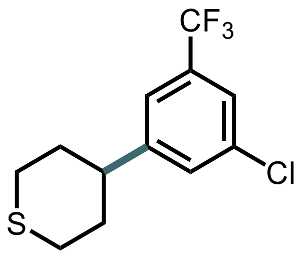

4-(3-CHLORO-5-(TRIFLUOROMETHYL)PHENYL)TETRAHYDRO-2H-THIOPYRAN (38)

Prepared according to the general procedure for unactivated carboxylic acid scope in the decarboxylative arylation from 1-chloro-3-iodo-5-(trifluoromethyl)benzene (30.6 mg, 15.7 µL, 0.10 mmol, 1.00 equiv) and tetrahydro-2H-thiopyran-4-carboxylic acid (19.0 mg, 0.13 mmol, 1.30 equiv) with 4-ethylpyridine (1.60 mg, 1.4 µL, 0.015 mmol, 0.15 equiv) in 2.00 mL of 1,4-dioxane. The title compound was purified via column chromatography (eluting with 100% pentane) to produce a clear oil. The yield (34%) was determined by ^{1}H NMR spectroscopy using dibromomethane as an internal standard.

<u>^{1}H NMR (400 MHz, $CDCl_3$):</u> δ 7.46 (s, 1H), 7.37 (s, 1H), 7.33 (s, 1H), 7.33 (s, 1H), 2.83 (td, *J* = 13.1, 2.5 Hz, 2H), 2.75-2.68 (d, *J = 14.1 Hz,* 2H), 2.59 (tt, *J* = 12.1, 3.1, 2H), 2.15 (d, *J* = 13.2, 2H), 1.85 (qd, *J* = 12.8, 3.4, 2H).

<u>^{13}C{^{1}H} (201 MHz, $CDCl_3$):</u> δ 149.8, 135.1, 132.5 (q, $^{2}J_{C\text{-}F}$ = 32.9 Hz), 130.6, 123.7(q, $^{3}J_{C\text{-}F}$ = 3.7 Hz), 123.5 (q, $^{1}J_{C\text{-}F}$ =273.1) 122.2 (q, $^{3}J_{C\text{-}F}$ = 3.7 Hz), 44.2, 34.9 (2C), 29.1 (2C).

<u>^{19}F NMR (377 MHz, $CDCl_3$):</u> δ -62.7.

HRMS-EI+ (m/z): $[M]^{\cdot+}$ calculated for $C_{12}H_{12}F_3ClS^+$, 280.0300; found, 280.0298

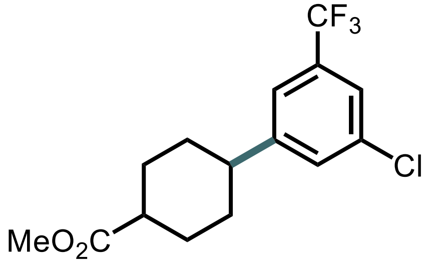

METHYL 4-(3-CHLORO-5-(TRIFLUOROMETHYL)PHENYL)CYCLOHEXANE-1-CARBOXYLATE (39)
Prepared according to the general procedure for unactivated carboxylic acid scope in the decarboxylative arylation from 1-chloro-3-iodo-5-(trifluoromethyl)benzene (30.6 mg, 15.7 µL, 0.10 mmol, 1.00 equiv) and 4-(methoxycarbonyl)cyclohexane-1-carboxylic acid (24.2 mg, 0.13 mmol, 1.30 equiv) with 4-ethylpyridine (1.60 mg, 1.4 µL, 0.015 mmol, 0.15 equiv) in 2.00 mL of 1,4-dioxane. The title compound was purified via gradient column chromatography (eluting with 0-10% ether in pentane) to produce a white solid (major diastereomer) and a clear oil (minor diastereomer). The yield (24%, dr 3:1) was determined by ^1H NMR spectroscopy using dibromomethane as an internal standard.

Major Diastereomer:
^1H NMR (600 MHz, $CDCl_3$): δ 7.47 (s, 1H), 7.39 (s, 1H), 7.36 (s, 1H), 3.72 (s, 3H) 2.60 (t, *J* = 12.2 Hz, 1H), 2.39 (t, *J* = 12.2 Hz, 1H), 2.16 (d, *J* = 10.4 Hz, 2H), 2.02 (d, *J* = 10.3 Hz, 2H), 1.63 (q, *J* = 13.0 Hz, 2H), 1.50 (q, *J* = 13.0 Hz, 2H).

$^{13}C\{^1H\}$ (201 MHz, $CDCl_3$): δ 176.1, 149.9, 135.0, 132.3 (q, $^2J_{C-F}$ = 32.6 Hz), 130.6, 123.5 (q, $^1J_{C-F}$ = 272.8 Hz), 123.5 (q, $^3J_{C-F}$ = 3.9 Hz), 122.1 (q, $^3J_{C-F}$ = 3.9 Hz), 51.8, 43.4, 42.8, 33.1 (2C), 29.1 (2C).

^{19}F NMR (377 MHz, $CDCl_3$): δ -62.7.

Minor Diastereomer:
^1H NMR (600 MHz, $CDCl_3$): δ 7.42 (s, 1H), 7.41 (s, 1H), 7.34 (s, 1H), δ 3.73 (s, 3H), δ 2.74 – 2.72 (m, 1H), δ 2.57 (m, 1H), δ 2.28 – 2.24 (m, 2H), δ 1.80 (m, 2H), δ 1.79 – 1.61 (overlapping proton, m, 4H).

$^{13}C\{^1H\}$ (201 MHz, $CDCl_3$): δ 175.5, 150.1, 134.9, 132.2 (q, $^2J_{C-F}$ = 32.3 Hz), 130.6, 123.5 (q, $^1J_{C-F}$ = 272.6 Hz), 123.4 (q, $^3J_{C-F}$ = 3.9 Hz), 122.2 (q, $^3J_{C-F}$ = 3.9 Hz), 51.9, 43.5, 38.6, 30.3 (2C), 27.4 (2C).

^{19}F NMR (377 MHz, $CDCl_3$): δ -62.7.

HRMS-EI+ (m/z): $[M]^{\cdot+}$ calculated for $C_{15}H_{16}F_3ClO_2^+$, 320.0790; found, 320.0783

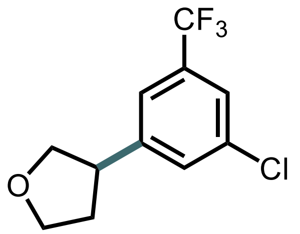

3-(3-CHLORO-5-(TRIFLUOROMETHYL)PHENYL)TETRAHYDROFURAN (40)

Prepared according to the general procedure for unactivated carboxylic acid scope in the decarboxylative arylation from 1-chloro-3-iodo-5-(trifluoromethyl)benzene (30.6 mg, 15.7 μL, 0.10 mmol, 1.00 equiv) and tetrahydrofuran-3-carboxylic acid (15.1 mg, 12.4 μL, 0.13 mmol, 1.30 equiv) in 2.00 mL of 1,4-dioxane. The title compound was purified via gradient column chromatography (eluting with 0-5% ether in pentane) to produce a clear oil. The yield (46%) was determined by ^{1}H NMR spectroscopy using dibromomethane as an internal standard.

^{1}H NMR (400 MHz, $CDCl_3$): δ 7.46 (s, 1H), 7.43 (s, 1H), 7.38 (s, 1H), 4.12 – 4.06 (m, 2H), 3.91 (q, J = 7.9 Hz, 1H), 3.75 (dd, J = 8.8, 6.4 Hz, 1H), 3.43 (p, J = 7.3 Hz, 1H), 2.47 – 2.36 (m, 1H), 1.97 (dq, J = 12.5, 7.7 Hz, 1H).

$^{13}C\{^1H\}$ (101 MHz, $CDCl_3$): δ 146.6, 135.2, 132.5 (q, $^2J_{C-F}$ = 32.9 Hz), 130.9, 123.9 (q, $^3J_{C-F}$ = 3.9 Hz), 123.4 (q, $^1J_{C-F}$ = 272.9 Hz), 122.5 (q, $^3J_{C-F}$ = 3.7 Hz), 74.4, 68.4, 44.7, 34.7.

^{19}F NMR (377 MHz, $CDCl_3$): -62.8.

HRMS-EI+ (m/z): $[M]^{\cdot+}$ calculated for $C_{11}H_{10}F_3ClO^+$, 350.0372; found, 350.0369

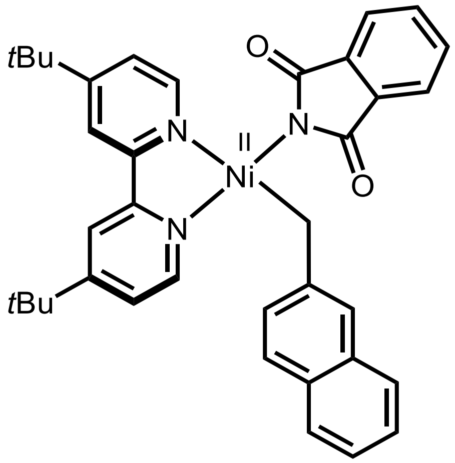

($^{4\text{-}TBU}$BPY)NI(2-NAPHTHYLENYLMETHYL)(PHTHALIMIDO) (46)

The intermediate oxidative addition complex was prepared following a modified literature procedure.[2] In a N_2-filled glovebox, an oven-dried 20 mL vial equipped with a PTFE-coated magnetic stir bar was charged Ni(cod)$_2$ (275.1 mg, 1.0 mmol, 1.0 equiv), dtbbpy (268.4 mg, 1.0 mmol, 1.0 equiv), and THF (5 mL). The vial was sealed with a PTFE-coated screw cap, and the mixture was stirred for at least 1 h at rt. The vial was moved to the glovebox freezer to cool to -35 °C for 10 min. The vial was removed from the freezer, and a solution of 2-(chloromethyl)naphthalene (1.0 mmol, 1.0 equiv) in THF (1.0 mL) was added dropwise to the mixture while stirring and warming to rt, resulting in the formation of a dark purple precipitate. The mixture was allowed to stir for 30 min before potassium phthalimide (185.2 mg, 1.0 mmol, 1.0 equiv) was added in one portion. The mixture was allowed to stir at rt for 12 h, resulting in the disappearance of the purple precipitate and formation of a dark red solution. The mixture was filtered through celite, and the product purified by crystallization from vapor diffusion of pentane into the filtrate to give dark purple needles (349.8 mg, 57% yield).

<u>^{1}H NMR (500 MHz, CD_3CN):</u> δ 8.17 (m, 2H), 8.12 (m, 2H), 7.48 (m, 2H), 7.42 (m, 2H), 7.35 – 7.22 (m, 6H), 7.22 – 7.13 (m, 3H), 2.55 (s, 2H), 1.38 (s, 18H).

<u>^{13}C{^{1}H} (126 MHz, CD_3CN):</u> δ 164.8, 150.8, 149.7, 135.2, 131.7, 130.9, 129.4, 128.3, 127.3, 127.0, 126.3, 124.15, 124.45, 123.8, 120.4, 120.0, 36.3, 30.3, 17.5.

XI. NMR Spectra

^{1}H NMR Spectrum of 4-(*tert*-butyl)-cyanopicolinimidamide (500 MHz, $CDCl_3$):

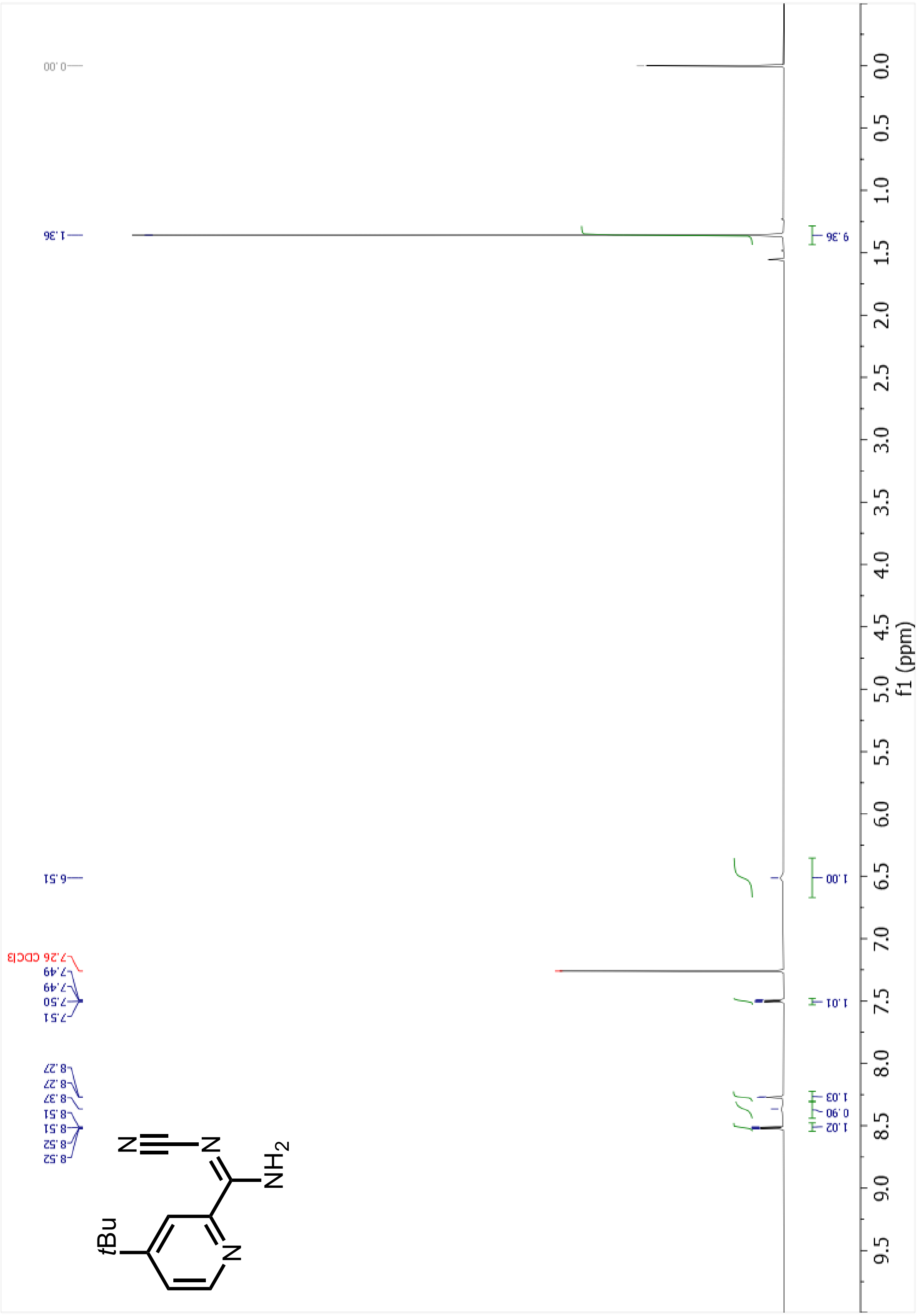

$^{13}C\{^{1}H\}$ NMR SPECTRUM OF 4-(*TERT*-BUTYL)-N-CYANOPICOLINIMIDAMIDE (101 MHz, $CDCl_3$):

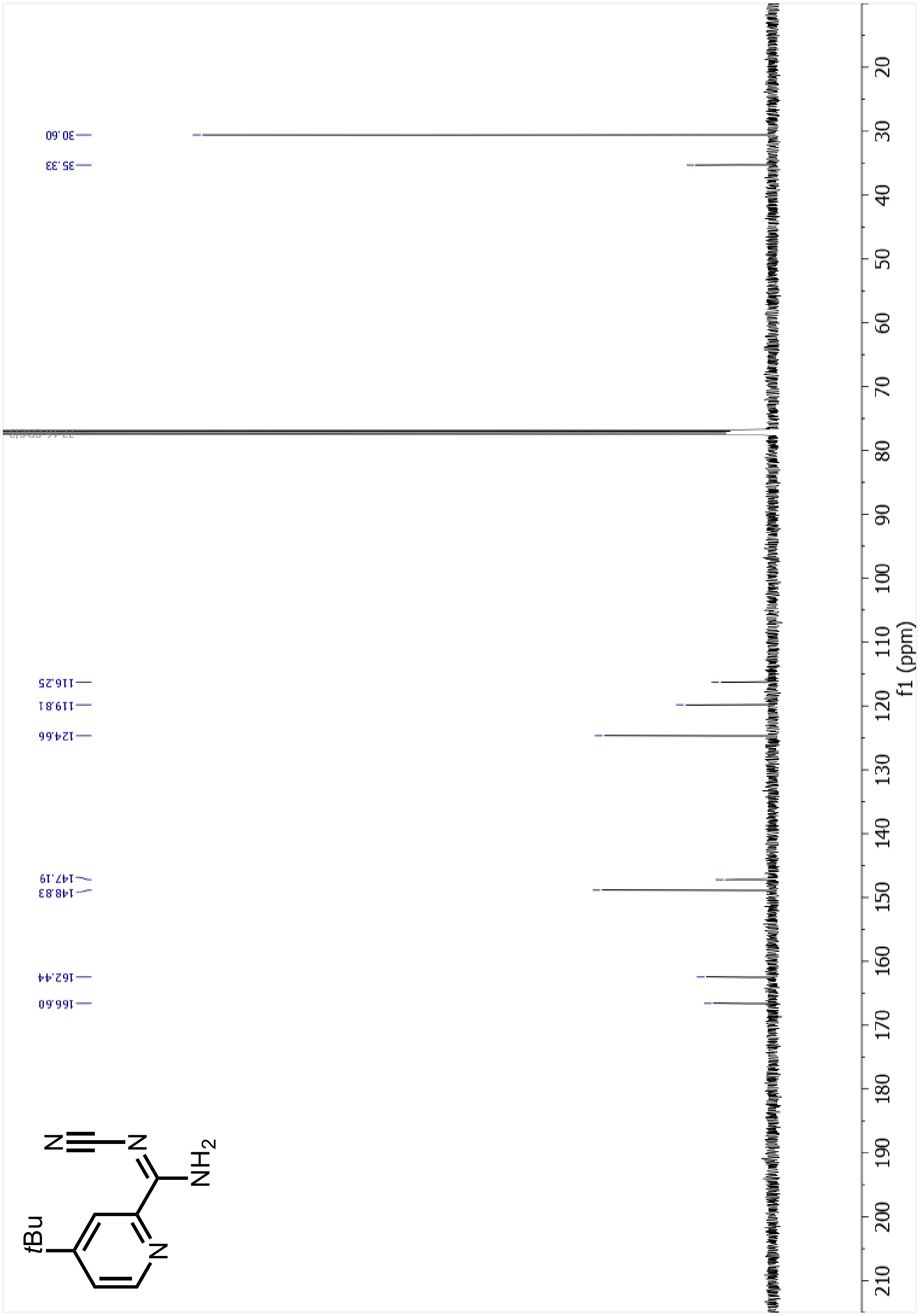

^{1}H NMR SPECTRUM OF 1,3-DIOXOISOINDOLIN-2-YL 2,3-DIHYDRO-1H-INDENE-2-CARBOXYLATE (400 MHz, $CDCl_3$):

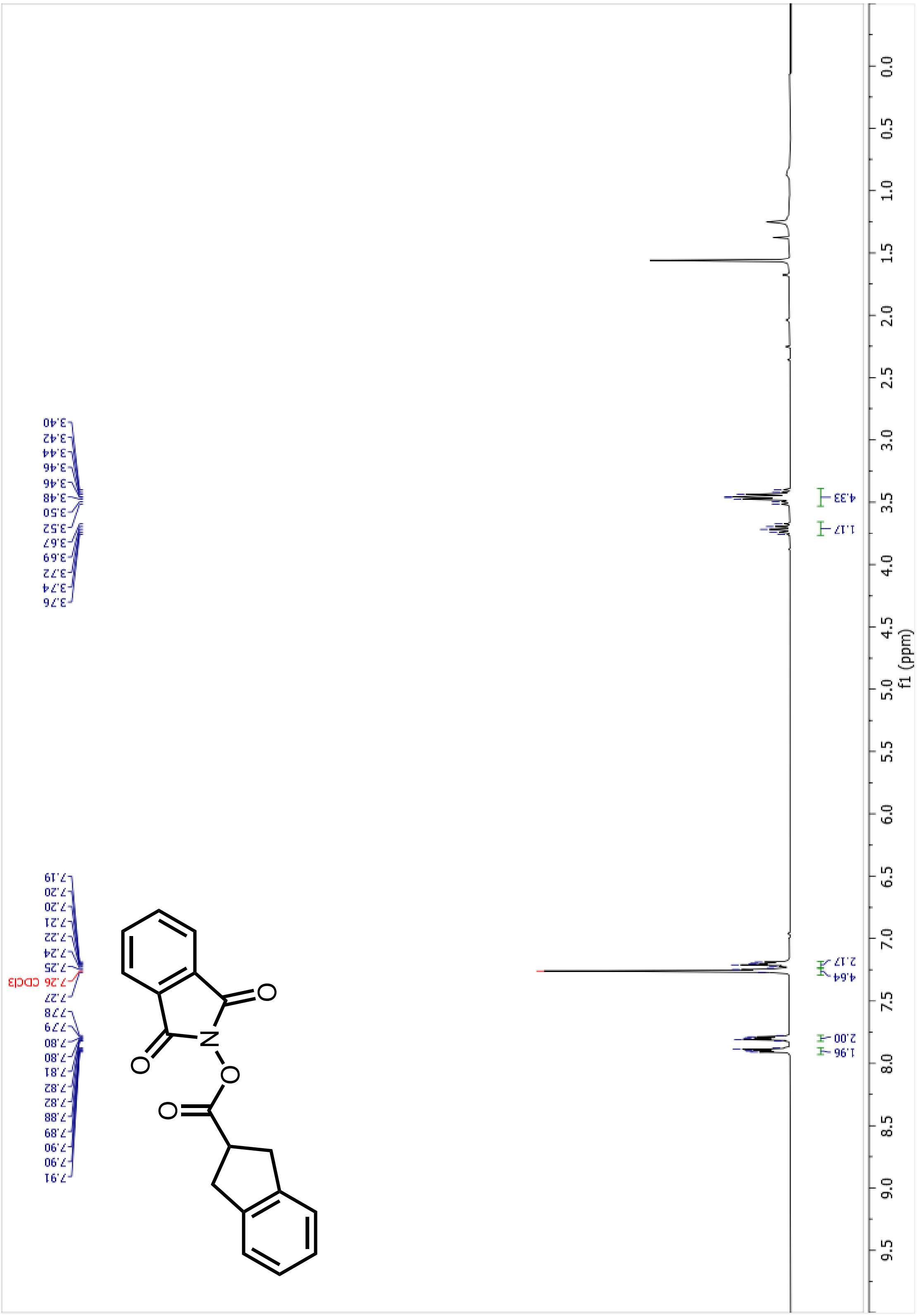

$^{13}C\{^{1}H\}$ NMR SPECTRUM OF 1,3-DIOXOISOINDOLIN-2-YL 2,3-DIHYDRO-1H-INDENE-2-CARBOXYLATE (101 MHz, $CDCl_3$):

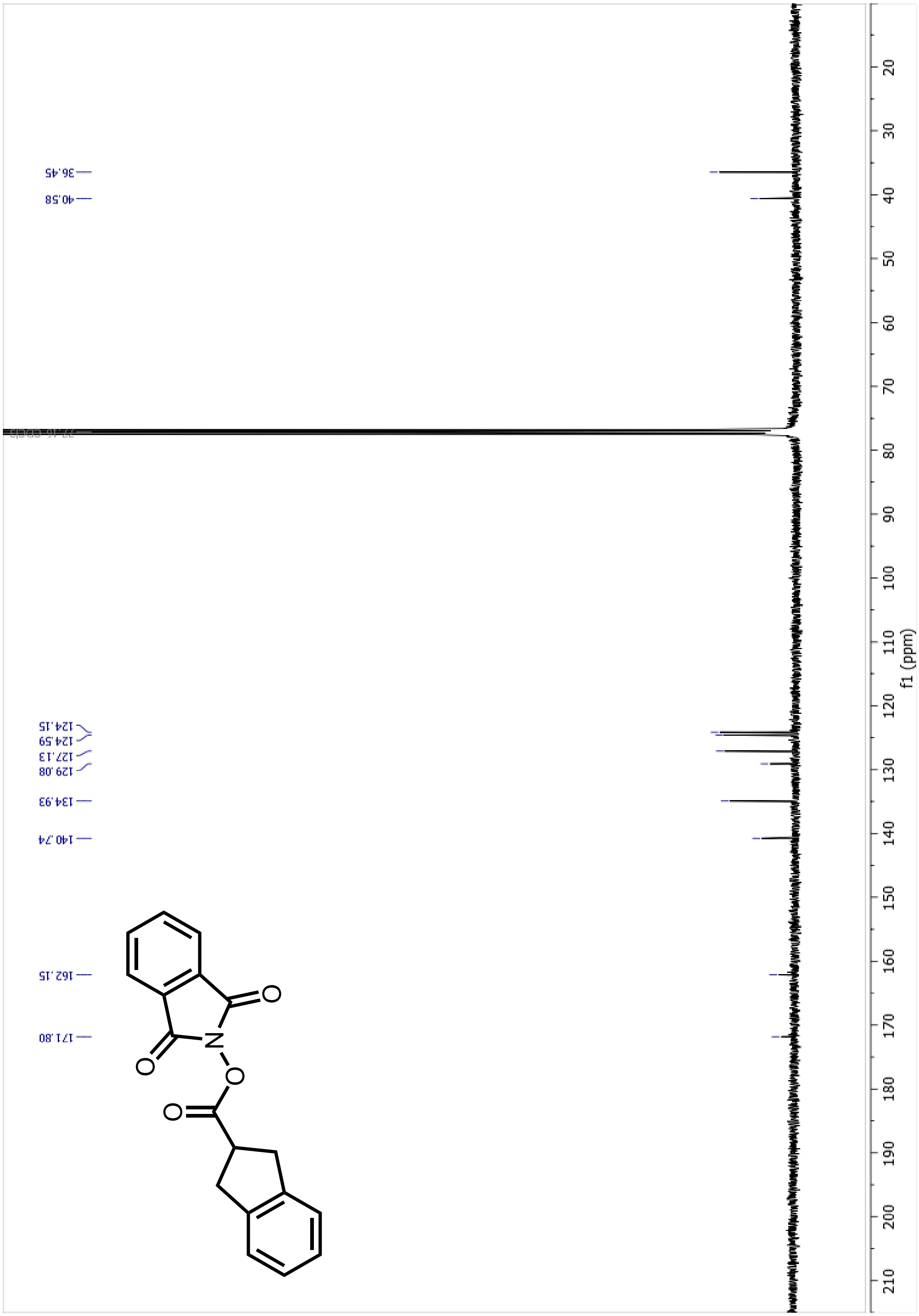

^{1}H NMR SPECTRUM OF **1A** (400 MHz, $CDCl_3$):

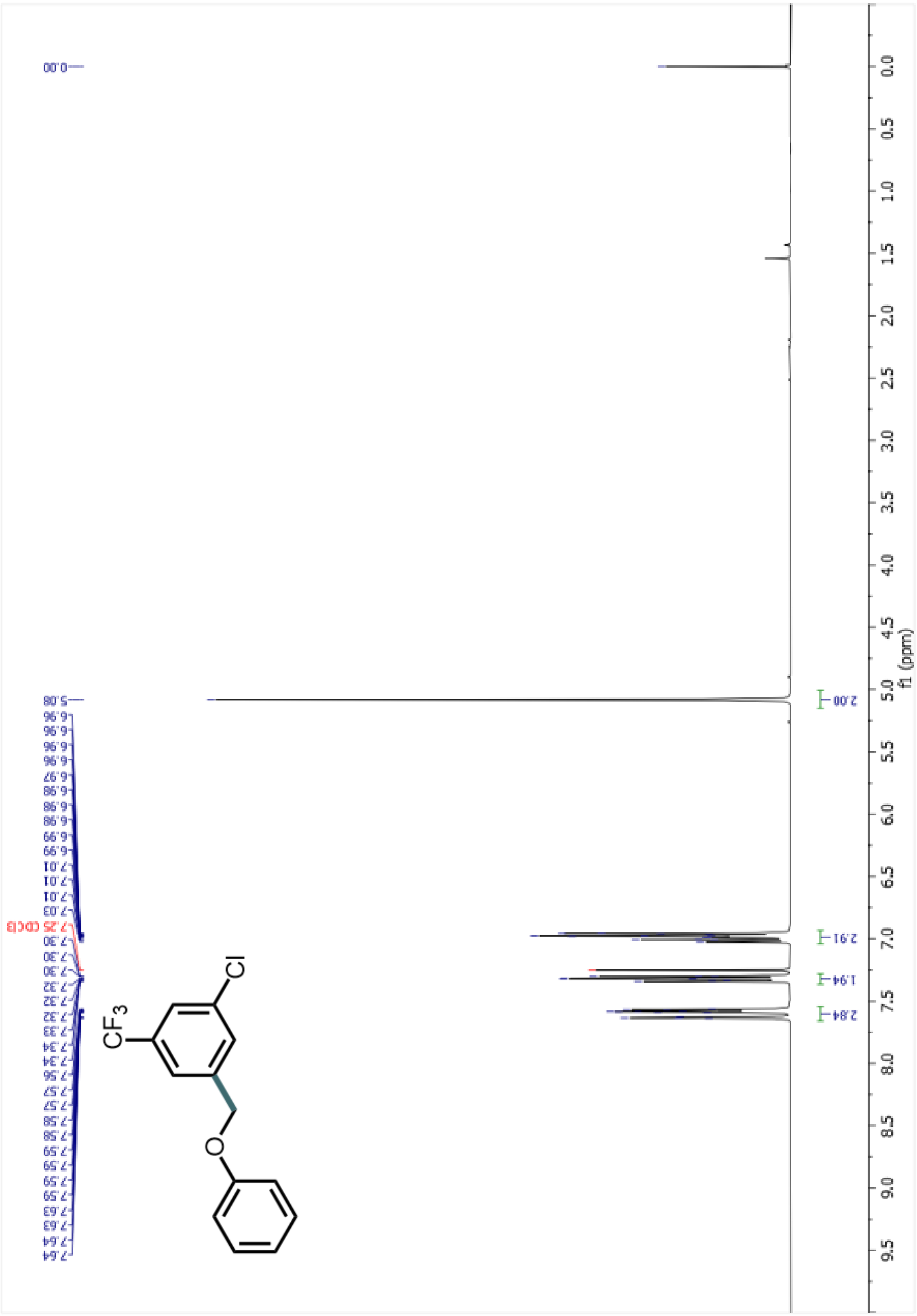

$^{13}C\{^{1}H\}$ NMR SPECTRUM OF 1A (101 MHz, $CDCl_3$):

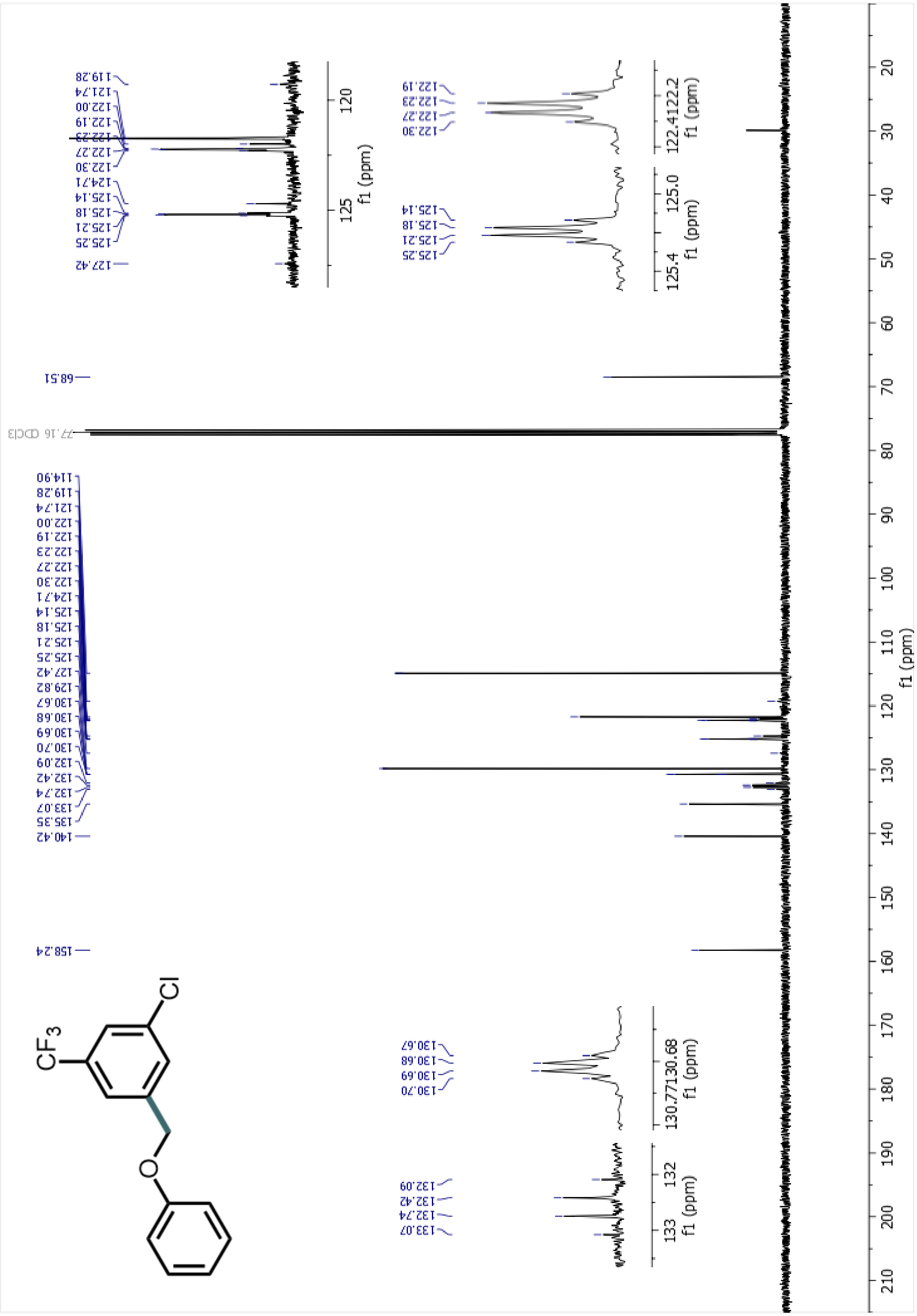

^{19}F NMR SPECTRUM OF 1**A** (377 MHz, $CDCl_3$):

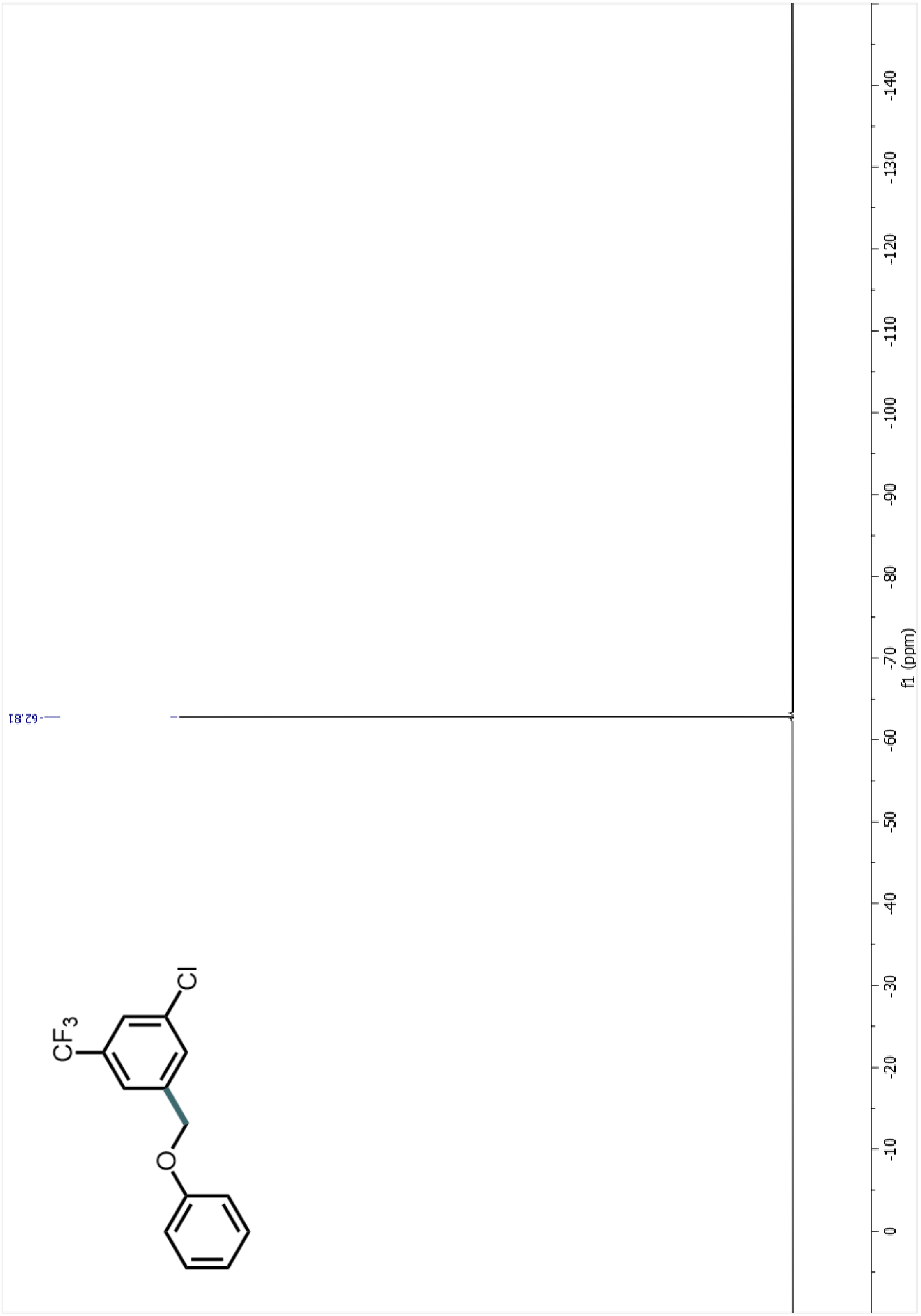

^{1}H NMR SPECTRUM OF 1**B** (101 MHz, $CDCl_3$):

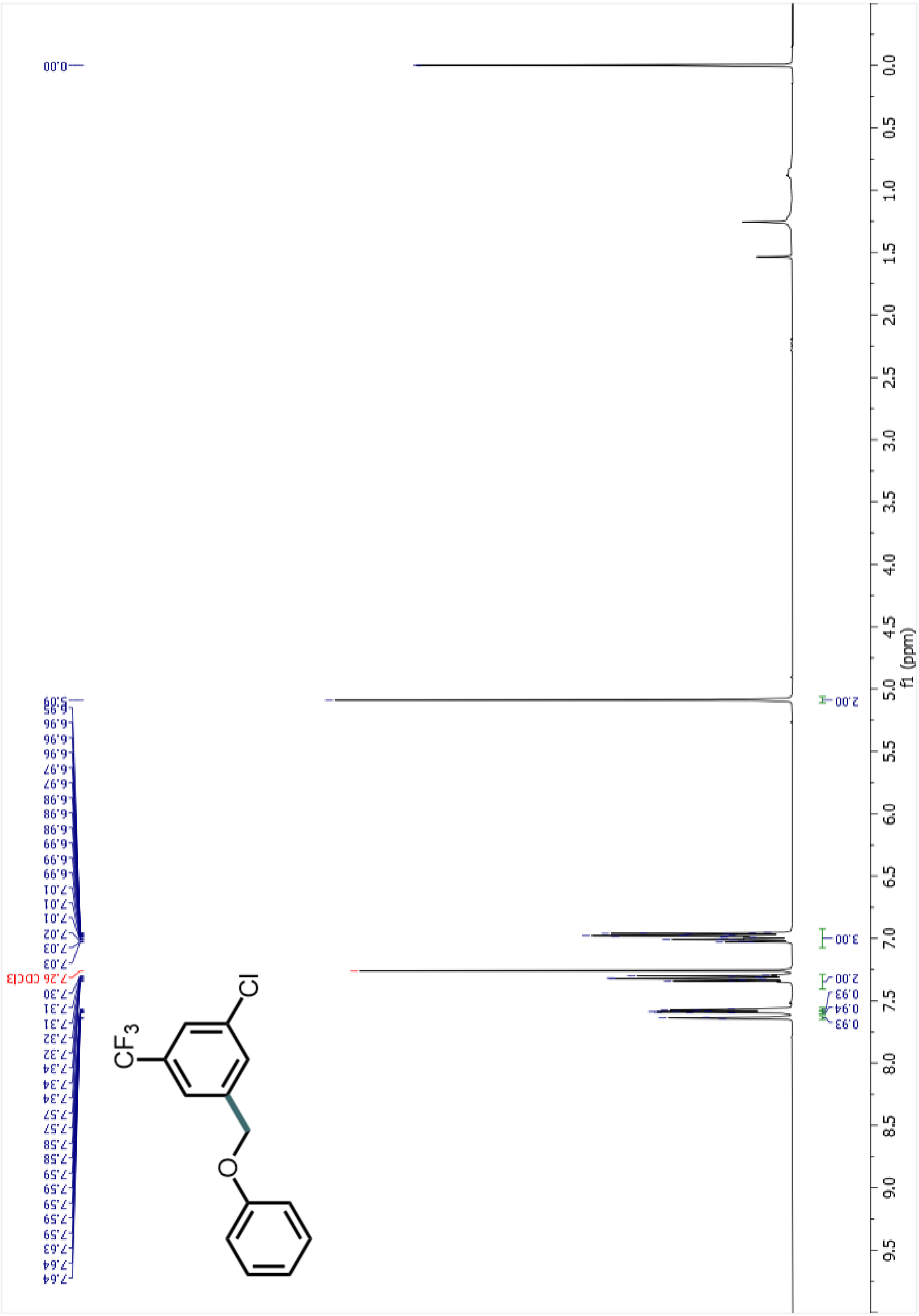

$^{13}C\{^{1}H\}$ NMR SPECTRUM OF 1**B** (101 MHz, $CDCl_3$):

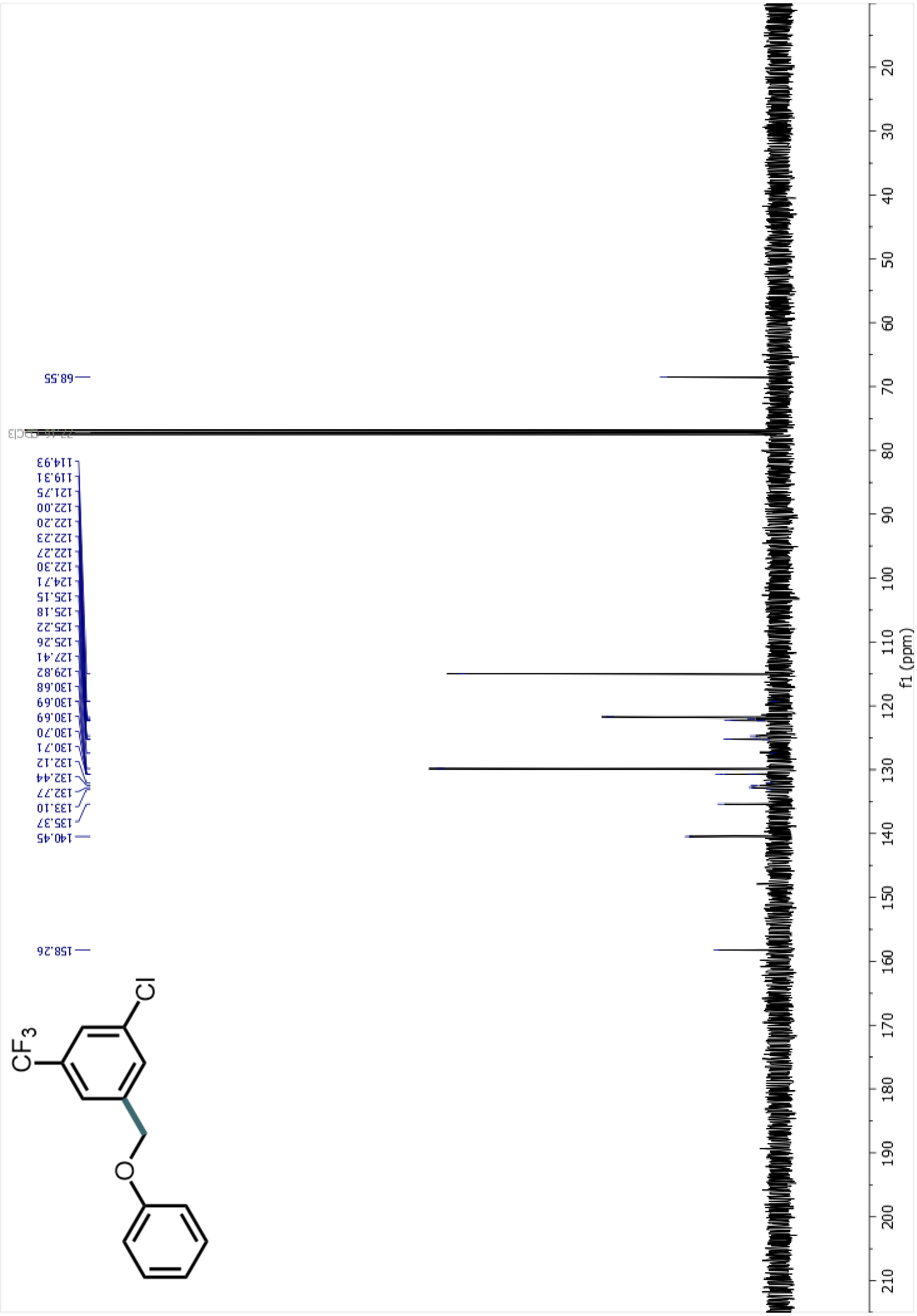

^{19}F NMR SPECTRUM OF 1**B** (377 MHz, $CDCl_3$):

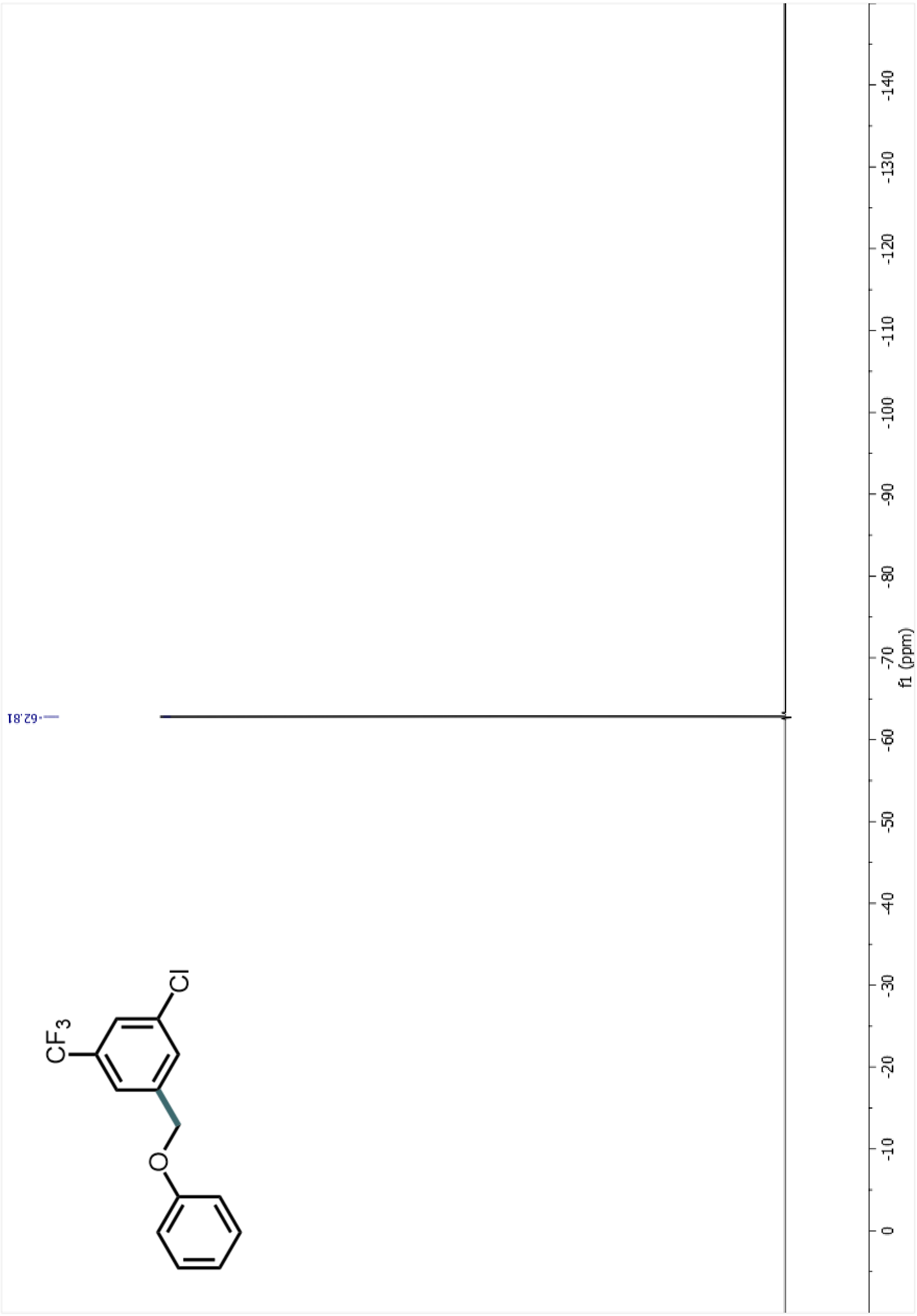

^{1}H NMR SPECTRUM OF **2** (400 MHz, $CDCl_3$):

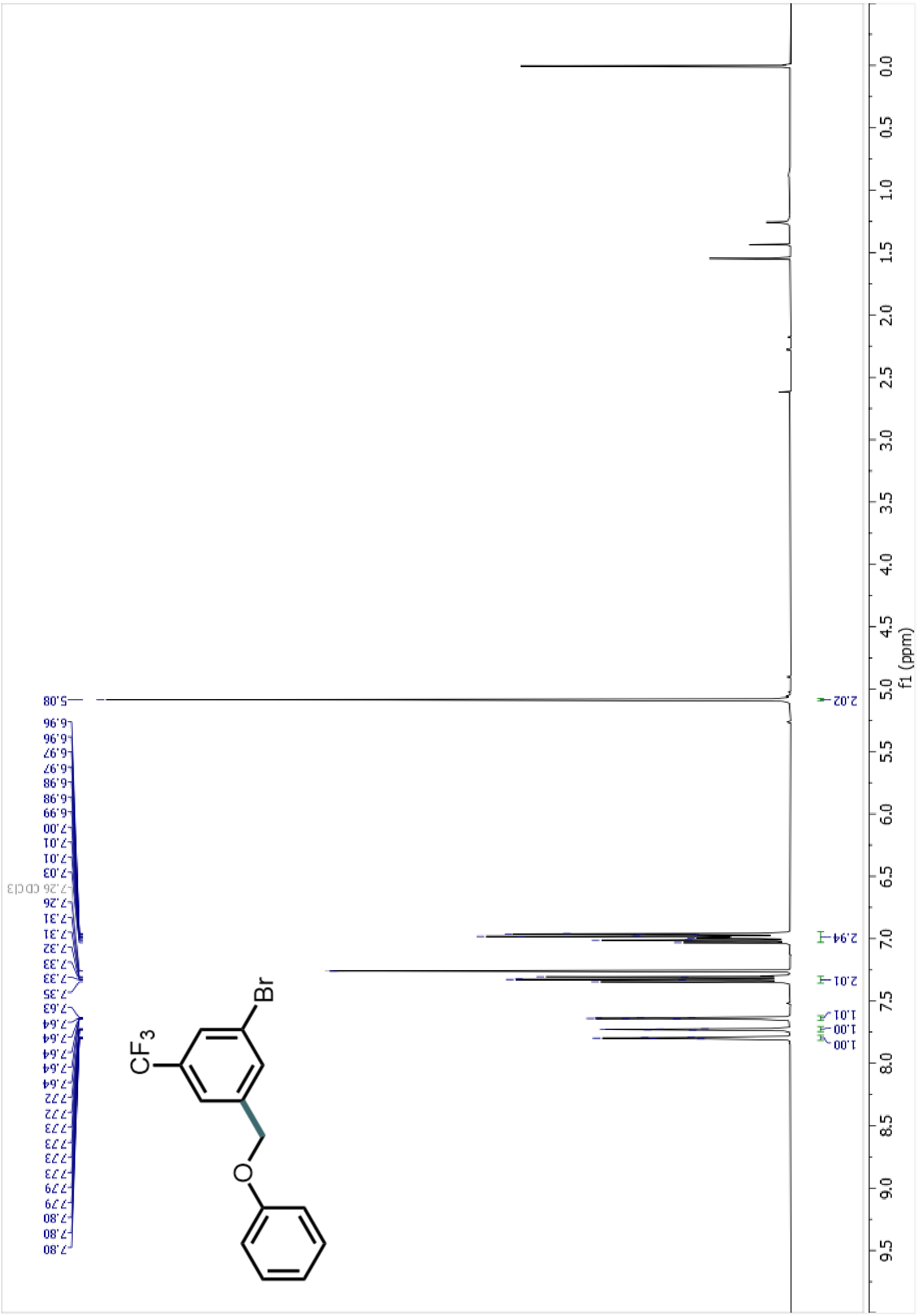

$^{13}C\{^1H\}$ NMR SPECTRUM OF **2** (101 MHz, $CDCl_3$):

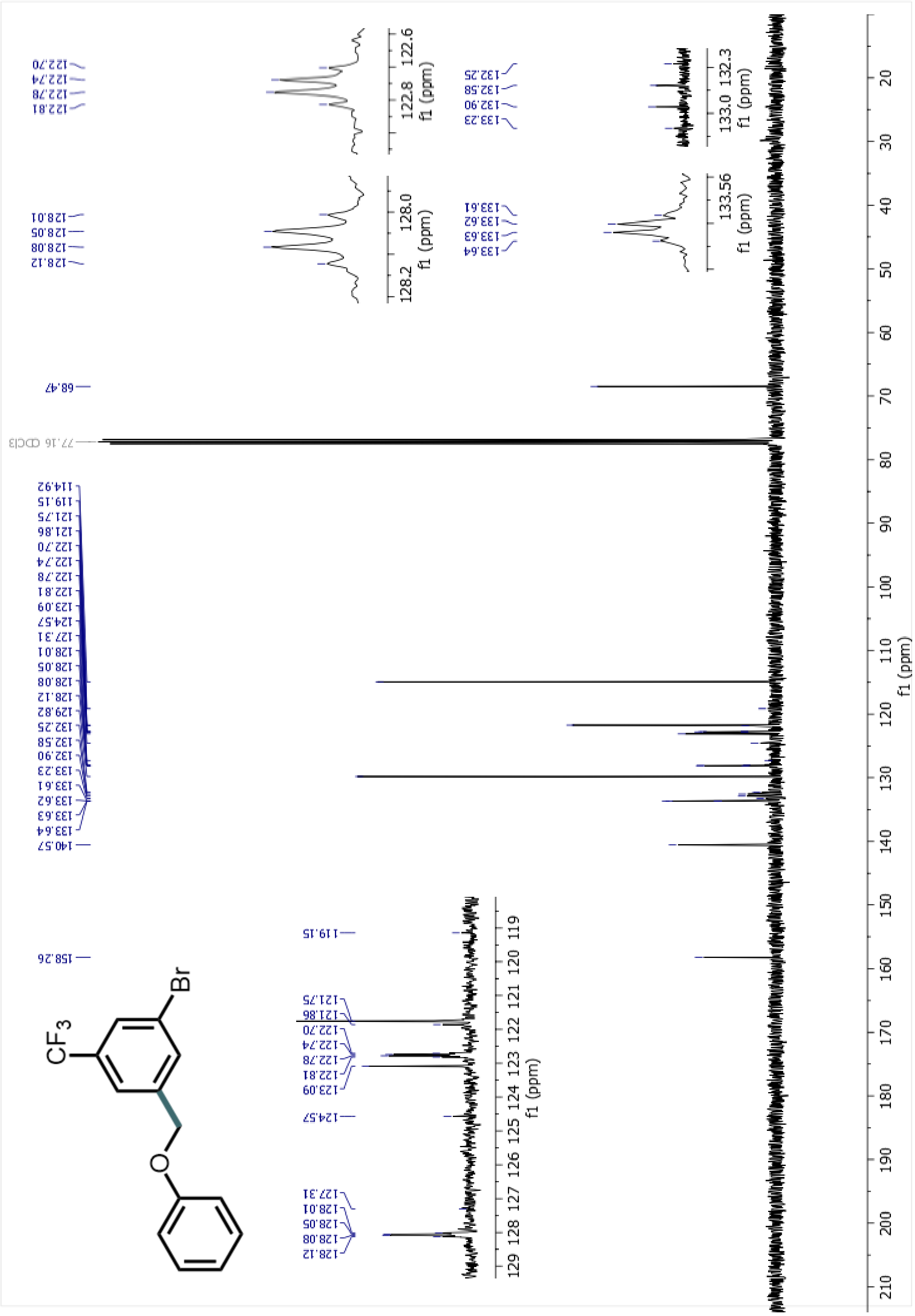

^{19}F NMR SPECTRUM OF **2** (377 MHz, $CDCl_3$):

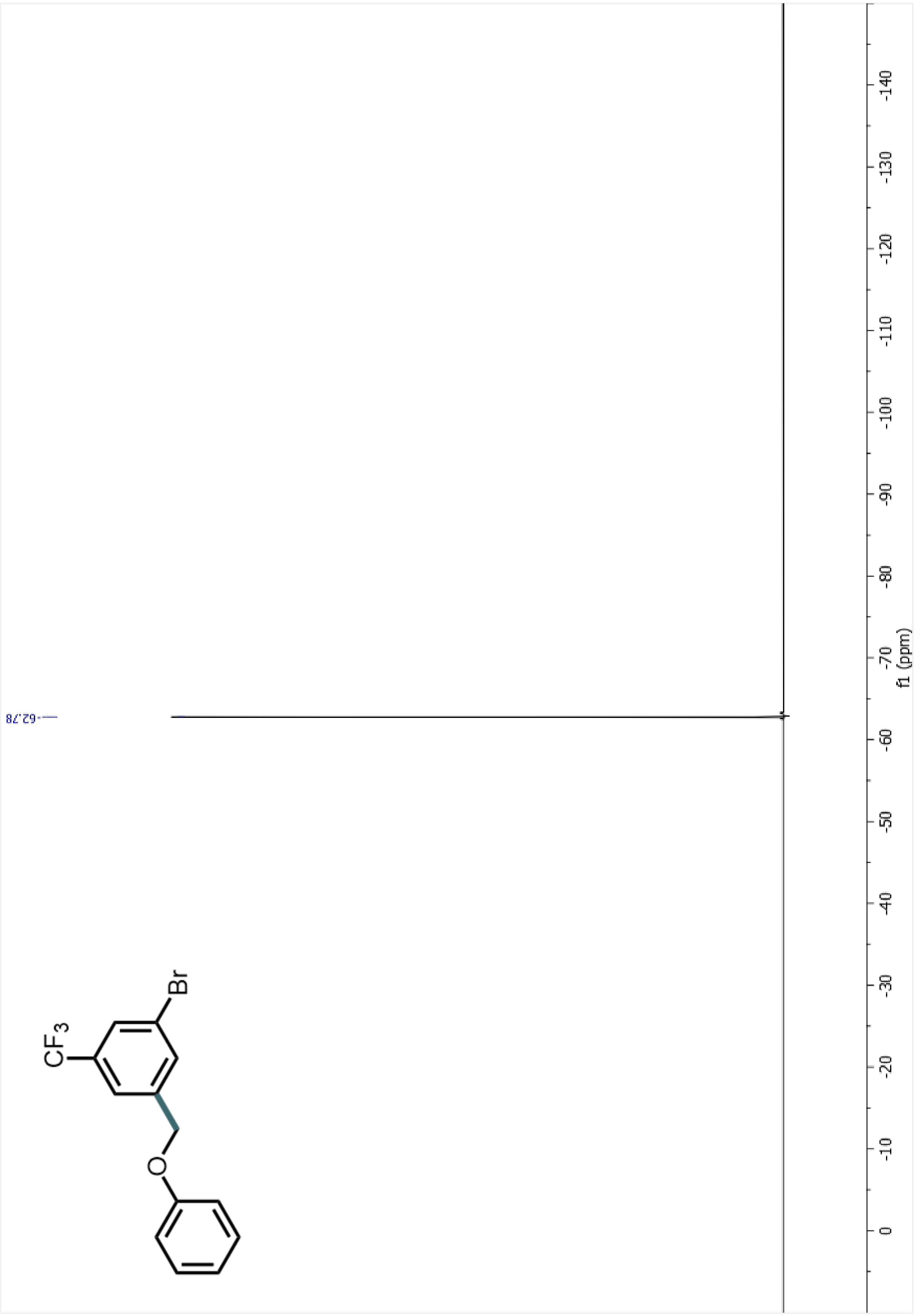

^{1}H NMR SPECTRUM OF **3** (400 MHz, $CDCl_3$):

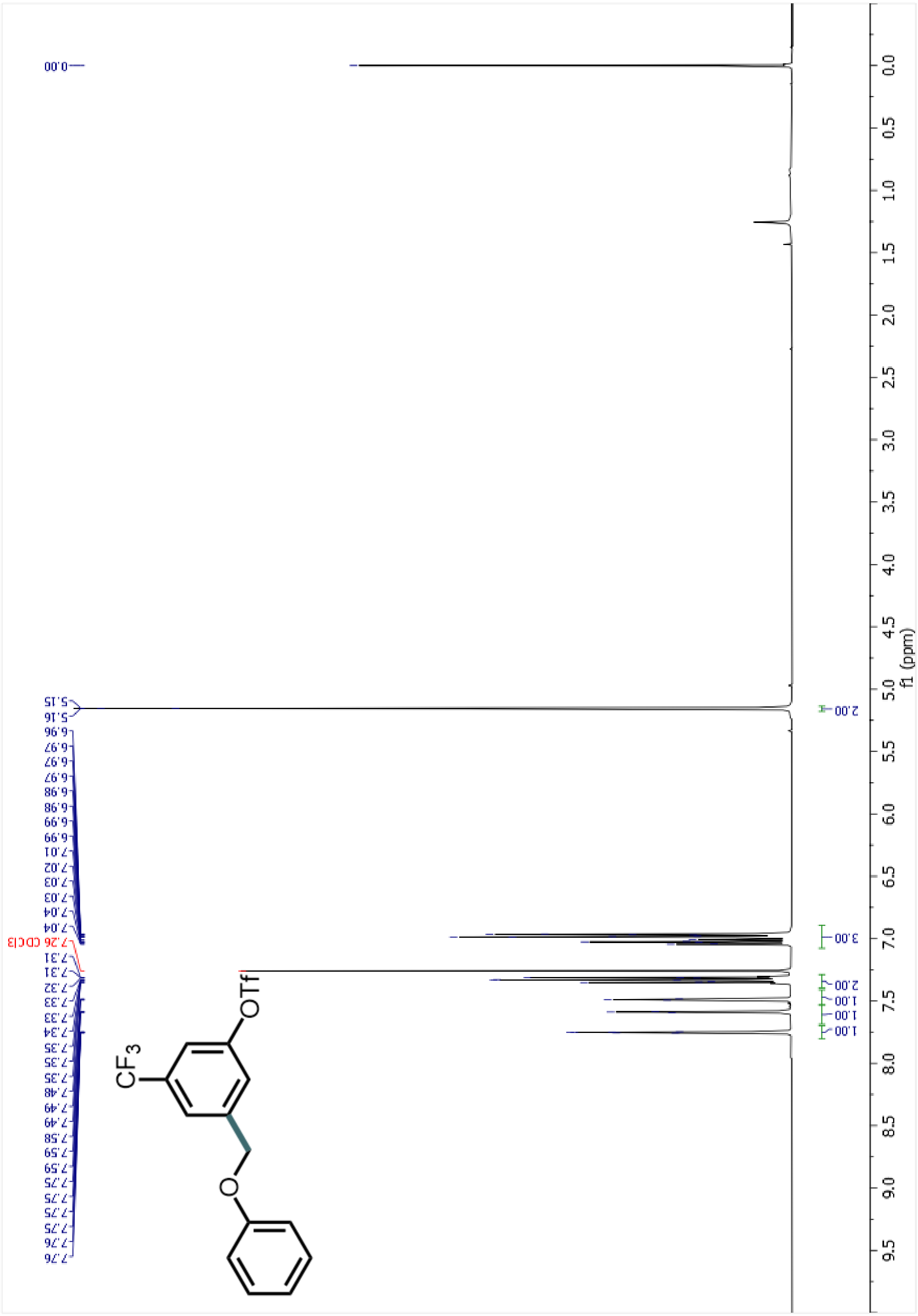

$^{13}C\{^{1}H\}$ NMR SPECTRUM OF **3** (101 MHz, $CDCl_3$):

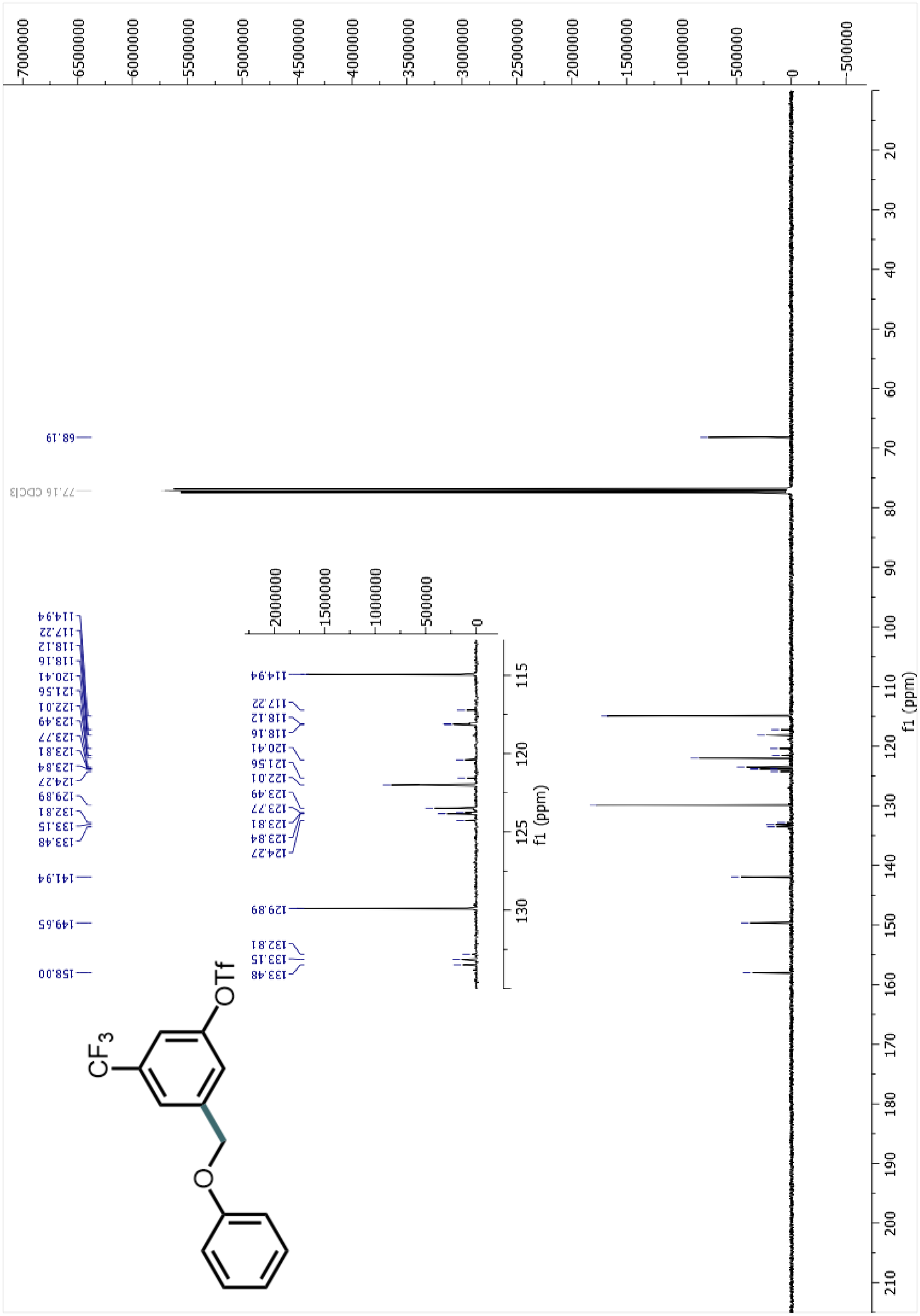

^{19}F NMR SPECTRUM OF **3** (377 MHz, $CDCl_3$):

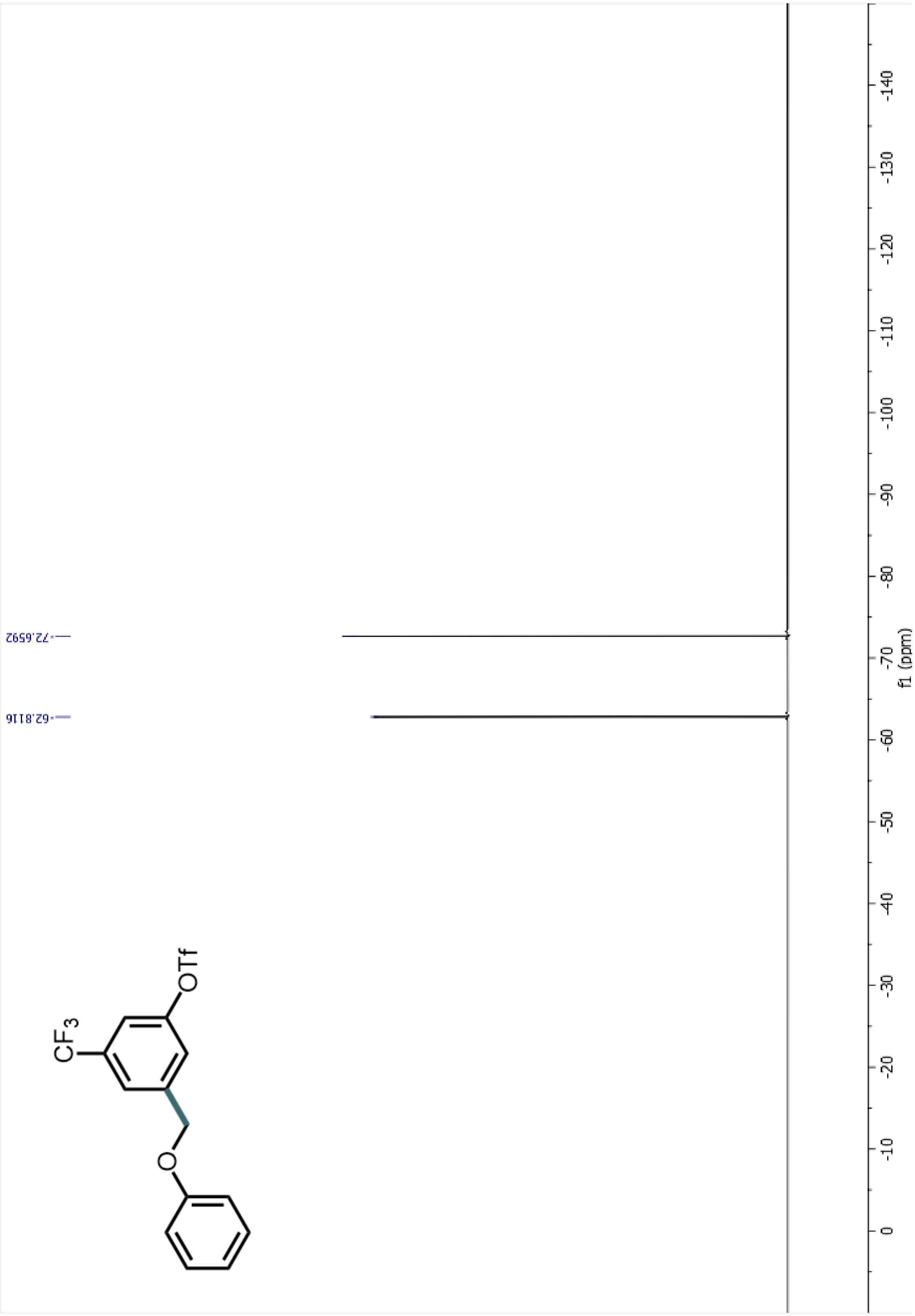

^{1}H NMR SPECTRUM OF **4** (400 MHz, $CDCl_3$):

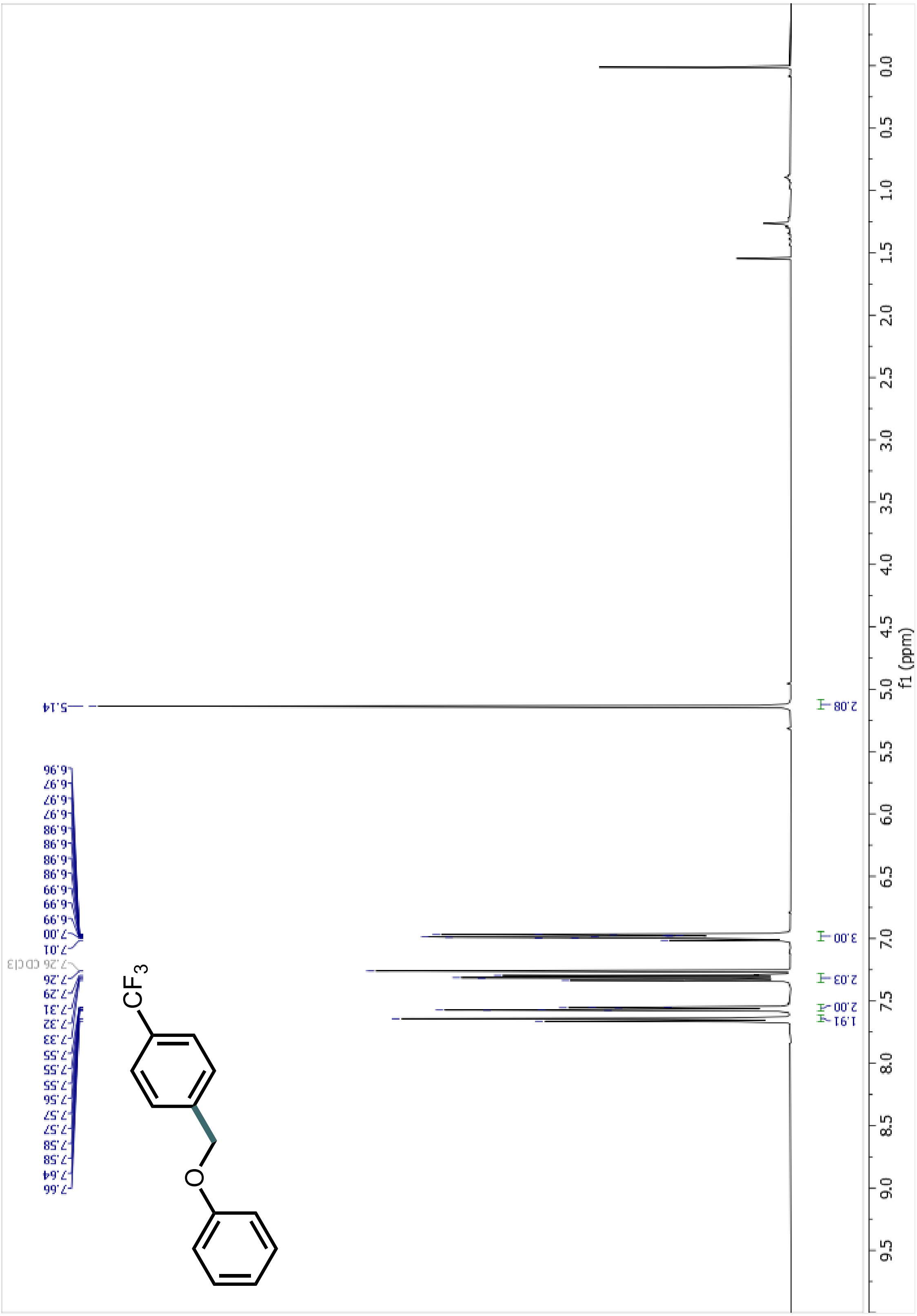

$^{13}C\{^{1}H\}$ NMR SPECTRUM OF **4** (101 MHz, $CDCl_3$):

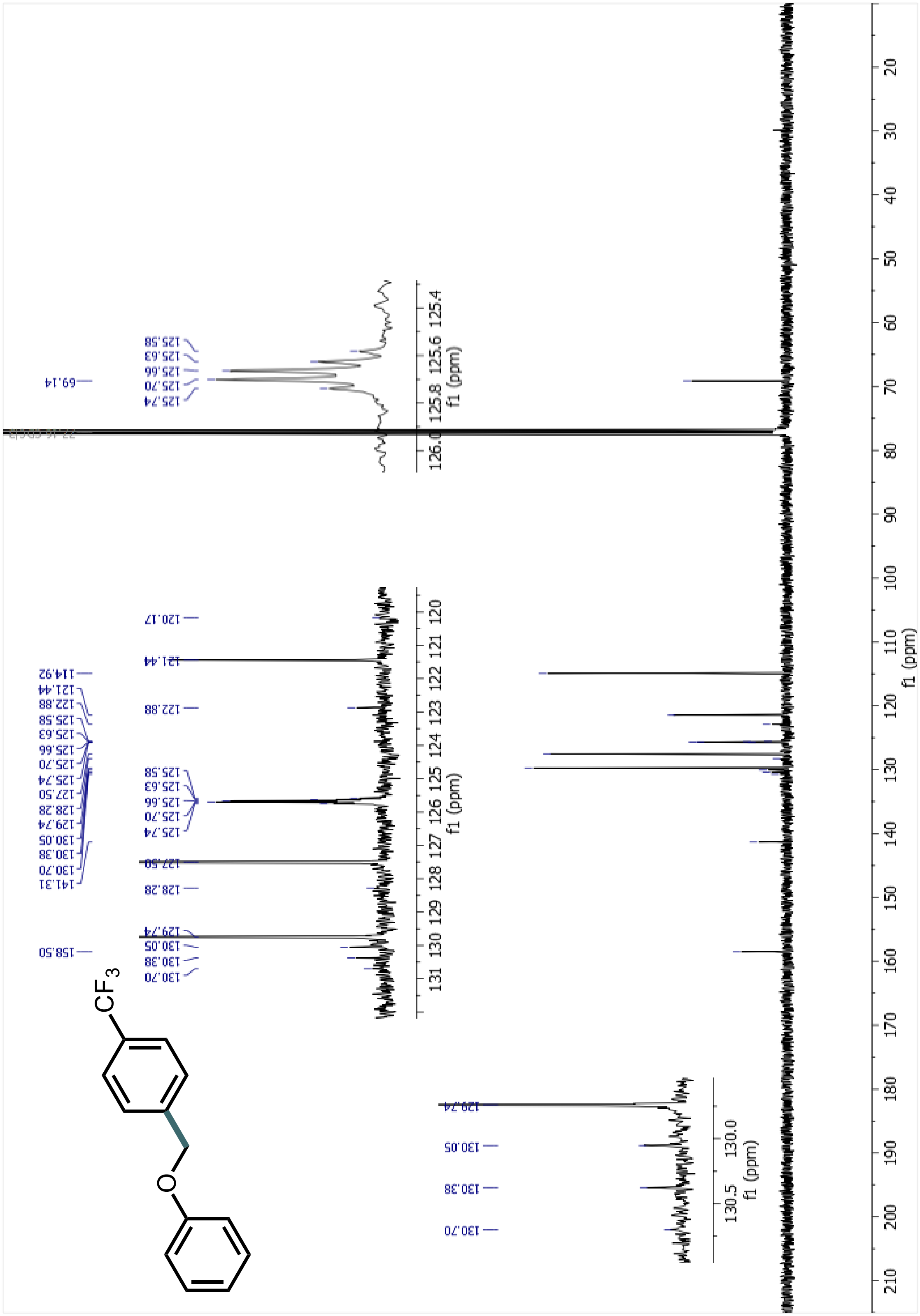

^{19}F NMR SPECTRUM OF **4** (377 MHz, $CDCl_3$):

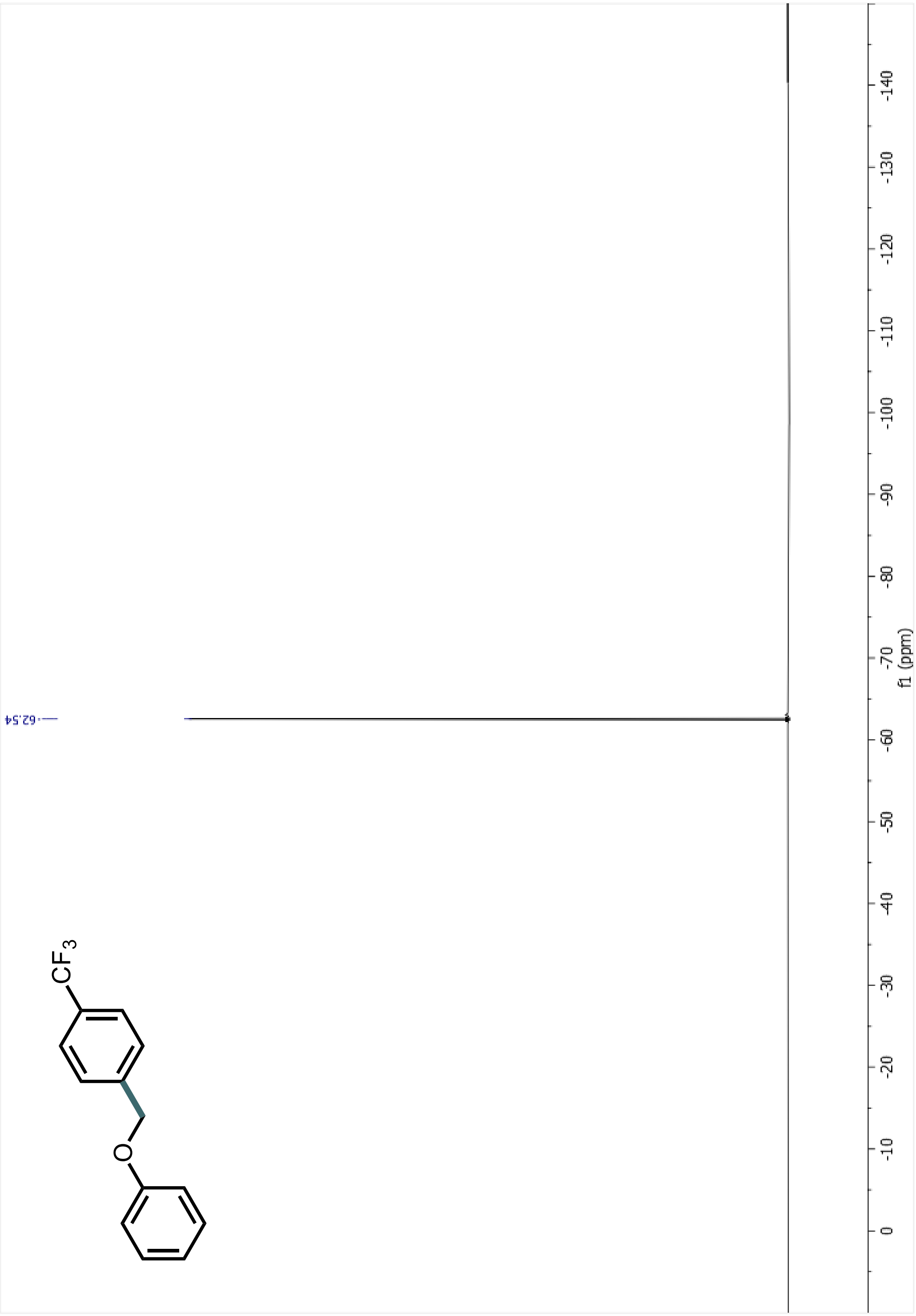

^{1}H NMR SPECTRUM OF **5** (400 MHz, $CDCl_3$):

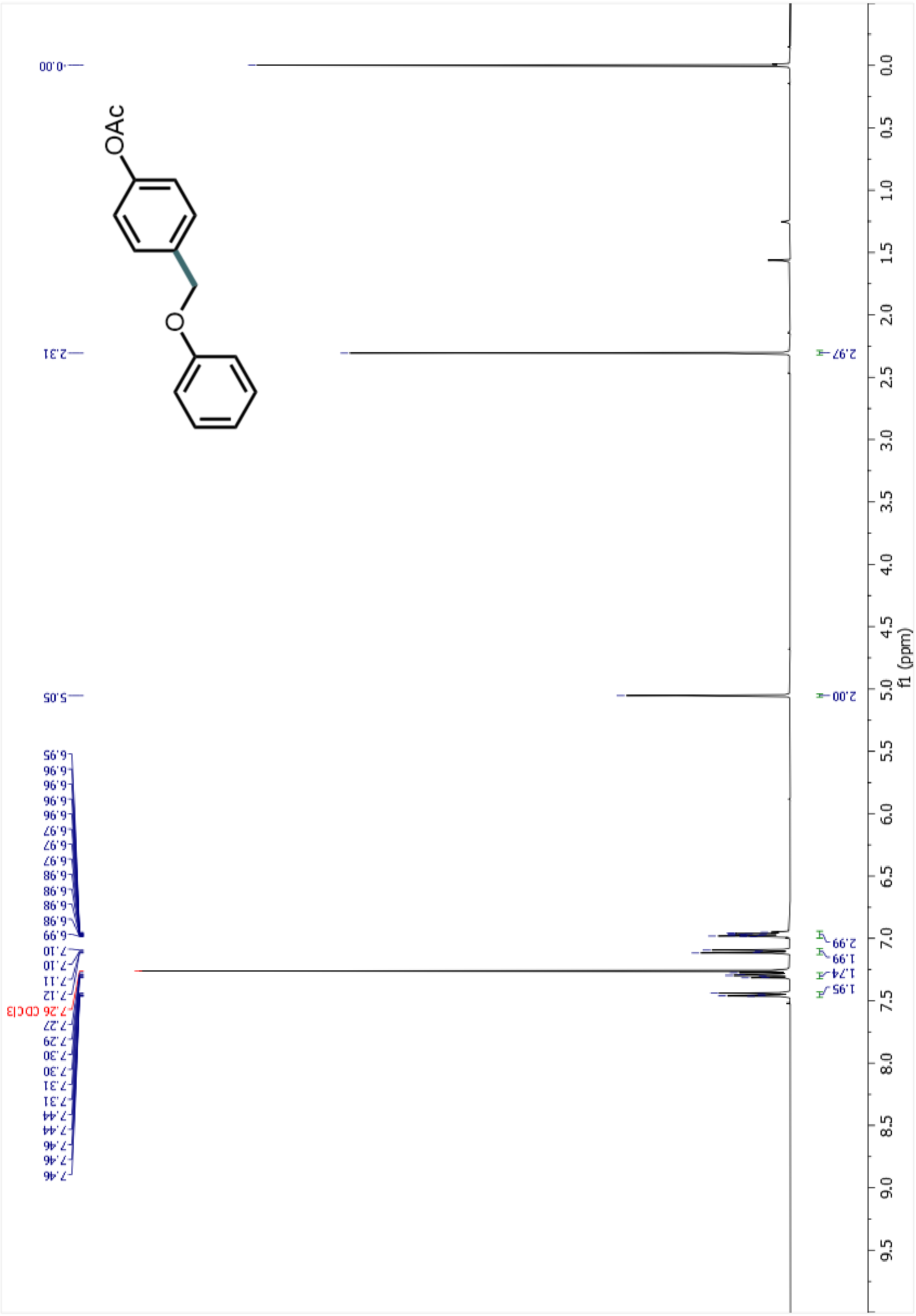

$^{13}C\{^{1}H\}$ NMR SPECTRUM OF **5** (101 MHz, $CDCl_3$):

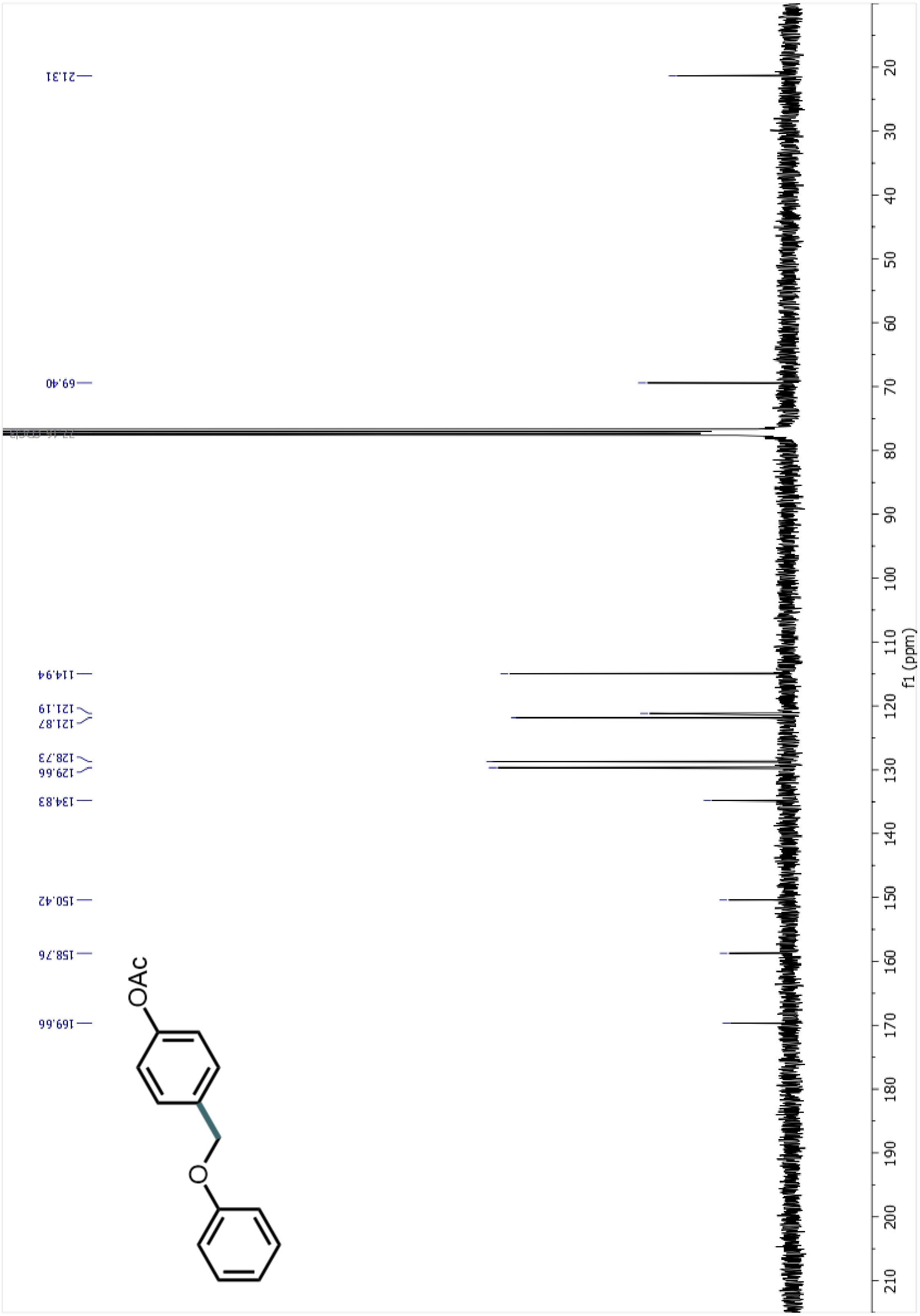

^{1}H NMR SPECTRUM OF **6** (400 MHz, $CDCl_3$):

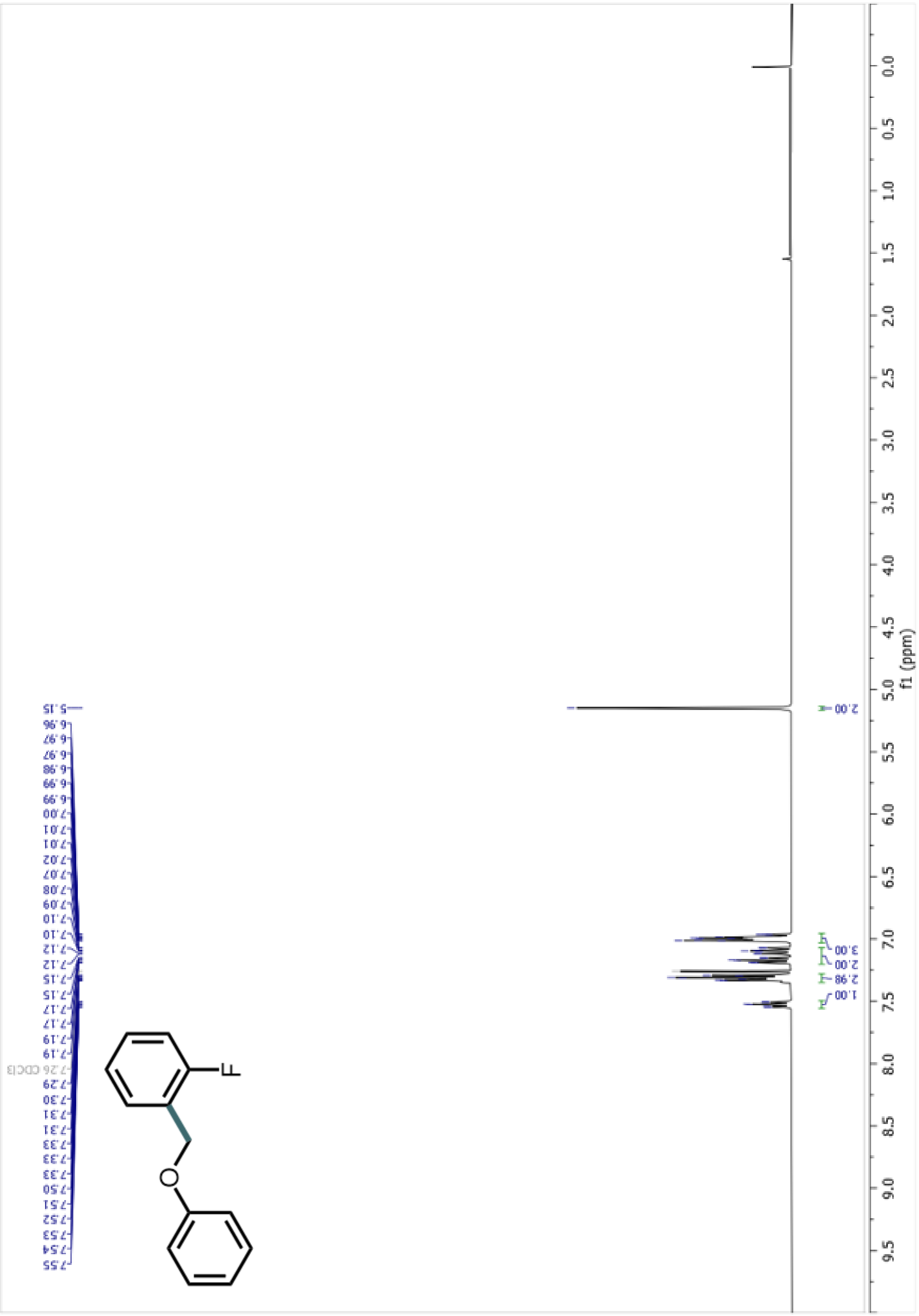

$^{13}C\{^{1}H\}$ NMR SPECTRUM OF **6** (101 MHz, $CDCl_3$):

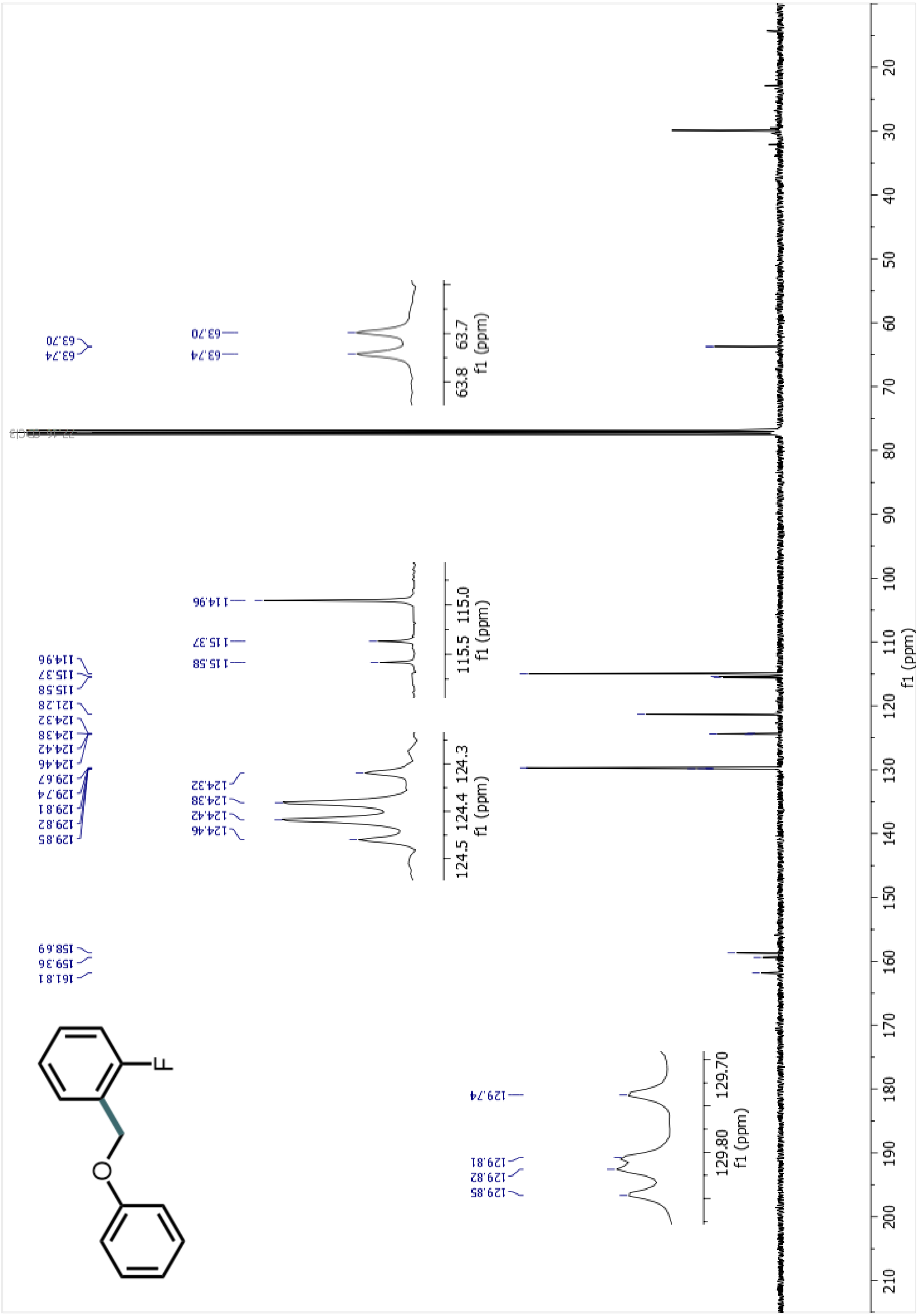

^{19}F NMR SPECTRUM OF **6** (377 MHz, $CDCl_3$):

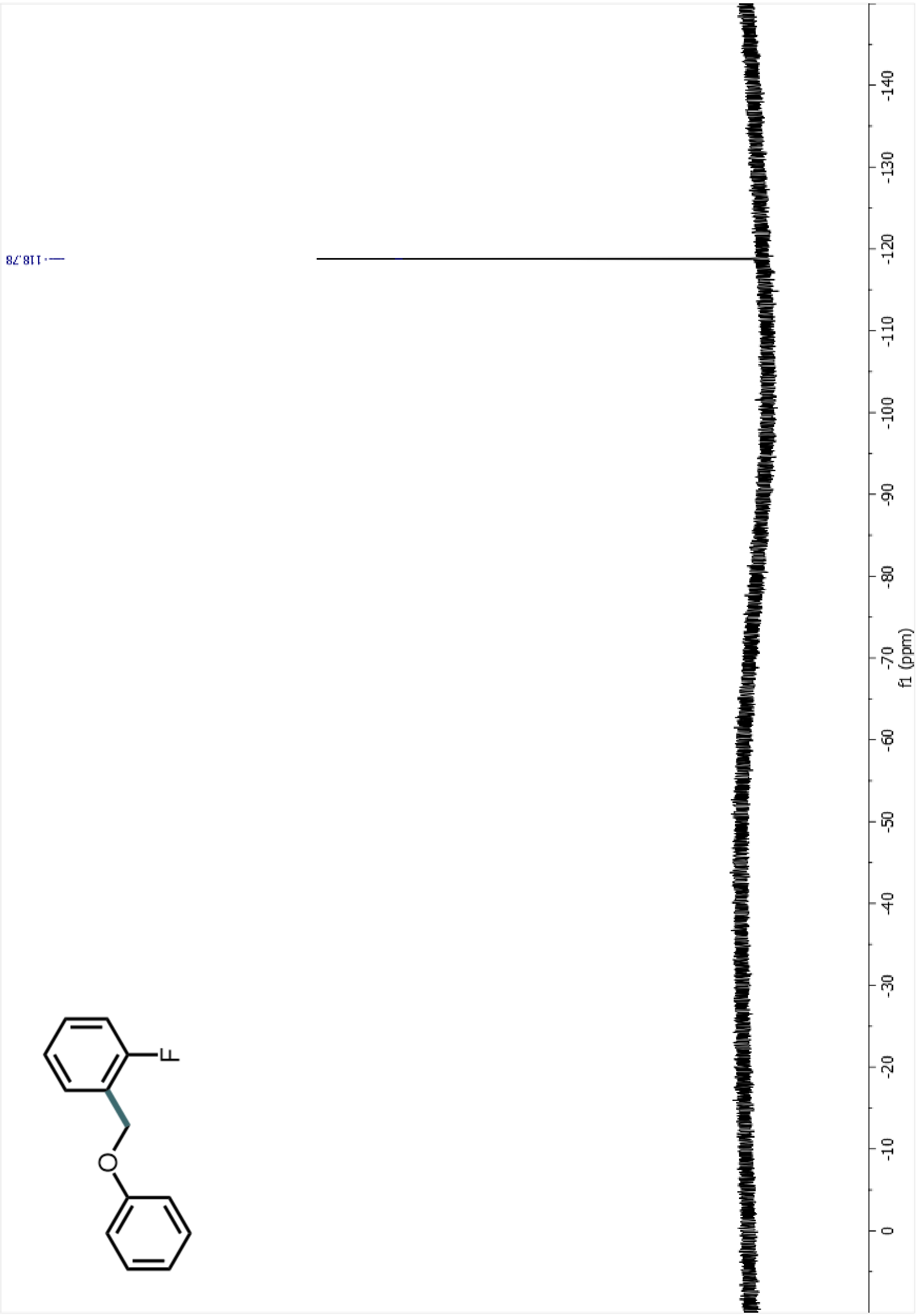

^{1}H NMR SPECTRUM OF **7** (377 MHz, $CDCl_3$):

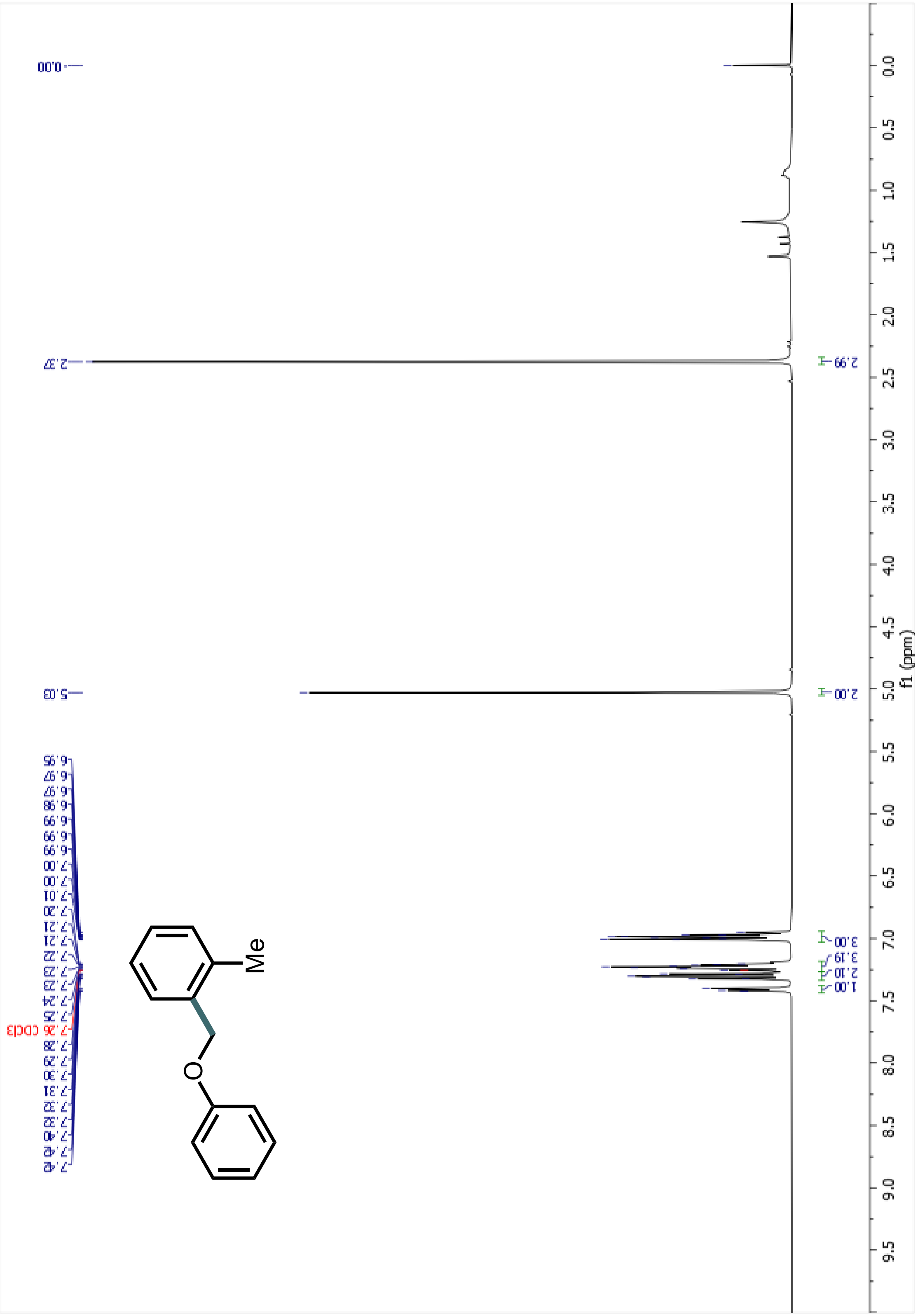

$^{13}C\{^{1}H\}$ NMR SPECTRUM OF **7** (101 MHz, $CDCl_3$):

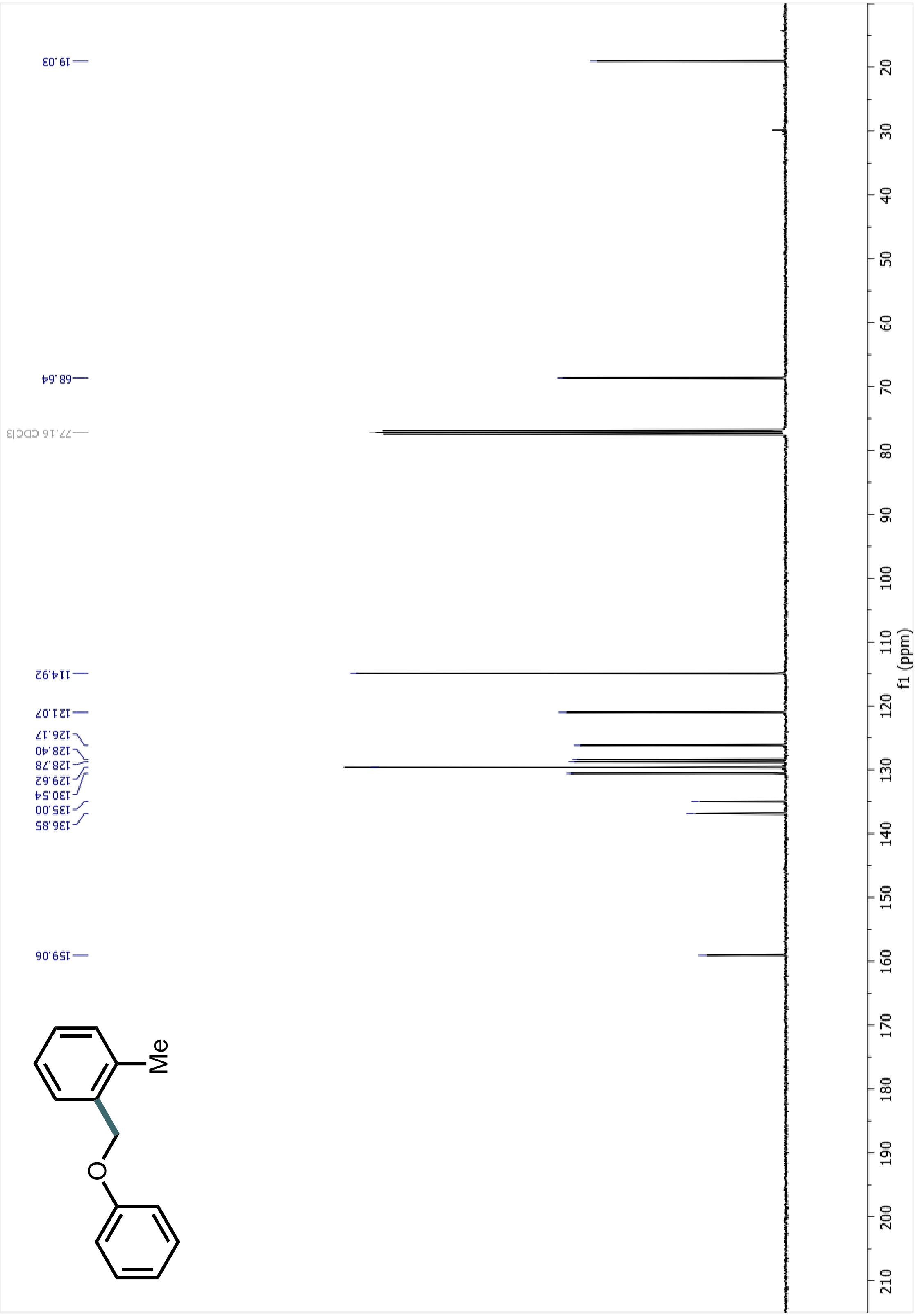

^{1}H NMR SPECTRUM OF **8** (400 MHz, $CDCl_3$):

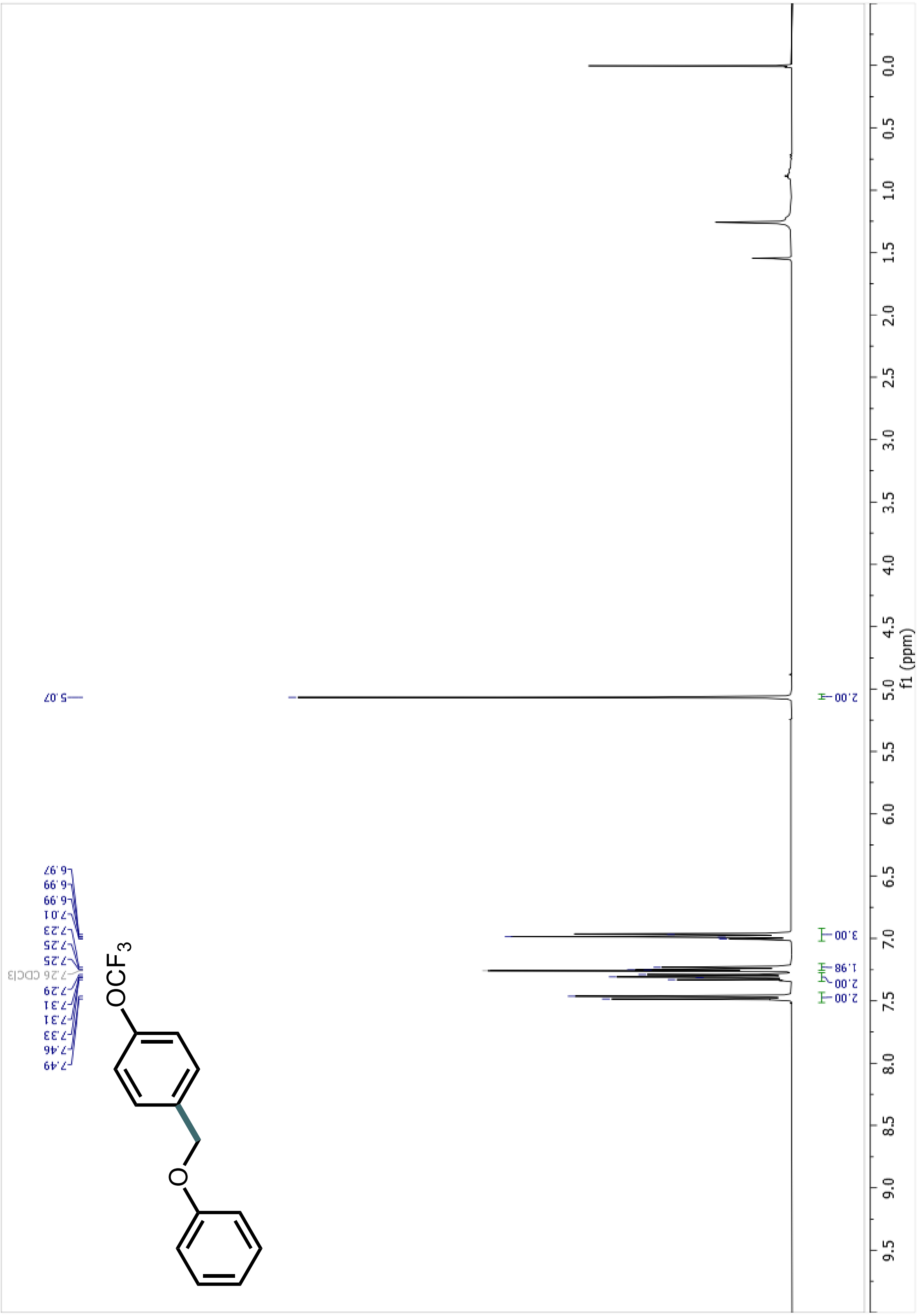

$^{13}C\{^{1}H\}$ NMR SPECTRUM OF **8** (101 MHz, $CDCl_3$):

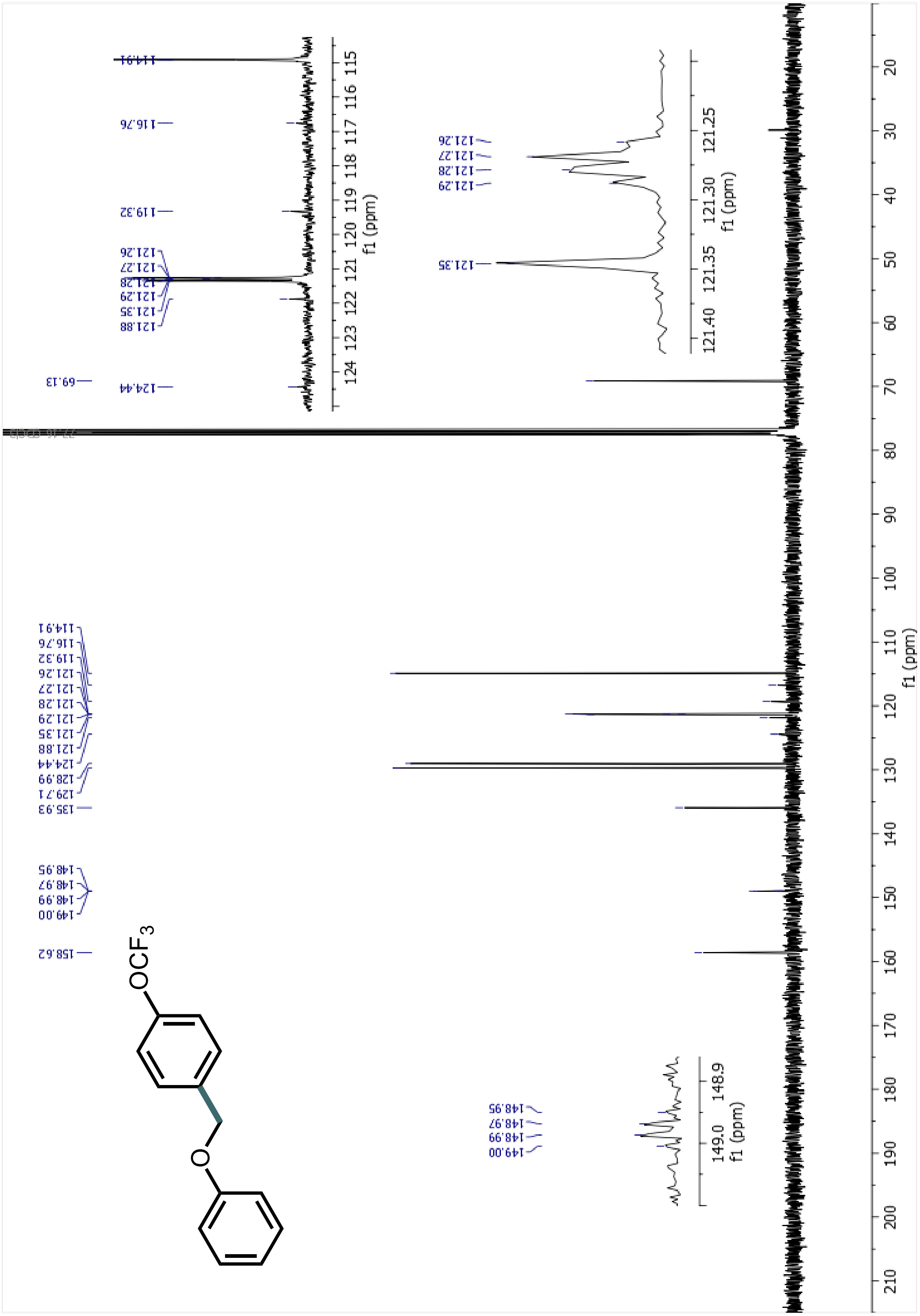

^{19}F NMR SPECTRUM OF **8** (377 MHz, $CDCl_3$):

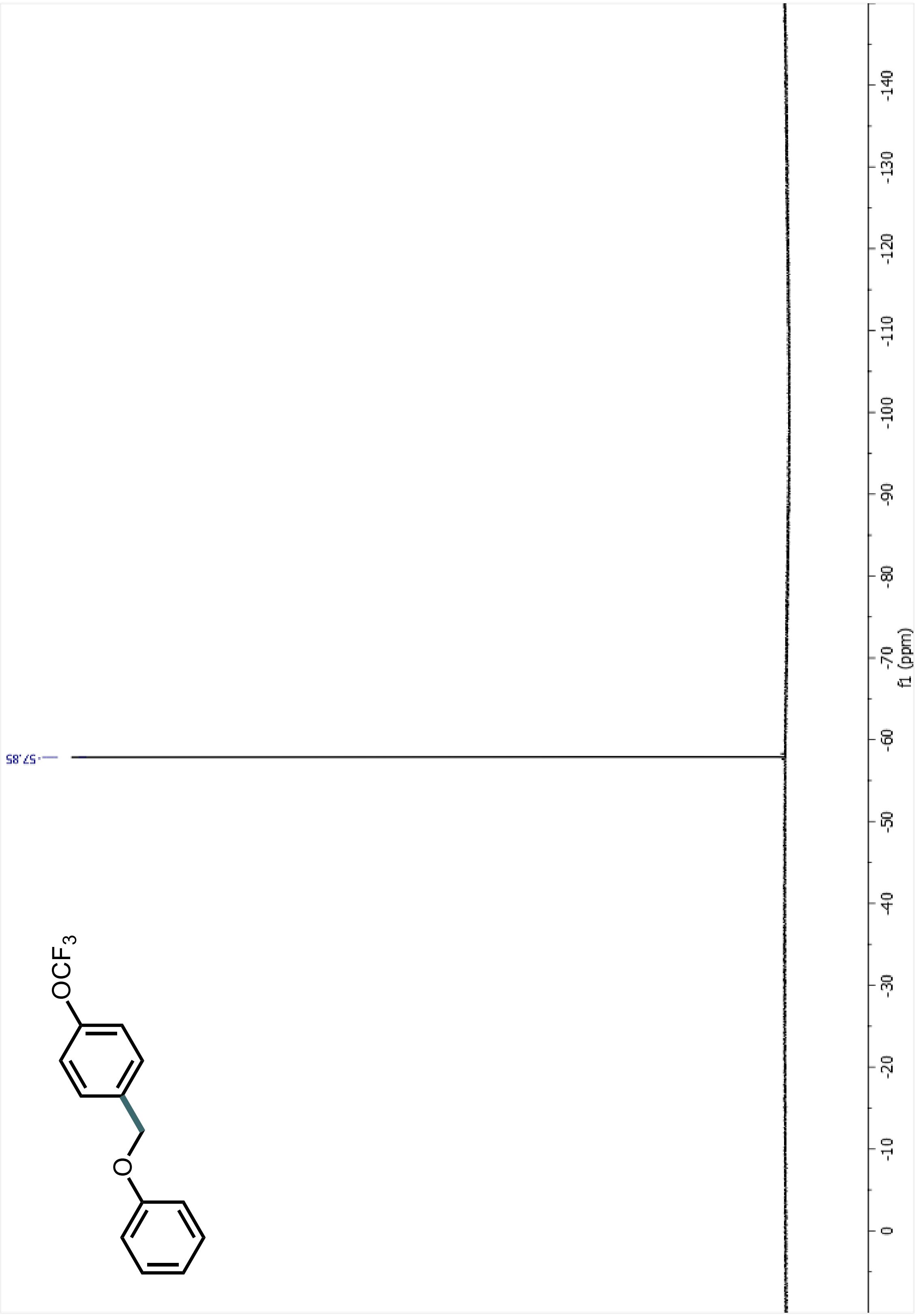

^{1}H NMR SPECTRUM OF **9** (400 MHz, $CDCl_3$):

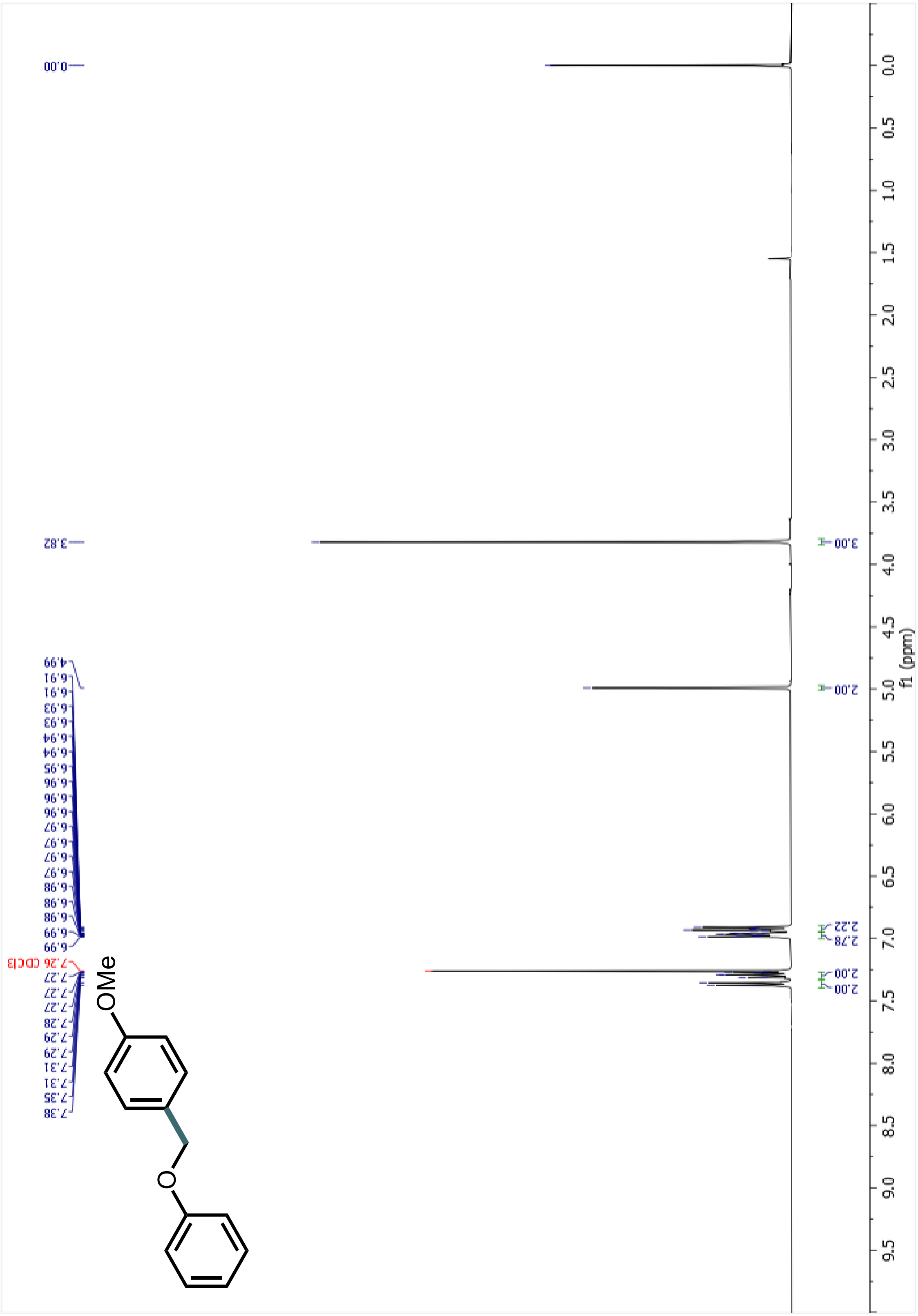

$^{13}C\{1H\}$ NMR SPECTRUM OF **9** (101 MHz, $CDCl_3$):

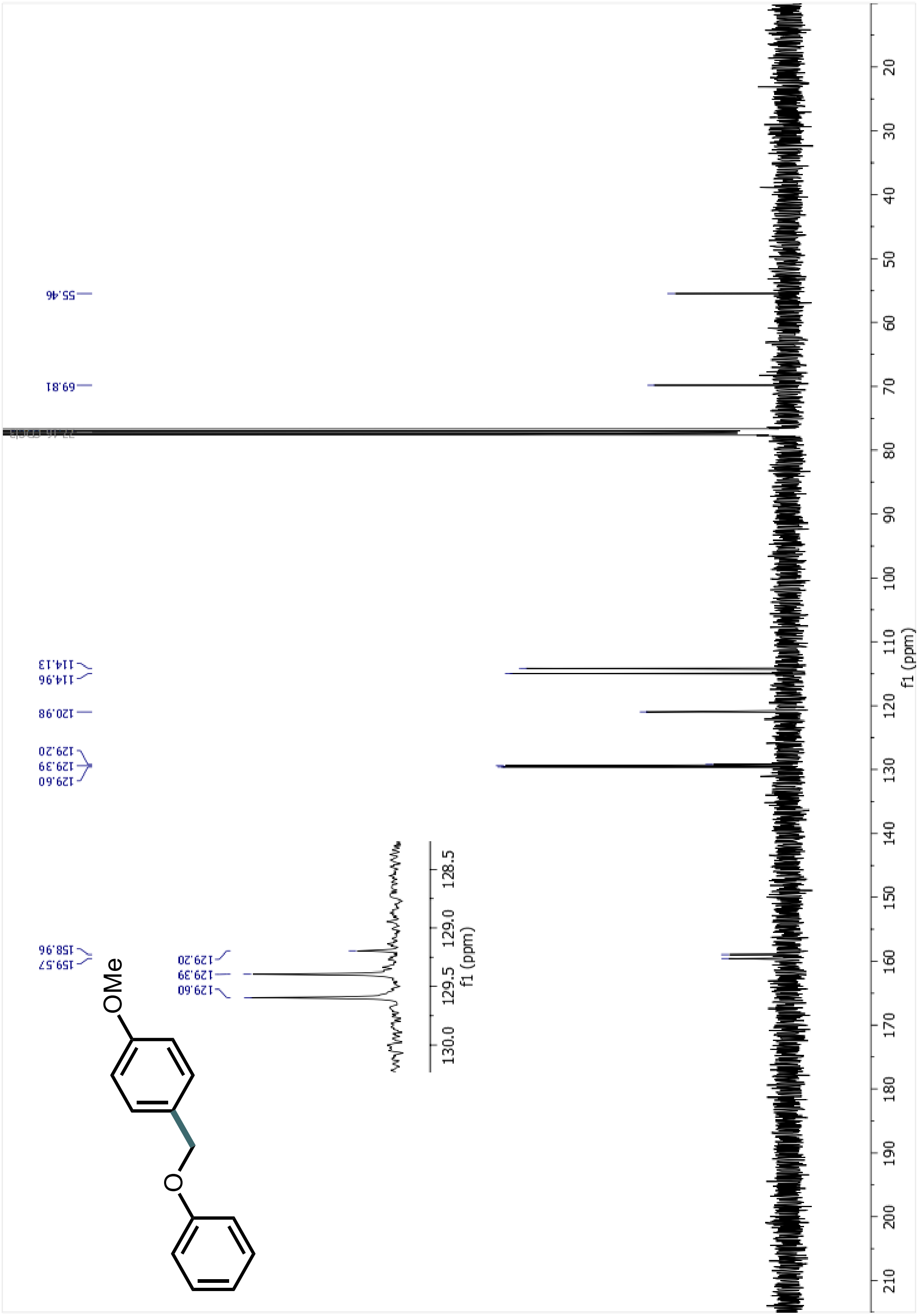

^{1}H NMR SPECTRUM OF **10** (400 MHz, $CDCl_3$):

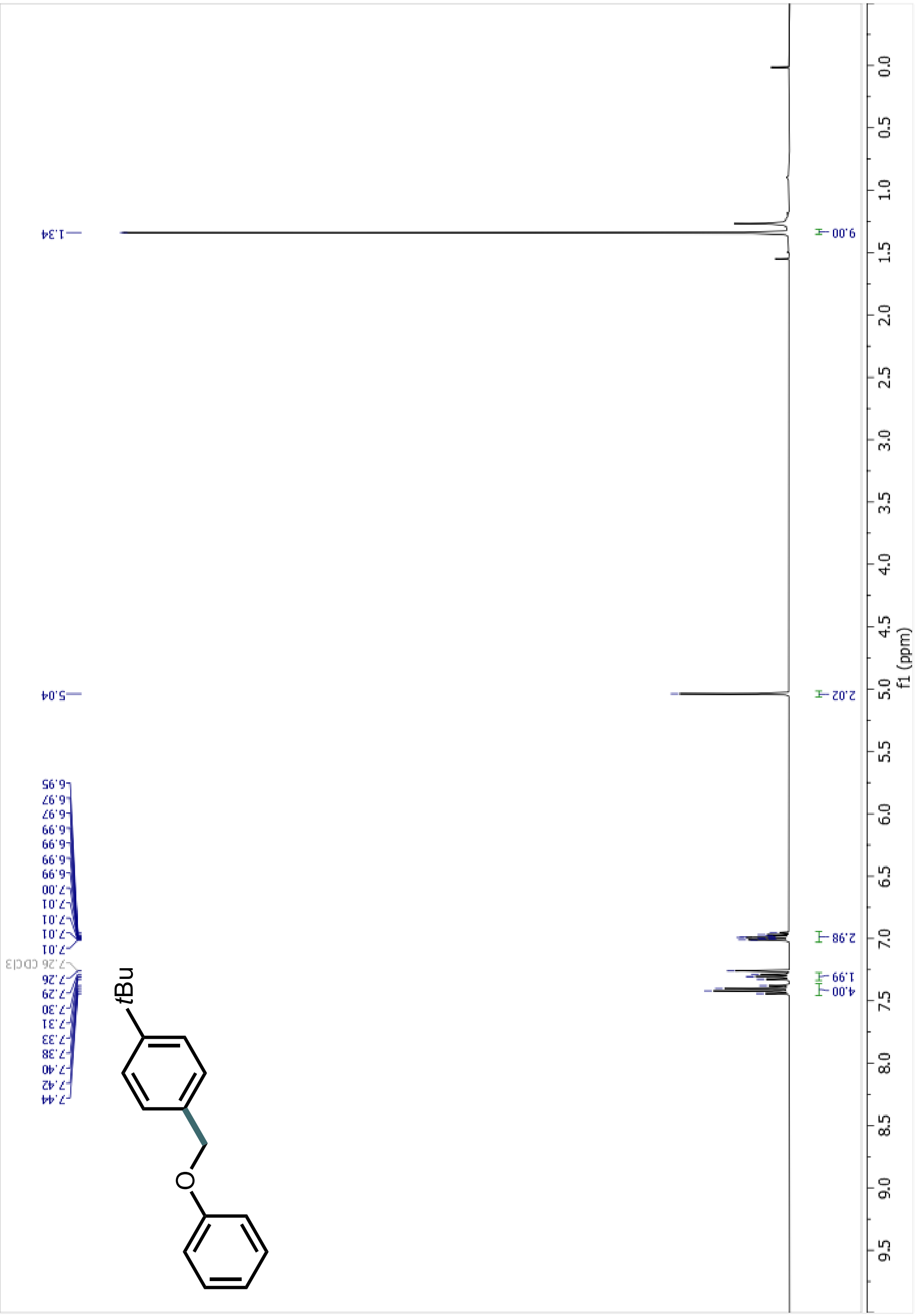

$^{13}C\{^{1}H\}$ NMR SPECTRUM OF **10** (101 MHz, $CDCl_3$):

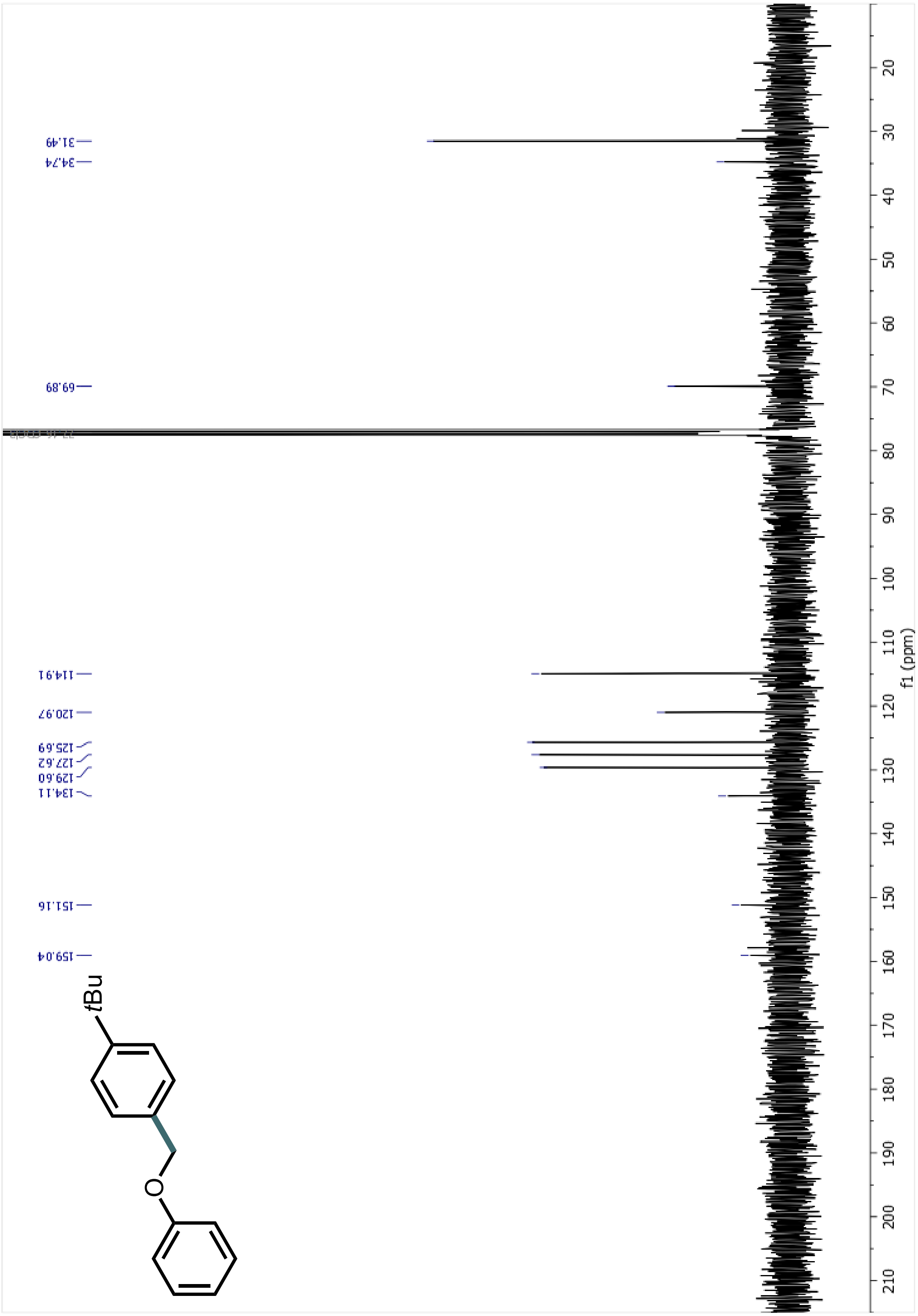

^{1}H NMR SPECTRUM OF **11** (400 MHz, $CDCl_3$):

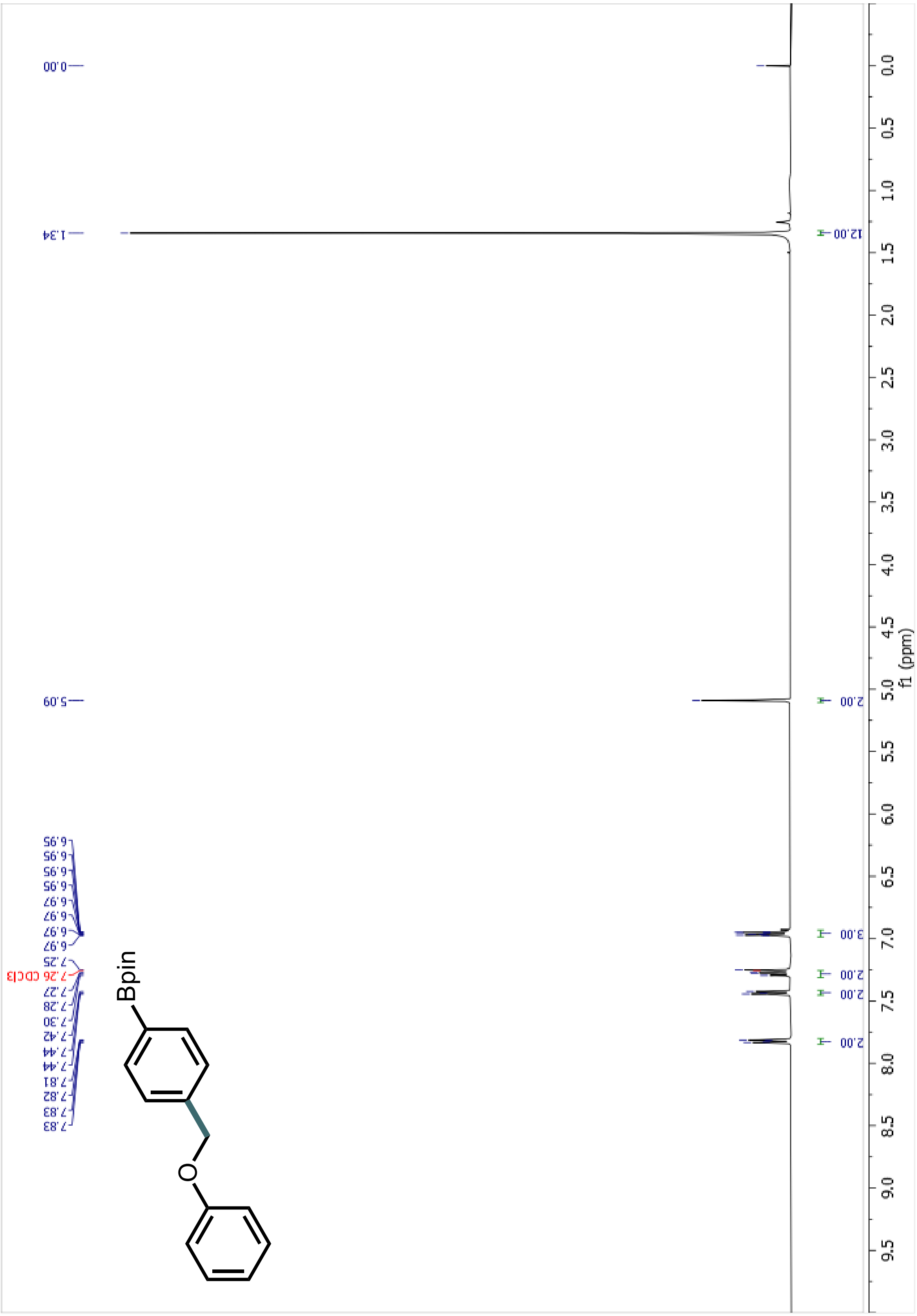

$^{13}C\{^{1}H\}$ NMR SPECTRUM OF **11** (101 MHz, $CDCl_3$):

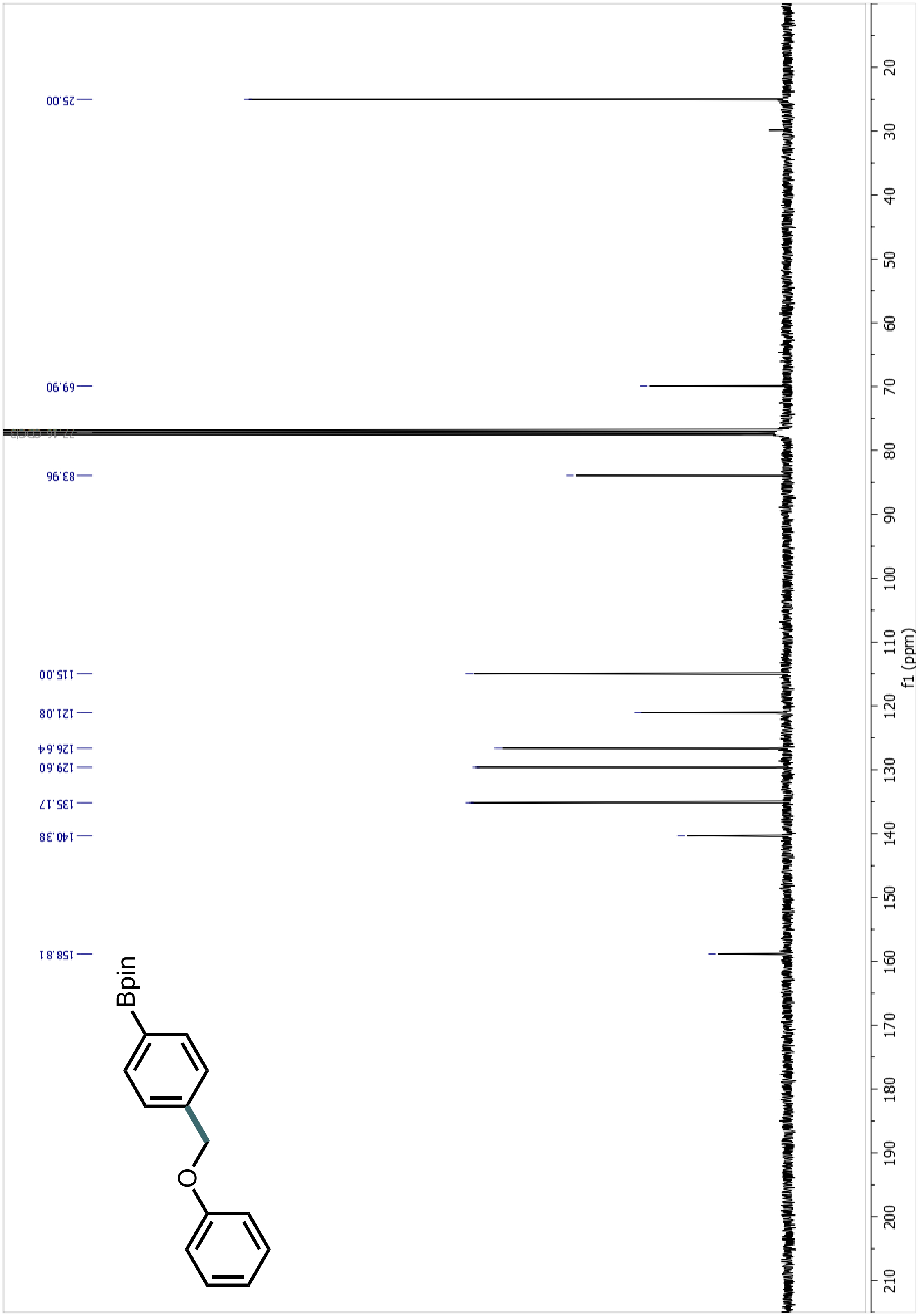

^{11}B NMR SPECTRUM OF **11** (128 MHz, $CDCl_3$):

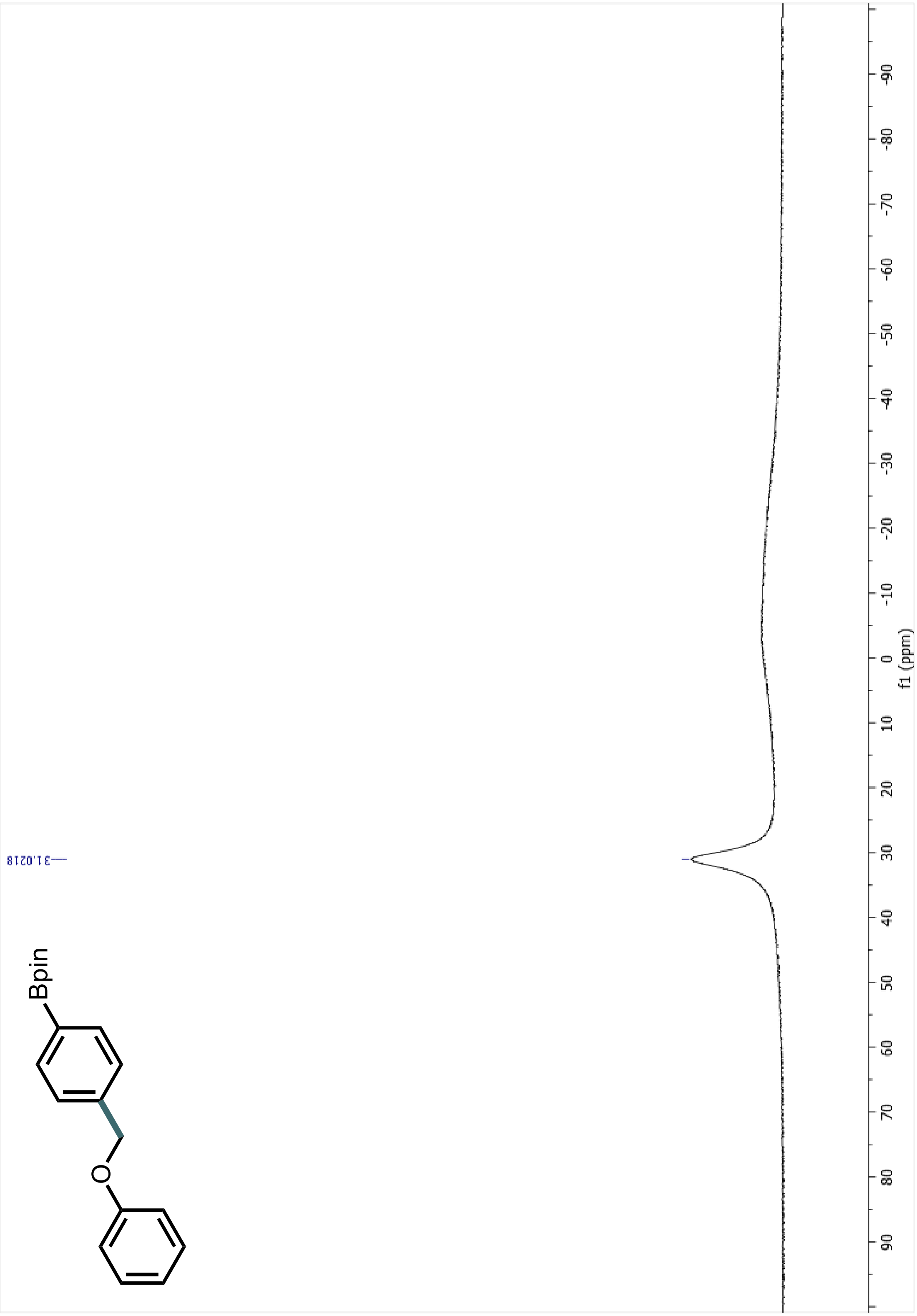

^{1}H NMR SPECTRUM OF **12** (400 MHz, $CDCl_3$):

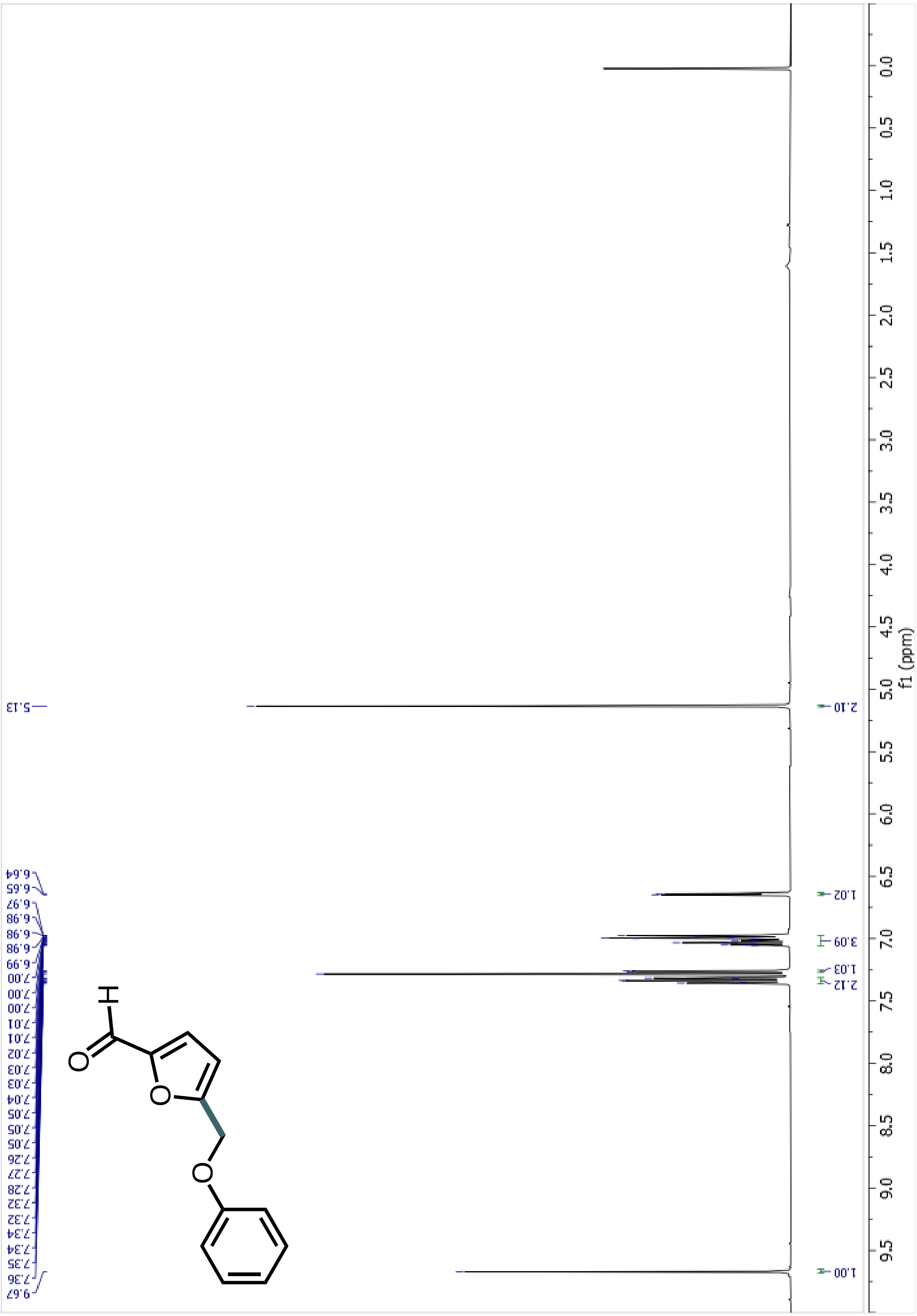

$^{13}C\{^{1}H\}$ NMR SPECTRUM OF **12** (101 MHz, $CDCl_3$):

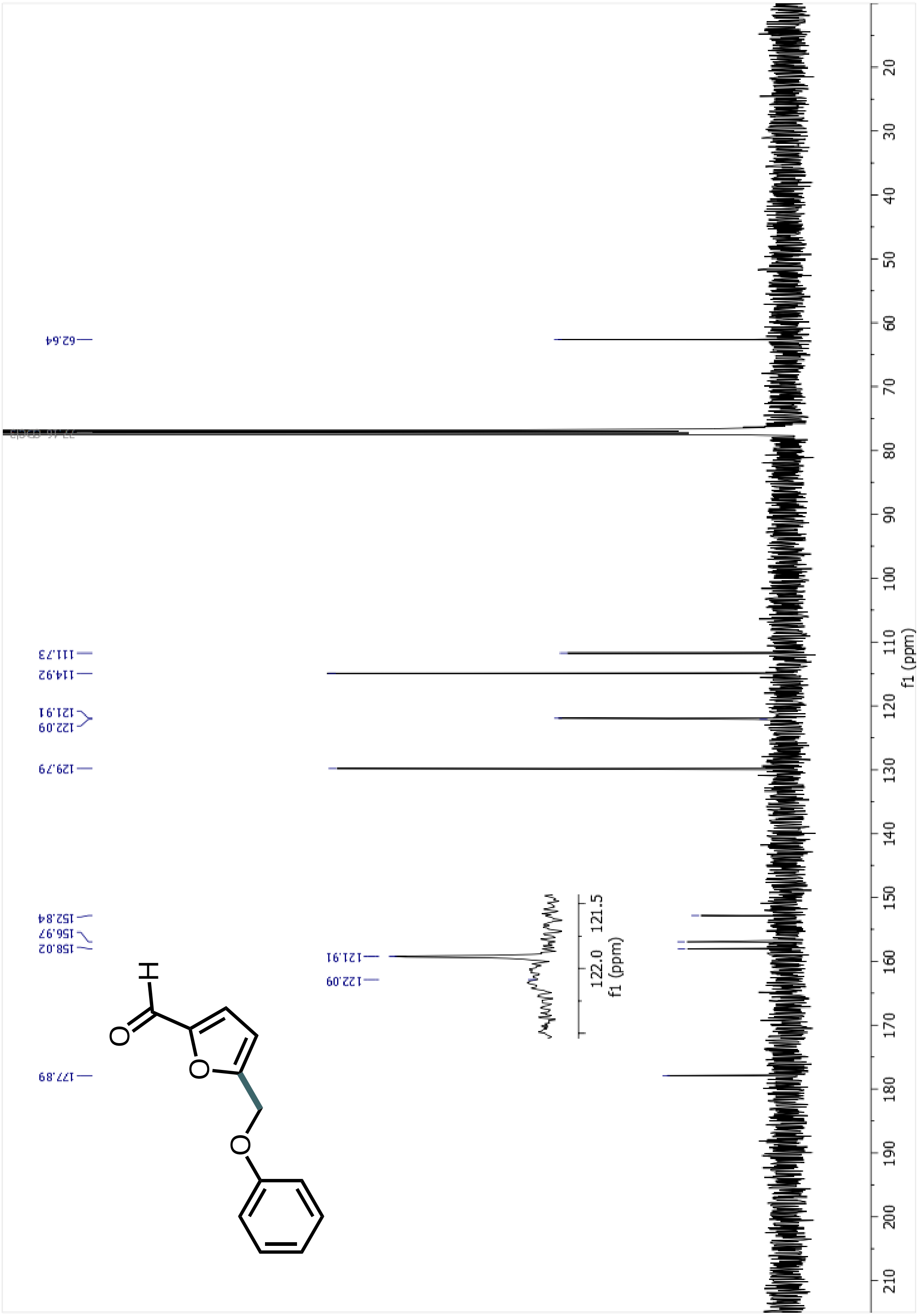

^{1}H-^{13}C HSQC of **12**:

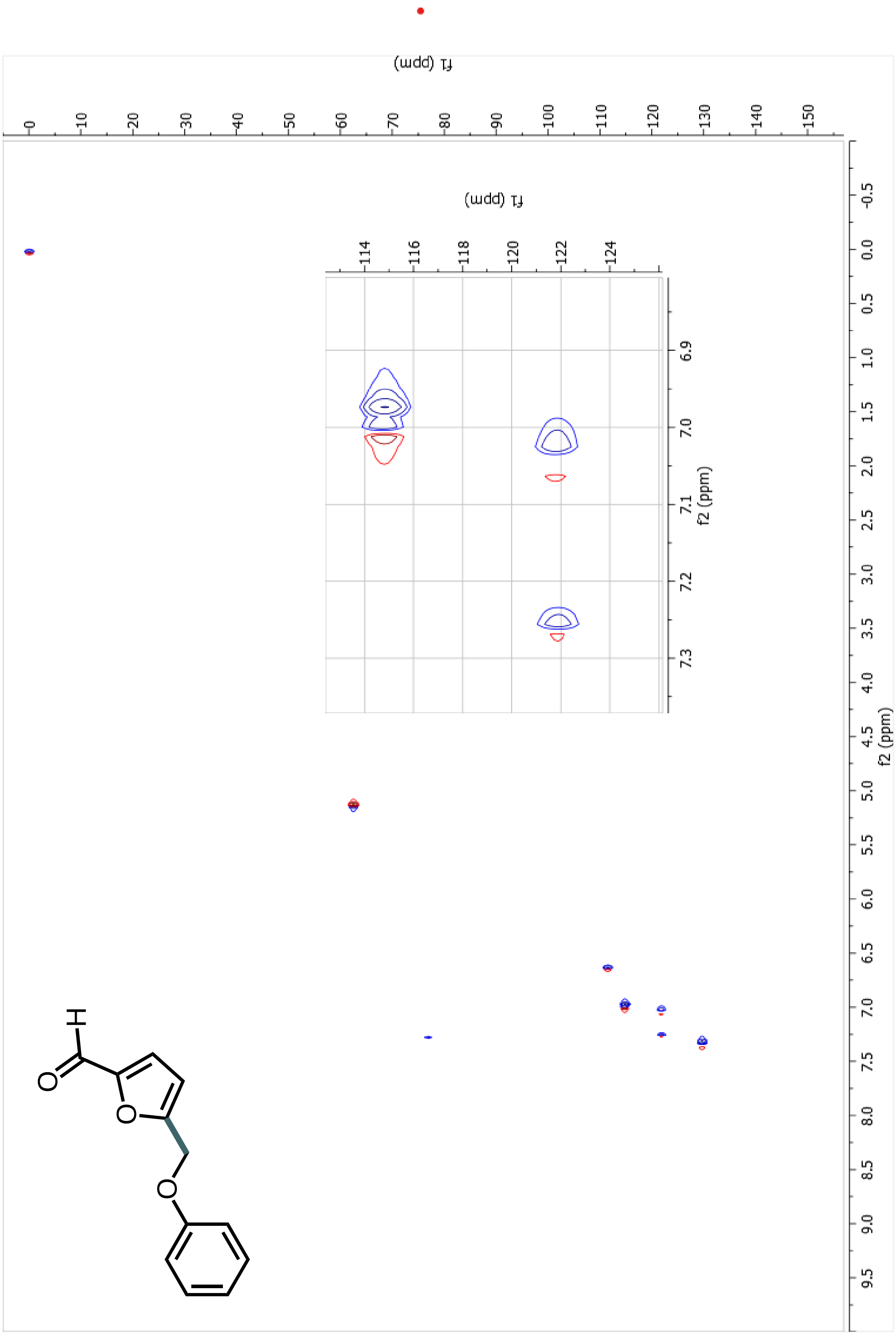

^{1}H NMR SPECTRUM OF **13** (400 MHz, $CDCl_3$):

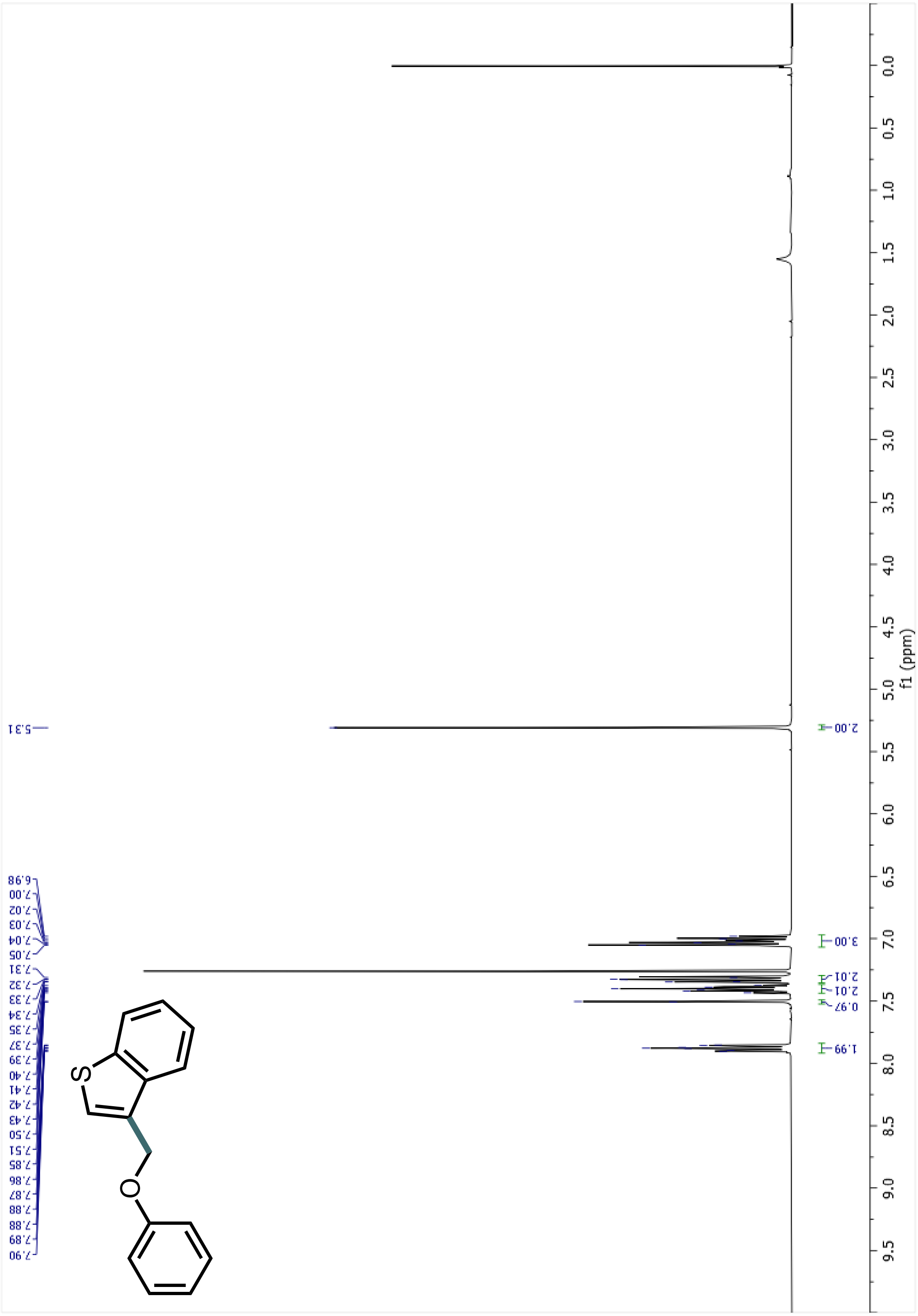

$^{13}C\{^1H\}$ NMR SPECTRUM OF **13** (101 MHz, $CDCl_3$):

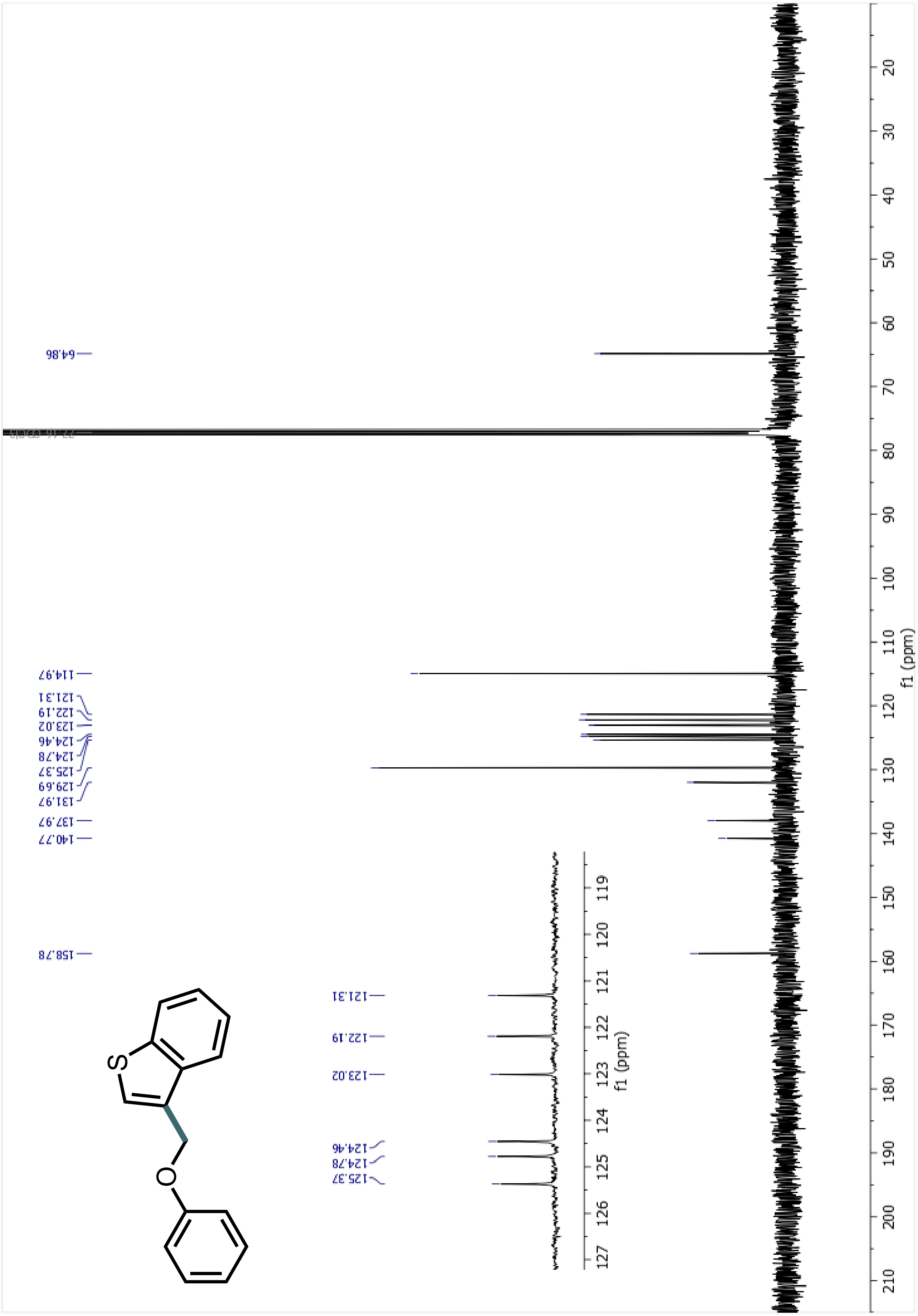

^{1}H NMR SPECTRUM OF **14** (400 MHz, $CDCl_3$):

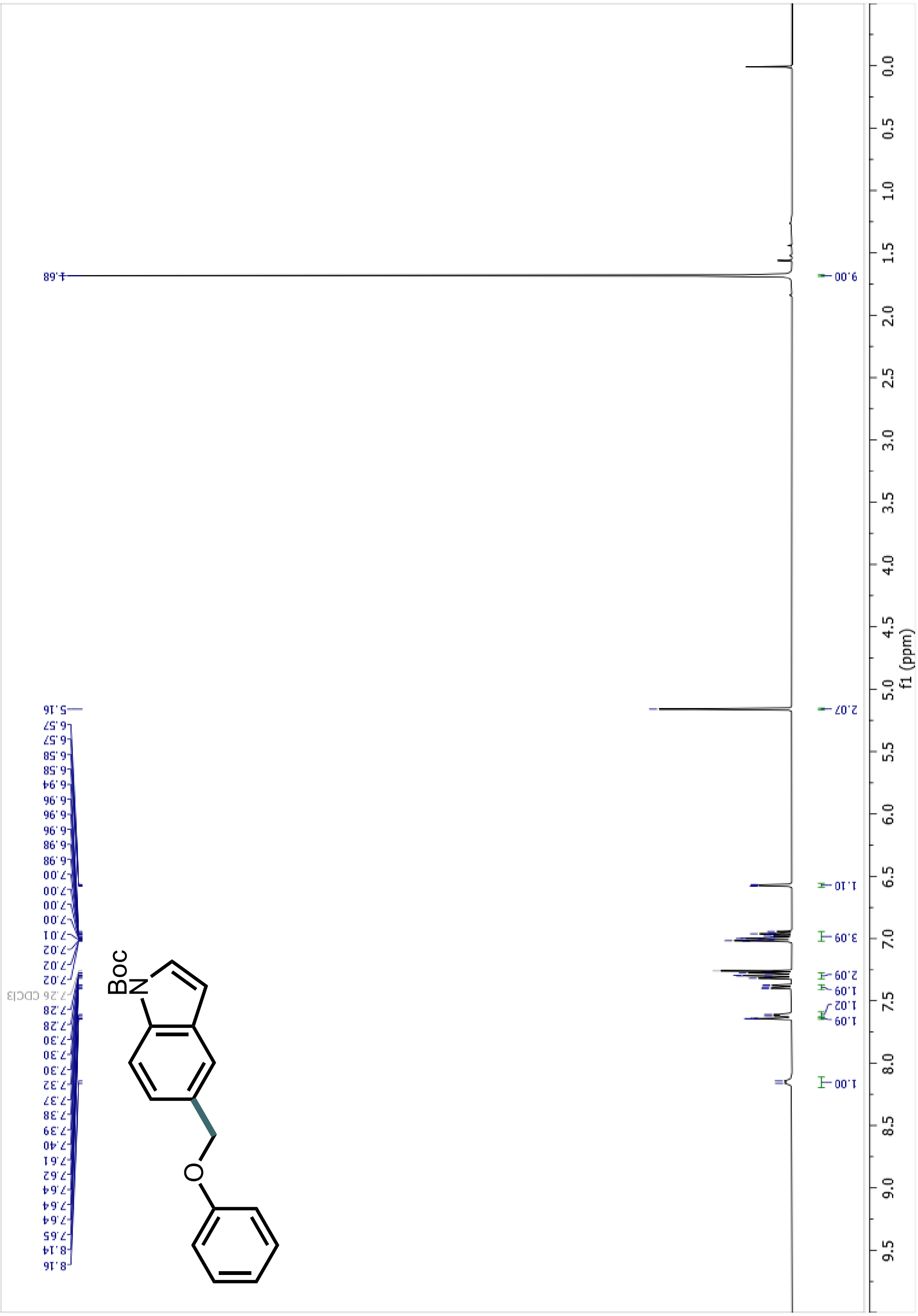

$^{13}C\{^{1}H\}$ NMR SPECTRUM OF **14** (101 MHz, $CDCl_3$):

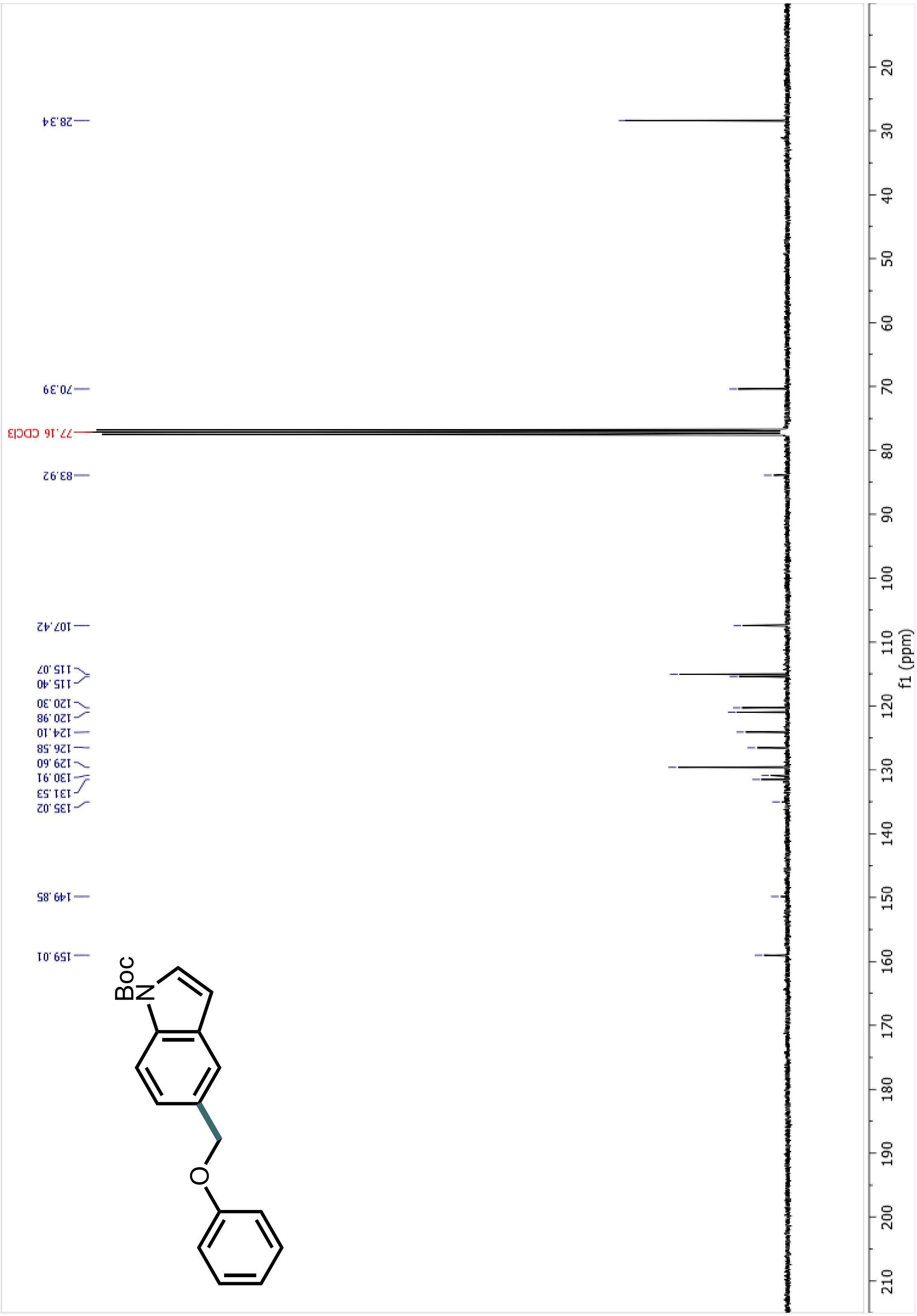

^{1}H NMR SPECTRUM OF **15** (400 MHz, $CDCl_3$):

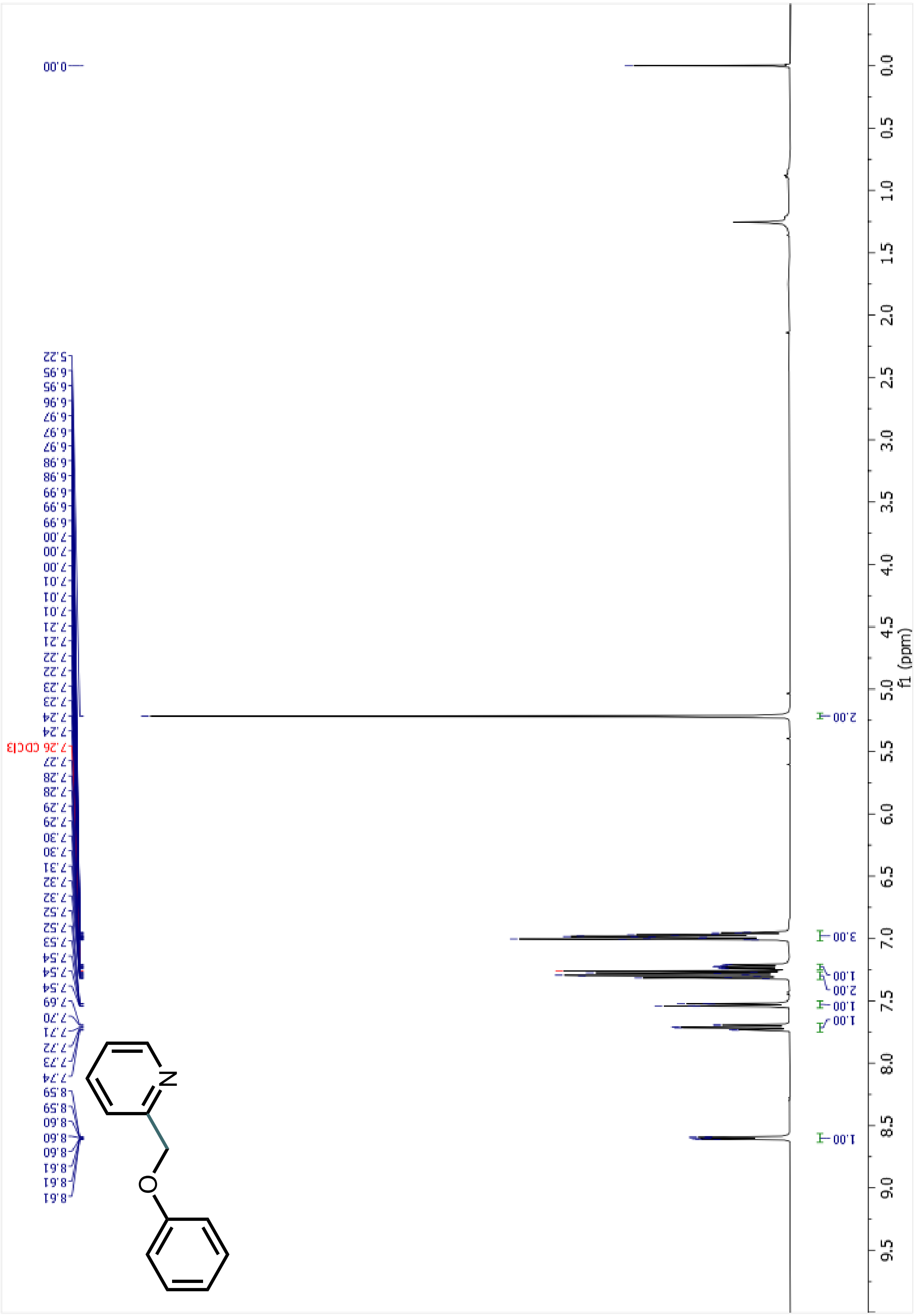

$^{13}C\{^{1}H\}$ NMR SPECTRUM OF **15** (101 MHz, $CDCl_3$):

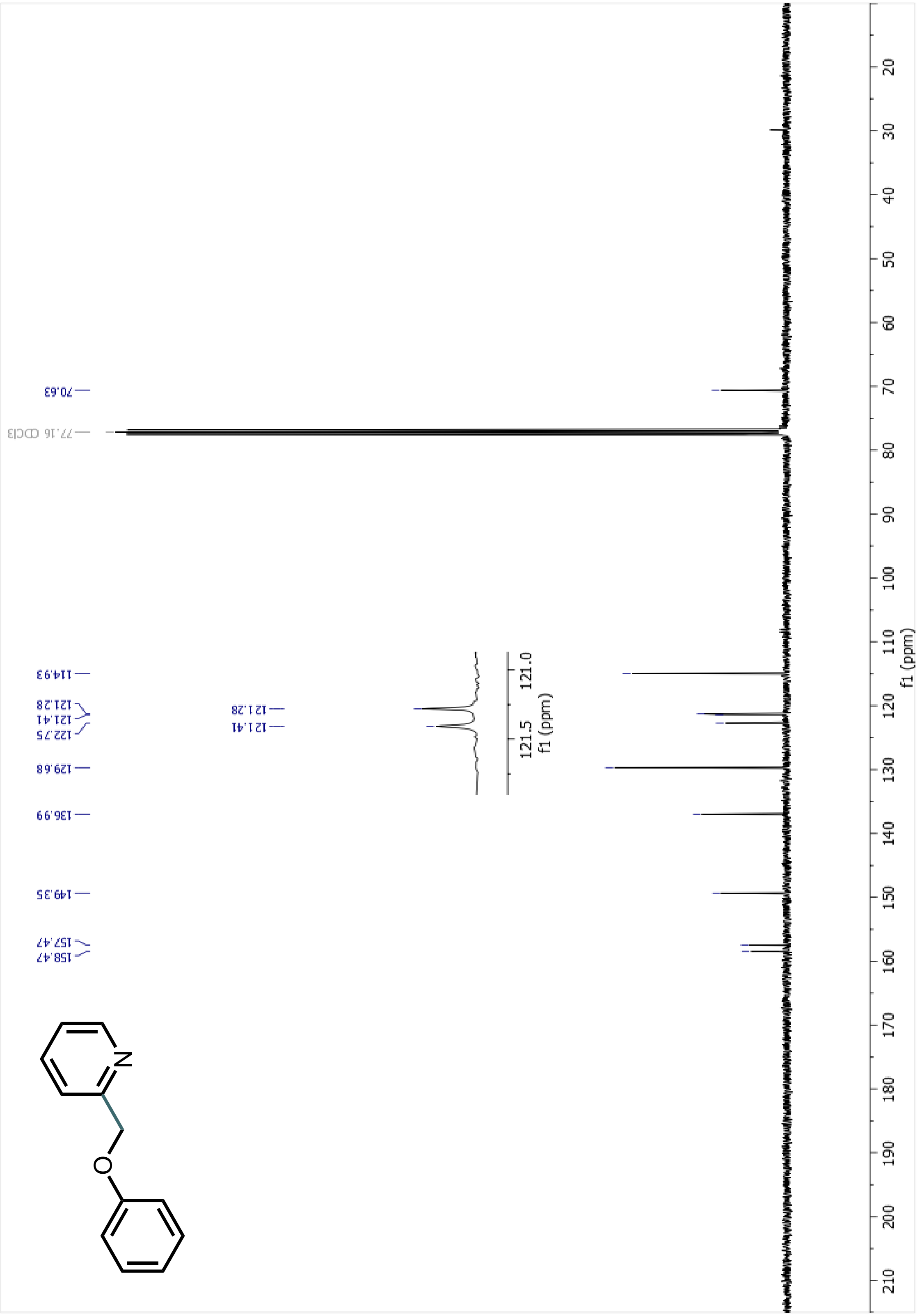

^{1}H NMR SPECTRUM OF **21A** (400 MHz, $CDCl_3$):

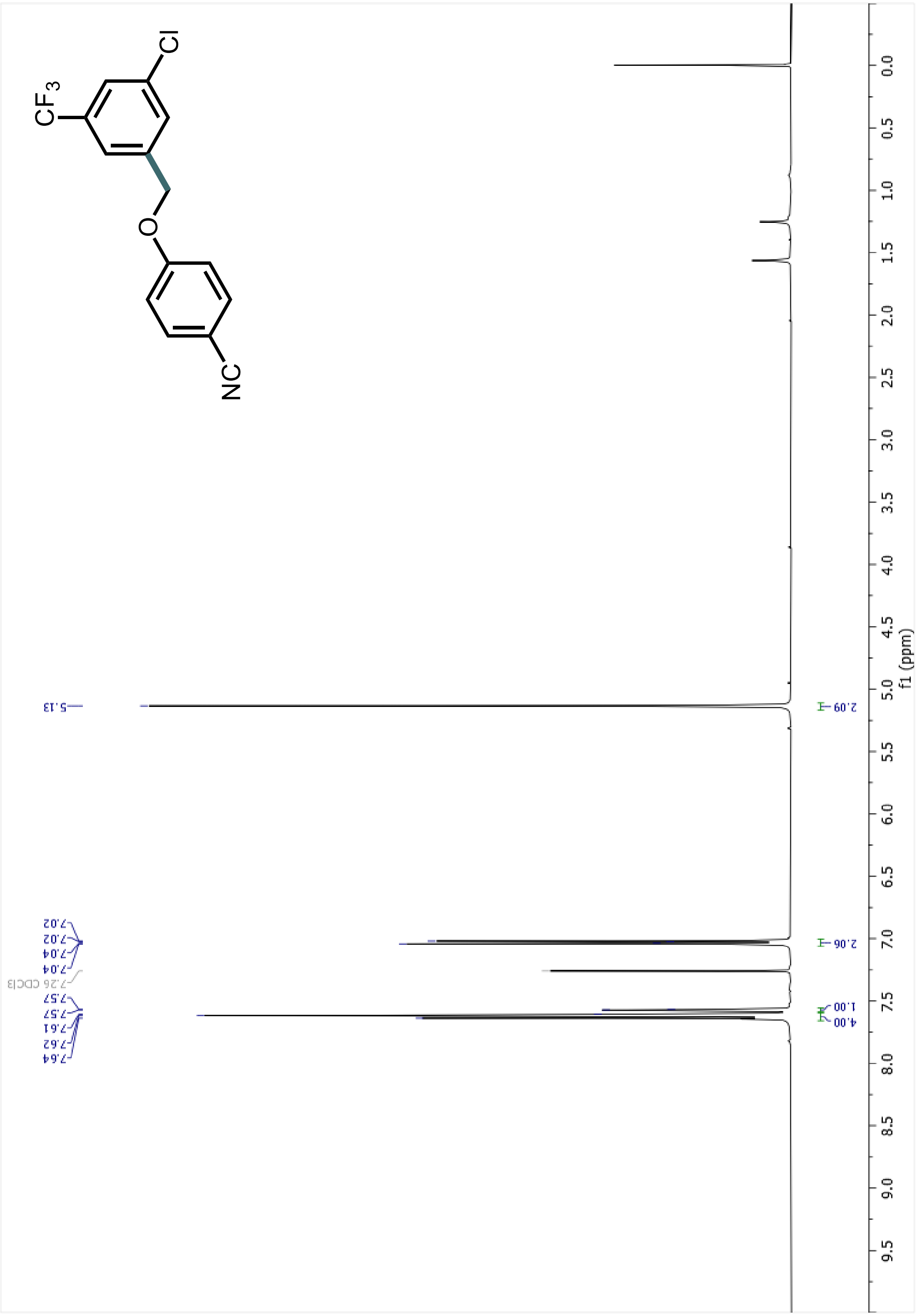

$^{13}C\{^{1}H\}$ NMR SPECTRUM OF **21A** (101 MHz, $CDCl_3$):

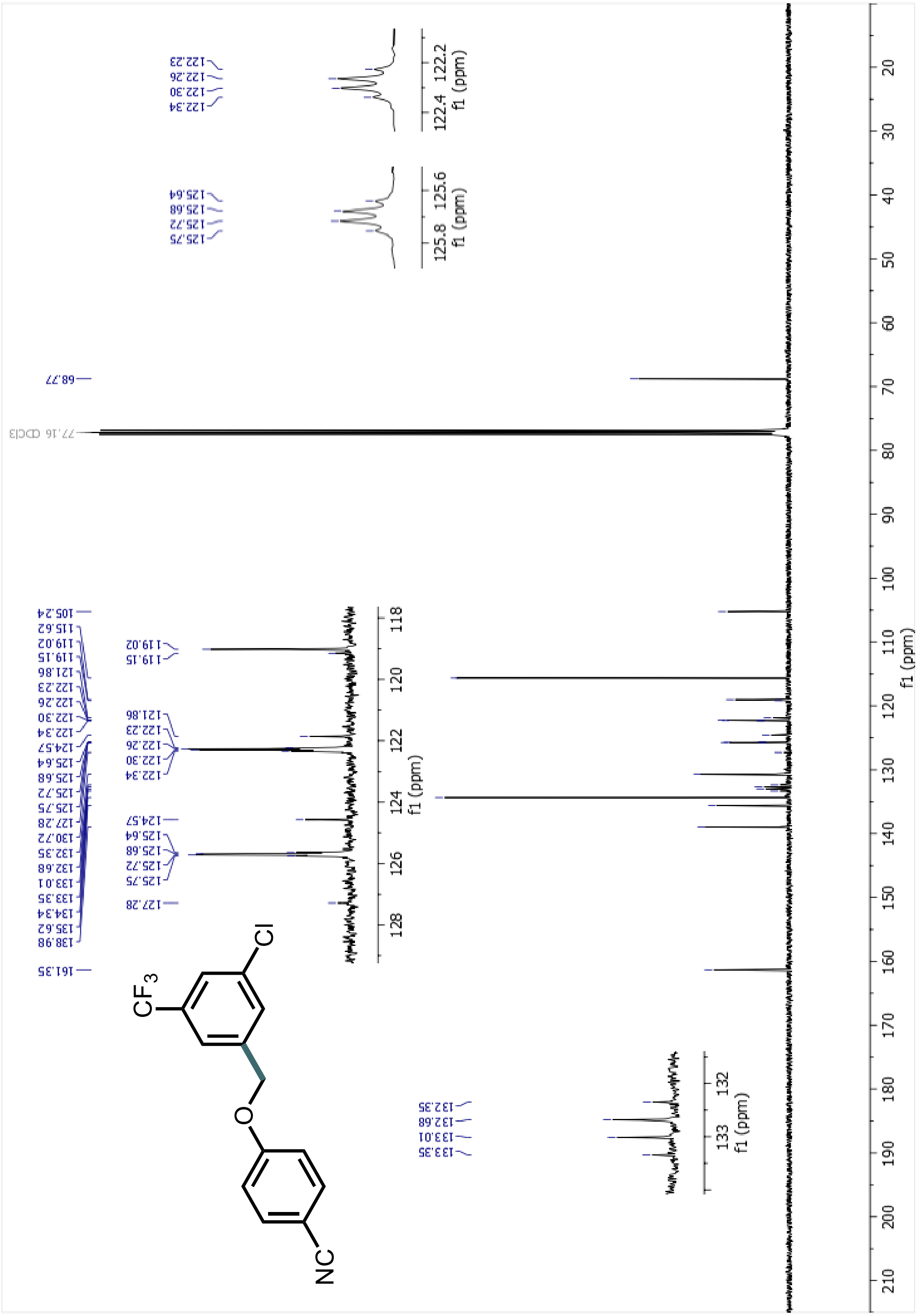

^{19}F NMR SPECTRUM OF **21A** (377 MHz, $CDCl_3$):

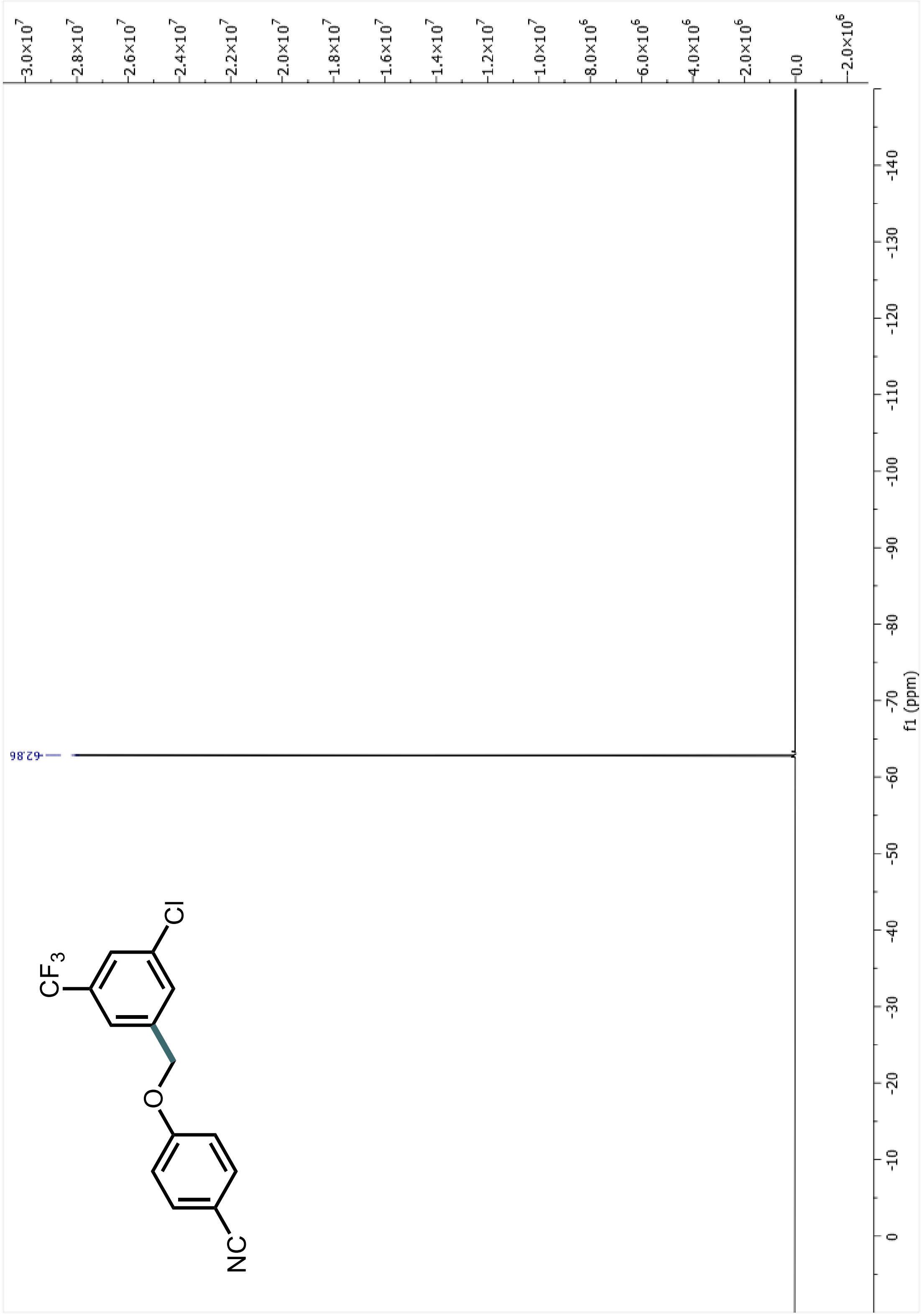

^{1}H NMR SPECTRUM OF **21B** (400 MHz, $CDCl_3$):

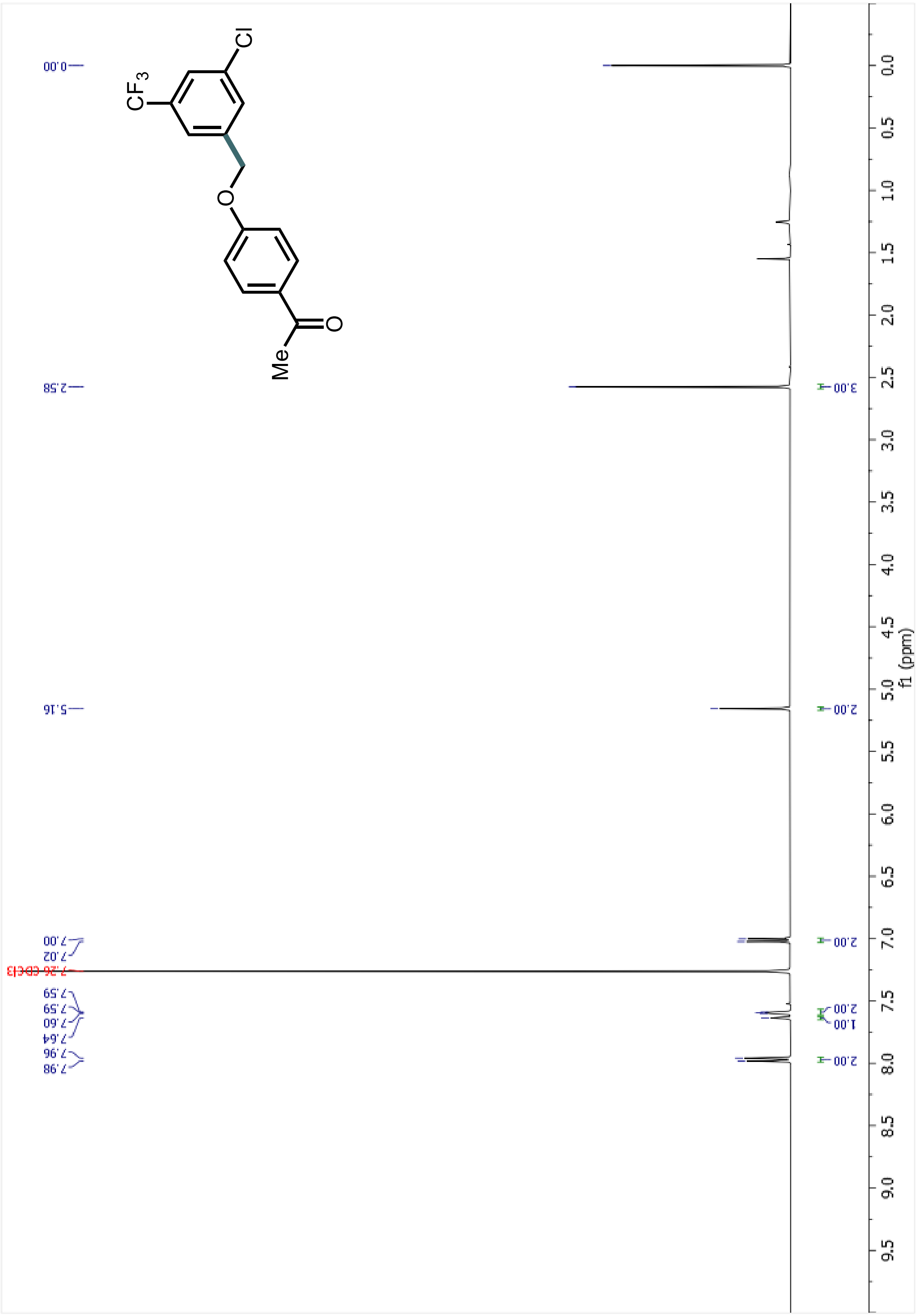

$^{13}C\{^1H\}$ NMR SPECTRUM OF **21B** (101 MHz, $CDCl_3$):

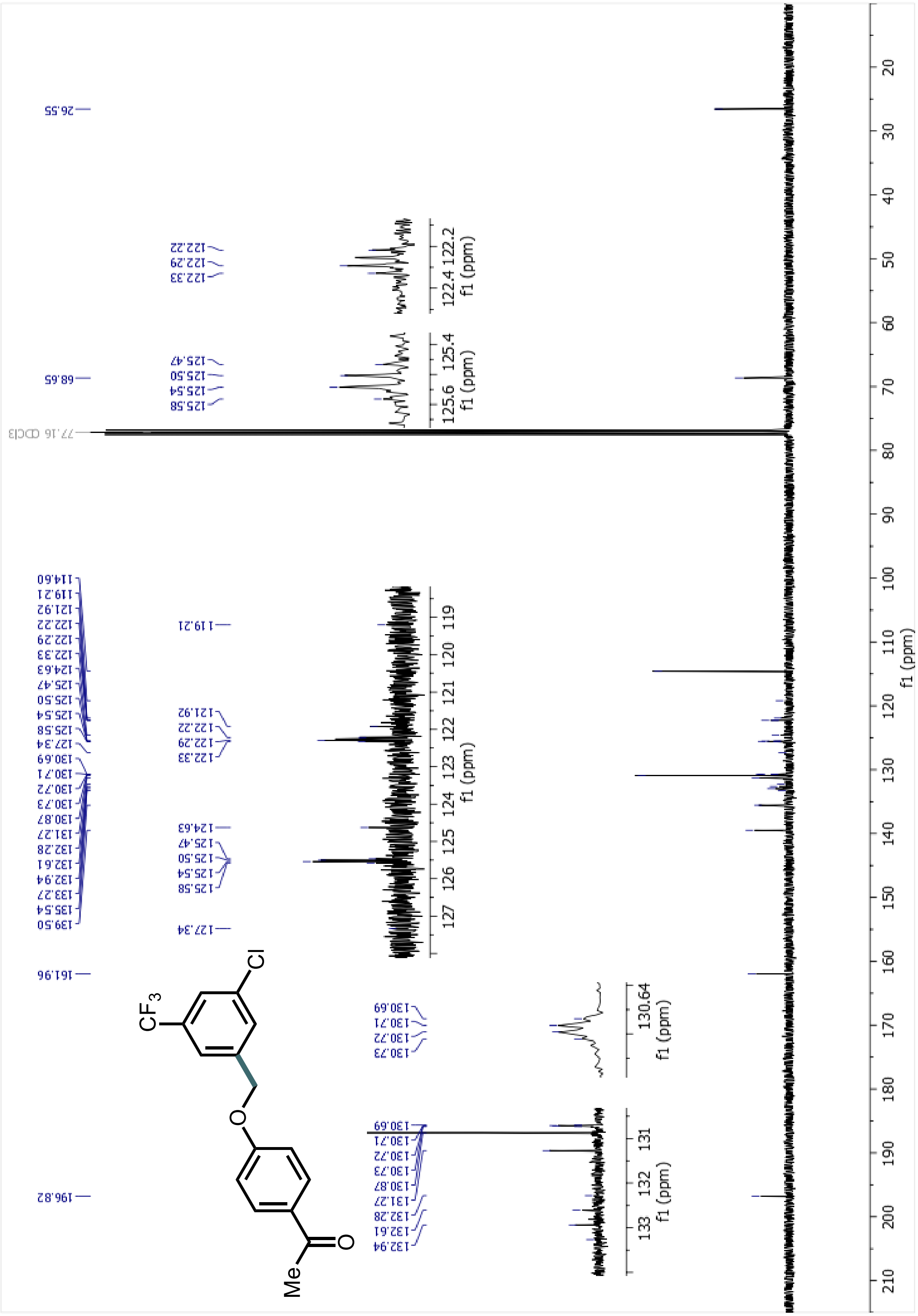

^{19}F NMR SPECTRUM OF **21B** (377 MHz, $CDCl_3$):

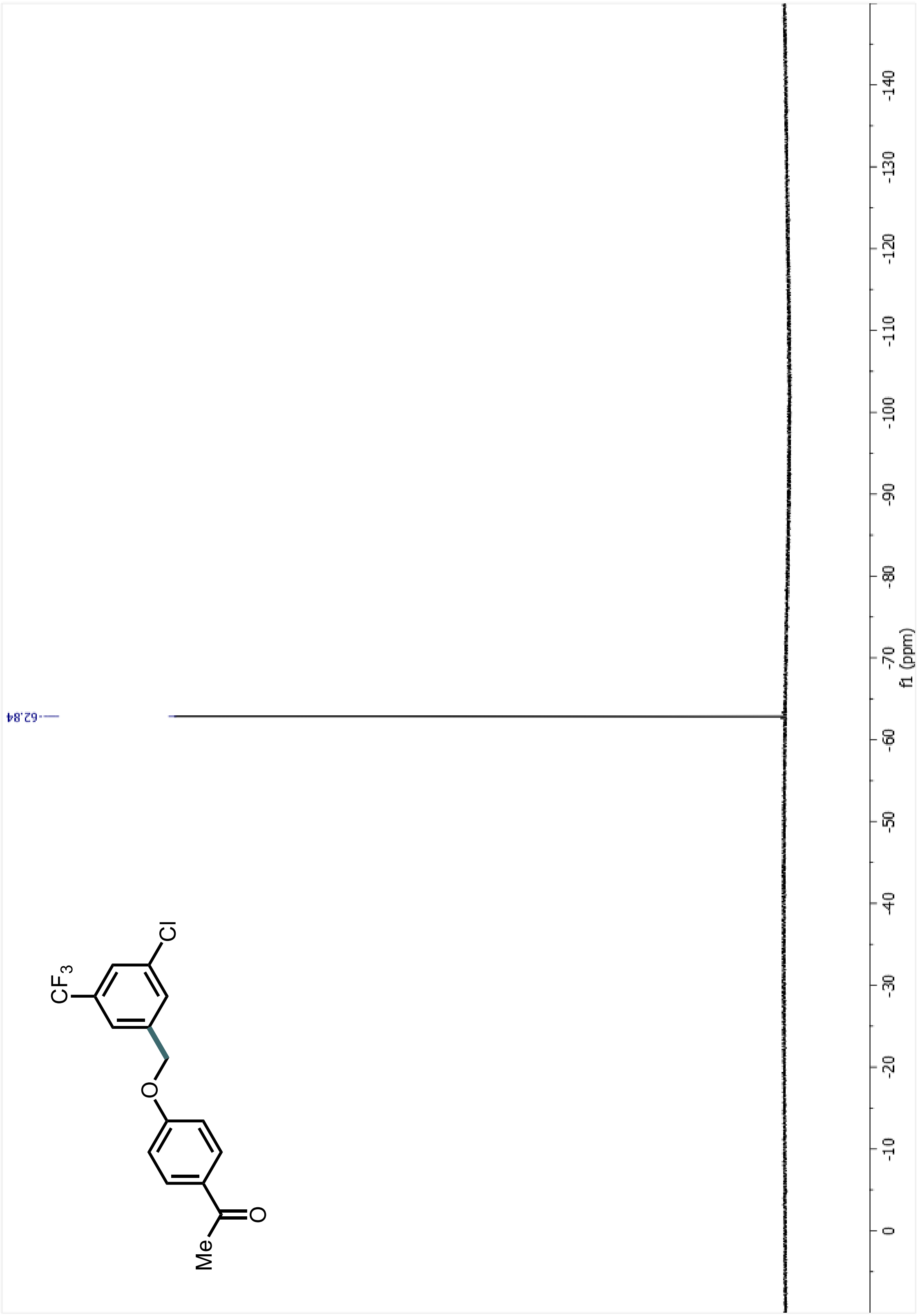

^{1}H NMR SPECTRUM OF **21C** (400 MHz, $CDCl_3$):

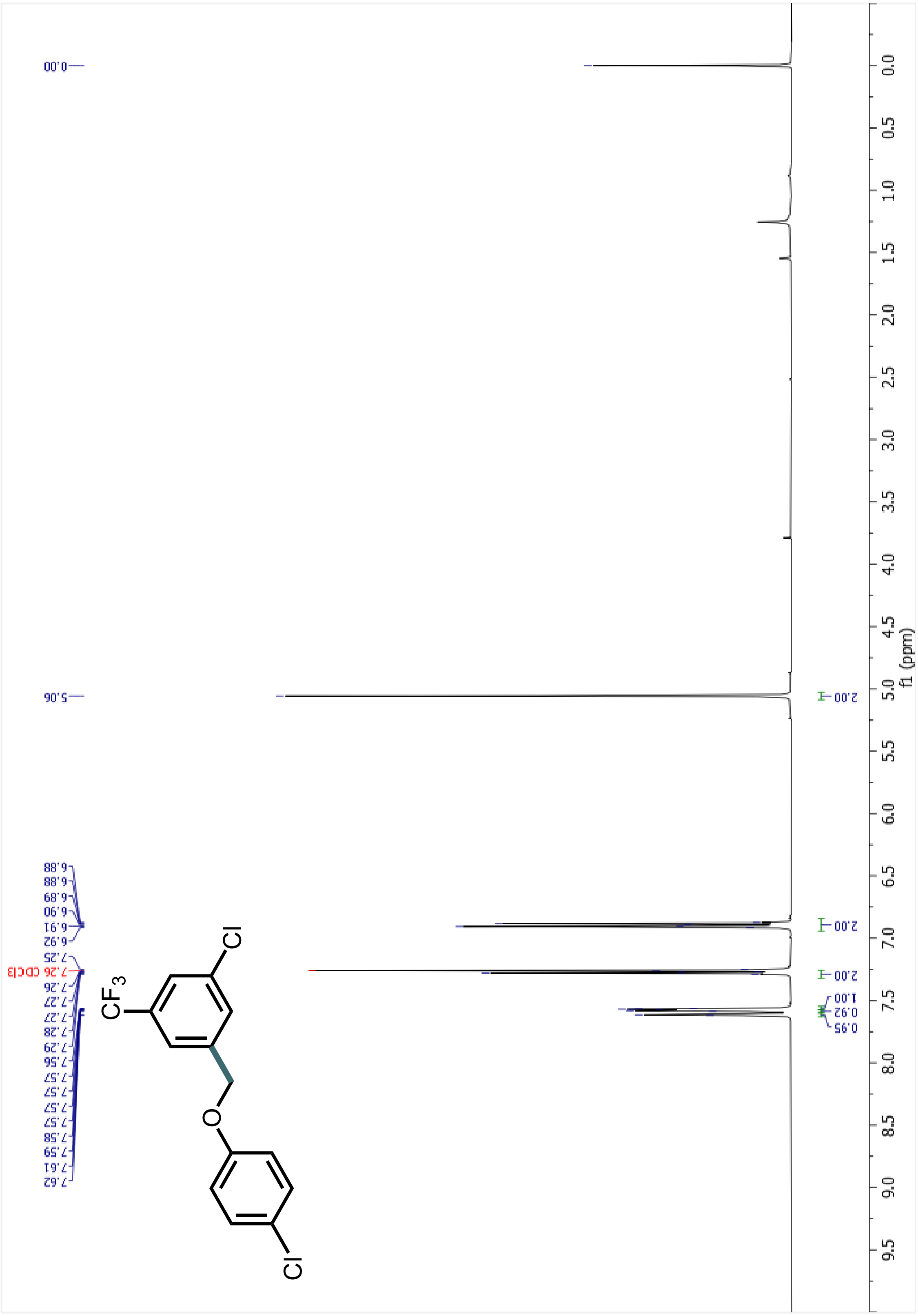

$^{13}C\{^1H\}$ NMR SPECTRUM OF **21C** (101 MHz, $CDCl_3$):

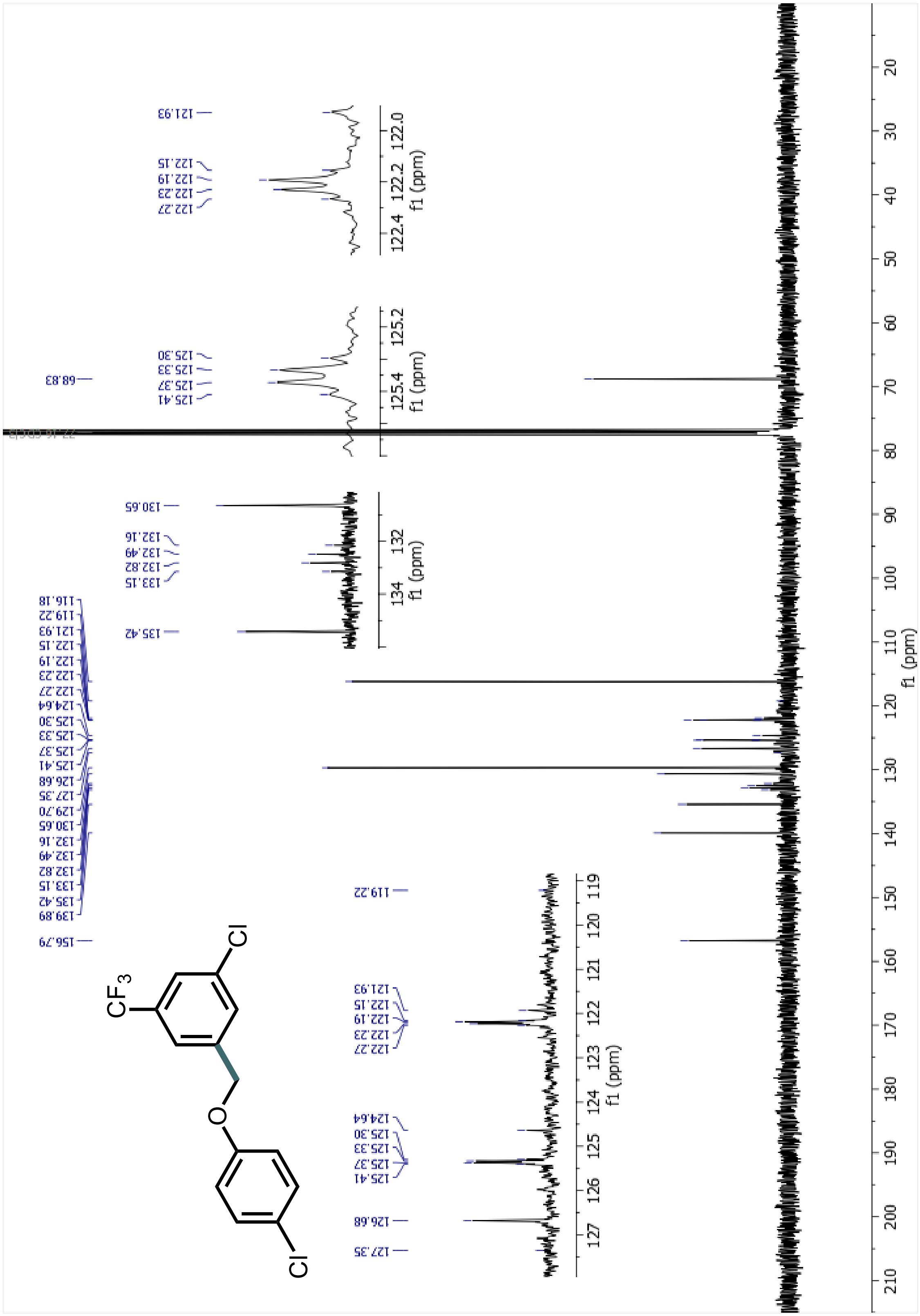

^{19}F NMR SPECTRUM OF **21C** (377 MHz, $CDCl_3$):

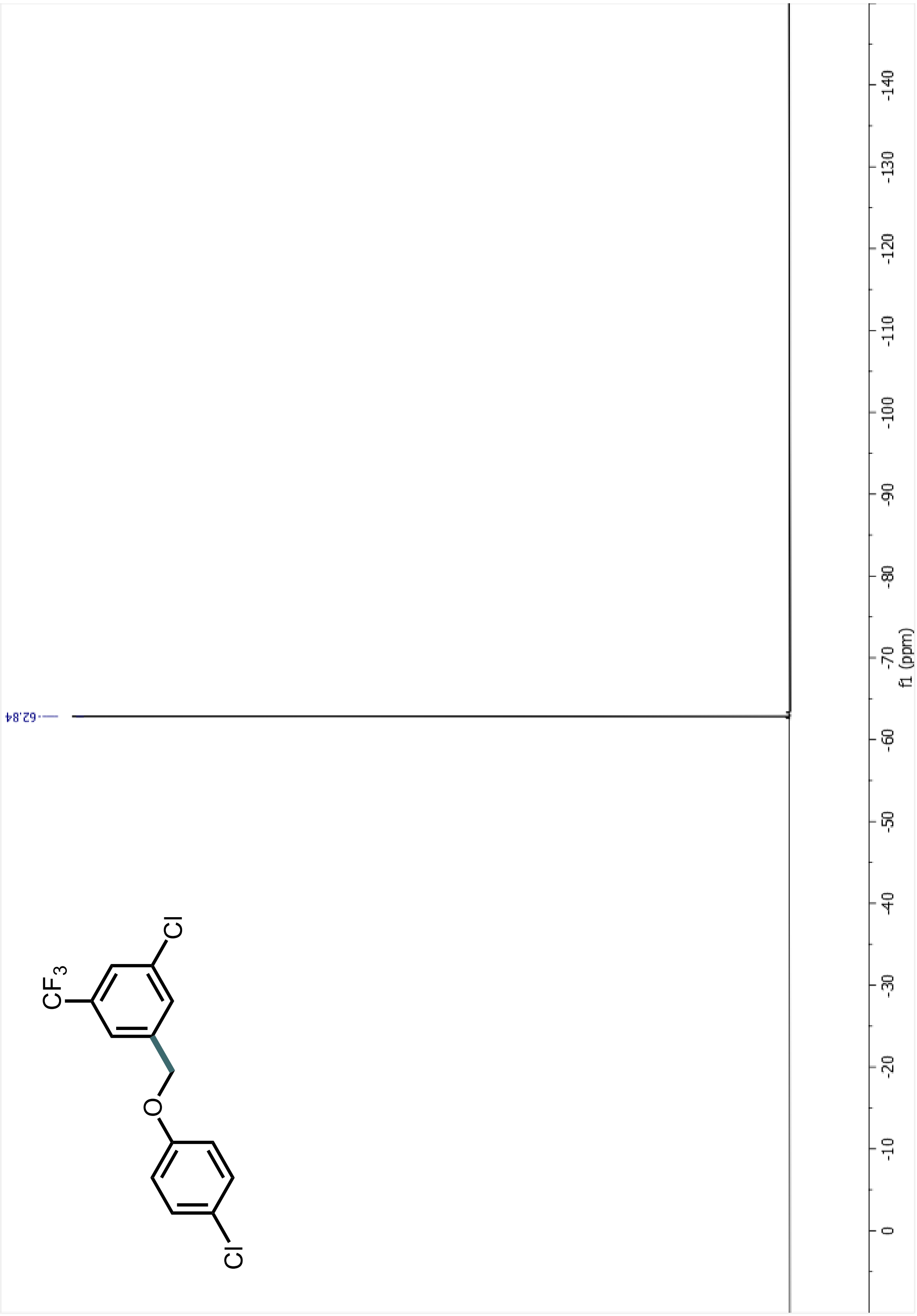

^{1}H NMR SPECTRUM OF **22** (400 MHz, $CDCl_3$):

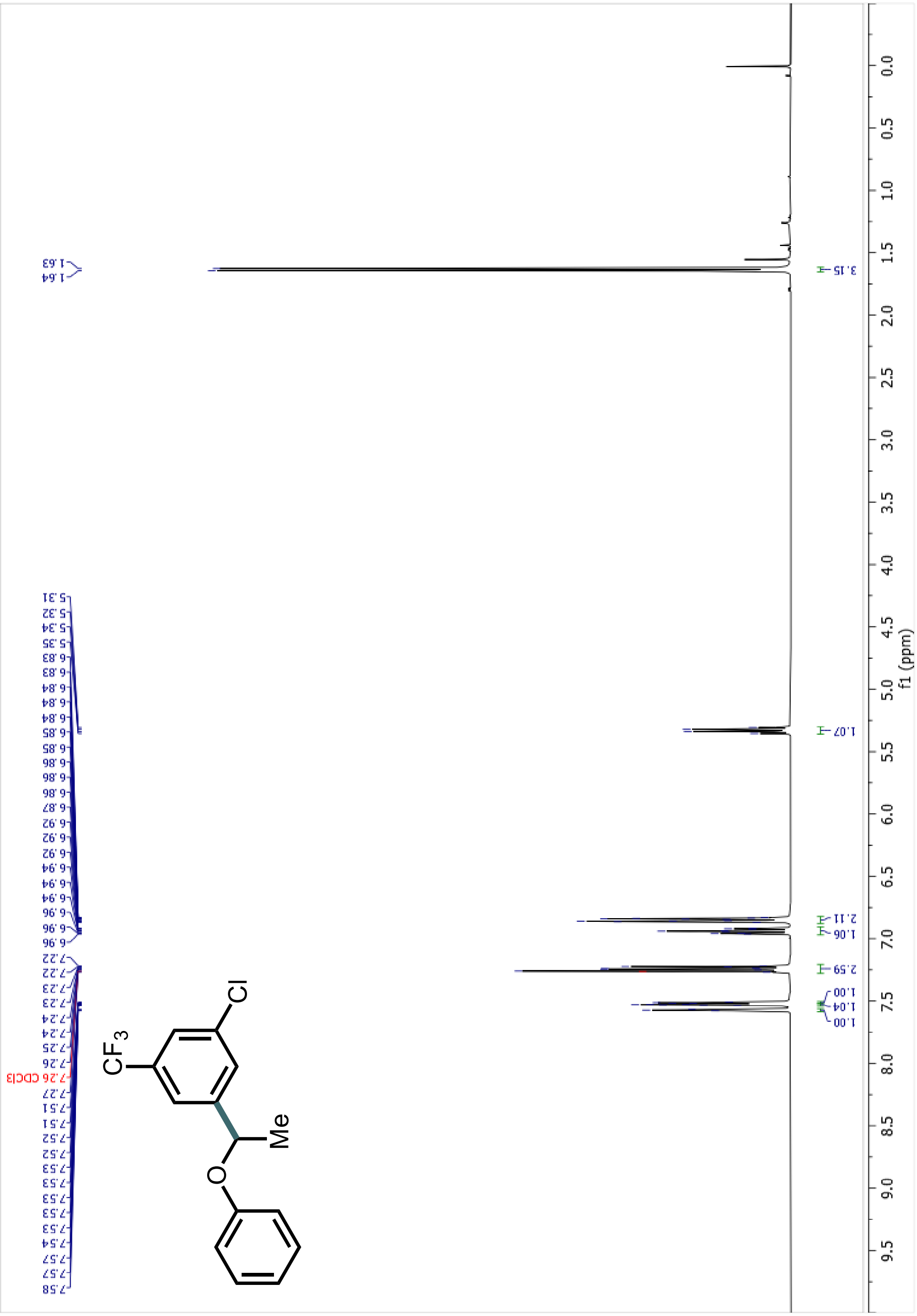

$^{13}C\{^{1}H\}$ NMR SPECTRUM OF **22** (101 MHz, $CDCl_3$):

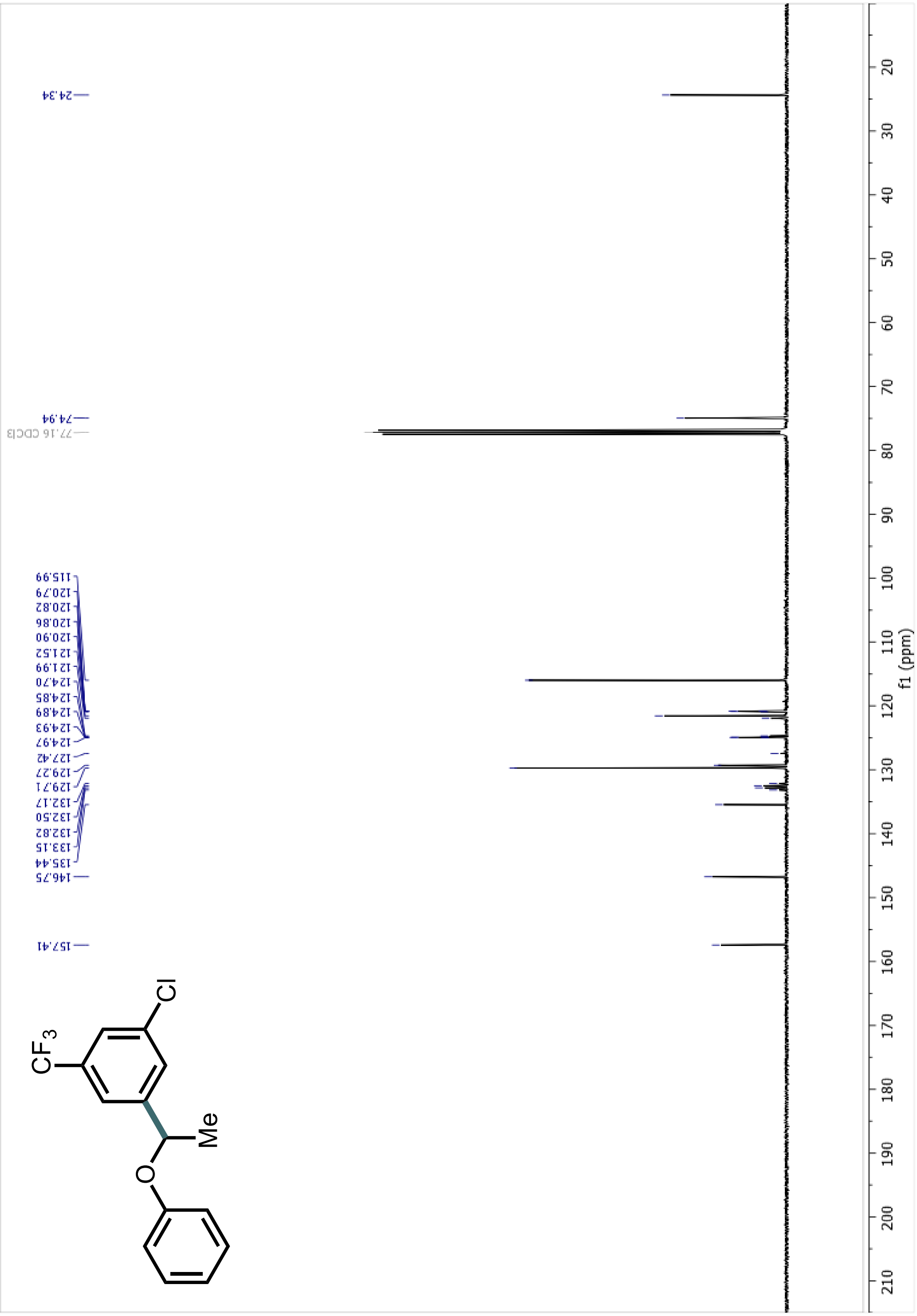

^{19}F NMR SPECTRUM OF **22** (377 MHz, $CDCl_3$):

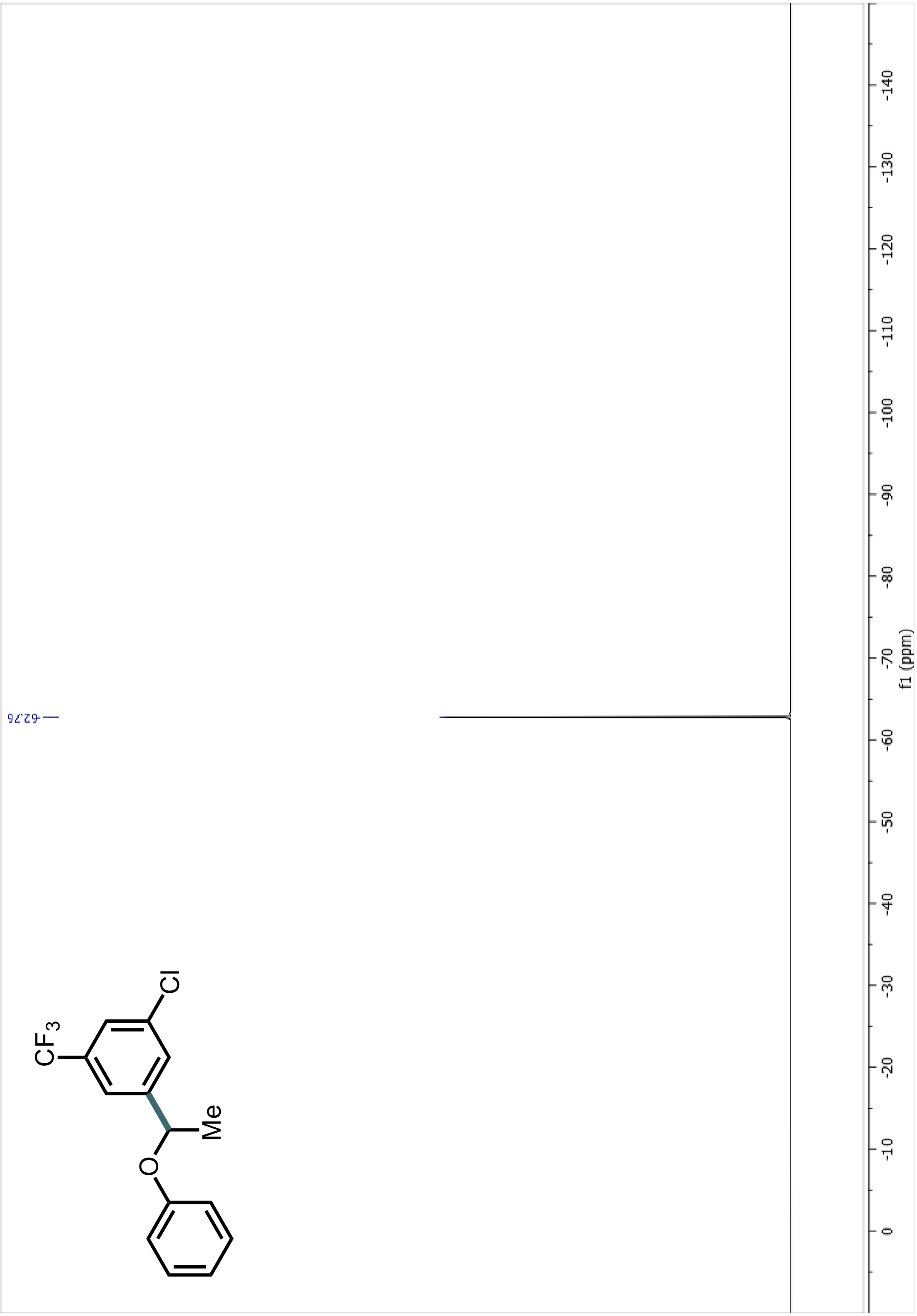

^{1}H NMR SPECTRUM OF **23** (400 MHz, $CDCl_3$):

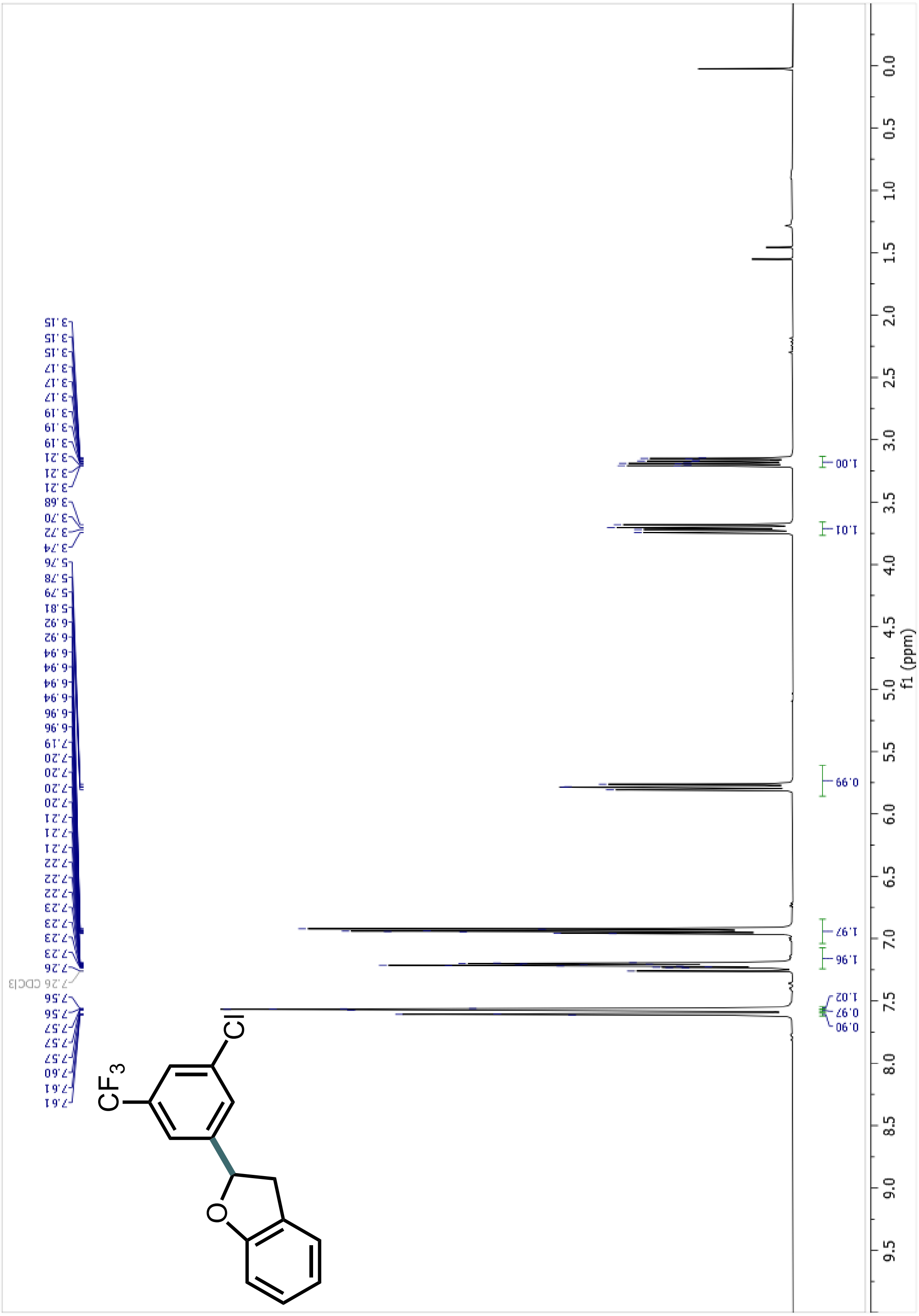

$^{13}C\{^1H\}$ NMR SPECTRUM OF **23** (101 MHz, $CDCl_3$):

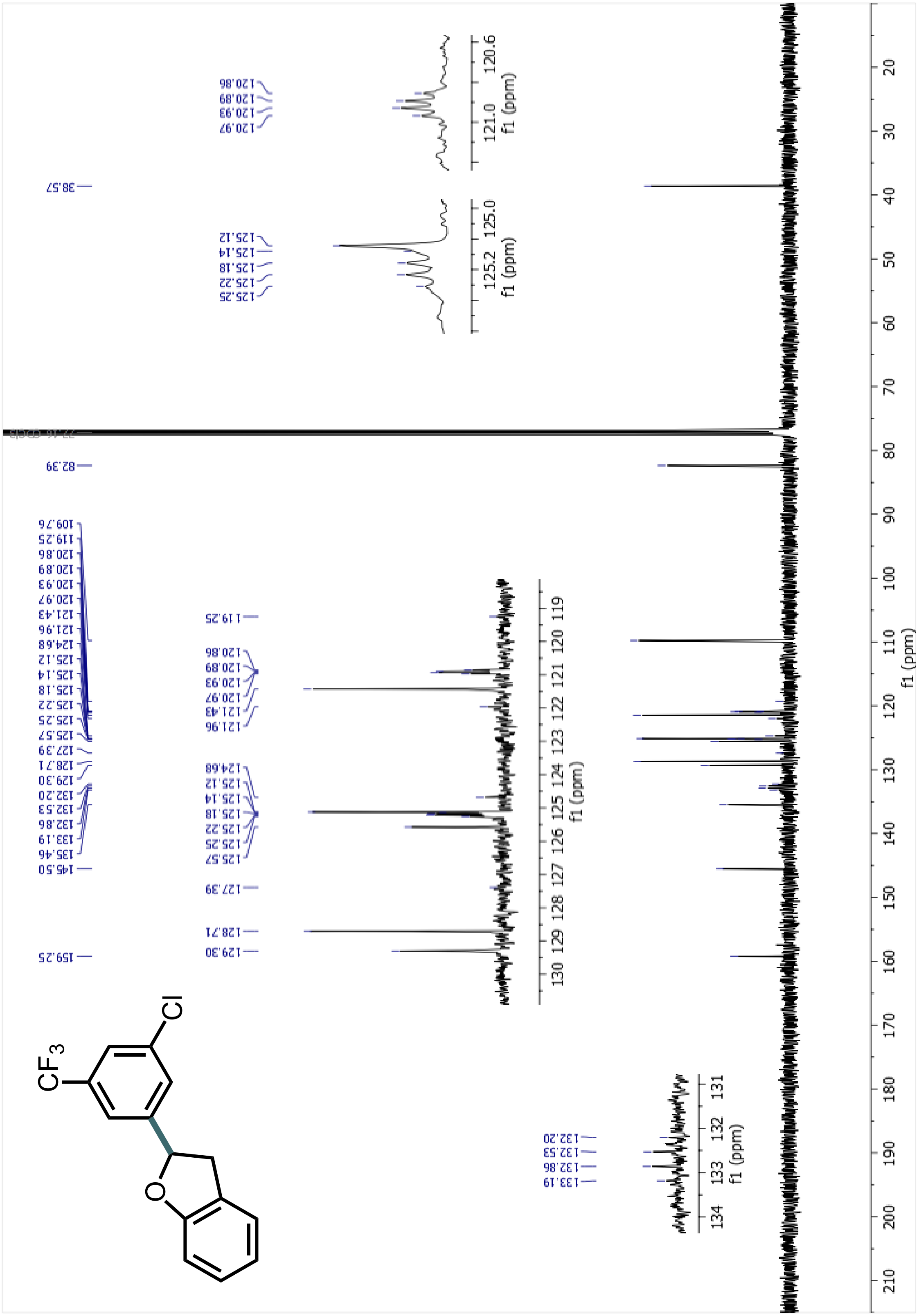

^{19}F NMR SPECTRUM OF **23** (377 MHz, $CDCl_3$):

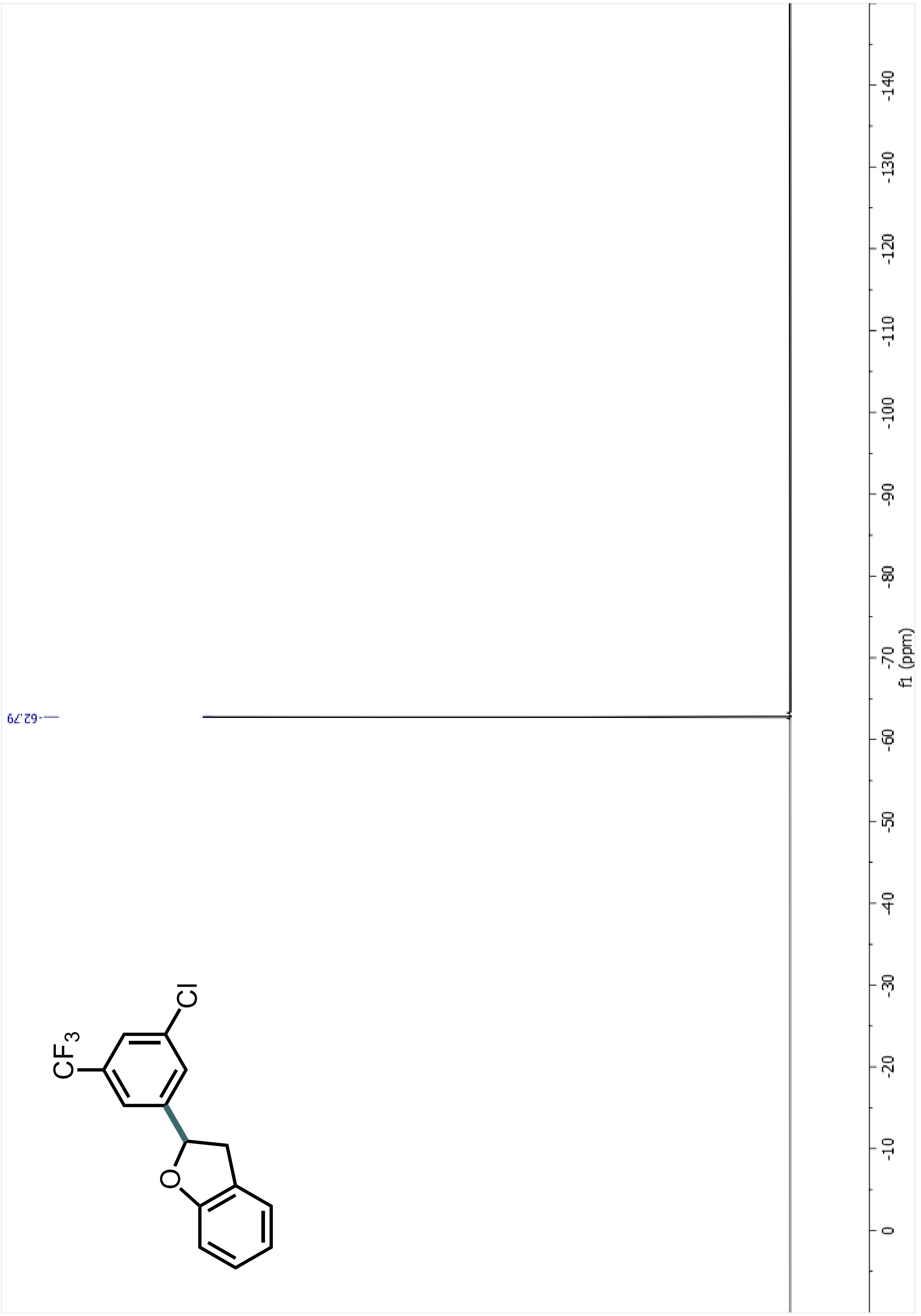

^{1}H NMR SPECTRUM OF **24** (400 MHz, $CDCl_3$):

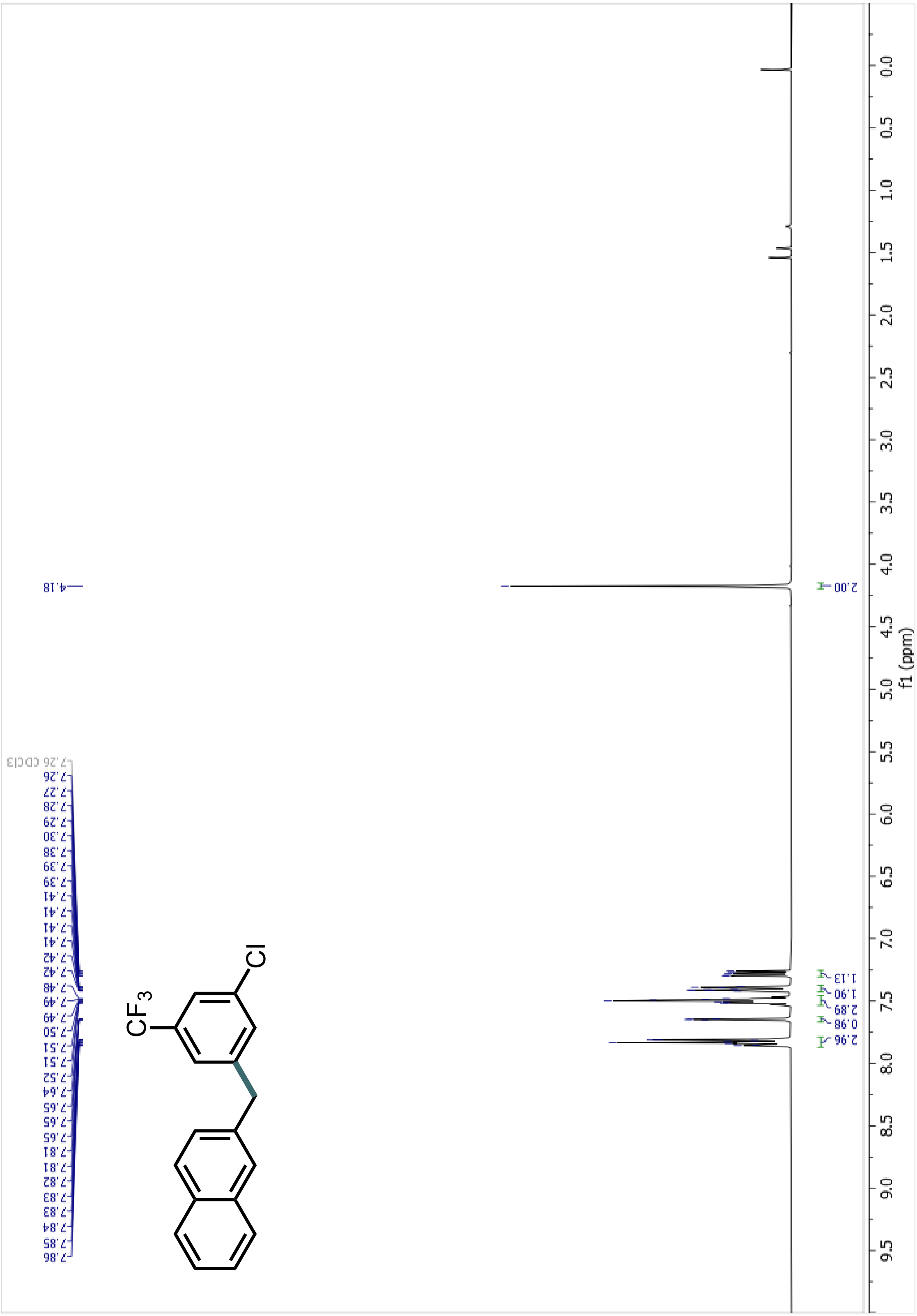

$^{13}C\{^{1}H\}$ NMR SPECTRUM OF **24** (101 MHz, $CDCl_3$):

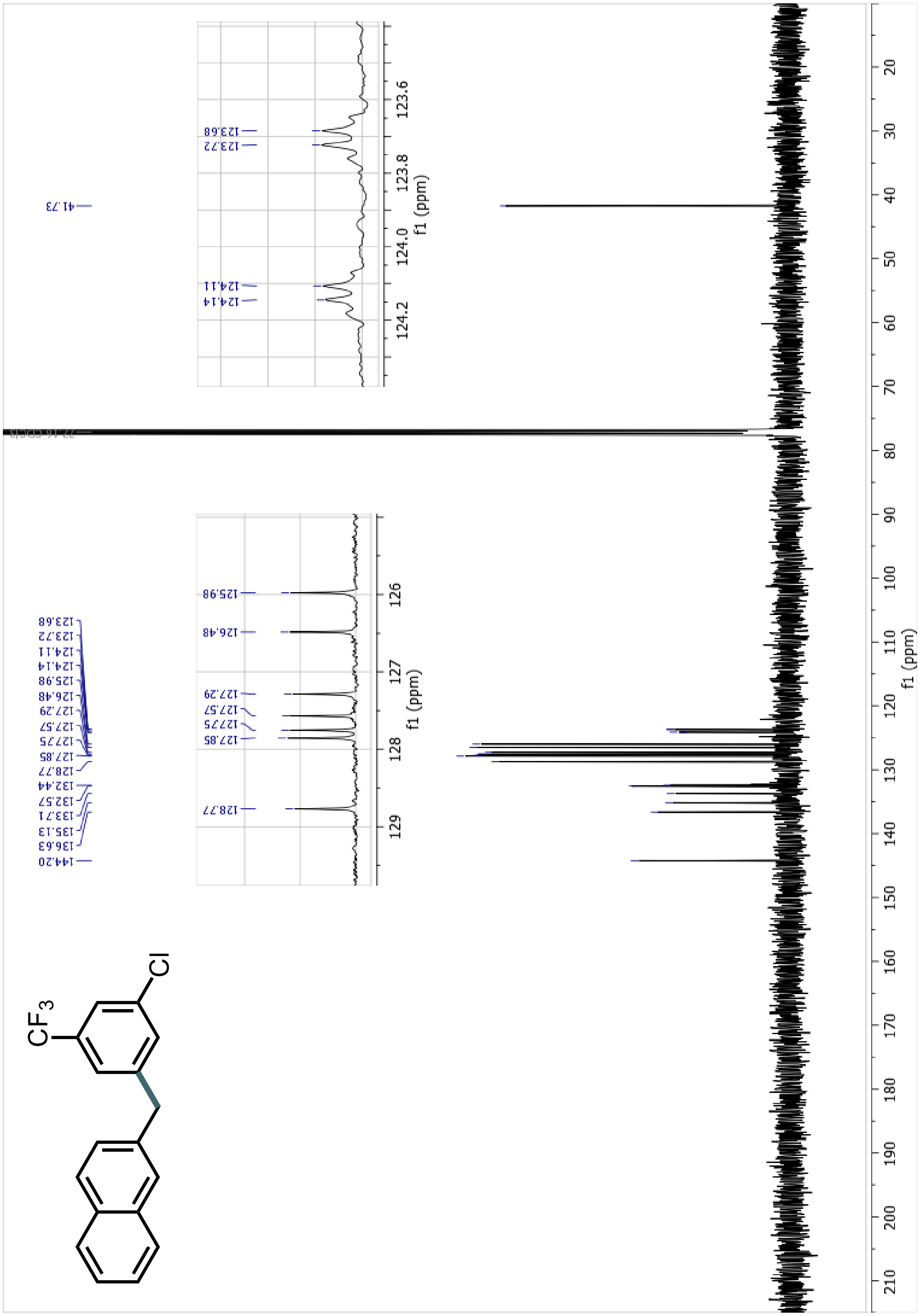

^{19}F NMR SPECTRUM OF **24** (377 MHz, $CDCl_3$):

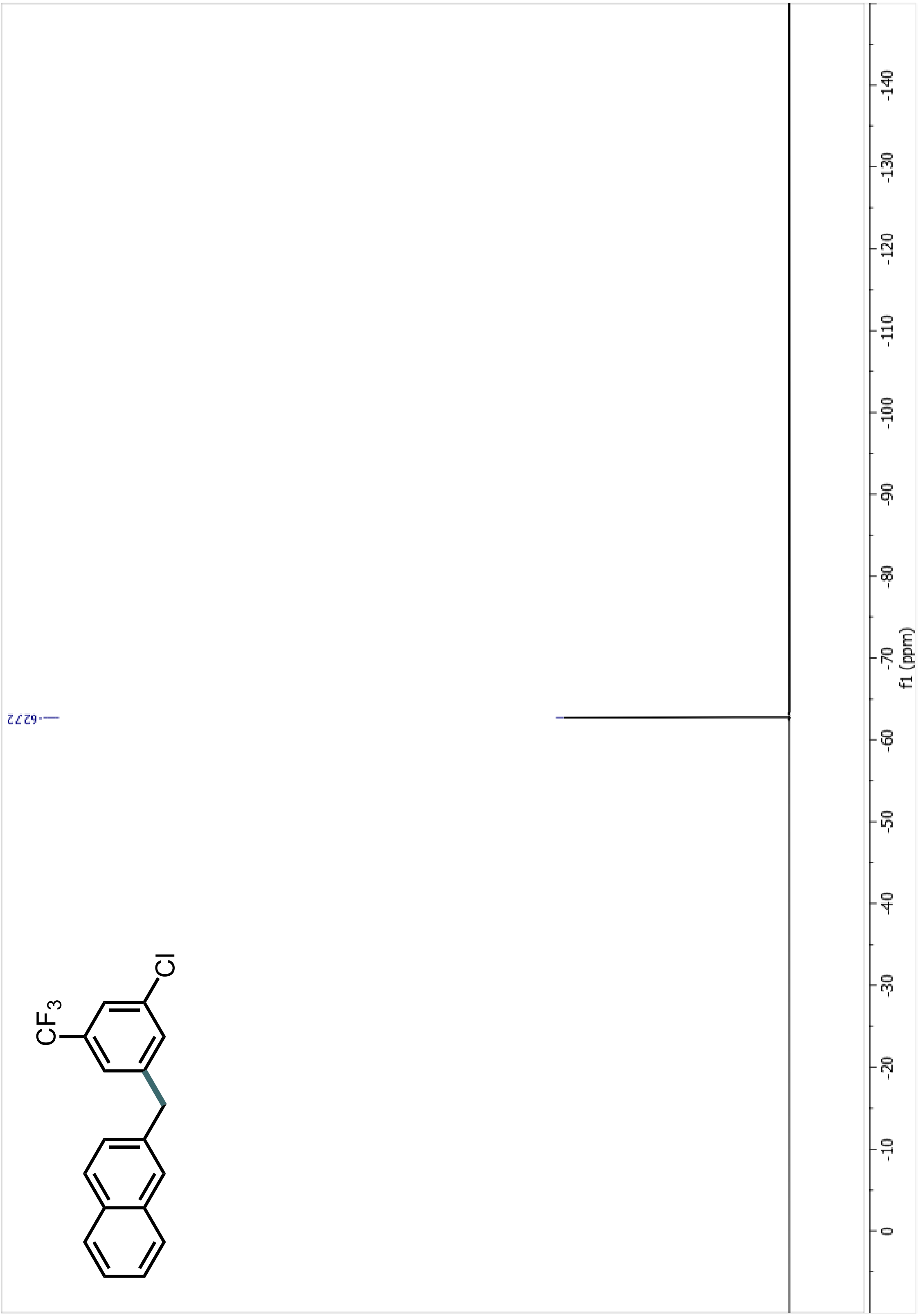

^{1}H NMR SPECTRUM OF **25** (400 MHz, $CDCl_3$):

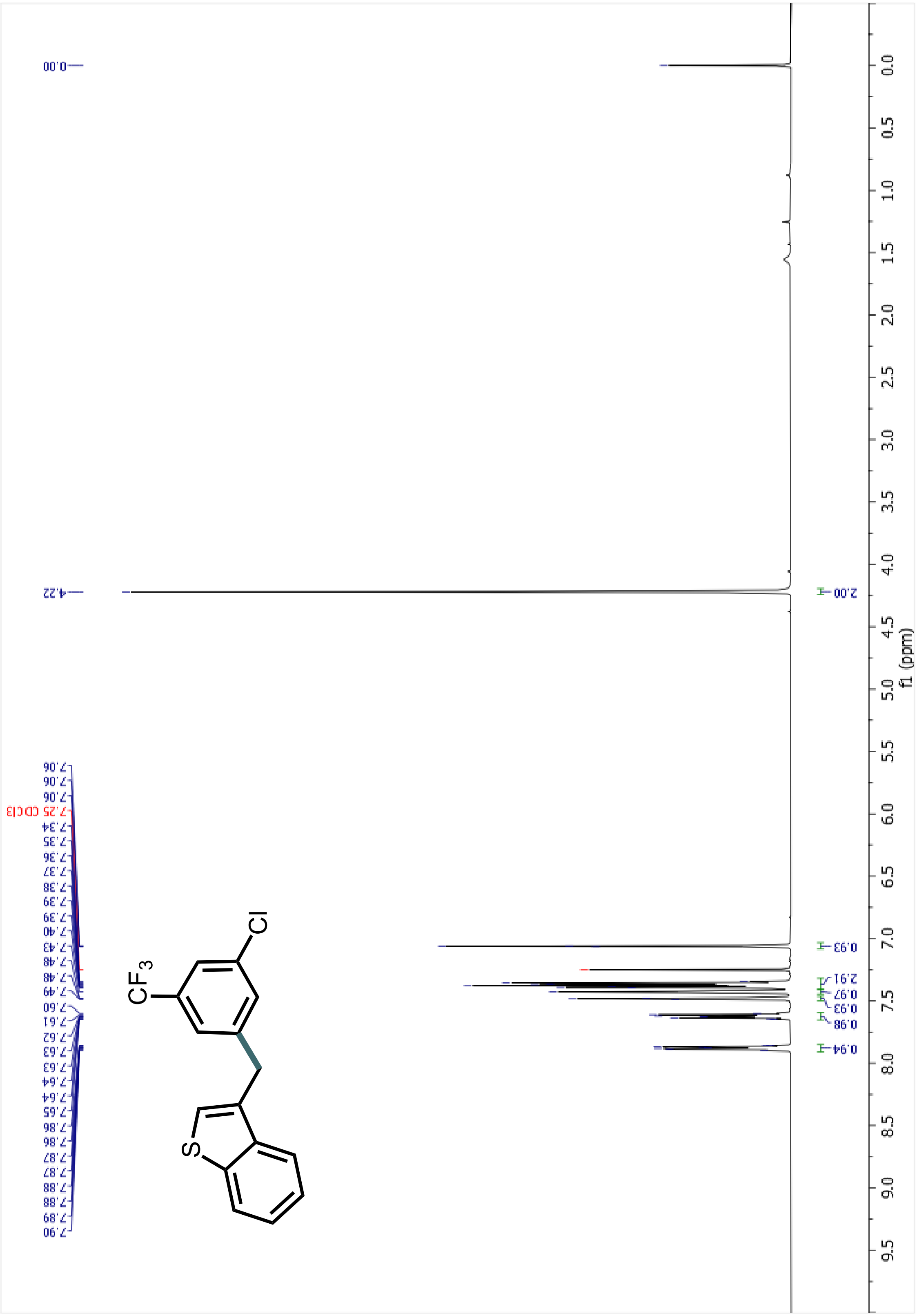

$^{13}C\{^{1}H\}$ NMR SPECTRUM OF **25** (101 MHz, $CDCl_3$):

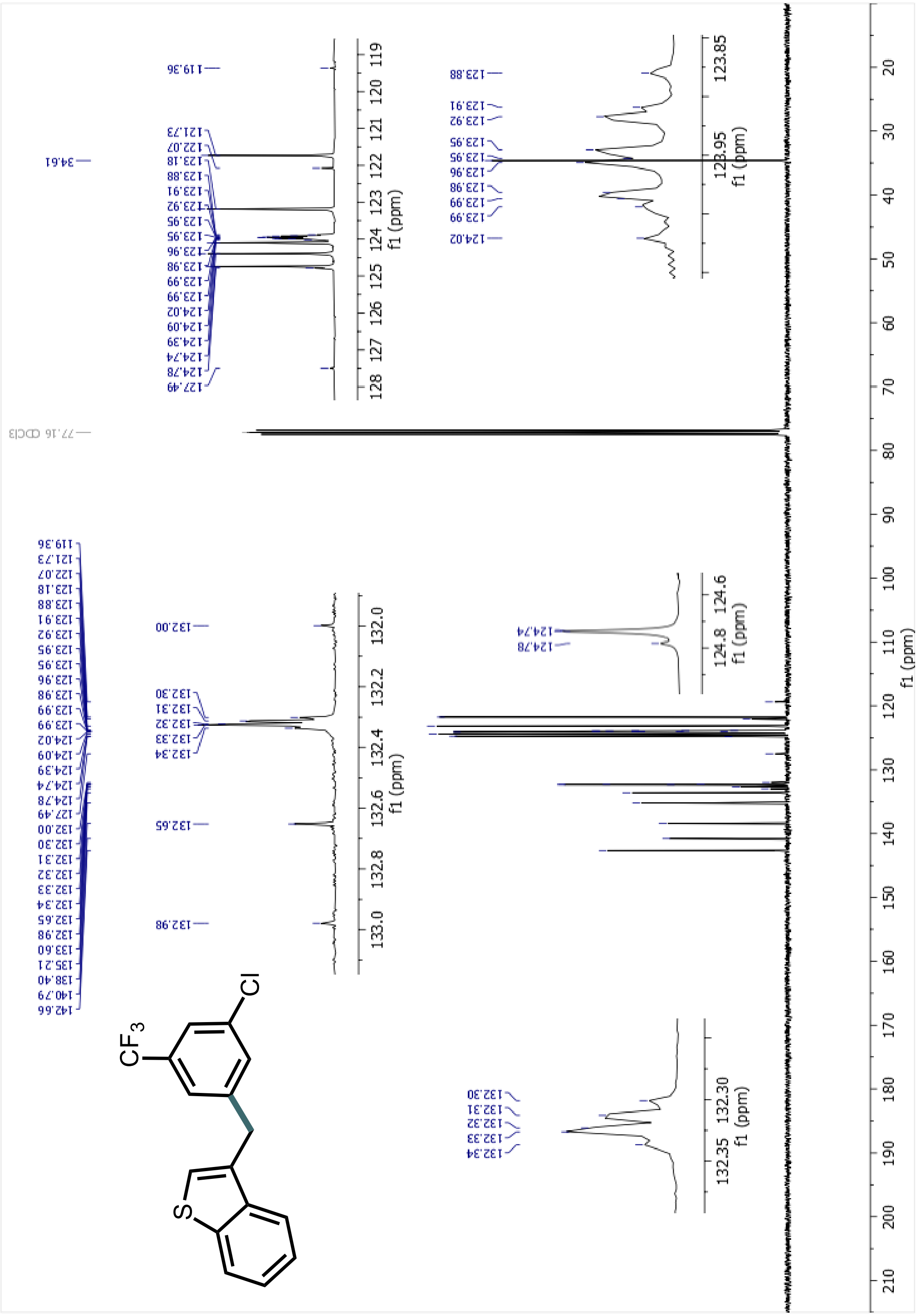

^{19}F NMR SPECTRUM OF **25** (377 MHz, $CDCl_3$):

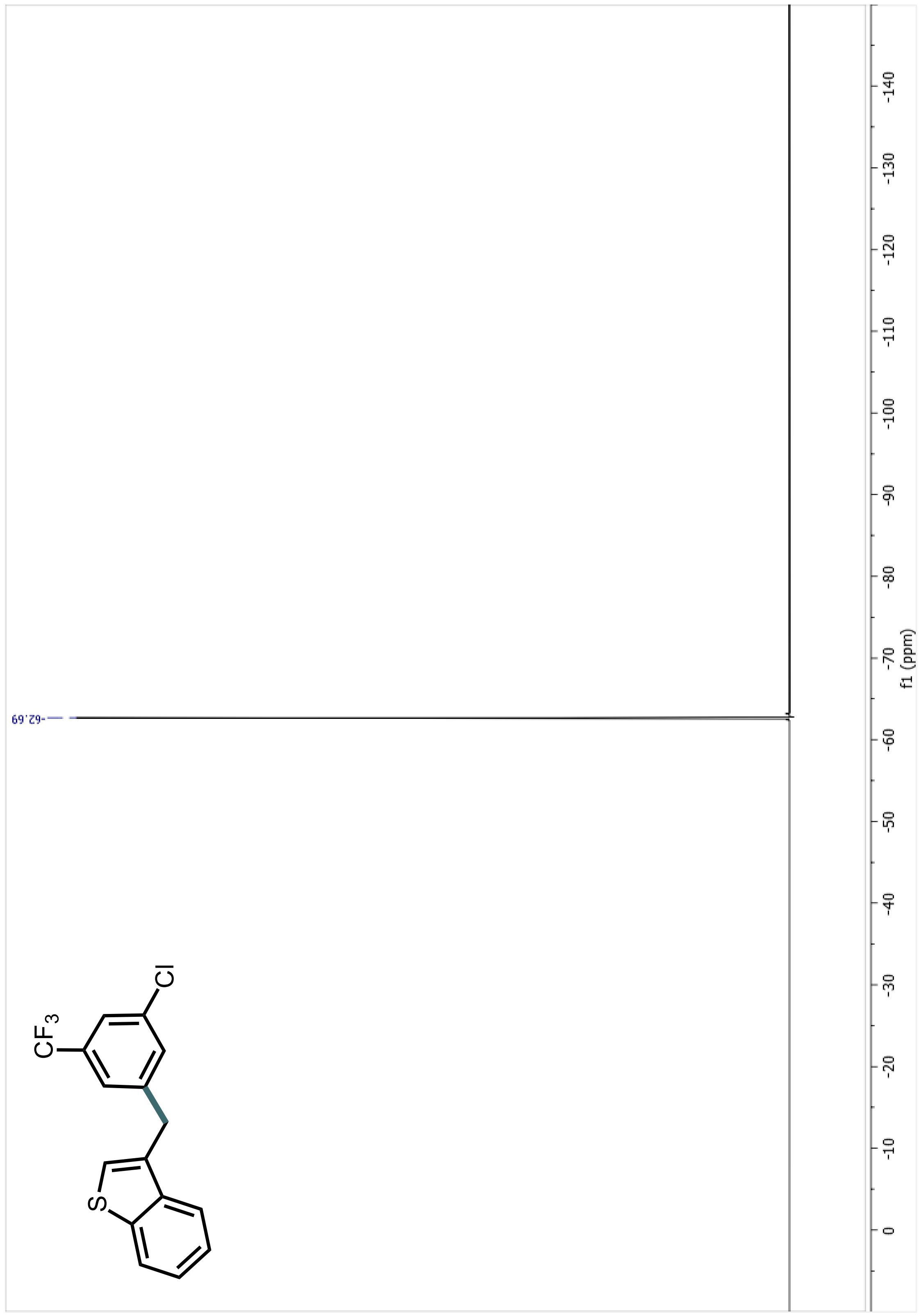

^{1}H NMR SPECTRUM OF **26** (400 MHz, $CDCl_3$):

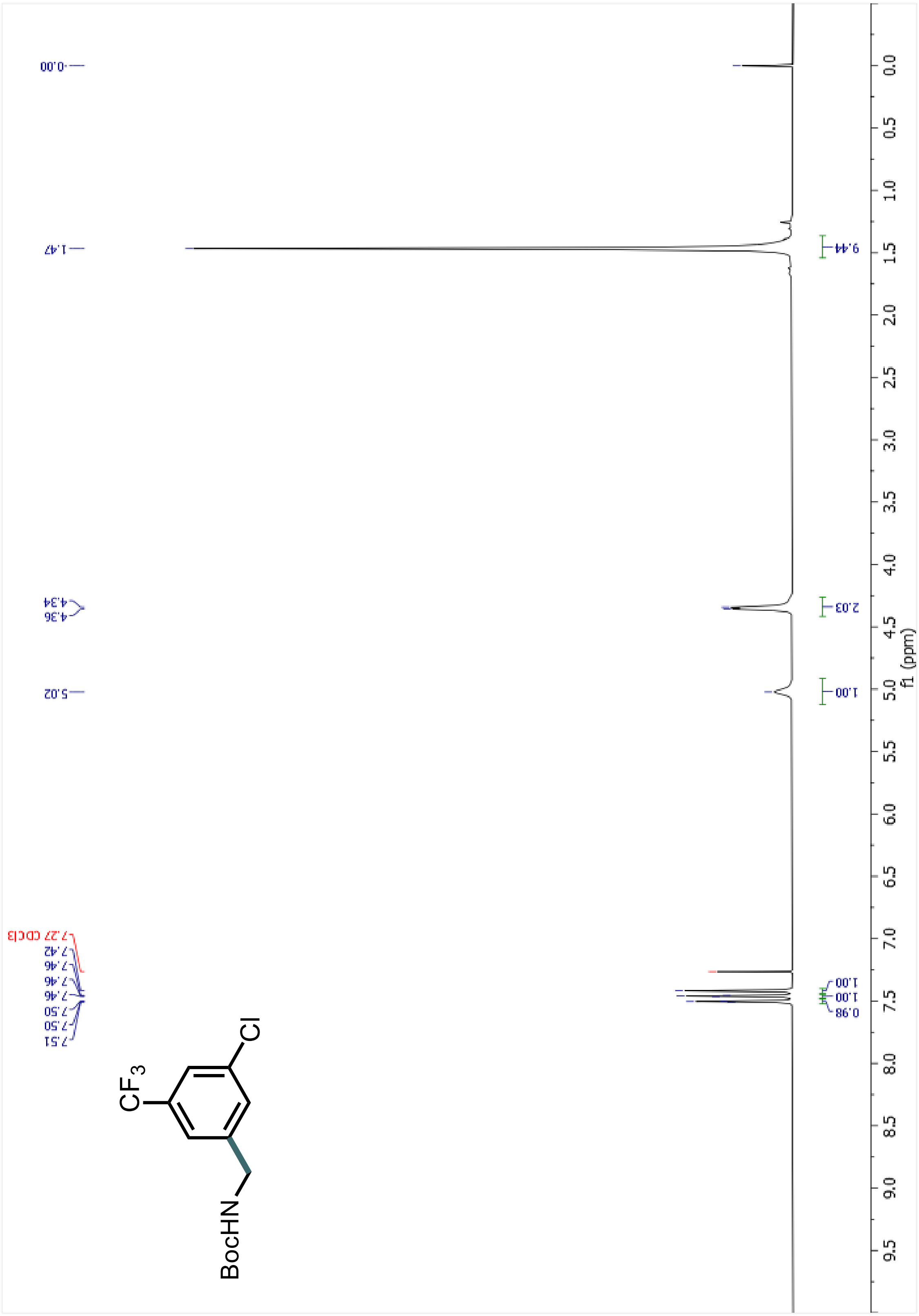

$^{13}C\{^{1}H\}$ NMR SPECTRUM OF **26** (101 MHz, $CDCl_3$):

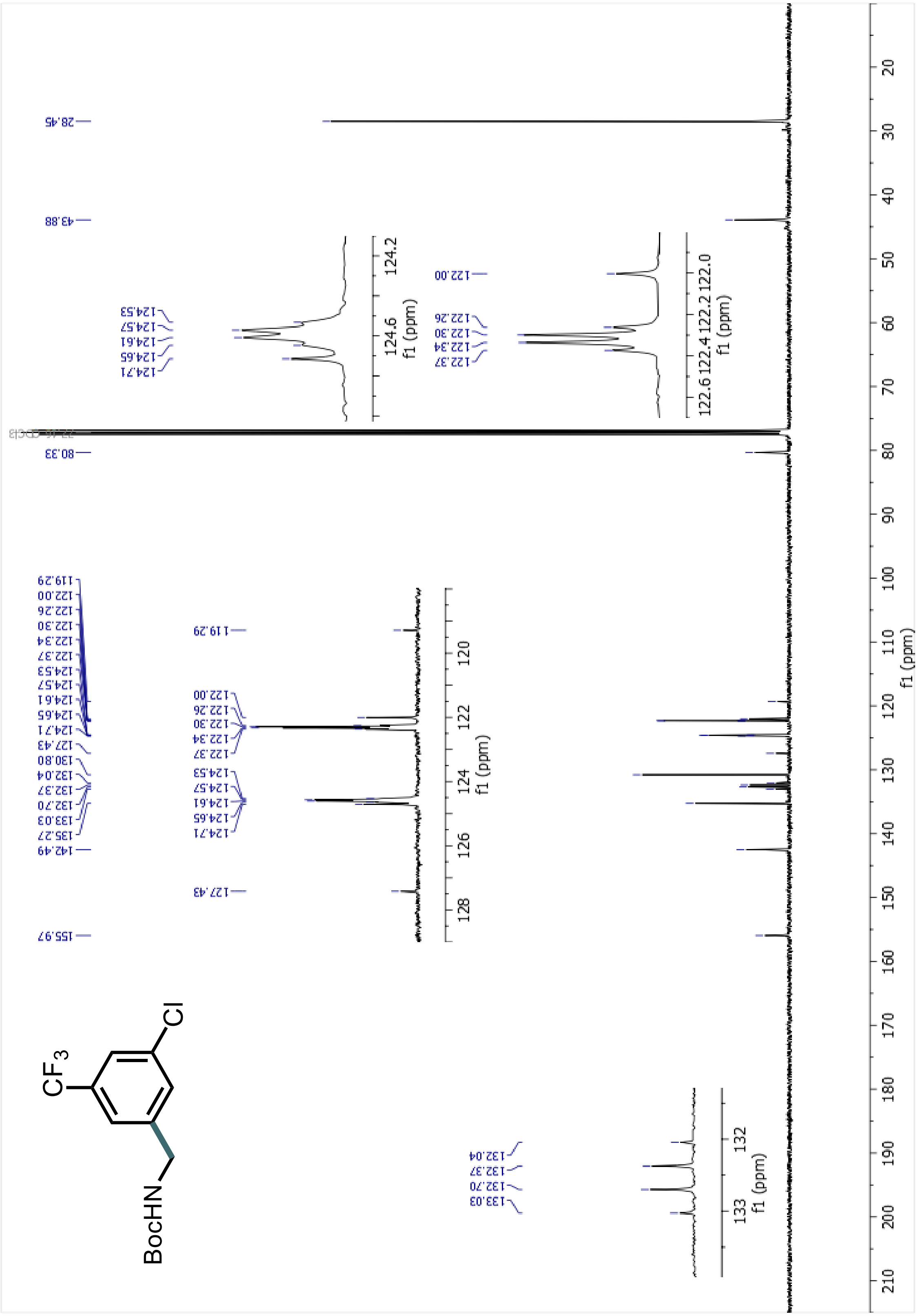

^{19}F NMR SPECTRUM OF **26** (377 MHz, $CDCl_3$):

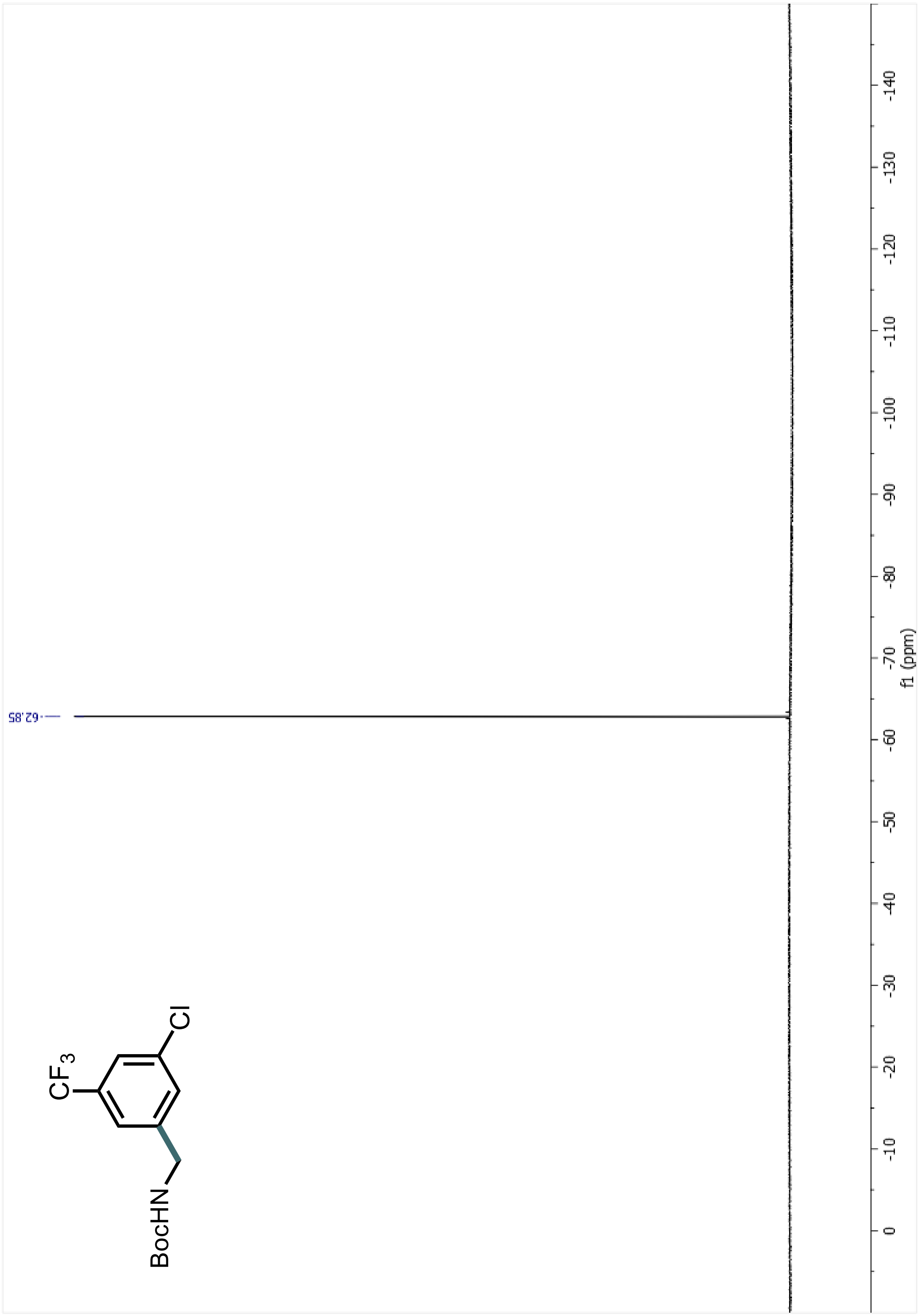

^{1}H NMR SPECTRUM OF **27** (400 MHz, $CDCl_3$):

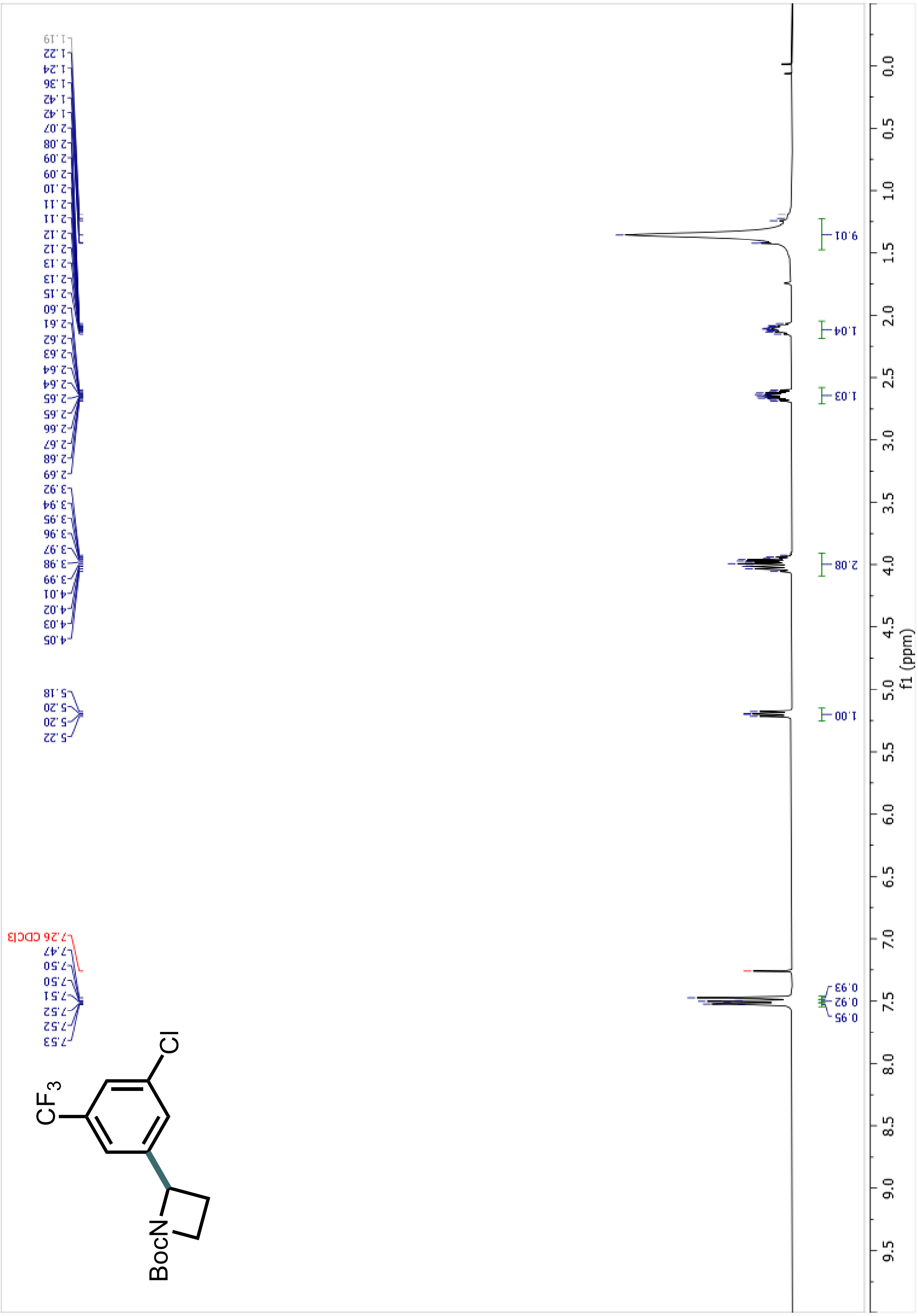

$^{13}C\{^{1}H\}$ NMR SPECTRUM OF **27** (101 MHz, $CDCl_3$):

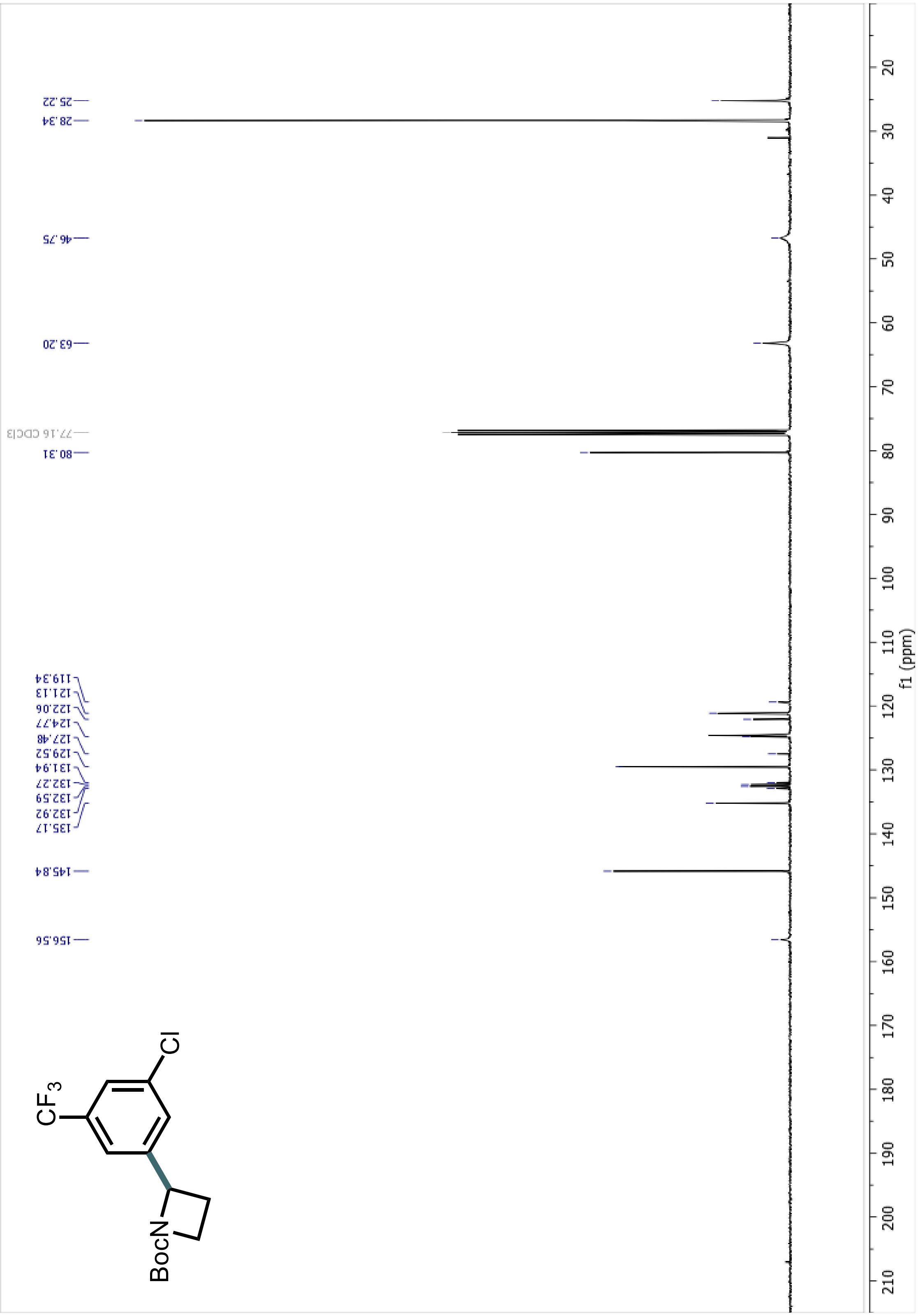

^{19}F NMR SPECTRUM OF **27** (377 MHz, $CDCl_3$):

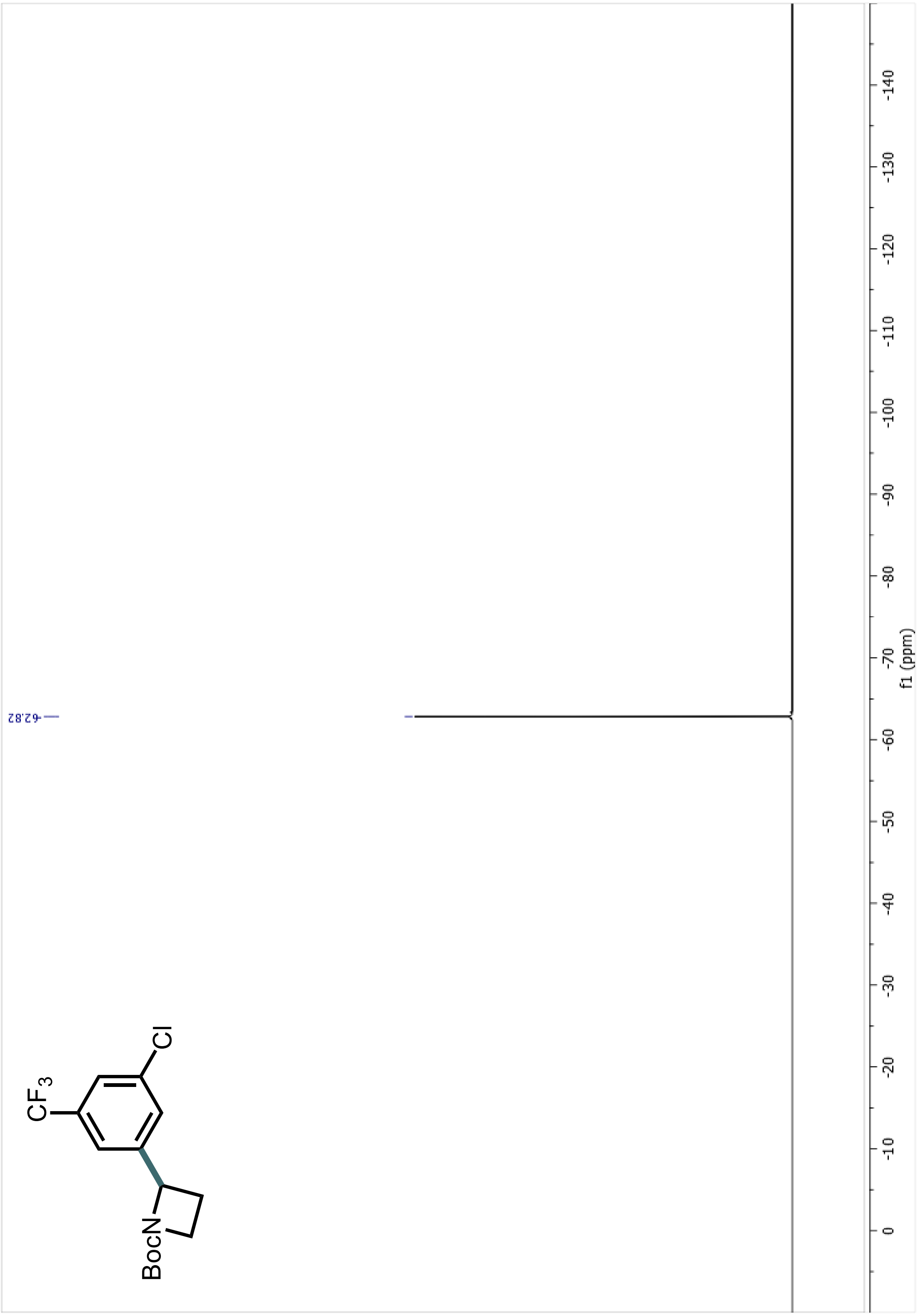

^{1}H NMR SPECTRUM OF **28** (400 MHz, $CDCl_3$):

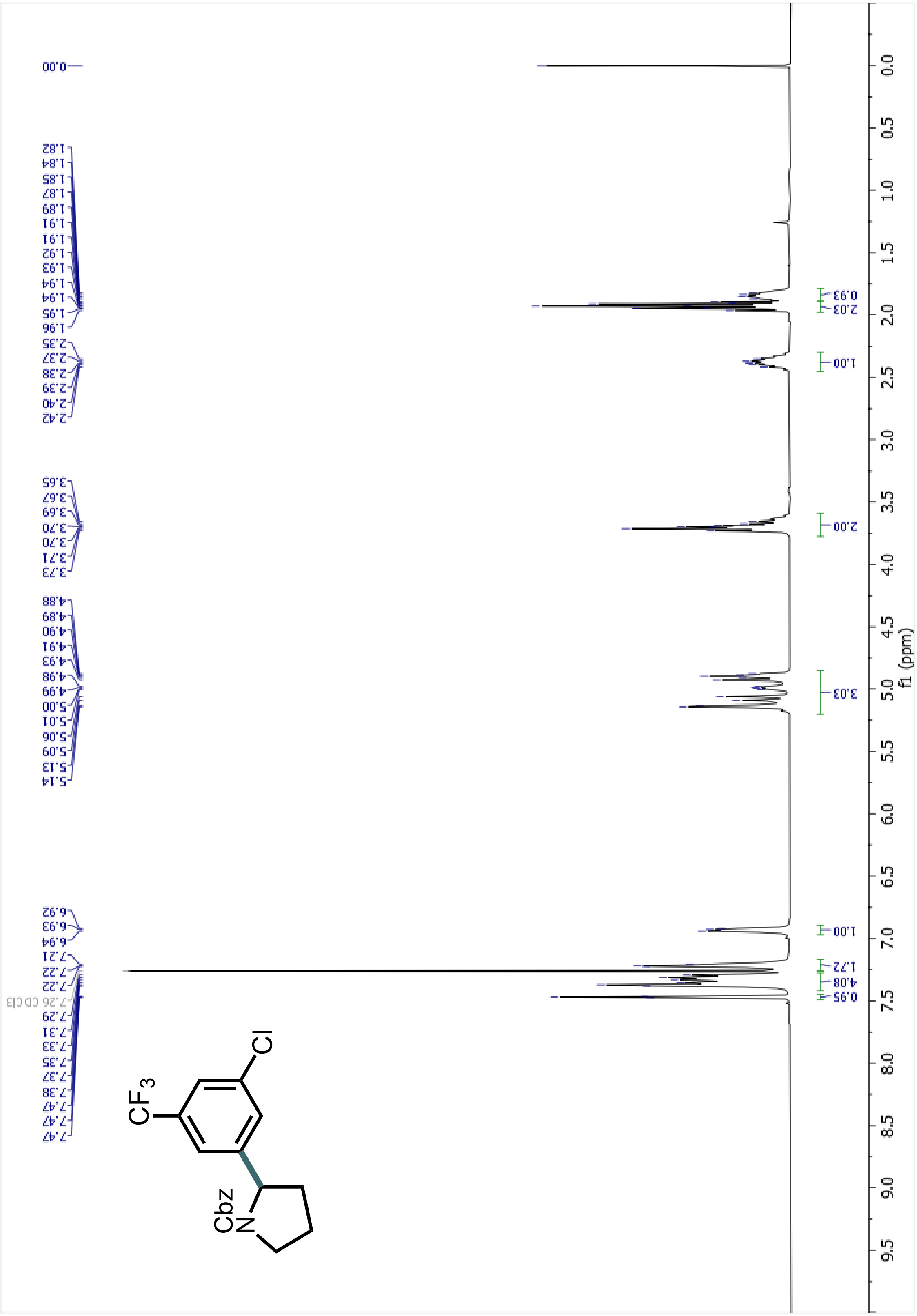

^{13}C{^{1}H} NMR SPECTRUM OF **28** (201 MHz, $CDCl_3$):

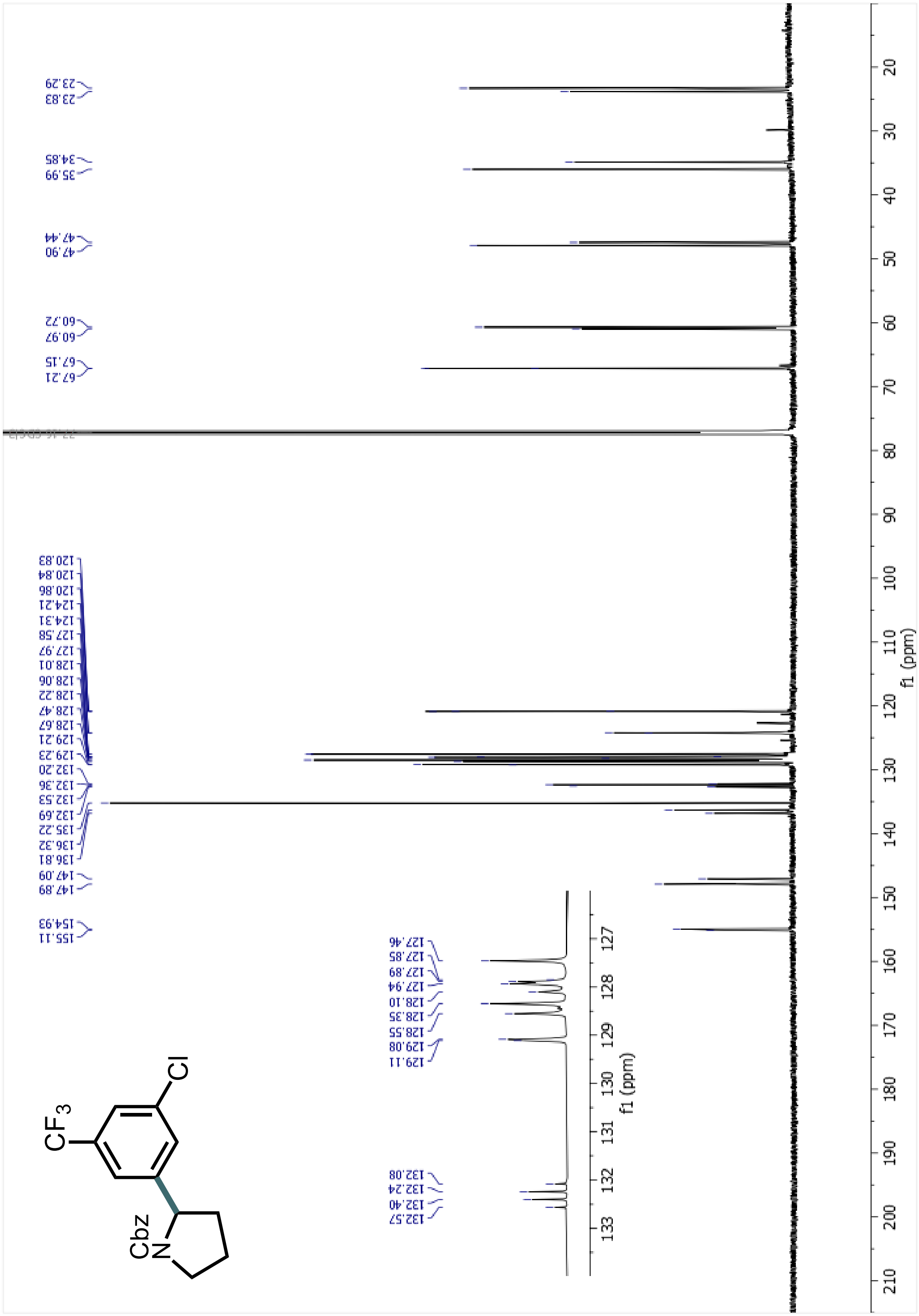

^{19}F NMR SPECTRUM OF **28** (377 MHz, $CDCl_3$):

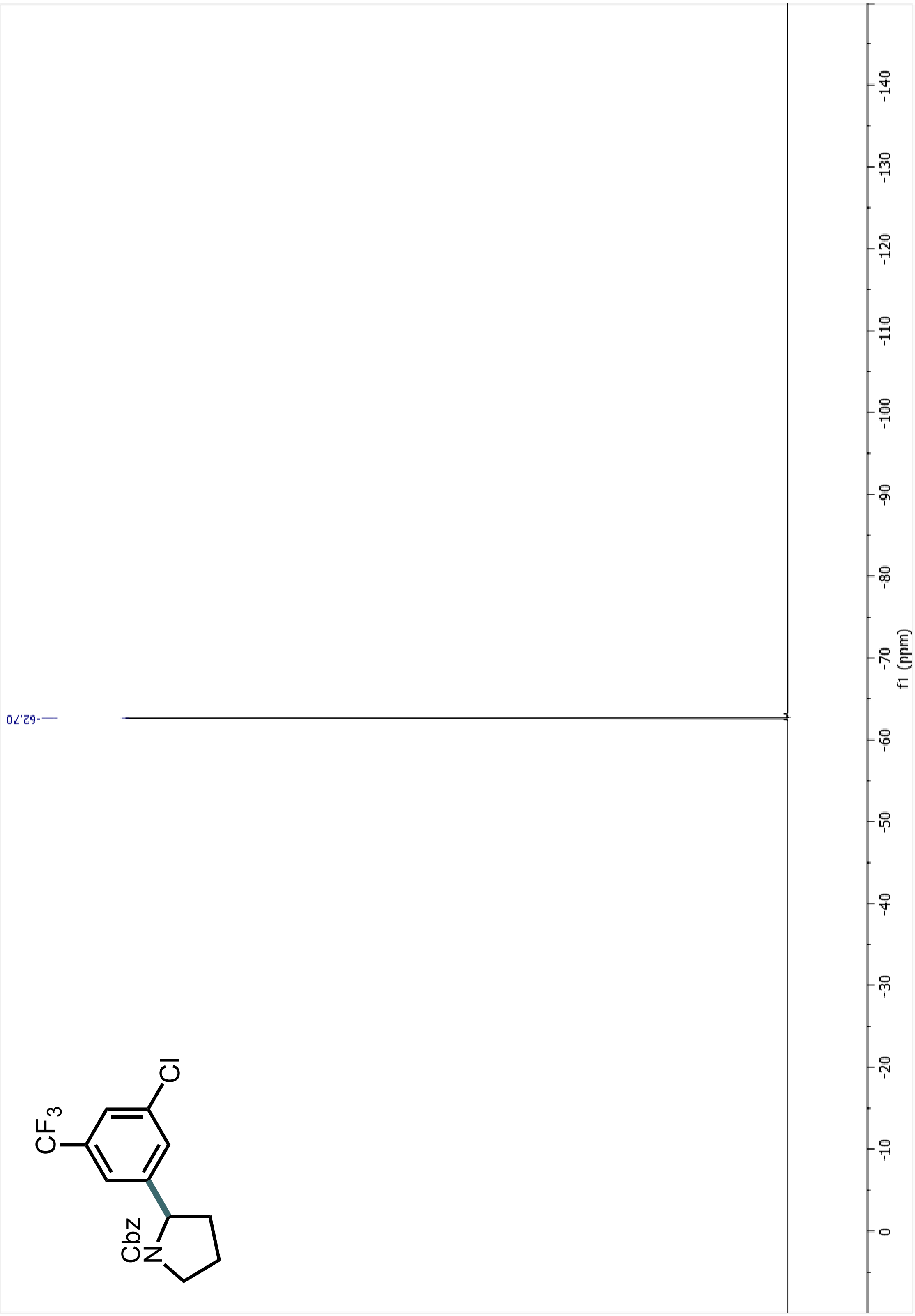

^{1}H NMR SPECTRUM OF **29** (400 MHz, $CDCl_3$):

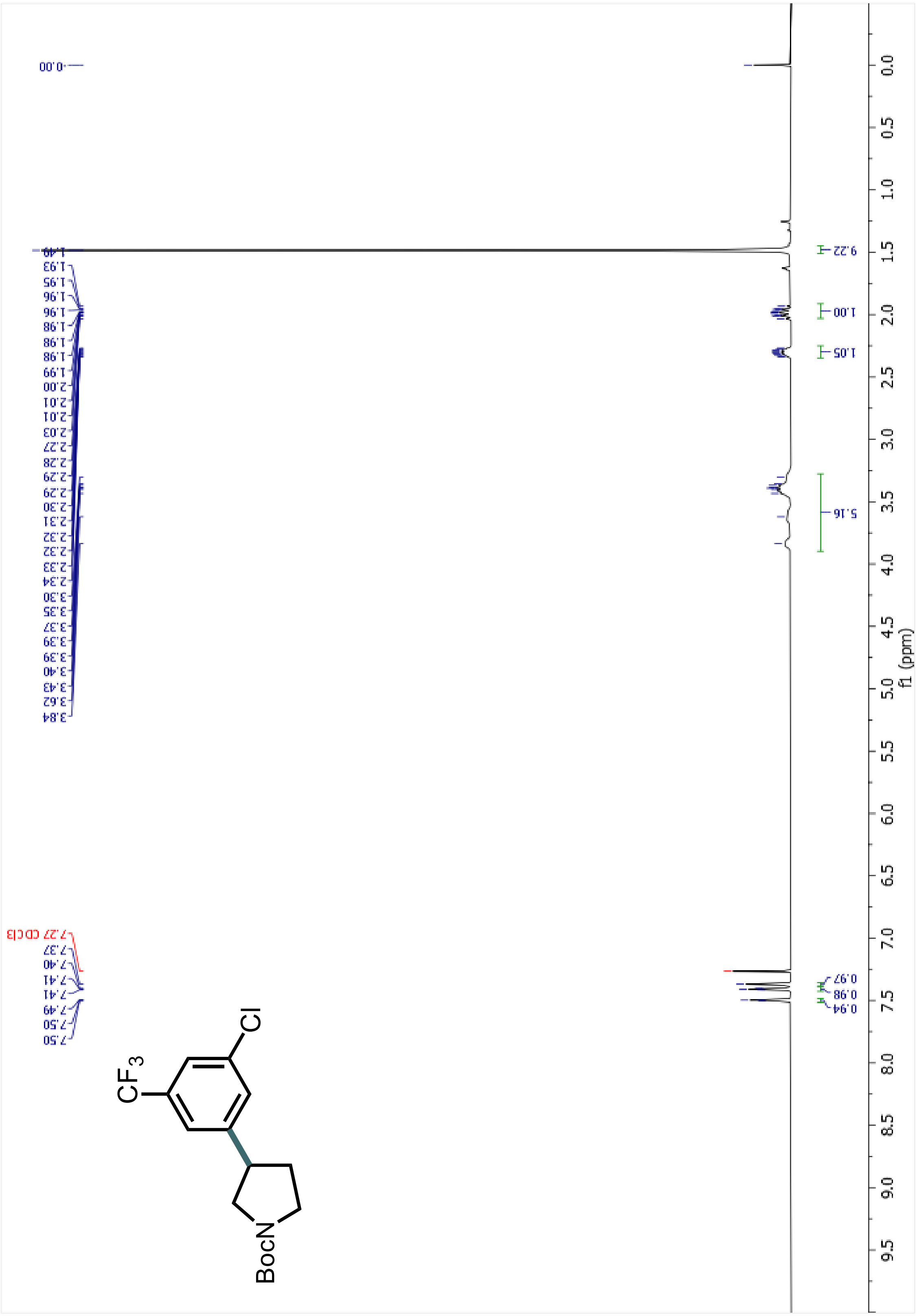

$^{13}C\{^{1}H\}$ NMR SPECTRUM OF **29** (201 MHz, $CDCl_3$):

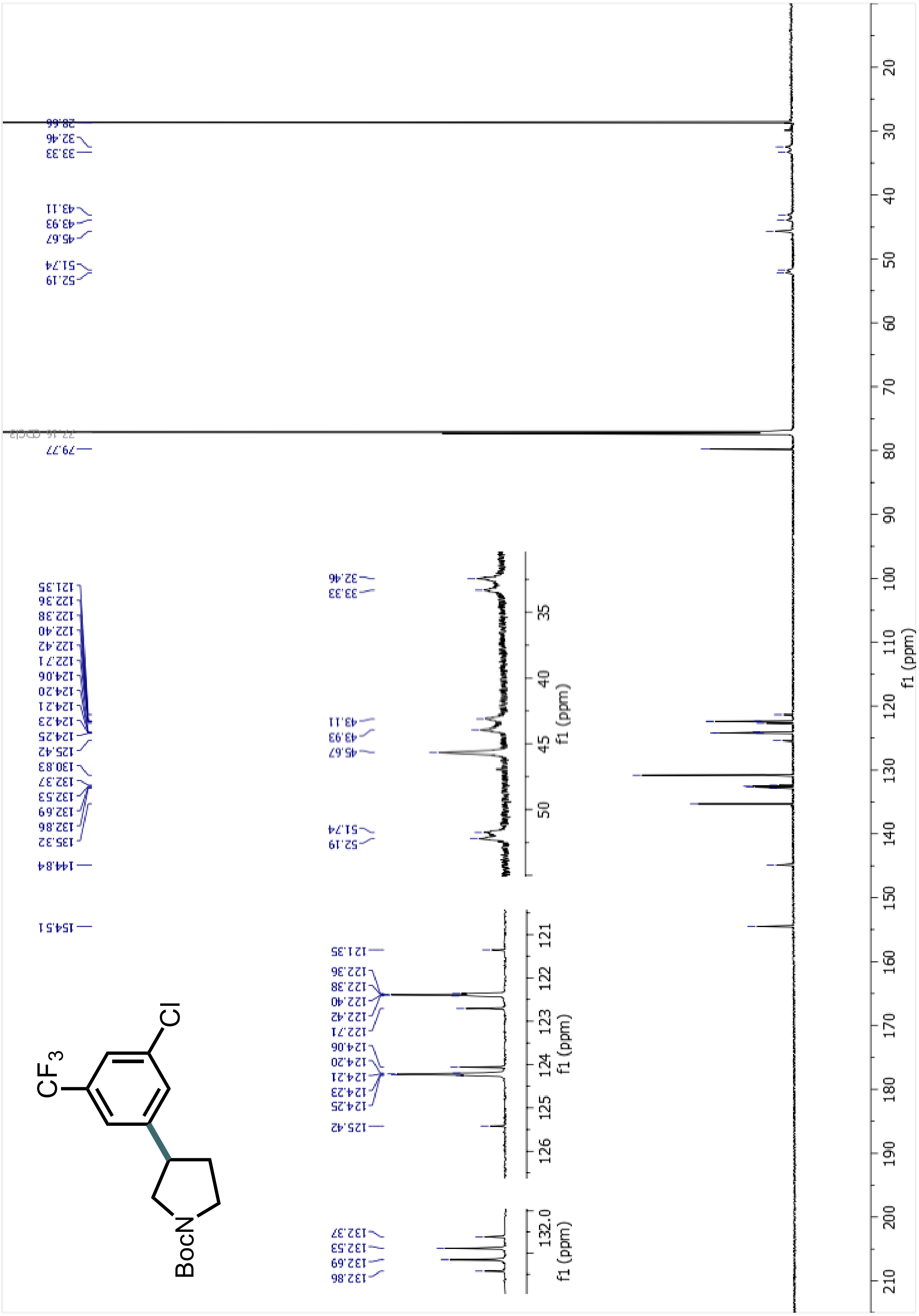

^{19}F NMR SPECTRUM OF **29** (377 MHz, $CDCl_3$):

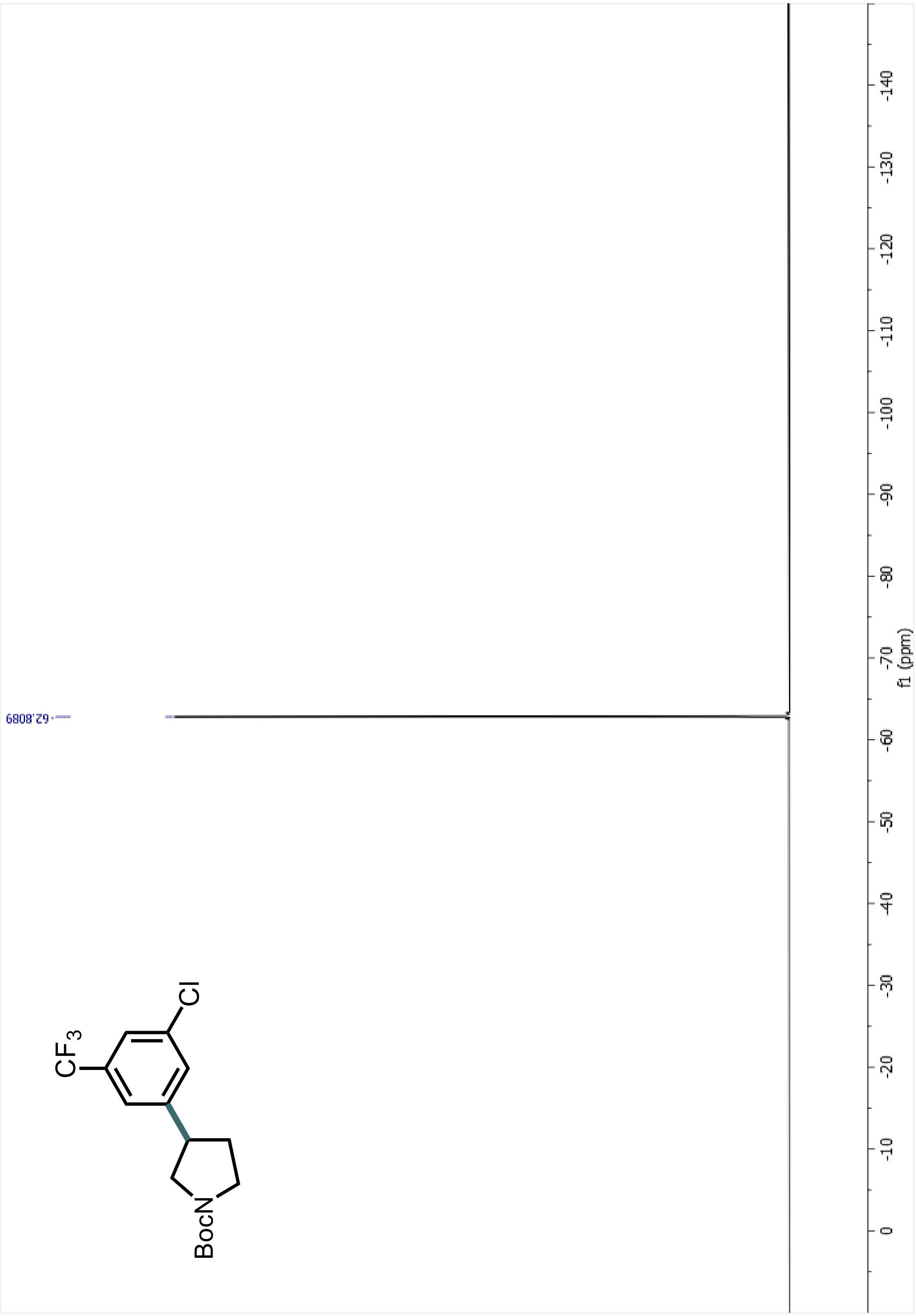

^{1}H NMR SPECTRUM OF **30** (400 MHZ, $CDCl_3$):

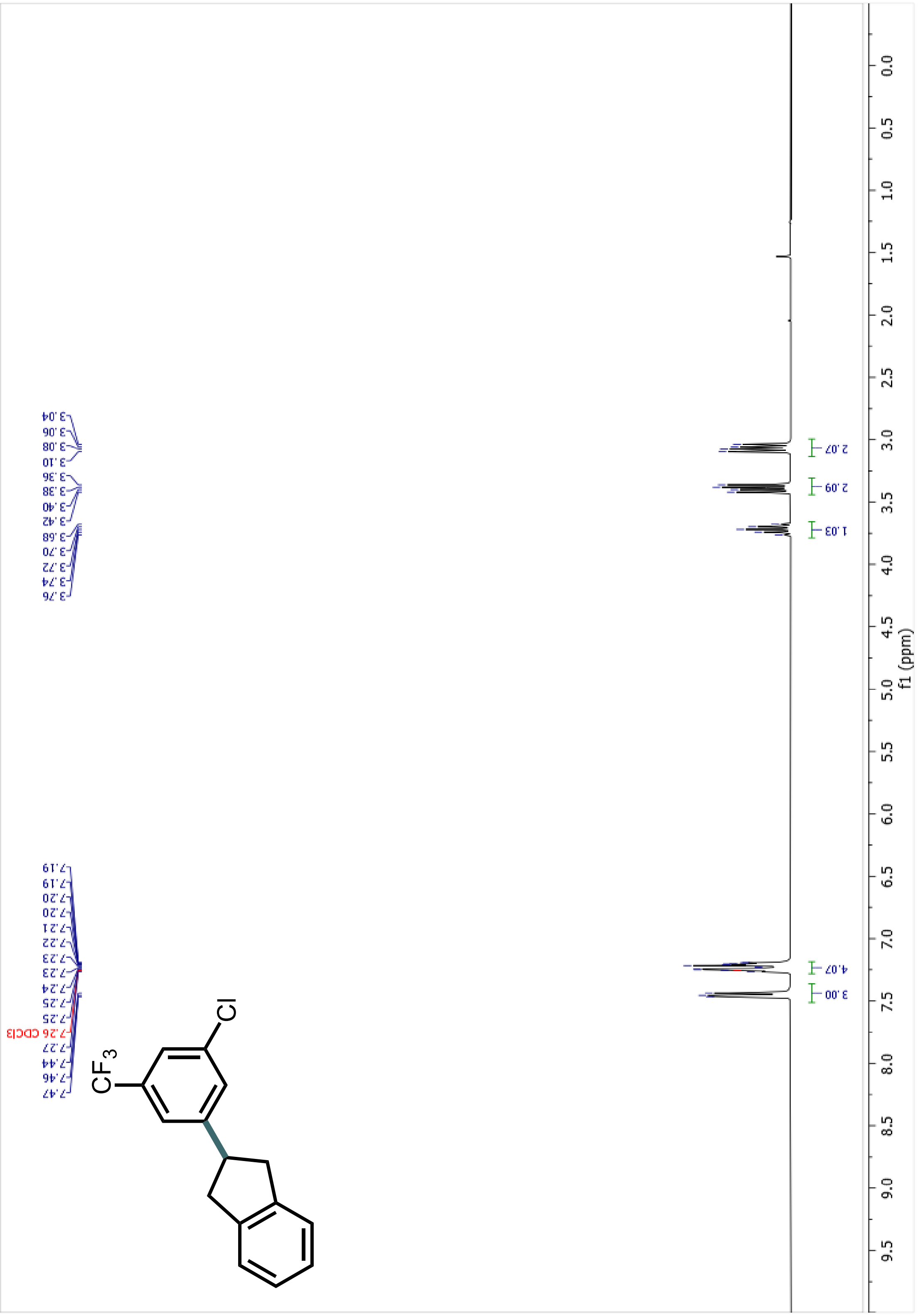

$^{13}C\{^{1}H\}$ NMR SPECTRUM OF **30** (201 MHz, $CDCl_3$):

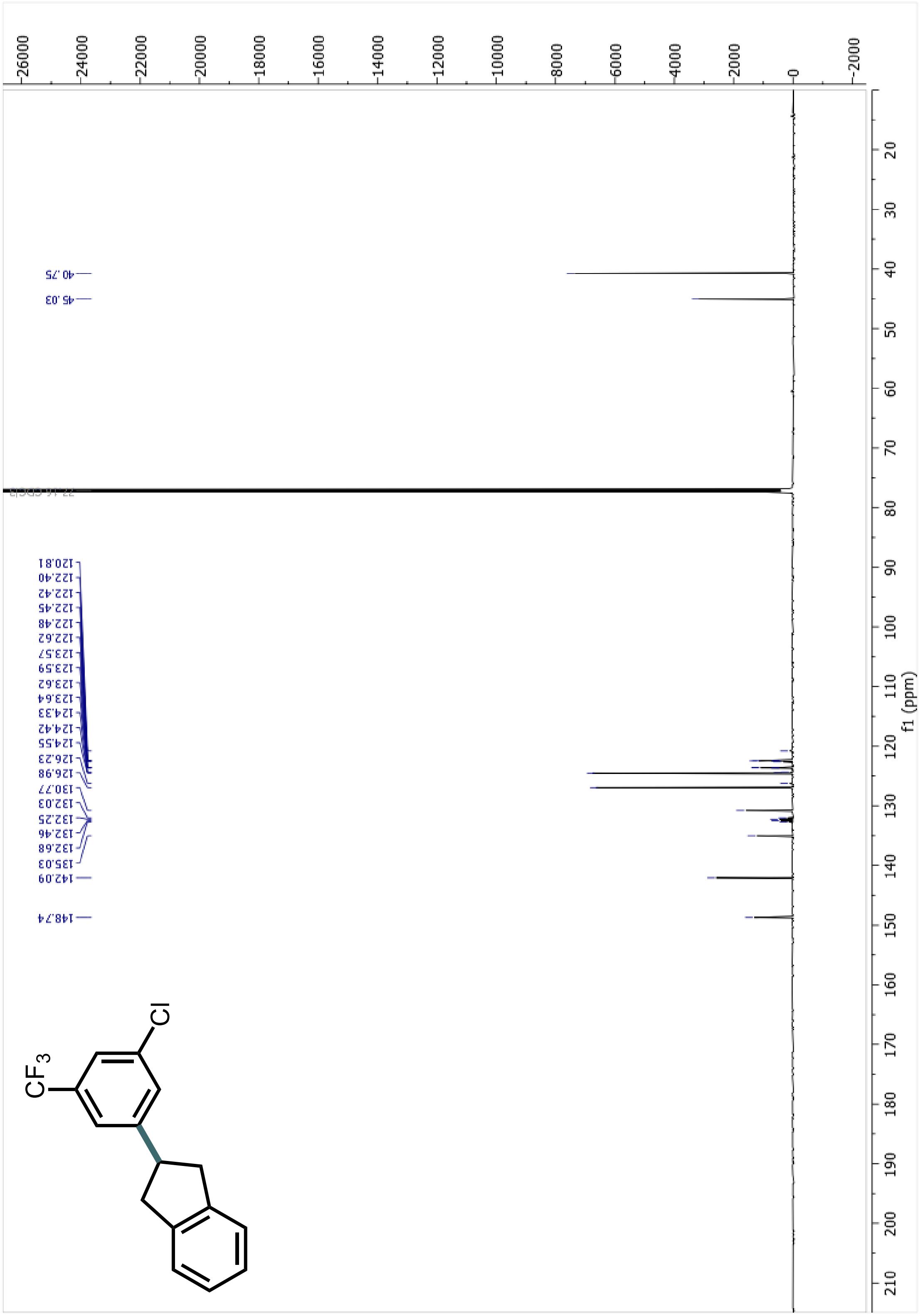

^{19}F NMR SPECTRUM OF **30** (377 MHz, $CDCl_3$):

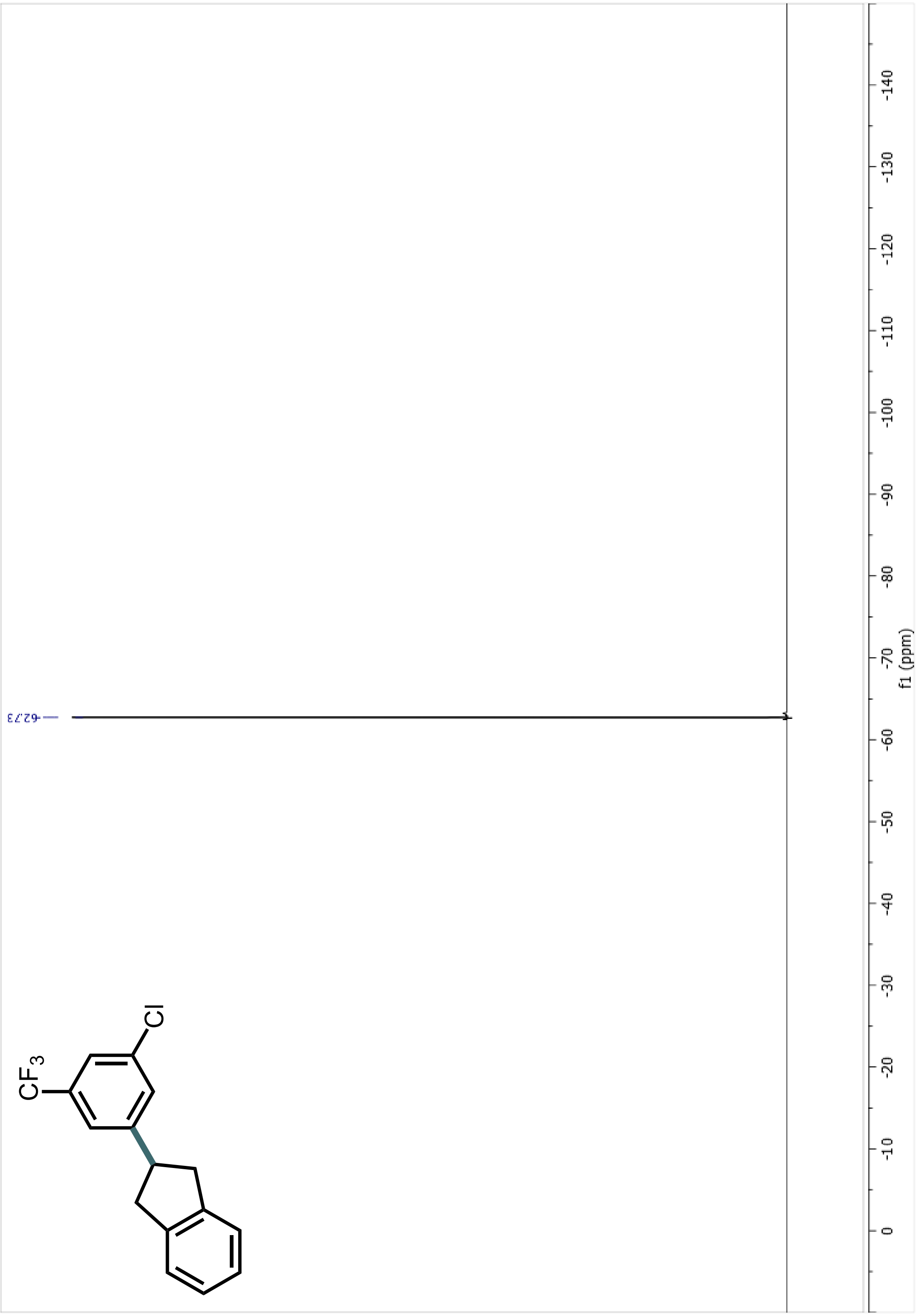

^{1}H NMR SPECTRUM OF **31** (400 MHz, $CDCl_3$):

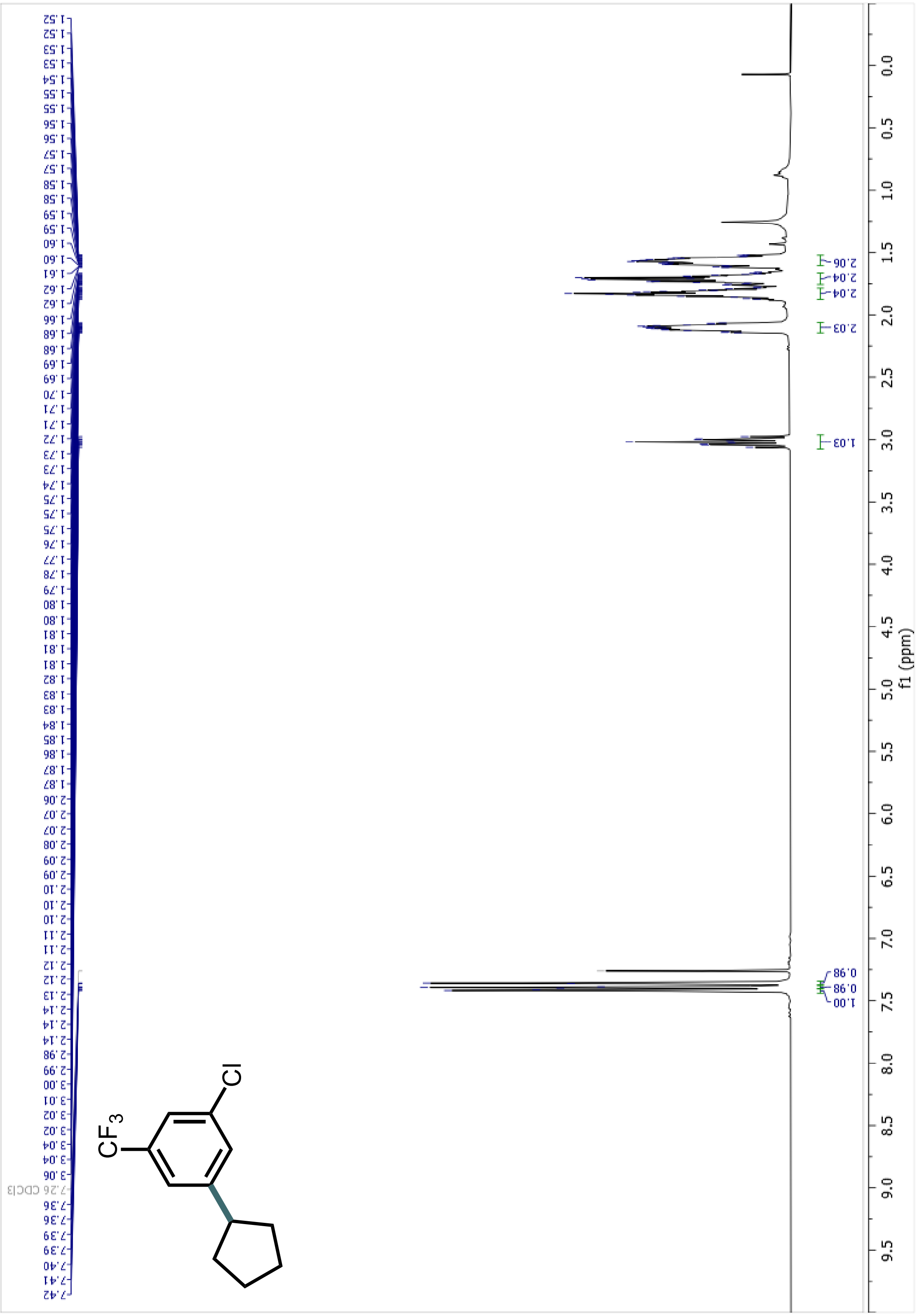

$^{13}C\{^1H\}$ NMR SPECTRUM OF **31** (201 MHz, $CDCl_3$):

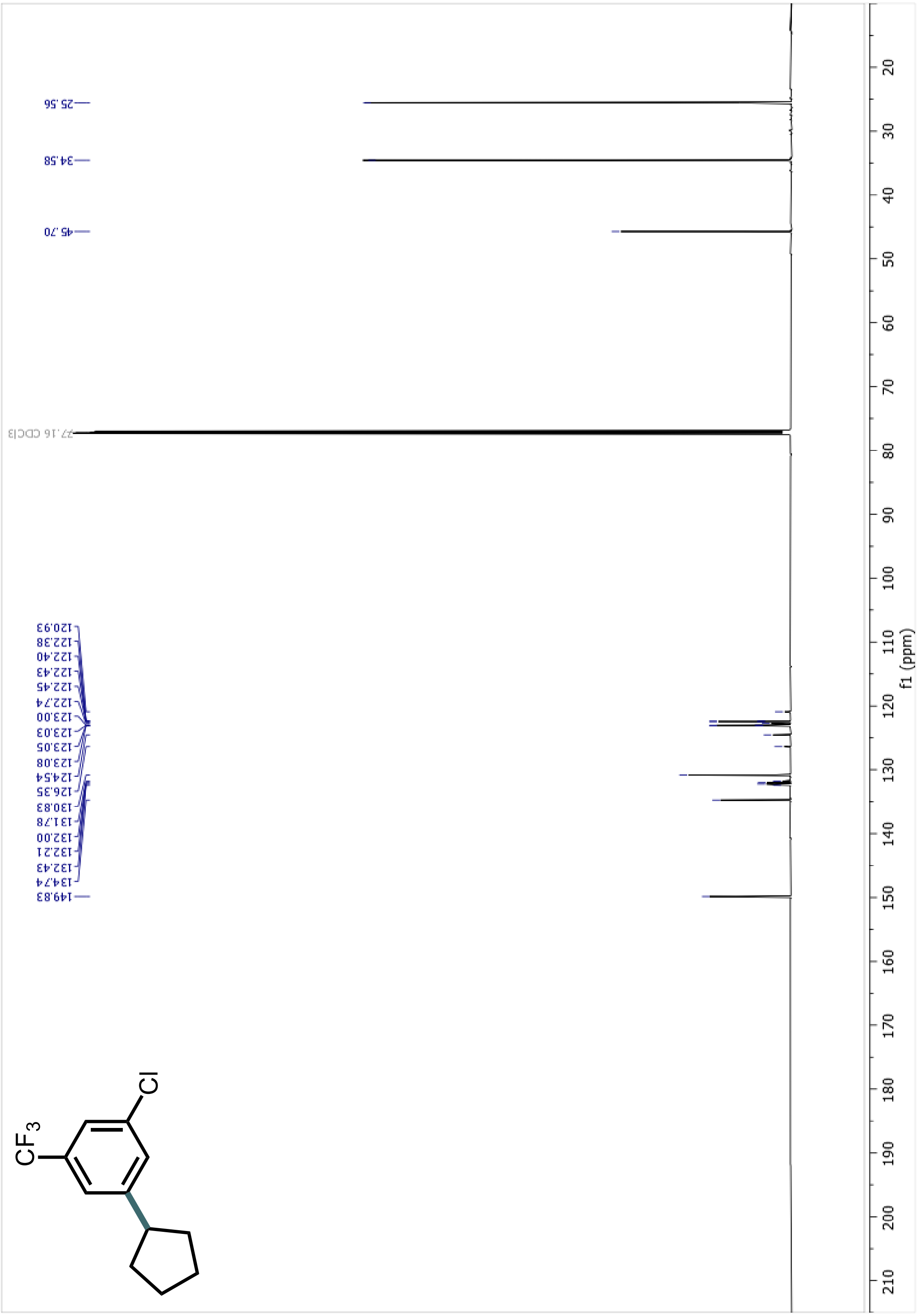

^{19}F NMR SPECTRUM OF **31** (377 MHz, $CDCl_3$):

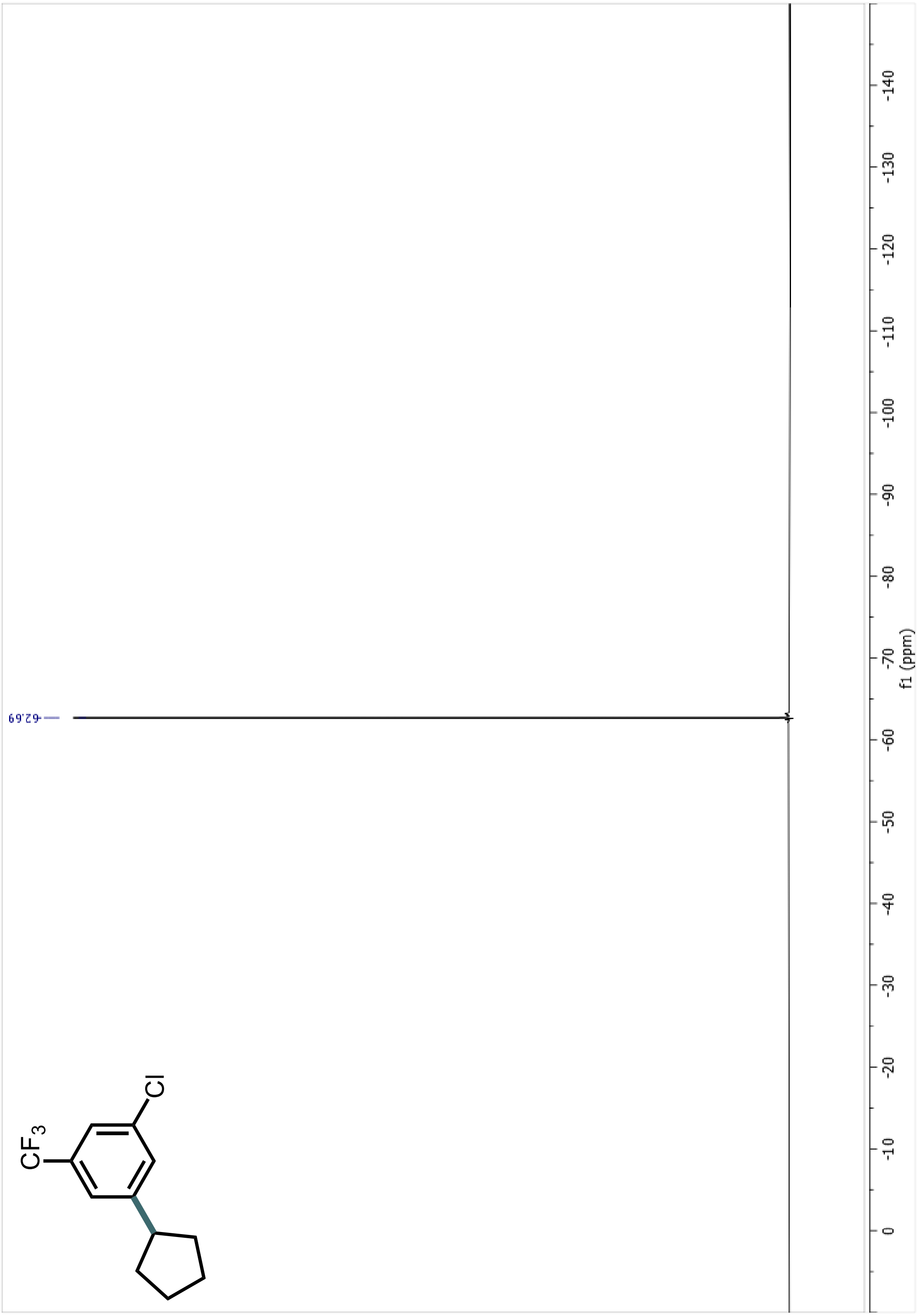

^{1}H NMR SPECTRUM OF **32** (400 MHz, $CDCl_3$):

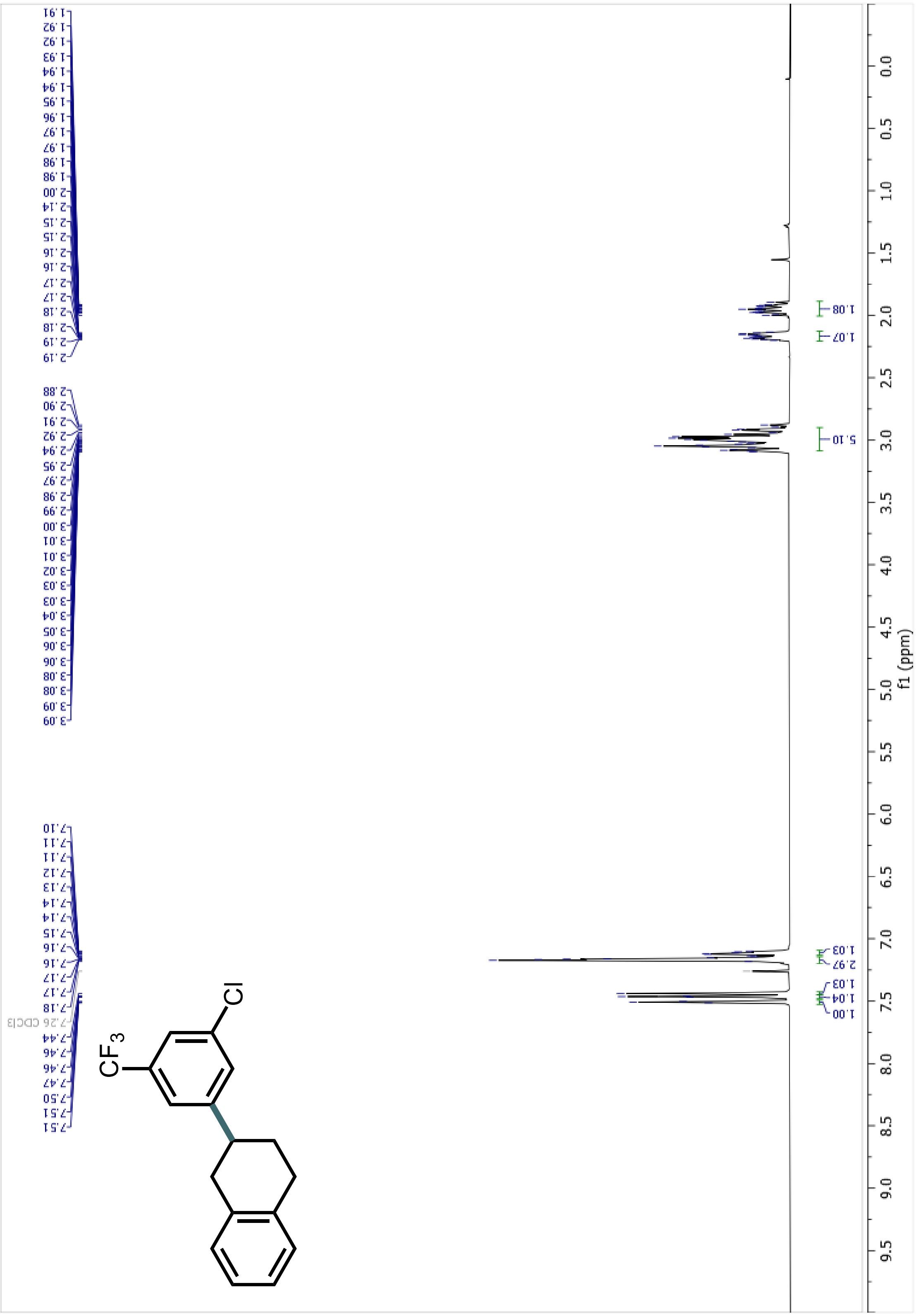

$^{13}C\{^{1}H\}$ NMR SPECTRUM OF **32** (201 MHz, $CDCl_3$):

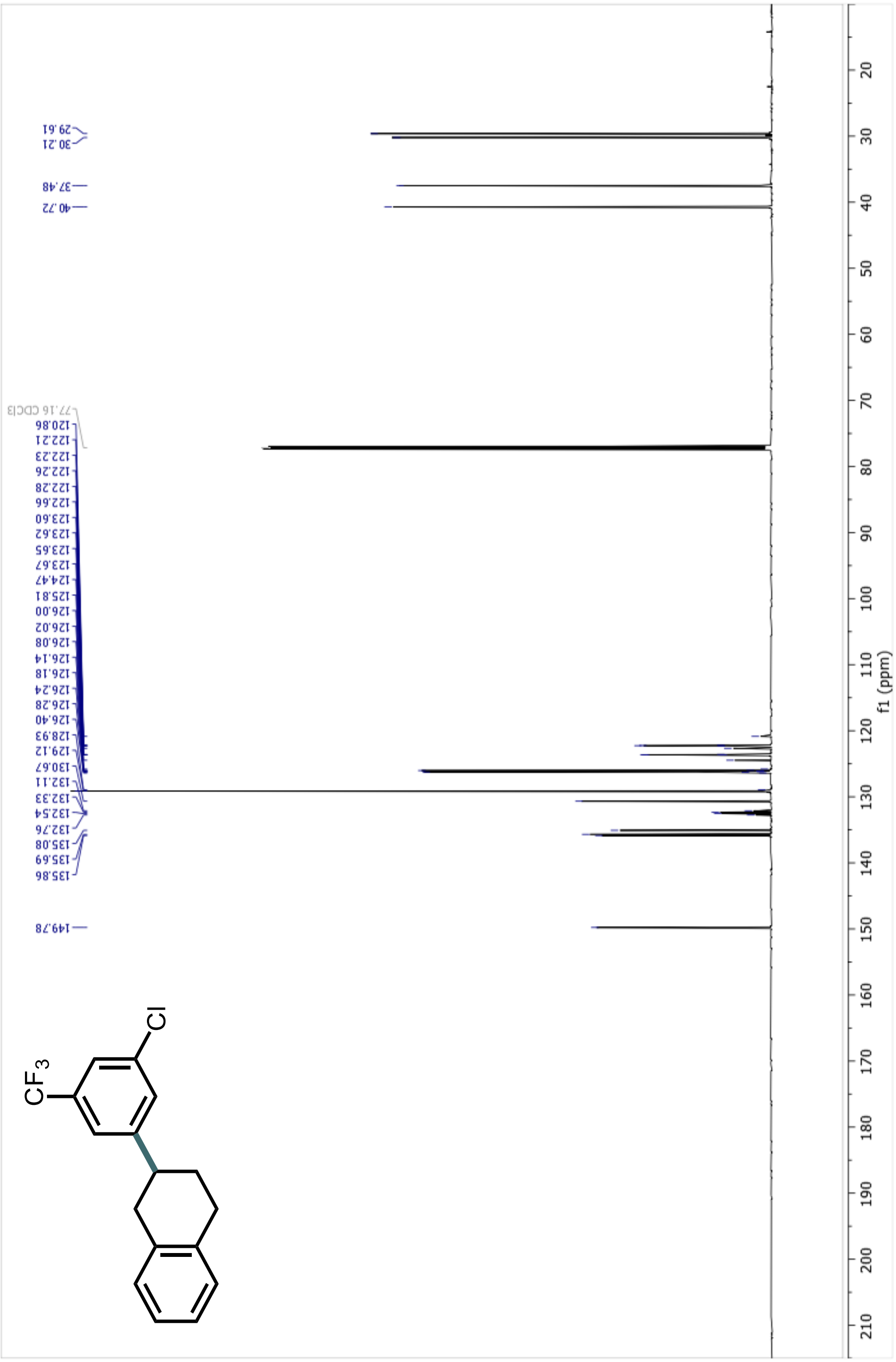

^{19}F NMR SPECTRUM OF **32** (377 MHz, $CDCl_3$):

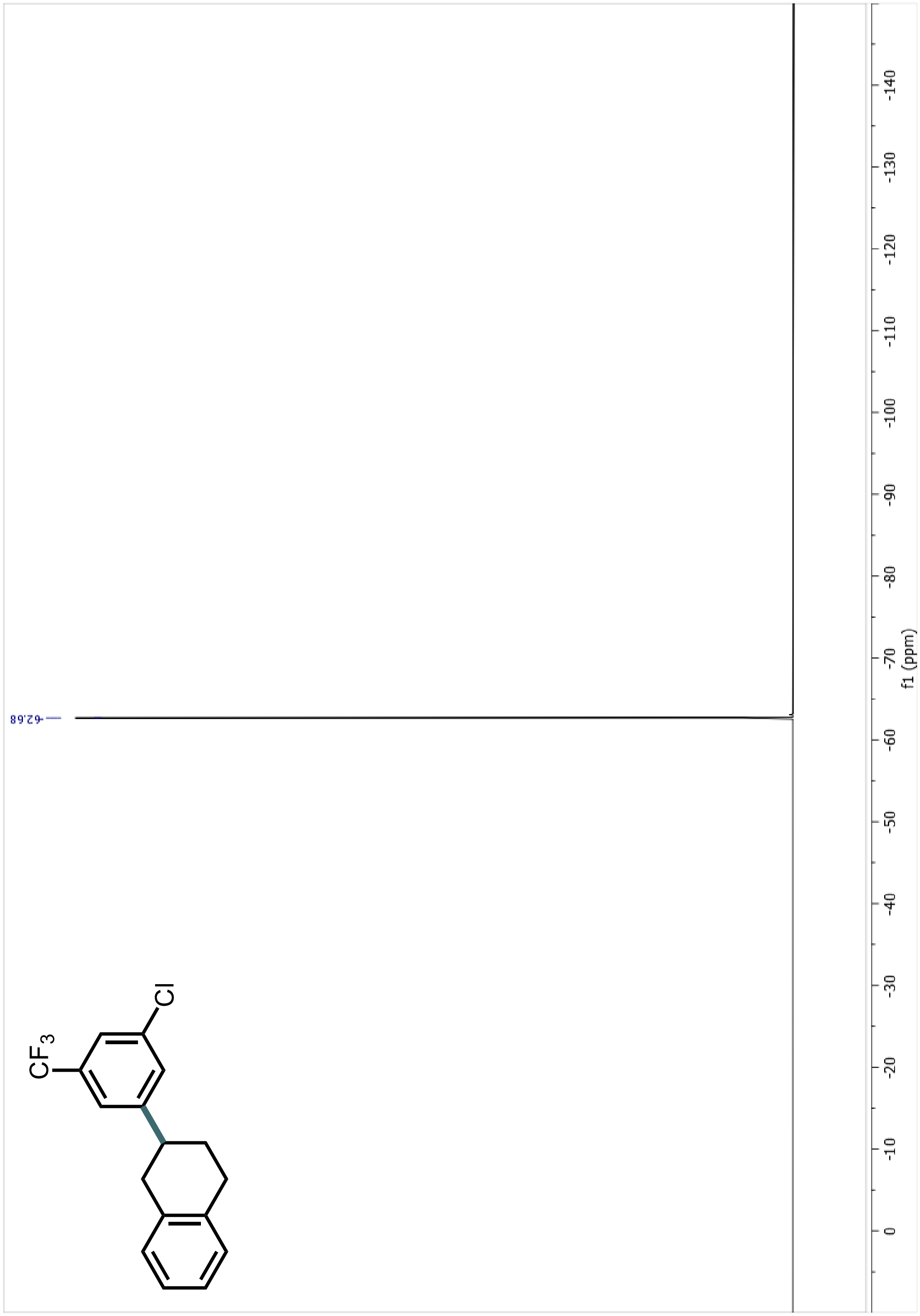

^{1}H NMR SPECTRUM OF **33** (400 MHz, $CDCl_3$):

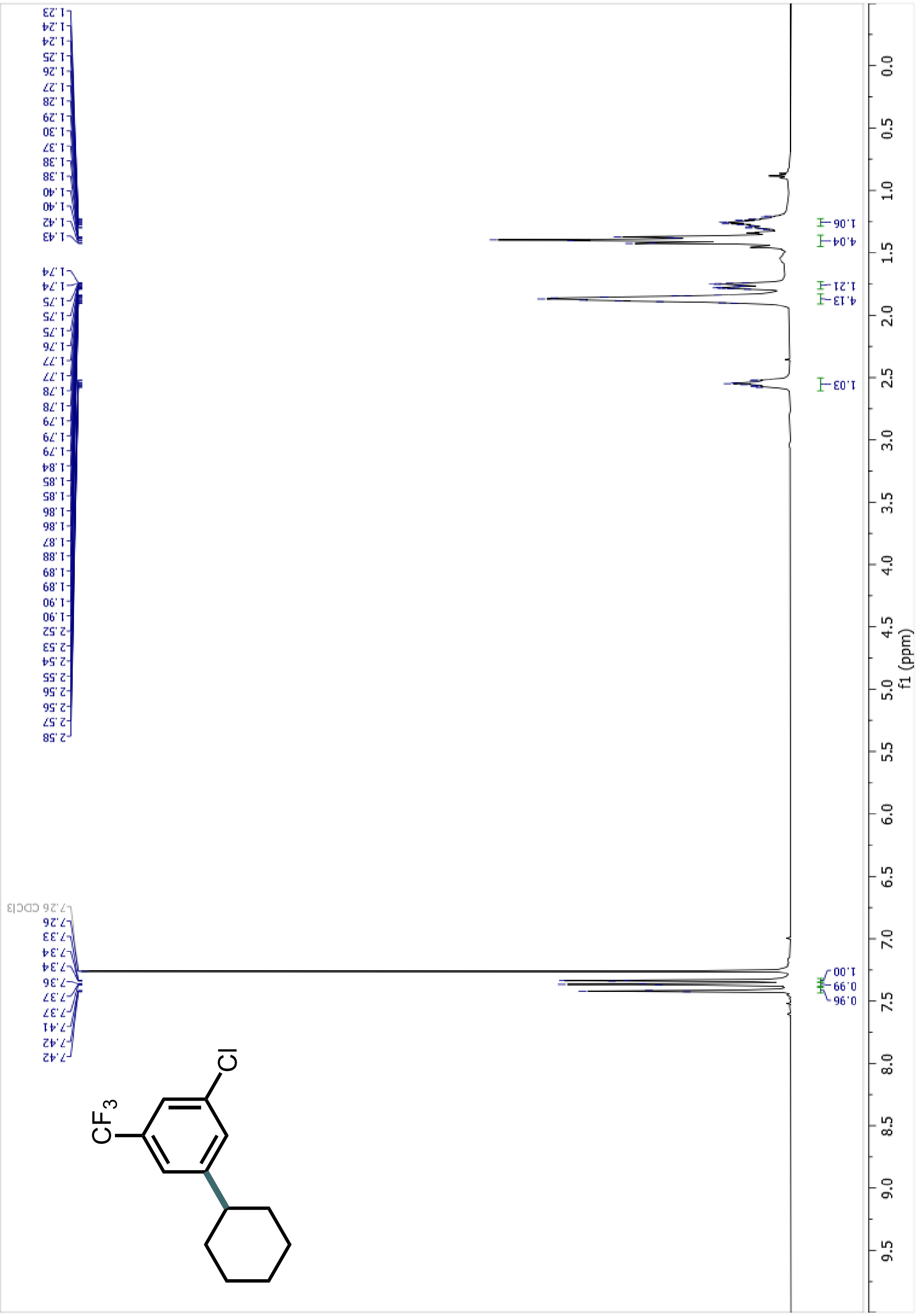

$^{13}C\{^1H\}$ NMR SPECTRUM OF **33** (201 MHz, $CDCl_3$):

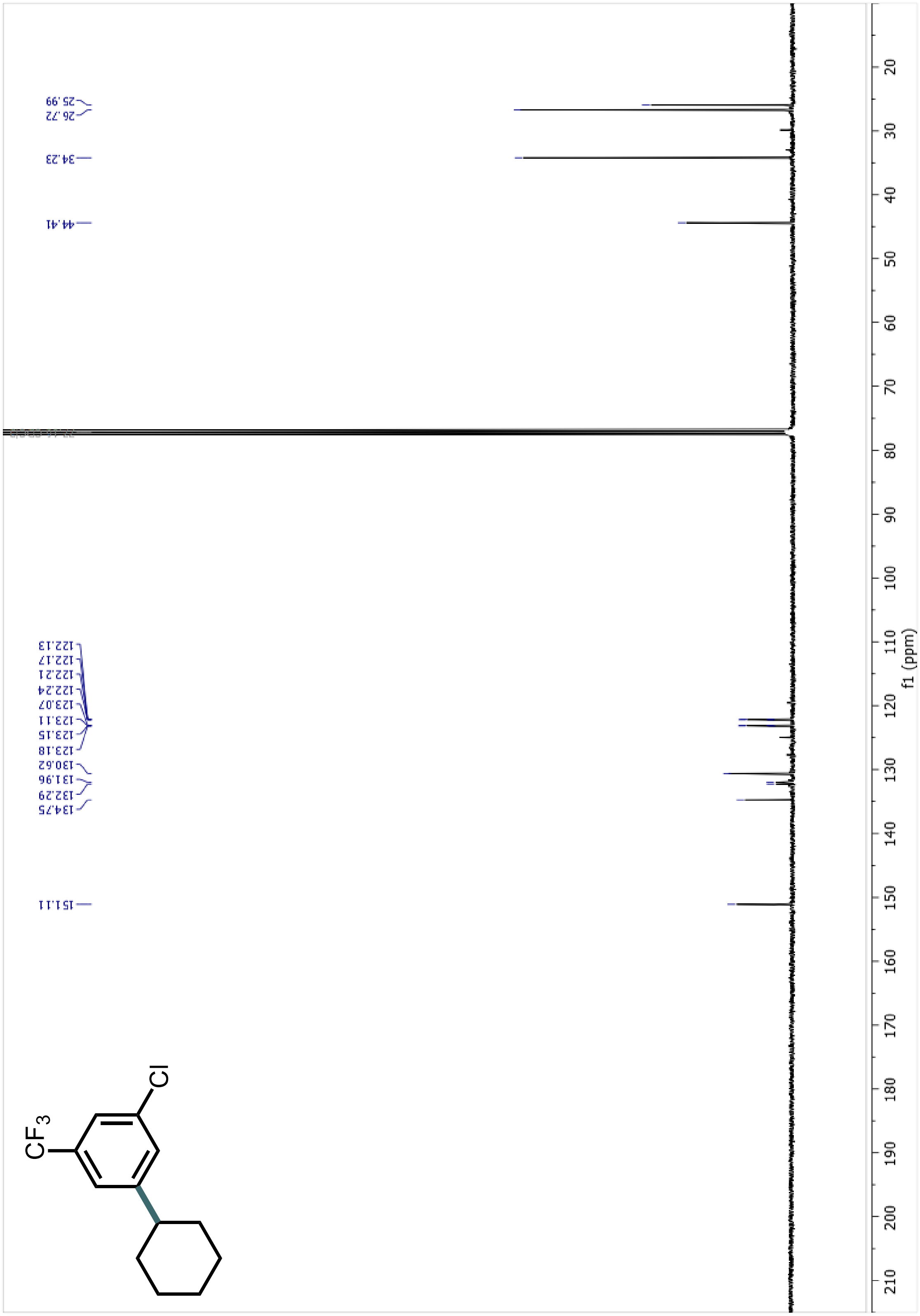

^{19}F NMR SPECTRUM OF **33** (377 MHz, $CDCl_3$):

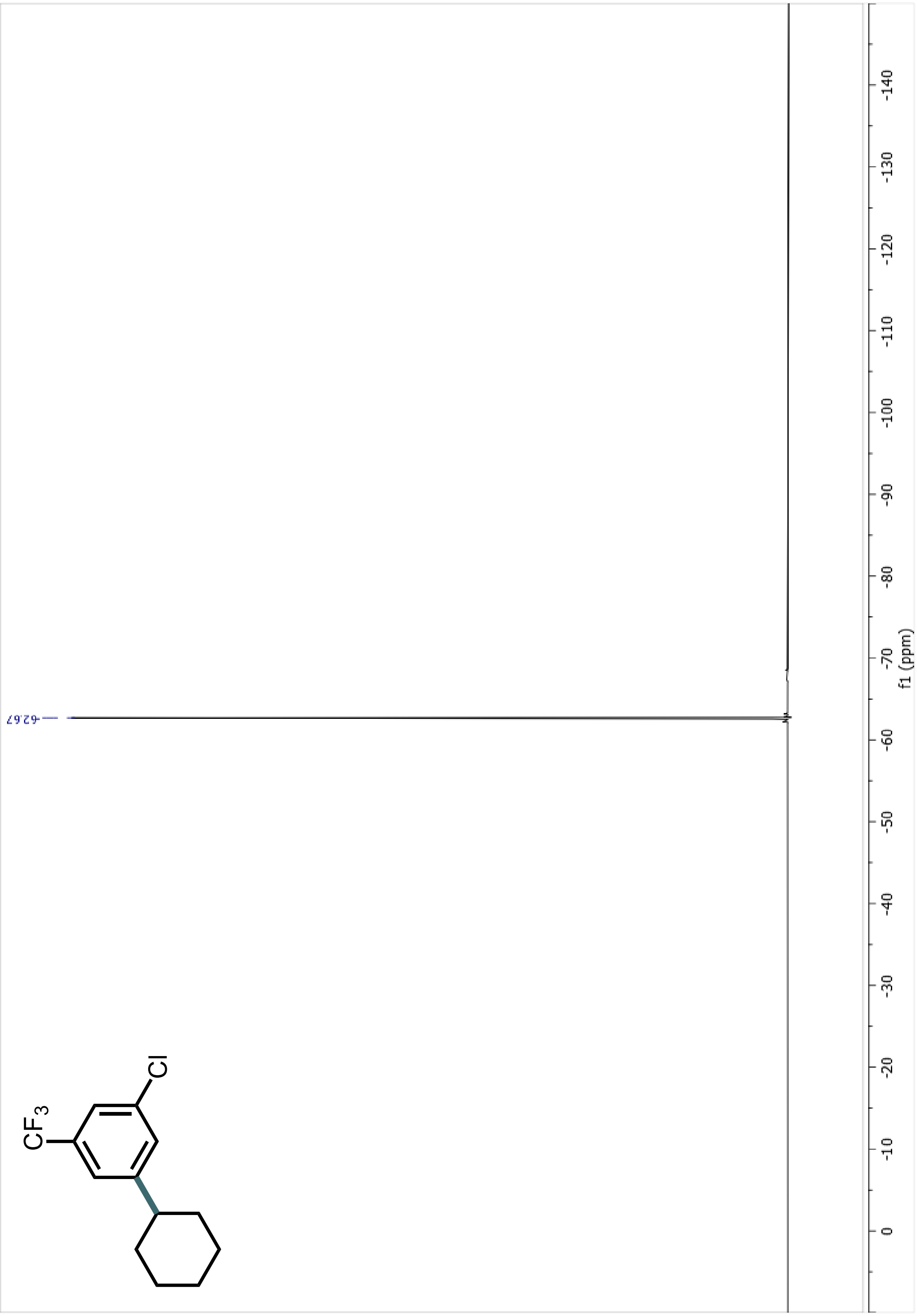

^{1}H NMR SPECTRUM OF **34** (400 MHz, $CDCl_3$):

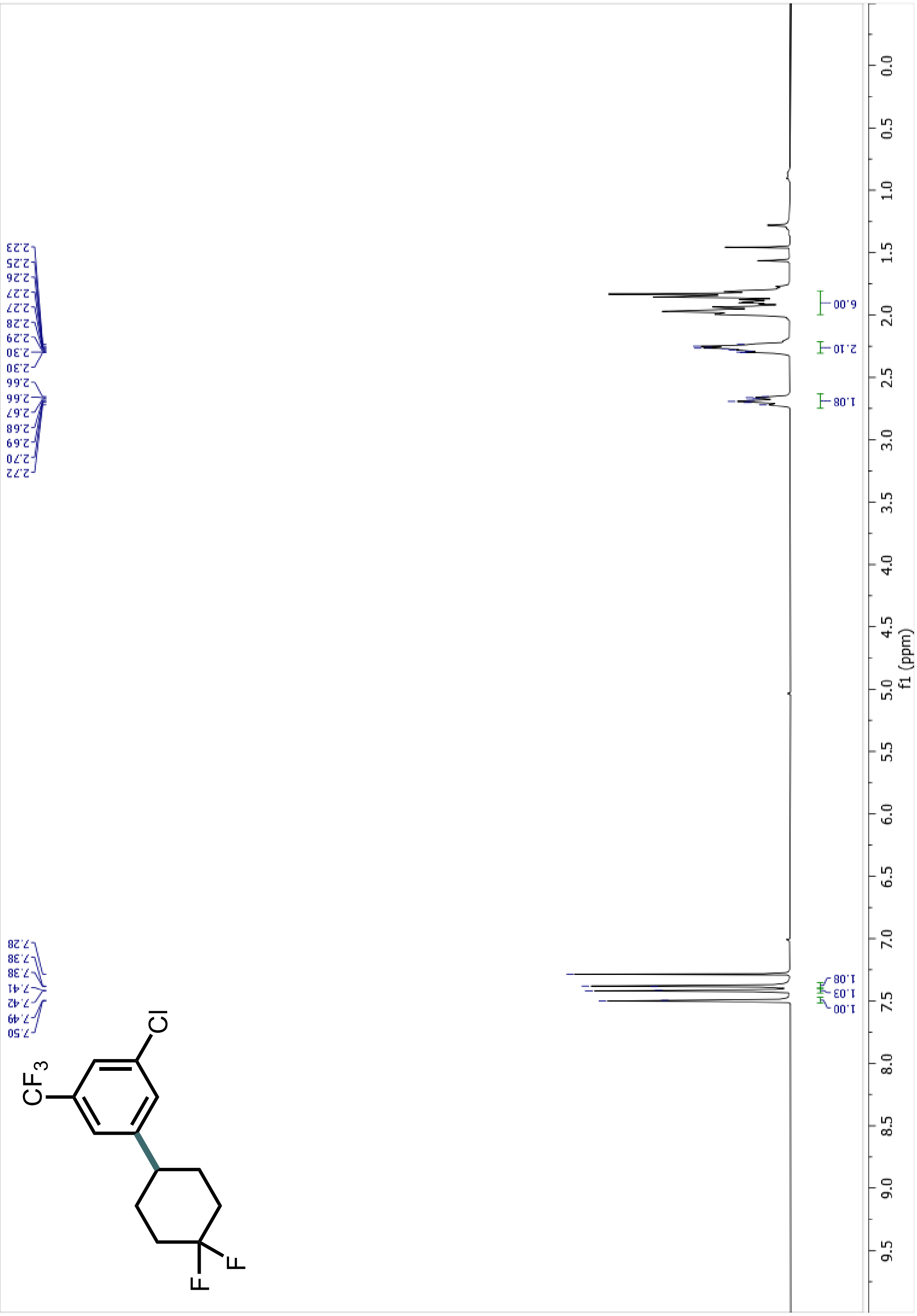

^{13}C{^{1}H} NMR SPECTRUM OF **34** (201 MHz, $CDCl_3$):

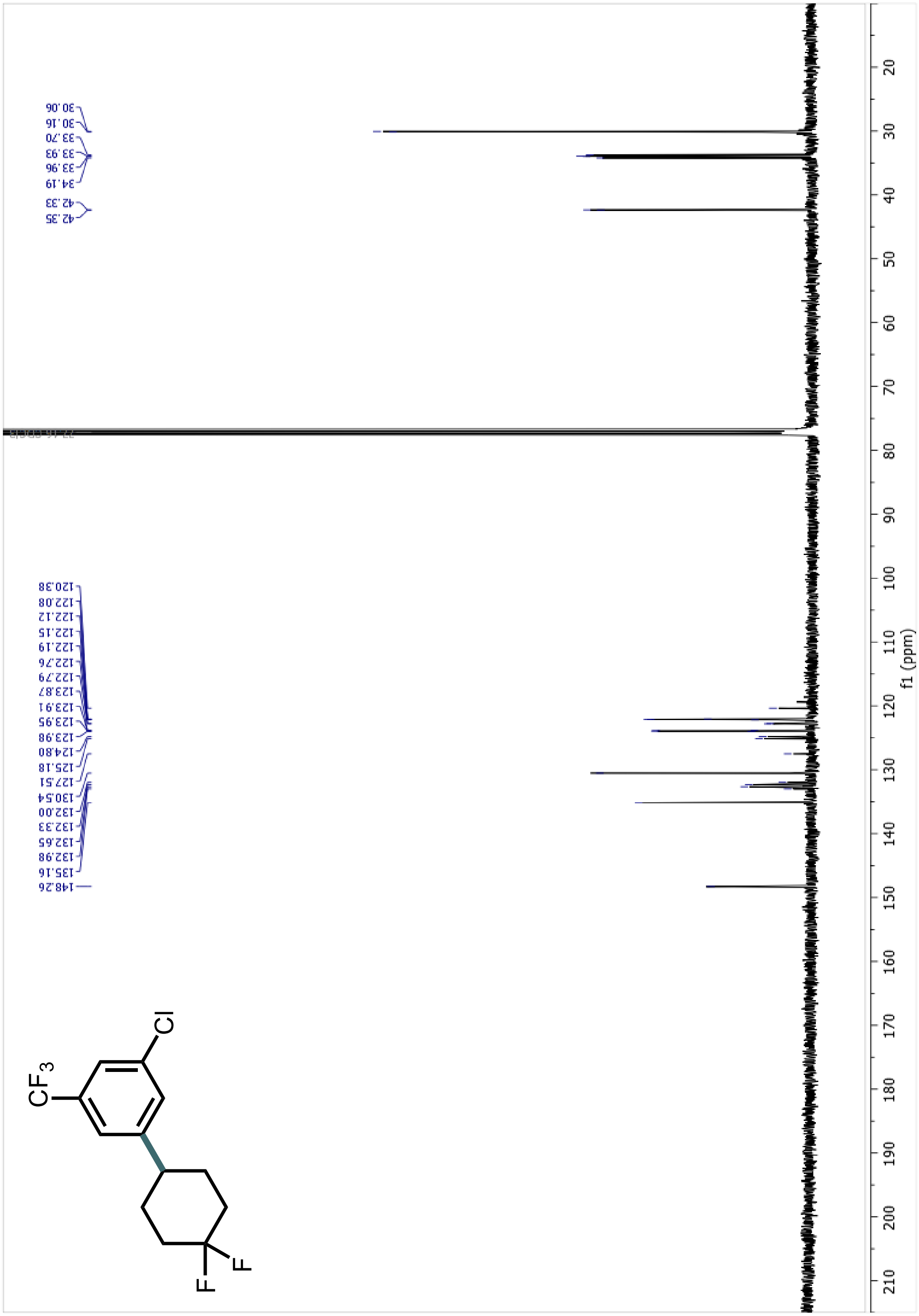

^{19}F NMR SPECTRUM OF **34** (377 MHz, $CDCl_3$):

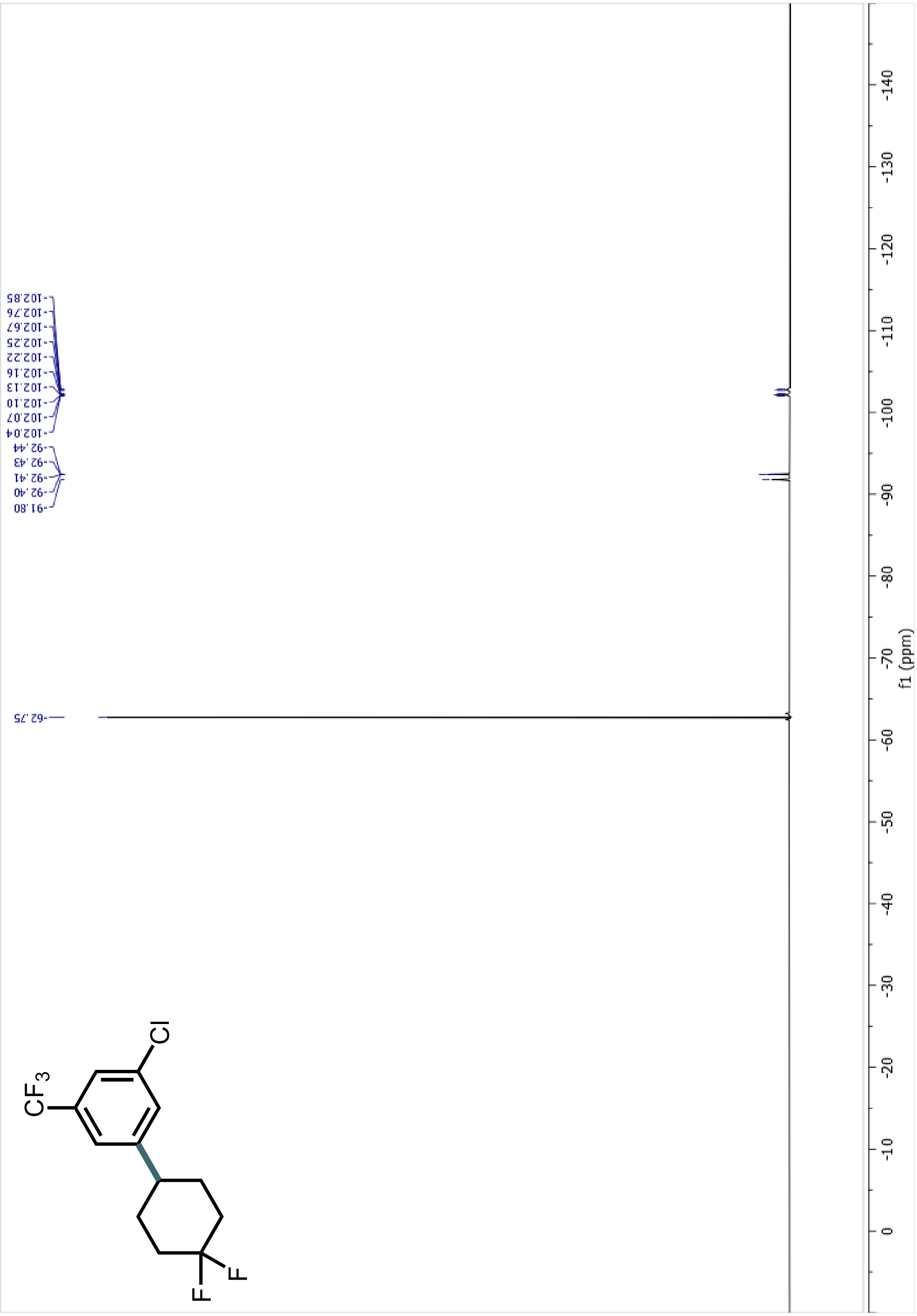

^{1}H NMR SPECTRUM OF **35** (400 MHz, $CDCl_3$):

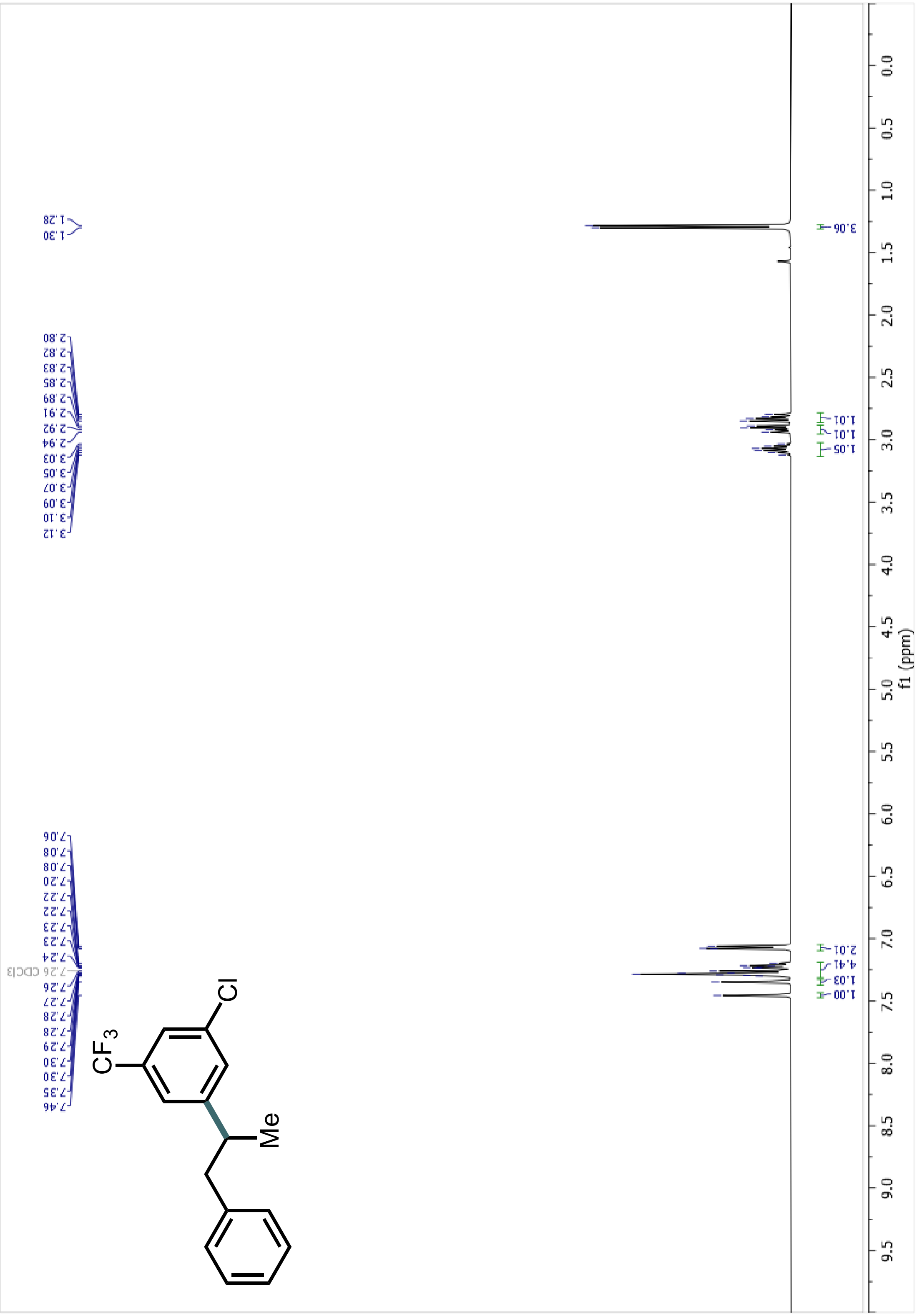

$^{13}C\{^{1}H\}$ NMR SPECTRUM OF **35** (201 MHz, $CDCl_3$):

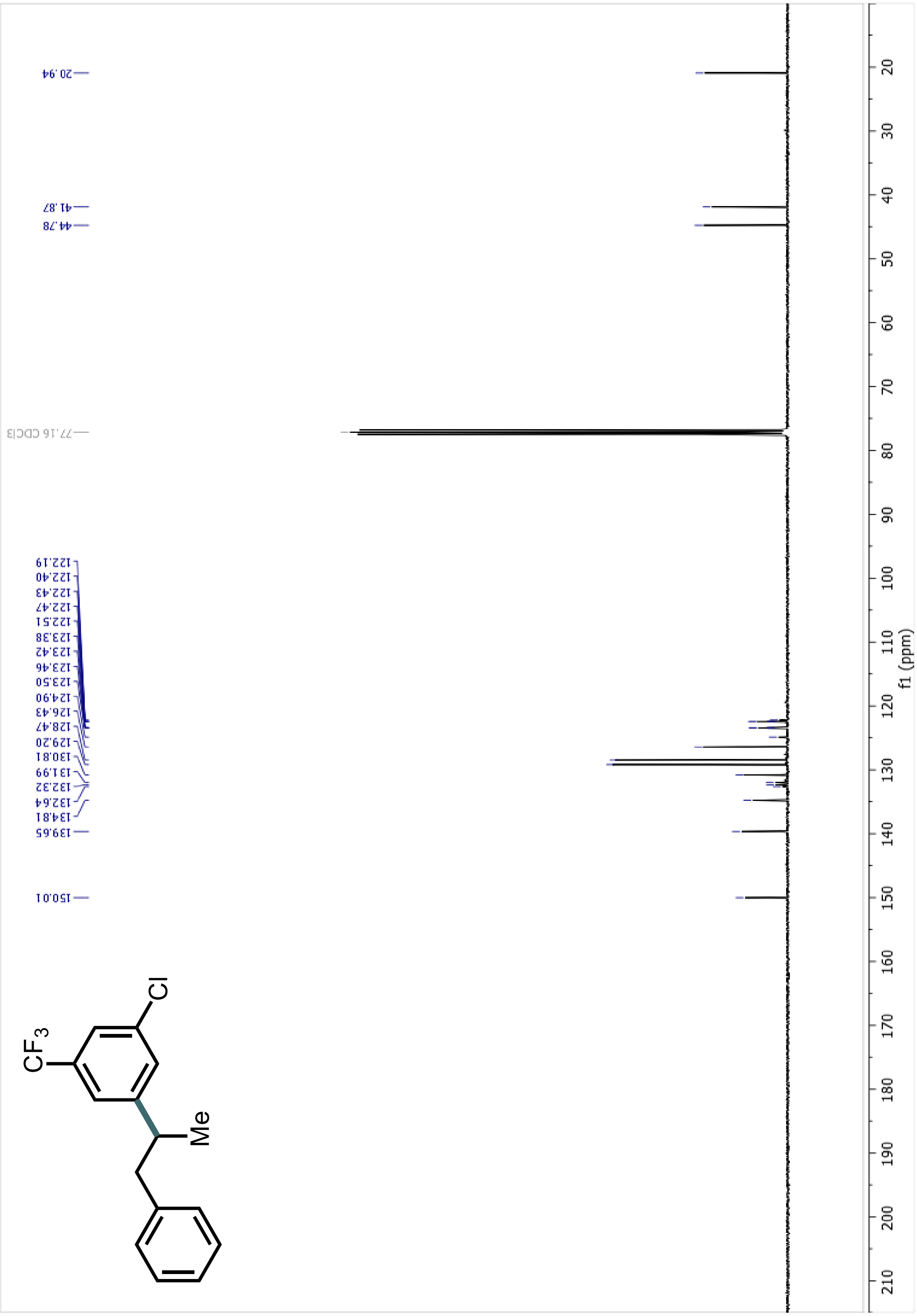

^{19}F NMR SPECTRUM OF **35** (377 MHz, $CDCl_3$):

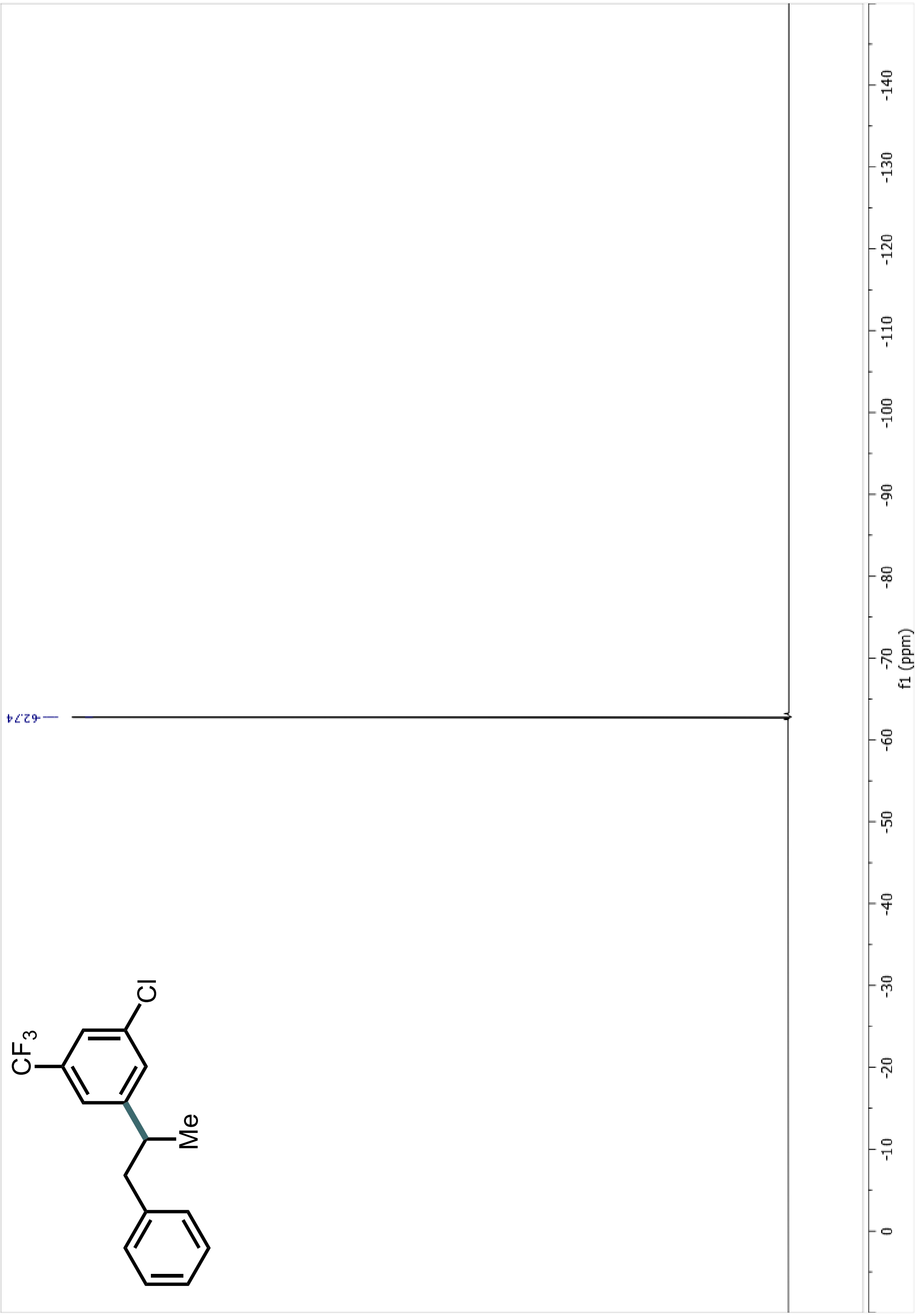

^{1}H NMR SPECTRUM OF **36** (400 MHz, $CDCl_3$):

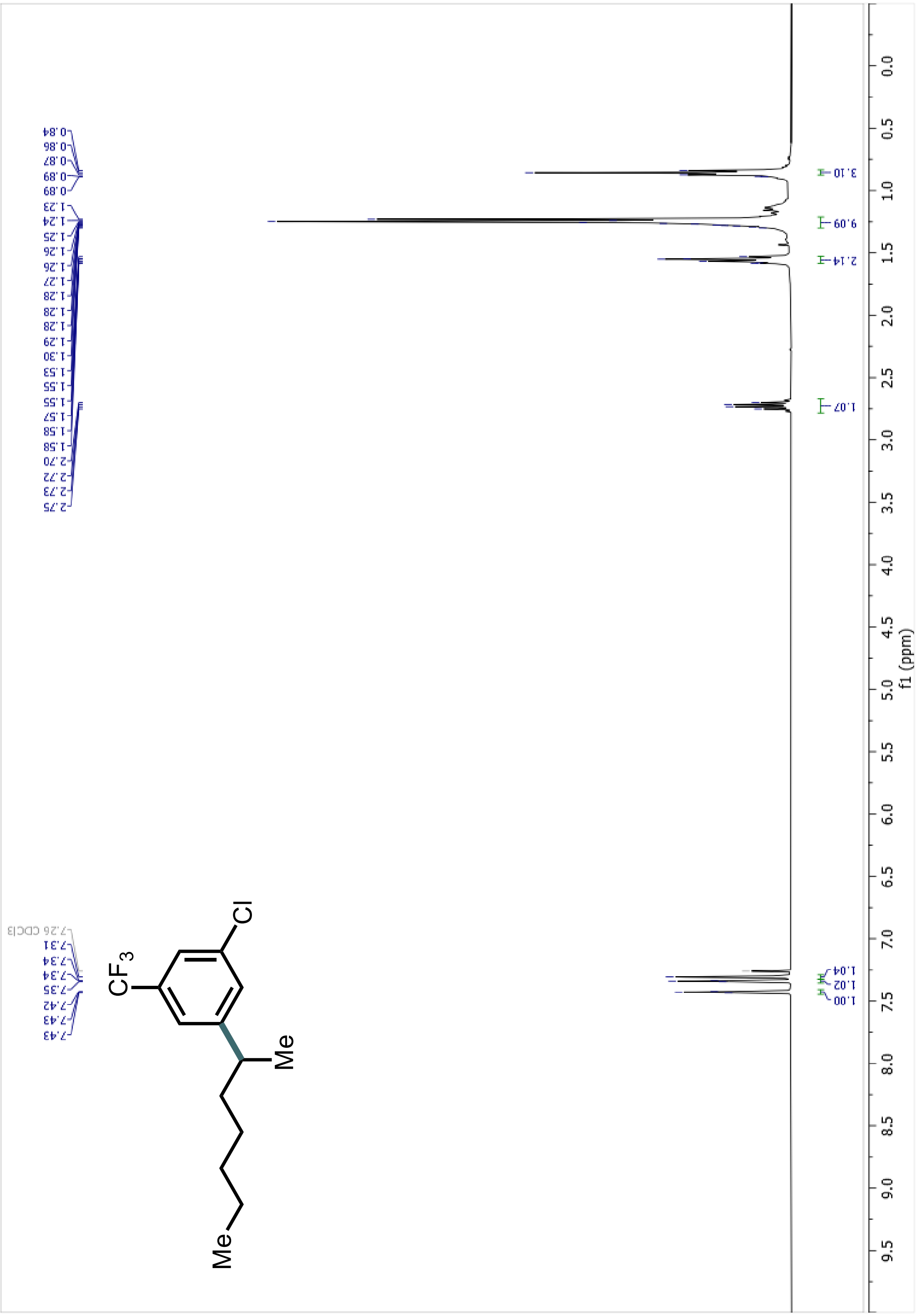

$^{13}C\{^{1}H\}$ NMR SPECTRUM OF **36** (201 MHz, $CDCl_3$):

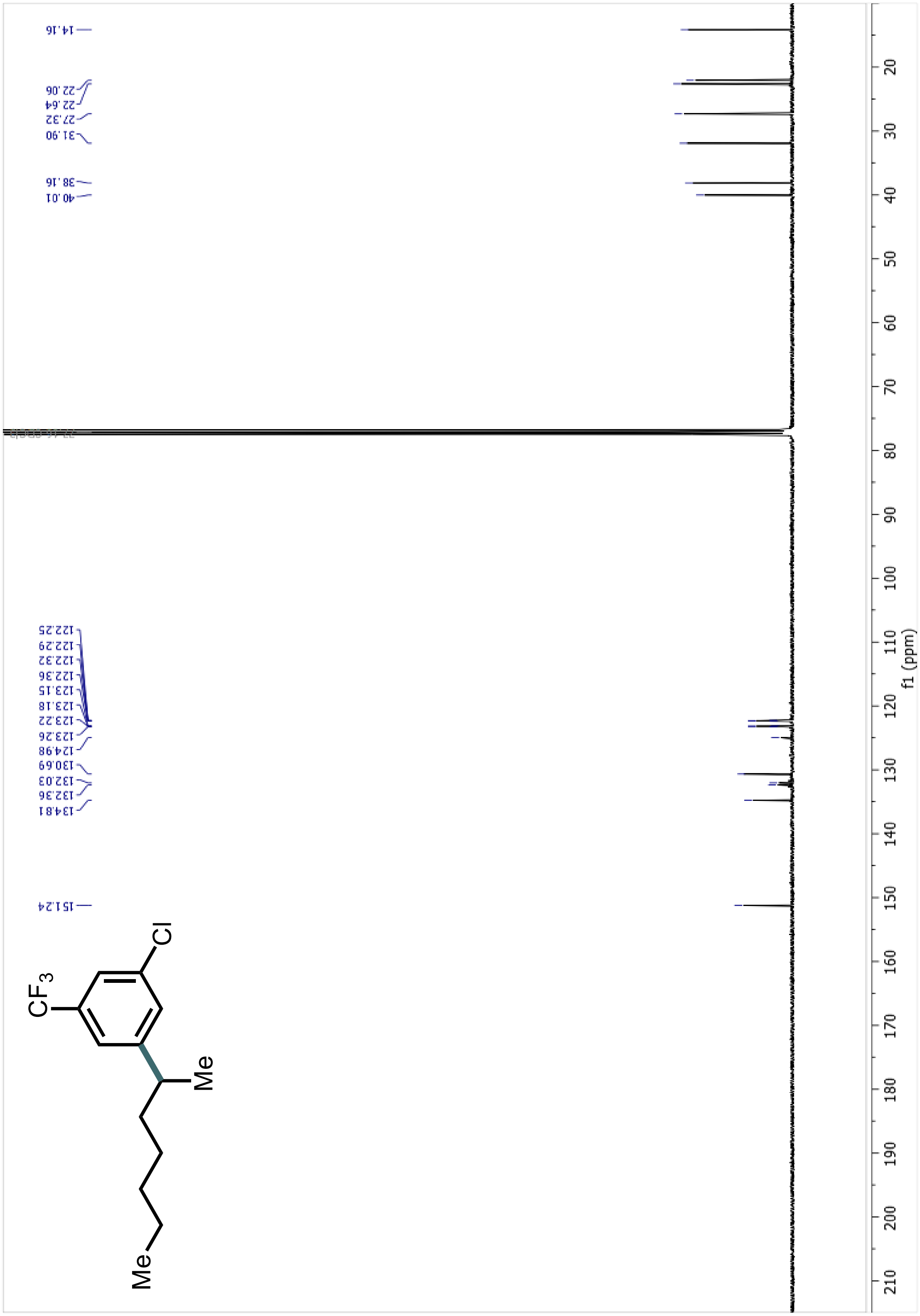

^{19}F NMR SPECTRUM OF **36** (377 MHz, $CDCl_3$):

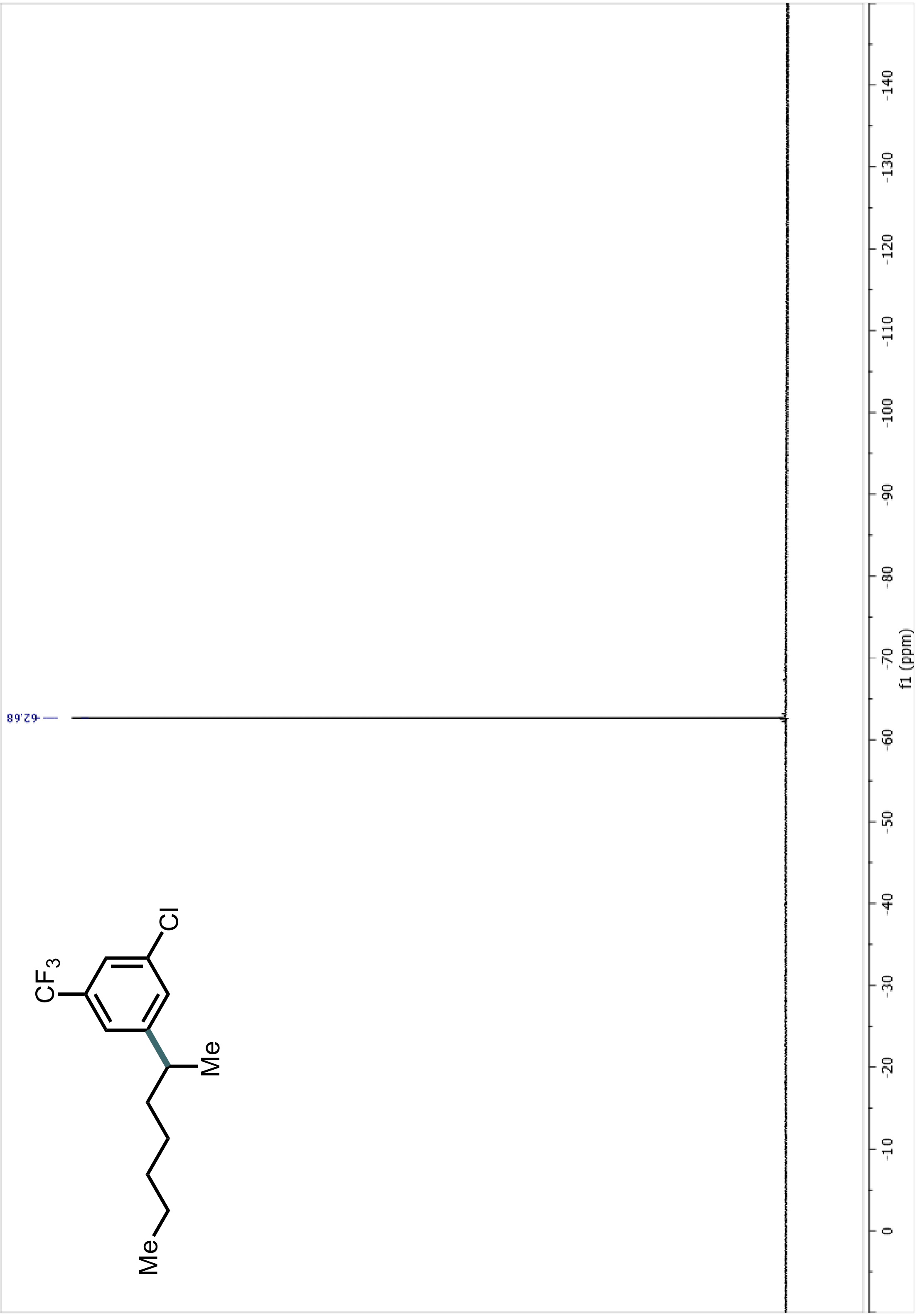

^{1}H NMR SPECTRUM OF **37** (400 MHz, $CDCl_3$):

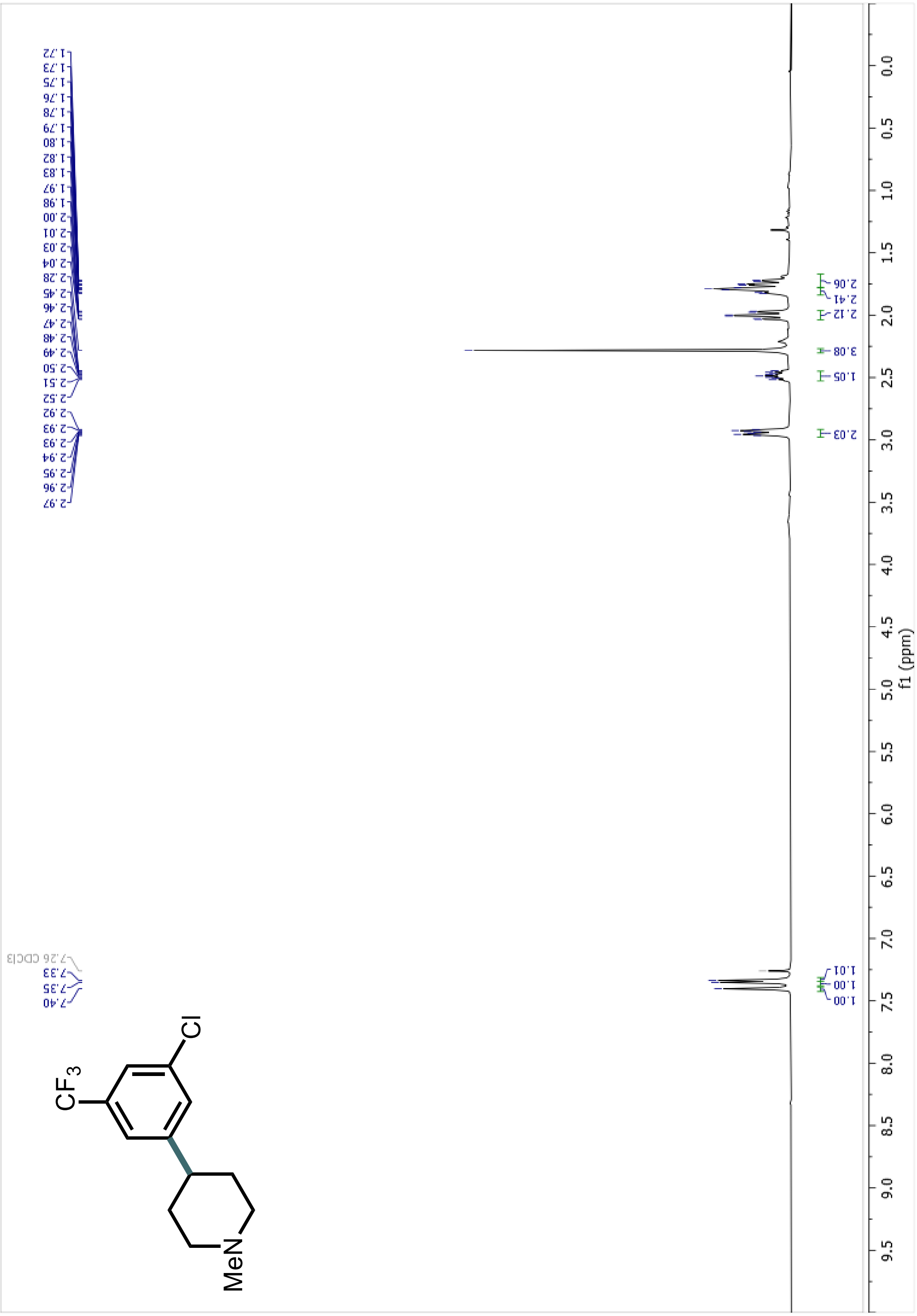

$^{13}C\{^{1}H\}$ NMR SPECTRUM OF **37** (201 MHz, $CDCl_3$):

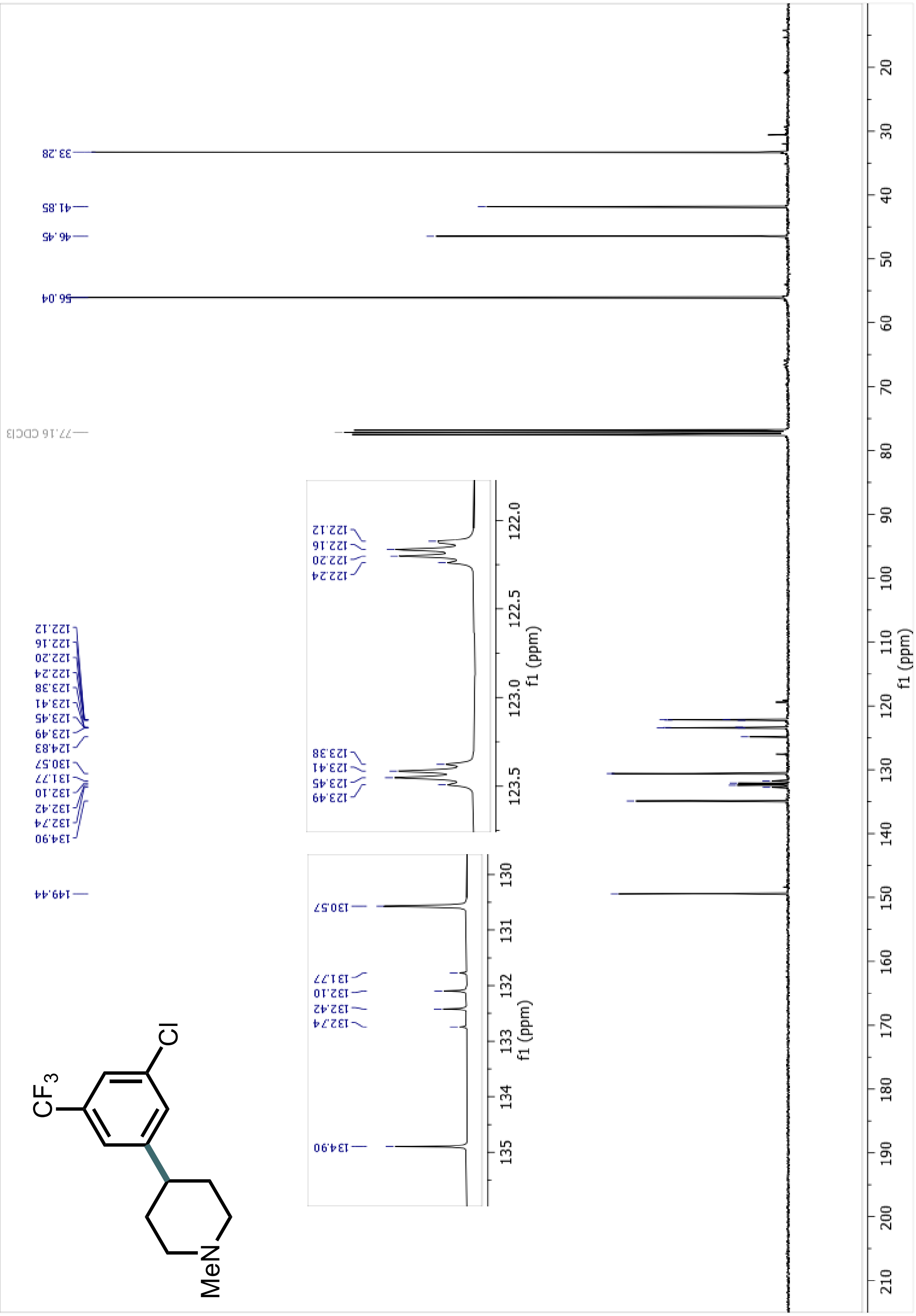

^{19}F NMR SPECTRUM OF **37** (377 MHz, $CDCl_3$):

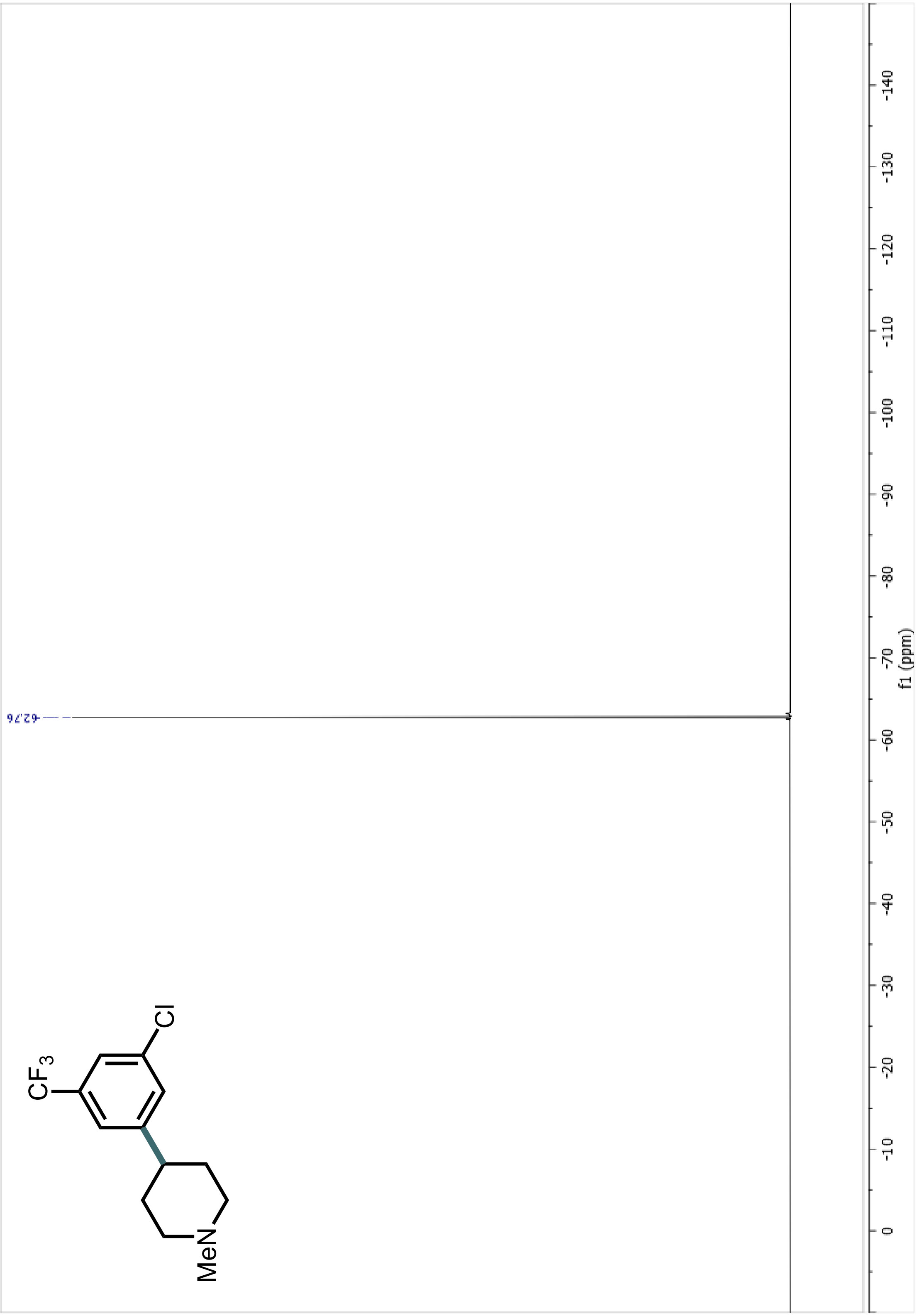

^{1}H NMR SPECTRUM OF **38** (400 MHz, $CDCl_3$):

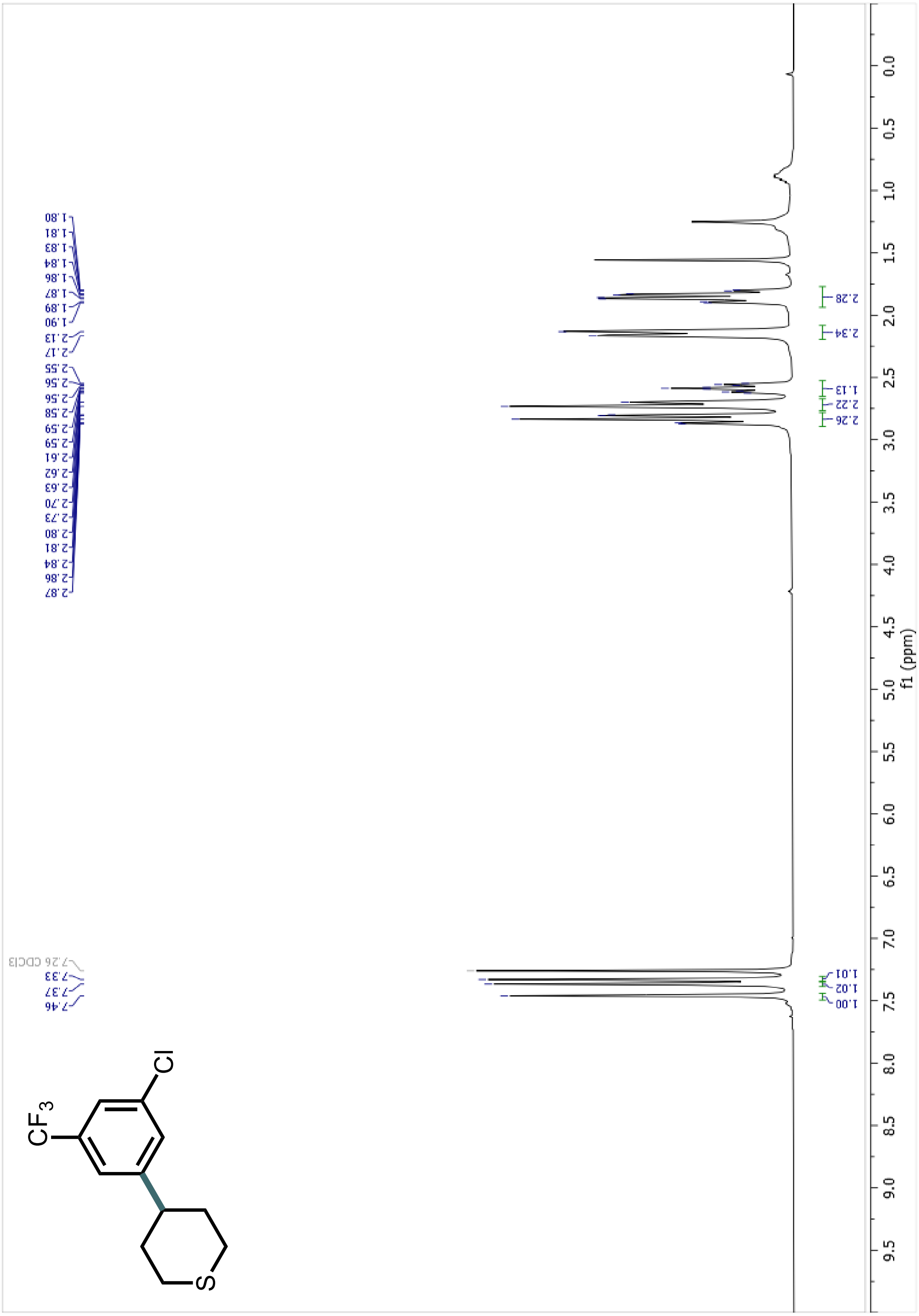

$^{13}C\{^{1}H\}$ NMR SPECTRUM OF **38** (201 MHz, $CDCl_3$):

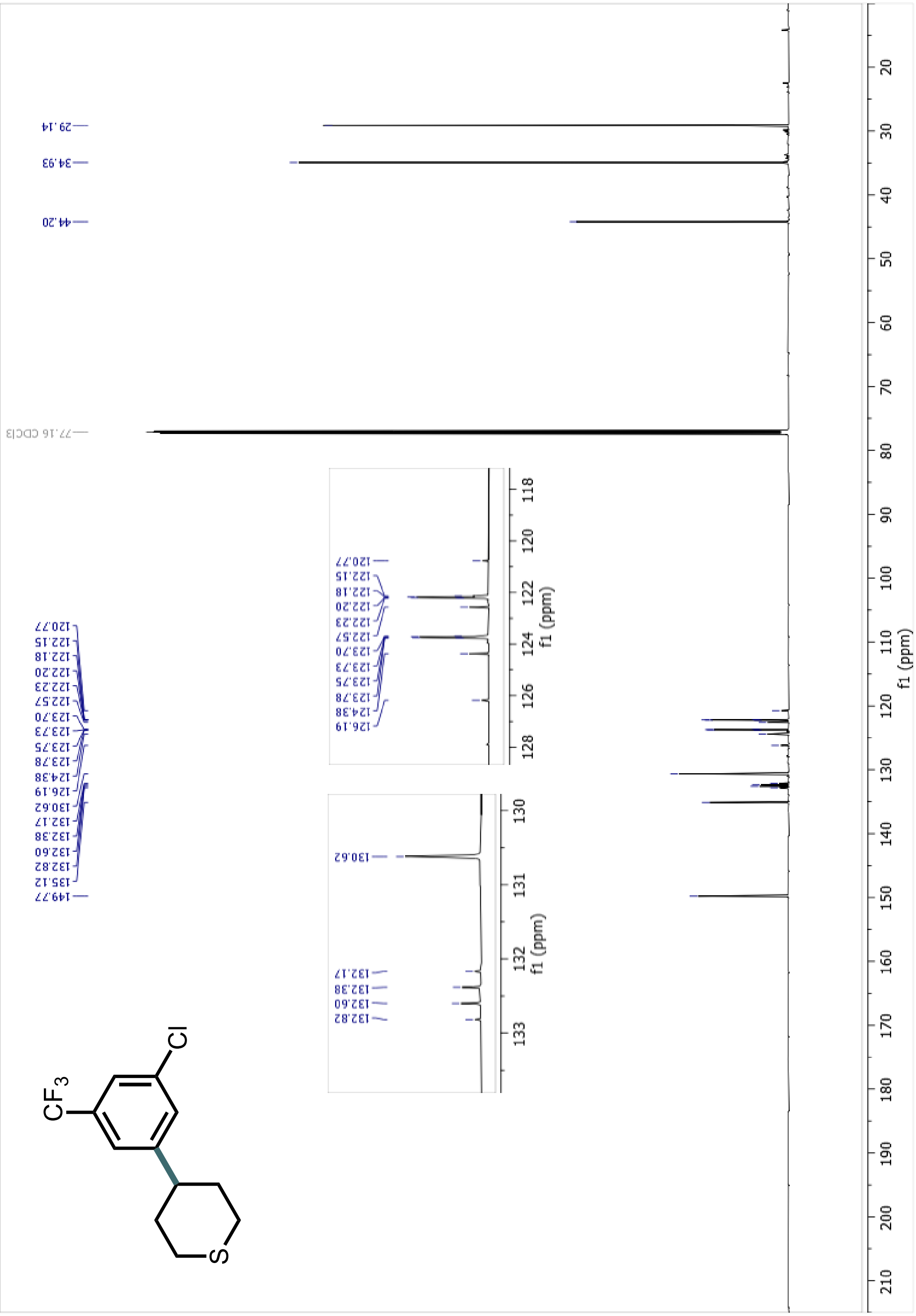

^{19}F NMR SPECTRUM OF **38** (377 MHz, $CDCl_3$):

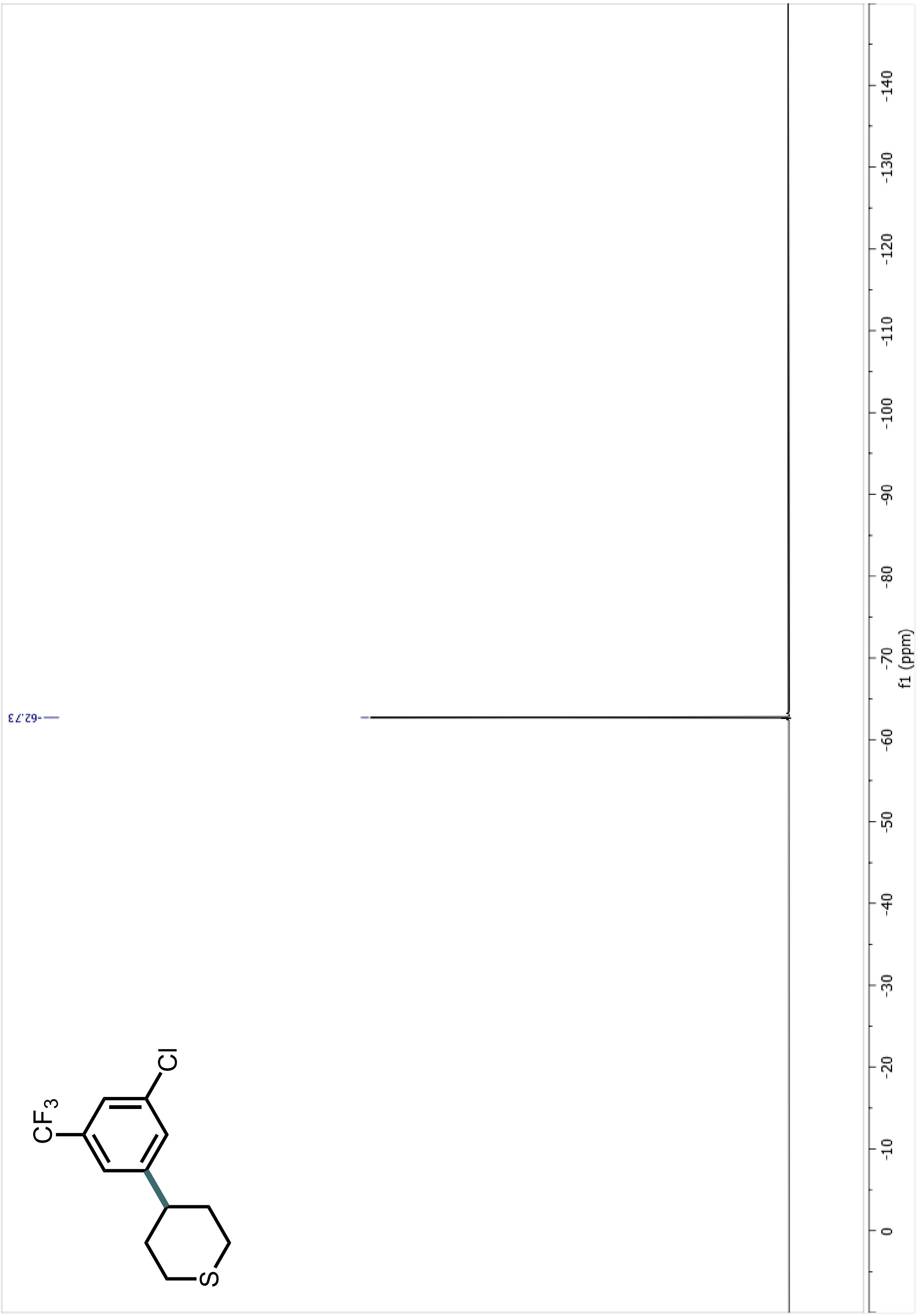

^{1}H NMR SPECTRUM OF **39** MAJOR DIASTEREOMER (400 MHz, $CDCl_3$):

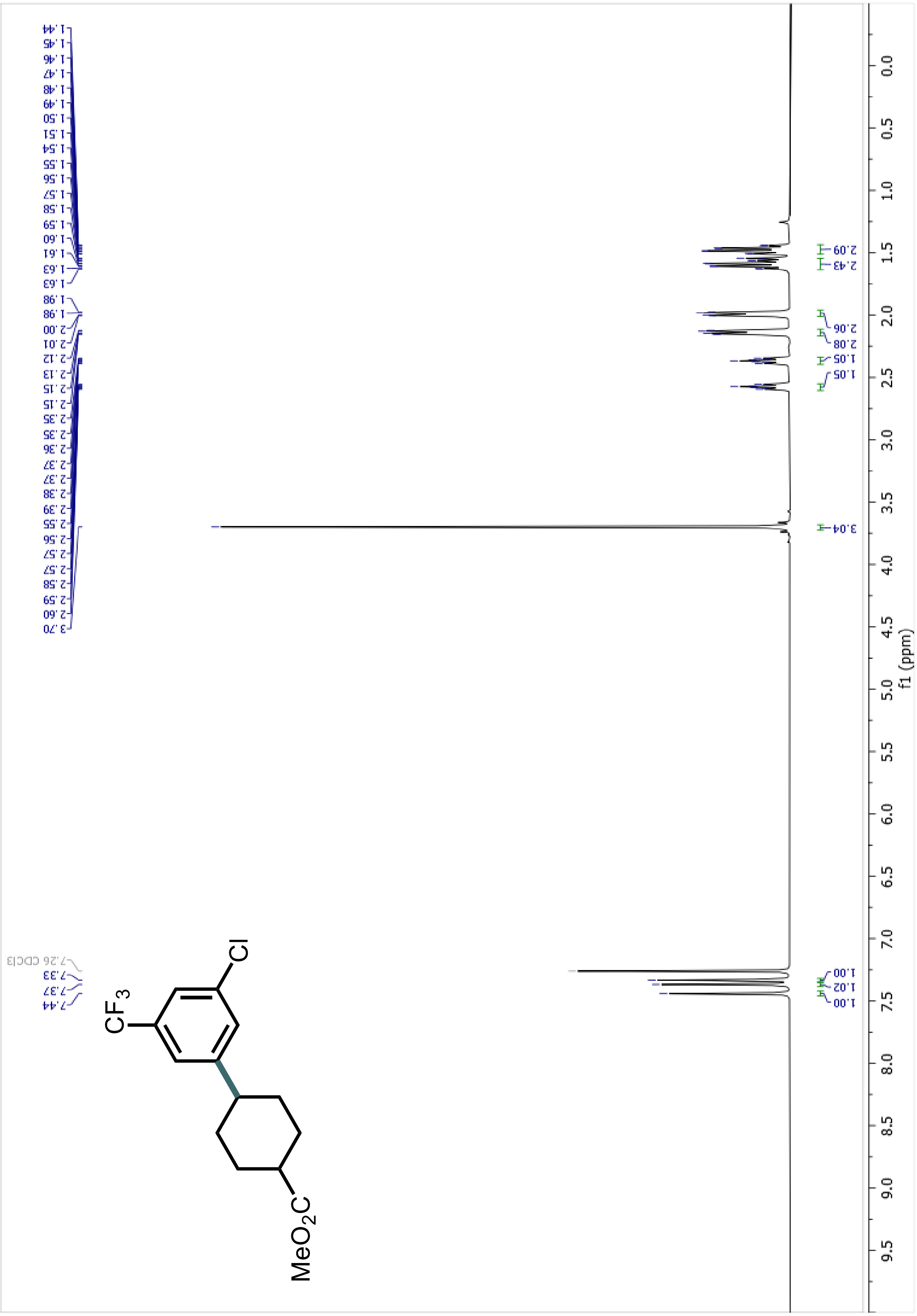

$^{13}C\{^{1}H\}$ NMR SPECTRUM OF **39** MAJOR DIASTEREOMER (201 MHz, $CDCl_3$):

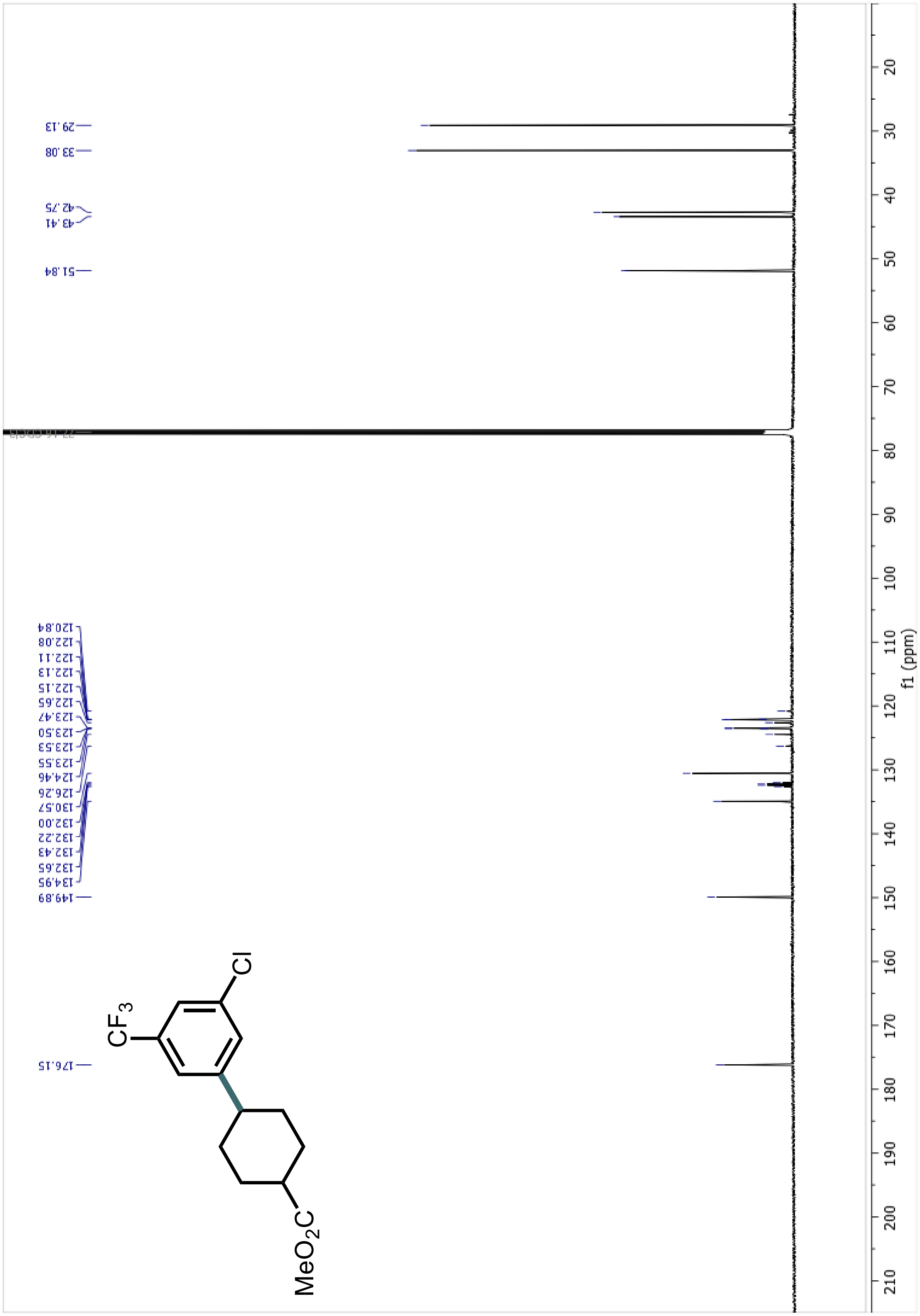

^{19}F NMR SPECTRUM OF **39** MAJOR DIASTEREOMER (377 MHz, $CDCl_3$):

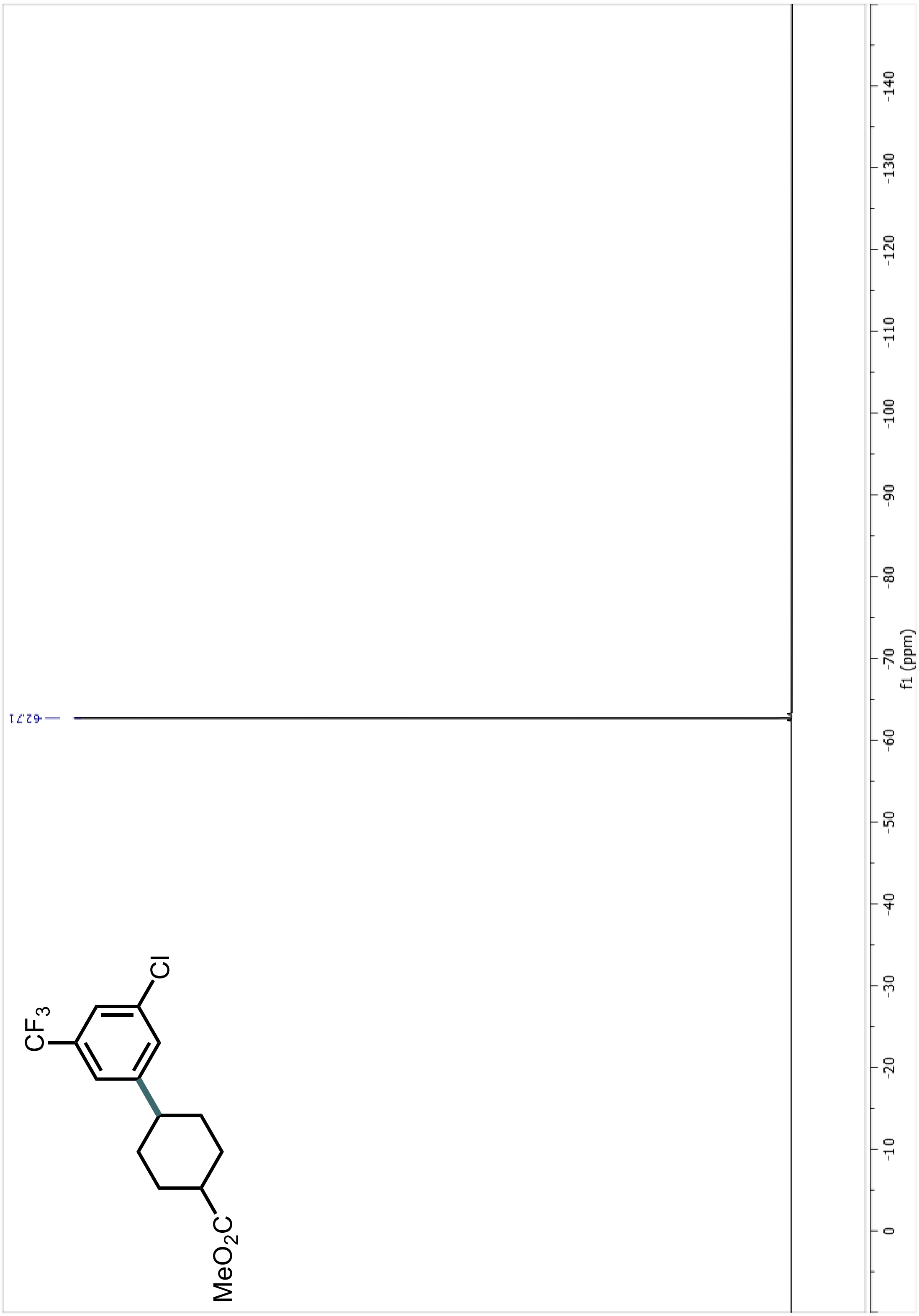

^{1}H NMR SPECTRUM OF **39** MINOR DIASTEREOMER (400 MHz, $CDCl_3$):

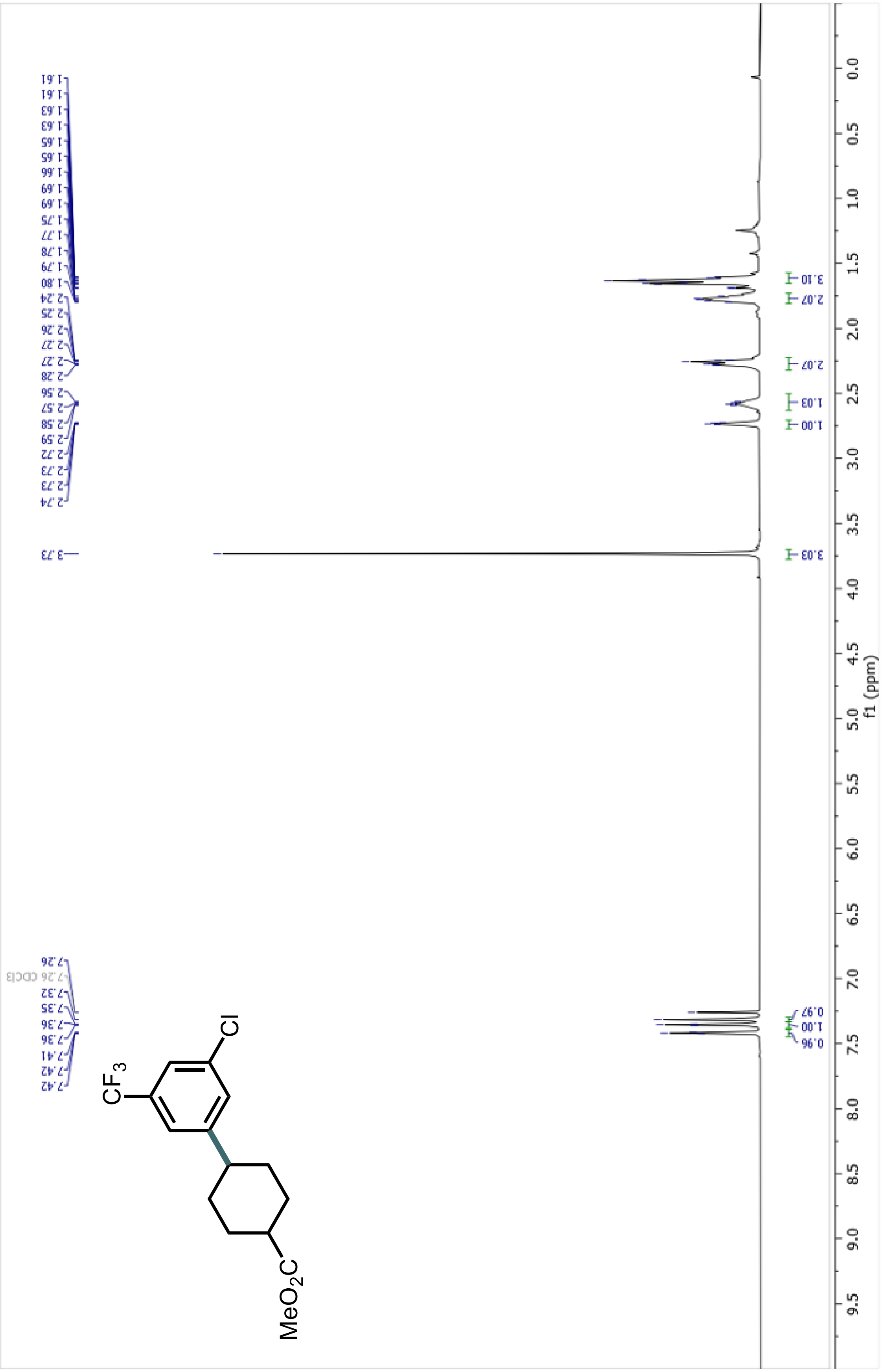

$^{13}C\{^{1}H\}$ NMR SPECTRUM OF **39** MINOR DIASTEREOMER (201 MHz, $CDCl_3$):

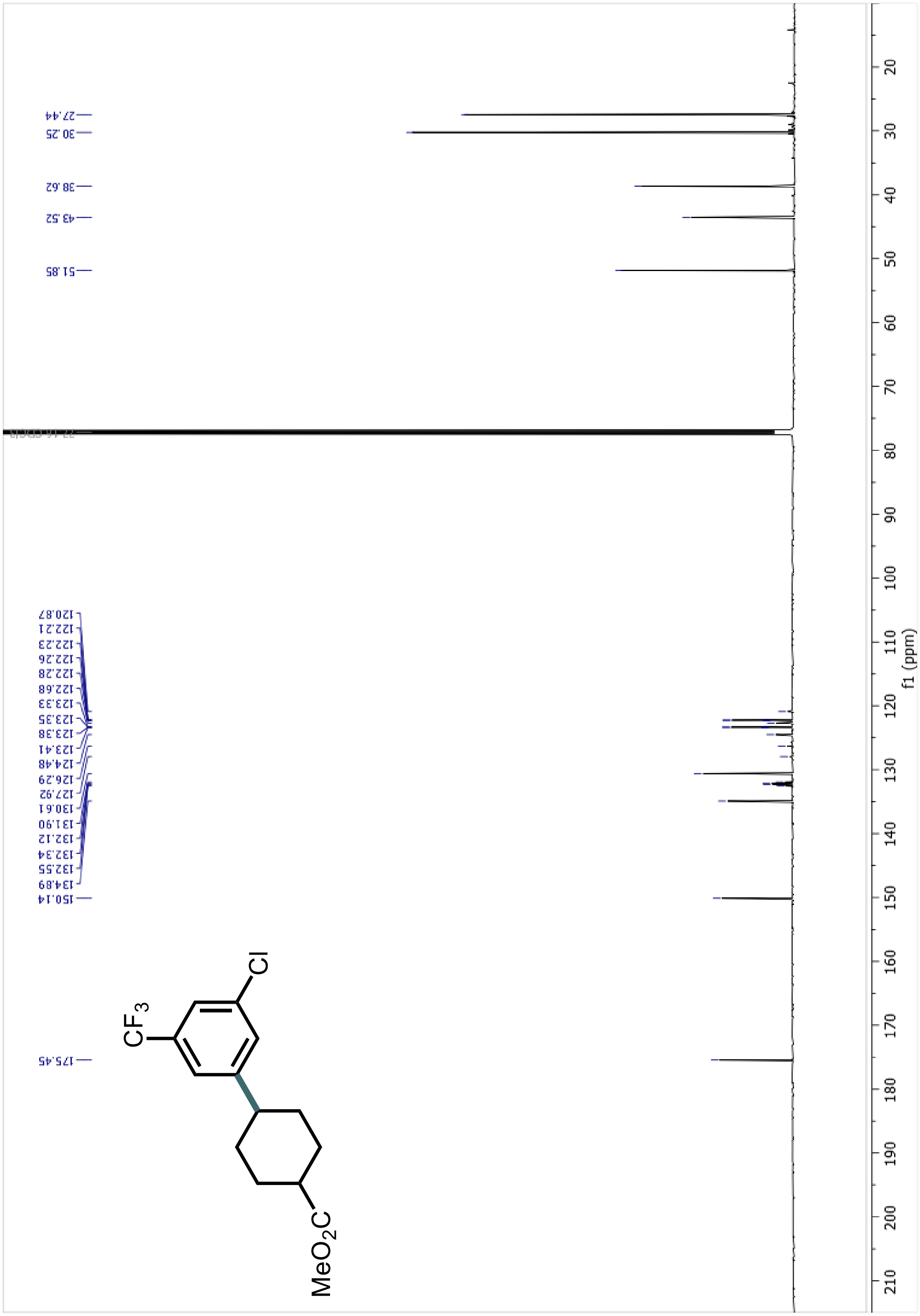

^{19}F NMR SPECTRUM OF **39** MINOR DIASTEREOMER (377 MHz, $CDCl_3$):

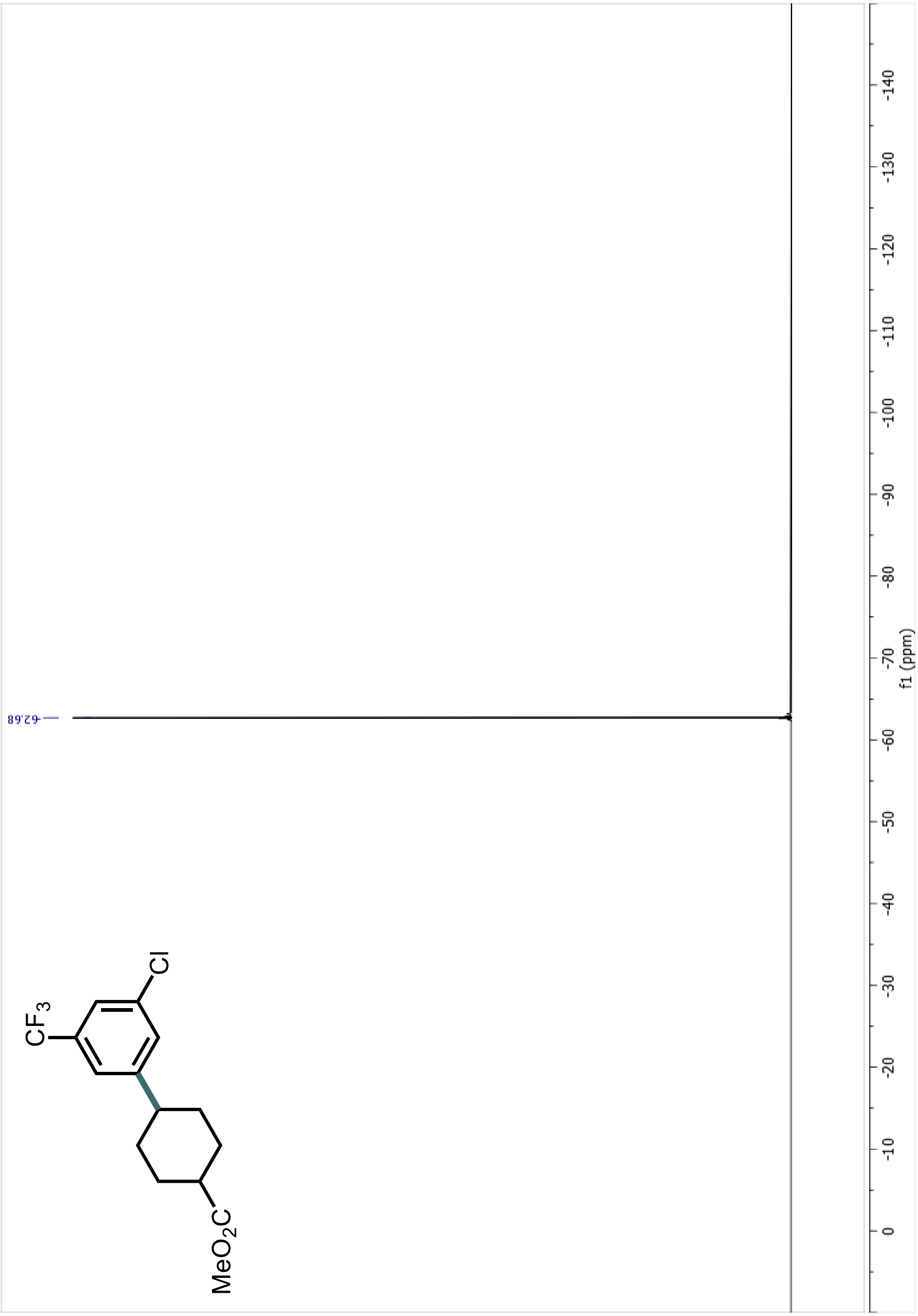

^{1}H NMR SPECTRUM OF **40** (400 MHz, CDCl3):

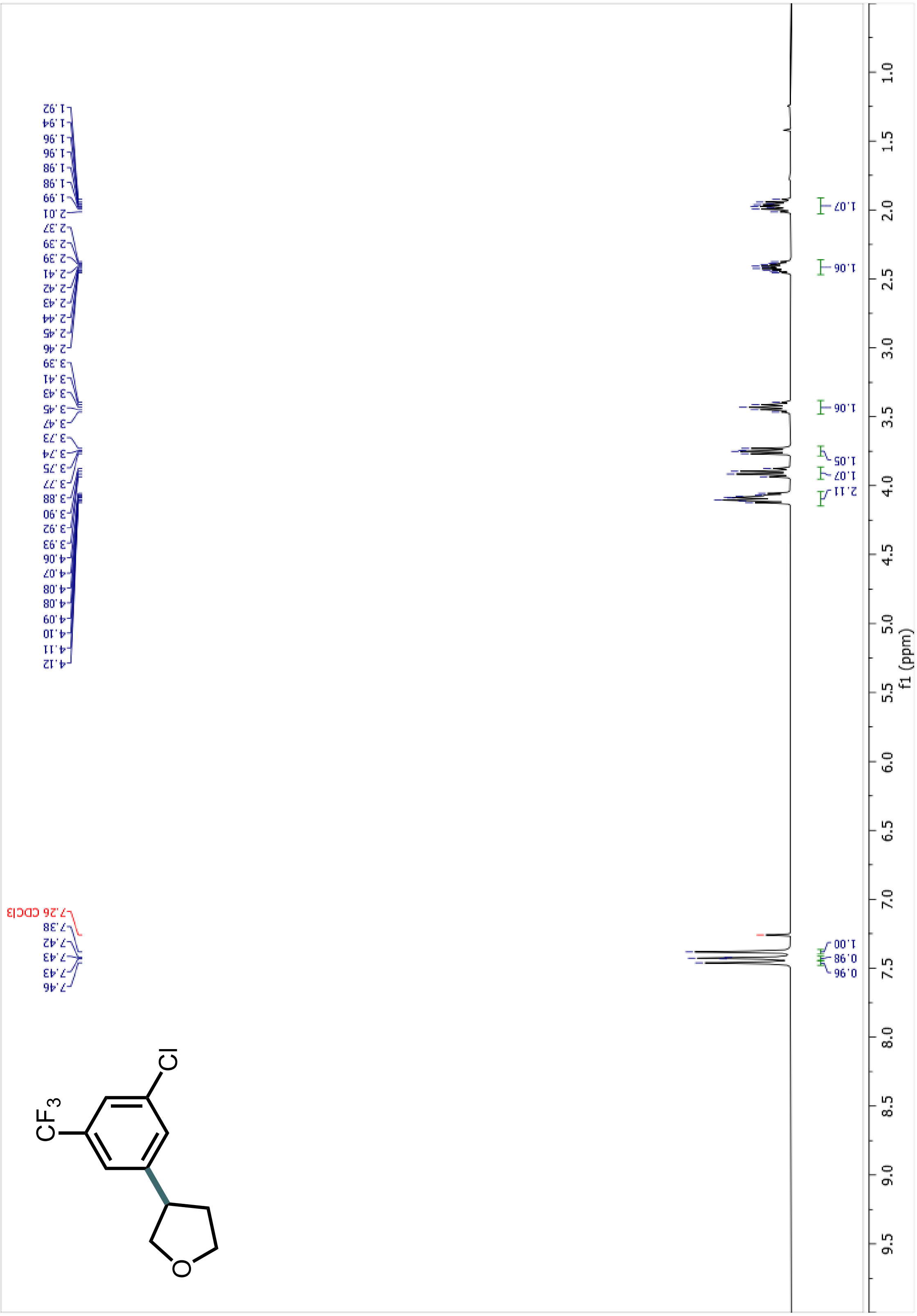

$^{13}C\{^{1}H\}$ NMR SPECTRUM OF **40** (101 MHz, $CDCl_3$):

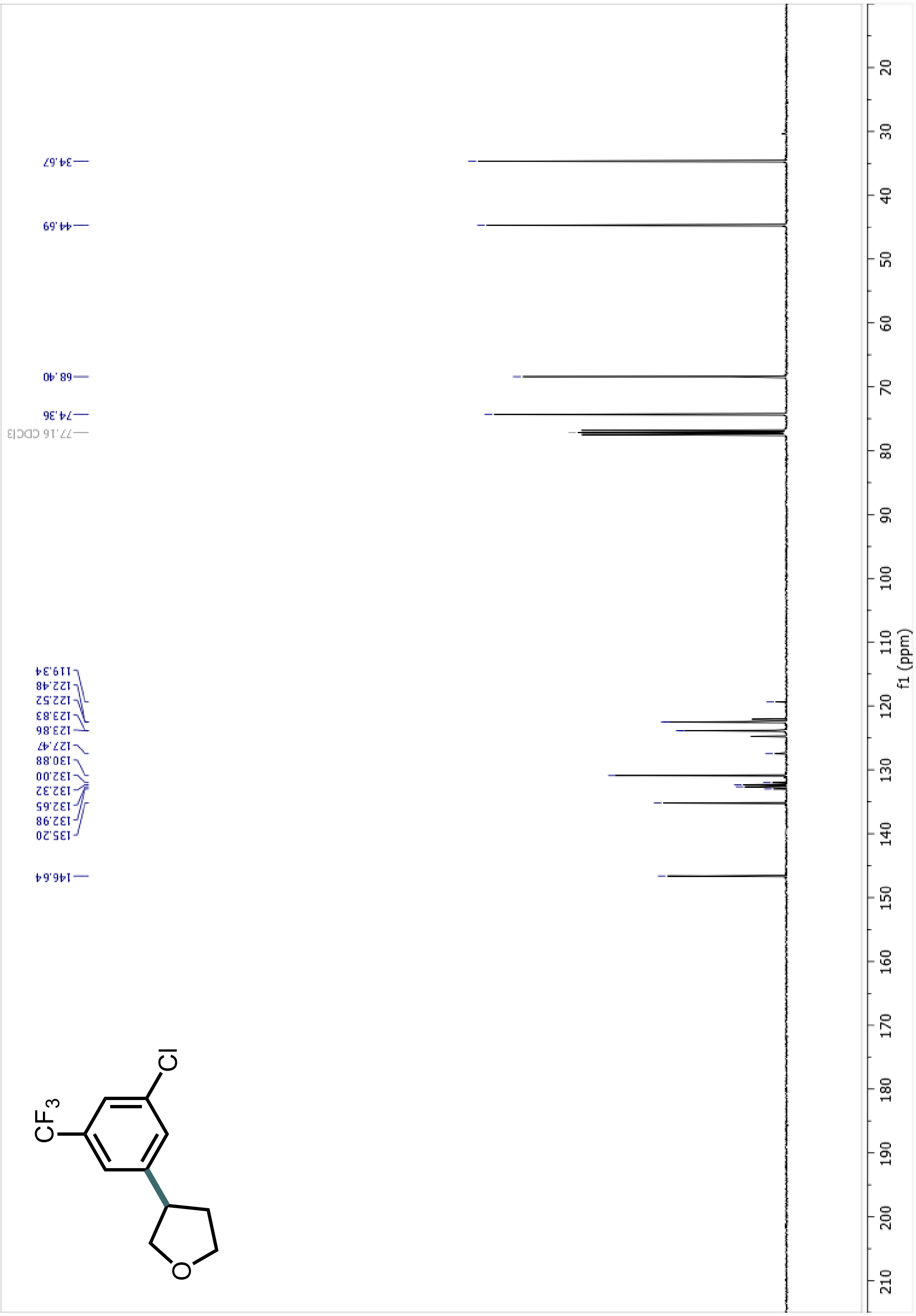

^{19}F NMR SPECTRUM OF **40** (377 MHz, $CDCl_3$):

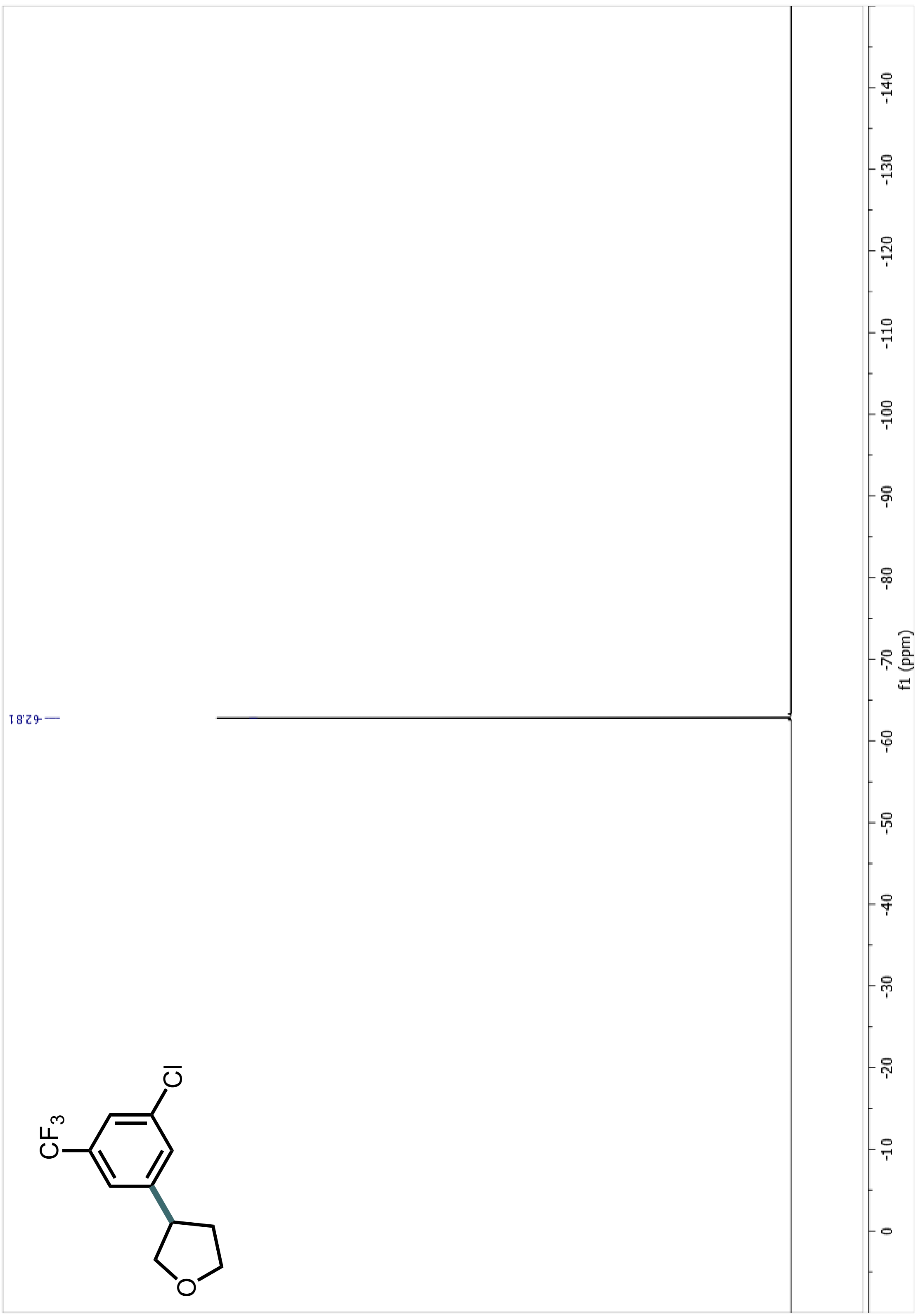

^{1}H NMR SPECTRUM OF ($^{4\text{-}t\text{Bu}}$BPY)NI(2-NAPHTHYLENYLMETHYL)(PHTHALIMIDO) (500 MHz, CD_3CN) **46**:

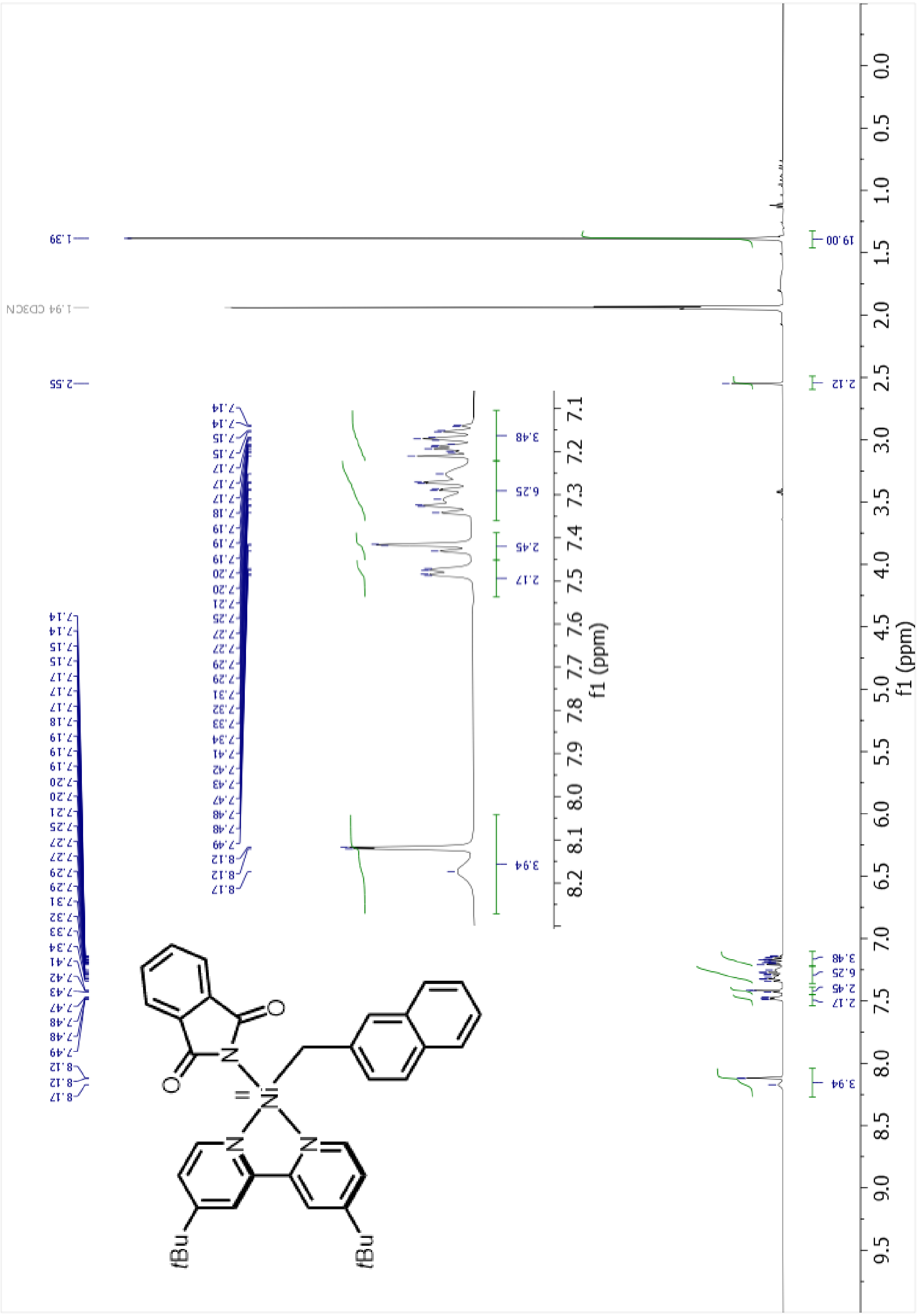

$^{13}C\{^1H\}$ NMR SPECTRUM OF ($^{4\text{-}tBu}$BPY)NI(2-NAPHTHYLENYLMETHYL)(PHTHALIMIDO) **46** (126 MHz, $CDCl_3$):

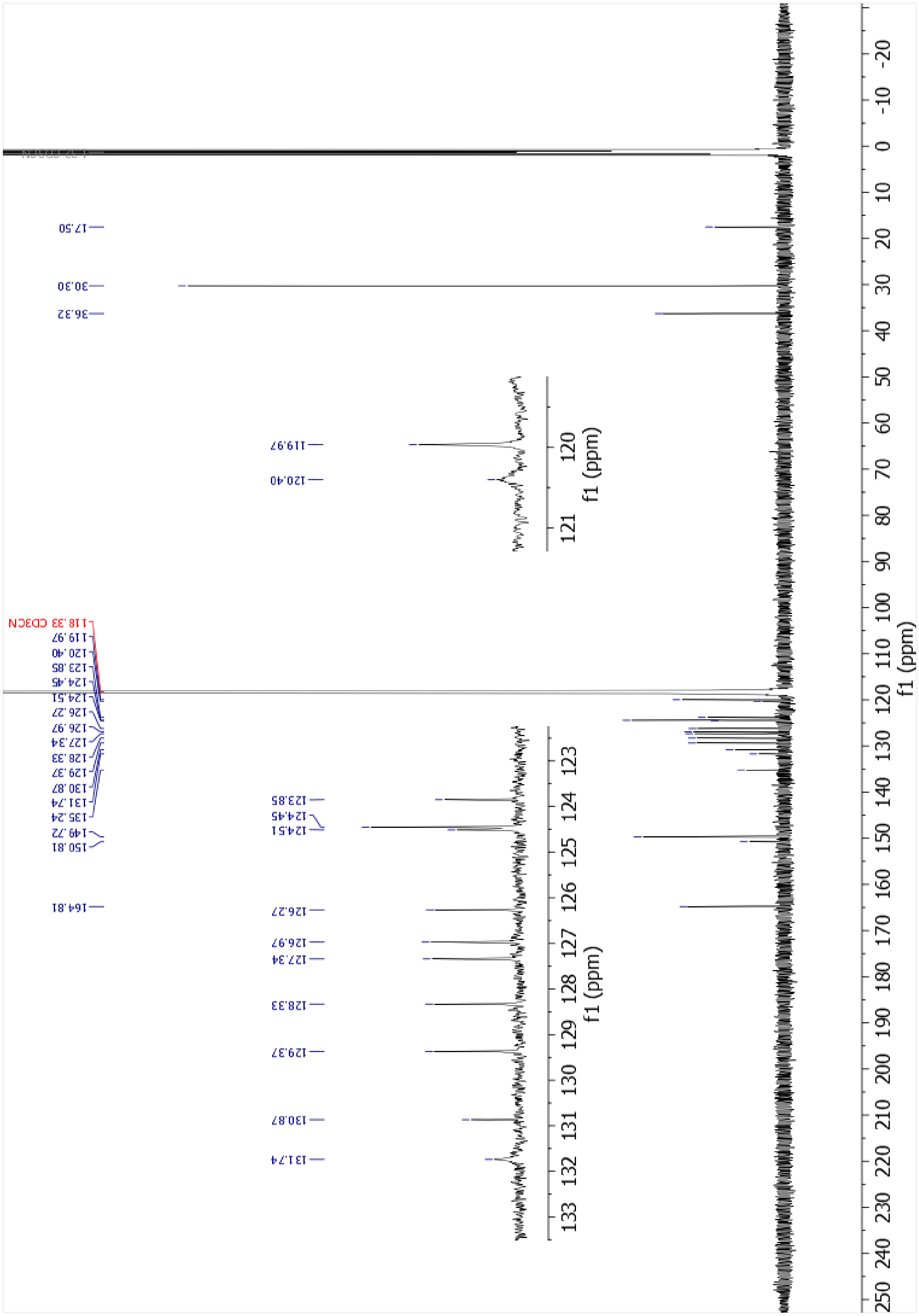

^{1}H-^{13}C HSQC of ($^{4\text{-}tBu}$bpy)Ni(2-naphthylenylmethyl)(phthalimido) **46**:

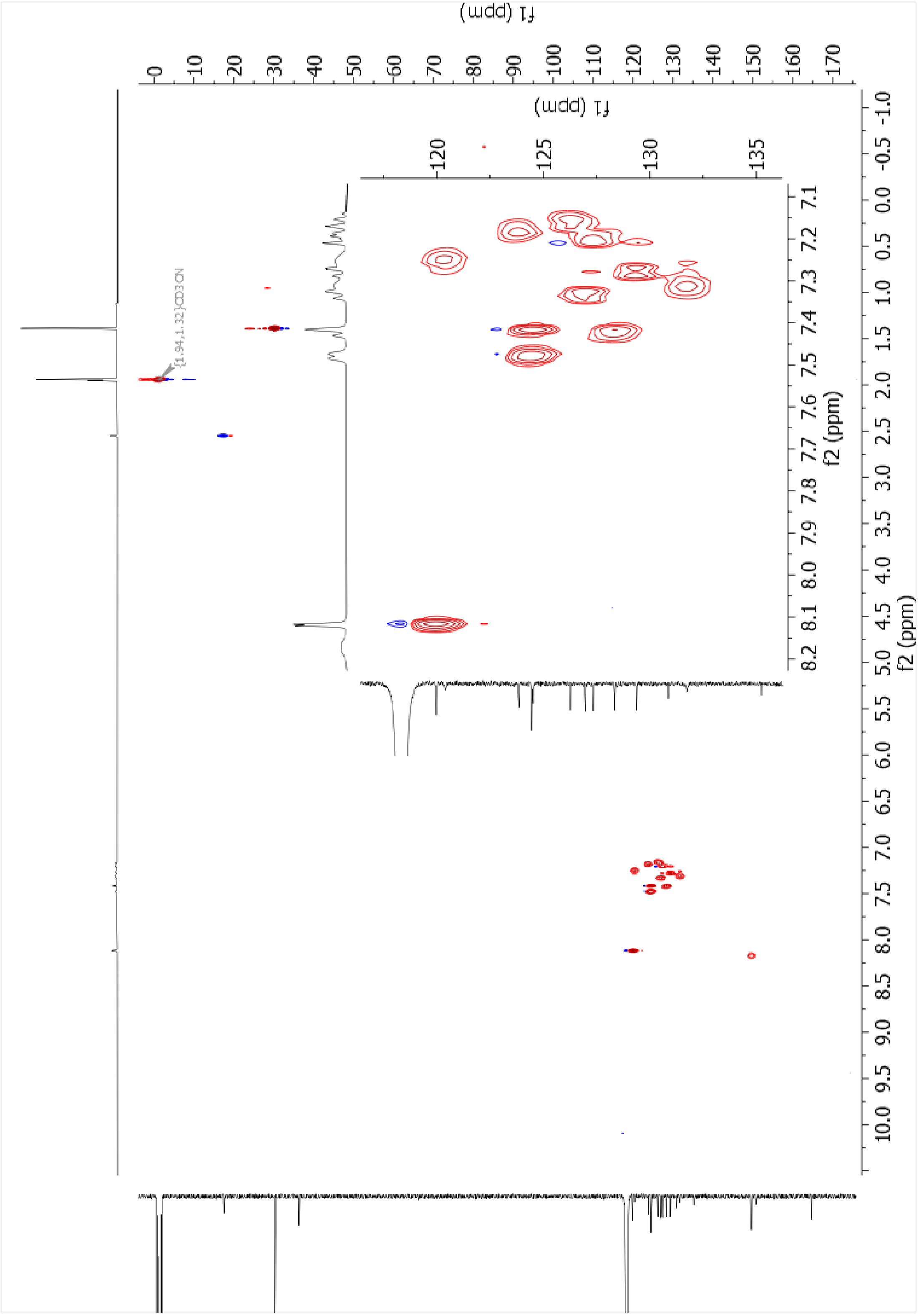

Supplement: Supplementary file 1 — ja4c09621_si_001.pdf [file ja4c09621_si_001.pdf]
